# Guideline adherence and expert-rated potential safety concerns in AI-generated dietary advice for patients with cirrhosis: a comparative evaluation of large language models

**DOI:** 10.3389/fnut.2026.1893597

**Published:** 2026-10-08

**Authors:** Qiong Liu, Guojun Liang, Xiaofang Liu, Yang Song, Hongsheng Wu

**Affiliations:** Huadu District People’s Hospital of Guangzhou, Guangzhou, Guangdong, China

**Keywords:** cirrhosis, guideline adherence, large language models, nutritional counseling, potential safety concerns

## Abstract

**Background:**

Patients with cirrhosis require complication-specific nutritional counseling. Large language models are increasingly used for health information seeking, but the guideline adherence and potential safety concerns of their dietary advice for cirrhosis remain uncertain.

**Methods:**

Thirty-six standardized simulated cases represented compensated cirrhosis, cirrhosis with ascites, and hepatic encephalopathy recovery. Each case was submitted once to DeepSeek, ChatGPT, Qwen, and Claude through their official web interfaces, generating 144 English-language recommendations. Three reviewers performed *de novo* model-blinded assessments using a locked framework. The primary outcome was response-level Guideline Adherence; secondary outcomes included Error Count, Any Major Error, and Harm Flag.

**Results:**

Mean Guideline Adherence was 7.00 ± 0.00 for ChatGPT, 6.97 ± 0.17 for Claude, 6.94 ± 0.19 for DeepSeek, and 6.44 ± 0.84 for Qwen (Friedman *p* < 0.001). In hepatic encephalopathy recovery, Qwen scored 5.36 ± 0.54 and was lower than each other model after Holm adjustment (all adjusted *p* = 0.009). Any Major Error occurred in 0 ChatGPT, 2 Claude, 4 DeepSeek, and 11 Qwen responses; corresponding Harm Flag counts were 0, 1, 2, and 8. Most events occurred during hepatic encephalopathy recovery and involved protein-related recommendations.

**Conclusion:**

Under standardized English-language structured prompting, all four models generally reproduced core cirrhosis nutrition principles. However, Qwen showed lower adherence and more frequent protein-related errors in hepatic encephalopathy recovery. Expert verification remains necessary before patient-facing or individualized use.

## 1 Introduction

Cirrhosis represents the advanced stage of chronic liver disease and remains a major contributor to global morbidity and mortality. In addition to portal hypertension, impaired hepatic function, and other systemic complications, cirrhosis is frequently accompanied by malnutrition, sarcopenia, and frailty (1, 2). These abnormalities arise from reduced dietary intake, altered substrate metabolism, inflammation, malabsorption, and hypermetabolism, and become more prevalent as liver disease progresses (3). Previous studies have reported that malnutrition occurs in approximately 20% of patients with compensated cirrhosis and in more than half of patients with decompensated disease. Sarcopenia is independently associated with hepatic decompensation, infection, hospitalization, and mortality (4, 5). Nutritional impairment is therefore a clinically relevant and potentially modifiable component of cirrhosis care.

Given the high prevalence and clinical consequences of nutritional impairment, nutritional management has become an essential component of comprehensive cirrhosis care. International guidelines recommend adequate energy intake, dietary protein of approximately 1.2 to 1.5 g/kg/day, avoidance of prolonged fasting, small frequent meals, and a late-evening snack (2, 3). Dietary management is further complicated by disease-specific complications that require individualized strategies. Sodium restriction is commonly required in ascites, whereas routine or prolonged protein restriction is not recommended in hepatic encephalopathy because it may aggravate muscle loss and impair extrahepatic ammonia disposal (6–8). Effective counseling must therefore combine general nutritional principles with the patient’s complication profile and nutritional risk.

Despite the availability of guideline-based recommendations, translating nutritional principles into practical dietary advice for individual patients remains challenging in routine clinical practice. Access to specialist dietitians is limited in many settings, and time constraints may restrict the depth of nutritional counseling during clinical encounters (9, 10). Consequently, patients frequently seek additional information from external sources when attempting to modify their diet or lifestyle (10). Websites, mobile applications, and conversational artificial intelligence systems have expanded the range of patient-facing information sources (11–13). Large language models can rapidly generate detailed natural-language responses to questions about disease management, dietary restrictions, and lifestyle modification, making them readily accessible tools for health information seeking (12, 13).

However, the growing use of artificial intelligence systems for health information seeking raises concerns regarding the accuracy and potential clinical consequences of their recommendations. Large language model outputs are probabilistically generated and can contain omissions, outdated recommendations, or advice that is inconsistent with the stated clinical context. A physician-led evaluation of patient-posed medical questions identified problematic and unsafe responses across several publicly available chatbots, while broader reviews have reported marked variation according to model, task, and prompting conditions (14–16). In cirrhosis, a response that appears comprehensive may still be clinically consequential if it recommends protein restriction during recovery from hepatic encephalopathy, fails to address sodium intake in ascites, or advises fluid restriction without an appropriate indication.

Recent investigations of large language models in healthcare have examined patient education, medical question answering, and other clinical communication tasks. Safe patient-oriented recommendations require not only factual knowledge but also the ability to integrate guideline recommendations with the clinical scenario (17). In hepatology, previous studies have assessed ChatGPT responses to general questions about cirrhosis and hepatocellular carcinoma and have compared artificial intelligence-generated with human-authored cirrhosis education materials (18, 19). These studies demonstrated the potential of large language models to communicate liver-related information but also identified incomplete answers, missing decision thresholds, and variable actionability. A nutrition-focused evaluation in metabolic dysfunction-associated steatotic liver disease similarly found that artificial intelligence-generated meal plans approximated some energy targets while omitting or misaligning important guideline components (20). Systematic evaluation of artificial intelligence-generated dietary advice for cirrhosis remains limited, and no previous study has compared multiple models using scenario-specific cirrhosis nutrition checklists together with separate expert-rated outcomes for potential safety concerns.

Therefore, this study aimed to compare guideline adherence and expert-rated potential safety concerns in dietary recommendations generated by ChatGPT, DeepSeek, Qwen, and Claude. We used 36 standardized simulated cases representing compensated cirrhosis, cirrhosis with ascites, and recovery from hepatic encephalopathy. Model outputs underwent *de novo* model-blinded assessment using a locked scenario-specific framework. The primary outcome was the response-level Guideline Adherence score. Secondary outcomes included predefined major dietary errors, Error Count, Any Major Error, and Harm Flag, while response length was examined as an exploratory variable. The study was designed to characterize performance under standardized prompting conditions and to identify complication-specific recommendations that may require professional verification before patient-facing use.

## 2 Methods

### 2.1 Study design

This study was designed as a cross-sectional expert evaluation of guideline adherence and expert-rated potential safety concerns in dietary recommendations generated by large language models for patients with cirrhosis. Reporting followed the Chatbot Assessment Reporting Tool (CHART) statement (21). TRIPOD-LLM was consulted as a complementary reporting reference (22). The study did not involve human participants or identifiable patient data; therefore, ethics committee review and informed consent were not required.

The overall workflow consisted of guideline review, simulated case construction, multidisciplinary development of the evaluation framework, large language model response generation, response normalization and anonymization, *de novo* model-blinded expert reassessment, structured scoring, and statistical analysis. The primary outcome was the Guideline Adherence score. Secondary outcomes included Error Count, individual predefined major dietary errors, Any Major Error, and Harm Flag. Clinically Appropriate Food Examples was summarized descriptively, and response length was recorded as an exploratory variable (Figure 1).

**Figure 1:**
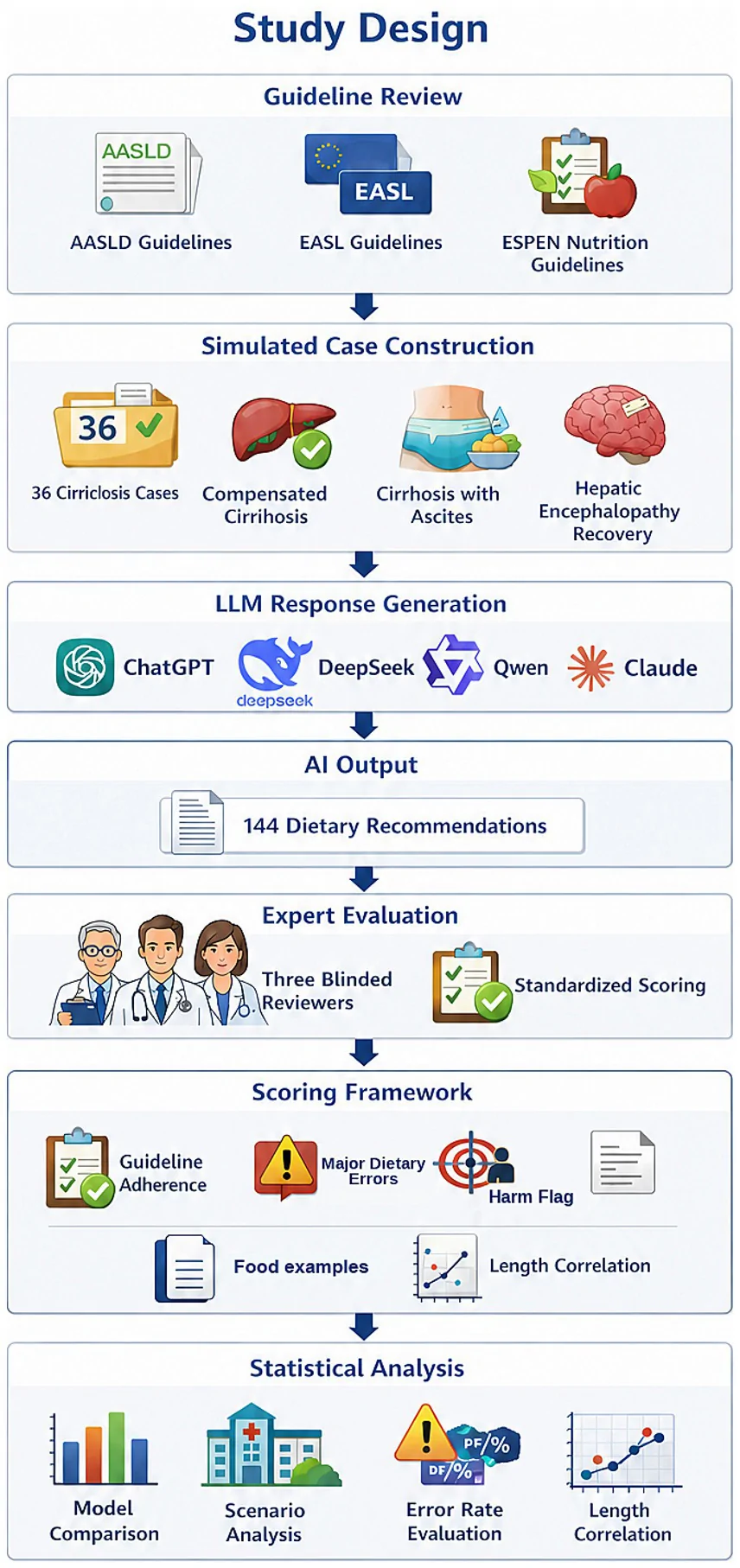
Study workflow. Thirty six simulated cases were submitted to four large language models, generating 144 responses. Three model-blinded reviewers independently assessed each response, yielding 432 reviewer-level records. Main outcomes were guideline adherence and expert-rated potential safety concerns.

The response-level unit of analysis was one simulated case submitted to one model. A total of 36 simulated cases were submitted to four large language models, yielding 144 model-generated dietary recommendations. Each response was independently rescored by three senior clinical reviewers using the locked scoring framework, resulting in 432 reviewer-level scoring records. Model comparisons were based on aggregated response-level data, while reviewer-level records were retained for inter-reviewer reliability analysis.

### 2.2 Guideline review and evaluation framework development

A structured guideline review was performed to develop the evaluation framework. The primary reference sources were international clinical nutrition and hepatology guidelines relevant to cirrhosis management, including recommendations from the European Society for Clinical Nutrition and Metabolism, the European Association for the Study of the Liver, and the American Association for the Study of Liver Diseases (2–4). These guidelines were used to identify core dietary principles for patients with cirrhosis, including adequate energy intake, sufficient dietary protein, avoidance of prolonged fasting, small frequent meals, late-evening snacks, sodium restriction in ascites, and appropriate protein management during recovery from hepatic encephalopathy. Recommendations on fluid restriction, nutritional monitoring, malnutrition, and sarcopenia were also considered.

The simulated cases and evaluation framework were developed by a multidisciplinary clinical team. The team included clinicians from hepatobiliary surgery, gastrointestinal surgery, gastroenterology, internal medicine, and clinical nutrition. The case content, checklist domains, and major evaluation constructs were reviewed with reference to the selected guidelines. Revisions were made until agreement was reached within the team.

The guideline review informed the construction of a scenario-specific scoring system. Because dietary priorities differ across cirrhosis stages and complications, a single universal checklist was not used. Instead, each simulated clinical scenario had its own seven-item guideline adherence checklist. In addition, a set of major dietary error categories was developed to capture potentially unsafe or clinically inappropriate recommendations (2, 3, 6). A binary Harm Flag was added as a separate expert-rated outcome for potential safety concerns to identify responses that could plausibly cause clinically relevant harm if followed without professional correction. The broad evaluation domains and scenario-specific checklist structure were established during study development. After all model responses had been generated, the detailed operational scoring definitions were refined before the final *de novo* model-blinded assessment to clarify protein requirements, acceptable energy recommendations, vague statements, conditional advice, the application of individual error categories, and the distinction between guideline nonadherence and potential harm. The final scoring manual and codebook were then locked before this assessment began, and no operational definition or decision rule was modified thereafter. The finalized manual is provided in Supplementary File A (Locked Codebook). No formal pilot study or independent external validation was performed.

### 2.3 Simulated case construction

Thirty-six standardized simulated cases were constructed to represent common outpatient or post-discharge nutritional counseling scenarios in cirrhosis. Each case contained demographic and clinical information that would commonly be available during a nutritional consultation, including age, sex, height, body weight, etiology of cirrhosis, and relevant complication status. The cases were presented as concise standardized consultation vignettes while providing sufficient clinical context for dietary recommendation generation. The sample size was determined pragmatically to provide equal representation of the three clinical scenarios while maintaining the feasibility of complete paired evaluation across four models and three reviewers. Formal Child-Pugh and MELD scores were not included. Within each scenario, cases followed a common information structure to support comparability while varying in demographic, anthropometric, etiological, and selected clinical features.

The cases were divided into three predefined clinical scenarios, with 12 cases in each scenario. The first scenario represented compensated cirrhosis, defined as stable outpatient cirrhosis without ascites or hepatic encephalopathy. The second represented cirrhosis with ascites, defined as stable outpatient or post-discharge cirrhosis complicated by ascites, with oral intake possible. The third represented hepatic encephalopathy recovery, defined as previous hepatic encephalopathy with clinical recovery and stability at the time of dietary counseling (Supplementary File C: Cases and Prompts).

The three scenario groups were designed to test different aspects of cirrhosis nutritional management. The compensated cirrhosis scenario assessed whether models could provide appropriate general nutritional advice for stable cirrhosis. The ascites scenario assessed whether models could integrate sodium restriction while maintaining adequate nutritional intake. The hepatic encephalopathy recovery scenario assessed whether models avoided inappropriate routine protein restriction and recognized the importance of maintaining adequate protein intake and nutritional status (2–4, 8).

### 2.4 Prompt design and large language model response generation

A standardized prompt was developed from the guideline domains and applied to all model interactions. It instructed the model to act as a physician specializing in clinical nutrition and to provide practical daily dietary recommendations for a patient with cirrhosis presenting for outpatient counseling. The prompt requested overall dietary principles, daily protein intake, meal frequency and eating pattern, specific food recommendations or restrictions, and additional case-relevant nutritional considerations. It was finalized before model testing and was not iteratively modified in response to model outputs.

Each simulated case was inserted into the same prompt structure. The same 36 cases were submitted independently to four publicly accessible LLM web platforms: ChatGPT (OpenAI), DeepSeek (DeepSeek), Qwen (Alibaba), and Claude (Anthropic). All four systems were accessed through their official consumer web interfaces, and the authors did not train, fine-tune, or otherwise modify the underlying models. Testing was conducted between May 1 and May 7, 2026, using free-tier accounts. The exact backend model deployment used for individual interactions could not be independently verified from the retained study records and was therefore treated as unknown. Accordingly, the evaluated systems were identified at the web-platform level rather than by specific backend model versions. Detailed platform and testing characteristics are provided in Supplementary Table S4. All prompts were submitted in English. Internet or web-search functionality and the reasoning or deep-thinking option available through each platform interface were enabled where available. Each platform generated one response for each case. No follow-up prompts, clarification questions, corrective feedback, or additional guideline documents were provided. The purpose was to evaluate the initial response generated under standardized single-prompt conditions.

The same account was used for all cases within each platform, and a new conversation was opened for every case. Memory, personalization, and custom instructions were not used. The cases were submitted in the fixed order C1-01 to C3-12. Temperature and other sampling parameters were not adjusted because these settings were not available through the web interfaces. No response was regenerated, replaced, or excluded. Across the four models, 144 dietary recommendations were generated. The outputs were copied from the model interfaces and stored in separate files. This single-generation design evaluated the initial response under standardized testing conditions, while within-system response variability across repeated submissions was beyond the scope of the present study.

### 2.5 Response normalization and *de novo* model-blinded expert evaluation

Each model output was copied from the web interface using the built-in copy function and stored in a separate Word document for each model. Before expert evaluation, emojis, Markdown formatting markers, and URL strings were removed. This process did not alter the substantive clinical content of the responses. All dietary recommendations, numerical values, conditional statements, qualifying language, and safety warnings were retained. The verbatim normalized responses are provided in Supplementary Data S1 (Verbatim Model Responses).

All model-generated responses were independently evaluated by three expert reviewers from hepatobiliary surgery, clinical nutrition, and internal medicine, respectively. All three reviewers held senior professional titles and had more than 10 years of clinical experience. The clinical nutrition reviewer also held registered dietitian credentials. The reviewers had participated in the multidisciplinary development of the cases and evaluation framework. They used the locked codebook provided in Supplementary File A, which defined the scenario-specific guideline adherence items, major dietary error categories, Harm Flag criteria, secondary outcomes, aggregation rules, and reviewer instructions. The scoring manual and codebook were finalized and locked before the *de novo* assessment used for the final analysis began and were not modified thereafter.

For the final analysis, all 144 responses underwent a new model-blinded assessment. Each reviewer received a separate anonymous response booklet. The order of the 36 cases and the order of the four responses within each case were independently randomized for each reviewer using computer-generated sequences. Model names were replaced with new anonymous response identifiers. The correspondence between these identifiers and model identities was retained by the study coordinator and was not disclosed to the reviewers during scoring.

Each reviewer completed the evaluation without access to the scores assigned by the other reviewers. They also had no access to previous ratings, model identities, or model-level results. Reviewers were instructed to score only information explicitly present in the model response and not to infer missing recommendations. For each response, reviewers confirmed the case identifier, clinical scenario, anonymous response identifier, and reviewer identifier, and then scored the seven scenario-specific guideline adherence items, six major dietary error items, Clinically Appropriate Food Examples, and Harm Flag. Under the locked reviewer instructions, response excerpts or brief clinical rationales were required for all negative guideline adherence ratings, positive major error ratings, positive Harm Flags, and negative food example ratings. The reviewers completed their assessments separately, and disagreements were retained without consensus adjustment.

The reviewer-level dataset contained 432 records, corresponding to 144 responses scored by three reviewers. For response-level analyses, reviewer scores were aggregated after completion of independent scoring. Continuous and ordinal scores were summarized using the mean reviewer score. Binary variables were summarized using majority rule across the three reviewers. Any Major Error was coded as 1 only when at least one specific error category reached majority agreement. Reviewer-level data were retained for inter-reviewer reliability analyses (23). The reviewer-level and response-level datasets are provided in Supplementary Data S2 (Reviewer-Level Dataset) and Supplementary Data S3 (Response-Level Dataset), respectively.

### 2.6 Outcomes and scoring criteria

The scoring framework comprised guideline adherence, major dietary error identification, Harm Flag, clinically appropriate food examples, and response length. The Guideline Adherence score was the primary outcome. Error Count, individual predefined major error categories, Any Major Error, and Harm Flag were secondary outcomes. Clinically Appropriate Food Examples was summarized descriptively, and Word Count was examined as an exploratory variable. The detailed operational definitions are summarized below and fully specified in the locked codebook.

Guideline adherence was assessed using scenario-specific seven-item checklists. Each item was scored as 1 if the recommendation was present and consistent with guideline principles and as 0 if it was absent, vague, or inconsistent. The Guideline Adherence score was calculated as the sum of G1 to G7 and ranged from 0 to 7. Because G1 to G7 were scenario-specific, individual item names were not considered interchangeable across scenarios.

For protein-related adherence items, a score of 1 required a numerical target consistent with approximately 1.2 to 1.5 g/kg/day or an equivalent daily amount calculable from the body weight provided in the case. General statements such as “adequate protein” or “moderate protein” were scored as 0 when no numerical target was given. For energy-related items, an energy target of approximately 30 to 35 kcal/kg/day was considered guideline-consistent for patients without obesity. For patients with obesity, a moderate energy deficit of approximately 500 to 800 kcal/day below estimated requirements, or another clinically reasoned target based on body weight, was accepted only when adequate protein intake and muscle preservation were protected. A response could also receive credit when it clearly advised sufficient energy intake to prevent nutritional deterioration or catabolism. General advice to eat a healthy or balanced diet was not sufficient. When a target was expressed per kilogram, calculations based on actual body weight, ideal body weight, or estimated dry weight were accepted when clinically justified. For patients with ascites, estimated dry weight or ideal body weight was considered appropriate.

Conditional recommendations were evaluated according to both the stated condition and the advised action. A conditional statement could satisfy an adherence item when it clearly provided appropriate guidance for the clinical scenario. It could also meet the definition of a major dietary error when the proposed action would be clinically inappropriate if the stated condition occurred. When a response contained both guideline-consistent and conflicting advice for the same item, that item was scored as 0 unless the incorrect statement was explicitly rejected or corrected. A specific numerical or actionable recommendation took precedence over a general reassuring statement.

Avoidance of prolonged fasting, small frequent meals, and a late-evening snack were treated as separate dietary practices. A general recommendation to eat regularly did not automatically satisfy all three items. Avoidance of prolonged fasting required advice against long intervals without food. Small frequent meals required an explicit recommendation to distribute intake across several eating occasions. A late-evening snack required a specific bedtime or late-evening eating recommendation.

For compensated cirrhosis, the seven guideline adherence items were adequate caloric intake, adequate protein intake, avoidance of prolonged fasting, small frequent meals, late-evening snack, alcohol avoidance, and nutritional monitoring or follow-up.

For cirrhosis with ascites, the seven items were sodium restriction, recognition of hidden sodium sources, adequate caloric intake, adequate protein intake, small frequent meals, late-evening snack, and ascites-related monitoring. Ascites-related monitoring included body weight, edema, appetite, ascites status, nutritional status, or clinical follow-up.

For hepatic encephalopathy recovery, the seven items were explicit avoidance of routine protein restriction, adequate protein intake, small frequent meals, avoidance of prolonged fasting, late-evening snack, recognition of malnutrition or sarcopenia risk, and appropriate protein source suggestions.

Major dietary errors were assessed independently from guideline adherence. Six major dietary error categories defined in the locked codebook were evaluated: unnecessary long-term protein restriction, high-sodium foods recommended in ascites, no protein guidance provided, extreme dietary restriction, advice ignoring the clinical context, and unsupported excessive fluid restriction. Error Count was calculated as the sum of E1 to E6 and ranged from 0 to 6. Any Major Error was coded as present only when at least one specific error category was supported by the majority of reviewers. The applicability of each error category across the three clinical scenarios was specified in the locked error-elicitation matrix before the *de novo* assessment used for the final analysis began.

Harm Flag was defined as an independent binary expert-rated outcome for potential safety concerns. It was coded as 1 if the response contained any recommendation that could plausibly increase the risk of nutritional deterioration, worsening ascites, worsening hepatic encephalopathy, dehydration, delayed clinical assessment, or other clinically relevant harm if followed without professional correction. Only recommendations explicitly stated in the response could trigger a Harm Flag. Reviewers were permitted to judge the plausible clinical consequence of following that stated recommendation. Harm Flag was not automatically equivalent to Error Count greater than zero, and minor omissions were not automatically considered harmful. Harm Flag was analyzed separately from Guideline Adherence and was not subtracted from the primary score. The locked codebook also specifies the harm categories and required supporting evidence used in the *de novo* assessment.

Clinically Appropriate Food Examples was coded as 1 when the response provided specific food or meal examples appropriate for the clinical scenario. It was coded as 0 when no specific examples were provided or when the examples were clinically inappropriate. This variable was treated as a descriptive outcome and was not incorporated into the Guideline Adherence score.

Word Count was calculated centrally as the number of alphanumeric lexical tokens in the normalized English response text. Decimal expressions and hyphenated or slash-separated lexical forms were counted as single tokens. Formatting markers, emojis, and URL strings were removed before counting. The same procedure was applied to all models. Word Count was used only for exploratory analysis and was not based on reviewer estimates or the count displayed by the model interface.

Two definitions of full adherence were retained. Unanimous full adherence required all three reviewers to assign a Guideline Adherence score of 7. Majority-item full adherence required each of the seven guideline items to receive a score of 1 from at least two reviewers. Because response-level Guideline Adherence was calculated as the reviewer mean, a response could meet the majority-item full adherence definition while having a mean score below 7 when one reviewer disagreed with the other two.

### 2.7 Statistical analysis

All statistical analyses were performed using R 4.4.2 and Python 3.13.5. The primary outcome was the response-level Guideline Adherence score, which represented scenario-specific guideline adherence and ranged from 0 to 7. Secondary outcomes included Error Count, individual major dietary error items, Any Major Error, and Harm Flag. Clinically Appropriate Food Examples was summarized descriptively, while Word Count was examined as an exploratory variable. The final dataset comprised 432 reviewer-level records and 144 aggregated response-level records. Reviewer-level data were used to assess inter-reviewer reliability, whereas model comparisons were performed using response-level aggregated data.

For response-level aggregation, Guideline Adherence score and Error Count were calculated as the mean of the three reviewer scores. Binary guideline items, major dietary error items, Harm Flag, and Clinically Appropriate Food Examples were determined by majority agreement across the three reviewers. Any Major Error was coded as 1 only when at least one specific error category received a positive rating from at least two reviewers. Majority-item full adherence was treated as a binary secondary summary outcome, whereas unanimous full adherence was summarized descriptively. Descriptive statistics were used to summarize model performance. Continuous or ordinal variables were reported as mean with standard deviation and median with interquartile range, as appropriate. Categorical variables were summarized as counts and percentages. Wilson 95% confidence intervals were calculated for proportions. Percentile bootstrap 95% confidence intervals were estimated from 3,000 resamples. For paired model comparisons, resampling was performed at the case level, retaining all four model responses for each sampled case; scenario-specific paired analyses resampled within the 12 cases of the relevant scenario.

Because each simulated case was submitted to all four models, model outputs were treated as paired observations within case. Comparisons of Guideline Adherence among the four models were performed using the Friedman test. Kendall’s W was reported as the corresponding effect size. When the overall test indicated a significant difference, pairwise Wilcoxon signed-rank tests with Holm adjustment were used for *post hoc* comparisons. Zero differences were handled using the Pratt method. Holm adjustment was applied across the six pairwise model comparisons for each outcome and analysis stratum. Matched-pairs rank-biserial correlation and its 95% confidence interval were reported for pairwise effect size estimation. The same paired nonparametric approach was applied to Error Count and Word Count (24).

Binary outcomes with sufficient response-level variation, including Any Major Error, Harm Flag, and majority-item full adherence, were compared using Cochran’s Q test. When the overall test indicated a statistically significant difference, pairwise exact McNemar tests with Holm adjustment were performed. Paired risk differences were calculated as the mean within-case difference in the binary outcome between models, with 95% confidence intervals estimated using the same case-level percentile bootstrap. Individual guideline items, individual major dietary error categories, and Clinically Appropriate Food Examples were summarized descriptively because several variables showed no variation or only sparse positive events. Because G1 to G7 represented scenario-specific checklist items, item-level analyses were conducted within the corresponding clinical scenario rather than pooled across scenarios.

Inter-reviewer reliability for Guideline Adherence was assessed using the intraclass correlation coefficient based on a two-way random-effects model with absolute agreement. Agreement for binary safety-related variables, including major dietary error items and Harm Flag, was summarized using complete agreement rates and Fleiss’ kappa when applicable (25, 26). The relationship between Word Count and Guideline Adherence was examined using Spearman rank correlation as an exploratory analysis. Correlations were calculated for the complete dataset and separately within each model. A model-specific correlation was considered not estimable when the Guideline Adherence score showed no variation. All statistical tests were two-sided, and a *p* value of less than 0.05 was considered statistically significant after adjustment where applicable. The reviewer-level and response-level datasets used for the analyses are provided as Supplementary Data S2, S3.

## 3 Results

### 3.1 Dataset overview and inter-reviewer reliability

A total of 36 standardized simulated cases were included in this study, covering three cirrhosis-related nutritional counseling scenarios: compensated cirrhosis, cirrhosis with ascites, and hepatic encephalopathy recovery. Each scenario contained 12 cases. The same set of cases was submitted to four large language models, including DeepSeek, ChatGPT, Qwen, and Claude, resulting in 144 model-generated dietary recommendations. Each response was independently evaluated by three model-blinded reviewers, yielding 432 reviewer-level scoring records. Complete reviewer-level assessments were available for all 144 responses. After completion of independent scoring, response-level aggregated data were generated for model comparison, with continuous and ordinal variables summarized using reviewer mean values and binary variables summarized using majority rule. The characteristics of the simulated cases are summarized in Table **1**.

**Table 1:** Study dataset, clinical scenarios, and evaluation structure.

Characteristic	Overall (*n* = 36)	Compensated cirrhosis (*n* = 12)	Cirrhosis with ascites (*n* = 12)	Hepatic encephalopathy recovery (*n* = 12)
Case characteristics				
Cases, *n*	36	12	12	12
Age, years	54.0 ± 12.8	53.3 ± 12.6	53.8 ± 13.5	54.8 ± 13.4
Female sex, *n* (%)	18 (50.0)	6 (50.0)	6 (50.0)	6 (50.0)
Height, cm	164.1 ± 6.7	164.4 ± 6.9	164.2 ± 7.1	163.7 ± 6.8
Weight, kg	68.1 ± 12.0	68.2 ± 12.2	69.8 ± 12.6	66.2 ± 11.8
Body mass index, kg/m^2^	25.2 ± 3.8	25.2 ± 3.9	25.8 ± 4.1	24.7 ± 3.7
Etiology, *n* (%)				
HBV	11 (30.6)	4 (33.3)	4 (33.3)	3 (25.0)
MASLD	12 (33.3)	4 (33.3)	4 (33.3)	4 (33.3)
Alcohol-related	12 (33.3)	4 (33.3)	4 (33.3)	4 (33.3)
Unspecified	1 (2.8)	0 (0.0)	0 (0.0)	1 (8.3)
Scenario definition	Three prespecified outpatient or postdischarge nutritional counseling scenarios	Stable outpatient cirrhosis without ascites or hepatic encephalopathy	Stable outpatient or postdischarge cirrhosis with ascites and oral intake possible	Previous hepatic encephalopathy; recovered and clinically stable at dietary counseling

Evaluation structure	
Large language models evaluated, *n*	4
Responses generated per case, *n*	4
Total model-generated responses, *n*	144
Reviewers per response, *n*	3
Reviewer-level scoring records, *n*	432
Aggregated response-level records, *n*	144

Values are presented as mean ± standard deviation or *n* (%), unless otherwise indicated. BMI, body mass index; MASLD, metabolic dysfunction-associated steatotic liver disease.

Body mass index was calculated from the height and weight stated in each simulated case. BMI, body mass index; HBV, hepatitis B virus; HE, hepatic encephalopathy; MASLD, metabolic dysfunction-associated steatotic liver disease; MELD, Model for End-Stage Liver Disease.

Inter-reviewer reliability for the Guideline Adherence score was high. The single-reviewer ICC(2,1) was 0.944 (95% CI, 0.864–0.985), and the average-measure ICC(2,k) was 0.981 (95% CI, 0.950–0.995). For binary safety-related variables with sufficient positive variation for meaningful interpretation, complete agreement was 93.8% for Harm Flag, 97.9% for E1, and 98.6% for E6; the corresponding Fleiss’ κ values were 0.752 (95% CI, 0.548–0.894), 0.917 (95% CI, 0.798–1.000), and 0.841 (95% CI, 0.495–1.000), respectively. Complete agreement rates and results for all binary variables, including sparse or invariant categories, are provided in Supplementary Table S3 (Inter-Reviewer Reliability).

### 3.2 Overall model performance

Overall guideline adherence was high across the four large language models, with a median Guideline Adherence score of 7.00 for all models. ChatGPT achieved a mean Guideline Adherence score of 7.00 ± 0.00, followed by Claude at 6.97 ± 0.17, DeepSeek at 6.94 ± 0.19, and Qwen at 6.44 ± 0.84. Unanimous full adherence, defined as all three reviewers assigning a score of 7, was observed in 36 of 36 ChatGPT responses, 35 of 36 Claude responses, 31 of 36 DeepSeek responses, and 23 of 36 Qwen responses. Under the majority-item definition, full adherence was observed in 36, 35, 35, and 24 responses generated by ChatGPT, Claude, DeepSeek, and Qwen, respectively.

The overall difference in Guideline Adherence among models was statistically significant (Friedman χ^2^[3] = 22.626, *p* = 0.0000483; Kendall’s W = 0.210). In Holm-adjusted pairwise analyses, ChatGPT had higher Guideline Adherence scores than Qwen (adjusted *p* = 0.00203), as did Claude (adjusted *p* = 0.00282). The difference between DeepSeek and Qwen did not reach statistical significance after adjustment (adjusted *p* = 0.10164), and no statistically significant differences were detected among DeepSeek, ChatGPT, and Claude. Majority-item full adherence also differed among models (Cochran’s Q = 30.632, *p* = 0.00000102). Qwen had a lower frequency of majority-item full adherence than DeepSeek (adjusted *p* = 0.00488), ChatGPT (adjusted *p* = 0.00293), and Claude (adjusted *p* = 0.00488). The response-level score distributions are shown in Figure 2.

**Figure 2:**
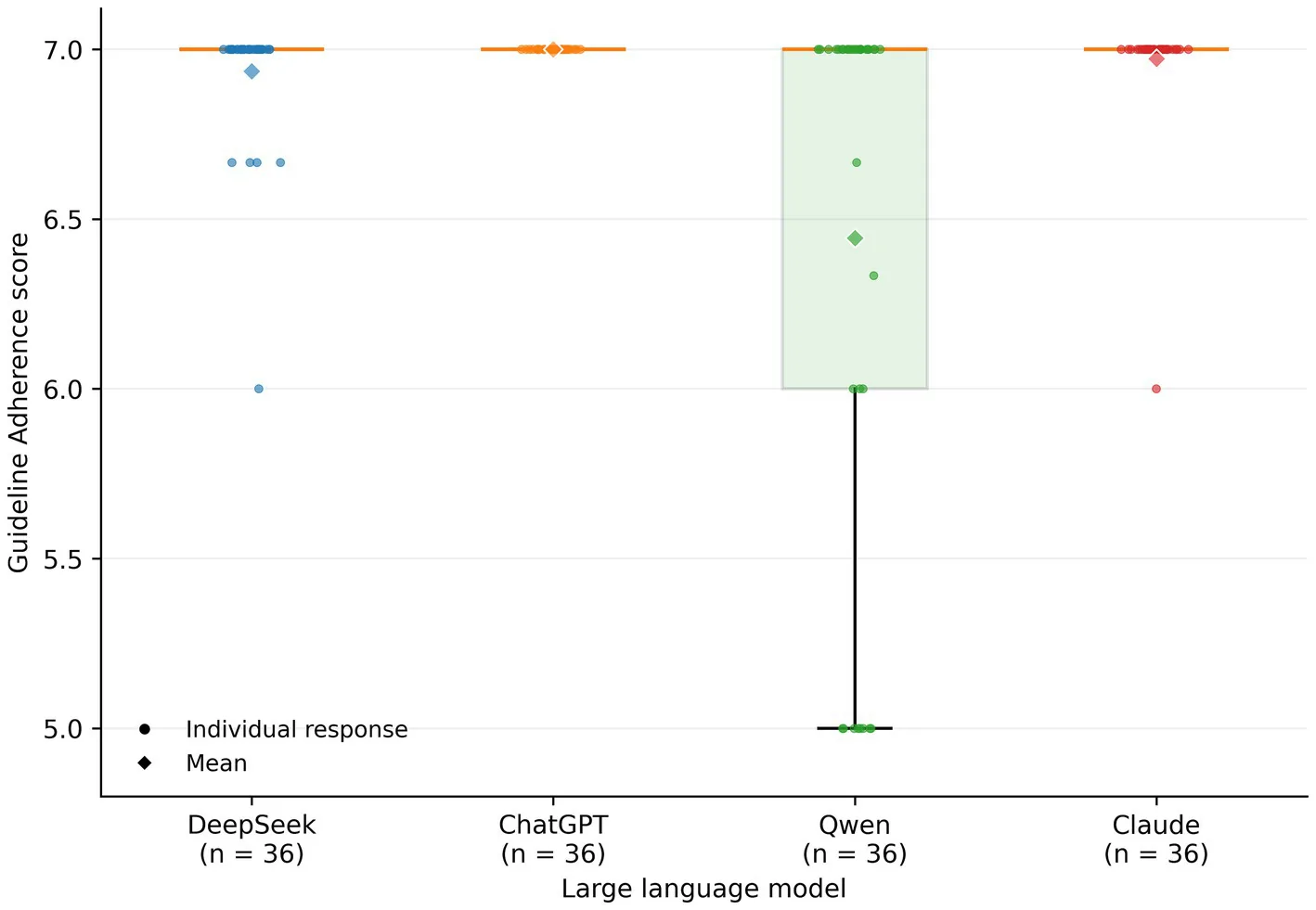
Distribution of response-level guideline adherence scores across four large language models. Each point represents one model-case response based on the mean score of three reviewers (*n* = 36 per model). Diamonds indicate model means scores range from 0 to 7, with higher scores indicating greater adherence.

The mean Error Count was 0.35 ± 0.56 for Qwen, 0.12 ± 0.29 for DeepSeek, 0.06 ± 0.24 for Claude, and 0.00 ± 0.00 for ChatGPT. The overall model difference was statistically significant (Friedman χ^2^[3] = 18.371, *p* = 0.000369; Kendall’s W = 0.170). Qwen had a higher Error Count than ChatGPT (adjusted *p* = 0.00338) and Claude (adjusted *p* = 0.02643), whereas its difference from DeepSeek was not statistically significant after Holm adjustment (adjusted *p* = 0.18885).

Any Major Error and Harm Flag were most frequent in Qwen responses, occurred less often in DeepSeek and Claude responses, and were absent from ChatGPT responses. Detailed analyses of potential safety concerns are reported in Section 3.5. Overall model performance is summarized in Table 2, and detailed *post hoc* results, effect sizes, paired risk differences, and confidence intervals are provided in Supplementary Table S1 (Pairwise Comparisons).

**Table 2:** Overall performance of the four large language models.

Model	Guideline adherence score, mean ± SD (95% CI)	Median [IQR]	Unanimous full adherence, *n* (%) [95% CI]	Majority-item full adherence, *n* (%) [95% CI]	Error Count, mean ± SD (95% CI)	Any major error, *n* (%) [95% CI]	Harm Flag, *n* (%) [95% CI]	Clinically appropriate food examples, *n* (%)	Responses, *n*
DeepSeek	6.94 ± 0.19 (6.87 to 6.99)	7.00 [7.00 to 7.00]	31 (86.1) [71.3 to 93.9]	35 (97.2) [85.8 to 99.5]	0.12 ± 0.29 (0.04 to 0.22)	4 (11.1) [4.4 to 25.3]	2 (5.6) [1.5 to 18.1]	36 (100.0)	36
ChatGPT	7.00 ± 0.00 (7.00 to 7.00)	7.00 [7.00 to 7.00]	36 (100.0) [90.4 to 100.0]	36 (100.0) [90.4 to 100.0]	0.00 ± 0.00 (0.00 to 0.00)	0 (0.0) [0.0 to 9.6]	0 (0.0) [0.0 to 9.6]	36 (100.0)	36
Qwen	6.44 ± 0.84 (6.15 to 6.70)	7.00 [6.00 to 7.00]	23 (63.9) [47.6 to 77.5]	24 (66.7) [50.3 to 79.8]	0.35 ± 0.56 (0.18 to 0.55)	11 (30.6) [18.0 to 46.9]	8 (22.2) [11.7 to 38.1]	36 (100.0)	36
Claude	6.97 ± 0.17 (6.92 to 7.00)	7.00 [7.00 to 7.00]	35 (97.2) [85.8 to 99.5]	35 (97.2) [85.8 to 99.5]	0.06 ± 0.24 (0.00 to 0.15)	2 (5.6) [1.5 to 18.1]	1 (2.8) [0.5 to 14.2]	36 (100.0)	36

Results are based on 36 responses per model. Guideline Adherence and Error Count are response-level means across three reviewers; binary outcomes were determined by majority rule. CI, confidence interval; IQR, interquartile range.

Overall Guideline Adherence: Friedman χ^2^(3) = 22.626, *p* = 0.0000483, Kendall’s W = 0.210. Majority-item full adherence: Cochran’s Q = 30.632, *p* = 0.00000102. Error Count: Friedman χ^2^(3) = 18.371, *p* = 0.000369, Kendall’s W = 0.170. Any Major Error: Cochran’s Q = 19.186, *p* = 0.000250. Harm Flag: Cochran’s Q = 14.091, *p* = 0.002784. CI, confidence interval; IQR, interquartile range; SD, standard deviation.

### 3.3 Scenario-specific model performance

Guideline Adherence scores were concentrated at or near the maximum in the compensated cirrhosis and ascites scenarios, while a marked between-model difference emerged in hepatic encephalopathy recovery. In compensated cirrhosis, the mean Guideline Adherence score was 6.94 ± 0.13 for DeepSeek and 7.00 ± 0.00 for ChatGPT, Qwen, and Claude. The overall model difference was not statistically significant (Friedman χ^2^[3] = 6.000, *p* = 0.111610; Kendall’s W = 0.167). Majority-item full adherence was observed in all 12 responses from each model. Unanimous full adherence was observed in 10 of 12 DeepSeek responses and all 12 responses from each of the other three models. No response in this scenario met the majority-rule criteria for Any Major Error or Harm Flag.

In cirrhosis with ascites, the mean Guideline Adherence scores were 6.94 ± 0.13 for DeepSeek, 7.00 ± 0.00 for ChatGPT, 6.97 ± 0.10 for Qwen, and 7.00 ± 0.00 for Claude. No statistically significant overall model difference was detected (Friedman χ^2^[3] = 3.667, *p* = 0.299781; Kendall’s W = 0.102). All four models achieved majority-item full adherence in all 12 cases. Unanimous full adherence was observed in 10 DeepSeek responses, 11 Qwen responses, and all 12 ChatGPT and Claude responses. One DeepSeek response was classified as containing Any Major Error and also received a Harm Flag. No majority-rule major error or Harm Flag was identified for ChatGPT, Qwen, or Claude in this scenario.

The largest divergence was observed in hepatic encephalopathy recovery. ChatGPT achieved a mean Guideline Adherence score of 7.00 ± 0.00, while the corresponding means were 6.92 ± 0.29 for DeepSeek, 6.92 ± 0.29 for Claude, and 5.36 ± 0.54 for Qwen. The overall model difference was statistically significant (Friedman χ^2^[3] = 28.558, *p* = 0.00000277; Kendall’s W = 0.793). In the Holm-adjusted pairwise comparisons, Qwen had lower Guideline Adherence scores than DeepSeek, ChatGPT, and Claude, with an adjusted *p* value of 0.00919 for each comparison. No statistically significant differences were detected among DeepSeek, ChatGPT, and Claude.

In this scenario, unanimous full adherence was observed in all 12 ChatGPT responses, 11 DeepSeek responses, 11 Claude responses, and none of the Qwen responses. The majority-item full-adherence results followed the same pattern. Qwen had a lower majority-item full-adherence frequency than DeepSeek, ChatGPT, and Claude after Holm adjustment, with adjusted *p* values of 0.00488, 0.00293, and 0.00488, respectively.

Error Count also differed among models in hepatic encephalopathy recovery (Friedman χ^2^[3] = 24.214, *p* = 0.00002253; Kendall’s W = 0.673). The mean Error Count was 1.06 ± 0.42 for Qwen, 0.22 ± 0.41 for DeepSeek, 0.17 ± 0.39 for Claude, and 0.00 ± 0.00 for ChatGPT. Qwen had a higher Error Count than DeepSeek, ChatGPT, and Claude, with Holm-adjusted *p* values of 0.02415, 0.00936, and 0.01377, respectively.

Any Major Error was identified in 11 of 12 Qwen responses, 3 of 12 DeepSeek responses, 2 of 12 Claude responses, and none of the ChatGPT responses in hepatic encephalopathy recovery. The overall difference was statistically significant (Cochran’s Q = 21.000, *p* = 0.000105). Qwen had a higher frequency than ChatGPT (adjusted *p* = 0.00586) and Claude (adjusted *p* = 0.01953), while the comparison with DeepSeek did not reach statistical significance after adjustment (adjusted *p* = 0.08594). Harm Flag was assigned to 8 Qwen responses, 1 DeepSeek response, 1 Claude response, and no ChatGPT response. The overall comparison was statistically significant (Cochran’s Q = 16.400, *p* = 0.000939), although only the Qwen versus ChatGPT comparison remained statistically significant after Holm adjustment (adjusted *p* = 0.04688). Scenario-specific results are summarized in Table 3 and Figure 3, and the complete pairwise results are provided in Supplementary Table S1 (Pairwise Comparisons).

**Table 3:** Scenario-specific performance of the four large language models.

Scenario	Model	Guideline Adherence score, mean ± SD (95% CI)	Median [IQR]	Unanimous full adherence, *n* (%) [95% CI]	Majority-item full adherence, *n* (%) [95% CI]	Responses, *n*
Panel A: Guideline adherence and full-adherence outcomes						
Compensated cirrhosis	DeepSeek	6.94 ± 0.13 (6.86 to 7.00)	7.00 [7.00 to 7.00]	10 (83.3) [55.2 to 95.3]	12 (100.0) [75.8 to 100.0]	12
	ChatGPT	7.00 ± 0.00 (7.00 to 7.00)	7.00 [7.00 to 7.00]	12 (100.0) [75.8 to 100.0]	12 (100.0) [75.8 to 100.0]	12
	Qwen	7.00 ± 0.00 (7.00 to 7.00)	7.00 [7.00 to 7.00]	12 (100.0) [75.8 to 100.0]	12 (100.0) [75.8 to 100.0]	12
	Claude	7.00 ± 0.00 (7.00 to 7.00)	7.00 [7.00 to 7.00]	12 (100.0) [75.8 to 100.0]	12 (100.0) [75.8 to 100.0]	12
Cirrhosis with ascites	DeepSeek	6.94 ± 0.13 (6.86 to 7.00)	7.00 [7.00 to 7.00]	10 (83.3) [55.2 to 95.3]	12 (100.0) [75.8 to 100.0]	12
	ChatGPT	7.00 ± 0.00 (7.00 to 7.00)	7.00 [7.00 to 7.00]	12 (100.0) [75.8 to 100.0]	12 (100.0) [75.8 to 100.0]	12
	Qwen	6.97 ± 0.10 (6.92 to 7.00)	7.00 [7.00 to 7.00]	11 (91.7) [64.6 to 98.5]	12 (100.0) [75.8 to 100.0]	12
	Claude	7.00 ± 0.00 (7.00 to 7.00)	7.00 [7.00 to 7.00]	12 (100.0) [75.8 to 100.0]	12 (100.0) [75.8 to 100.0]	12
Hepatic encephalopathy recovery	DeepSeek	6.92 ± 0.29 (6.75 to 7.00)	7.00 [7.00 to 7.00]	11 (91.7) [64.6 to 98.5]	11 (91.7) [64.6 to 98.5]	12
	ChatGPT	7.00 ± 0.00 (7.00 to 7.00)	7.00 [7.00 to 7.00]	12 (100.0) [75.8 to 100.0]	12 (100.0) [75.8 to 100.0]	12
	Qwen	5.36 ± 0.54 (5.08 to 5.67)	5.00 [5.00 to 6.00]	0 (0.0) [0.0 to 24.2]	0 (0.0) [0.0 to 24.2]	12
	Claude	6.92 ± 0.29 (6.75 to 7.00)	7.00 [7.00 to 7.00]	11 (91.7) [64.6 to 98.5]	11 (91.7) [64.6 to 98.5]	12

Scenario	Model	Error count, mean ± SD (95% CI)	Any major error, *n* (%) [95% CI]	Harm flag, *n* (%) [95% CI]	Responses, *n*	Interpretive note
Panel B: Error outcomes and expert-rated potential safety concerns						
Compensated cirrhosis	DeepSeek	0.08 ± 0.21 (0.00 to 0.19)	0 (0.0) [0.0 to 24.2]	0 (0.0) [0.0 to 24.2]	12	No majority-rule major error or Harm Flag.
	ChatGPT	0.00 ± 0.00 (0.00 to 0.00)	0 (0.0) [0.0 to 24.2]	0 (0.0) [0.0 to 24.2]	12	No majority-rule major error or Harm Flag.
	Qwen	0.00 ± 0.00 (0.00 to 0.00)	0 (0.0) [0.0 to 24.2]	0 (0.0) [0.0 to 24.2]	12	No majority-rule major error or Harm Flag.
	Claude	0.03 ± 0.10 (0.00 to 0.08)	0 (0.0) [0.0 to 24.2]	0 (0.0) [0.0 to 24.2]	12	No majority-rule major error or Harm Flag.
Cirrhosis with ascites	DeepSeek	0.06 ± 0.19 (0.00 to 0.17)	1 (8.3) [1.5 to 35.4]	1 (8.3) [1.5 to 35.4]	12	One E6 event also received a Harm Flag.
	ChatGPT	0.00 ± 0.00 (0.00 to 0.00)	0 (0.0) [0.0 to 24.2]	0 (0.0) [0.0 to 24.2]	12	
	Qwen	0.00 ± 0.00 (0.00 to 0.00)	0 (0.0) [0.0 to 24.2]	0 (0.0) [0.0 to 24.2]	12	
	Claude	0.00 ± 0.00 (0.00 to 0.00)	0 (0.0) [0.0 to 24.2]	0 (0.0) [0.0 to 24.2]	12	
Hepatic encephalopathy recovery	DeepSeek	0.22 ± 0.41 (0.00 to 0.47)	3 (25.0) [8.9 to 53.2]	1 (8.3) [1.5 to 35.4]	12	A small number of majority-rule potential safety-concern event were identified.
	ChatGPT	0.00 ± 0.00 (0.00 to 0.00)	0 (0.0) [0.0 to 24.2]	0 (0.0) [0.0 to 24.2]	12	
	Qwen	1.06 ± 0.42 (0.83 to 1.31)	11 (91.7) [64.6 to 98.5]	8 (66.7) [39.1 to 86.2]	12	Safety events were concentrated in HE recovery responses.
	Claude	0.17 ± 0.39 (0.00 to 0.42)	2 (16.7) [4.7 to 44.8]	1 (8.3) [1.5 to 35.4]	12	A small number of majority-rule potential safety-concern events were identified.

Each scenario included 12 responses per model. Panel A summarizes guideline adherence, and Panel B summarizes error outcomes and expert-rated potential safety concerns. Continuous outcomes are response-level means across three reviewers; binary outcomes were determined by majority rule. CI, confidence interval; HE, hepatic encephalopathy; IQR, interquartile range.

Mean confidence intervals are percentile bootstrap intervals based on 3,000 response-level resamples. Proportion confidence intervals are Wilson 95% confidence intervals. Scenario-specific Guideline Adherence tests: compensated cirrhosis, Friedman χ^2^(3) = 6.000, *p* = 0.111610, Kendall’s W = 0.167; cirrhosis with ascites, χ^2^(3) = 3.667, *p* = 0.299781, W = 0.102; hepatic encephalopathy recovery, χ^2^(3) = 28.558, *p* = 0.00000277, W = 0.793. Within hepatic encephalopathy recovery, Error Count differed among models (Friedman χ^2^[3] = 24.214, *p* = 0.00002253; Kendall’s W = 0.673). Any Major Error and Harm Flag also differed (Cochran’s Q = 21.000, *p* = 0.000105; Q = 16.400, *p* = 0.000939, respectively).

**Figure 3:**
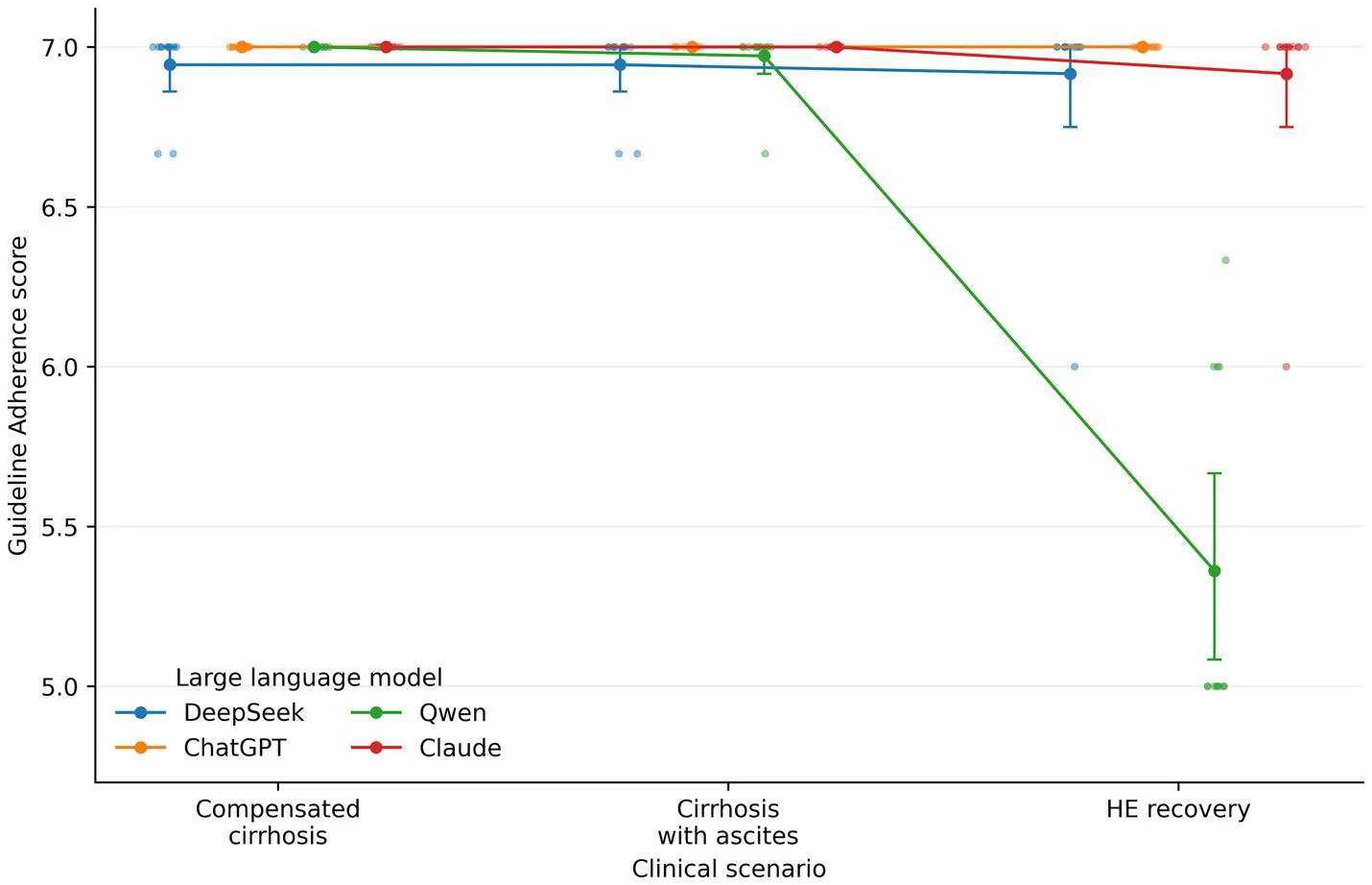
Scenario-specific guideline adherence scores across four large language models. Each scenario included 12 responses per model. Small points represent individual response-level scores, large points represent model means, and error bars indicate bootstrap 95% confidence intervals. Scores range from 0 to 7. HE, hepatic encephalopathy.

### 3.4 Scenario-specific guideline item adherence

Item-level adherence was examined separately within each clinical scenario because the G1 to G7 items represented different nutritional domains across the three scenario-specific checklists. Each binary item was aggregated across the three reviewers using the majority rule specified in Supplementary File A.

In compensated cirrhosis, all four models achieved majority-rule adherence in 12 of 12 responses for each of the seven items: adequate caloric intake, adequate protein intake, avoidance of prolonged fasting, small frequent meals, late-evening snack, alcohol avoidance, and nutritional monitoring or follow-up.

The same ceiling pattern was observed in cirrhosis with ascites. DeepSeek, ChatGPT, Qwen, and Claude each achieved adherence in 12 of 12 responses for sodium restriction, recognition of hidden sodium sources, adequate caloric intake, adequate protein intake, small frequent meals, late-evening snack, and ascites-related monitoring. Thus, all 28 model-item combinations achieved full majority-rule adherence.

Item-level variation was confined to hepatic encephalopathy recovery. ChatGPT achieved adherence in 12 of 12 responses for all seven items. DeepSeek and Claude each achieved adherence in 11 of 12 responses for G1, explicit avoidance of routine protein restriction, corresponding to 91.7% (95% CI, 64.6%–98.5%); both models achieved 100.0% adherence for G2 through G7.

Qwen achieved adherence in 0 of 12 responses for G1, explicit avoidance of routine protein restriction, corresponding to 0% (95% CI, 0%–24.2%). For G2, adequate protein intake, adherence was observed in 4 of 12 responses, corresponding to 33.3% (95% CI, 13.8%–60.9%). Qwen achieved adherence in all 12 responses for G3 through G7, including small frequent meals, avoidance of prolonged fasting, late-evening snack, recognition of malnutrition or sarcopenia risk, and appropriate protein source suggestions. Therefore, the reduced item-level performance in hepatic encephalopathy recovery was concentrated in the two core protein-management items rather than distributed across the broader nutritional checklist. Scenario-specific item adherence is shown in Figure 4.

**Figure 4:**
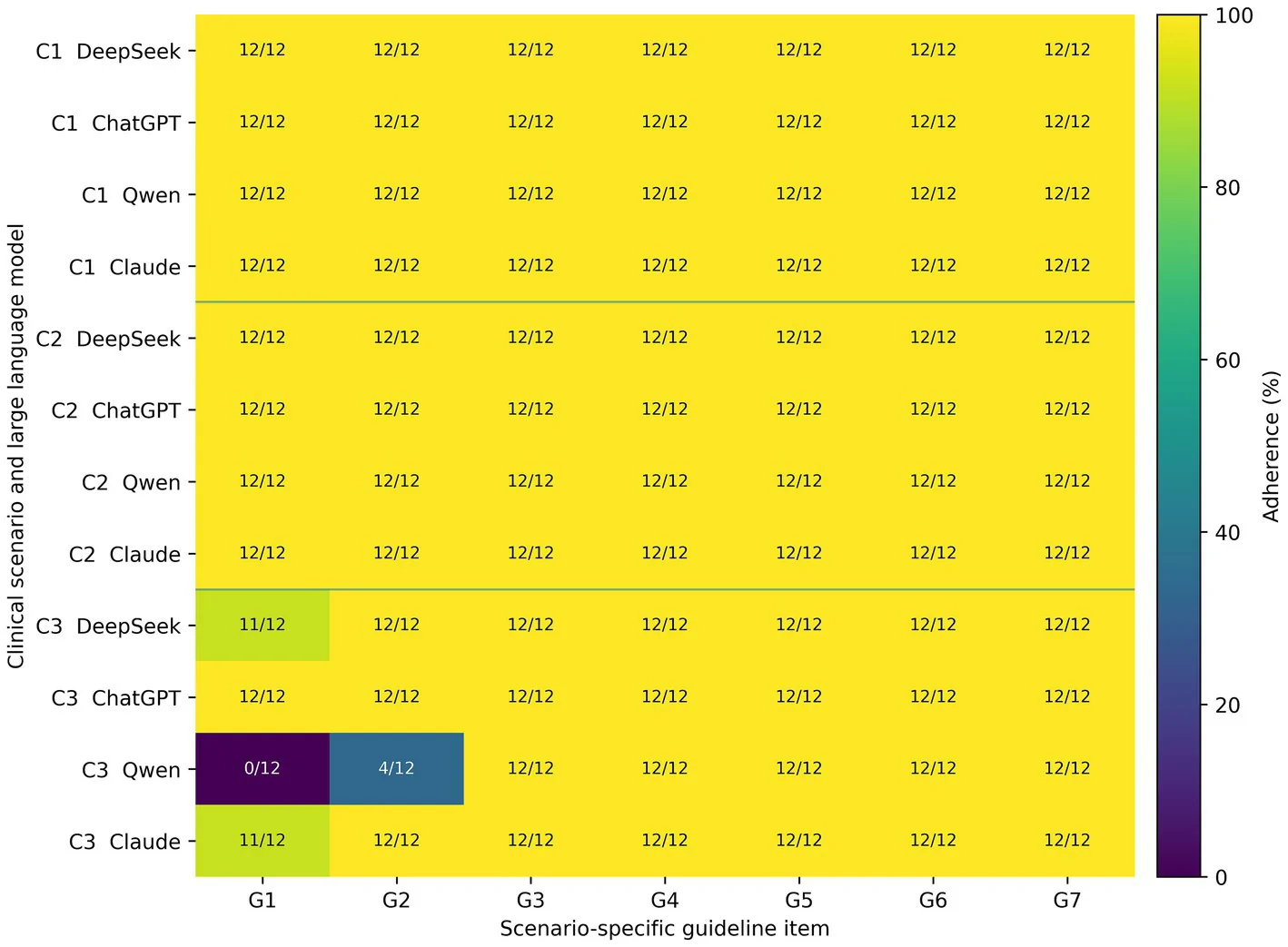
Scenario-specific guideline item adherence across four large language models. Each cell shows the number of responses meeting the item under majority rule out of 12, with color indicating the percentage. For compensated cirrhosis G1-G7 represent energy, protein, fasting avoidance, small frequent meals, late-evening snack, alcohol avoidance, and nutritional monitoring. For ascites, they represent sodium restriction, hidden sodium, energy, protein, small frequent meals, late-evening snack, and ascites monitoring. For hepatic encephalopathy recovery, they represent avoidance of routine protein restriction, adequate protein, small frequent meals, fasting avoidance, late-evening snack, malnutrition or sarcopenia risk, and appropriate protein sources.

### 3.5 Major dietary errors and expert-rated potential safety concerns

Across the 144 response-level records, 17 responses met the majority-rule definition of Any Major Error. These included 11 of 36 Qwen responses, 4 of 36 DeepSeek responses, and 2 of 36 Claude responses. No ChatGPT response contained a majority-rule major dietary error. The errors were concentrated in hepatic encephalopathy recovery, which accounted for 16 of the 17 affected responses. The remaining event occurred in one DeepSeek response in the cirrhosis with ascites scenario. No majority-rule major dietary error was identified in compensated cirrhosis.

Among the six predefined error categories, E1, inappropriate protein restriction or protein reduction, was the most frequent and was identified in 13 responses. E1 occurred in 11 Qwen responses, one DeepSeek response, and one Claude response, all within the hepatic encephalopathy recovery scenario. The E1 recommendations included routine protein targets below 1.2 g/kg/day and instructions to reduce protein temporarily if hepatic encephalopathy symptoms recurred. E5, advice that did not adequately account for the clinical context, was identified in one Qwen response. E6, unsupported excessive fluid restriction, was identified in five responses, including three generated by DeepSeek, one by Qwen, and one by Claude. No response met the majority-rule criteria for E2, E3, or E4.

The error categories were not mutually exclusive. One Qwen response met both E1 and E5, and another Qwen response met both E1 and E6. Therefore, the sum of the individual error-category counts exceeded the number of responses classified as containing Any Major Error.

Harm Flag was assigned to 11 responses overall, including 8 Qwen responses, 2 DeepSeek responses, and 1 Claude response. No ChatGPT response received a Harm Flag. Ten Harm Flags were classified as protein-related and were associated with inappropriate protein reduction in hepatic encephalopathy recovery. The remaining Harm Flag was fluid-related and occurred in a DeepSeek ascites response that advised fluid restriction without a stated indication such as clinically relevant hyponatremia. Six responses that met the definition of Any Major Error did not receive a Harm Flag. Thus, Any Major Error and Harm Flag did not identify identical sets of responses.

The overall frequency of Any Major Error differed among models (Cochran’s Q = 19.186, *p* = 0.000250). In Holm-adjusted pairwise analyses, Qwen had a higher frequency than ChatGPT (adjusted *p* = 0.00586) and Claude (adjusted *p* = 0.01953), whereas the comparison between Qwen and DeepSeek was not statistically significant (adjusted *p* = 0.26172). Harm Flag frequency also differed among models (Cochran’s Q = 14.091, *p* = 0.002784), although only the Qwen versus ChatGPT comparison remained statistically significant after Holm adjustment (adjusted *p* = 0.04688). The distribution of major error categories and expert-rated Harm Flags is shown in Figure 5. Detailed pairwise comparisons are provided in Supplementary Table S1 (Pairwise Comparisons).

**Figure 5:**
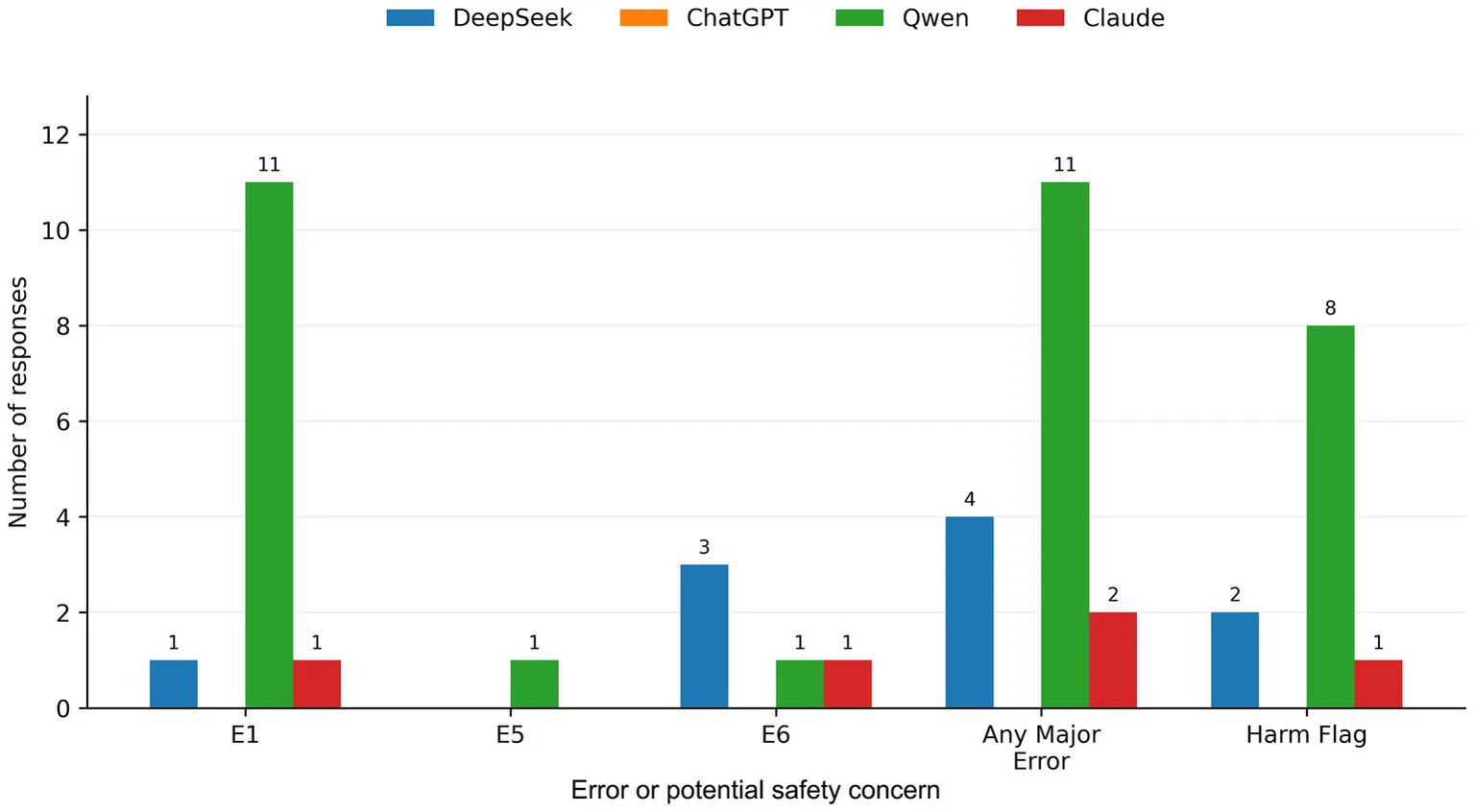
Major dietary errors and expert-rated Harm Flags across four large language models. Bars show response-level events under majority rule (*n* = 36 per model). E1, inappropriate protein restriction or reduction; E5, advice inconsistent with the clinical context; E6, unsupported excessive fluid restriction. E2-E4 had no majority-rule events and are not displayed. Error categories were not mutually exclusive.

### 3.6 Descriptive and exploratory analyses

Clinically Appropriate Food Examples were identified under the majority rule in all 144 responses, including 36 of 36 responses generated by each model. Because this outcome showed no variation at the aggregated response level, no between-model inferential comparison was performed.

Response length differed among the four models. ChatGPT generated the longest responses, with a mean Word Count of 753.9 ± 97.3, followed by Qwen at 692.3 ± 203.6, DeepSeek at 619.4 ± 117.0, and Claude at 602.0 ± 137.1. Qwen showed the greatest variability in response length. The overall model difference was statistically significant (Friedman χ^2^ [3] = 19.633, *p* = 0.000202; Kendall’s W = 0.182). In Holm-adjusted pairwise analyses, ChatGPT responses were longer than those generated by DeepSeek (adjusted *p* = 0.000814) and Claude (adjusted *p* = 0.000216). None of the other pairwise comparisons reached statistical significance after adjustment.

Across all 144 responses, Word Count was negatively correlated with Guideline Adherence (Spearman ρ = −0.221, *p* = 0.00785; 95% CI, −0.403 to −0.020). In the model-specific analyses, a negative correlation was also observed for Qwen (ρ = −0.597, *p* = 0.000121; 95% CI, −0.802 to −0.263). No statistically significant correlation was detected for DeepSeek (ρ = 0.046, *p* = 0.789; 95% CI, −0.228 to 0.330) or Claude (ρ = −0.089, *p* = 0.604; 95% CI, −0.247 to 0.008). The correlation could not be estimated for ChatGPT because all 36 responses had the same Guideline Adherence score of 7.00. Detailed response-length results and correlation estimates are provided in Supplementary Table S2 (Descriptive and Word Count Analyses) and Supplementary Figure S1 (Word Count and Guideline Adherence).

## 4 Discussion

This study compared guideline adherence and expert-rated potential safety concerns in dietary recommendations generated by four large language models across 3 cirrhosis scenarios. Overall adherence was high, but performance varied by model and scenario. ChatGPT achieved complete guideline adherence in this sample, Claude and DeepSeek performed near the upper limit, and Qwen showed lower and more variable performance, particularly in hepatic encephalopathy recovery. Major errors and Harm Flags were most frequent in Qwen responses, with fewer events in DeepSeek and Claude and none in ChatGPT. Because each platform generated one response per case during a defined testing period, these findings represent a time-specific comparison rather than a durable platform hierarchy.

The high overall scores should be interpreted in light of the structured prompt, which explicitly requested the domains assessed by the checklist. This design improved comparability but likely increased checklist completion relative to brief, vague, or emotionally framed patient questions.

Performance was near the ceiling in compensated cirrhosis and ascites, limiting discrimination among the higher-performing models. Non-significant differences in these scenarios should not be interpreted as equivalence. The largest divergence occurred in hepatic encephalopathy recovery, where Qwen had lower adherence and a higher Error Count. Protein management after hepatic encephalopathy was the main failure point. Current EASL, ESPEN, and AASLD guidance recommends protein intake of approximately 1.2 to 1.5 g/kg/day and advises against routine protein restriction because it can increase protein catabolism and worsen sarcopenia (2–4, 7, 8).

E1 occurred in 13 responses, including 11 from Qwen and one each from DeepSeek and Claude. In hepatic encephalopathy recovery, Qwen did not explicitly reject routine protein restriction in any of the 12 responses and provided an adequate numerical target in only four. Excessive restriction may worsen sarcopenia and reduce extrahepatic ammonia handling, making protein adequacy a safety-relevant domain (4, 8).

This pattern shows that a response can satisfy general nutritional items yet fail at one complication-sensitive decision. Such an isolated error may remain clinically important even when the response appears comprehensive.

Any Major Error and Harm Flag captured related but nonidentical constructs: 17 responses met the error definition, whereas 11 received a Harm Flag. Error categories identified textual defects defined in the locked codebook; Harm Flag additionally required an explicit recommendation judged capable of clinically meaningful harm. Neither outcome represented observed patient behavior or adverse events.

ChatGPT produced the longest responses while Qwen showed the greatest length variability. Word Count was negatively associated with Guideline Adherence overall, largely because of the stronger relationship within Qwen. This does not imply that shorter responses are inherently better; length alone is not a reliable indicator of clinical quality.

Accordingly, readability, completeness, response length, and food examples alone should not substitute for evaluation of potential safety concerns (27). A single high-impact error may outweigh otherwise comprehensive advice. Nutrition-focused evaluations of large language models have reached similarly mixed conclusions. Previous studies found that artificial intelligence-generated meal plans may provide useful general dietary ideas and readable explanations while missing energy or micronutrient targets, lacking clinically meaningful personalization, including contraindicated foods, or deviating from disease-specific guidance (28–31). The present findings extend this evidence to cirrhosis by showing that apparently comprehensive advice can remain vulnerable at a complication-sensitive decision point, particularly protein management after hepatic encephalopathy.

Large language models may have value as auxiliary education tools for broad cirrhosis nutrition principles under structured prompting (18, 19). They should support, not replace, counseling from hepatology and nutrition professionals (32). Professional verification is particularly important for recommendations involving protein after hepatic encephalopathy, sodium or fluid management in ascites, prolonged fasting, extreme energy restriction, supplementation, or delayed clinical assessment.

A practical workflow is to detect such high-risk statements, verify them against the clinical context and guideline thresholds, and correct, withhold, or escalate advice that cannot be verified (23, 33, 34). Strengths include the paired case design, three clinically relevant scenarios, *de novo* model-blinded assessment by three senior reviewers, a finalized and locked scenario-specific codebook, explicit operational error definitions, and evidence requirements for discordant ratings. Reliability was high, and the combination of adherence and outcomes capturing potential safety concerns allowed a multidimensional evaluation.

Limitations include the use of simulated rather than real patient encounters, omission of formal Child-Pugh and MELD scores, and a structured prompt that may have increased checklist completion. All prompts were in English, which may have influenced Qwen and precludes interpreting the findings as a language-neutral ranking.

Only one response was generated per platform-case pair, so within-platform response variability and follow-up interactions were not assessed. Because the evaluated systems were accessed through consumer web interfaces, the exact backend model deployments used for individual interactions could not be independently verified from the retained study records. The findings should therefore be interpreted as a time-specific comparison of outputs from the ChatGPT, DeepSeek, Qwen, and Claude web platforms during the defined testing period rather than as a version-specific comparison of fixed backend models. The sample included only 12 cases per scenario, and several potential safety-concern events were sparse. The same group developed the cases and framework, without formal pilot testing or external validation. Ceiling effects limited discrimination in two scenarios, and absent error categories may emerge in more provocative cases. The study evaluated written recommendations rather than patient comprehension, behavior, or clinical outcomes. Future studies should retain contemporaneous platform metadata where available, evaluate repeated and multilingual generations using real or more complex cases, independently validate the framework, and test supervised use in clinical workflows with patient- and clinician-centered outcomes.

## 5 Conclusion

Under structured English-language prompting, the four evaluated large language models generally produced guideline-consistent dietary advice for simulated patients with cirrhosis. Performance was near the ceiling in compensated cirrhosis and ascites but diverged in hepatic encephalopathy recovery, where Qwen showed lower adherence and more protein-related major errors and expert-rated Harm Flags. These findings reflect a time-specific, single-generation comparison and do not establish clinical safety or a stable platform hierarchy. Large language models may support general nutrition education, but complication-sensitive recommendations, particularly those involving protein, sodium, fluid intake, or fasting, require clinician or dietitian verification before individualized use.

## Data Availability

The original contributions presented in the study are included in the article/Supplementary material, further inquiries can be directed to the corresponding authors.

## Supplementary material

SUPPLEMENTARY FIGURE S1 Model-specific relationship between word count and response-level guideline adherence. Vertical jitter was used for visualization only; analyses used the unjittered values.
