# Supplementary material for: Guideline adherence and expert-rated potential safety concerns in AI-generated dietary advice for patients with cirrhosis: a comparative evaluation of large language models
Source: Front Nutr. 2026 Oct 8;13:1893597. doi: 10.3389/fnut.2026.1893597 (PMC13689687; doi:10.3389/fnut.2026.1893597)
Supplement: Supplementary file 1 [file Supplementary_file_1.zip › Supplementary File B CHART Checklist..docx]

**CHART checklist**

**Guideline Adherence and Expert-Rated Potential Safety Concerns in AI-Generated Dietary Advice for Patients with Cirrhosis: A Comparative Evaluation of Large Language Models**

| **Section** | **Item** | **Paraphrased CHART requirement** | **Status** | **Notes** |
| --- | --- | --- | --- | --- |
| Title and abstract | **1a** | Identify the work as an evaluation of one or more generative AI chatbots used for health advice. | Covered | The title identifies an evaluation of AI-generated dietary advice and explicitly frames the safety endpoint as expert-rated potential safety concerns; the abstract names the evaluated platforms and outcomes. |
| Title and abstract | **1b** | Use a structured abstract when appropriate. | Covered | Structured Background, Methods, Results, and Conclusion sections are provided. |
| Introduction | **2a** | Explain the scientific background, rationale, and health care context, supported by relevant literature. | Covered | The clinical context, rationale, and evidence gap are supported by cirrhosis, nutrition, and LLM literature. |
| Introduction | **2b** | State the study aims and specify the target users, evaluated intervention, comparators, and outcomes. | Covered | The evaluated web platforms, clinical scenarios, primary and secondary outcomes, and intended patient-facing context are specified. |
| Methods: model identifiers | **3a** | Report the chatbot/model names and identifiers, together with release or update information when available. | Covered | ChatGPT, DeepSeek, Qwen, and Claude are reported at the web-platform level with providers, testing period, official websites, free-tier access, and user-accessible settings. Exact backend deployments are unknown or not independently verifiable. |
| Methods: model identifiers | **3b** | State whether each evaluated chatbot/model is open source or proprietary. | Partial | The evaluated services are provider-hosted consumer web platforms; the exact backend model status could not be independently verified. |
| Methods: model details | **4a** | Clarify whether the study used an existing base chatbot/model or an author-developed, tuned, or fine-tuned system. | Covered | All four evaluated systems were existing public web platforms; the authors did not train, fine-tune, or otherwise modify the underlying models. |
| Methods: model details | **4b** | For an existing base model, provide sufficient identification or citations where feasible. | Covered | Platform and provider identities, official access routes, testing period, subscription tier, and user-accessible settings are reported. No unsupported backend version identifier is asserted. |
| Methods: model details | **4c** | For a newly developed or modified model, describe training or deployment data and parameters. | Not applicable | No new, tuned, or fine-tuned model was developed. |
| Methods: prompt engineering | **5a** | Describe how the study prompt was developed and whether it evolved through testing. | Covered | The prompt was derived from guideline domains, finalized before testing, and not iteratively modified using model outputs. |
| Methods: prompt engineering | **5ai** | Identify the sources used to construct the prompts. | Covered | International cirrhosis nutrition guidelines and scenario-specific clinical requirements informed prompt and case construction. |
| Methods: prompt engineering | **5aii** | Report the number and relevant expertise of people involved in prompt development. | Partial | Clinical specialties are reported; the exact number of prompt-development contributors is not separately stated. |
| Methods: prompt engineering | **5aiii** | Describe any patient or public involvement in prompt development. | Not reported | Not reported. |
| Methods: prompt engineering | **5b** | Provide the complete study prompts. | Covered | The standardized English prompt and all 36 simulated cases are provided. |
| Methods: query strategy | **6a** | Report how the chatbots/models were accessed. | Covered | Official provider websites and free-tier accounts are specified; no API was used. |
| Methods: query strategy | **6b** | Report query dates and geographic location, including city and country. | Partial | The testing period (May 1-7, 2026) is reported. |
| Methods: query strategy | **6c** | Explain whether prompts were entered in separate chat sessions and how sessions were managed. | Covered | A new conversation was opened for each case; memory, personalization, and custom instructions were not used. |
| Methods: query strategy | **6d** | Make all chatbot responses available. | Covered | Verbatim normalized responses from all 144 model-case interactions are provided. |
| Methods: performance evaluation | **7a** | Define the reference standard used to judge successful performance. | Covered | The finalized and locked guideline-based codebook and operational definitions used for the de novo model-blinded assessment form the reference standard. |
| Methods: performance evaluation | **7b** | Describe the evaluation procedure in sufficient detail for replication. | Covered | Normalization, de novo model-blinded scoring, locked reviewer instructions, evidence requirements, response-level aggregation, and inter-reviewer reliability procedures are reported. |
| Methods: performance evaluation | **7bi** | Report the number and expertise of evaluators. | Covered | Reviewer specialties, senior status, experience, and dietitian credentials are reported. |
| Methods: performance evaluation | **7bii** | Describe any patient or public involvement in response evaluation. | Not reported | Not reported. |
| Methods: performance evaluation | **7biii** | State whether evaluators were blinded to chatbot identity. | Covered | Reviewers received independently randomized anonymous response booklets and were blinded to model identity. |
| Methods: sample size | **8** | Explain how the number of cases and responses was determined. | Covered | The pragmatic case-count rationale and precision limitations are reported. |
| Methods: data analysis | **9a** | Describe statistical methods, including any assessment of response reproducibility. | Covered | Paired analyses preserve the matched case structure. Percentile bootstrap confidence intervals for paired comparative effects use 3,000 case-level resamples, retaining all four platform responses for each sampled case; the single-generation design and absence of repeated-response reproducibility testing are also stated. |
| Methods: data analysis | **9ai** | List the performance measures and safety outcomes. | Covered | Guideline Adherence, Error Count, Any Major Error, expert-rated Harm Flag, Clinically Appropriate Food Examples, and Word Count are defined, with potential safety concerns kept distinct from observed clinical harm. |
| Results | **10a** | Report model performance against the reference standard. | Covered | Overall and scenario-specific performance of the four web platforms is reported. |
| Results | **10b** | Describe how nonconforming responses differed from the reference standard. | Covered | Item-level nonadherence and error categories are described, particularly protein management after hepatic encephalopathy. |
| Results | **10c** | Report potentially harmful, biased, or misleading responses. | Covered | Any Major Error, Harm Flag, error categories, paired comparisons, and confidence intervals are reported as expert-rated indicators of potential safety concerns rather than evidence of observed patient harm. |
| Discussion | **11a** | Interpret findings in relation to existing evidence. | Covered | Findings are interpreted against cirrhosis guidelines and prior clinical nutrition and LLM evaluations. |
| Discussion | **11b** | Discuss strengths and limitations. | Covered | Strengths and limitations address structured prompting, English-language testing, single generation, sparse potential safety-concern events, ceiling effects, lack of external validation, and the inability to verify exact backend deployments. |
| Discussion | **11c** | Discuss implications for practice, education, policy or regulation, and future research as relevant. | Covered | Clinical verification, a concise human-in-the-loop workflow, and priorities for future research are discussed. |
| Open science: disclosures | **12a** | Report relevant author conflicts of interest. | Covered | All authors declare that they have no competing interests. |
| Open science: funding | **12b** | Report funding sources and describe the funders role in the study and reporting. | Covered | Funding sources are reported, together with an explicit statement that the funders had no role in study design, data collection, expert assessment, analysis, interpretation, manuscript preparation, or the decision to submit. |
| Open science: ethics | **12c** | Describe ethical review or the reason formal review was not required. | Covered | The manuscript explains why ethics review and informed consent were not required. |
| Open science: privacy | **12ci** | Describe safeguards for patient health information where applicable. | Covered | No real patient information or identifiable health data were used or entered into a chatbot. |
| Open science: permissions | **12cii** | State whether permissions or licences were required for original copyrighted data. | Not applicable | No original copyrighted data were used. |
| Open science: protocol | **12d** | Provide or identify the study protocol. | Partial | The manuscript states that the study was not prospectively registered and that no protocol was publicly available before data collection. Supplementary Files A and C provide the final codebook and study cases/prompts but do not constitute a prospectively registered protocol. |
| Open science: data availability | **12e** | State where data, code, and accessible model information can be obtained. | Partial | The manuscript identifies the locations of the verbatim responses, reviewer-level data, response-level data, locked codebook, cases and prompts, web-platform testing characteristics, pairwise analyses, and supporting results. A separate analysis-code file is not listed in the current supplementary package. |
