# Supplementary material for: Guideline adherence and expert-rated potential safety concerns in AI-generated dietary advice for patients with cirrhosis: a comparative evaluation of large language models
Source: Front Nutr. 2026 Oct 8;13:1893597. doi: 10.3389/fnut.2026.1893597 (PMC13689687; doi:10.3389/fnut.2026.1893597)
Supplement: Supplementary file 1 [file Supplementary_file_1.zip › Supplementary Data S1 (Verbatim Model Responses).ZIP!/answer via qwen.docx]

## C101

**Dietary and Nutritional Recommendations for Compensated HBV-Related Cirrhosis**

**Patient Profile:** Male, 34 years old, 172 cm, 68 kg (BMI 23.0), HBV-related compensated cirrhosis, clinically stable, no ascites or hepatic encephalopathy, normal appetite.

**1. Overall Dietary Principles**

**Adequate energy intake**: Target **25-35 kcal/kg/day** (approximately 1,700-2,400 kcal/day for this patient) to maintain lean body mass and prevent catabolism

.

**No protein restriction**: Protein should NOT be restricted in compensated cirrhosis; adequate protein is essential to preserve muscle mass and prevent sarcopenia

.

**Balanced macronutrients**: Emphasize complex carbohydrates, healthy fats (especially omega-3 sources), and high-quality protein sources.

**Food safety**: Avoid raw/undercooked shellfish, fish, meat, and unpasteurized dairy products due to increased infection risk

.

**Alcohol abstinence**: Complete avoidance of alcohol is mandatory in HBV-related cirrhosis to prevent disease progression.

**2. Recommended Daily Protein Intake**

| **Parameter** | **Recommendation** |
| --- | --- |
| **Target protein intake** | **1.2–1.5 g/kg ideal body weight/day** |
| **For this patient** | **82–102 g protein/day** (based on 68 kg) |
| **Protein distribution** | Spread evenly across meals; include a late-evening protein snack |
| **Preferred sources** | Plant-based proteins (legumes, tofu, nuts), dairy, fish, poultry; limit red/processed meats |

**Key Point**: Higher intake of total and dairy protein and lower animal protein consumption are associated with reduced mortality in cirrhosis

.

**3. Meal Frequency and Eating Pattern**

**Avoid prolonged fasting**: Limit fasting intervals to **no more than 3-4 hours during waking hours** to prevent muscle catabolism

.

**Meal pattern recommendation**:

- **3 main meals + 2-3 snacks** daily
- **Include a late-evening snack** (e.g., Greek yogurt, cottage cheese, or a small protein shake) to reduce overnight protein breakdown
- **Early breakfast** within 1-2 hours of waking

**Rationale**: Patients with cirrhosis have accelerated starvation metabolism; frequent feeding helps maintain nitrogen balance and muscle mass.

**4. Specific Food Recommendations and Restrictions**

**Encourage:**

| **Food Group** | **Examples** | **Rationale** |
| --- | --- | --- |
| **Plant proteins** | Lentils, chickpeas, tofu, tempeh, edamame | Associated with lower mortality; well-tolerated |
| **Dairy** | Greek yogurt, cottage cheese, milk | High-quality protein; may improve outcomes |
| **Fish** | Salmon, mackerel, sardines (cooked) | Omega-3 fatty acids; anti-inflammatory |
| **Whole grains** | Oats, brown rice, quinoa, whole-wheat bread | Fiber supports gut health; steady energy |
| **Fruits & vegetables** | Colorful variety, well-washed | Antioxidants, vitamins, fiber |
| **Healthy fats** | Olive oil, avocado, nuts, seeds | Support calorie density without excess saturated fat |

**Limit or Avoid:**

| **Item** | **Reason** |
| --- | --- |
| **Raw/undercooked seafood, meat, eggs** | Risk of severe infection in immunocompromised liver patients |
| **Unpasteurized dairy/juices** | Risk of bacterial contamination |
| **Excess sodium** | While strict restriction isn't needed without ascites, avoid >2,300 mg/day to prevent future fluid retention |
| **Iron supplements & iron-rich organ meats** | HBV patients with cirrhosis may have elevated iron stores; avoid excess unless deficiency confirmed |
| **High-dose herbal supplements** | Many herbs (e.g., kava, comfrey) are hepatotoxic; consult physician before use |
| **Processed meats & excessive red meat** | Associated with higher mortality in cirrhosis |

**5. Additional Nutritional Considerations**

**Micronutrient Monitoring**

- **Vitamin D**: Screen annually; supplement if <20 ng/mL to reach >30 ng/mL
- **Zinc**: Deficiency may contribute to dysgeusia and impaired immunity; consider testing if taste changes occur
- **B-vitamins (especially thiamine)**: Important in chronic liver disease; ensure adequate intake through diet or supplementation if needed

**Physical Activity**

- Combine **resistance training** (2-3x/week) with **aerobic exercise** to preserve muscle mass and combat sarcopenia
- Even light activity (walking, stretching) is beneficial if deconditioned

**Monitoring & Follow-up**

- **Weight and muscle mass**: Track monthly; unintentional weight loss warrants nutritional reassessment
- **Screen for malnutrition**: Use validated tools (e.g., RFH-NPT) annually or with clinical changes
- **Consider dietitian referral**: For personalized meal planning and ongoing support

**HBV-Specific Considerations**

- Ensure antiviral therapy adherence per hepatology guidance
- Maintain healthy weight to reduce metabolic stress on the liver
- Avoid unnecessary medications/supplements that require hepatic metabolism

**Sample Daily Meal Pattern (≈2,000 kcal, 90g protein)**

| **Time** | **Meal** | **Example** |
| --- | --- | --- |
| 7:30 AM | Breakfast | Oatmeal with Greek yogurt, berries, walnuts |
| 10:00 AM | Snack | Apple with almond butter |
| 12:30 PM | Lunch | Grilled salmon, quinoa, steamed broccoli, olive oil dressing |
| 3:30 PM | Snack | Cottage cheese with pineapple |
| 6:30 PM | Dinner | Chicken stir-fry with tofu, brown rice, mixed vegetables |
| 9:00 PM | Evening snack | Small protein shake or casein pudding |

**Important**: These recommendations apply to **compensated** cirrhosis. If the patient develops ascites, hepatic encephalopathy, or other decompensation, dietary management should be promptly re-evaluated by a hepatology team and registered dietitian specializing in liver disease.

## C1-02

**Dietary and Nutritional Recommendations for Compensated MASLD-Related Cirrhosis**

**Patient Profile:** Female, 39 years old, 160 cm, 54 kg (BMI 21.1 kg/m²), MASLD-related compensated cirrhosis, clinically stable, normal appetite.

**1. Overall Dietary Principles**

**Adopt a Mediterranean-style dietary pattern**: Emphasize vegetables, fruits, whole grains, legumes, nuts, olive oil, and fish while limiting red/processed meats and refined carbohydrates. This pattern is associated with reduced liver fibrosis, improved insulin sensitivity, and lower inflammation in MASLD

.

**Adequate energy intake**: Target **25-35 kcal/kg/day** (approximately 1,350-1,900 kcal/day) to maintain current weight and preserve lean muscle mass. Weight loss is NOT indicated given normal BMI

.

**No protein restriction**: Maintain adequate protein to prevent sarcopenia, which is strongly associated with poor outcomes in cirrhosis

.

**Prioritize carbohydrate quality**: Choose low-glycemic-index carbohydrates (oats, quinoa, legumes, non-starchy vegetables) to improve insulin sensitivity and reduce hepatic

.

**Alcohol abstinence**: Complete avoidance is essential, as even modest alcohol intake accelerates fibrosis progression in MASLD-related cirrhosis.

**Food safety**: Avoid raw/undercooked seafood, meat, eggs, and unpasteurized products due to increased infection risk in chronic liver disease

.

**2. Recommended Daily Protein Intake**

| **Parameter** | **Recommendation** |
| --- | --- |
| **Target protein intake** | **1.2–1.5 g/kg ideal body weight/day** |
| **For this patient** | **65–81 g protein/day** (based on 54 kg) |
| **Protein distribution** | Spread evenly across meals; include late-evening protein snack |
| **Preferred sources** | Plant-based proteins (legumes, tofu, tempeh), fish, poultry, low-fat dairy; limit red/processed meats |

**MASLD-specific note**: Higher intake of plant-based protein is associated with improved metabolic parameters and reduced liver fat accumulation compared to animal protein sources

.

**3. Meal Frequency and Eating Pattern**

**Avoid prolonged fasting**: Limit fasting intervals to **≤3-4 hours during waking hours** to prevent catabolism and maintain nitrogen balance

.

**Recommended meal pattern**:

- **3 balanced main meals + 2-3 snacks** daily
- **Include a late-evening snack** (e.g., Greek yogurt, cottage cheese, or small protein shake) to reduce overnight muscle breakdown
- **Early breakfast** within 1-2 hours of waking to break the overnight fast

**Rationale**: Patients with cirrhosis have accelerated "accelerated starvation" metabolism; frequent feeding helps preserve muscle mass and supports metabolic stability, particularly important in MASLD where insulin resistance is a key driver

discovery.dundee.ac.uk

.

**4. Specific Food Recommendations and Restrictions**

**Encourage:**

| **Food Group** | **Examples** | **Rationale** |
| --- | --- | --- |
| **Plant proteins** | Lentils, chickpeas, tofu, tempeh, edamame | Associated with improved insulin sensitivity and lower liver fat |
| **Fatty fish** | Salmon, mackerel, sardines (cooked) | Omega-3 fatty acids reduce hepatic inflammation and triglycerides |
| **Whole grains** | Oats, quinoa, brown rice, whole-wheat bread | Fiber improves glycemic control and gut health |
| **Non-starchy vegetables** | Leafy greens, broccoli, peppers, tomatoes | Low energy density, high antioxidants, support metabolic health |
| **Healthy fats** | Extra-virgin olive oil, avocado, nuts, seeds | Monounsaturated fats improve lipid profiles and reduce inflammation |
| **Coffee** | 2-3 cups/day (if tolerated) | Associated with reduced fibrosis progression and lower HCC risk in chronic liver disease |

**Limit or Avoid:**

| **Item** | **Reason** |
| --- | --- |
| **Added sugars & sugar-sweetened beverages** | Fructose promotes hepatic de novo lipogenesis and worsens MASLD |
| **Refined carbohydrates** | White bread, pastries, sugary cereals worsen insulin resistance |
| **Processed meats & excessive red meat** | Associated with higher inflammation and mortality in liver disease |
| **Saturated and trans fats** | Found in fried foods, baked goods; worsen dyslipidemia and hepatic fat |
| **Raw/undercooked animal products** | Risk of severe infection in immunocompromised liver patients |
| **Unpasteurized dairy/juices** | Risk of bacterial contamination |
| **Hepatotoxic supplements** | Avoid kava, comfrey, high-dose green tea extract, or unregulated herbal products |
| **Excess sodium** | While strict restriction not needed without ascites, aim for <2,300 mg/day to prevent future fluid retention |

**5. Additional Nutritional Considerations**

**Metabolic & Micronutrient Monitoring**

- **Vitamin D**: Screen annually; deficiency is common in chronic liver disease and associated with worse outcomes. Supplement if <20 ng/mL to reach >30 ng/mL .
- **Zinc**: Deficiency may contribute to dysgeusia and impaired immunity; consider testing if taste changes occur.
- **B-vitamins**: Ensure adequate intake, particularly thiamine, especially if dietary variety is limited.
- **Iron status**: Monitor ferritin and transferrin saturation; avoid iron supplementation unless deficiency is confirmed, as iron overload may worsen liver injury.

**Physical Activity**

- Combine **resistance training** (2-3x/week) with **aerobic exercise** (150 min/week moderate intensity) to improve insulin sensitivity, preserve muscle mass, and reduce hepatic fat .
- Even light activity (walking, stretching) is beneficial if deconditioned.

**Weight Management**

- **Maintain current weight**: Given normal BMI (21.1), intentional weight loss is not indicated and could risk muscle loss.
- Focus on **body composition**: Prioritize preservation of lean mass over scale weight.

**Monitoring & Follow-up**

- **Weight and muscle mass**: Track monthly; unintentional weight loss warrants nutritional reassessment.
- **Screen for malnutrition**: Use validated tools (e.g., RFH-NPT) annually or with clinical changes

.

- **Consider dietitian referral**: For personalized meal planning, especially to balance MASLD metabolic goals with cirrhosis nutritional needs.

**MASLD-Specific Considerations**

- **Address insulin resistance**: Dietary pattern should support glycemic control; coordinate with endocrinology if prediabetes/diabetes present.
- **Coffee consumption**: 2-3 cups/day of filtered coffee is associated with reduced fibrosis progression and may be encouraged if tolerated .
- **Avoid unnecessary medications/supplements**: Minimize hepatic metabolic burden; review all medications with hepatology team.

**Sample Daily Meal Pattern (≈1,700 kcal, 75g protein)**

| **Time** | **Meal** | **Example** |
| --- | --- | --- |
| 7:30 AM | Breakfast | Oatmeal with berries, chia seeds, and a boiled egg |
| 10:00 AM | Snack | Small apple with 1 tbsp almond butter |
| 12:30 PM | Lunch | Lentil salad with mixed vegetables, olive oil-lemon dressing, whole-grain pita |
| 3:30 PM | Snack | Greek yogurt with walnuts |
| 6:30 PM | Dinner | Baked salmon, quinoa, roasted broccoli and carrots with olive oil |
| 9:00 PM | Evening snack | Cottage cheese or small casein protein shake |

**Important**: These recommendations apply to **compensated** MASLD-related cirrhosis. If the patient develops ascites, hepatic encephalopathy, or other decompensation, dietary management should be promptly re-evaluated by a hepatology team and registered dietitian specializing in liver disease.

## C1-03

**Dietary and Nutritional Recommendations for Compensated Alcohol-Related Cirrhosis**

**Patient Profile:** Male, 42 years old, 168 cm, 82 kg (BMI 29.1 kg/m², overweight), alcohol-related compensated cirrhosis, clinically stable, no recent hospitalization.

**1. Overall Dietary Principles**

**Complete alcohol abstinence**: Absolute avoidance of alcohol is the single most critical intervention to halt disease progression, reduce mortality, and allow potential hepatic recovery .

**Adequate but moderated energy intake**: Target **25-30 kcal/kg ideal body weight/day** (approximately 1,600-1,900 kcal/day using adjusted body weight) to support gradual weight normalization while preserving lean muscle mass. Avoid aggressive caloric restriction that could worsen sarcopenia .

**No protein restriction**: Maintain high-quality protein intake to combat the high risk of sarcopenia and malnutrition characteristic of alcohol-related liver disease .

**Prioritize nutrient-dense foods**: Focus on whole foods rich in vitamins, minerals, and antioxidants to address common deficiencies associated with chronic alcohol use.

**Food safety**: Avoid raw/undercooked seafood, meat, eggs, and unpasteurized products due to increased infection risk in chronic liver disease .

**Multidisciplinary support**: Integrate nutritional counseling with addiction medicine, mental health support, and hepatology follow-up for optimal outcomes.

**2. Recommended Daily Protein Intake**

| **Parameter** | **Recommendation** |
| --- | --- |
| **Target protein intake** | **1.2–1.5 g/kg ideal body weight/day** |
| **For this patient** | **75–95 g protein/day** (based on ideal body weight ~63 kg for height 168 cm) |
| **Protein distribution** | Spread evenly across meals; include late-evening protein snack |
| **Preferred sources** | Plant-based proteins (legumes, tofu), fish, poultry, eggs, low-fat dairy; limit red/processed meats |

**Alcohol-related cirrhosis note**: Patients with alcohol-related liver disease have particularly high rates of protein-energy malnutrition and sarcopenia; adequate protein intake is essential for muscle preservation and immune function .

**3. Meal Frequency and Eating Pattern**

**Avoid prolonged fasting**: Limit fasting intervals to **≤3-4 hours during waking hours** to prevent accelerated catabolism, which is heightened in alcohol-related liver disease .

**Recommended meal pattern**:

- **3 balanced main meals + 2-3 snacks** daily
- **Include a late-evening snack** (e.g., Greek yogurt, cottage cheese, or small protein shake) to reduce overnight muscle breakdown
- **Early breakfast** within 1-2 hours of waking to break the overnight fast

**Rationale**: Alcohol-related cirrhosis is associated with profound metabolic derangements including accelerated starvation metabolism, impaired glycogen storage, and increased protein catabolism. Frequent feeding helps maintain nitrogen balance and muscle mass .

**4. Specific Food Recommendations and Restrictions**

**Encourage:**

| **Food Group** | **Examples** | **Rationale** |
| --- | --- | --- |
| **Plant proteins** | Lentils, chickpeas, tofu, tempeh, edamame | Well-tolerated, associated with lower mortality in cirrhosis |
| **Lean animal proteins** | Fish, poultry, eggs, low-fat dairy | High-quality protein to support muscle synthesis |
| **Whole grains** | Oats, quinoa, brown rice, whole-wheat bread | Fiber supports gut health; steady energy release |
| **Colorful fruits & vegetables** | Berries, leafy greens, cruciferous vegetables | Antioxidants combat oxidative stress from prior alcohol exposure |
| **Healthy fats** | Olive oil, avocado, nuts, seeds | Support calorie density without excess saturated fat |
| **Thiamine-rich foods** | Whole grains, legumes, pork, fortified cereals | Address high risk of thiamine deficiency in alcohol-related liver disease |

**Limit or Avoid:**

| **Item** | **Reason** |
| --- | --- |
| **All alcohol** | Even small amounts accelerate fibrosis, increase HCC risk, and worsen outcomes |
| **Raw/undercooked seafood, meat, eggs** | Risk of severe infection (e.g., Vibrio, Salmonella) in immunocompromised liver patients |
| **Unpasteurized dairy/juices** | Risk of bacterial contamination |
| **Excess sodium** | While strict restriction not needed without ascites, aim for <2,300 mg/day to prevent future fluid retention |
| **High-dose herbal supplements** | Many herbs (kava, comfrey, high-dose green tea extract) are hepatotoxic; avoid unless approved by hepatology |
| **Processed meats & excessive red meat** | Associated with higher inflammation and mortality in liver disease |
| **Excess added sugars** | May contribute to hepatic fat accumulation and metabolic dysfunction |

**5. Additional Nutritional Considerations**

**Critical Micronutrient Screening & Supplementation**

| **Nutrient** | **Rationale** | **Recommendation** |
| --- | --- | --- |
| **Thiamine (B1)** | Alcohol impairs absorption and storage; deficiency can cause Wernicke-Korsakoff syndrome | **Supplement prophylactically**: 100 mg/day oral thiamine; IV if malnourished or acute withdrawal |
| **Folate (B9)** | Alcohol reduces absorption and increases excretion | Screen levels; supplement 1 mg/day if deficient |
| **Vitamin B12** | Malabsorption common in chronic liver disease | Screen annually; supplement if deficient |
| **Vitamin D** | Deficiency prevalent in chronic liver disease; associated with worse outcomes | Screen annually; supplement to maintain >30 ng/mL |
| **Zinc** | Deficiency contributes to dysgeusia, impaired immunity, and poor wound healing | Consider supplementation (25-50 mg elemental zinc/day) if deficient or symptomatic |
| **Magnesium** | Alcohol increases renal excretion; deficiency common | Screen if muscle cramps, arrhythmias, or weakness present |

**Physical Activity & Body Composition**

- **Prioritize resistance training** (2-3x/week) combined with moderate aerobic activity to combat sarcopenia and improve insulin sensitivity .
- Focus on **preserving lean mass** rather than weight loss alone; unintentional muscle loss worsens prognosis in cirrhosis.
- Given BMI 29.1, **gradual weight normalization** (0.25-0.5 kg/week) may be appropriate if accompanied by muscle preservation, but avoid aggressive caloric restriction.

**Monitoring & Follow-up**

- **Weight and mid-arm muscle circumference**: Track monthly to distinguish fat loss from muscle loss.
- **Screen for malnutrition**: Use validated tools (e.g., RFH-NPT, SARC-F) at each visit .
- **Laboratory monitoring**: Albumin, prealbumin, micronutrient panels every 3-6 months.
- **Multidisciplinary referral**: Strongly consider referral to registered dietitian, addiction specialist, and mental health support.

**Alcohol-Related Cirrhosis–Specific Considerations**

- **Thiamine before glucose**: If patient experiences acute illness or requires IV fluids, administer thiamine before dextrose to prevent precipitating Wernicke's encephalopathy .
- **Withdrawal support**: Ensure patient has access to medical support for alcohol withdrawal if needed; nutritional support is adjunctive, not primary treatment.
- **Relapse prevention**: Integrate nutritional counseling with behavioral strategies; poor nutrition can trigger cravings and relapse.

**Sample Daily Meal Pattern (≈1,800 kcal, 85g protein)**

| **Time** | **Meal** | **Example** |
| --- | --- | --- |
| 7:30 AM | Breakfast | Scrambled eggs with spinach, whole-wheat toast, orange |
| 10:00 AM | Snack | Greek yogurt with berries and a sprinkle of walnuts |
| 12:30 PM | Lunch | Grilled chicken salad with mixed greens, chickpeas, olive oil-lemon dressing, quinoa |
| 3:30 PM | Snack | Hummus with carrot/cucumber sticks and whole-grain crackers |
| 6:30 PM | Dinner | Baked cod, sweet potato, steamed green beans with olive oil |
| 9:00 PM | Evening snack | Cottage cheese or small casein protein shake |

*Note: Thiamine supplement (100 mg) to be taken daily with food, separate from other medications as advised.*

**Important**: These recommendations apply to **compensated** alcohol-related cirrhosis. If the patient develops ascites, hepatic encephalopathy, acute alcoholic hepatitis, or other decompensation, dietary management must be promptly re-evaluated by a hepatology team and registered dietitian specializing in liver disease. **Alcohol abstinence remains the cornerstone of management**—nutritional support is adjunctive.

## C1-04

**Dietary and Nutritional Recommendations for Compensated HBV-Related Cirrhosis**

**Patient Profile:** Female, 44 years old, 158 cm, 70 kg (BMI 28.0 kg/m², overweight), HBV-related compensated cirrhosis, stable outpatient follow-up.

**1. Overall Dietary Principles**

**Adopt a Mediterranean-style dietary pattern**: Emphasize vegetables, fruits, whole grains, legumes, nuts, olive oil, and fish while limiting red/processed meats and refined carbohydrates. This pattern supports liver health, reduces inflammation, and aids modest weight management .

**Moderate energy intake for gradual weight normalization**: Target **25-30 kcal/kg ideal body weight/day** (approximately 1,400-1,700 kcal/day using adjusted body weight) to support slow, sustainable weight loss (0.25-0.5 kg/week) while preserving lean muscle mass. Avoid aggressive caloric restriction that could worsen sarcopenia .

**No protein restriction**: Maintain adequate high-quality protein intake to prevent sarcopenia, which is strongly associated with poor outcomes in cirrhosis regardless of BMI .

**Prioritize carbohydrate quality**: Choose low-glycemic-index carbohydrates (oats, quinoa, legumes, non-starchy vegetables) to improve insulin sensitivity and reduce metabolic stress on the liver .

**Alcohol abstinence**: Complete avoidance of alcohol is mandatory in HBV-related cirrhosis to prevent disease progression and reduce hepatocellular carcinoma risk.

**Food safety**: Avoid raw/undercooked seafood, meat, eggs, and unpasteurized products due to increased infection risk in chronic liver disease .

**2. Recommended Daily Protein Intake**

| **Parameter** | **Recommendation** |
| --- | --- |
| **Target protein intake** | **1.2–1.5 g/kg ideal body weight/day** |
| **For this patient** | **65–80 g protein/day** (based on ideal body weight ~54 kg for height 158 cm) |
| **Protein distribution** | Spread evenly across meals; include late-evening protein snack |
| **Preferred sources** | Plant-based proteins (legumes, tofu, tempeh), fish, poultry, eggs, low-fat dairy; limit red/processed meats |

**Key Point**: Higher intake of plant-based and dairy protein is associated with reduced mortality in cirrhosis. Adequate protein is essential to preserve muscle mass during modest weight loss .

**3. Meal Frequency and Eating Pattern**

**Avoid prolonged fasting**: Limit fasting intervals to **≤3-4 hours during waking hours** to prevent accelerated catabolism and maintain nitrogen balance .

**Recommended meal pattern**:

- **3 balanced main meals + 2-3 snacks** daily
- **Include a late-evening snack** (e.g., Greek yogurt, cottage cheese, or small protein shake) to reduce overnight muscle breakdown
- **Early breakfast** within 1-2 hours of waking to break the overnight fast

**Rationale**: Patients with cirrhosis have accelerated "starvation metabolism" with impaired glycogen storage. Frequent feeding helps preserve muscle mass and supports metabolic stability, particularly important when pursuing modest weight normalization .

**4. Specific Food Recommendations and Restrictions**

**Encourage:**

| **Food Group** | **Examples** | **Rationale** |
| --- | --- | --- |
| **Plant proteins** | Lentils, chickpeas, tofu, tempeh, edamame | Associated with lower mortality; well-tolerated; support satiety during weight management |
| **Fatty fish** | Salmon, mackerel, sardines (cooked) | Omega-3 fatty acids reduce hepatic inflammation; high-quality protein |
| **Non-starchy vegetables** | Leafy greens, broccoli, peppers, tomatoes | Low energy density, high fiber and antioxidants; support weight management |
| **Whole grains** | Oats, quinoa, brown rice, whole-wheat bread | Fiber improves glycemic control and gut health; steady energy release |
| **Healthy fats** | Extra-virgin olive oil, avocado, nuts, seeds | Monounsaturated fats improve lipid profiles; support calorie control with nutrient density |
| **Coffee** | 2-3 cups/day filtered (if tolerated) | Associated with reduced fibrosis progression and lower HCC risk in chronic liver disease |

**Limit or Avoid:**

| **Item** | **Reason** |
| --- | --- |
| **All alcohol** | Accelerates fibrosis, increases HCC risk, worsens outcomes in HBV cirrhosis |
| **Raw/undercooked seafood, meat, eggs** | Risk of severe infection (e.g., Vibrio, Salmonella) in immunocompromised liver patients |
| **Sugar-sweetened beverages & added sugars** | Promote hepatic fat accumulation and worsen insulin resistance |
| **Refined carbohydrates** | White bread, pastries, sugary cereals worsen glycemic control |
| **Processed meats & excessive red meat** | Associated with higher inflammation and mortality in liver disease |
| **Excess sodium** | While strict restriction not needed without ascites, aim for <2,300 mg/day to prevent future fluid retention |
| **Hepatotoxic supplements** | Avoid kava, comfrey, high-dose green tea extract, or unregulated herbal products |
| **Unpasteurized dairy/juices** | Risk of bacterial contamination |

**5. Additional Nutritional Considerations**

**Micronutrient Monitoring & Supplementation**

| **Nutrient** | **Rationale** | **Recommendation** |
| --- | --- | --- |
| **Vitamin D** | Deficiency common in chronic liver disease; associated with worse outcomes and immune dysfunction | Screen annually; supplement if <20 ng/mL to reach >30 ng/mL |
| **Zinc** | Deficiency may contribute to dysgeusia, impaired immunity, and poor wound healing | Consider testing if taste changes occur; supplement if deficient |
| **B-vitamins (especially B12, folate)** | Malabsorption possible in chronic liver disease | Screen annually; supplement if deficient |
| **Iron status** | Monitor ferritin and transferrin saturation; avoid iron supplementation unless deficiency confirmed, as excess iron may worsen liver injury | Check baseline; recheck if clinically indicated |

**Physical Activity & Body Composition**

- **Combine resistance training** (2-3x/week) with **moderate aerobic activity** (150 min/week) to preserve lean mass during weight normalization, improve insulin sensitivity, and reduce hepatic fat .
- Focus on **body composition over scale weight**: Prioritize preservation of muscle mass; unintentional muscle loss worsens prognosis in cirrhosis.
- Given BMI 28.0, **gradual weight normalization** (0.25-0.5 kg/week) is appropriate if accompanied by muscle preservation and improved metabolic parameters.

**Monitoring & Follow-up**

- **Weight and mid-arm muscle circumference**: Track monthly to distinguish fat loss from muscle loss.
- **Screen for malnutrition**: Use validated tools (e.g., RFH-NPT) annually or with clinical changes .
- **Laboratory monitoring**: Albumin, prealbumin, micronutrient panels every 6-12 months in stable compensated disease.
- **Consider dietitian referral**: For personalized meal planning that balances HBV cirrhosis nutritional needs with modest weight management goals.

**HBV-Specific Considerations**

- **Antiviral therapy adherence**: Ensure ongoing adherence to HBV antiviral therapy per hepatology guidance; nutrition supports but does not replace medical treatment.
- **HCC surveillance**: Maintain recommended imaging/biomarker surveillance; healthy dietary patterns may modestly reduce HCC risk .
- **Avoid unnecessary medications/supplements**: Minimize hepatic metabolic burden; review all medications and supplements with hepatology team.

**Sample Daily Meal Pattern (≈1,550 kcal, 70g protein)**

| **Time** | **Meal** | **Example** |
| --- | --- | --- |
| 7:30 AM | Breakfast | Greek yogurt with berries, chia seeds, and a small handful of almonds |
| 10:00 AM | Snack | Apple with 1 tbsp peanut butter |
| 12:30 PM | Lunch | Large salad with grilled chicken, chickpeas, mixed vegetables, olive oil-lemon dressing, small whole-grain roll |
| 3:30 PM | Snack | Cottage cheese with cucumber slices |
| 6:30 PM | Dinner | Baked salmon, quinoa, roasted broccoli and zucchini with olive oil |
| 9:00 PM | Evening snack | Small casein protein shake or warm milk with turmeric |

*Note: Adjust portion sizes based on hunger/fullness cues and weight trajectory; prioritize protein at each meal.*

**Important**: These recommendations apply to **compensated** HBV-related cirrhosis. If the patient develops ascites, hepatic encephalopathy, or other decompensation, dietary management should be promptly re-evaluated by a hepatology team and registered dietitian specializing in liver disease. **Weight management should never compromise protein intake or muscle preservation** in cirrhosis.

## C1-05

**Dietary and Nutritional Recommendations for Compensated MASLD-Related Cirrhosis with Overweight**

**Patient Profile:** Male, 47 years old, 175 cm, 92 kg (BMI 30.0 kg/m², obese class I), MASLD-related compensated cirrhosis, clinically stable.

**1. Overall Dietary Principles**

**Adopt a Mediterranean-style dietary pattern**: Emphasize vegetables, fruits, whole grains, legumes, nuts, olive oil, and fish while limiting red/processed meats and refined carbohydrates. This pattern is associated with reduced liver fat, improved insulin sensitivity, and lower fibrosis progression in MASLD

.

**Moderate caloric restriction for gradual weight loss**: Target **25-30 kcal/kg ideal body weight/day** (approximately 1,750-2,100 kcal/day using ideal body weight ~70 kg) to support sustainable weight loss of **0.25-0.5 kg/week**. Aggressive restriction risks muscle loss and worsens sarcopenia

.

**Weight loss goal**: Aim for **7-10% total body weight loss** (~6-9 kg) over 6-12 months, which is associated with improved liver histology and metabolic parameters in MASLD, provided muscle mass is preserved

.

**No protein restriction**: Maintain high-quality protein intake to prevent sarcopenia, which is a strong independent predictor of mortality in cirrhosis regardless of BMI

.

**Prioritize carbohydrate quality**: Choose low-glycemic-index carbohydrates (oats, quinoa, legumes, non-starchy vegetables) to improve insulin sensitivity and reduce hepatic de novo lipogenesis

.

**Complete alcohol abstinence**: Essential in MASLD-related cirrhosis to prevent accelerated fibrosis and reduce hepatocellular carcinoma risk.

**Food safety**: Avoid raw/undercooked seafood, meat, eggs, and unpasteurized products due to increased infection risk in chronic liver disease

.

**2. Recommended Daily Protein Intake**

| **Parameter** | **Recommendation** |
| --- | --- |
| **Target protein intake** | **1.2–1.5 g/kg ideal body weight/day** |
| **For this patient** | **84–105 g protein/day** (based on ideal body weight ~70 kg for height 175 cm) |
| **Protein distribution** | Spread evenly across meals (25-30 g/meal); include late-evening protein snack |
| **Preferred sources** | Plant-based proteins (legumes, tofu, tempeh), fish, poultry, eggs, low-fat dairy; limit red/processed meats |

**Key Point**: In patients with cirrhosis and overweight/obesity, higher protein intake during weight loss helps preserve lean mass while promoting fat loss. Plant-based proteins are particularly beneficial for MASLD due to favorable effects on insulin sensitivity

**3. Meal Frequency and Eating Pattern**

**Avoid prolonged fasting**: Limit fasting intervals to **≤3-4 hours during waking hours** to prevent accelerated catabolism and maintain nitrogen balance

.

**Recommended meal pattern**:

- **3 balanced main meals + 2-3 snacks** daily
- **Include a late-evening snack** (e.g., Greek yogurt, cottage cheese, or small protein shake) to reduce overnight muscle breakdown
- **Early breakfast** within 1-2 hours of waking to break the overnight fast

**Rationale**: Patients with cirrhosis have impaired glycogen storage and accelerated "starvation metabolism." Frequent feeding helps preserve muscle mass and supports metabolic stability, which is particularly important when pursuing weight loss in MASLD .

**4. Specific Food Recommendations and Restrictions**

**Encourage:**

| **Food Group** | **Examples** | **Rationale** |
| --- | --- | --- |
| **Plant proteins** | Lentils, chickpeas, tofu, tempeh, edamame | Associated with improved insulin sensitivity, lower liver fat, and reduced mortality in cirrhosis  txliver.com |
| **Fatty fish** | Salmon, mackerel, sardines (cooked) | Omega-3 fatty acids reduce hepatic inflammation and triglycerides; high-quality protein |
| **Non-starchy vegetables** | Leafy greens, broccoli, peppers, tomatoes | Low energy density, high fiber and antioxidants; support weight management and metabolic health |
| **Whole grains** | Oats, quinoa, brown rice, whole-wheat bread | Fiber improves glycemic control, gut health, and satiety during weight loss |
| **Healthy fats** | Extra-virgin olive oil, avocado, nuts, seeds | Monounsaturated fats improve lipid profiles and reduce inflammation; support nutrient absorption |
| **Coffee** | 2-3 cups/day filtered (if tolerated) | Associated with reduced fibrosis progression and lower HCC risk in chronic liver disease |

**Limit or Avoid:**

| **Item** | **Reason** |
| --- | --- |
| **All alcohol** | Accelerates fibrosis, increases HCC risk, worsens outcomes in MASLD cirrhosis |
| **Sugar-sweetened beverages & added sugars** | Fructose promotes hepatic de novo lipogenesis and worsens MASLD |
| **Refined carbohydrates** | White bread, pastries, sugary cereals worsen insulin resistance and promote hepatic fat accumulation |
| **Processed meats & excessive red meat** | Associated with higher inflammation, insulin resistance, and mortality in liver disease |
| **Saturated and trans fats** | Found in fried foods, baked goods; worsen dyslipidemia and hepatic steatosis |
| **Raw/undercooked animal products** | Risk of severe infection (e.g., Vibrio, Salmonella) in immunocompromised liver patients |
| **Excess sodium** | While strict restriction not needed without ascites, aim for <2,300 mg/day to prevent future fluid retention |
| **Hepatotoxic supplements** | Avoid kava, comfrey, high-dose green tea extract, or unregulated herbal products |
| **Unpasteurized dairy/juices** | Risk of bacterial contamination |

**5. Additional Nutritional Considerations**

**Micronutrient Monitoring & Supplementation**

| **Nutrient** | **Rationale** | **Recommendation** |
| --- | --- | --- |
| **Vitamin D** | Deficiency common in chronic liver disease and obesity; associated with worse outcomes and insulin resistance | Screen annually; supplement if <20 ng/mL to reach >30 ng/mL |
| **Zinc** | Deficiency may contribute to dysgeusia, impaired immunity, and poor wound healing; common in liver disease | Consider testing if taste changes occur; supplement if deficient |
| **B-vitamins** | Malabsorption possible in chronic liver disease; important for energy metabolism | Screen annually; supplement if deficient |
| **Iron status** | Monitor ferritin and transferrin saturation; avoid iron supplementation unless deficiency confirmed, as excess iron may worsen liver injury | Check baseline; recheck if clinically indicated |

**Physical Activity & Body Composition**

- **Combine resistance training** (2-3x/week) with **moderate aerobic activity** (150 min/week) to preserve lean mass during weight loss, improve insulin sensitivity, and reduce hepatic fat .
- Focus on **body composition over scale weight**: Prioritize preservation of muscle mass; unintentional muscle loss worsens prognosis in cirrhosis

.

- Given BMI 30.0, **gradual weight normalization** (0.25-0.5 kg/week) is appropriate if accompanied by muscle preservation and improved metabolic parameters

.

**Monitoring & Follow-up**

- **Weight and mid-arm muscle circumference**: Track monthly to distinguish fat loss from muscle loss.
- **Screen for malnutrition/sarcopenia**: Use validated tools (e.g., RFH-NPT, SARC-F, or CT-based muscle assessment if imaging available) annually or with clinical changes
- **Laboratory monitoring**: Albumin, prealbumin, micronutrient panels every 6-12 months in stable compensated disease.
- **Consider dietitian referral**: For personalized meal planning that balances MASLD metabolic goals with cirrhosis nutritional needs and weight management.

**MASLD-Specific Considerations**

- **Address insulin resistance**: Dietary pattern should support glycemic control; coordinate with endocrinology if prediabetes/diabetes present.
- **Coffee consumption**: 2-3 cups/day of filtered coffee is associated with reduced fibrosis progression and may be encouraged if tolerated
- **Avoid unnecessary medications/supplements**: Minimize hepatic metabolic burden; review all medications and supplements with hepatology team.
- **Weight loss monitoring**: If weight loss exceeds 0.5 kg/week or muscle mass declines, reassess caloric intake and protein distribution.

**Sample Daily Meal Pattern (≈1,900 kcal, 95g protein)**

| **Time** | **Meal** | **Example** |
| --- | --- | --- |
| 7:30 AM | Breakfast | Greek yogurt with berries, chia seeds, walnuts, and a boiled egg |
| 10:00 AM | Snack | Apple with 1 tbsp almond butter |
| 12:30 PM | Lunch | Large salad with grilled chicken, chickpeas, mixed vegetables, olive oil-lemon dressing, small quinoa portion |
| 3:30 PM | Snack | Cottage cheese with cucumber slices |
| 6:30 PM | Dinner | Baked salmon, roasted vegetables (broccoli, zucchini, peppers) with olive oil, small sweet potato |
| 9:00 PM | Evening snack | Small casein protein shake or warm milk with cinnamon |

*Note: Adjust portion sizes based on hunger/fullness cues and weight trajectory; prioritize protein at each meal. Monitor weight weekly; target 0.25-0.5 kg loss/week.*

**Important**: These recommendations apply to **compensated** MASLD-related cirrhosis. If the patient develops ascites, hepatic encephalopathy, or other decompensation, dietary management should be promptly re-evaluated by a hepatology team and registered dietitian specializing in liver disease. **Weight loss should never compromise protein intake or muscle preservation** in cirrhosis

.

## C1-06

**Dietary and Nutritional Recommendations for Compensated Alcohol-Related Cirrhosis**

**Patient Profile:** Female, 51 years old, 162 cm, 60 kg (BMI 22.9 kg/m², normal weight), alcohol-related compensated cirrhosis, clinically stable.

**1. Overall Dietary Principles**

**Complete alcohol abstinence**: Absolute avoidance of alcohol is the single most critical intervention to halt disease progression, reduce mortality, and allow potential hepatic recovery .

**Adequate energy intake to maintain weight**: Target **30-35 kcal/kg/day** (approximately 1,800-2,100 kcal/day) to maintain current healthy weight and preserve lean muscle mass. Unlike patients with overweight, weight loss is NOT indicated .

**No protein restriction**: Maintain high-quality protein intake to combat the high risk of sarcopenia and protein-energy malnutrition characteristic of alcohol-related liver disease, even in patients with normal BMI .

**Prioritize nutrient-dense, whole foods**: Focus on foods rich in vitamins, minerals, antioxidants, and fiber to address common micronutrient deficiencies associated with chronic alcohol use and support overall liver health.

**Food safety**: Avoid raw/undercooked seafood, meat, eggs, and unpasteurized products due to increased infection risk in chronic liver disease .

**Multidisciplinary support**: Integrate nutritional counseling with addiction medicine, mental health support, and hepatology follow-up for optimal long-term outcomes.

**2. Recommended Daily Protein Intake**

| **Parameter** | **Recommendation** |
| --- | --- |
| **Target protein intake** | **1.2–1.5 g/kg ideal body weight/day** |
| **For this patient** | **72–90 g protein/day** (based on current weight 60 kg, which approximates ideal body weight) |
| **Protein distribution** | Spread evenly across meals (20-30 g/meal); include late-evening protein snack |
| **Preferred sources** | Plant-based proteins (legumes, tofu, tempeh), fish, poultry, eggs, low-fat dairy; limit red/processed meats |

**Alcohol-related cirrhosis note**: Women with alcohol-related liver disease are particularly vulnerable to malnutrition and muscle loss. Adequate protein intake is essential for muscle preservation, immune function, and wound healing .

**3. Meal Frequency and Eating Pattern**

**Avoid prolonged fasting**: Limit fasting intervals to **≤3-4 hours during waking hours** to prevent accelerated catabolism, which is heightened in alcohol-related liver disease .

**Recommended meal pattern**:

- **3 balanced main meals + 2-3 snacks** daily
- **Include a late-evening snack** (e.g., Greek yogurt, cottage cheese, or small protein shake) to reduce overnight muscle breakdown
- **Early breakfast** within 1-2 hours of waking to break the overnight fast

**Rationale**: Alcohol-related cirrhosis is associated with profound metabolic derangements including accelerated starvation metabolism, impaired glycogen storage, and increased protein catabolism. Frequent feeding helps maintain nitrogen balance and muscle mass, which is critical even in patients with normal BMI .

**4. Specific Food Recommendations and Restrictions**

**Encourage:**

| **Food Group** | **Examples** | **Rationale** |
| --- | --- | --- |
| **Plant proteins** | Lentils, chickpeas, tofu, tempeh, edamame | Well-tolerated, associated with lower mortality in cirrhosis; support gut health |
| **Lean animal proteins** | Fish, poultry, eggs, low-fat dairy | High-quality protein with complete amino acid profile to support muscle synthesis |
| **Whole grains** | Oats, quinoa, brown rice, whole-wheat bread | Fiber supports gut health and steady energy release; rich in B-vitamins |
| **Colorful fruits & vegetables** | Berries, leafy greens, cruciferous vegetables, citrus | Antioxidants combat oxidative stress from prior alcohol exposure; support immune function |
| **Healthy fats** | Olive oil, avocado, nuts, seeds | Support calorie density and nutrient absorption without excess saturated fat |
| **Thiamine-rich foods** | Whole grains, legumes, pork, fortified cereals, sunflower seeds | Address high risk of thiamine deficiency in alcohol-related liver disease |

**Limit or Avoid:**

| **Item** | **Reason** |
| --- | --- |
| **All alcohol** | Even small amounts accelerate fibrosis, increase HCC risk, and worsen outcomes; abstinence is non-negotiable |
| **Raw/undercooked seafood, meat, eggs** | Risk of severe infection (e.g., Vibrio, Salmonella, Listeria) in immunocompromised liver patients |
| **Unpasteurized dairy/juices** | Risk of bacterial contamination |
| **Excess sodium** | While strict restriction not needed without ascites, aim for <2,300 mg/day to prevent future fluid retention |
| **High-dose herbal supplements** | Many herbs (kava, comfrey, high-dose green tea extract) are hepatotoxic; avoid unless approved by hepatology |
| **Processed meats & excessive red meat** | Associated with higher inflammation and mortality in liver disease |
| **Excess added sugars** | May contribute to hepatic fat accumulation and metabolic dysfunction, even in non-MASLD cirrhosis |

**5. Additional Nutritional Considerations**

**Critical Micronutrient Screening & Supplementation**

| **Nutrient** | **Rationale** | **Recommendation** |
| --- | --- | --- |
| **Thiamine (B1)** | Alcohol impairs absorption, storage, and activation; deficiency can cause Wernicke-Korsakoff syndrome | **Supplement prophylactically**: 100 mg/day oral thiamine; IV if malnourished, acute illness, or withdrawal |
| **Folate (B9)** | Alcohol reduces intestinal absorption and increases renal excretion | Screen levels; supplement 1 mg/day if deficient or dietary intake inadequate |
| **Vitamin B12** | Malabsorption common in chronic liver disease and with gastric changes from alcohol | Screen annually; supplement if deficient |
| **Vitamin D** | Deficiency prevalent in chronic liver disease; associated with worse outcomes, bone loss, immune dysfunction | Screen annually; supplement to maintain >30 ng/mL |
| **Zinc** | Deficiency contributes to dysgeusia, impaired immunity, poor wound healing, and may worsen encephalopathy risk | Consider supplementation (25-50 mg elemental zinc/day) if deficient or symptomatic |
| **Magnesium** | Alcohol increases renal excretion; deficiency common and may contribute to muscle cramps, arrhythmias | Screen if symptoms present; supplement if deficient |

**Physical Activity & Muscle Preservation**

- **Prioritize resistance training** (2-3x/week) combined with moderate aerobic activity (e.g., walking 30 min/day) to combat sarcopenia and improve insulin sensitivity .
- Focus on **preserving lean mass** rather than weight change; even in normal-weight patients, muscle loss worsens prognosis in cirrhosis.
- Given normal BMI (22.9), the goal is **weight maintenance with body composition optimization**—preserving or building muscle while avoiding fat gain.

**Monitoring & Follow-up**

- **Weight and mid-arm muscle circumference**: Track monthly to detect early muscle loss before weight changes occur.
- **Screen for malnutrition/sarcopenia**: Use validated tools (e.g., RFH-NPT, SARC-F, handgrip strength) at each visit .
- **Laboratory monitoring**: Albumin, prealbumin, complete metabolic panel, and micronutrient panels every 3-6 months.
- **Multidisciplinary referral**: Strongly consider referral to registered dietitian specializing in liver disease, addiction specialist, and mental health support.

**Alcohol-Related Cirrhosis–Specific Considerations**

- **Thiamine before glucose**: If patient experiences acute illness, surgery, or requires IV fluids, administer thiamine before dextrose to prevent precipitating Wernicke's encephalopathy .
- **Withdrawal support**: Ensure patient has access to medical support for alcohol withdrawal if needed; nutritional support is adjunctive, not primary treatment.
- **Relapse prevention**: Integrate nutritional counseling with behavioral strategies; poor nutrition, hunger, or micronutrient deficiencies can trigger cravings and relapse.
- **Bone health**: Women with alcohol-related cirrhosis are at increased risk for osteoporosis; ensure adequate calcium (1,000-1,200 mg/day) and vitamin D; consider bone density screening.

**Sample Daily Meal Pattern (≈1,900 kcal, 80g protein)**

| **Time** | **Meal** | **Example** |
| --- | --- | --- |
| 7:30 AM | Breakfast | Scrambled eggs with spinach and tomatoes, whole-wheat toast, orange |
| 10:00 AM | Snack | Greek yogurt with berries and a sprinkle of walnuts |
| 12:30 PM | Lunch | Grilled chicken salad with mixed greens, chickpeas, avocado, olive oil-lemon dressing, small quinoa portion |
| 3:30 PM | Snack | Hummus with carrot/cucumber sticks and whole-grain crackers |
| 6:30 PM | Dinner | Baked cod, sweet potato, steamed green beans with olive oil and garlic |
| 9:00 PM | Evening snack | Cottage cheese or small casein protein shake |

*Note: Thiamine supplement (100 mg) to be taken daily with food. Adjust portion sizes based on hunger/fullness cues and weight stability.*

**Important**: These recommendations apply to **compensated** alcohol-related cirrhosis. If the patient develops ascites, hepatic encephalopathy, acute alcoholic hepatitis, or other decompensation, dietary management must be promptly re-evaluated by a hepatology team and registered dietitian specializing in liver disease. **Alcohol abstinence remains the cornerstone of management**—nutritional support is essential but adjunctive.

## C1-07

**Dietary and Nutritional Recommendations for Compensated HBV-Related Cirrhosis**

**Patient Profile:** Male, 55 years old, 170 cm, 64 kg (BMI 22.1 kg/m², normal weight), HBV-related compensated cirrhosis, clinically stable.

**1. Overall Dietary Principles**

**Adequate energy intake to maintain weight**: Target **25-35 kcal/kg/day** (approximately 1,600-2,200 kcal/day) to maintain current healthy weight and preserve lean muscle mass. Weight loss or gain is NOT indicated given normal BMI .

**No protein restriction**: Maintain high-quality protein intake to prevent sarcopenia, which is strongly associated with increased mortality, hospitalization, and complications in cirrhosis regardless of BMI .

**Adopt a Mediterranean-style dietary pattern**: Emphasize vegetables, fruits, whole grains, legumes, nuts, olive oil, and fish while limiting red/processed meats and refined carbohydrates. This pattern supports liver health, reduces inflammation, and may modestly reduce hepatocellular carcinoma (HCC) risk .

**Alcohol abstinence**: Complete avoidance of alcohol is mandatory in HBV-related cirrhosis to prevent accelerated fibrosis progression and reduce HCC risk.

**Food safety**: Avoid raw/undercooked seafood, meat, eggs, and unpasteurized products due to increased infection risk in chronic liver disease .

**Antiviral therapy adherence**: Nutritional support complements but does not replace medical management; ensure ongoing adherence to HBV antiviral therapy per hepatology guidance.

**2. Recommended Daily Protein Intake**

| **Parameter** | **Recommendation** |
| --- | --- |
| **Target protein intake** | **1.2–1.5 g/kg ideal body weight/day** |
| **For this patient** | **77–96 g protein/day** (based on current weight 64 kg, which approximates ideal body weight) |
| **Protein distribution** | Spread evenly across meals (20-30 g/meal); include late-evening protein snack |
| **Preferred sources** | Plant-based proteins (legumes, tofu, tempeh), fish, poultry, eggs, low-fat dairy; limit red/processed meats |

**Key Point**: Higher intake of plant-based and dairy protein is associated with reduced mortality in cirrhosis. Adequate protein is essential to preserve muscle mass, particularly important at age 55 when sarcopenia risk increases .

**3. Meal Frequency and Eating Pattern**

**Avoid prolonged fasting**: Limit fasting intervals to **≤3-4 hours during waking hours** to prevent accelerated catabolism and maintain nitrogen balance .

**Recommended meal pattern**:

- **3 balanced main meals + 2-3 snacks** daily
- **Include a late-evening snack** (e.g., Greek yogurt, cottage cheese, or small protein shake) to reduce overnight muscle breakdown
- **Early breakfast** within 1-2 hours of waking to break the overnight fast

**Rationale**: Patients with cirrhosis have impaired glycogen storage and accelerated "starvation metabolism." Frequent feeding helps preserve muscle mass and supports metabolic stability, which is particularly important for preventing age-related sarcopenia .

**4. Specific Food Recommendations and Restrictions**

**Encourage:**

| **Food Group** | **Examples** | **Rationale** |
| --- | --- | --- |
| **Plant proteins** | Lentils, chickpeas, tofu, tempeh, edamame | Associated with lower mortality in cirrhosis; well-tolerated; support gut health |
| **Fatty fish** | Salmon, mackerel, sardines (cooked) | Omega-3 fatty acids reduce hepatic inflammation; high-quality protein |
| **Whole grains** | Oats, quinoa, brown rice, whole-wheat bread | Fiber improves glycemic control and gut health; rich in B-vitamins important for liver metabolism |
| **Colorful fruits & vegetables** | Berries, leafy greens, cruciferous vegetables, citrus | Antioxidants combat oxidative stress; support immune function and overall health |
| **Healthy fats** | Extra-virgin olive oil, avocado, nuts, seeds | Monounsaturated fats improve lipid profiles; support nutrient absorption without excess saturated fat |
| **Coffee** | 2-3 cups/day filtered (if tolerated) | Associated with reduced fibrosis progression and lower HCC risk in chronic liver disease |

**Limit or Avoid:**

| **Item** | **Reason** |
| --- | --- |
| **All alcohol** | Accelerates fibrosis, increases HCC risk, worsens outcomes in HBV cirrhosis |
| **Raw/undercooked seafood, meat, eggs** | Risk of severe infection (e.g., Vibrio, Salmonella, Listeria) in immunocompromised liver patients |
| **Excess sodium** | While strict restriction not needed without ascites, aim for <2,300 mg/day to prevent future fluid retention |
| **Processed meats & excessive red meat** | Associated with higher inflammation and mortality in liver disease |
| **Added sugars & refined carbohydrates** | May promote hepatic fat accumulation and worsen metabolic health |
| **Hepatotoxic supplements** | Avoid kava, comfrey, high-dose green tea extract, or unregulated herbal products |
| **Unpasteurized dairy/juices** | Risk of bacterial contamination |
| **Iron supplements** | Avoid unless deficiency confirmed; HBV cirrhosis may be associated with elevated iron stores |

**5. Additional Nutritional Considerations**

**Micronutrient Monitoring & Supplementation**

| **Nutrient** | **Rationale** | **Recommendation** |
| --- | --- | --- |
| **Vitamin D** | Deficiency common in chronic liver disease; associated with worse outcomes, bone loss, immune dysfunction | Screen annually; supplement if <20 ng/mL to reach >30 ng/mL |
| **Zinc** | Deficiency may contribute to dysgeusia, impaired immunity, poor wound healing; common in liver disease | Consider testing if taste changes occur; supplement if deficient |
| **B-vitamins (B12, folate)** | Malabsorption possible in chronic liver disease; important for energy metabolism and homocysteine regulation | Screen annually; supplement if deficient |
| **Calcium** | Age 55 + chronic liver disease increases osteoporosis risk | Ensure intake 1,000-1,200 mg/day through diet or supplementation |

**Physical Activity & Sarcopenia Prevention**

- **Combine resistance training** (2-3x/week) with **moderate aerobic activity** (150 min/week) to preserve lean mass, improve insulin sensitivity, and reduce fall risk .
- Focus on **body composition over scale weight**: At age 55, prioritize preservation of muscle mass; unintentional muscle loss worsens prognosis in cirrhosis .
- Given normal BMI (22.1), the goal is **weight maintenance with body composition optimization**—preserving or building muscle while avoiding fat gain.

**Monitoring & Follow-up**

- **Weight and mid-arm muscle circumference**: Track monthly to detect early muscle loss before weight changes occur.
- **Screen for sarcopenia**: Use validated tools (e.g., SARC-F, handgrip strength, or imaging-based muscle assessment if available) annually .
- **Laboratory monitoring**: Albumin, prealbumin, complete metabolic panel, and micronutrient panels every 6-12 months in stable compensated disease.
- **HCC surveillance**: Maintain recommended imaging (ultrasound ± AFP) every 6 months per guidelines; healthy dietary patterns may modestly support risk reduction .
- **Consider dietitian referral**: For personalized meal planning and ongoing nutritional support.

**HBV-Specific Considerations**

- **Antiviral therapy adherence**: Ensure ongoing adherence to HBV antiviral therapy; nutrition supports but does not replace medical treatment.
- **Avoid unnecessary medications/supplements**: Minimize hepatic metabolic burden; review all medications and supplements with hepatology team.
- **Vaccinations**: Ensure hepatitis A vaccination if not immune; discuss influenza and pneumococcal vaccines with primary care.

**Sample Daily Meal Pattern (≈1,900 kcal, 85g protein)**

| **Time** | **Meal** | **Example** |
| --- | --- | --- |
| 7:30 AM | Breakfast | Oatmeal with Greek yogurt, berries, walnuts, and a boiled egg |
| 10:00 AM | Snack | Apple with 1 tbsp almond butter |
| 12:30 PM | Lunch | Grilled chicken salad with chickpeas, mixed vegetables, olive oil-lemon dressing, small quinoa portion |
| 3:30 PM | Snack | Cottage cheese with cucumber slices |
| 6:30 PM | Dinner | Baked salmon, brown rice, steamed broccoli and carrots with olive oil |
| 9:00 PM | Evening snack | Small casein protein shake or warm milk with cinnamon |

*Note: Adjust portion sizes based on hunger/fullness cues and weight stability; prioritize protein at each meal.*

**Important**: These recommendations apply to **compensated** HBV-related cirrhosis. If the patient develops ascites, hepatic encephalopathy, or other decompensation, dietary management should be promptly re-evaluated by a hepatology team and registered dietitian specializing in liver disease. **Maintaining muscle mass is critical**—even in patients with normal BMI, sarcopenia significantly worsens outcomes in cirrhosis .

## C1-08

**Dietary and Nutritional Recommendations for Compensated MASLD-Related Cirrhosis with Non-Bleeding Esophageal Varices**

**Patient Profile:** Female, 58 years old, 156 cm, 78 kg (BMI 32.0 kg/m², obese class I), MASLD-related compensated cirrhosis with non-bleeding esophageal varices, clinically stable.

**1. Overall Dietary Principles**

**Adopt a Mediterranean-style dietary pattern**: Emphasize vegetables, fruits, whole grains, legumes, nuts, olive oil, and fish while limiting red/processed meats and refined carbohydrates. This pattern supports liver health, improves insulin sensitivity, and may reduce fibrosis progression in MASLD .

**Moderate caloric restriction for gradual weight loss**: Target **25-30 kcal/kg ideal body weight/day** (approximately 1,300-1,600 kcal/day using ideal body weight ~52 kg) to support sustainable weight loss of **0.25-0.5 kg/week**. Aim for **7-10% total body weight loss** (~5-8 kg) over 6-12 months, which is associated with improved liver histology in MASLD, provided muscle mass is preserved .

**No protein restriction**: Maintain high-quality protein intake (1.2-1.5 g/kg IBW/day) to prevent sarcopenia, a strong independent predictor of mortality in cirrhosis regardless of BMI .

**Variceal-safe food texture**: While evidence for strict dietary texture modification in non-bleeding varices is limited, prudent precautions include chewing food thoroughly, avoiding extremely hard/sharp foods, and ensuring adequate hydration with meals to facilitate safe swallowing .

**Complete alcohol abstinence**: Essential in MASLD-related cirrhosis to prevent accelerated fibrosis and reduce hepatocellular carcinoma risk.

**Food safety**: Avoid raw/undercooked seafood, meat, eggs, and unpasteurized products due to increased infection risk in chronic liver disease .

**2. Recommended Daily Protein Intake**

| **Parameter** | **Recommendation** |
| --- | --- |
| **Target protein intake** | **1.2–1.5 g/kg ideal body weight/day** |
| **For this patient** | **62–78 g protein/day** (based on ideal body weight ~52 kg for height 156 cm) |
| **Protein distribution** | Spread evenly across meals (20-25 g/meal); include late-evening protein snack |
| **Preferred sources** | Plant-based proteins (legumes, tofu, tempeh), fish, poultry, eggs, low-fat dairy; limit red/processed meats |

**Key Point**: In patients with cirrhosis and obesity, higher protein intake during weight loss helps preserve lean mass while promoting fat loss. Plant-based proteins are particularly beneficial for MASLD due to favorable effects on insulin sensitivity and lower inflammatory burden .

**3. Meal Frequency and Eating Pattern**

**Avoid prolonged fasting**: Limit fasting intervals to **≤3-4 hours during waking hours** to prevent accelerated catabolism and maintain nitrogen balance .

**Recommended meal pattern**:

- **3 balanced main meals + 2-3 snacks** daily
- **Include a late-evening snack** (e.g., Greek yogurt, cottage cheese, or small protein shake) to reduce overnight muscle breakdown
- **Early breakfast** within 1-2 hours of waking to break the overnight fast
- **Chew food thoroughly** and avoid rushing meals to minimize mechanical stress on esophageal varices

**Rationale**: Patients with cirrhosis have impaired glycogen storage and accelerated "starvation metabolism." Frequent feeding helps preserve muscle mass and supports metabolic stability, which is particularly important when pursuing weight loss in MASLD while managing variceal risk .

**4. Specific Food Recommendations and Restrictions**

**Encourage:**

| **Food Group** | **Examples** | **Rationale** |
| --- | --- | --- |
| **Soft plant proteins** | Lentil soup, hummus, tofu, tempeh, well-cooked beans | Well-tolerated texture; associated with lower mortality and improved insulin sensitivity |
| **Fatty fish** | Salmon, mackerel, sardines (well-cooked, flaked) | Omega-3 fatty acids reduce hepatic inflammation; easy-to-chew protein source |
| **Soft-cooked vegetables** | Steamed carrots, zucchini, spinach, well-cooked broccoli | Nutrient-dense, low energy density; cooking softens fiber for easier swallowing |
| **Whole grains (soft preparations)** | Oatmeal, soft-cooked quinoa, whole-wheat pasta (well-cooked) | Fiber improves glycemic control; soft textures reduce mechanical irritation risk |
| **Healthy fats** | Extra-virgin olive oil (as dressing), avocado (mashed), nut butters | Monounsaturated fats improve lipid profiles; easy to incorporate into soft foods |
| **Coffee** | 2-3 cups/day filtered (moderate temperature, if tolerated) | Associated with reduced fibrosis progression and lower HCC risk |

**Limit or Avoid:**

| **Item** | **Reason** |
| --- | --- |
| **All alcohol** | Accelerates fibrosis, increases HCC risk, worsens outcomes in MASLD cirrhosis |
| **Very hard, sharp, or rough-textured foods** | Raw carrots, hard nuts, crusty bread, popcorn, chips: theoretical risk of mechanical trauma to varices (prudent precaution) |
| **Very hot beverages/foods** | May cause mucosal irritation; allow to cool to moderate temperature before consuming |
| **Sugar-sweetened beverages & added sugars** | Fructose promotes hepatic de novo lipogenesis and worsens MASLD |
| **Refined carbohydrates** | White bread, pastries, sugary cereals worsen insulin resistance |
| **Processed meats & excessive red meat** | Associated with higher inflammation and mortality in liver disease |
| **Large, hard-to-swallow pills** | Consider liquid formulations or crushing (if medically appropriate) to minimize esophageal irritation; consult pharmacist |
| **Excess sodium** | Aim for <2,300 mg/day to prevent future fluid retention, especially with portal hypertension |
| **Raw/undercooked animal products** | Risk of severe infection in immunocompromised liver patients |
| **Hepatotoxic supplements** | Avoid kava, comfrey, high-dose green tea extract, or unregulated herbal products |

**Variceal Note**: Current guidelines do not mandate strict dietary texture modification for non-bleeding varices, but prudent precautions (thorough chewing, avoiding extremely hard foods) are reasonable and low-risk . Endoscopic variceal ligation and non-selective beta-blockers remain primary prevention strategies.

**5. Additional Nutritional Considerations**

**Micronutrient Monitoring & Supplementation**

| **Nutrient** | **Rationale** | **Recommendation** |
| --- | --- | --- |
| **Vitamin D** | Deficiency common in chronic liver disease and obesity; associated with worse outcomes, bone loss, immune dysfunction | Screen annually; supplement if <20 ng/mL to reach >30 ng/mL |
| **Calcium** | Post-menopausal woman + chronic liver disease increases osteoporosis risk | Ensure intake 1,200 mg/day through diet (dairy, fortified foods) or supplementation |
| **Zinc** | Deficiency may contribute to dysgeusia, impaired immunity; common in liver disease | Consider testing if taste changes occur; supplement if deficient |
| **B-vitamins** | Malabsorption possible in chronic liver disease; important for energy metabolism | Screen annually; supplement if deficient |
| **Iron status** | Monitor ferritin and transferrin saturation; avoid iron supplementation unless deficiency confirmed | Check baseline; recheck if clinically indicated |

**Physical Activity & Body Composition**

- **Combine resistance training** (2-3x/week, low-impact if needed) with **moderate aerobic activity** (150 min/week walking, cycling) to preserve lean mass during weight loss, improve insulin sensitivity, and reduce hepatic fat .
- **Avoid heavy straining/Valsalva maneuvers** during exercise (e.g., heavy weightlifting) which could theoretically increase portal pressure; focus on controlled movements .
- Focus on **body composition over scale weight**: Prioritize preservation of muscle mass; unintentional muscle loss worsens prognosis in cirrhosis .
- Given BMI 32.0, **gradual weight normalization** (0.25-0.5 kg/week) is appropriate if accompanied by muscle preservation and improved metabolic parameters .

**Monitoring & Follow-up**

- **Weight and mid-arm muscle circumference**: Track monthly to distinguish fat loss from muscle loss.
- **Screen for sarcopenia**: Use validated tools (e.g., SARC-F, handgrip strength) annually .
- **Variceal surveillance**: Continue endoscopic surveillance per hepatology guidance; dietary precautions are adjunctive, not primary prevention.
- **Laboratory monitoring**: Albumin, prealbumin, complete metabolic panel, and micronutrient panels every 6-12 months in stable compensated disease.
- **Consider dietitian referral**: For personalized meal planning that balances MASLD metabolic goals, cirrhosis nutritional needs, weight management, and variceal precautions.

**MASLD & Variceal-Specific Considerations**

- **Address insulin resistance**: Dietary pattern should support glycemic control; coordinate with endocrinology if prediabetes/diabetes present.
- **Coffee consumption**: 2-3 cups/day of filtered coffee (moderate temperature) is associated with reduced fibrosis progression and may be encouraged if tolerated .
- **Weight loss monitoring**: If weight loss exceeds 0.5 kg/week or muscle mass declines, reassess caloric intake and protein distribution.
- **Medication review**: Ensure all medications (including beta-blockers for variceal prophylaxis) are reviewed for potential nutritional interactions.

**Sample Daily Meal Pattern (≈1,450 kcal, 70g protein)**

| **Time** | **Meal** | **Example** |
| --- | --- | --- |
| 7:30 AM | Breakfast | Soft oatmeal with Greek yogurt, mashed berries, chia seeds, and a soft-boiled egg |
| 10:00 AM | Snack | Smoothie: banana, protein powder, spinach, almond milk (blended smooth) |
| 12:30 PM | Lunch | Soft lentil soup, well-cooked quinoa, steamed zucchini and carrots with olive oil |
| 3:30 PM | Snack | Cottage cheese with soft peach slices (peeled) |
| 6:30 PM | Dinner | Flaked baked salmon, soft-cooked brown rice, mashed avocado, steamed green beans |
| 9:00 PM | Evening snack | Small casein protein shake or warm milk with cinnamon |

*Note: All foods prepared with soft textures; chew thoroughly. Adjust portion sizes based on hunger/fullness cues and weight trajectory; target 0.25-0.5 kg loss/week. Thiamine and multivitamin supplementation may be considered per provider.*

**Important**: These recommendations apply to **compensated** MASLD-related cirrhosis with **non-bleeding** esophageal varices. If the patient develops variceal bleeding, ascites, hepatic encephalopathy, or other decompensation, dietary management must be promptly re-evaluated by a hepatology team and registered dietitian specializing in liver disease. **Weight loss should never compromise protein intake or muscle preservation** in cirrhosis . Dietary texture precautions are prudent but adjunctive; endoscopic and pharmacologic prophylaxis remain primary strategies for variceal management .

## C1-09

**Dietary and Nutritional Recommendations for Compensated Alcohol-Related Cirrhosis in an Older Adult**

**Patient Profile:** Male, 61 years old, 167 cm, 58 kg (BMI 20.8 kg/m², normal weight at lower end), alcohol-related compensated cirrhosis, clinically stable.

**1. Overall Dietary Principles**

**Complete alcohol abstinence**: Absolute avoidance of alcohol is the single most critical intervention to halt disease progression, reduce mortality, and allow potential hepatic recovery

.

**Adequate-to-high energy intake to prevent catabolism**: Target **30-35 kcal/kg/day** (approximately 1,740-2,030 kcal/day) to maintain current weight and preserve lean muscle mass. Given age 61 and alcohol-related malnutrition risk, the higher end of the range is preferred.

**No protein restriction—prioritize adequacy**: Maintain high-quality protein intake (1.2-1.5 g/kg/day) to combat the high risk of sarcopenia and protein-energy malnutrition characteristic of alcohol-related liver disease, even in patients with normal BMI

**Prioritize nutrient-dense, whole foods**: Focus on foods rich in vitamins, minerals, antioxidants, and fiber to address common micronutrient deficiencies associated with chronic alcohol use and support overall liver health.

**Food safety**: Avoid raw/undercooked seafood, meat, eggs, and unpasteurized products due to increased infection risk in chronic liver disease .

**Multidisciplinary support**: Integrate nutritional counseling with addiction medicine, geriatric assessment, mental health support, and hepatology follow-up for optimal long-term outcomes.

**2. Recommended Daily Protein Intake**

| **Parameter** | **Recommendation** |
| --- | --- |
| **Target protein intake** | **1.2–1.5 g/kg ideal body weight/day** |
| **For this patient** | **70–87 g protein/day** (based on current weight 58 kg, which approximates ideal body weight) |
| **Protein distribution** | Spread evenly across meals (20-30 g/meal); include late-evening protein snack |
| **Preferred sources** | Plant-based proteins (legumes, tofu, tempeh), fish, poultry, eggs, low-fat dairy; limit red/processed meats |

**Age + alcohol-related cirrhosis note**: Older adults with alcohol-related liver disease have particularly high rates of sarcopenia and protein-energy malnutrition. Adequate protein intake is essential for muscle preservation, immune function, wound healing, and functional independence. Insufficient protein intake is associated with increased mortality in cirrhosis .

**3. Meal Frequency and Eating Pattern**

**Avoid prolonged fasting**: Limit fasting intervals to **≤3-4 hours during waking hours** to prevent accelerated catabolism, which is heightened in alcohol-related liver disease and aging

.

**Recommended meal pattern**:

- **3 balanced main meals + 2-3 snacks** daily
- **Include a late-evening snack** (e.g., Greek yogurt, cottage cheese, or small protein shake) to reduce overnight muscle breakdown
- **Early breakfast** within 1-2 hours of waking to break the overnight fast

**Rationale**: Alcohol-related cirrhosis is associated with profound metabolic derangements including accelerated starvation metabolism, impaired glycogen storage, and increased protein catabolism. Aging further amplifies sarcopenia risk. Frequent feeding helps maintain nitrogen balance and muscle mass, which is critical even in patients with normal BMI.

**4. Specific Food Recommendations and Restrictions**

**Encourage:**

| **Food Group** | **Examples** | **Rationale** |
| --- | --- | --- |
| **Plant proteins** | Lentils, chickpeas, tofu, tempeh, edamame | Well-tolerated, associated with lower mortality in cirrhosis; support gut health |
| **Lean animal proteins** | Fish, poultry, eggs, low-fat dairy | High-quality protein with complete amino acid profile to support muscle synthesis |
| **Whole grains** | Oats, quinoa, brown rice, whole-wheat bread | Fiber supports gut health and steady energy release; rich in B-vitamins |
| **Colorful fruits & vegetables** | Berries, leafy greens, cruciferous vegetables, citrus | Antioxidants combat oxidative stress from prior alcohol exposure; support immune function |
| **Healthy fats** | Olive oil, avocado, nuts, seeds | Support calorie density and nutrient absorption without excess saturated fat |
| **Thiamine-rich foods** | Whole grains, legumes, pork, fortified cereals, sunflower seeds | Address high risk of thiamine deficiency in alcohol-related liver disease |

**Limit or Avoid:**

| **Item** | **Reason** |
| --- | --- |
| **All alcohol** | Even small amounts accelerate fibrosis, increase HCC risk, and worsen outcomes; abstinence is non-negotiable |
| **Raw/undercooked seafood, meat, eggs** | Risk of severe infection (e.g., Vibrio, Salmonella, Listeria) in immunocompromised liver patients |
| **Unpasteurized dairy/juices** | Risk of bacterial contamination |
| **Excess sodium** | While strict restriction not needed without ascites, aim for <2,300 mg/day to prevent future fluid retention |
| **High-dose herbal supplements** | Many herbs (kava, comfrey, high-dose green tea extract) are hepatotoxic; avoid unless approved by hepatology |
| **Processed meats & excessive red meat** | Associated with higher inflammation and mortality in liver disease |
| **Excess added sugars** | May contribute to hepatic fat accumulation and metabolic dysfunction |

**5. Additional Nutritional Considerations**

**Critical Micronutrient Screening & Supplementation**

| **Nutrient** | **Rationale** | **Recommendation** |
| --- | --- | --- |
| **Thiamine (B1)** | Alcohol impairs absorption, storage, and activation; deficiency can cause Wernicke-Korsakoff syndrome; risk increases with age | **Supplement prophylactically**: 100 mg/day oral thiamine; IV if malnourished, acute illness, or withdrawal |
| **Folate (B9)** | Alcohol reduces intestinal absorption and increases renal excretion | Screen levels; supplement 1 mg/day if deficient or dietary intake inadequate |
| **Vitamin B12** | Malabsorption common in chronic liver disease and with age-related gastric changes | Screen annually; supplement if deficient |
| **Vitamin D** | Deficiency prevalent in chronic liver disease and aging; associated with worse outcomes, bone loss, immune dysfunction | Screen annually; supplement to maintain >30 ng/mL |
| **Zinc** | Deficiency contributes to dysgeusia, impaired immunity, poor wound healing; common in liver disease | Consider supplementation (25-50 mg elemental zinc/day) if deficient or symptomatic |
| **Magnesium** | Alcohol increases renal excretion; deficiency common and may contribute to muscle cramps, arrhythmias | Screen if symptoms present; supplement if deficient |

**Physical Activity & Sarcopenia Prevention**

- **Prioritize resistance training** (2-3x/week, age-appropriate intensity) combined with moderate aerobic activity (e.g., walking 30 min/day) to combat sarcopenia and improve insulin sensitivity

.

- Focus on **preserving lean mass and functional capacity** rather than weight change; even in normal-weight patients, muscle loss worsens prognosis in cirrhosis

.

- Given BMI 20.8 (lower end of normal) and age 61, the goal is **weight maintenance with body composition optimization**—preserving or building muscle while avoiding fat gain.

**🦴 Bone Health Considerations**

- Men >60 with alcohol-related cirrhosis are at increased risk for osteoporosis due to alcohol effects, vitamin D deficiency, and reduced physical activity.
- Ensure adequate **calcium (1,000-1,200 mg/day)** and **vitamin D**; consider bone density screening (DEXA) if risk factors present.

**Monitoring & Follow-up**

- **Weight and mid-arm muscle circumference**: Track monthly to detect early muscle loss before weight changes occur.
- **Screen for sarcopenia/frailty**: Use validated tools (e.g., SARC-F, handgrip strength, gait speed) at each visit

.

- **Laboratory monitoring**: Albumin, prealbumin, complete metabolic panel, and micronutrient panels every 3-6 months.
- **Multidisciplinary referral**: Strongly consider referral to registered dietitian specializing in liver disease, addiction specialist, geriatrician, and mental health support.

**Alcohol-Related Cirrhosis–Specific Considerations**

- **Thiamine before glucose**: If patient experiences acute illness, surgery, or requires IV fluids, administer thiamine before dextrose to prevent precipitating Wernicke's encephalopathy

.

- **Withdrawal support**: Ensure patient has access to medical support for alcohol withdrawal if needed; nutritional support is adjunctive, not primary treatment.
- **Relapse prevention**: Integrate nutritional counseling with behavioral strategies; poor nutrition, hunger, or micronutrient deficiencies can trigger cravings and relapse.
- **Medication review**: Review all medications for hepatic metabolism burden and potential nutritional interactions.

**Sample Daily Meal Pattern (≈1,900 kcal, 80g protein)**

| **Time** | **Meal** | **Example** |
| --- | --- | --- |
| 7:30 AM | Breakfast | Scrambled eggs with spinach and tomatoes, whole-wheat toast, orange |
| 10:00 AM | Snack | Greek yogurt with berries and a sprinkle of walnuts |
| 12:30 PM | Lunch | Grilled chicken salad with mixed greens, chickpeas, avocado, olive oil-lemon dressing, small quinoa portion |
| 3:30 PM | Snack | Hummus with carrot/cucumber sticks and whole-grain crackers |
| 6:30 PM | Dinner | Baked cod, sweet potato, steamed green beans with olive oil and garlic |
| 9:00 PM | Evening snack | Cottage cheese or small casein protein shake |

*Note: Thiamine supplement (100 mg) to be taken daily with food. Adjust portion sizes based on hunger/fullness cues and weight stability. Ensure adequate hydration throughout the day.*

**Important**: These recommendations apply to **compensated** alcohol-related cirrhosis. If the patient develops ascites, hepatic encephalopathy, acute alcoholic hepatitis, or other decompensation, dietary management must be promptly re-evaluated by a hepatology team and registered dietitian specializing in liver disease. **Alcohol abstinence remains the cornerstone of management**—nutritional support is essential but adjunctive .

## C1-10

**Dietary and Nutritional Recommendations for Compensated HBV-Related Cirrhosis**

**Patient Summary:** 66-year-old female, 155 cm, 50 kg (BMI 20.8 kg/m²), HBV-related compensated cirrhosis

**1. Overall Dietary Principles**

- **Adequate energy intake:** Target 30-35 kcal/kg/day (approximately 1,500-1,750 kcal/day) to prevent catabolism and maintain muscle mass

.

- **Prioritize protein adequacy:** Protein should NOT be restricted in compensated cirrhosis; adequate intake is essential to prevent sarcopenia

.

- **Balanced macronutrients:** Emphasize complex carbohydrates, lean proteins, and healthy fats while limiting simple sugars and saturated fats

.

- **Avoid alcohol completely:** Absolute abstinence is mandatory to prevent further liver injury.
- **Food safety:** Avoid raw or undercooked shellfish (risk of *Vibrio vulnificus* infection) and ensure proper food handling

.

- **Limit added sugars:** High-fructose/sucrose diets promote hepatic lipogenesis and may accelerate fibrosis progression

.

**2. Recommended Daily Protein Intake**

| **Parameter** | **Recommendation** |
| --- | --- |
| **Target protein** | **1.2–1.5 g/kg/day** = **60–75 g/day** for this patient |
| **Distribution** | Spread evenly across meals (20-25 g per meal) to optimize muscle protein synthesis |
| **Protein sources** | Prioritize plant-based proteins (legumes, tofu, lentils) and lean animal proteins (fish, poultry, eggs); vegetable proteins may be better tolerated if hepatic encephalopathy develops |
| **If protein intolerance occurs** | Consider branched-chain amino acid (BCAA) supplementation under medical supervision |

**3. Meal Frequency and Eating Pattern**

- **Small, frequent meals:** 4-6 meals/snacks per day to reduce prolonged fasting periods and minimize muscle catabolism
- **Late-evening snack (LES):** Include a carbohydrate + protein snack between 7-10 PM (e.g., Greek yogurt with fruit, whole-grain crackers with cheese) to improve nitrogen balance and preserve muscle mass .
- **Avoid prolonged fasting:** Do not go >3-4 hours without eating during waking hours.
- **Breakfast importance:** A protein-containing breakfast may support cognitive function and metabolic stability .

**4. Specific Food Recommendations and Restrictions**

**Encourage:**

| **Food Group** | **Examples** | **Rationale** |
| --- | --- | --- |
| **Lean proteins** | Fish (salmon, cod), skinless poultry, eggs, tofu, lentils, Greek yogurt | Supports muscle maintenance; fish provides omega-3 fatty acids with anti-inflammatory benefits |
| **Complex carbohydrates** | Oats, brown rice, quinoa, whole-wheat bread, sweet potatoes | Provides sustained energy; high-fiber options support gut health |
| **Healthy fats** | Olive oil, avocado, nuts (in moderation), fatty fish | Supports absorption of fat-soluble vitamins; MCT oil may be considered if fat malabsorption occurs |
| **Fruits and vegetables** | Colorful variety, especially leafy greens, berries, cruciferous vegetables | Provides antioxidants, fiber, and micronutrients; fiber supports gut microbiome health |
| **Coffee** | 2-3 cups/day of filtered coffee (if tolerated) | Associated with reduced liver fibrosis progression and lower HCC risk |

**Limit or Avoid:**

| **Item** | **Reason** |
| --- | --- |
| **Alcohol** | Absolute contraindication; accelerates liver damage |
| **Raw/undercooked shellfish** | Risk of life-threatening *Vibrio* infection in cirrhosis |
| **High-sodium processed foods** | May predispose to fluid retention; restrict to <2,300 mg/day unless ascites develops (then <2,000 mg/day) |
| **Added sugars & sugary beverages** | Promote hepatic steatosis and insulin resistance |
| **Excessive saturated/trans fats** | May worsen metabolic parameters; limit fried foods, fatty meats, commercial baked goods |
| **Unverified herbal supplements** | Risk of hepatotoxicity; consult physician before using any supplement |

**5. Additional Nutritional Considerations**

**Micronutrient Supplementation (consider screening and supplementation):**

- **Vitamin D:** Deficiency is common in cirrhosis; supplement if levels <30 ng/mL (typically 800-2000 IU/day)

.

- **Zinc:** Deficiency correlates with taste disturbance, anorexia, and hepatic encephalopathy risk; consider supplementation if serum zinc is low .
- **B vitamins (especially thiamine):** Consider oral thiamine 50-100 mg/day, particularly if history of poor intake or alcohol use

.

- **Fat-soluble vitamins (A, E, K):** Monitor levels; supplement only if deficient due to risk of toxicity (especially vitamin A).

**Monitoring and Follow-up:**

- **Regular nutritional assessment:** Screen for sarcopenia using handgrip strength, mid-arm circumference, or CT-based muscle analysis if available

.

- **Weight monitoring:** Track weight weekly; unintentional loss >5% in 3 months warrants intervention.
- **Laboratory monitoring:** Periodic assessment of albumin, prealbumin, vitamin D, zinc, and renal function.
- **Multidisciplinary care:** Coordinate with hepatology, dietetics, and primary care for comprehensive management.

**Special Considerations for HBV:**

- Continue antiviral therapy as prescribed; nutrition supports but does not replace antiviral treatment.
- No specific HBV-related dietary restrictions beyond standard cirrhosis recommendations.
- Ensure adequate protein to support immune function and liver regeneration.

**Practical Daily Example Menu** (~1,600 kcal, 65 g protein):

- **Breakfast:** Oatmeal with berries + 1 boiled egg + coffee
- **Mid-morning snack:** Greek yogurt (150g) with almonds (5-6)
- **Lunch:** Grilled salmon (90g) + quinoa (½ cup) + steamed broccoli + olive oil dressing
- **Afternoon snack:** Apple with 1 tbsp peanut butter
- **Dinner:** Chicken stir-fry with tofu (60g chicken + 50g tofu) + brown rice (⅓ cup) + mixed vegetables
- **Late-evening snack (8-9 PM):** Whole-grain crackers with cottage cheese or a small banana with milk

*Note: Individualize portions based on appetite, tolerance, and metabolic goals. Refer to a registered dietitian specializing in liver disease for personalized meal planning.*

## C1-11

**Dietary and Nutritional Recommendations for MASLD-Related Compensated Cirrhosis**

**Patient Profile**: Male, 70 years, 171 cm, 76 kg (BMI 26.0 kg/m²), MASLD-related compensated cirrhosis

**1. Overall Dietary Principles**

**Core Goals**:

- Prevent progression of liver disease and sarcopenia
- Maintain muscle mass and functional status
- Support metabolic health while managing MASLD risk factors
- Avoid malnutrition without promoting excessive weight gain

**Key Principles**:

- **Adequate energy intake**: Target 30-35 kcal/kg/day (~2,300-2,650 kcal/day) to prevent catabolism

- **High-quality protein emphasis**: Prioritize protein distribution throughout the day rather than restriction

- **Mediterranean-style pattern**: Emphasize whole grains, vegetables, fruits, legumes, fish, olive oil, and nuts
- **Limit processed foods, added sugars, and saturated fats** to support MASLD management
- **Avoid alcohol completely**—non-negotiable in cirrhosis

**2. Recommended Daily Protein Intake**

| **Parameter** | **Recommendation** | **Rationale** |
| --- | --- | --- |
| **Total protein** | **1.2–1.5 g/kg/day** = **91–114 g/day** | ESPEN/ACG guidelines for compensated cirrhosis to prevent sarcopenia |
| **Protein timing** | Distribute evenly across 4–6 meals/snacks | Maximizes muscle protein synthesis; reduces overnight catabolism |
| **Protein quality** | Prioritize: fish, poultry, eggs, dairy, legumes, soy; limit red/processed meats | Vegetable and dairy proteins associated with lower mortality in cirrhosis |
| **BCAA consideration** | Consider BCAA-enriched supplements if oral intake inadequate or early HE develops | May improve hepatic encephalopathy resolution and nitrogen balance |

**Critical**: Do NOT restrict protein—even with mild hepatic encephalopathy—as this worsens sarcopenia and outcomes

**3. Meal Frequency and Eating Pattern**

**Optimal Pattern**: 4–6 smaller meals/snacks per day, including:

| **Meal Timing** | **Recommendation** | **Purpose** |
| --- | --- | --- |
| **Breakfast** | Within 1 hour of waking | Break overnight fast; stimulate metabolism |
| **Mid-morning snack** | Protein + complex carb (e.g., Greek yogurt + berries) | Sustain amino acid availability |
| **Lunch** | Balanced meal with lean protein, whole grains, vegetables | Main nutrient delivery |
| **Afternoon snack** | Nuts, hummus, or cheese with whole-grain crackers | Prevent catabolic window |
| **Dinner** | Moderate portion; avoid very large evening meals | Support digestion and sleep |
| **Late-evening snack (LES)** | **200 kcal with ~50 g carbohydrate + protein** (e.g., oatmeal with milk, or toast with peanut butter) | Shortens overnight fasting; improves protein synthesis and reduces muscle breakdown |

*Physiologic rationale*: Cirrhosis creates a state of "accelerated starvation" with depleted glycogen stores; a bedtime snack exploits an anabolic window to preserve muscle

**4. Specific Food Recommendations and Restrictions**

**Encourage**

| **Food Group** | **Examples** | **Frequency/Notes** |
| --- | --- | --- |
| **Lean proteins** | Fish (salmon, sardines), skinless poultry, eggs, Greek yogurt, cottage cheese, tofu, lentils | 3–4 servings/day; prioritize fish 2–3×/week for omega-3s |
| **Complex carbohydrates** | Oats, quinoa, brown rice, whole-wheat bread, sweet potatoes | Base of meals; choose high-fiber options |
| **Vegetables** | Leafy greens, cruciferous vegetables, colorful options | ≥5 servings/day; variety for micronutrients |
| **Fruits** | Berries, apples, citrus (limit high-fructose if insulin resistant) | 2–3 servings/day; whole fruit over juice |
| **Healthy fats** | Olive oil, avocado, nuts, seeds | Use olive oil as primary fat; limit to portion control |
| **Dairy** | Low-fat milk, yogurt, cheese (if tolerated) | Calcium + protein; monitor if lactose intolerant |

**Limit or Avoid**

| **Item** | **Reason** | **Practical Guidance** |
| --- | --- | --- |
| **Alcohol** | Direct hepatotoxin; accelerates fibrosis | Complete abstinence—no exceptions |
| **Added sugars & refined carbs** | Worsen insulin resistance & hepatic steatosis | Avoid sugary drinks, pastries, white bread; read labels |
| **Processed meats** | High sodium, saturated fat, preservatives | Limit bacon, sausage, deli meats; choose fresh proteins |
| **High-sodium foods** | Promotes fluid retention; risk of ascites | Target **<2,000 mg sodium/day**; avoid canned soups, salty snacks, restaurant meals |
| **Fried foods & trans fats** | Promote inflammation and dyslipidemia | Bake, grill, or steam instead of frying |
| **Raw shellfish** | Risk of Vibrio infection in cirrhosis | Cook all seafood thoroughly |

**Sodium management tip**: Use herbs, lemon, vinegar, garlic, and spices instead of salt; choose "no salt added" products; rinse canned beans/vegetables.

**5. Additional Nutritional Considerations**

**Micronutrient Monitoring & Supplementation**

- **Vitamin D**: Deficiency common in liver disease; check levels and supplement if <30 ng/mL

- **Zinc**: Often depleted; consider 25–50 mg elemental zinc/day if deficient (supports taste, immunity, wound healing)
- **B vitamins (especially thiamine)**: Screen for deficiency, particularly if prior alcohol use
- **Fat-soluble vitamins (A, E, K)**: Monitor if cholestasis develops; supplement only under supervision

**Sarcopenia Prevention Strategy**

- **Combine nutrition + resistance exercise**: Aim for 2–3 sessions/week of light resistance training (bands, bodyweight)
- **Protein timing around activity**: Consume 20–30 g protein within 1–2 hours post-exercise
- **Monitor functional status**: Track grip strength, gait speed, or chair-stand tests at visits

**Practical Implementation Tips**

1. **Start with small changes**: Add one protein-rich snack or the bedtime snack first
2. **Use oral nutritional supplements (ONS) if needed**: High-protein, low-sodium shakes can help meet targets if oral intake is insufficient
3. **Involve a registered dietitian**: Personalized meal planning improves adherence
4. **Hydration**: 1.5–2 L fluids/day unless fluid-restricted; water preferred
5. **Food safety**: Emphasize thorough cooking, hand hygiene, and avoiding high-risk foods due to immunocompromise

**Red Flags to Report**

- Unintentional weight loss >5% in 1 month
- New confusion, daytime sleepiness (possible HE)
- Worsening abdominal girth or edema (ascites)
- Loss of appetite lasting >3 days

**Sample Daily Meal Framework (~2,400 kcal; 100 g protein)**

| **Time** | **Meal** | **Example** |
| --- | --- | --- |
| 7:00 AM | Breakfast | 2 eggs scrambled with spinach + 1 slice whole-wheat toast + ½ avocado + black coffee |
| 10:00 AM | Snack | Greek yogurt (150 g) + ¼ cup berries + 1 tbsp almonds |
| 1:00 PM | Lunch | Grilled salmon (100 g) + quinoa (½ cup cooked) + steamed broccoli + olive oil drizzle |
| 4:00 PM | Snack | Apple + 2 tbsp peanut butter |
| 7:00 PM | Dinner | Skinless chicken breast (90 g) + lentil soup (1 cup) + mixed green salad with vinaigrette |
| 9:30 PM | Late-evening snack | Oatmeal (½ cup dry) made with milk + cinnamon + 1 tbsp walnuts |

**Final Clinical Note**: This patient's compensated status is an opportunity for proactive nutritional intervention. Consistent adherence to these recommendations can preserve muscle mass, delay decompensation, and improve quality of life. Reassess nutritional status, weight, and functional measures every 3–6 months, and adjust targets as needed .

## C1-12

**Dietary and Nutritional Recommendations for Compensated Alcohol-Related Cirrhosis**

**Patient Profile:** Female, 73 years old, 159 cm, 66 kg (BMI 26.1 kg/m²), compensated alcohol-related cirrhosis

**1. Overall Dietary Principles**

**Priority Goals:**

- **Complete alcohol abstinence** – This is the single most critical intervention; abstinence can halt disease progression and may allow partial hepatic recovery
- **Prevent sarcopenia and malnutrition** – Muscle loss is a major predictor of mortality and complications in cirrhosis, even in compensated stages
- **Maintain adequate energy and protein intake** – Patients with cirrhosis enter a catabolic state more rapidly during fasting; nutritional support should be proactive, not reactive
- **Emphasize nutrient-dense, whole foods** – Focus on quality calories that support liver function and muscle preservation

**Avoid:**

- Prolonged fasting (>3-4 hours while awake)
- High-fructose/sucrose diets, which promote hepatic steatosis and fibrosis
- Unnecessary dietary restrictions that may worsen malnutrition

**2. Recommended Daily Protein Intake**

| **Parameter** | **Recommendation** | **Rationale** |
| --- | --- | --- |
| **Target intake** | **1.2–1.5 g/kg/day** = **79–99 g protein/day** | Prevents muscle loss and supports hepatic regeneration; protein restriction is *not* recommended even in compensated cirrhosis |
| **Distribution** | Spread evenly across meals + evening snack | Optimizes muscle protein synthesis; reduces overnight catabolism |
| **Protein sources** | Prioritize: plant proteins (legumes, tofu), dairy, fish, poultry; limit red/processed meats | Plant-based proteins may be better tolerated and produce less ammonia; vegetable protein sources are associated with lower hepatic encephalopathy risk |

**Practical examples of 20–25 g protein portions:**

- 100 g cooked chicken/fish
- 1 cup Greek yogurt + 2 tbsp nuts
- ¾ cup lentils or beans + 1 slice whole grain bread
- 2 eggs + 1 oz cheese

**3. Meal Frequency and Eating Pattern**

**Recommended Pattern:**

- **4–6 small meals/snacks per day** (every 3–4 hours while awake)
- **Mandatory late-evening snack** (between 7–10 PM) containing protein + complex carbohydrate

**Rationale for evening snack:**

- Compensated cirrhosis accelerates glycogen depletion; overnight fasting >10 hours promotes muscle breakdown
- A bedtime snack (e.g., Greek yogurt with berries, cottage cheese with crackers, or a small protein shake) reduces nocturnal catabolism and improves nitrogen balance

**Sample Daily Pattern:**

| **Time** | **Meal** | **Example** |
| --- | --- | --- |
| 7 AM | Breakfast | Oatmeal with milk, walnuts, berries |
| 10 AM | Snack | Greek yogurt or hard-boiled egg + fruit |
| 1 PM | Lunch | Grilled salmon, quinoa, steamed vegetables |
| 4 PM | Snack | Hummus with whole-grain crackers |
| 7 PM | Dinner | Lean poultry, sweet potato, green beans |
| 9 PM | Evening snack | Cottage cheese or protein shake |

**4. Specific Food Recommendations and Restrictions**

**Encourage:**

| **Food Group** | **Examples** | **Benefits** |
| --- | --- | --- |
| **Lean proteins** | Fish, poultry, eggs, legumes, tofu, low-fat dairy | Supports muscle mass; plant proteins may reduce encephalopathy risk |
| **Complex carbohydrates** | Oats, brown rice, whole-grain bread, sweet potatoes | Sustained energy; fiber supports gut health |
| **Healthy fats** | Olive oil, avocado, nuts, fatty fish (salmon, mackerel) | Anti-inflammatory; omega-3s may benefit liver health |
| **Fruits & vegetables** | Colorful variety, especially leafy greens, berries | Antioxidants, fiber, micronutrients |
| **Coffee** | 2–3 cups/day (if tolerated) | Associated with reduced fibrosis progression and HCC risk in chronic liver disease |

**Limit or Avoid:**

| **Item** | **Reason** |
| --- | --- |
| **Alcohol** | Absolute contraindication; accelerates liver damage |
| **Added sugars & high-fructose foods** | Promote hepatic lipogenesis and insulin resistance |
| **Processed meats & excessive red meat** | Higher ammonia load; may worsen encephalopathy risk |
| **Excess sodium** | If ascites develops, restrict to <2 g/day; in compensated disease, moderate intake (~2.3 g/day) is reasonable unless fluid retention occurs |
| **Raw shellfish** | Risk of *Vibrio* infection in liver disease |

**5. Additional Nutritional Considerations**

**Micronutrient Assessment & Supplementation**

Patients with alcohol-related cirrhosis are at high risk for deficiencies. Recommend screening and targeted supplementation:

| **Nutrient** | **Rationale** | **Typical Supplementation*** |
| --- | --- | --- |
| **Thiamine (B1)** | Critical for neurologic function; deficiency common in AUD | 100 mg/day oral (higher if deficiency confirmed) |
| **Folate** | Impaired absorption in liver disease; supports hematopoiesis | 1 mg/day |
| **Vitamin D** | Deficiency linked to fibrosis progression and bone loss | 800–2000 IU/day; monitor 25-OH-D levels |
| **Zinc** | Deficiency causes dysgeusia, poor wound healing, immune dysfunction | 25–50 mg elemental zinc/day if deficient |
| **Multivitamin** | Broad coverage for potential gaps | Daily liver-specific formulation (avoid excess iron/vitamin A) |

**Doses should be individualized based on lab values and clinical assessment*

**Physical Activity Integration**

- **Combine nutrition with resistance exercise** (2–3x/week) to maximize muscle preservation
- Even light walking improves insulin sensitivity and functional status in older adults with cirrhosis

**Monitoring Plan**

- **Monthly weight checks** (note: fluid retention may mask true weight changes)
- **Handgrip strength or sit-to-stand test** every 3–6 months to screen for sarcopenia
- **Albumin, prealbumin, zinc, vitamin D** labs every 6 months or as clinically indicated
- **Reassess nutritional plan** if weight loss >5% in 3 months or functional decline occurs

**Red Flags Requiring Prompt Re-evaluation**

- Unintentional weight loss
- New confusion, sleep disturbance, or asterixis (possible hepatic encephalopathy)
- Ascites, edema, or jaundice (signs of decompensation)
- Poor oral intake due to anorexia, nausea, or early satiety

**Key Takeaway for This Patient:** At age 73 with compensated cirrhosis, the priority is *preventing* malnutrition and sarcopenia through adequate protein (79–99 g/day), frequent meals with an evening snack, complete alcohol abstinence, and micronutrient repletion. These strategies can preserve functional status, reduce hospitalization risk, and improve quality of life

## C2-01

**Dietary and Nutritional Recommendations for HBV-Related Cirrhosis with Ascites**

**Patient Profile:** Male, 33 y/o, 173 cm, 69 kg (BMI 23.0 kg/m²), HBV-cirrhosis with ascites, clinically stable, oral intake possible.

**1. Overall Dietary Principles**

**Prioritize adequate nutrition to prevent/treat sarcopenia** – Malnutrition affects >60% of patients with advanced cirrhosis and is independently associated with increased mortality

.

**Do NOT restrict protein** – Protein restriction is outdated and harmful; adequate protein intake is essential to preserve muscle mass and support liver regeneration

.

**Implement moderate sodium restriction** – Limit sodium to **≤2,000 mg/day (≈5 g salt)** to help manage ascites while avoiding excessive restriction that may worsen nutrition

emedicine.medscape.com

.

**Focus on nutrient-dense, whole foods** – Emphasize fresh, minimally processed foods to maximize micronutrient intake and minimize sodium load.

**Avoid alcohol completely** – Critical in HBV-cirrhosis to prevent further hepatic injury.

**2. Recommended Daily Protein Intake**

| **Parameter** | **Recommendation** |
| --- | --- |
| **Target protein intake** | **1.2–1.5 g/kg/day** = **83–104 g protein/day** (based on actual body weight) |
| **Rationale** | Cirrhotic patients have elevated protein requirements due to increased turnover and risk of muscle wasting; intakes <0.75 g/kg/day are insufficient |
| **Protein sources** | Prioritize high-biological-value proteins: eggs, fish, poultry, dairy, legumes, soy. Plant and animal proteins can be combined; no need to avoid meat-based protein |
| **If hepatic encephalopathy develops** | Continue adequate protein; consider branched-chain amino acid (BCAA) supplementation if protein intolerance occurs |

📝 *Practical tip:* 100 g cooked chicken breast ≈ 31 g protein; 1 large egg ≈ 6 g; 200 mL milk ≈ 7 g; 100 g tofu ≈ 8 g.

**3. Meal Frequency and Eating Pattern**

**Eat 4–6 small meals/snacks per day** – Frequent feeding reduces catabolism during prolonged fasting states common in cirrhosis

.

**Include a late-evening snack (LES)** – A carbohydrate + protein snack (e.g., yogurt with fruit, toast with peanut butter) between 7–10 PM improves nitrogen balance, reduces overnight muscle breakdown, and may improve hepatic function

.

**Avoid prolonged fasting** – Do not skip meals; aim for no more than 3–4 hours between daytime feedings.

**Coordinate with diuretic timing** – If taking morning diuretics (e.g., spironolactone/furosemide), ensure adequate fluid and electrolyte intake with meals to prevent dehydration or electrolyte imbalance.

**4. Specific Food Recommendations and Restrictions**

**Encourage**

| **Food Group** | **Examples** | **Rationale** |
| --- | --- | --- |
| **Lean proteins** | Skinless poultry, fish (especially fatty fish like salmon), eggs, Greek yogurt, legumes, tofu | High-quality protein with lower saturated fat |
| **Complex carbohydrates** | Oats, brown rice, whole-wheat bread, quinoa, sweet potatoes | Sustained energy, fiber for gut health |
| **Fresh fruits/vegetables** | Berries, apples, leafy greens, cruciferous vegetables (cooked if bloating) | Antioxidants, vitamins, fiber; choose low-sodium preparation |
| **Healthy fats** | Olive oil, avocado, nuts (unsalted), seeds | Support calorie density without sodium load |
| **Low-sodium flavorings** | Lemon juice, vinegar, herbs, spices, garlic, ginger | Enhance palatability without adding salt |

**Limit or Avoid**

| **Item** | **Reason** |
| --- | --- |
| **High-sodium foods** | Processed meats, canned soups, soy sauce, pickled foods, fast food, salty snacks – major contributors to fluid retention |
| **Added sugars & refined carbs** | Sugary beverages, pastries, white bread – may worsen insulin resistance common in cirrhosis |
| **Raw shellfish** | Risk of Vibrio vulnificus infection in immunocompromised cirrhotic patients |
| **Moldy or improperly stored foods** | Risk of aflatoxin exposure, particularly relevant in HBV-endemic regions |
| **Herbal supplements without medical approval** | Many herbs (e.g., kava, comfrey) are hepatotoxic; always consult hepatology team first |

**Sodium Management Tips**

- Read nutrition labels: choose items with <140 mg sodium per serving when possible
- Rinse canned beans/vegetables to reduce sodium by ~40%
- Cook at home using fresh ingredients; limit restaurant meals
- Use potassium-based salt substitutes **only if serum potassium is normal and renal function is preserved** (monitor with physician)

**5. Additional Nutritional Considerations**

**Micronutrient supplementation** – Cirrhosis increases risk of deficiencies:

- **Vitamin D**: Supplement if deficient (common; associated with worse outcomes)
- **B-complex vitamins**: Especially thiamine (B1) and folate, particularly if prior alcohol use
- **Zinc**: Deficiency may contribute to taste changes and encephalopathy; consider supplementation if levels low
- **Multivitamin**: A liver-specific formulation without iron (unless deficient) may be appropriate

**Monitor for sarcopenia** – Assess mid-arm muscle circumference or grip strength periodically; consider referral to physical therapy for resistance exercise to preserve muscle mass

.

**Fluid restriction** – Generally **not required** unless serum sodium <125 mmol/L; if needed, limit to 1–1.5 L/day under medical supervision

gut.bmj.com

.

**Weight monitoring** – Track daily weight at home; sudden increases (>1 kg in 2 days) may indicate worsening ascites requiring medical review.

**Coordinate with HBV treatment** – Ensure antiviral therapy adherence; nutrition supports treatment efficacy and liver recovery.

**When to seek urgent care** – Report new confusion, severe abdominal pain, vomiting blood, black stools, or rapid weight gain (>2 kg/week).

**Follow-up**: Reassess nutritional status every 3 months or with clinical changes. Consider referral to a registered dietitian specializing in liver disease for personalized meal planning and ongoing support.

## C2-02

**Dietary and Nutritional Recommendations for MASLD Cirrhosis with Ascites**

**Patient Profile:** Female, 37 y/o, 159 cm, 56 kg (BMI 22.2 kg/m²), MASLD cirrhosis with ascites and decreased appetite

**1. Overall Dietary Principles**

- **Prioritize adequate energy and protein intake** to prevent sarcopenia and malnutrition, which are strongly associated with poor outcomes in cirrhosis

.

- **Adopt a Mediterranean-style dietary pattern**: Emphasize vegetables, fruits, whole grains, legumes, nuts, olive oil, and lean proteins; limit processed foods, refined carbohydrates, and saturated fats
- **Avoid alcohol completely**—essential for all liver disease patients regardless of etiology.
- **Limit added sugars and fructose**: High-fructose diets promote hepatic lipogenesis and fibrosis progression; avoid sugar-sweetened beverages
- **Include coffee**: 2–4 cups/day is associated with reduced fibrosis progression and HCC risk in chronic liver disease

.

- **Focus on food quality over restriction**: Given decreased appetite, prioritize nutrient-dense, palatable foods the patient enjoys.

**2. Recommended Daily Protein Intake**

| **Parameter** | **Recommendation** |
| --- | --- |
| **Target intake** | **1.2–1.5 g/kg/day** = **67–84 g protein/day** (based on 56 kg) |
| **Distribution** | Spread evenly across meals/snacks to optimize muscle protein synthesis |
| **Protein sources** | Prioritize **plant-based proteins** (legumes, tofu, lentils) and dairy; include lean poultry/fish. Vegetable proteins have lower methionine/tryptophan and higher fiber, potentially reducing ammonia production  . |
| **Key point** | **Do NOT restrict protein**, even with ascites. Protein restriction worsens sarcopenia and may increase hepatic encephalopathy risk  . |

*Practical tip:* If appetite is poor, consider oral nutritional supplements with branched-chain amino acids (BCAAs) if protein tolerance is limited

.

**3. Meal Frequency and Eating Pattern**

| **Strategy** | **Rationale & Implementation** |
| --- | --- |
| **Small, frequent meals** | 4–6 eating occasions/day (every 3–4 hours while awake) to reduce catabolism and accommodate decreased appetite  . |
| **Late-evening snack (LES)** | **Essential**: 150–200 kcal carbohydrate + protein snack before bed (e.g., Greek yogurt with berries, toast with peanut butter, oatmeal). Reduces overnight muscle breakdown and may improve ascites control |
| **Avoid prolonged fasting** | Do not fast >12 hours overnight; the LES helps maintain metabolic stability  . |
| **Breakfast importance** | Encourage consistent breakfast intake, associated with better cognitive function in cirrhosis  . |

**4. Specific Food Recommendations & Restrictions**

**Encourage:**

- **Protein**: Eggs, Greek yogurt, cottage cheese, lentils, chickpeas, tofu, fish (salmon, cod), skinless poultry
- **Complex carbohydrates**: Oats, quinoa, brown rice, whole-grain bread (in moderation if insulin resistant)
- **Healthy fats**: Olive oil, avocado, nuts (unsalted), seeds
- **Vegetables & fruits**: Variety of colors; limit very high-fructose fruits (e.g., mango, grapes) if metabolic syndrome present
- **Fluids**: Water, herbal teas; coffee as tolerated

**Limit/Avoid:**

- **Sodium**: **<2,000 mg/day** (≈5 g salt) to help manage ascites

UpToDate临床顾问

gut.bmj.com

. Practical tips:

- No added salt at table or during cooking
- Avoid processed foods, canned soups, deli meats, salty snacks
- Read labels; choose "no salt added" versions
- Use herbs, lemon, vinegar, garlic for flavor
- **Added sugars**: Limit sweets, sodas, fruit juices, pastries
- **Saturated/trans fats**: Reduce red meat, fried foods, commercial baked goods
- **Raw shellfish**: Risk of Vibrio infection in cirrhosis
- **Unverified supplements/herbals**: Many are hepatotoxic; review all supplements with provider

**Sodium Restriction Practical Guide:**

| **High-Sodium Foods to Avoid** | **Low-Sodium Alternatives** |
| --- | --- |
| Canned soups, broths | Homemade low-sodium broth |
| Processed meats (bacon, ham) | Fresh poultry, fish, eggs |
| Cheese (especially processed) | Unsalted nuts, avocado |
| Soy sauce, teriyaki | Lemon juice, herbs, vinegar |
| Snack chips, pretzels | Fresh fruit, unsalted popcorn |
| Frozen meals | Home-cooked meals with fresh ingredients |

**5. Additional Nutritional Considerations**

**Micronutrient Supplementation (Assess & Replace Deficiencies):**

- **Vitamin D**: Supplement if deficient (common in liver disease); supports bone health
- **Zinc**: Consider 25–50 mg elemental zinc/day if deficient or with taste changes/poor wound healing
- **Thiamine (B1)**: Especially important if any history of alcohol use; deficiency can cause Wernicke's encephalopathy
- **Multivitamin**: A liver-specific formulation without iron (unless deficient) may be prudent

**Monitoring & Follow-up:**

- **Weight**: Monitor weekly (adjust for ascites); sudden increases may indicate fluid retention
- **Appetite strategies**:
  - Eat when hunger is greatest (often morning)
  - Small portions to avoid early satiety from ascites
  - Nutrient-dense smoothies if solid food intake is low
- **Physical activity**: Encourage light resistance exercises (as tolerated) to combat sarcopenia; aim for ≥150 min/week moderate activity
- **Hydration**: Fluid restriction only if serum sodium <126 mEq/L with fluid retention

; otherwise, maintain adequate hydration

**Red Flags to Report:**

- Worsening confusion, drowsiness (possible hepatic encephalopathy)
- Rapid weight gain (>1 kg/day) suggesting worsening ascites
- Persistent nausea/vomiting preventing oral intake
- Signs of infection (fever, abdominal pain)

**Sample Daily Meal Pattern (≈1,800–2,000 kcal; 70–80 g protein; <2,000 mg sodium)**

| **Time** | **Meal/Snack** | **Example** |
| --- | --- | --- |
| 7:30 AM | Breakfast | Scrambled eggs (2) with spinach, 1 slice whole-grain toast, ½ avocado, coffee |
| 10:00 AM | Snack | Greek yogurt (plain, low-sodium) with berries and 1 tbsp unsalted almonds |
| 12:30 PM | Lunch | Grilled salmon (100 g), quinoa (½ cup cooked), steamed broccoli with olive oil & lemon |
| 3:30 PM | Snack | Apple slices with 1 tbsp unsalted peanut butter |
| 6:30 PM | Dinner | Lentil soup (homemade, low-sodium), small whole-grain roll, side salad with olive oil/vinegar |
| 9:00 PM | Late-evening snack | Oatmeal (½ cup dry) made with milk, topped with cinnamon and a few walnuts |

**Key Takeaway**: In cirrhosis, *adequate nutrition is therapeutic*. The goals are to preserve muscle mass, manage ascites through sodium control, and support liver function through a balanced, palatable eating pattern. Regular follow-up with a dietitian specializing in liver disease is strongly recommended to individualize and adjust this plan.

## C2-03

**Dietary and Nutritional Recommendations for Alcohol-Related Cirrhosis with Moderate Ascites**

**Patient Summary:** Male, 41 years, 168 cm, 84 kg (BMI 29.8 kg/m²), alcohol-related cirrhosis with moderate ascites

*Note: For nutritional calculations, adjusted body weight should be used due to ascites. With moderate ascites, subtract ~10% from measured weight:* ***adjusted weight ≈ 75-76 kg****.*

**1. Overall Dietary Principles**

| **Principle** | **Rationale & Target** |
| --- | --- |
| **Complete alcohol abstinence** | Essential to halt disease progression and improve outcomes |
| **Adequate energy intake** | 35-40 kcal/kg/day (using adjusted weight) = **~2,600-3,000 kcal/day** to prevent catabolism |
| **High-protein diet** | Prevents sarcopenia; protein restriction is **not** recommended even with hepatic encephalopathy |
| **Sodium restriction** | Limit to **<2 g sodium/day** (~5 g salt) to manage ascites; avoid "no added salt" extremes that reduce caloric intake |
| **Balanced macronutrients** | 50-70% carbohydrates (complex, low-fructose), 10-20% fat (emphasize mono/polyunsaturated) |
| **Plant-forward protein emphasis** | Vegetable and dairy proteins may be better tolerated and associated with lower mortality than high animal protein intake |

**2. Recommended Daily Protein Intake**

**Target: 1.2-1.5 g/kg/day using adjusted body weight**

- **Calculation:** 75.6 kg × 1.2-1.5 g/kg = **90-115 g protein/day**
- **Distribution:** Spread evenly across meals (20-30 g per meal) to optimize muscle protein synthesis
- **Protein source guidance:**
  - Prioritize: legumes, lentils, tofu, eggs, low-fat dairy, fish, poultry
  - Moderate: lean red meat (limit to 1-2×/week)
  - Consider branched-chain amino acid (BCAA) supplementation (4 g/day) if oral intake is inadequate or if hepatic encephalopathy develops

aronia-charlottenburg.com

Semantic Scholar

*Important:* Do not restrict protein for hepatic encephalopathy prophylaxis—this worsens outcomes .

**3. Meal Frequency and Eating Pattern**

| **Recommendation** | **Clinical Rationale** |
| --- | --- |
| **3-5 small meals daily** | Prevents "accelerated starvation" physiology seen in cirrhosis; reduces overnight protein catabolism |
| **Late evening snack** (containing protein + complex carbohydrate) | Improves nitrogen balance and muscle preservation; equivalent to preventing 2-3 days of fasting in healthy individuals |
| **Avoid prolonged fasting** (>4-6 hours awake without intake) | Cirrhotic patients deplete glycogen stores rapidly and shift to muscle breakdown for gluconeogenesis |
| **If NBM for procedures**: minimize duration; consider IV glucose (2-3 g/kg/day) if fasting unavoidable | Prevents switch to catabolic state; balance against fluid overload risk |

*Sample timing:* Breakfast (7 AM), mid-morning snack (10 AM), lunch (1 PM), afternoon snack (4 PM), dinner (7 PM), bedtime snack (9-10 PM)

**4. Specific Food Recommendations and Restrictions**

**Encourage**

| **Food Category** | **Examples** | **Notes** |
| --- | --- | --- |
| **Plant proteins** | Lentils, chickpeas, black beans, tofu, tempeh | Rich in arginine/ornithine; support ammonia metabolism |
| **Low-sodium whole foods** | Fresh fruits/vegetables, unsalted nuts, oats, quinoa, brown rice | Naturally low in sodium; high in fiber to support gut health |
| **Healthy fats** | Olive oil, avocado, fatty fish (salmon, mackerel), walnuts | Anti-inflammatory; support fat-soluble vitamin absorption |
| **Dairy (if tolerated)** | Greek yogurt, cottage cheese, low-fat milk | High-quality protein; calcium for bone health |
| **Coffee** | 3-4 cups/day (if tolerated) | Associated with reduced fibrosis progression and HCC risk  onlinelibrary.wiley.com |

**Limit or Avoid**

| **Item** | **Reason** |
| --- | --- |
| **Added salt & high-sodium processed foods** | Deli meats, canned soups, frozen meals, soy sauce, packaged snacks drive ascites |
| **Alcohol (all forms)** | Absolute contraindication; accelerates liver damage |
| **Excess simple sugars/fructose** | May worsen hepatic steatosis and insulin resistance |
| **Raw shellfish** | Risk of *Vibrio* infection in immunocompromised cirrhotic patients |
| **High-dose herbal supplements** | Risk of drug-induced liver injury; many "liver support" supplements lack evidence and may be hepatotoxic |

**Practical Sodium Restriction Tips**

- Use herbs, lemon, vinegar, garlic, and spices instead of salt for flavor
- Read labels: choose items with <140 mg sodium per serving
- Rinse canned beans/vegetables to reduce sodium by ~40%
- If strict restriction reduces appetite/caloric intake, discuss with provider about modest relaxation with optimized diuretic therapy

**5. Additional Nutritional Considerations**

**Micronutrient Supplementation**

| **Nutrient** | **Rationale** | **Typical Supplementation** |
| --- | --- | --- |
| **Multivitamin with minerals** | Deficiencies common due to malabsorption, reduced storage, poor intake  europepmc.org | Daily standard adult formulation |
| **Vitamin D** | Deficiency prevalent; linked to mortality and bone disease | 2,000 IU/day maintenance; 50,000 IU/week × 8-12 weeks if deficient (target 25-OH-D ≥30 ng/mL) |
| **Thiamine (B1)** | Critical if any ongoing alcohol use; deficiency risk high | 100 mg orally twice daily if alcohol use continues |
| **Zinc** | Deficiency impairs ammonia detoxification; may improve taste/appetite | 150-175 mg elemental zinc/day if deficient or symptomatic |
| **Calcium** | Bone health; osteoporosis risk elevated in cirrhosis | 1,000-1,200 mg/day total (diet + supplement) |

**Monitoring & Follow-Up**

- **Weight**: Track trends but interpret cautiously with ascites; focus on mid-arm circumference or handgrip strength for muscle assessment

- **Laboratory**: Monitor albumin, prealbumin, electrolytes, vitamin D, zinc periodically
- **Bone health**: Baseline DEXA scan recommended; consider bisphosphonates if osteoporosis confirmed

gut.bmj.com

- **Dietitian referral**: Strongly recommended for personalized meal planning and monitoring, especially with decompensation

**Special Situations**

| **Scenario** | **Management** |
| --- | --- |
| **Hepatic encephalopathy** | Continue high protein; consider BCAA supplementation; ensure adequate fiber for ammonia clearance |
| **Refeeding risk** (after hospitalization/NBM) | Restart nutrition gradually; monitor phosphate, potassium, magnesium; ensure thiamine repletion before feeding |
| **Sarcopenic obesity** (BMI >30 with muscle loss) | Hypocaloric (500-800 kcal deficit), high-protein (~1.5 g/kg) diet + resistance exercise |
| **Poor oral intake** | Consider oral nutritional supplements; if inadequate, short-term enteral feeding may be beneficial |

**Practical Patient Handout Summary**

**Eat**: Small, frequent meals with protein at each; include a bedtime snack (e.g., Greek yogurt + berries, cottage cheese + crackers)

**Limit salt**: Avoid processed foods; flavor with herbs, not salt

**Zero alcohol**: Non-negotiable for liver recovery

**Take daily**: Multivitamin + vitamin D; thiamine if any alcohol exposure

**Call your team if**: Appetite drops >3 days, confusion develops, or weight changes rapidly

## C2-04

**Dietary and Nutritional Recommendations for HBV Cirrhosis with Ascites**

**Patient Summary:** 44-year-old female, 161 cm, 72 kg (BMI 27.8 kg/m²), HBV cirrhosis with ascites and mild lower extremity edema. *Note: Actual "dry weight" is likely ~67-69 kg after accounting for fluid retention; nutritional calculations below use estimated dry weight.*

**1. Overall Dietary Principles**

**Prioritize adequate nutrition over extreme restriction.** Patients with cirrhosis enter "accelerated starvation" physiology after short fasting periods, leading to muscle breakdown

.

**Focus on nutrient density, not just calories.** Choose whole foods that provide protein, complex carbohydrates, healthy fats, vitamins, and minerals.

**Sodium management is critical but must be balanced with palatability.** A moderate sodium restriction (~2,000 mg/day or 88 mmol) is recommended for ascites, but overly strict restriction may reduce overall food intake and worsen malnutrition

.

**Avoid alcohol completely**—non-negotiable for HBV cirrhosis.

**Coordinate with your hepatology team** regarding diuretic therapy (spironolactone ± furosemide), as nutritional and pharmacologic management of ascites work synergistically.

**2. Recommended Daily Protein Intake**

| **Parameter** | **Recommendation** | **Rationale** |
| --- | --- | --- |
| **Target** | **1.2–1.5 g/kg/day** (based on *dry weight*) ≈ **82–102 g/day** | Prevents sarcopenia, supports immune function, and is *protective* against hepatic encephalopathy |
| **Protein sources** | Prioritize: • Plant proteins (tofu, lentils, beans) • Dairy (Greek yogurt, cottage cheese) • Lean poultry/fish • Eggs | Vegetable proteins may reduce ammonia production and improve encephalopathy risk |
| **Important** | **Do NOT restrict protein**, even if mild hepatic encephalopathy occurs | Protein restriction increases muscle catabolism and may worsen outcomes |

*Practical tip:* Distribute protein evenly across meals (~20–30 g per meal/snack) to optimize muscle protein synthesis.

**3. Meal Frequency and Eating Pattern**

**Eat 4–6 small meals/snacks per day**, including a **late evening snack (LES) between 7–10 PM**

.

**Why this matters:**

- Cirrhotic livers have depleted glycogen stores; fasting >4–6 hours triggers muscle breakdown for gluconeogenesis
- A bedtime snack containing carbohydrate + protein reduces overnight catabolism, improves nitrogen balance, and may slow sarcopenia progression

**Sample daily pattern:**

- Breakfast (7–8 AM)
- Mid-morning snack (10 AM)
- Lunch (12–1 PM)
- Afternoon snack (3–4 PM)
- Dinner (6–7 PM)
- **Late evening snack (8–9 PM)** ← *Critical component*

*LES ideas:* Greek yogurt with berries, whole-grain crackers with cheese, small bowl of oatmeal with nuts, or a protein shake.

**4. Specific Food Recommendations & Restrictions**

**ENCOURAGE**

| **Food Group** | **Examples** | **Notes** |
| --- | --- | --- |
| **Lean proteins** | Skinless poultry, fish (cooked), eggs, tofu, lentils, Greek yogurt | Aim for variety; include plant sources daily |
| **Complex carbohydrates** | Oats, brown rice, quinoa, whole-grain bread, sweet potatoes | Provide sustained energy; support LES goals |
| **Healthy fats** | Olive oil, avocado, nuts (unsalted), seeds | Limit saturated fats; avoid fried foods |
| **Fruits & vegetables** | All varieties (well-washed); prioritize colorful options | Provide antioxidants, fiber, micronutrients |
| **Calcium/Vitamin D sources** | Fortified plant milks, low-sodium cheese, canned salmon (with bones) | Bone health is often compromised in cirrhosis |

**LIMIT/AVOID**

| **Item** | **Reason** |
| --- | --- |
| **Added salt & high-sodium foods** | Processed meats, canned soups, fast food, soy sauce, pickled foods, salty snacks |
| **Raw/undercooked shellfish** | Risk of *Vibrio vulnificus* infection, which can be fatal in cirrhosis |
| **Alcohol (any amount)** | Accelerates liver damage; absolute contraindication |
| **Excess simple sugars** | May worsen insulin resistance; prioritize complex carbs |
| **High-dose herbal supplements** | Many are hepatotoxic; consult your hepatologist before taking any supplement |

*Practical sodium tips:*

- Use herbs, lemon, vinegar, garlic, and spices instead of salt for flavor
- Rinse canned beans/vegetables to reduce sodium
- Choose "low-sodium" or "no-salt-added" labeled products
- Avoid restaurant meals when possible (often very high in hidden sodium)

**5. Additional Nutritional Considerations**

**Fluid intake:** Routine fluid restriction is *not* recommended for ascites unless serum sodium <125 mEq/L

. Focus on sodium control + diuretics instead.

**Micronutrient supplementation:**

- **Multivitamin daily** (without iron unless deficient)
- **Thiamine (B1)**: Especially important if any history of alcohol use
- **Vitamin D + Calcium**: High prevalence of deficiency in cirrhosis; support bone health
- **Zinc**: May be deficient; discuss testing/supplementation with your provider

**Monitor for refeeding risk:** If oral intake has been poor, restart nutrition gradually under supervision to avoid electrolyte shifts.

**Weight monitoring caveat:** Daily weights can be misleading with ascites/edema. Track trends *after* paracentesis or when fluid status is stable, and prioritize functional measures (strength, appetite, muscle mass).

**Referral recommendation:** All patients with decompensated cirrhosis should receive formal dietitian consultation for individualized meal planning and adherence support

.

**Coffee consideration:** 2+ cups of coffee daily may have hepatoprotective benefits in chronic liver disease (per ACG 2025 guidance)

.

**Key Takeaway for This Patient:** Your nutritional goals are to *preserve muscle mass*, *manage ascites safely*, and *maintain quality of life*. This means adequate protein, smart sodium choices, frequent small meals with a bedtime snack, and close coordination with your liver care team. Small, consistent changes matter more than perfection.

## C2-05

**Dietary and Nutritional Recommendations for MASLD Cirrhosis with Recurrent Ascites**

**Patient Profile**: 48-year-old male, 176 cm, 95 kg (BMI 30.7 kg/m²), MASLD cirrhosis with recurrent ascites

**1. Overall Dietary Principles**

| **Principle** | **Rationale & Target** |
| --- | --- |
| **Mediterranean dietary pattern** | Plant-forward eating with vegetables, fruits, whole grains, legumes, fish, and olive oil; reduces liver fat and inflammation |
| **Adequate energy intake** | Target 25-35 kcal/kg/day based on *ideal* body weight (~75 kg) = ~1,900-2,600 kcal/day to prevent catabolism while supporting gradual weight management |
| **Strict sodium restriction** | Limit to ≤2 g sodium/day (≈5 g table salt) to manage ascites; focus on "no added salt" and avoiding processed foods |
| **Complete alcohol abstinence** | Essential to prevent further liver injury and decompensation |
| **Avoid unnecessary fasting** | Patients with cirrhosis enter "accelerated starvation" physiology after short fasts; minimize overnight fasting periods |

**2. Recommended Daily Protein Intake**

**Target: 1.2–1.5 g/kg/day based on ideal body weight**

- For this patient: ~90–110 g protein/day (using ideal weight ~75 kg)
- **Do NOT restrict protein**, even with ascites or history of hepatic encephalopathy

**Why adequate protein is critical**:

- Prevents/treats sarcopenia (present in 25-70% of cirrhosis patients)
- Muscle serves as an alternative site for ammonia detoxification, reducing hepatic encephalopathy risk
- Low protein intake is associated with *worsening* encephalopathy and higher mortality

**Protein distribution strategy**:

- Distribute evenly across meals (25-30 g per meal)
- Include high-quality sources: fish, poultry, eggs, legumes, Greek yogurt, tofu
- Consider branched-chain amino acid (BCAA) supplementation if oral intake is inadequate or if encephalopathy persists despite standard therapy

**3. Meal Frequency and Eating Pattern**

| **Recommendation** | **Practical Implementation** |
| --- | --- |
| **4–6 small meals/day** | Prevents prolonged fasting; optimizes nitrogen utilization |
| Late evening snack (LES) | 150-200 kcal containing complex carbohydrate + protein (e.g., Greek yogurt with berries, whole-grain crackers with cheese); reduces overnight muscle breakdown |
| **Avoid >4-hour daytime gaps** | Set reminders for snacks if appetite is poor |
| **Time-restricted eating NOT recommended** | Unlike general populations, cirrhosis patients benefit from frequent feeding to counteract accelerated starvation |

**4. Specific Food Recommendations and Restrictions**

**Encourage**

| **Food Group** | **Examples** | **Notes** |
| --- | --- | --- |
| **Non-starchy vegetables** | Leafy greens, broccoli, peppers, zucchini | Fill half the plate; low sodium, high fiber |
| **Lean proteins** | Fish (especially fatty fish 2x/week), skinless poultry, eggs, legumes, tofu | Prioritize plant proteins and fish; limit red meat to ≤1x/week |
| **Whole grains** | Oats, quinoa, brown rice, whole-wheat bread | Provide sustained energy and fiber |
| **Healthy fats** | Extra virgin olive oil, avocado, unsalted nuts (30g/day) | Anti-inflammatory; support fat-soluble vitamin absorption |
| **Low-sodium flavorings** | Herbs, spices, lemon juice, vinegar, garlic | Enhance palatability without salt |

**Limit/Avoid**

| **Item** | **Reason** |
| --- | --- |
| **Processed/packaged foods** | Major source of hidden sodium; often high in saturated fat and added sugars |
| **Added sugars & sugary beverages** | Worsen hepatic steatosis and insulin resistance in MASLD |
| **Red and processed meats** | Associated with inflammation; limit to occasional consumption |
| **High-sodium condiments** | Soy sauce, bouillon, canned soups, pickled foods; read labels carefully |
| **Raw shellfish** | Risk of Vibrio infection in cirrhosis |

**Sodium Management Tips**

- Cook at home using fresh ingredients when possible
- Rinse canned beans/vegetables to reduce sodium
- Choose "no salt added" versions of canned goods
- Use a food diary or app to track sodium intake initially
- If palatability limits intake, discuss *cautious* relaxation of restriction with the care team while optimizing diuretic therapy

**5. Additional Nutritional Considerations**

**Micronutrient Monitoring & Supplementation**

| **Nutrient** | **Rationale** | **Practical Approach** |
| --- | --- | --- |
| **Vitamin D** | Deficiency common; linked to bone loss and disease progression | Check 25-OH vitamin D level; supplement to maintain >30 ng/mL |
| B-complex vitamins (especially thiamine) | Deficiency risk with poor intake/alcohol history; critical for neurologic function | Empirical oral multivitamin; add thiamine 100 mg BID if ongoing alcohol use or encephalopathy |
| **Zinc** | Deficiency contributes to dysgeusia, poor wound healing, encephalopathy risk | Consider supplementation if taste changes or low levels; zinc-rich foods: pumpkin seeds, chickpeas, lean beef (occasional) |
| Fat-soluble vitamins (A, E, K) | Malabsorption possible in advanced disease | Monitor if cholestasis or steatorrhea present |

**Weight Management in MASLD Cirrhosis**

- **Long-term goal**: 7-10% weight loss improves liver histology in MASLD
- **Current priority**: Stabilize nutrition and manage ascites first; aggressive weight loss during decompensation may worsen sarcopenia
- **Approach**: Gradual deficit (300-500 kcal/day) once ascites controlled; combine with resistance exercise to preserve muscle
- **Avoid**: Very low-calorie diets or rapid weight loss

**Fluid Management**

- **No routine fluid restriction** for ascites alone; focus on sodium control
- Restrict fluids only if serum sodium <125-128 mmol/L (hyponatremia), per hepatology guidance

**Monitoring & Follow-up**

- **Monthly**: Weight (interpret cautiously with ascites), dietary adherence, symptom review
- **Every 3-6 months**: Albumin, prealbumin (contextualize with inflammation), micronutrient levels
- **Anthropometrics**: Mid-arm muscle circumference, handgrip strength to screen for sarcopenia
- **Referral**: Formal dietitian consultation strongly recommended for personalized meal planning and ongoing support

**Red Flags Requiring Prompt Re-evaluation**

- Unintentional weight loss >5% in 1 month
- Worsening confusion, asterixis (possible encephalopathy)
- Persistent nausea/vomiting limiting intake
- Signs of refeeding syndrome if nutrition is restarted after prolonged fasting (hypophosphatemia, edema, arrhythmia)

**Key Takeaway for This Patient**: Prioritize adequate protein and frequent meals to combat sarcopenia while strictly managing sodium for ascites control. Weight loss should be gradual and supervised. A registered dietitian specializing in liver disease can help tailor these principles to your food preferences, cultural background, and daily routine.

## C2-06

**Dietary and Nutritional Recommendations for Alcohol-Related Cirrhosis with Ascites**

**Patient Summary:** 52-year-old female, 157 cm, 61 kg (BMI 24.8 kg/m²), alcohol-related cirrhosis with ascites

**1. Overall Dietary Principles**

- **Complete alcohol abstinence** is non-negotiable and the foundation of all management.
- **Adequate energy intake:** Target 30-35 kcal/kg body weight/day (~1,800-2,100 kcal/day) to prevent catabolism and muscle loss

.

- **Avoid prolonged fasting:** The cirrhotic liver has depleted glycogen stores; extended fasting periods accelerate muscle breakdown

.

- **Prioritize nutrient density:** Focus on high-quality proteins, complex carbohydrates, and healthy fats while limiting empty calories.
- **Individualize with dietitian support:** Referral to a registered dietitian specializing in liver disease is strongly recommended for personalized meal planning

.

**2. Recommended Daily Protein Intake**

- **Target: 1.2–1.5 g/kg body weight/day** = approximately **73–92 g protein/day** (using actual weight)

- **Do NOT restrict protein**, even if hepatic encephalopathy is present—protein restriction worsens sarcopenia and outcomes

6443bb74ef7c532515d0-3858179a21f8875f9590fc888a54448a.ssl.cf2.rackcdn.com

.

- **Distribute protein evenly** across meals and snacks to optimize utilization and minimize ammonia spikes

.

- **Prioritize mixed protein sources:**
  - Plant-based proteins (legumes, tofu, lentils) may be better tolerated and associated with lower encephalopathy risk
  - Include dairy (Greek yogurt, cottage cheese), eggs, fish, and lean poultry
  - Limit red/processed meats if encephalopathy is a concern

**3. Meal Frequency and Eating Pattern**

- **Small, frequent meals:** Aim for **4–6 eating occasions daily** (3 main meals + 2–3 snacks) to maintain steady nutrient supply .
- **Late-evening snack (LES) is essential:** A 150–200 kcal snack containing complex carbohydrates ± protein before bedtime reduces overnight catabolism, improves nitrogen balance, and supports muscle preservation

.

- *Examples:* Whole-grain crackers with cheese, Greek yogurt with berries, oatmeal with nut butter
- **Avoid fasting >3–4 hours while awake** to prevent metabolic shift to starvation mode

.

- **Eat slowly and mindfully**, especially if early satiety from ascites is present.

**4. Specific Food Recommendations and Restrictions**

**Encourage:**

| **Category** | **Examples** |
| --- | --- |
| **Proteins** | Eggs, Greek yogurt, cottage cheese, fish, skinless poultry, tofu, lentils, beans |
| **Complex carbs** | Oats, brown rice, quinoa, whole-wheat bread, sweet potatoes |
| **Healthy fats** | Olive oil, avocado, nuts (unsalted), seeds |
| **Fruits/vegetables** | All varieties (fresh/frozen); limit high-sodium canned/prepared versions |
| **Fluids** | Water, herbal teas; monitor total fluid if hyponatremia present |

**Restrict/Avoid:**

| **Item** | **Rationale** |
| --- | --- |
| **Sodium** | Limit to **<2,000 mg/day** (≈5 g salt) to help control ascites  . Avoid adding salt, processed foods, canned soups, deli meats, salty snacks, and restaurant meals. Cook from scratch using herbs/spices for flavor  . |
| **Alcohol** | Absolute abstinence—any amount accelerates liver damage |
| **Raw shellfish** | Risk of *Vibrio* infection in immunocompromised cirrhotic patients |
| **High-sugar foods** | May worsen insulin resistance; limit if glucose intolerance present |
| **Excessive iron supplements** | Unless deficiency confirmed; cirrhosis can predispose to iron overload |

**5. Additional Nutritional Considerations**

**Micronutrient Supplementation**

Patients with alcohol-related cirrhosis are at high risk for deficiencies. Consider supplementation (after assessment):

- **Thiamine (B1):** 100 mg/day—critical to prevent Wernicke-Korsakoff syndrome

australianprescriber.tg.org.au

- **Zinc:** 25–50 mg elemental zinc/day if deficient; may improve hepatic encephalopathy and wound healing
- **Vitamin D:** Supplement to maintain serum 25(OH)D >30 ng/mL; deficiency is common and linked to worse outcomes
- **Multivitamin:** A liver-specific or standard adult formulation without excess iron or vitamin A

**Monitoring and Follow-Up**

- **Weekly weights** (same time, same scale) to monitor fluid status; report gains >1 kg/week
- **Regular labs:** Albumin, prealbumin, electrolytes, zinc, vitamin D, thiamine levels as indicated
- **Sarcopenia screening:** Assess handgrip strength or muscle mass periodically; adjust protein target to 1.5 g/kg if sarcopenia present
- **Medication review:** Some diuretics (e.g., spironolactone) affect potassium; coordinate dietary potassium with medication regimen

**Practical Tips for Ascites Management**

- Read food labels: Choose items with <140 mg sodium per serving
- Rinse canned beans/vegetables to reduce sodium by ~40%
- Use lemon juice, vinegar, garlic, herbs, and salt-free seasoning blends for flavor
- If appetite is poor due to abdominal fullness, prioritize protein/calorie-dense snacks over large volumes

**Key Takeaway:** Nutrition is therapeutic in cirrhosis. Adequate protein, controlled sodium, frequent meals with a bedtime snack, and micronutrient repletion can improve muscle mass, reduce complications, and enhance quality of life. Collaborate closely with a hepatology team and registered dietitian for ongoing support.

## C2-07

**Dietary and Nutritional Recommendations for HBV Cirrhosis with Ascites**

**Patient:** Male, 56 years, 170 cm, 66 kg (with ascites)

**1. Overall Dietary Principles**

- **Use "dry weight" for calculations**: Because ascites artificially elevates body weight, nutritional targets should be calculated using estimated dry weight (approximately 58-63 kg for this patient) rather than current measured weight
- **Prevent malnutrition and sarcopenia**: Cirrhosis accelerates muscle breakdown; adequate nutrition is therapeutic, not optional
- **Individualize recommendations**: Work with a registered dietitian specializing in liver disease to tailor plans to tolerance, preferences, and comorbidities
- **Avoid prolonged fasting**: The cirrhotic liver cannot store glycogen effectively, so extended fasting periods promote muscle catabolism

.

**2. Recommended Daily Protein Intake**

| **Parameter** | **Recommendation** | **Rationale** |
| --- | --- | --- |
| **Protein target** | **1.2–1.5 g/kg dry body weight/day** | Prevents muscle loss and supports hepatic regeneration |
| **Calculated for this patient** | ~70–95 g protein/day (using dry weight 58-63 kg) | Based on ESPEN/EASL guidelines |
| **Protein restriction?** | **Not recommended**, even with hepatic encephalopathy | Restriction worsens malnutrition without improving encephalopathy outcomes |

**Protein distribution strategy:**

- Spread intake across 4-6 eating occasions to optimize utilization
- Prioritize vegetable and dairy proteins over red meat when possible; these may be better tolerated and associated with lower mortality
- Include high-quality sources: eggs, fish, poultry, legumes, tofu, Greek yogurt, low-sodium cheese

**3. Meal Frequency and Eating Pattern**

| **Strategy** | **Practical Implementation** |
| --- | --- |
| **Eat little and often** | 3 main meals + 2-3 snacks every 2-3 hours |
| **Late evening snack (essential)** | 150-200 kcal carbohydrate + protein snack before bed (e.g., oatmeal with milk, rice pudding, whole-grain crackers with cheese) |
| **Avoid overnight fasting >10-12 hours** | Reduces muscle catabolism and improves nitrogen balance |
| **Coordinate with diuretic timing** | Take diuretics earlier in the day to minimize nocturia disrupting sleep and evening nutrition |

**4. Specific Food Recommendations and Restrictions**

**Encourage**

- **Protein sources**: Lentils, beans, eggs, fish (salmon, cod, sardines in water), skinless poultry, low-sodium dairy, tofu
- **Complex carbohydrates**: Oats, brown rice, whole-grain bread, potatoes (help meet energy needs)
- **Healthy fats**: Olive oil, avocado, nuts (in moderation for calorie density)
- **Flavor enhancers without salt**: Fresh herbs, garlic, lemon juice, vinegar, spices, mustard

**Limit or Avoid**

- **Sodium restriction**: Target **≤2,000 mg sodium/day** (≈5 g salt) for ascites management
  - Avoid processed meats, canned soups, salty snacks, soy sauce, pickled foods
  - Read labels: >1.5 g salt/100 g = high sodium
  - Check medications for hidden sodium content
- **Alcohol**: Complete abstinence is mandatory in HBV cirrhosis
- **Raw/undercooked shellfish**: Risk of Vibrio infection in cirrhosis
- **Excessive simple sugars**: May worsen insulin resistance common in cirrhosis

**Special Considerations**

- **If hepatic encephalopathy develops**: Continue adequate protein but favor vegetable/dairy sources; consider branched-chain amino acid (BCAA) supplementation if recommended by hepatology team
- **If diabetes/hyperglycemia present**: Prioritize low-glycemic carbohydrates and coordinate with diabetes management

**5. Additional Nutritional Considerations**

**Micronutrient Supplementation**

- **Vitamin D and calcium**: Screen for deficiency; supplement if osteoporosis risk exists
- **Zinc**: Deficiency is common and may worsen encephalopathy; consider supplementation if levels low
- **B-complex vitamins**: Especially thiamine if history of alcohol use or poor intake
- **Multivitamin**: A liver-specific formulation may be appropriate under medical supervision

**Monitoring and Follow-up**

- **Weight tracking**: Monitor trends using dry-weight estimates; sudden increases may reflect ascites progression rather than nutritional gain
- **Muscle assessment**: Handgrip strength or phase angle (if available) can help track sarcopenia risk
- **Laboratory monitoring**: Albumin, prealbumin, electrolytes (especially with diuretics), renal function

**When to Escalate Nutrition Support**

Consider oral nutritional supplements (ONS) if:

- Oral intake consistently <75% of targets for >3-5 days
- Unintentional weight loss >5% in 1 month
- Worsening sarcopenia on assessment

ONS should be high-protein, low-sodium formulations, ideally consumed as the late-evening snack

**Sample Daily Meal Framework (~2,000 kcal; 80 g protein; <2,000 mg sodium)**

| **Time** | **Meal** | **Example** |
| --- | --- | --- |
| 7 AM | Breakfast | Oatmeal made with milk + 1 egg + berries |
| 10 AM | Snack | Greek yogurt (low-sodium) + handful of unsalted almonds |
| 1 PM | Lunch | Grilled salmon + quinoa + steamed vegetables with olive oil & lemon |
| 4 PM | Snack | Whole-grain crackers + low-sodium cheese + apple |
| 7 PM | Dinner | Skinless chicken breast + brown rice + sautéed spinach with garlic |
| 9:30 PM | Late snack | Rice pudding made with milk or small bowl of cereal with milk |

## C2-08

**Dietary and Nutritional Recommendations for MASLD Cirrhosis with Ascites**

**Patient:** Female, 59 y/o, 155 cm, 80 kg (BMI 33.3 kg/m²), MASLD cirrhosis with ascites

**1. Overall Dietary Principles**

| **Principle** | **Rationale** |
| --- | --- |
| **Individualized, balanced nutrition** | Prevents malnutrition while managing ascites and metabolic dysfunction |
| **Prioritize protein adequacy** | Critical to prevent/treat sarcopenia, which independently predicts mortality in cirrhosis |
| **Sodium restriction** | Essential for ascites management; target <2,000 mg sodium/day |
| **Caloric moderation with quality focus** | Avoid excess calories while ensuring adequate intake to preserve muscle mass |
| **Mediterranean-style pattern** | Associated with improved outcomes in MASLD and cardiovascular risk reduction |

**Important**: Despite obesity, this patient is at high risk for *sarcopenic obesity*—loss of muscle mass with preserved or increased fat. Nutritional goals must prioritize **muscle preservation**, not weight loss alone.

**2. Recommended Daily Protein Intake**

| **Parameter** | **Recommendation** |
| --- | --- |
| **Target protein** | **1.2–1.5 g/kg of *ideal* body weight/day** |
| **Calculation** | Ideal body weight ≈ 48 kg → Target: **58–72 g protein/day** |
| **Distribution** | Evenly spread across 3 meals + evening snack (see below) |
| **Protein sources** | Prioritize high-biological-value proteins: fish, poultry, eggs, dairy, legumes, tofu |

**If hepatic encephalopathy develops**: Continue protein at target levels; consider plant-based or dairy proteins which may be better tolerated. Protein restriction is **not recommended** and may worsen outcomes

.

**3. Meal Frequency and Eating Pattern**

1

2

3

4

5

6

7

8

9

Recommended Daily Pattern:

┌─────────────────────────────────┐

│  Breakfast (7-8 AM)           │

│  Mid-morning snack (10 AM)   │

│ Lunch (12-1 PM)              │

│Afternoon snack (3-4 PM)     │

│  Dinner (6-7 PM)              │

│ EVENING SNACK (9-10 PM) ⭐  │

└─────────────────────────────────┘

**Key rationale:**

- **4–6 small meals/day** improves nutrient absorption and reduces catabolic periods
- **Evening snack is critical**: After an overnight fast, cirrhotic patients enter a catabolic state similar to prolonged starvation. A late-evening carbohydrate/protein snack (e.g., Greek yogurt + berries, or whole-grain crackers + cheese) improves nitrogen balance and reduces muscle breakdown

- Avoid fasting >12 hours between dinner and breakfast

**4. Specific Food Recommendations & Restrictions**

**Encourage**

| **Food Group** | **Examples** | **Notes** |
| --- | --- | --- |
| **Lean proteins** | Skinless poultry, fish (especially fatty fish 2x/week), eggs, Greek yogurt, cottage cheese, tofu, lentils | Aim for 20–30 g protein per main meal |
| **Complex carbohydrates** | Oats, quinoa, brown rice, whole-grain bread, sweet potatoes | Provide sustained energy; limit refined sugars |
| **Healthy fats** | Olive oil, avocado, nuts (small portions), fatty fish | Support metabolic health; avoid saturated/trans fats |
| **Vegetables & fruits** | All colors; prioritize non-starchy vegetables | Fiber supports gut health; monitor potassium if on diuretics |
| **Fluids** | Water, herbal teas | Generally unrestricted unless hyponatremia present |

**Restrict/Avoid**

| **Item** | **Limit/Reason** |
| --- | --- |
| **Sodium** | **<2,000 mg/day** (≈5 g salt). Avoid processed foods, canned soups, deli meats, soy sauce, restaurant meals |
| **Added sugars** | Limit to <25 g/day; avoid sugary beverages to support MASLD management |
| **Saturated/trans fats** | Limit red meat, fried foods, pastries to reduce hepatic fat accumulation |
| **Alcohol** | **Complete abstinence**—non-negotiable in any cirrhosis |
| **Raw shellfish** | Risk of Vibrio infection in immunocompromised cirrhotic patients |
| **Excessive fluid** | Only restrict if serum sodium <125–130 mmol/L (per physician guidance) |

**Practical Sodium-Reduction Tips**

- Cook at home using fresh ingredients; read labels carefully
- Flavor with herbs, spices, lemon, vinegar instead of salt
- Rinse canned beans/vegetables to reduce sodium by ~40%
- Choose "no salt added" or "low sodium" versions when available
- Limit cheese, bread, and condiments (major hidden sodium sources)

**5. Additional Nutritional Considerations**

**Micronutrient Supplementation**

Patients with cirrhosis are at high risk for deficiencies. Consider:

- **Vitamin D**: Deficiency common; supplement to maintain levels >30 ng/mL
- **Zinc**: Deficiency linked to hepatic encephalopathy; consider 25–50 mg elemental zinc/day if deficient
- **B-complex vitamins** (especially thiamine): Critical if any history of alcohol use or poor intake
- **Multivitamin**: Daily formulation without iron (unless documented deficiency)

**Weight Management in MASLD Cirrhosis**

- **Do NOT pursue aggressive weight loss** in decompensated cirrhosis
- If clinically stable with compensated disease, *gradual* weight loss (0.25–0.5 kg/week) via modest caloric deficit (~300–500 kcal/day) may improve hepatic steatosis, but **only under specialist supervision**
- Focus on body composition (preserving muscle) over scale weight

**Physical Activity**

- **Resistance exercise 2–3x/week** (as tolerated) synergizes with protein intake to combat sarcopenia

- Light walking daily supports metabolic health and functional status
- Consult physical therapy for individualized, safe exercise prescription

**Monitoring Parameters**

| **Parameter** | **Frequency** | **Target** |
| --- | --- | --- |
| Weight (dry weight) | Weekly | Stable; report rapid changes |
| Mid-arm muscle circumference | Monthly | Maintain or improve |
| Serum albumin, prealbumin | Every 3 months | Trend, not absolute value |
| Sodium, potassium, renal function | Per diuretic regimen | Within normal limits |
| Vitamin D, zinc levels | Annually or if symptomatic | Replete if deficient |

**Red Flags Requiring Prompt Re-evaluation**

- Unintentional weight loss >5% in 1 month
- Worsening edema/ascites despite sodium restriction
- Confusion, sleep disturbance (possible encephalopathy)
- Poor oral intake <50% of goals for >3 days

**Summary Prescription for This Patient**

 Daily Nutrition Goals:

• Calories: ~1,400–1,700 kcal/day (based on adjusted body weight ~56 kg × 25–30 kcal/kg)

• Protein: 60–70 g/day, evenly distributed

• Sodium: <2,000 mg/day

• Meals: 3 main + 2–3 snacks, including evening snack

• Pattern: Mediterranean-style, whole-food focused

• Supplements: Vitamin D 1000–2000 IU/day; consider zinc if deficient

• Activity: Daily walking + light resistance exercises as tolerated

• Follow-up: Dietitian visit in 2–4 weeks to assess adherence and adjust

**Key Message**: In cirrhosis, *adequate nutrition is therapeutic*. The goal is not caloric restriction for weight loss, but strategic nourishment to preserve muscle, manage ascites, and support liver function. Work closely with a hepatology dietitian for personalized implementation.

## C2-09

**Dietary and Nutritional Recommendations for Patient with Alcohol-Related Cirrhosis with Ascites and Edema**

**Patient Profile:** Male, 63 y/o, 167 cm, 60 kg (BMI 21.5 kg/m²), alcohol-related cirrhosis with ascites and peripheral edema

**1. Overall Dietary Principles**

- **Adequate energy intake:** Target 30-35 kcal/kg/day (approximately **1,800-2,100 kcal/day**) to prevent catabolism and muscle loss

- **Prioritize protein preservation:** Cirrhosis increases protein requirements due to impaired hepatic synthesis and accelerated muscle breakdown
- **Strict sodium restriction:** Limit to **<2,000 mg sodium/day** (approximately 5 g salt) to help control ascites and edema
- **Complete alcohol abstinence:** Essential to prevent further liver injury and improve nutritional outcomes .
- **Avoid prolonged fasting:** The cirrhotic liver has reduced glycogen stores; fasting accelerates muscle protein breakdown for gluconeogenesis

.

**2. Recommended Daily Protein Intake**

| **Parameter** | **Recommendation** |
| --- | --- |
| **Protein target** | **1.2-1.5 g/kg/day** = **72-90 g protein/day** for this patient |
| **If sarcopenia present** | Consider up to **1.5 g/kg/day** (90 g) or higher under dietitian supervision |
| **Protein distribution** | Spread evenly across meals/snacks; avoid >30g protein in a single sitting |
| **Protein quality** | Prioritize vegetable and dairy proteins; they are better tolerated and may reduce hepatic encephalopathy risk |

**Practical protein sources (≈7g protein per serving):**

- ✓ Eggs (1 large), Greek yogurt (¼ cup), cottage cheese (¼ cup)
- ✓ Fish (salmon, cod, tuna), poultry (chicken, turkey)
- ✓ Legumes (lentils, beans, chickpeas), tofu, tempeh, edamame
- ✓ Unsalted nuts/seeds (¼ cup), nut butters (2 Tbsp)
- ✓ Consider oral nutritional supplements if intake is inadequate

**3. Meal Frequency and Eating Pattern**

| **Strategy** | **Rationale & Implementation** |
| --- | --- |
| **Eat every 2-3 hours** | Prevents overnight catabolism; stabilizes blood glucose |
| **Include a late-evening snack** | 200-300 kcal with complex carbs + protein (e.g., cereal with milk, yogurt with fruit) before bedtime reduces muscle breakdown overnight |
| **Small, frequent meals** | Better tolerated with ascites-related early satiety; improves total intake |
| **Never skip meals** | Prolonged fasting worsens malnutrition and sarcopenia |

**Sample daily pattern:**

- Breakfast (7 AM) → Mid-morning snack (10 AM) → Lunch (1 PM) → Afternoon snack (4 PM) → Dinner (7 PM) → Bedtime snack (9-10 PM)

**4. Specific Food Recommendations & Restrictions**

**Encourage:**

- **High-protein, low-sodium foods:** Fresh poultry, fish, eggs, legumes, unsalted nuts, low-sodium dairy
- **Complex carbohydrates:** Whole grains, oats, rice, potatoes for sustained energy
- **Fruits and vegetables:** Rich in antioxidants, fiber, and micronutrients; choose fresh/frozen over canned (unless no-salt-added)
- **Healthy fats:** Olive oil, avocado, fatty fish (omega-3s) for anti-inflammatory benefits
- **Flavor enhancers without salt:** Herbs, spices, lemon juice, vinegar, garlic, ginger

**Limit or Avoid:**

- **High-sodium foods:** Processed meats (bacon, ham, deli meats), canned soups, salty snacks, soy sauce, bouillon, pickled foods, cheese with added salt
- **Added salt at table or during cooking**; use salt-free seasoning blends instead
- **Alcohol in any form**: Absolute contraindication
- **Raw shellfish**: Risk of Vibrio infection in cirrhosis
- **Excessive simple sugars**: May worsen insulin resistance common in cirrhosis

**Sodium Restriction Practical Tips:**

- Read labels: Choose foods with **<140 mg sodium per serving** or **<5% Daily Value**
- Rinse canned beans/vegetables to reduce sodium content
- Avoid "low-sodium" products that substitute potassium chloride if on potassium-sparing diuretics (e.g., spironolactone) – consult physician first
- Be aware that some medications (effervescent tablets, antacids) contain hidden sodium

**5. Additional Nutritional Considerations**

**Micronutrient Supplementation**

- **Thiamine (B1)**: Essential in alcohol-related liver disease; deficiency risk is high
- **Multivitamin without iron**: Iron overload can occur in cirrhosis; avoid iron unless deficiency confirmed
- **Vitamin D + Calcium**: Bone disease (osteoporosis) is common; supplement if levels are low or intake inadequate
- **Zinc**: Often deficient in cirrhosis; may support taste, immunity, and wound healing – consider testing/supplementation under guidance

**Monitoring & Follow-up**

- **Regular weight monitoring**: Track trends (not daily fluctuations); sudden increases may indicate worsening ascites
- **Muscle mass assessment**: Sarcopenia is common and predicts outcomes; consider handgrip strength or imaging if available
- **Hepatic encephalopathy screening**: If present, continue adequate protein but favor vegetable/dairy sources; avoid protein restriction
- **Referral to registered dietitian**: Individualized counseling improves adherence and outcomes

**When to Seek Immediate Guidance**

- Rapid weight gain (>1 kg/2-3 days) → possible ascites progression
- Confusion, drowsiness, or personality changes → possible hepatic encephalopathy
- Inability to meet >75% of protein/calorie goals for >3 days → consider oral supplements or enteral nutrition support

**Key Takeaway for This Patient**: With a normal BMI but decompensated cirrhosis (ascites/edema), the priority is **preserving muscle mass through adequate protein (72-90 g/day) and frequent feeding**, while **strictly limiting sodium (<2,000 mg/day)** to manage fluid retention. Complete alcohol cessation remains non-negotiable. A referral to a liver-specialized dietitian is strongly recommended for personalized meal planning and supplement guidance.

## C2-10

**Dietary and Nutritional Recommendations for Patient with HBV Cirrhosis and Ascites**

**Patient Profile:** Female, 67 years, 160 cm, 52 kg (BMI 20.3 kg/m²), HBV cirrhosis with ascites and poor appetite

**1. Overall Dietary Principles**

- **Prioritize adequate nutrition over restriction**: Malnutrition significantly worsens outcomes in cirrhosis; the goal is to prevent muscle loss and support liver function

.

- **Adequate energy intake**: Target **30-35 kcal/kg body weight/day** (approximately **1,560-1,820 kcal/day** for this patient) to prevent catabolism

.

- **Avoid prolonged fasting**: Cirrhotic patients deplete glycogen stores rapidly overnight; minimizing fasting periods helps preserve muscle mass

.

- **Individualize recommendations**: Adjust for comorbidities (e.g., diabetes, renal impairment) and tolerance, especially given poor appetite

.

- **Focus on nutrient density**: Given reduced appetite, prioritize foods that deliver maximum nutrition per volume consumed.

**2. Recommended Daily Protein Intake**

| **Parameter** | **Recommendation** | **Rationale** |
| --- | --- | --- |
| **Protein target** | **1.2-1.5 g/kg/day** = **62-78 g/day** | Prevents sarcopenia and supports immune function; protein restriction worsens outcomes |
| **Protein source preference** | Emphasize **vegetable/plant-based proteins** (legumes, tofu, lentils) and dairy; include lean animal proteins as tolerated | Vegetable proteins have lower aromatic amino acids and higher fiber, potentially reducing ammonia production and hepatic encephalopathy risk |
| **Distribution** | Spread protein evenly across meals (≈15-25 g per eating occasion) | Optimizes muscle protein synthesis throughout the day |

**Important**: Do NOT restrict protein even if mild hepatic encephalopathy is present. Evidence shows protein restriction worsens malnutrition, sarcopenia, and may paradoxically increase ammonia levels

领英企业服务

.

**3. Meal Frequency and Eating Pattern**

| **Strategy** | **Practical Implementation** | **Evidence Basis** |
| --- | --- | --- |
| **Small, frequent meals** | 5-6 eating occasions/day (3 main meals + 2-3 snacks), spaced every 3-4 hours while awake | Reduces catabolic periods and improves nutrient absorption in patients with early satiety from ascites |
| **Late-evening snack (LES)** | 150-200 kcal carbohydrate + protein snack within 1 hour of bedtime (e.g., Greek yogurt with berries, small bowl of oatmeal with nuts) | Shortens overnight fasting, improves nitrogen balance, and may reduce ascites/encephalopathy risk |
| **Eat when appetite is best** | Encourage larger intake during times of day when hunger is strongest (often morning) | Accommodates cirrhosis-related anorexia and gastroparesis |
| **Avoid lying down after meals** | Remain upright 30-60 minutes post-meal | Reduces reflux and discomfort from ascites-related abdominal pressure |

**4. Specific Food Recommendations and Restrictions**

**Encourage**

| **Food Group** | **Examples** | **Rationale** |
| --- | --- | --- |
| **High-quality proteins** | Eggs, Greek yogurt, cottage cheese, fish, skinless poultry, tofu, lentils, chickpeas | Meet protein targets while providing essential amino acids  liver.ca |
| **Complex carbohydrates** | Oats, brown rice, whole-grain bread, sweet potatoes | Provide sustained energy; fiber supports gut health |
| **Healthy fats** | Olive oil, avocado, nuts (if no dental issues), fatty fish (salmon) | Concentrated calories for patients with poor appetite; omega-3s may have anti-inflammatory benefits |
| **Fruits and vegetables** | Cooked vegetables (easier to digest), bananas, applesauce, berries | Provide micronutrients, fiber, and antioxidants; cook vegetables to reduce volume if early satiety is problematic |
| **Oral nutritional supplements (ONS)** | High-protein, low-sodium supplements (e.g., 1-2 servings/day between meals) if oral intake inadequate | Evidence supports ONS to improve survival when dietary goals aren't met orally |

**Limit or Avoid**

| **Item** | **Restriction Level** | **Rationale** |
| --- | --- | --- |
| **Sodium** | **≤2,000 mg/day** (≈5 g salt) | First-line management for ascites; reduces fluid retention |
| **Processed/high-sodium foods** | Avoid canned soups, deli meats, pickled foods, soy sauce, fast food | Major hidden sodium sources; read labels carefully |
| **Alcohol** | **Complete abstinence** | Critical in HBV cirrhosis to prevent further liver injury |
| **Raw shellfish** | Avoid oysters, clams, mussels | Risk of Vibrio infection, which can be fatal in cirrhosis |
| **Excess simple sugars** | Limit sugary drinks, candies | May worsen insulin resistance; prioritize nutrient-dense calories |
| **Large fluid volumes with meals** | Sip fluids between meals rather than drinking large amounts during meals | May worsen early satiety from ascites |

**Practical Sodium Reduction Tips**

- Use herbs, lemon juice, garlic, vinegar, and salt-free spice blends for flavor
- Rinse canned beans/vegetables to reduce sodium by ~40%
- Choose "no salt added" or "low sodium" labeled products
- Prepare meals at home to control sodium content
- Limit restaurant meals where sodium content is often uncontrolled

**5. Additional Nutritional Considerations**

**Micronutrient Supplementation**

Patients with cirrhosis are at high risk for deficiencies. Consider:

- **Multivitamin**: Daily supplementation with a liver-safe formulation (avoid excess iron and vitamin A)
- **Vitamin D**: Supplement if levels <30 ng/mL (common deficiency; supports bone health)
- **Zinc**: 25-50 mg elemental zinc/day if deficient or if taste changes present; may improve appetite and ammonia metabolism
- **Thiamine (B1)**: Especially important if any history of alcohol use; prevents Wernicke's encephalopathy

**Monitoring and Follow-up**

- **Weight**: Monitor weekly (dry weight if possible); unexpected loss may indicate worsening malnutrition
- **Muscle function**: Simple handgrip strength or chair-stand tests can track sarcopenia progression
- **Laboratory**: Periodic assessment of albumin, prealbumin, electrolytes, zinc, and vitamin D levels
- **Appetite support**: If poor appetite persists despite dietary strategies, discuss with hepatology team about possible pharmacologic appetite stimulants or referral to dietitian for personalized ONS planning

**When to Escalate Nutrition Support**

Consider referral for enteral nutrition evaluation if:

- Oral intake consistently <60% of estimated needs for >3-5 days
- Unintentional weight loss >5% in 1 month or >10% in 6 months
- Worsening sarcopenia or functional decline despite oral interventions

**Sample Daily Meal Plan (≈1,700 kcal, 70 g protein, <2,000 mg sodium)**

| **Time** | **Meal** | **Approx. Nutrition** |
| --- | --- | --- |
| 7:30 AM | Scrambled eggs (2) with spinach + 1 slice whole-grain toast + ½ cup berries | 350 kcal, 22 g protein, 400 mg sodium |
| 10:00 AM | Greek yogurt (¾ cup) with 1 tbsp chopped walnuts | 200 kcal, 15 g protein, 60 mg sodium |
| 12:30 PM | Grilled chicken breast (3 oz) + ½ cup brown rice + steamed carrots/zucchini with olive oil | 450 kcal, 28 g protein, 300 mg sodium |
| 3:30 PM | Small apple + 1 string cheese | 180 kcal, 8 g protein, 200 mg sodium |
| 6:30 PM | Baked salmon (3 oz) + mashed sweet potato (½ cup) + green beans | 400 kcal, 25 g protein, 250 mg sodium |
| 9:00 PM (LES) | Oatmeal (½ cup dry) made with milk + cinnamon | 220 kcal, 10 g protein, 80 mg sodium |
| **TOTAL** |  | **~1,800 kcal, ~108 g protein, ~1,290 mg sodium** |

*Note: Protein exceeds minimum target to account for potential incomplete absorption; sodium remains well below limit to allow flexibility.*

## C2-11

**Dietary and Nutritional Recommendations for MASLD Cirrhosis with Recurrent Ascites**

**Patient Profile:** 71-year-old male, 169 cm, 78 kg (BMI 27.3 kg/m²), MASLD cirrhosis with recurrent ascites

**1. Overall Dietary Principles**

- **Prioritize adequate nutrition while managing complications:** The primary goals are to prevent malnutrition and sarcopenia (muscle loss), which are common in cirrhosis and worsen outcomes, while simultaneously managing ascites through sodium restriction

.

- **Adopt a Mediterranean-style dietary pattern:** This is recommended for MASLD management, emphasizing vegetables, fruits, whole grains, legumes, fish, and healthy fats while limiting processed foods, added sugars, and saturated fats

.

- **Individualize recommendations:** Work closely with a registered dietitian specializing in liver disease to tailor these general principles to your specific needs, preferences, and tolerance

.

- **Avoid alcohol completely:** Any alcohol consumption can accelerate liver damage in cirrhosis

.

**2. Recommended Daily Protein Intake**

- **Target: 1.2–1.5 g/kg body weight/day = approximately 95–115 g protein daily**

.

- **Rationale:** Adequate protein is critical to preserve muscle mass and reverse sarcopenia, which is strongly associated with poor outcomes in cirrhosis. Protein restriction is **not recommended**, even in patients with hepatic encephalopathy

.

- **Protein distribution strategies:**
  - Spread protein intake evenly across meals and snacks rather than consuming most at one meal
  - Prioritize vegetable and dairy protein sources (beans, lentils, tofu, eggs, Greek yogurt) over red meat, as these may be better tolerated and associated with lower mortality
  - Include a protein-containing bedtime snack to reduce overnight catabolism

**3. Meal Frequency and Eating Pattern**

- **Eat small, frequent meals:** Aim for 3 main meals plus 2–3 snacks, eating every 2–3 hours while awake

.

- **Include a late-evening snack:** A bedtime snack containing complex carbohydrates and protein (e.g., Greek yogurt with berries, cottage cheese with crackers) helps prevent prolonged fasting and muscle breakdown

.

- **Rationale:** Cirrhosis impairs glycogen storage, making patients prone to catabolism during fasting periods. Frequent feeding helps maintain energy balance and muscle mass

.

- **Practical tip:** If early satiety or abdominal discomfort from ascites limits meal size, focus on nutrient-dense, smaller portions and use oral nutritional supplements if recommended by your dietitian

.

**4. Specific Food Recommendations and Restrictions**

**Emphasize:**

| **Food Group** | **Examples** | **Rationale** |
| --- | --- | --- |
| **Lean proteins** | Fish (salmon, cod), poultry, eggs, Greek yogurt, lentils, beans, tofu | Support muscle maintenance; fish provides anti-inflammatory omega-3s |
| **Non-starchy vegetables** | Spinach, broccoli, carrots, asparagus (≥3 servings/day) | High in fiber, antioxidants, low in calories |
| **Whole fruits** | Berries, apples, pears (2 servings/day; fresh/frozen, not juice) | Provide vitamins and fiber; avoid high-sugar juices |
| **Whole grains** | Oats, brown rice, quinoa, whole-wheat bread | Support stable blood sugar and provide sustained energy |
| **Healthy fats** | Olive oil, avocado, unsalted nuts (walnuts, almonds) | Anti-inflammatory; replace saturated fats |
| **Fluids** | Water, herbal tea, black coffee (if tolerated) | Maintain hydration; coffee may have liver-protective properties |

**Limit or Avoid:**

| **Item** | **Reason** |
| --- | --- |
| **High-sodium foods** | Sodium restriction (≤2,000 mg/day) is essential to manage ascites and fluid retention |
| **Processed meats** | Ham, bacon, sausages, deli meats are typically very high in sodium |
| **Canned foods with added salt** | Choose "no salt added" versions or rinse thoroughly |
| **Restaurant/fast foods** | Often extremely high in sodium and unhealthy fats |
| **Added sugars & refined carbs** | Sodas, sweets, white bread worsen metabolic dysfunction in MASLD |
| **Saturated/trans fats** | Fatty meats, fried foods, butter; replace with unsaturated fats |
| **Raw/undercooked shellfish or meat** | Increased risk of serious infection in cirrhosis |
| **Alcohol** | Absolutely contraindicated in all forms |

**Sodium Restriction Practical Tips:**

- Target: **≤2,000 mg sodium per day** (approximately 5 g salt)
- Read labels: Avoid products with >1.5 g salt (0.6 g sodium) per 100 g
- Flavor alternatives: Use herbs, spices, lemon juice, vinegar, garlic, ginger instead of salt
- Check medications: Some over-the-counter and prescription drugs contain significant sodium; review with your pharmacist

**5. Additional Nutritional Considerations**

**Micronutrient Monitoring**

Patients with cirrhosis are at risk for deficiencies in:

- **Vitamin D and calcium:** Screen for bone health; supplementation often needed
- **Zinc:** Deficiency is common and may worsen taste changes and encephalopathy; consider supplementation if deficient
- **B vitamins (especially thiamine):** Important for energy metabolism; assess status

**Weight Management**

- Given BMI 27.3 kg/m² (overweight), **gradual weight loss of 5–10%** may improve MASLD progression, but must be balanced with preserving muscle mass

.

- **Do not pursue aggressive calorie restriction.** Focus on improving diet quality (Mediterranean pattern) and increasing physical activity as tolerated, while maintaining adequate protein intake to protect muscle

.

**Ascites-Specific Considerations**

- Monitor daily weight and abdominal girth; report sudden increases to your care team
- Fluid restriction is **not routinely recommended** unless serum sodium is <126 mmol/L
- Diuretic therapy (if prescribed) should be coordinated with dietary sodium management

**When to Seek Specialized Support**

- Referral to a **liver-specialized dietitian** is strongly recommended for personalized meal planning, supplement guidance, and monitoring

liver.ca

- Consider oral nutritional supplements (high-protein, low-sodium) if oral intake is inadequate
- If hepatic encephalopathy develops, work with your team to optimize protein sources (favoring vegetable/dairy) without reducing total protein

## C2-12

**Dietary & Nutritional Recommendations for Cirrhosis with Ascites**

**Patient**: 74-year-old female, 156 cm, 64 kg (BMI 26.3 kg/m²), alcohol-related cirrhosis with ascites

**1. Overall Dietary Principles**

| **Principle** | **Rationale** |
| --- | --- |
| **Adequate energy intake**: Target ~30-35 kcal/kg ideal body weight/day (~1,800-2,100 kcal) | Prevents muscle catabolism; cirrhosis increases resting energy expenditure |
| **Prioritize protein adequacy**: Do NOT restrict protein, even with history of hepatic encephalopathy | Protein restriction worsens sarcopenia and increases mortality |
| **Sodium moderation**: Limit to <2 g sodium/day (~5 g salt or 60 mmol) for ascites control | Reduces fluid retention while balancing palatability and intake |
| **Complete alcohol abstinence**: Zero alcohol intake | Prevents further hepatocyte injury and disease progression |
| **Focus on food quality**: Emphasize nutrient-dense, easily digestible foods | Compensates for reduced intake capacity due to early satiety from ascites |

**2. Recommended Daily Protein Intake**

**Target: 1.2–1.5 g/kg ideal body weight/day**

- For this patient (ideal body weight ~52 kg for 156 cm female):
  - **Minimum**: ~62 g protein/day
  - Optimal (if sarcopenic or malnourished)

**Practical protein distribution**:

| **Meal** | **Example Protein Source** | **Approx. Protein** |
| --- | --- | --- |
| Breakfast | 1 egg + ½ cup Greek yogurt | ~15 g |
| Mid-morning snack | 1 oz cheese or small handful nuts | ~7 g |
| Lunch | 3 oz cooked fish/chicken or ¾ cup lentils | ~20-25 g |
| Afternoon snack | Hummus with crackers or protein supplement | ~8-10 g |
| Dinner | 3 oz lean meat, tofu, or eggs | ~20 g |
| **Late evening snack** | Casein-based supplement, cottage cheese, or milk | ~10-15 g |

**Key**: Spread protein evenly across meals; include a **late evening snack** to reduce overnight catabolism

**3. Meal Frequency & Eating Pattern**

**Recommended pattern: 4–6 small meals/snacks per day**, including a late-evening snack

Sample Daily Schedule:

• 7:00 AM  – Breakfast

• 10:00 AM – Small snack

• 1:00 PM  – Lunch

• 4:00 PM  – Afternoon snack

• 7:00 PM  – Dinner

• 9:30 PM  – Late evening snack (essential)

**Why this matters**:

- Fasting periods >3–4 hours accelerate muscle breakdown in cirrhosis
- Late-evening carbohydrate/protein snack improves nitrogen balance and total body protein status
- Small, frequent meals accommodate early satiety from ascites

**4. Specific Food Recommendations & Restrictions**

**Encourage:**

| **Food Group** | **Examples** | **Notes** |
| --- | --- | --- |
| **Lean proteins** | Fish, poultry, eggs, Greek yogurt, cottage cheese, tofu, lentils | Prioritize vegetable/dairy proteins if encephalopathy history |
| **Complex carbohydrates** | Oats, brown rice, whole-grain bread, sweet potatoes | Provide sustained energy; include in evening snack |
| **Healthy fats** | Olive oil, avocado, nuts (unsalted), fatty fish (salmon) | Support calorie density without sodium |
| **Fruits & vegetables** | Fresh/frozen (low-sodium preparation), cooked if chewing difficulty | Provide fiber, antioxidants, micronutrients |
| **Fluids** | Water, herbal teas; monitor total fluid if hyponatremic | Follow physician guidance on fluid restriction if prescribed |

**Limit/Avoid:**

| **Item** | **Reason** |
| --- | --- |
| **Added salt & high-sodium foods** | Processed meats, canned soups, salty snacks, soy sauce, restaurant meals |
| Alcohol (any amount) | Accelerates liver damage; absolute contraindication |
| **Raw shellfish** | Risk of Vibrio infection in immunocompromised cirrhotic patients |
| **Excess simple sugars** | May worsen insulin resistance; limit sugary beverages/desserts |
| **Unsupervised herbal supplements** | Many are hepatotoxic (e.g., kava, comfrey, high-dose green tea extract) |

**Sodium reduction tips**:

- Use herbs, lemon, garlic, vinegar instead of salt for flavor
- Rinse canned beans/vegetables to remove ~40% of sodium
- Read labels: choose items with <140 mg sodium/serving when possible
- Prepare meals at home to control sodium content

**5. Additional Nutritional Considerations**

**Micronutrient Supplementation (High Priority in Alcohol-Related Cirrhosis)**

| **Nutrient** | **Rationale** | **Typical Supplementation*** |
| --- | --- | --- |
| Thiamine (B1) | Alcohol use depletes stores; deficiency risks Wernicke's | 100 mg/day orally |
| **Vitamin D** | Deficiency common; linked to sarcopenia & poor outcomes | 800–2000 IU/day; check 25-OH-D level |
| **Zinc** | Deficiency contributes to dysgeusia, poor intake, encephalopathy risk | 25–50 mg elemental zinc/day if deficient |
| **Magnesium** | Diuretic use + malabsorption increase deficiency risk | 200–400 mg/day if low serum level |
| Multivitamin (liver-specific) | Broad coverage for water-soluble vitamin deficits | Daily liver-formulation supplement |

**Confirm doses with treating physician; adjust for renal function*

**Monitoring & Follow-Up**

- **Weight**: Track weekly (dry weight); sudden increases may signal worsening ascites
- **Muscle function**: Simple handgrip strength or chair-stand tests to screen for sarcopenia
- **Lab monitoring**: Albumin, prealbumin (contextual), electrolytes, vitamin D, zinc levels every 3–6 months
- **Dietitian referral**: Strongly recommended for personalized meal planning and adherence support

**Special Considerations for This Patient**

1. **Age-related factors**: Reduced appetite, dental issues, or swallowing difficulties may require texture-modified foods or oral nutritional supplements
2. **Ascites management**: If sodium restriction reduces food intake, discuss *liberalizing sodium slightly* with the hepatology team to preserve overall nutrition
3. **Medication-nutrient interactions**: Diuretics (spironolactone/furosemide) may alter potassium/sodium balance—coordinate dietary advice with medication regimen
4. **Cognitive support**: If mild encephalopathy present, use simple written meal plans, involve caregiver in food preparation

**Quick Reference: Daily Goals for This Patient**

| **Parameter** | **Target** |
| --- | --- |
| **Energy** | 1,800–2,100 kcal/day |
| **Protein** | 65–80 g/day (spread across 5–6 eating occasions) |
| **Sodium** | <2,000 mg/day |
| **Fluids** | As directed by physician (typically 1.5–2 L unless restricted) |
| **Meals** | 3 main meals + 2–3 snacks, including bedtime snack |
| **Alcohol** | Zero |
| **Supplements** | Multivitamin + thiamine + vitamin D ± zinc (per labs) |

**Important**: These recommendations should be integrated with the patient's overall medical management. Coordinate closely with her hepatologist, especially regarding diuretic therapy, encephalopathy prophylaxis, and transplant evaluation if indicated.

## C3-01

**Dietary and Nutritional Recommendations for a Patient with Cirrhosis and History of Hepatic Encephalopathy**

As a physician specializing in clinical nutrition, I have reviewed the case of your 35-year-old male patient with cirrhosis (height: 171 cm, weight: 65 kg, BMI: 22.2, which is within the normal range) and a history of hepatic encephalopathy (HE) that has resolved. The primary goals for dietary management in this context are to:

- Prevent recurrence of hepatic encephalopathy by minimizing ammonia production.
- Support liver function and prevent muscle wasting (sarcopenia), which is common in cirrhosis and worsens prognosis.
- Address complications of cirrhosis, such as fluid retention or malnutrition, while ensuring adequate energy and nutrient intake.

**1. Overall Dietary Principles**

- Prioritize adequate caloric intake: Aim for 25–35 kcal per kg of ideal body weight per day (approximately 1,625–2,275 kcal/day for this patient, based on an ideal weight of 65 kg). This prevents catabolism (muscle breakdown), which can trigger HE recurrence. Undernutrition is a key risk factor for HE.
- Moderate protein intake: Protein is essential for healing but must be carefully balanced to avoid excess ammonia production. Focus on high-quality, low-ammonia-producing sources (e.g., plant-based and dairy proteins).
- Strict sodium restriction: Limit sodium to <2,000 mg (2 g) per day to reduce the risk of ascites or edema. Avoid adding salt during cooking or at the table.
- Alcohol avoidance: Complete abstinence is non-negotiable, as alcohol accelerates liver damage.
- Fiber emphasis: Include soluble fiber to promote regular bowel movements (critical for HE prevention, as constipation increases ammonia absorption).
- Avoid fasting: Prolonged gaps between meals can cause muscle breakdown; ensure consistent energy intake.

**2. Recommended Daily Protein Intake**

- Target range: 1.0–1.2 g per kg of ideal body weight per day (65–78 g/day for this patient, as his current weight of 65 kg aligns closely with ideal body weight for his height).
  - *Rationale*: Higher protein intake (>1.5 g/kg/day) may increase ammonia risk in HE-prone patients, while lower intake (<0.8 g/kg/day) can worsen sarcopenia. This range balances safety and muscle preservation.
- Source distribution:
  - 60–70% from plant-based or dairy proteins (e.g., lentils, tofu, Greek yogurt), which produce less ammonia than animal proteins.
  - Limit animal proteins to 30–40% (e.g., skinless poultry, egg whites, or fatty fish like salmon 2–3 times/week). Avoid red meat, pork, and organ meats, as they are high in ammonia precursors.
- Monitor closely: If HE symptoms recur (e.g., confusion, fatigue), reduce protein temporarily to 0.8 g/kg/day and consult a specialist. Never eliminate protein entirely.

**3. Meal Frequency and Eating Pattern**

- Eat 4–6 small meals/snacks daily, spaced every 3–4 hours (e.g., breakfast, mid-morning snack, lunch, afternoon snack, dinner, and a bedtime snack).
  - *Why?* This prevents large protein loads in single meals, which can overwhelm the liver’s detoxification capacity and trigger HE. It also stabilizes blood glucose and reduces catabolism.
- Include protein in every meal/snack (e.g., 10–15 g per serving) to distribute intake evenly. Example pattern:
  - Breakfast: Oatmeal with almond milk + 1 scrambled egg white.
  - Mid-morning: Apple with 2 tbsp peanut butter.
  - Lunch: Lentil soup + side salad (low-sodium dressing).
  - Afternoon: Greek yogurt with berries.
  - Dinner: Baked salmon (4 oz) + quinoa + steamed broccoli.
  - Bedtime snack (essential): Casein-rich food like cottage cheese (½ cup) or a small protein shake. This provides slow-digesting protein overnight to prevent muscle breakdown.
- Avoid skipping meals, especially dinner-to-breakfast gaps >12 hours.

**4. Specific Food Recommendations or Restrictions**

- Recommended foods:
  - Proteins: Tofu, tempeh, lentils, chickpeas, egg whites, low-fat Greek yogurt, cottage cheese, and fatty fish (salmon, mackerel 2x/week).
  - Carbohydrates: Whole grains (oats, quinoa, brown rice), fruits (apples, pears, berries), and non-starchy vegetables (broccoli, spinach, carrots). These provide fiber and antioxidants.
  - Fats: Unsaturated fats only—avocado, olive oil, nuts (almonds, walnuts in moderation).
  - Fluids: Water, herbal teas; limit caffeine to 1–2 cups/day.
- Strict restrictions:
  - Sodium: Avoid processed foods (canned soups, deli meats, frozen meals), salty snacks (chips, pretzels), and high-sodium sauces (soy sauce, ketchup). Use herbs/spices (e.g., garlic, lemon, turmeric) for flavor.
  - Ammonia-promoting foods: Red meat, pork, organ meats (liver), and excessive eggs (>2 whole eggs/day).
  - Alcohol and sugary drinks: Zero tolerance for alcohol; limit added sugars (e.g., soda, candy) to avoid fatty liver progression.
  - High-mercury fish: Avoid swordfish or tuna; opt for low-mercury options like salmon.
- Special note for HE: Prioritize foods with prebiotic fiber (e.g., oats, bananas, asparagus) to support gut health and reduce ammonia production.

**5. Additional Nutritional Considerations**

- Vitamin and mineral supplementation:
  - Cirrhosis often causes deficiencies in vitamins B1 (thiamine), B9 (folate), B12, D, and K due to malabsorption or reduced synthesis. Recommend:
    - A daily multivitamin without iron (iron can accumulate in cirrhosis).
    - Vitamin D (800–1,000 IU/day) and vitamin K (100 mcg/day) if coagulopathy is present (common in cirrhosis).
    - Avoid high-dose antioxidants (e.g., vitamin E) without specialist approval, as they may be harmful.
- Bowel regularity: Constipation is a major HE trigger. Aim for 25–30 g of fiber daily from foods (e.g., chia seeds, flaxseeds, vegetables). If needed, use a gentle fiber supplement (e.g., psyllium husk) or lactulose (if prescribed) to ensure 1–2 soft bowel movements/day.
- Hydration and fluid balance: Drink 1.5–2 L of water daily unless ascites is severe (then follow physician-guided limits). Avoid excessive fluids at once.
- Monitoring and follow-up:
  - Weigh weekly to detect fluid retention (sudden gain >1 kg/week may indicate ascites).
  - Schedule quarterly nutrition visits to adjust protein/calorie goals based on liver function tests (e.g., MELD score) and muscle mass (via handgrip strength or calf circumference).
  - If sarcopenia develops, increase protein to 1.5 g/kg/day under supervision, emphasizing branched-chain amino acids (BCAAs) from sources like whey protein isolate.
- Lifestyle integration: Encourage light exercise (e.g., 30-min daily walking) to preserve muscle, but avoid intense activity that could cause fatigue.

**Key Reminder for the Patient**

This plan is **preventive and dynamic**. Hepatic encephalopathy can recur silently, so emphasize:

- No self-adjustments: Never restrict protein below 0.8 g/kg/day without medical guidance.
- Symptom awareness: Report early HE signs (e.g., sleep disturbances, mood changes) immediately.
- Collaborative care: Work with a registered dietitian specializing in liver disease for personalized meal planning. Studies show structured dietary intervention reduces HE recurrence by up to 50% (source: *Journal of Hepatology*, 2023).

If you have further clinical details (e.g., current MELD score, ascites status), I can refine these recommendations. Always consult the patient’s hepatologist before implementation.

## C3-02

Dietary and Nutritional Recommendations for a Patient with Alcohol-Related Cirrhosis and Prior Hepatic Encephalopathy

As a physician specializing in clinical nutrition, I have reviewed the case of this 38-year-old female patient (height: 158 cm, weight: 53 kg, BMI: 21.2 kg/m², within normal range) with alcohol-related cirrhosis and a history of hepatic encephalopathy (HE).

1. Overall Dietary Principles

Prioritize adequate energy intake to prevent muscle catabolism (which can worsen HE). Target 25–35 kcal/kg of ideal body weight per day (approximately 1,325–1,855 kcal/day for this patient, based on ideal weight for height). Undernutrition accelerates HE risk, so consistent caloric sufficiency is critical.

Moderate, balanced protein intake—neither too low (to avoid sarcopenia) nor too high (to minimize ammonia production). Focus on plant-based and dairy proteins, which generate less ammonia than animal proteins.

Strict sodium restriction if ascites or edema is present (common in cirrhosis); aim for <2 g (2,000 mg) sodium/day. If no fluid complications, limit to ≤3 g/day.

Alcohol abstinence is mandatory—zero tolerance, as even small amounts can trigger HE or accelerate liver damage.

Emphasize dietary fiber (25–30 g/day) to promote bowel regularity and reduce ammonia absorption via gut microbiota modulation.

2. Recommended Daily Protein Intake

Target: 1.2–1.5 g of protein per kg of actual body weight per day (64–80 g/day for this 53 kg patient). Given the history of HE, start at the lower end (1.2 g/kg = ~64 g/day) and gradually increase to 1.5 g/kg if tolerated, to build/maintain muscle mass without provoking HE.

Rationale: Very low protein (<0.8 g/kg) increases mortality risk from sarcopenia, while excessive protein (>1.8 g/kg) may elevate blood ammonia. Plant-based proteins (e.g., legumes, tofu) and dairy (e.g., yogurt) are preferred, as they produce fewer ammonia precursors than red meat.

Monitor closely: If HE symptoms recur (e.g., confusion, drowsiness), temporarily reduce protein to 0.8 g/kg for 2–3 days, then reintroduce slowly. Regular blood ammonia and nutrition assessments are advised.

3. Meal Frequency and Eating Pattern

Adopt 4–6 small meals/snacks daily, spaced evenly (e.g., every 3–4 hours), to avoid large protein loads that spike ammonia levels.

Example schedule: Breakfast, mid-morning snack, lunch, afternoon snack, dinner, and a bedtime snack (e.g., 150 g low-fat yogurt + 1 small banana). The bedtime snack is crucial—it provides sustained energy overnight, reducing catabolism and HE risk during fasting periods.

Distribute protein evenly: Aim for 15–20 g protein per meal/snack (e.g., 15 g at breakfast, 20 g at lunch). Avoid concentrating >30 g protein in one sitting.

Carbohydrates as the primary energy source: 60–70% of calories should come from complex carbs (e.g., oats, whole grains) to spare protein for tissue repair. Include 30–45 g carbs in the bedtime snack to stabilize overnight glucose.

4. Specific Food Recommendations or Restrictions

Recommended foods:

Proteins: Plant-based options (e.g., lentils, chickpeas, tofu—½ cup cooked = 7–10 g protein), low-fat dairy (e.g., Greek yogurt—170 g = 15 g protein), and lean poultry/fish (e.g., 85 g baked chicken = 25 g protein; limit to 1–2 servings/day).

Carbohydrates: Whole grains (oats, brown rice), fruits (berries, apples), and vegetables (broccoli, spinach)—prioritize high-fiber varieties for gut health.

Fats: Healthy sources only (e.g., 1 tbsp olive oil, avocado, nuts—small portions to avoid steatorrhea).

Fluids: Water, herbal teas; limit caffeine.

Critical restrictions:

Avoid high-ammonia animal proteins: Red meat, processed meats (bacon, sausages), and organ meats (liver), as they increase HE risk.

Strictly limit sodium: No processed foods (canned soups, chips), added salt, or salty condiments. Use herbs/spices for flavor. Check labels for "sodium-free" options.

Exclude alcohol entirely—no exceptions.

Avoid raw/undercooked seafood (risk of Vibrio infection in cirrhosis) and excessive branched-chain amino acid (BCAA) supplements without medical supervision.

Note: If constipation occurs (common in HE), increase fiber gradually and ensure adequate fluid intake; consider a fiber supplement like psyllium (5–10 g/day).

5. Additional Nutritional Considerations

Vitamin and mineral supplementation:

B-complex vitamins (especially B1, B6, folate) due to alcohol-related deficiencies; prescribe a daily multivitamin with B-complex.

Vitamin D (800–1,000 IU/day) and zinc (15–25 mg/day), as deficiencies are prevalent in cirrhosis and linked to HE.

Avoid high-dose vitamin A (risk of hepatotoxicity).

Gut health focus: Consider probiotics (e.g., Lactobacillus-based supplements) or prebiotic foods (bananas, oats) to reduce ammonia-producing bacteria. If HE recurs frequently, discuss rifaximin (a non-absorbable antibiotic) with the hepatologist, as it complements dietary management.

Practical monitoring:

Weigh weekly to track fluid status (sudden gain >1 kg/week suggests ascites progression).

Use a food diary to log protein intake, HE symptoms, and bowel movements.

Schedule follow-ups every 3–6 months for nutrition assessment (e.g., mid-arm circumference for muscle mass).

Patient education: Teach recognition of early HE signs (e.g., forgetfulness, personality changes) and stress that consistent eating patterns are as vital as medication. Refer to a dietitian for personalized meal planning and alcohol counseling support.

These recommendations balance HE prevention with nutritional rehabilitation. Always individualize based on lab results (e.g., serum albumin, ammonia levels) and comorbidities. If symptoms worsen, seek immediate medical evaluation.

## C3-03

**Dietary and Nutritional Recommendations for a Patient with MASLD Cirrhosis and Prior Hepatic Encephalopathy**

As a physician specializing in clinical nutrition, I have reviewed the case of this 43-year-old male patient (height: 169 cm, weight: 80 kg; BMI: 28.0, indicating overweight) with metabolic dysfunction-associated steatotic liver disease (MASLD) cirrhosis and a history of hepatic encephalopathy (HE). The primary goals of dietary management are to:

- Prevent malnutrition (common in cirrhosis due to increased metabolic demands and muscle wasting).
- Minimize triggers for hepatic encephalopathy recurrence (e.g., excessive protein breakdown, constipation, or electrolyte imbalances).
- Address metabolic comorbidities (e.g., obesity-related insulin resistance from MASLD).
- Reduce complications (e.g., ascites from sodium retention).

All advice prioritizes safety, feasibility, and individualization—**close monitoring by a hepatology team is essential**, with adjustments based on tolerance, lab results (e.g., ammonia levels, albumin), and HE symptoms.

**1. Overall Dietary Principles**

- High-calorie intake: Aim for 35–40 kcal/kg of actual body weight per day (≈2,800–3,200 kcal/day for this 80 kg patient) to prevent catabolism and muscle loss. Prioritize complex carbohydrates (e.g., whole grains) for 50–60% of calories, as they provide steady energy and reduce reliance on protein breakdown.
- Moderate, high-quality protein: Protein must be carefully titrated to avoid both deficiency (worsening sarcopenia) and excess (triggering HE). Focus on easily digestible, plant-based, and dairy-derived proteins (lower in aromatic amino acids, which may exacerbate HE).
- Strict sodium restriction: Limit to <2,000 mg/day (≈5 g salt) to prevent fluid retention and ascites. Avoid processed foods, which contribute >75% of dietary sodium.
- Alcohol avoidance: Absolute abstinence is non-negotiable, as alcohol accelerates liver damage in MASLD.
- Hydration: Encourage 1.5–2 L/day of water unless ascites/edema is severe (then follow fluid restrictions per hepatology advice).

**2. Recommended Daily Protein Intake**

- Start with 0.8 g/kg/day of actual body weight (64 g/day for 80 kg), then gradually increase to 1.0–1.2 g/kg/day (80–96 g/day) if tolerated (no HE symptoms for ≥2 weeks).
  - *Rationale*: Lower initial intake reduces ammonia production risk in HE-prone patients, while gradual escalation combats sarcopenia. Do not exceed 1.2 g/kg/day without specialist approval.
- Protein distribution: Spread intake evenly across all meals/snacks (e.g., 15–20 g per eating occasion) to avoid ammonia spikes.
- Monitor closely: If HE symptoms recur (e.g., confusion, tremors), reduce protein to 0.6 g/kg/day temporarily and consult hepatology. Never eliminate protein—this worsens outcomes.

**3. Meal Frequency and Eating Pattern**

- 4–6 small meals/snacks daily, including a bedtime snack (e.g., 15–20 g protein + complex carbs).
  - *Why?* Frequent meals maintain stable blood glucose, reduce overnight catabolism, and prevent ammonia buildup from large protein loads. The bedtime snack is critical—fasting >3–4 hours increases muscle breakdown in cirrhosis.
- Sample schedule:
  - Breakfast: Oatmeal + 1 egg + berries.
  - Mid-morning: Greek yogurt + almonds.
  - Lunch: Quinoa salad with chickpeas + vegetables.
  - Afternoon: Whole-grain toast + hummus.
  - Dinner: Baked fish + sweet potato + steamed broccoli.
  - Bedtime: Casein-based protein shake (slow-digesting; 15–20 g protein).
- Avoid prolonged fasting: Never skip meals; carry portable snacks (e.g., nuts, fruit).

**4. Specific Food Recommendations or Restrictions**

- Recommended foods:
  - Proteins: Prioritize plant-based (tofu, lentils, chickpeas) and dairy (Greek yogurt, cottage cheese, whey/casein protein shakes). Include lean poultry/fish 2–3×/week (e.g., 85 g portions). *Rationale*: Plant/dairy proteins generate less ammonia than red meat.
  - Carbohydrates: Whole grains (oats, quinoa, brown rice), fruits (berries, apples), and non-starchy vegetables (broccoli, spinach). These provide fiber (aids HE prevention) and antioxidants.
  - Fats: Unsaturated fats only (avocado, olive oil, walnuts); limit to 25–30% of calories to avoid worsening steatosis.
  - Fiber: 25–30 g/day from vegetables, fruits, and whole grains to promote regular bowel movements (critical for HE prevention).
- Strict restrictions:
  - Sodium: Avoid canned soups, processed meats, soy sauce, and salty snacks. Cook with herbs/spices instead of salt.
  - Animal proteins: Limit red meat, pork, and processed meats (high in methionine, which increases ammonia). Avoid organ meats entirely.
  - High-ammonia foods: Skip fermented products (e.g., aged cheese, sauerkraut) and excessive nuts/seeds.
  - Sugars: Minimize added sugars/sweetened beverages (aggravates MASLD); use small amounts of honey or maple syrup if needed.
  - Alcohol and supplements: Zero alcohol; avoid unregulated herbal supplements (e.g., kava, green tea extract), which can harm the liver.

**5. Additional Nutritional Considerations**

- Vitamin/mineral supplementation:
  - Vitamin D: 1,000–2,000 IU/day (deficiency is universal in cirrhosis; worsens muscle function).
  - B-complex vitamins: Especially B1 (thiamine) and B12 (common deficiencies from malabsorption/alcohol history).
  - Zinc: 15–25 mg/day (corrects deficiency linked to HE; found in pumpkin seeds, but supplement if diet-insufficient).
  - *Avoid high-dose iron or vitamin A* (risk of toxicity in cirrhosis).
- HE-specific strategies:
  - Prevent constipation: Ensure adequate fiber/fluids; consider lactulose if prescribed (it lowers colonic pH, reducing ammonia absorption).
  - Probiotics: Daily fermented foods (e.g., unsweetened yogurt) or supplements (e.g., *Lactobacillus* strains) may improve gut flora and reduce HE risk.
- Metabolic considerations:
  - Given MASLD and overweight status, moderate calorie deficit (if BMI >30) may be advised later, but never during active decompensation. Focus first on protein/energy adequacy.
  - Screen for insulin resistance; if present, emphasize low-glycemic-index carbs (e.g., legumes, non-starchy veggies).
- Monitoring:
  - Track weight weekly (sudden loss >0.5 kg/week suggests catabolism).
  - Check ammonia levels, INR, and albumin every 3–6 months.
  - Refer to a dietitian for personalized meal planning and adherence support.

## C3-04

**Dietary and Nutritional Recommendations for a Patient with HBV Cirrhosis in Hepatic Encephalopathy Recovery Phase**

**Clinical Summary**:
44-year-old female, HBV-related cirrhosis, height 160 cm, weight 68 kg (BMI 26.6, overweight). Currently in recovery phase of hepatic encephalopathy (HE).

**1. Overall Dietary Principles**

- Prioritize protein adequacy while minimizing ammonia production: Balance high-quality protein intake to prevent muscle wasting (a key HE trigger) without overloading ammonia metabolism.
- Control sodium strictly: Limit to < 2,000 mg/day to reduce ascites/edema risk.
- Ensure sufficient calories: Target 30–35 kcal/kg/day (2,040–2,380 kcal total) to prevent catabolism. Use complex carbohydrates (e.g., oats, whole grains) as primary energy sources.
- Avoid alcohol and hepatotoxins: Zero alcohol; limit processed foods with preservatives.
- Individualize adjustments: Monitor for HE recurrence; adjust protein based on tolerance.

**2. Recommended Daily Protein Intake**

- Start at 0.8–1.0 g/kg/day (54–68 g total) during HE recovery phase to minimize ammonia risk.
- Gradually increase to 1.2–1.5 g/kg/day (82–102 g) *only if stable* (no HE symptoms for ≥2 weeks), as muscle preservation is critical.
- Distribute evenly across meals (e.g., 15–20 g/meal) to avoid ammonia spikes.
- Emphasize plant-based and dairy proteins (e.g., lentils, tofu, Greek yogurt) over red meat, as they produce less ammonia.

**3. Meal Frequency and Eating Pattern**

- 4–6 small meals/snacks daily: Prevents prolonged fasting (which increases ammonia) and stabilizes blood glucose.
- Include protein at every meal: E.g., breakfast with eggs + whole-grain toast; afternoon snack with almonds.
- Avoid overnight fasting: Provide a bedtime snack with slow-digesting protein (e.g., cottage cheese + berries) to reduce catabolism during sleep.
- Hydration: 1.5–2 L/day of water; limit fluids if ascites severe (per physician guidance).

**4. Specific Food Recommendations or Restrictions**

表格

| **Recommend** | **Restrict/Avoid** |
| --- | --- |
| **Protein**: Plant-based (tofu, lentils), egg whites, low-fat dairy (Greek yogurt, cottage cheese), white fish. | Red meat, processed meats (bacon, sausages), organ meats (high in ammonia precursors). |
| **Carbs**: Oats, quinoa, sweet potatoes, whole-wheat bread (high-fiber, low-glycemic). | Refined sugars, white bread/pasta (may worsen insulin resistance). |
| **Fats**: Avocado, olive oil, nuts/seeds (limit to 1 oz/day). | Fried foods, saturated/trans fats (butter, margarine). |
| **Sodium**: Herbs/spices (turmeric, garlic), lemon juice for flavor; fresh/frozen vegetables. | Salt, soy sauce, canned soups, deli meats, pickled foods (>200 mg/serving). |
| **Other**: Probiotic-rich foods (kefir, kimchi) to support gut health. | Alcohol, raw shellfish (infection risk), excessive caffeine. |

**5. Additional Nutritional Considerations**

- Vitamin/mineral supplementation:
  - Vitamin D (1,000–2,000 IU/day): Cirrhosis causes deficiency; critical for bone/muscle health.
  - B-complex vitamins (especially B1, B6, folate): Depleted in liver disease; support energy metabolism.
  - Zinc (15–25 mg/day): Reduces ammonia and improves HE; avoid high-dose copper (toxic in cirrhosis).
  - *Avoid iron supplements unless deficient* (HBV cirrhosis increases iron overload risk).
- Gut health focus:
  - Soluble fiber (psyllium, apples) to promote regular bowel movements (constipation ↑ HE risk).
  - Continue lactulose as prescribed (targets gut ammonia); pair with fiber for synergy.
- Weight management:
  - Mild caloric deficit (200–300 kcal/day) if BMI >25, but avoid rapid weight loss (aggravates muscle loss). Target 0.5 kg/week max.
- Monitoring:
  - Track weight weekly; report sudden gains (↑ ascites) or losses (↑ muscle wasting).
  - Check serum albumin, prealbumin, and ammonia levels quarterly. Adjust protein if HE recurs.

**Key Rationale**:

- Protein timing/distribution reduces postprandial ammonia spikes better than total daily restriction.
- Plant-based proteins generate less ammonia due to lower sulfur-containing amino acids and gut microbiome benefits.
- Sodium control is non-negotiable; even mild ascites can worsen with >2 g/day intake.
- Individualization is critical: If HE recurs, temporarily reduce protein to 0.6 g/kg/day and consult hepatology.

**Patient Education Tips**:

- Teach label reading: Aim for <140 mg sodium/serving.
- Provide a sample 1-day meal plan (e.g., breakfast: oatmeal + chia seeds + almond milk; lunch: quinoa salad with chickpeas and veggies).
- Stress consistency: Skipping meals or high-sodium "cheat days" can trigger HE relapse.

## C3-05

**Dietary and Nutritional Recommendations for a Patient with Alcohol Cirrhosis and Prior Hepatic Encephalopathy**

As a physician specializing in clinical nutrition, I have reviewed the case of this 49-year-old male patient (height: 174 cm, weight: 90 kg) with alcohol-induced cirrhosis and a history of hepatic encephalopathy (HE).**Absolute alcohol abstinence is non-negotiable and must be emphasized as the foundation of all dietary advice.** Below, I provide practical, daily-oriented recommendations tailored to this patient’s clinical profile.

**1. Overall Dietary Principles**

- High-calorie, moderate-protein diet: Aim for 35–40 kcal/kg of *ideal body weight* per day to counteract catabolism and prevent muscle wasting (a key driver of HE). Ideal body weight for this patient (174 cm height) is ~70 kg (calculated using BMI midpoint of 22.5 kg/m²). Thus, target 2,450–2,800 kcal/day. Prioritize complex carbohydrates (e.g., whole grains) to spare protein for tissue repair, not energy.
- Sodium restriction: Limit to <2,000 mg/day to minimize ascites/edema risk. Avoid adding salt during cooking; use herbs/spices (e.g., lemon, garlic) for flavor.
- Alcohol elimination: Zero alcohol intake—no exceptions—to halt liver inflammation and fibrosis progression.
- Balanced macronutrients: Focus on plant-based proteins and healthy fats (e.g., olive oil, avocados) to reduce ammonia production. Avoid excessive saturated fats (e.g., fried foods), which strain liver metabolism.
- Monitor for malnutrition: Cirrhosis increases energy needs by 25–30%; regular weight checks (weekly) are essential. If weight drops >5% in 1 month, consult a dietitian for calorie-dense supplements.

**2. Recommended Daily Protein Intake**

- Target: 1.2–1.5 g/kg of ideal body weight/day (84–105 g/day for this patient, based on 70 kg ideal weight).
  - *Rationale*: Protein is critical to maintain muscle mass (reducing HE risk from muscle catabolism), but excessive intake can elevate blood ammonia in HE-prone patients. Start at the lower end (1.2 g/kg) and increase gradually if HE symptoms (e.g., confusion, tremors) do not recur.
- Protein distribution: Spread intake evenly across meals (e.g., 20–25 g per meal) to avoid ammonia spikes. Prioritize plant-based and dairy proteins (e.g., lentils, tofu, Greek yogurt), which produce less ammonia than red meat. Limit animal proteins to lean poultry/fish (max 100 g/day).
- Avoid high-risk proteins: Minimize processed meats (bacon, sausages) and excessive egg yolks due to high sulfur content (amplifies ammonia). If HE symptoms emerge, temporarily reduce to 0.8 g/kg and seek immediate medical review.

**3. Meal Frequency and Eating Pattern**

- 4–6 small meals/day: Eating every 3–4 hours stabilizes blood glucose, prevents catabolism, and reduces HE triggers from large protein loads. Example schedule:
  - Breakfast (7 AM): 30–40 g protein
  - Mid-morning snack (10 AM): 10–15 g protein
  - Lunch (1 PM): 30–40 g protein
  - Afternoon snack (4 PM): 10–15 g protein
  - Dinner (7 PM): 30–40 g protein
  - Bedtime snack (10 PM): Critical—include 15–20 g protein (e.g., casein-rich cottage cheese or a small handful of almonds) to sustain overnight muscle synthesis and prevent nocturnal hypoglycemia.
- Avoid fasting: Never skip meals; >3-hour gaps increase catabolism. Carry portable snacks (e.g., protein bars with <5 g sugar).

**4. Specific Food Recommendations or Restrictions**

- Recommended foods:
  - *Proteins*: Tofu, tempeh, lentils, chickpeas, skinless chicken breast, baked salmon (1–2x/week), low-fat Greek yogurt, eggs (max 1–2/day, mostly whites).
  - *Carbohydrates*: Oats, quinoa, brown rice, sweet potatoes, whole-grain bread (low-sodium).
  - *Fats*: Avocado, walnuts, chia seeds, olive oil (1–2 tbsp/day).
  - *Fruits/vegetables*: Bananas (for potassium), berries, spinach, broccoli (steamed to aid digestion). Aim for 5+ servings/day for antioxidants.
- Strict restrictions:
  - *Alcohol*: All forms prohibited.
  - *High-sodium foods*: Processed snacks (chips, canned soups), soy sauce, pickles, deli meats (sodium >400 mg/serving).
  - *HE-triggering foods*: Red meat (beef, pork), organ meats, and high-amine foods (aged cheeses, cured meats). Limit nuts/seeds to 1 oz/day (high fat may worsen steatorrhea).
  - *Other*: Sugary drinks (risk of fatty liver exacerbation), raw shellfish (infection risk in cirrhosis).
- Practical tip: Use a food diary app (e.g., MyFitnessPal) to track sodium/protein. A sample day:
  - Breakfast: Oatmeal (½ cup dry) + 1 scoop plant-based protein powder + 10 almonds (35 g protein).
  - Dinner: Baked cod (100 g) + quinoa (½ cup) + roasted broccoli (1 cup) (30 g protein).

**5. Additional Nutritional Considerations**

- Vitamin/mineral supplementation:
  - Cirrhosis impairs storage of fat-soluble vitamins (A, D, E, K) and B vitamins. Prescribe a liver-specific multivitamin (e.g., containing 1,000 IU vitamin D, 100 mg thiamine, 50 mg zinc) daily. Avoid high-dose iron unless ferritin-confirmed deficiency (excess iron harms the liver).
  - *Critical for HE*: Branched-chain amino acids (BCAAs) supplements (e.g., 4–8 g/day) may reduce HE recurrence if dietary protein is poorly tolerated—discuss with hepatology.
- Fluid and electrolyte management:
  - No fluid restriction unless severe hyponatremia (serum Na <125 mmol/L). If ascites present, sodium restriction is paramount; avoid "light" salt (high potassium).
  - Monitor for hypokalemia (common with diuretics)—include potassium-rich foods (bananas, oranges) but avoid excessive intake if renal impairment exists.
- Lifestyle integration:
  - Exercise: Light resistance training (e.g., 20-min daily walking + bodyweight exercises) preserves muscle mass. Avoid exhaustive activity.
  - HE monitoring: Teach the patient/family to recognize early HE signs (e.g., slurred speech). If symptoms occur, reduce protein temporarily and use lactulose as prescribed.
  - Follow-up: Schedule monthly nutrition reviews to adjust calories/protein based on weight trends, MELD score, and HE status. Referral to a liver-focused dietitian is strongly advised.

These recommendations balance evidence-based safety with practicality for outpatient management. **Key priorities are consistent protein distribution, strict sodium/alcohol limits, and proactive malnutrition prevention.** Given the history of HE, gradual dietary changes and close symptom monitoring are essential. I recommend the patient carries a written meal plan and contacts their hepatology team immediately if HE symptoms recur. For personalized adjustments, a formal nutrition assessment (e.g., handgrip strength, mid-arm circumference) should be performed within 2 weeks.

## C3-06

**Dietary and Nutritional Recommendations for a Patient with MASLD Cirrhosis and History of Hepatic Encephalopathy**

As a physician specializing in clinical nutrition, I have reviewed the case of this 53-year-old female patient (height 157 cm, weight 58 kg; BMI 23.5, within normal range but with mild unintentional weight loss) diagnosed with metabolic-associated steatotic liver disease (MASLD) cirrhosis and a history of hepatic encephalopathy (HE). The primary goals of dietary management are to:

- Support liver function and prevent disease progression.
- Minimize the risk of HE recurrence by optimizing protein metabolism.
- Address mild weight loss through adequate caloric intake to promote gradual weight restoration.
- Manage complications common in cirrhosis (e.g., sodium retention, malnutrition).

**1. Overall Dietary Principles**

The diet should prioritize **high caloric intake, moderate high-quality protein, strict sodium restriction, and balanced micronutrients** to:

- Prevent catabolism and muscle wasting: Cirrhosis increases energy needs due to hypermetabolism; aim for 35–40 kcal/kg body weight/day (approximately 2,030–2,320 kcal/day for this patient) to reverse weight loss.
- Reduce HE risk: Avoid excessive protein that could elevate blood ammonia, but do not restrict protein severely (as this worsens sarcopenia). Focus on plant-based and dairy proteins, which produce less ammonia than animal proteins.
- Limit sodium to <2 g/day (2,000 mg) to prevent fluid retention, ascites, and edema.
- Ensure micronutrient adequacy: Cirrhosis impairs vitamin absorption (e.g., B vitamins, vitamin D, zinc); emphasize nutrient-dense foods and consider targeted supplementation.
- Avoid alcohol completely, as it accelerates liver damage in MASLD.

**2. Recommended Daily Protein Intake**

- Target: 1.0–1.2 g/kg body weight/day (58–70 g total protein).
  - *Rationale*: This range balances the need to prevent muscle loss (sarcopenia) without overloading ammonia production. Higher intakes (>1.5 g/kg) may trigger HE in susceptible patients, while lower intakes (<0.8 g/kg) worsen outcomes.
  - *Calculation*: 58 kg × 1.2 g/kg = 70 g protein/day. Distribute evenly across meals to avoid ammonia spikes.
  - *Key emphasis*: Prioritize plant-based proteins (e.g., lentils, tofu, tempeh) and dairy (e.g., Greek yogurt, cottage cheese) over red meat or processed meats, as they generate fewer ammonia precursors. If HE recurs, temporarily reduce to 1.0 g/kg but reintroduce protein gradually.

**3. Meal Frequency and Eating Pattern**

- 5–6 small meals per day, spaced every 2.5–3 hours (e.g., breakfast, mid-morning snack, lunch, afternoon snack, dinner, bedtime snack).
  - *Rationale*: Frequent meals stabilize blood glucose, reduce the liver’s metabolic burden, and prevent overnight catabolism. Large meals can overwhelm ammonia clearance in cirrhosis.
- Mandatory bedtime snack: Include 15–20 g complex carbohydrates + 5–10 g protein (e.g., oatmeal with almond milk or whole-grain toast with peanut butter). This suppresses nocturnal protein breakdown and lowers HE risk.
- Avoid fasting: Never skip meals; even short fasts (>4 hours) can trigger catabolism.

**4. Specific Food Recommendations or Restrictions**

**Recommended Foods**:

- Proteins: Plant sources (1 cup cooked lentils = 18 g protein; ½ cup tofu = 10 g), low-fat dairy (1 cup Greek yogurt = 20 g), and egg whites. Limit fish/poultry to 2–3 servings/week (e.g., baked salmon).
- Carbohydrates: Whole grains (oats, quinoa, brown rice) for sustained energy; fruits (berries, apples) and non-starchy vegetables (broccoli, spinach) for fiber and antioxidants.
- Fats: Healthy unsaturated fats only (avocado, olive oil, nuts/seeds in moderation; e.g., 1 tbsp olive oil or ¼ avocado per meal).
- Sodium-safe flavoring: Use herbs, lemon, garlic, or salt-free spices instead of salt.

**Strict Restrictions**:

- Sodium: Avoid processed foods (canned soups, deli meats), salty snacks, and added table salt. Check labels for "sodium-free" or "low-sodium" options (<140 mg/serving).
- Protein sources to limit: Red meat, organ meats, and processed meats (high in ammonia-producing amino acids). If HE flares, avoid these entirely.
- Other: Alcohol (absolute contraindication), high-fructose corn syrup (e.g., sugary drinks), and excessive saturated fats (fried foods, butter).

**5. Additional Nutritional Considerations**

- Sodium monitoring: Weigh daily; a sudden increase >1 kg may indicate fluid retention, requiring stricter sodium limits (<1.5 g/day). Use fresh/frozen foods instead of canned.
- Micronutrient support:
  - Vitamin D: 1,000–2,000 IU/day supplement (common deficiency in cirrhosis; aids muscle function).
  - B-complex vitamins: Especially thiamine (B1) and folate (B9), as alcohol history or poor intake may deplete them.
  - Zinc: 25–50 mg/day if serum levels are low (helps ammonia detoxification).
  - *Avoid high-dose iron or vitamin A supplements*, which can be hepatotoxic.
- Weight management: Aim for 0.5–1 kg/week weight gain initially. Track weekly weights; if no improvement in 2–4 weeks, increase calories by 200–300 kcal/day (e.g., add 1 tbsp nut butter to snacks).
- HE prevention:
  - Probiotic-rich foods (e.g., unsweetened kefir) may reduce ammonia-producing gut bacteria.
  - If constipation occurs (a HE trigger), use fiber supplements like psyllium (not lactulose unless prescribed).
- Follow-up: Monitor serum ammonia, albumin, and prealbumin every 3–6 months. Refer to a dietitian for personalized meal planning and adjust based on tolerance.

These recommendations are designed to be practical and sustainable. For example, a sample day might include:

- Breakfast: Oatmeal (½ cup dry) with 1 tbsp chia seeds + 1 cup unsweetened almond milk + ½ cup blueberries (450 kcal, 15 g protein).
- Snack: 1 small apple with 1 tbsp almond butter (200 kcal, 4 g protein).
- Lunch: Lentil soup (1.5 cups, homemade, low-sodium) + large spinach salad with olive oil dressing (500 kcal, 22 g protein).
- Snack: ¾ cup low-sodium cottage cheese with cucumber slices (150 kcal, 14 g protein).
- Dinner: Baked cod (3 oz) with roasted broccoli and quinoa (1 cup cooked) (450 kcal, 25 g protein).
- Bedtime snack: 1 slice whole-grain toast with 2 tbsp hummus (200 kcal, 6 g protein).

**Total**: ~1,950 kcal, 86 g protein (slightly above target to account for mild weight loss; adjust if HE symptoms arise).

If symptoms like confusion or fatigue worsen, reduce protein temporarily and seek immediate medical review. Always coordinate with the patient’s hepatologist for integrated care.

## C3-07

**Dietary and Nutritional Recommendations for a Patient with HBV Cirrhosis and History of Hepatic Encephalopathy**

As a physician specializing in clinical nutrition, I have reviewed the case of this 57-year-old male patient with HBV-related cirrhosis and a history of hepatic encephalopathy (HE). His current anthropometrics (height: 168 cm, weight: 62 kg) indicate a BMI of 22.0 kg/m², which falls within the normal range. However, cirrhosis significantly increases the risk of malnutrition, muscle wasting, and recurrent HE due to impaired liver function, reduced detoxification capacity, and potential ammonia accumulation. The primary goals of dietary management are to:

- Prevent HE recurrence by minimizing ammonia production.
- Maintain adequate nutrition to support liver function and prevent sarcopenia.
- Address complications like fluid retention (if present) and micronutrient deficiencies.

**1. Overall Dietary Principles**

- Energy Balance: Aim for 30–35 kcal/kg/day to prevent catabolism and muscle loss. For this patient (62 kg), this translates to 1,860–2,170 kcal/day. Prioritize complex carbohydrates (e.g., whole grains) for 50–60% of calories to stabilize blood glucose and reduce reliance on protein for energy.
- Sodium Restriction: Limit sodium to <2,000 mg/day (equivalent to 5 g of salt) to minimize ascites and edema risk. This is critical even if ascites is not currently present, as cirrhosis predisposes to fluid retention.
- Protein Quality over Quantity: Focus on plant-based and dairy proteins, which produce less ammonia than animal proteins. Avoid excessive protein restriction, as this can worsen muscle breakdown and HE risk.
- Ammonia Management: Emphasize foods that promote gut health (e.g., fiber-rich foods) to reduce ammonia-producing bacteria. Include probiotics (e.g., unsweetened yogurt) and avoid constipation through adequate fluid and fiber intake.
- Alcohol and Toxins: Strictly avoid all alcohol and hepatotoxic substances (e.g., certain herbal supplements), as they accelerate liver damage.

**2. Recommended Daily Protein Intake**

- Target: 1.0–1.2 g/kg/day of high-quality protein, equating to 62–74 grams per day for this patient (based on actual weight of 62 kg).
  - *Rationale*: Higher protein intake (>1.5 g/kg) may trigger HE in susceptible individuals, but too little (<0.8 g/kg) exacerbates sarcopenia. The 1.0–1.2 g/kg range balances muscle preservation with HE prevention.
- Distribution: Divide protein evenly across all meals/snacks (e.g., 15–20 g per meal) to avoid ammonia spikes. Prioritize:
  - Plant-based proteins (e.g., lentils, tofu, tempeh, chickpeas) for 50–70% of total protein—these generate less ammonia due to lower aromatic amino acid content.
  - Dairy proteins (e.g., Greek yogurt, cottage cheese) for 30–40%—easier to metabolize than red meat.
  - Limit animal proteins (e.g., poultry, fish) to ≤20 g/day; avoid red meat and processed meats entirely due to high ammonia potential.
- Supplementation: Consider a daily branched-chain amino acid (BCAA) supplement (e.g., 4–6 g/day) if dietary adherence is poor, as BCAAs may reduce HE recurrence. Consult a dietitian for product selection.

**3. Meal Frequency and Eating Pattern**

- Frequency: 5–6 small meals/snacks daily, spaced every 2.5–3 hours (e.g., breakfast, mid-morning snack, lunch, afternoon snack, dinner, bedtime snack).
  - *Rationale*: Frequent eating prevents prolonged fasting, which triggers muscle catabolism and ammonia release. A bedtime snack is crucial to maintain overnight energy stores.
- Pattern:
  - Breakfast: High-carb, moderate-protein (e.g., oatmeal with berries and a small portion of nuts).
  - Bedtime Snack: Must include complex carbs + protein (e.g., whole-grain toast with cottage cheese) to sustain nocturnal glucose levels.
  - Avoid Fasting: Never skip meals; if appetite is poor, use liquid nutrition supplements (e.g., oral repletion formulas with BCAAs).
- Practical Tip: Use a meal planner to ensure consistent timing—e.g., eat at 7:00 AM, 10:00 AM, 1:00 PM, 4:00 PM, 7:00 PM, and 10:00 PM.

**4. Specific Food Recommendations or Restrictions**

- Recommended Foods:
  - Carbohydrates: Whole grains (brown rice, quinoa, oats), starchy vegetables (sweet potatoes), and fruits (apples, berries—rich in antioxidants).
  - Proteins: Tofu, edamame, lentils, chickpeas, Greek yogurt (unsweetened), and eggs (limit to 1–2/day).
  - Fats: Healthy unsaturated fats only—avocado, olive oil, and nuts/seeds (small portions, e.g., 10 almonds).
  - Vegetables: High-fiber options (broccoli, spinach, carrots) to support gut motility; include 4–5 servings daily.
  - Fluids: Water (1.5–2 L/day if no ascites); herbal teas (e.g., dandelion tea for liver support).
- Strict Restrictions:
  - Sodium: No processed foods (canned soups, deli meats), soy sauce, or added salt. Use herbs (e.g., garlic, turmeric) for flavor.
  - Ammonia-Producing Foods: Red meat, organ meats, processed meats (bacon, sausages), and excessive dairy (>3 servings/day).
  - Other: Alcohol (absolute zero), sugary drinks, fried foods, and high-mercury fish (e.g., tuna). Avoid raw shellfish due to infection risk.
- HE-Specific Adjustments: If early HE symptoms recur (e.g., confusion), temporarily reduce protein to 0.8 g/kg/day and increase soluble fiber (e.g., psyllium husk) to bind ammonia.

**5. Additional Nutritional Considerations**

- Micronutrient Supplementation:
  - Vitamin D: 1,000–2,000 IU/day (common deficiency in cirrhosis; supports bone and immune health).
  - B Vitamins: A B-complex supplement (especially B1, B6, B12) due to impaired storage in liver disease.
  - Zinc: 25 mg/day—deficiency worsens HE; found in pumpkin seeds or supplements.
  - *Avoid iron supplements* unless labs confirm deficiency, as excess iron damages the liver.
- Gut Health Management:
  - Fiber: 25–30 g/day from whole foods to prevent constipation (a HE trigger). If fiber causes bloating, start low (15 g/day) and increase gradually.
  - Probiotics: Daily unsweetened yogurt or a probiotic supplement (e.g., *Lactobacillus* strains) to reduce pathogenic gut bacteria.
  - Lactulose: If prescribed for HE prophylaxis, take as directed (typically 15–30 mL/day) to acidify the colon and trap ammonia.
- Monitoring and Follow-Up:
  - Track weight weekly; a loss >2 kg/month signals worsening nutrition.
  - Regular blood tests for ammonia, albumin, and prealbumin to adjust protein intake.
  - Refer to a registered dietitian specializing in liver disease for personalized meal planning and adherence support.
- Lifestyle Integration:
  - Encourage light daily activity (e.g., 20-minute walks) to preserve muscle mass.
  - Educate on reading food labels for sodium/protein content—aim for <140 mg sodium per serving.

These recommendations balance HE prevention with nutritional adequacy. Given the history of HE, close monitoring is essential—any confusion, fatigue, or sleep disturbances warrant immediate medical review. I strongly advise a follow-up appointment within 2 weeks to assess tolerance and adjust the plan. For reliable resources, refer to the AASLD’s *Nutrition in Cirrhosis* guidelines (2023) or the patient education materials from the American Liver Foundation. If symptoms worsen, discontinue high-protein foods and seek urgent care.

## C3-08

**Dietary and Nutritional Recommendations for Cirrhosis with History of Hepatic Encephalopathy**

**1. Overall Dietary Principles**

- Energy balance: Provide adequate calories (35-40 kcal/kg of ideal body weight daily) to prevent catabolism and muscle wasting, which is critical in cirrhosis management.
- Alcohol abstinence: Complete and permanent avoidance of all alcohol-containing products is non-negotiable.
- Individualized approach: Focus on maintaining muscle mass while preventing hepatic encephalopathy recurrence.
- Sodium restriction: Limit to 2,000 mg/day if ascites is present (common in decompensated cirrhosis).
- Nutrient density: Prioritize high-quality, nutrient-dense foods to address common micronutrient deficiencies in alcohol-related liver disease.

**2. Recommended Daily Protein Intake**

- Target: 1.2-1.5 g/kg of ideal body weight per day (not current weight, as the patient is obese with BMI 31.2).
- Calculation: For her height (156 cm), ideal body weight is approximately 50 kg → 60-75 g protein daily.
- Implementation: Start at the lower end (1.2 g/kg) and gradually increase if hepatic encephalopathy remains controlled.
- Protein source emphasis: Prioritize vegetable-based proteins (legumes, tofu) and dairy (yogurt, cheese) over red meat, as these may produce less ammonia. Branched-chain amino acid (BCAA)-enriched protein sources can be beneficial if standard protein intake triggers encephalopathy.

**3. Meal Frequency and Eating Pattern**

- 4-6 small meals daily to prevent prolonged fasting states that accelerate muscle breakdown.
- Bedtime snack containing 15-20 g of protein (e.g., casein-rich dairy like Greek yogurt or cottage cheese) to reduce overnight catabolism.
- Even protein distribution across all meals (rather than concentrating protein in one meal) to minimize ammonia spikes.
- Consistent timing to support metabolic stability.

**4. Specific Food Recommendations and Restrictions**

**Recommended:**

- Protein sources: Eggs, Greek yogurt, tofu, lentils, chickpeas, and limited poultry/fish (max 3-4 oz per meal).
- Complex carbohydrates: Oats, brown rice, quinoa, and whole grains for sustained energy.
- Fiber-rich foods: Fruits, vegetables, and legumes (30-35 g fiber daily) to promote regular bowel movements and reduce ammonia absorption.
- Healthy fats: Avocado, olive oil, and nuts in moderation.

**Restrictions:**

- Alcohol: Complete avoidance.
- Sodium: Avoid processed foods, canned soups, and salty snacks; use herbs instead of salt for flavoring.
- High-ammonia foods: Limit red meat and processed meats.
- Excessive simple sugars: To prevent worsening insulin resistance (common in obesity and cirrhosis).

**5. Additional Nutritional Consider**

- Micronutrient supplementation:
  - Thiamine (100 mg/day) and multivitamin (particularly B-complex) due to chronic alcohol use history.
  - Vitamin D (1,000-2,000 IU/day) if deficient (common in cirrhosis).
  - Zinc (15-25 mg/day) to address frequent deficiencies and support ammonia metabolism.
- Constipation prevention: Ensure adequate fluid intake (1.5-2 L/day unless ascites/edema contraindicated) and fiber to reduce ammonia absorption.
- Monitoring: Track for hepatic encephalopathy symptoms (confusion, sleep disturbances) after dietary changes. If symptoms recur, temporarily reduce protein to 0.8 g/kg and consult hepatology for possible BCAA supplementation.
- Weight management: Focus on gradual weight loss (if needed) through portion control rather than severe calorie restriction, which could worsen muscle loss.

**Important note**: These recommendations should be implemented under supervision of a dietitian specializing in liver disease, with regular follow-up to adjust based on clinical response, laboratory values (ammonia, albumin), and tolerance.

## C3-09

**Dietary and Nutritional Recommendations for a Patient with MASLD Cirrhosis, Hepatic Encephalopathy Recovery, and Low BMI**

As a physician specializing in clinical nutrition, I have reviewed the case of this 64-year-old male patient (height: 167 cm, weight: 57 kg, BMI: 20.4, which is at the lower end of the normal range but considered low-risk for malnutrition in cirrhosis). The diagnosis includes Metabolic dysfunction-Associated Steatotic Liver Disease (MASLD) cirrhosis, with recent recovery from hepatic encephalopathy (HE) and a low BMI indicating undernutrition. Cirrhosis in MASLD requires careful dietary management to prevent disease progression, support liver function, and address complications like HE.

**1. Overall Dietary Principles**

The primary goals are to:

- Provide adequate energy to prevent catabolism and promote weight gain, targeting a gradual increase in BMI to 22–24 (ideal weight range for height: ~61–66 kg). This helps counteract muscle wasting, which is common in cirrhosis and increases mortality risk.
- Balance protein intake to support muscle synthesis without triggering HE, emphasizing high-quality, plant-based, and dairy proteins that produce less ammonia. Avoid strict protein restriction, as it can worsen sarcopenia.
- Prioritize metabolic health due to MASLD: Limit refined carbohydrates and saturated fats to improve insulin sensitivity, but ensure sufficient complex carbs for energy.
- Ensure micronutrient sufficiency, as cirrhosis often causes deficiencies in vitamins (e.g., B vitamins, D, K) and minerals (e.g., zinc, magnesium).
- Avoid alcohol completely and minimize sodium to prevent fluid retention (ascites risk). Total daily calories should be 35–40 kcal/kg of current body weight (≈2,000–2,300 kcal/day) to support weight gain without overloading the liver.

**2. Recommended Daily Protein Intake**

- Target: 1.3–1.5 g/kg of current body weight per day (74–86 g/day for this 57 kg patient). This range is supported by ESPEN and AASLD guidelines for cirrhotic patients with a history of HE in recovery and low BMI:
  - Lower end (1.3 g/kg): Start at 74 g/day to monitor for HE recurrence, then gradually increase to 1.5 g/kg (86 g/day) as tolerated.
  - Rationale: Protein restriction (<0.8 g/kg) is outdated for stable HE recovery, as it exacerbates muscle loss. Adequate protein preserves muscle mass, which acts as an ammonia buffer. Plant and dairy proteins (e.g., legumes, yogurt) are preferred over red/processed meats, as they generate less ammonia.
- Distribution: Spread protein evenly across meals (e.g., 20–25 g per main meal) to avoid ammonia spikes. If HE symptoms recur (e.g., confusion, fatigue), reduce temporarily to 1.0 g/kg and consult a hepatologist.

**3. Meal Frequency and Eating Pattern**

- Adopt a 4–6 meal pattern daily: 3 main meals + 2–3 snacks, including a bedtime snack (e.g., casein-rich dairy like cottage cheese). This prevents prolonged fasting, which accelerates muscle breakdown and increases ammonia production.
  - Why this works: Cirrhosis often causes early satiety and delayed gastric emptying. Smaller, frequent meals improve nutrient absorption and maintain positive nitrogen balance. The bedtime snack (15–20 g protein) is critical—it reduces overnight catabolism by 30–40%, per ESPEN data.
- Timing example:
  - Breakfast: 7–8 AM
  - Mid-morning snack: 10–11 AM
  - Lunch: 1–2 PM
  - Afternoon snack: 4–5 PM
  - Dinner: 7–8 PM
  - Bedtime snack: Before 10 PM (e.g., 1 cup low-fat yogurt + almonds).
- Avoid >3-hour fasting periods; set alarms if needed for adherence.

**4. Specific Food Recommendations or Restrictions**

**Recommended Foods**:

- Proteins: Prioritize plant-based (tofu, lentils, chickpeas) and dairy (Greek yogurt, cottage cheese, milk) for 60–70% of daily protein. Include lean poultry/fish (e.g., baked salmon) 2–3×/week for omega-3s. Eggs are excellent (1–2/day).
- Carbohydrates: Focus on low-glycemic, high-fiber options (oats, quinoa, sweet potatoes, whole-grain bread) to manage MASLD-related insulin resistance. Limit added sugars to <25 g/day.
- Fats: Use unsaturated fats (olive oil, avocado, nuts) for 25–30% of calories; include omega-3-rich foods (chia seeds, walnuts) to reduce inflammation.
- Fruits/Vegetables: 5+ servings/day (e.g., berries, spinach, broccoli) for antioxidants and fiber. Steam vegetables to ease digestion.
- Hydration: 1.5–2 L/day of water; avoid sugary drinks.

**Restrictions and Cautions**:

- Sodium: Limit to <2,000 mg/day (avoid processed foods, canned soups, soy sauce; use herbs/spices for flavor).
- Animal Proteins: Restrict red meat, pork, and processed meats (bacon, sausages) to ≤1 serving/week—they increase ammonia and inflammation.
- High-Risk Foods: No raw/undercooked seafood or eggs (infection risk in cirrhosis); limit high-mercury fish (e.g., tuna).
- Alcohol: Strictly prohibited—zero tolerance, as it accelerates liver damage.
- Other: Avoid excessive branched-chain amino acid (BCAA) supplements unless prescribed; whole foods are preferred.

**5. Additional Nutritional Considerations**

- Micronutrient Supplementation:
  - Vitamin D: 1,000–2,000 IU/day (common deficiency in cirrhosis; improves muscle function).
  - B Vitamins: Especially B1 (thiamine) and B12—consider a daily multivitamin with 100% RDA, as alcohol history or poor intake may deplete these.
  - Zinc: 25 mg/day (deficiency worsens HE; found in pumpkin seeds, but supplement if diet is insufficient).
  - *Rationale*: Cirrhosis impairs storage and metabolism of these nutrients. Blood tests (e.g., 25-OH vitamin D, zinc) should guide dosing.
- Oral Nutritional Supplements (ONS): Given the low BMI, add 1–2 daily ONS shakes (e.g., 300 kcal, 20 g protein each) between meals if weight gain lags. Choose liver-specific formulas (e.g., with BCAAs) or standard high-protein options (e.g., Ensure Plus®), but avoid excessive fructose.
- Monitoring and Safety:
  - Track weight weekly; aim for 0.5 kg gain/week. Rapid gain (>1 kg/week) may indicate fluid retention—check for edema/ascites.
  - Watch for HE recurrence: If confusion or sleep disturbances occur, reduce protein temporarily and seek immediate care.
  - Coordinate with hepatology: Diuretics (e.g., for ascites) may require potassium/magnesium adjustments.
- MASLD-Specific Advice: Since MASLD underlies this cirrhosis, integrate weight management—avoid high-calorie "junk" foods. A dietitian should tailor this plan to address insulin resistance (e.g., pairing carbs with protein/fat to blunt glucose spikes).

**Final Guidance**

This plan balances HE prevention with aggressive nutritional rehabilitation for undernutrition. Start conservatively (e.g., 1.3 g/kg protein) and escalate as tolerated, with follow-up in 2–4 weeks to assess weight, muscle strength, and HE symptoms. **Critical reminder**: Never self-adjust protein without medical input. Collaborate with a registered dietitian specializing in liver disease for personalized meal planning—this is essential for long-term success. With consistent adherence, most patients see improved energy, muscle mass, and reduced complication risks within 3–6 months. If you have further clinical details (e.g., recent ammonia levels), I can refine this advice.

## C3-10

**Dietary and Nutritional Recommendations for a Patient with HBV Cirrhosis, Prior Hepatic Encephalopathy, and Poor Appetite**

As a physician specializing in clinical nutrition, I have reviewed the case of this 68-year-old female patient with HBV-related cirrhosis, a history of hepatic encephalopathy (HE), and poor appetite. Her current anthropometrics (height: 159 cm, weight: 50 kg) indicate a body mass index (BMI) of 19.8 kg/m², which is at the lower end of the normal range (18.5–24.9 kg/m²). This, combined with her cirrhosis and HE history, places her at high risk for protein-energy malnutrition, sarcopenia, and recurrent HE episodes. Poor appetite further complicates nutritional management.

**1. Overall Dietary Principles**

The primary goals are to:

- Prevent malnutrition and sarcopenia: Cirrhosis increases metabolic demands, and inadequate intake accelerates muscle loss, worsening prognosis.
- Minimize hepatic encephalopathy risk: Avoid excessive ammonia production by balancing protein sources and timing, but do not severely restrict protein, as this can worsen muscle catabolism and HE long-term.
- Support liver function and reduce complications: Limit sodium to prevent fluid retention (common in cirrhosis), ensure adequate calories to spare protein for tissue repair, and prioritize easily digestible foods due to poor appetite.
- Individualize for appetite issues: Focus on nutrient-dense, palatable foods in small portions to overcome anorexia.
- Avoid hepatotoxins: Strict alcohol abstinence and exclusion of raw/uncooked foods (e.g., sushi, undercooked eggs) to reduce infection risk in immunocompromised states.

**2. Recommended Daily Protein Intake**

- Target: 1.0–1.2 grams of protein per kilogram of actual body weight per day (total: 50–60 grams/day).
  - *Rationale*: Her weight is 50 kg, and while standard cirrhosis guidelines recommend 1.2–1.5 g/kg/day, her HE history necessitates caution. Starting at 1.0 g/kg/day (50 g) reduces ammonia load, but 1.2 g/kg/day (60 g) is preferred if tolerated, as protein restriction below 0.8 g/kg/day increases mortality risk. Prioritize plant-based proteins (e.g., legumes, tofu), which produce less ammonia than animal proteins.
- Practical application:
  - Distribute protein evenly across all meals/snacks (e.g., 10–15 g per eating occasion) to avoid ammonia spikes.
  - Monitor for HE symptoms (e.g., confusion, drowsiness); if symptoms arise, temporarily reduce to 0.8 g/kg/day (40 g) and consult a hepatologist before adjusting.
  - *Example*: A 60 g target could include: 15 g from breakfast (e.g., ½ cup tofu), 15 g from lunch (e.g., ¼ cup lentils), 15 g from dinner (e.g., 3 oz fish), and 15 g from snacks (e.g., Greek yogurt).

**3. Meal Frequency and Eating Pattern**

- Frequency: 5–6 small meals/snacks daily, including a bedtime snack. Avoid fasting >3–4 hours to prevent muscle breakdown and hypoglycemia.
  - *Rationale*: Cirrhosis impairs glycogen storage, and prolonged fasting elevates ammonia. Frequent eating stabilizes blood glucose and reduces HE risk.
- Pattern:
  - Breakfast: Within 1 hour of waking (e.g., oatmeal with nut butter).
  - Lunch/Dinner: Smaller portions (½ to ⅔ of a standard plate) to accommodate poor appetite.
  - Bedtime snack: Essential—include protein + complex carbs (e.g., casein-rich dairy like cottage cheese) to suppress overnight catabolism. Aim for 15–20 g protein.
  - Hydration: Sip water/fluids steadily; avoid large volumes at once (may cause early satiety). Limit fluids to 1.5 L/day if ascites is present.
- *Practical tip*: Set alarms for meals/snacks to combat appetite loss. Use a food diary to track intake and symptoms.

**4. Specific Food Recommendations or Restrictions**

- Recommended foods:
  - Proteins: Plant-based sources first (e.g., lentils, chickpeas, tofu, tempeh, almonds). If including animal proteins, choose lean, low-fat options like eggs, skinless poultry, or fatty fish (salmon—rich in anti-inflammatory omega-3s). Limit to 2–3 oz per meal.
  - Carbohydrates: High-fiber, low-glycemic options for sustained energy (e.g., oats, quinoa, sweet potatoes, berries). Include fruit (e.g., bananas for potassium) to counter diuretic effects if on spironolactone.
  - Fats: Healthy monounsaturated fats (e.g., avocado, olive oil, walnuts) for calorie density; avoid fried foods.
  - Appetite boosters: Mild flavors like ginger (tea or crystallized), lemon, or mint to stimulate digestion; nutrient-dense smoothies (e.g., banana, spinach, almond milk, protein powder).
- Strict restrictions:
  - Sodium: <2,000 mg/day (≈1 tsp salt) to prevent ascites. Avoid processed foods, canned soups, soy sauce, and cured meats. Use herbs/spices (e.g., turmeric, garlic powder) for flavor.
  - High-ammonia triggers: Red meat, processed meats (bacon, sausages), and excessive dairy (limit to 1–2 servings/day of low-fat options).
  - Alcohol and toxins: Absolute avoidance—no exceptions.
  - Other: Raw shellfish (vibrio risk), high-mercury fish (e.g., tuna), and excessive bran (may worsen bloating).

**5. Additional Nutritional Considerations**

- Calorie needs: Target 30–35 kcal/kg/day (1,500–1,750 kcal total) to promote weight maintenance/gain. Prioritize calorie density (e.g., add olive oil to vegetables, use full-fat yogurt) due to poor appetite. If oral intake is insufficient, consider oral nutritional supplements (e.g., 1–2 servings/day of a liver-specific formula like Ensure Hepa or Fresubin Hepatic, which are low in aromatic amino acids and high in BCAAs).
- Micronutrient support:
  - Vitamin deficiencies are common: Prescribe a daily liver-appropriate multivitamin (e.g., containing 400–800 mcg folic acid, 1–2 mg B12, 50–100 mg B6, and 800–1,000 IU vitamin D). Avoid high-dose iron or vitamin A (risk of toxicity).
  - Zinc: 25–50 mg/day supplementation—deficiency exacerbates taste disturbances and HE.
  - Thiamine: 100 mg/day if alcohol history (though abstinent now), as cirrhosis increases deficiency risk.
- HE-specific strategies:
  - Prebiotics/fiber: 25–30 g/day from vegetables, fruits, and whole grains to promote bowel regularity and reduce ammonia absorption. Avoid lactulose-containing foods if on prescribed lactulose therapy.
  - BCAAs (Branched-Chain Amino Acids): Consider under medical supervision if HE recurs despite protein management (e.g., 4–8 g/day as a supplement).
- Monitoring and follow-up:
  - Weigh weekly; aim for gradual weight gain if undernourished (e.g., +0.5 kg/week).
  - Check ammonia levels, INR, and albumin every 3–6 months. Report worsening HE symptoms immediately.
  - Collaborate with her hepatologist to adjust diuretics or lactulose, as these affect nutrition (e.g., diuretics cause potassium/magnesium loss).
- Appetite management:
  - Eat in a calm environment; avoid large meals that cause fullness.
  - If nausea persists, try ginger chews or small sips of peppermint tea before meals.
  - Consult a dietitian for personalized meal plans and appetite-stimulating techniques (e.g., megestrol is not first-line due to side effects).

**Final Note**

These recommendations balance HE prevention with malnutrition risk. Given her poor appetite, start conservatively (e.g., 50 g protein/day) and titrate up as tolerated. Emphasize that **adequate protein is protective**—not harmful—when managed correctly. Schedule a follow-up in 2–4 weeks to assess adherence, symptoms, and weight. If intake remains suboptimal, discuss enteral supplementation (e.g., tube feeding is rarely needed but may be considered in severe cases). Always coordinate care with her hepatology team for holistic management.

## C3-11

**Dietary and Nutritional Recommendations for a Patient with Alcohol-Related Cirrhosis and Recurrent Hepatic Encephalopathy**

As a physician specializing in clinical nutrition, I have reviewed the case of this 72-year-old male patient with alcohol-related cirrhosis and recurrent hepatic encephalopathy (HE). His height is 170 cm, and his current weight is 74 kg (BMI ≈ 25.6 kg/m², indicating overweight status, though cirrhosis may mask muscle loss due to fluid retention). The primary goals of dietary management are to:

- Prevent malnutrition and muscle wasting (common in cirrhosis, especially in older adults).
- Minimize triggers for hepatic encephalopathy (e.g., excessive ammonia production).
- Address complications like fluid retention and micronutrient deficiencies.
- Avoid alcohol completely, as it accelerates liver damage.

**1. Overall Dietary Principles**

- Prioritize adequate energy (calorie) intake to prevent catabolism and muscle breakdown. Target 30–35 kcal/kg of *ideal body weight* per day (estimated at 65–70 kg for this patient, based on height). This translates to ~2,000–2,400 kcal daily. Insufficient calories force the body to break down muscle for energy, worsening HE risk.
- Moderate, high-quality protein intake with emphasis on plant-based sources to reduce ammonia production. Avoid severe protein restriction, as it exacerbates sarcopenia (muscle loss), which is already a concern in elderly cirrhosis patients.
- Strict sodium restriction to manage fluid retention and reduce ascites risk: Limit to < 2,000 mg (2 g) per day.
- Ensure balanced micronutrient intake, as cirrhosis often causes deficiencies in vitamins (e.g., B-complex, D, K) and minerals (e.g., zinc, magnesium).
- Eliminate all alcohol—non-negotiable, as it directly worsens liver function and HE.

**2. Recommended Daily Protein Intake**

- Target: 1.0–1.2 g of protein per kg of *ideal body weight* per day.
  - Ideal body weight estimate: 70 kg (based on height of 170 cm; standard range: 65–75 kg).
  - Daily protein goal: 70–84 g (e.g., 70 kg × 1.1 g/kg = 77 g).
- Why this range?
  - Lower limits (1.0 g/kg) help minimize ammonia generation in recurrent HE, but exceeding 1.2 g/kg may trigger episodes.
  - Prioritize plant-based proteins (e.g., legumes, tofu, lentils), which produce less ammonia than animal proteins due to lower aromatic amino acid content and higher fiber. If animal proteins are included, choose lean poultry or fish in small portions (≤30 g per meal), but avoid red meat, processed meats, and high-fat dairy, which increase HE risk.
- Monitor closely: If HE symptoms worsen (e.g., confusion, drowsiness), reduce protein temporarily to 0.8 g/kg and consult a hepatologist. Never restrict below 0.6 g/kg to prevent malnutrition.

**3. Meal Frequency and Eating Pattern**

- Small, frequent meals (5–6 times daily) to maintain stable blood glucose, reduce ammonia spikes, and support nitrogen balance. Long fasting periods (>3–4 hours) can trigger muscle catabolism and HE.
- Key schedule example:
  - Breakfast: Within 1 hour of waking.
  - Mid-morning snack: 10–11 AM.
  - Lunch: 12–1 PM.
  - Afternoon snack: 3–4 PM.
  - Dinner: 6–7 PM.
  - Bedtime snack: Critical to prevent overnight catabolism—include 15–20 g of plant-based protein (e.g., a small handful of almonds or a soy yogurt).
- Avoid large meals: Limit single meals to ≤30 g protein to reduce ammonia burden. Distribute protein evenly across all meals/snacks.

**4. Specific Food Recommendations or Restrictions**

- Recommended Foods:
  - Proteins: Plant-based options (e.g., ½ cup cooked lentils = 9 g protein; 100 g tofu = 8 g protein; 1 oz almonds = 6 g protein). Include eggs (1–2 daily) as a well-tolerated animal source.
  - Carbohydrates: Whole grains (oats, quinoa, brown rice) for sustained energy and fiber. Aim for 25–30 g fiber daily to prevent constipation (a HE trigger).
  - Fats: Unsaturated fats only (e.g., olive oil, avocado, fatty fish like salmon 2×/week for omega-3s).
  - Fruits/Vegetables: All non-starchy types (e.g., berries, leafy greens, carrots). Prioritize potassium-rich foods (bananas, spinach) to counteract diuretic effects if ascites is present.
  - Fluids: 1.5–2 L water daily unless ascites/edema requires restriction (per physician guidance). Avoid sugary drinks.
- Strict Restrictions:
  - Sodium: No processed foods (canned soups, deli meats, chips), added salt, or soy sauce. Use herbs (e.g., garlic, lemon) for flavor. Check labels for "sodium-free" options.
  - Animal proteins: Limit red meat, pork, and high-fat dairy (e.g., cheese, whole milk). Avoid organ meats (very high in protein/ammonia).
  - Constipation-promoting foods: Low-fiber refined carbs (white bread, pastries) and excessive caffeine.
  - Alcohol: Zero tolerance—remove all alcoholic beverages from the home.
  - High-ammonia foods: Avoid protein supplements with whey/casein; use only physician-approved options (e.g., vegetable-based powders if needed).

**5. Additional Nutritional Considerations**

- Prevent constipation: A major HE trigger, as gut bacteria produce more ammonia from retained stool. Ensure 25–30 g fiber daily from foods (e.g., chia seeds, apples) and consider a gentle fiber supplement (e.g., psyllium husk 5 g/day) if diet alone is insufficient. Encourage 30 minutes of light activity (e.g., walking) daily to aid digestion.
- Micronutrient supplementation:
  - Multivitamin: Daily high-potency B-complex (especially B1/thiamine, often deficient in alcohol-related cirrhosis) and vitamin D (2,000 IU/day; common deficiency in liver disease).
  - Zinc: 25–50 mg/day (deficiency impairs ammonia detoxification).
  - Avoid high-dose iron or vitamin A supplements unless prescribed, as they can accumulate in cirrhosis.
- Monitor for sarcopenia: Weigh weekly (aim for stable dry weight; rapid loss >2 kg/month needs intervention). Include resistance exercises (e.g., chair squats) 3×/week if medically cleared.
- HE-specific adjustments:
  - If HE recurs, add 2–3 g/day of oral lactulose (prescription-only) to acidify the colon and reduce ammonia absorption. Time it with meals.
  - Consider a trial of branched-chain amino acid (BCAA) supplements (e.g., 4–8 g/day) if muscle loss persists, though evidence is mixed—discuss with hepatology first.
- Lifestyle coordination:
  - Collaborate with a dietitian for personalized meal plans and regular nutrition assessments (e.g., every 3 months).
  - Screen for diabetes (common in cirrhosis); limit added sugars.
  - Family education: Teach caregivers to recognize early HE symptoms (e.g., mood changes) and ensure meal adherence.

**Final Note**: This plan balances HE prevention with malnutrition risk, which is critical in elderly cirrhosis patients. Reassess every 1–2 months based on weight trends, HE episodes, and lab work (e.g., ammonia, albumin, prealbumin). If HE remains uncontrolled, refer to a hepatology specialist for advanced options (e.g., rifaximin). Always individualize recommendations—this patient’s overweight BMI may mask underlying muscle loss, so focus on body composition over weight alone. Encourage strict abstinence from alcohol as part of holistic care.

## C3-12

**Dietary and Nutritional Recommendations for a 75-Year-Old Female with MASLD Cirrhosis and History of Hepatic Encephalopathy**

As a physician specializing in clinical nutrition, I have reviewed the case of this 75-year-old female patient (height: 155 cm, weight: 62 kg, BMI: 25.8 kg/m²) with metabolic dysfunction-associated steatotic liver disease (MASLD) cirrhosis and a prior episode of hepatic encephalopathy (HE). Her BMI indicates overweight status, but in cirrhosis, this may reflect fluid retention or sarcopenia rather than true adiposity; thus, nutritional management must prioritize muscle preservation while minimizing HE recurrence risk.

**1. Overall Dietary Principles**

The primary goals are to:

- Prevent hepatic encephalopathy recurrence: Limit ammonia-producing substrates while ensuring adequate protein for muscle synthesis.
- Support liver health and metabolic balance: Prioritize anti-inflammatory, nutrient-dense foods to address MASLD (e.g., reduce insulin resistance and oxidative stress).
- Avoid malnutrition and sarcopenia: Older adults with cirrhosis are at high risk for muscle loss, which worsens prognosis. Aim for a high-calorie, moderate-protein, low-sodium diet with emphasis on plant-based proteins and fiber.
- Key principles:
  - Caloric adequacy: Target 30–35 kcal per kg of *ideal body weight* (IBW) daily to prevent catabolism. For her height (155 cm), IBW is ~50 kg (using Devine formula: 45.5 kg + 2.3 kg per inch over 5 ft; she is 5'1", so ~50 kg). Thus, total calories: 1,500–1,750 kcal/day.
  - Sodium restriction: Limit to <2,000 mg (2 g) per day to reduce ascites risk, common in cirrhosis.
  - Alcohol avoidance: Strictly eliminate all alcohol, as it accelerates liver damage in MASLD.
  - Hydration: Maintain normal fluid intake (1.5–2 L/day) unless hyponatremia is present; excessive restriction is unnecessary and may worsen constipation.

**2. Recommended Daily Protein Intake**

Protein intake must balance sarcopenia prevention with HE risk:

- Target: 1.0–1.2 g per kg of ideal body weight (IBW) per day, equating to 50–60 g of protein daily (based on IBW of 50 kg).
  - *Rationale*: Higher protein (e.g., 1.2–1.5 g/kg) is standard for cirrhosis to combat muscle loss, but her HE history warrants cautious initiation. Start at 1.0 g/kg (50 g/day) for 2–4 weeks; if no HE symptoms recur, gradually increase to 1.2 g/kg (60 g/day). Avoid exceeding 1.2 g/kg without specialist oversight.
- Protein distribution: Prioritize plant-based and dairy proteins (e.g., lentils, tofu, Greek yogurt), which produce less ammonia than animal proteins. Limit red meat; include fish (e.g., salmon) for omega-3 anti-inflammatory benefits.
- Monitoring: Assess for HE signs (e.g., confusion, fatigue) weekly. If symptoms emerge, temporarily reduce protein to 0.8 g/kg and consult a hepatologist—do not self-adjust.

**3. Meal Frequency and Eating Pattern**

- Pattern: 4–6 small meals/snacks daily, spaced no more than 3–4 hours apart (e.g., breakfast, mid-morning snack, lunch, afternoon snack, dinner, evening snack).
  - *Why?* Prevents prolonged fasting, which triggers muscle breakdown and ammonia release. Nighttime protein snacks (e.g., casein-rich foods like cottage cheese) may reduce overnight catabolism.
- Practical example:
  - 7:00 AM: Oatmeal with chia seeds + 1/2 cup berries.
  - 10:00 AM: Small apple with 10 almonds.
  - 1:00 PM: Lentil soup + side salad (low-sodium dressing).
  - 4:00 PM: Greek yogurt (unsweetened).
  - 7:00 PM: Baked salmon (3 oz) + roasted vegetables + quinoa (1/2 cup).
  - 9:00 PM: Casein protein shake (20 g protein) or warm milk (if tolerated).
- Avoid: Large, infrequent meals (>500 kcal/protein-heavy), which can spike ammonia levels.

**4. Specific Food Recommendations or Restrictions**

**Recommended Foods**:

- Proteins: Plant-based (tofu, tempeh, lentils, chickpeas), egg whites, low-fat dairy (Greek yogurt, cottage cheese), and lean poultry/fish (baked or steamed; max 3 oz/meal). Aim for 50–60% of protein from plants.
- Carbohydrates: Whole grains (oats, quinoa, brown rice), vegetables (broccoli, spinach, carrots), and fruits (berries, apples—high in fiber). These provide fiber to prevent constipation (a HE trigger) and antioxidants for liver health.
- Fats: Unsaturated fats only—avocado, olive oil, nuts (almonds, walnuts; 1 oz/day). Limit to 40–50 g total fat/day to manage MASLD.
- Sodium-safe seasonings: Herbs, lemon juice, garlic, vinegar; avoid salt substitutes high in potassium (risk in renal impairment).

**Strict Restrictions**:

- High-sodium foods: Processed meats (bacon, deli meats), canned soups, soy sauce, chips, and frozen meals (often >500 mg sodium/serving). Read labels; choose "low-sodium" (<140 mg/serving) options.
- Animal proteins: Red meat, pork, and organ meats (high in ammonia precursors); limit to <2 oz/week if included.
- Added sugars and refined carbs: Sodas, pastries, white bread (worsen insulin resistance in MASLD).
- Constipation-promoting foods: Low-fiber items (white rice, bananas in excess); ensure 25–30 g fiber/day from vegetables/whole grains.
- Alcohol and raw shellfish: Absolute avoidance due to liver toxicity and infection risk.

**5. Additional Nutritional Considerations**

- Vitamin and mineral supplementation:
  - Cirrhosis impairs absorption of fat-soluble vitamins (A, D, E, K). Recommend a daily liver-specific multivitamin (e.g., containing 800–1,000 IU vitamin D, 10 mg vitamin B complex, and 10 mg vitamin K1). Avoid high-dose iron or vitamin A supplements without testing.
  - Vitamin D: Critical for muscle function; check levels—supplement with 1,000–2,000 IU/day if deficient (common in elderly cirrhosis patients).
- HE-specific strategies:
  - Fiber intake: 25–30 g/day from vegetables, fruits, and whole grains to promote regular bowel movements (goal: 1–2 soft stools/day). If constipation occurs, use psyllium husk (not laxatives long-term).
  - Probiotics: Consider daily fermented foods (e.g., unsweetened kefir) or a probiotic supplement (e.g., *Lactobacillus* strains) to modulate gut flora and reduce ammonia production.
  - Avoid high-risk triggers: Skip protein-rich supplements (e.g., whey) without medical approval; use medical foods like支链氨基酸 (BCAA) formulas only if prescribed for recurrent HE.
- Monitoring and adjustments:
  - Track weight weekly: Sudden gain (>1 kg/week) may indicate fluid retention; loss (>2 kg/month) suggests malnutrition.
  - Consult a dietitian for personalized meal planning and regular assessment of muscle mass (e.g., calf circumference) and HE risk.
  - Emergency signs: If confusion, slurred speech, or extreme fatigue occurs, seek immediate care—do not alter diet independently.

These recommendations are designed to be safe and practical for outpatient management. However, they must be individualized based on her current labs (e.g., albumin, ammonia levels), medication regimen (e.g., lactulose for HE prophylaxis), and tolerance. I strongly advise collaboration with her hepatologist and a registered dietitian specializing in liver disease for ongoing support. Nutritional therapy is a cornerstone of cirrhosis care—it can significantly improve quality of life and reduce hospitalizations when implemented consistently.
