# Supplementary material for: Guideline adherence and expert-rated potential safety concerns in AI-generated dietary advice for patients with cirrhosis: a comparative evaluation of large language models
Source: Front Nutr. 2026 Oct 8;13:1893597. doi: 10.3389/fnut.2026.1893597 (PMC13689687; doi:10.3389/fnut.2026.1893597)
Supplement: Supplementary file 1 [file Supplementary_file_1.zip › Supplementary Data S1 (Verbatim Model Responses).ZIP!/answer via chatgpt.docx]

# C1-01

For this **34-year-old man, 172 cm/68 kg, BMI ≈ 23.0 kg/m²**, with **HBV-related compensated cirrhosis, no ascites, no encephalopathy, and normal appetite**, the goal is to **maintain muscle mass, prevent malnutrition/sarcopenia, avoid unnecessary restriction, and support stable liver function**.

**1. Overall dietary principles**

Use a **balanced, high-protein, adequate-calorie diet** rather than a “light” or low-protein diet. Patients with cirrhosis are prone to accelerated fasting metabolism and muscle loss, even when compensated.

Recommended daily energy target: **30–35 kcal/kg/day**, about **2,040–2,380 kcal/day** for his current weight, adjusted by weight trend, activity level, and body composition. This range is consistent with cirrhosis nutrition guidance recommending adequate calories and protein.

Diet pattern: mostly **Mediterranean-style / whole-food-based**: grains or starchy foods, vegetables, fruit, lean protein, dairy or soy products, legumes, nuts, and healthy oils.

Avoid:

- Alcohol completely.
- “Detox” diets, prolonged fasting, very low-carbohydrate dieting, or unnecessary food restriction.
- Raw or undercooked seafood, especially raw shellfish, because cirrhosis increases risk of severe foodborne infection.

**2. Recommended daily protein intake**

Target **1.2–1.5 g/kg/day**.

For **68 kg**:

**Protein goal: ~82–102 g/day**

A practical target is **90 g/day**, divided across the day. Current AASLD and ESPEN-aligned guidance recommends **1.2–1.5 g/kg/day** and emphasizes that protein should **not** be restricted, even in many patients with hepatic encephalopathy; this patient has no encephalopathy, so there is no indication for protein restriction.

Practical distribution:

- Breakfast: 20–25 g
- Lunch: 25–30 g
- Dinner: 25–30 g
- Late snack: 10–20 g

Examples of ~20–30 g protein:

- 2 eggs + milk/soy milk
- 100–120 g fish/chicken/lean meat
- 150–200 g tofu
- 250 g Greek yogurt
- Beans/lentils plus rice or whole grains

**3. Meal frequency and eating pattern**

Recommend **4–6 eating occasions daily**, avoiding fasting periods longer than about **3–4 hours while awake**.

A **late-evening snack** is especially useful in cirrhosis to reduce overnight catabolism and protect muscle. Guidance supports small frequent meals plus an early breakfast and/or late-night high-protein snack.

Suggested pattern:

- Breakfast
- Lunch
- Afternoon snack
- Dinner
- Late-evening snack before bed

Good late-evening snack options:

- Milk or soy milk + whole-grain bread
- Greek yogurt + fruit
- Oatmeal with milk
- Tofu pudding / soy products
- Egg + toast
- Rice, noodles, or congee plus tofu/egg/fish
- Protein-containing oral nutrition supplement if intake is inadequate

The bedtime snack should include **carbohydrate plus protein**, not just fruit or tea.

**4. Specific food recommendations or restrictions**

**Encourage**

**Protein foods**

- Fish, poultry, eggs
- Milk, yogurt, cheese if tolerated
- Soy milk, tofu, tempeh
- Beans, lentils, chickpeas
- Lean pork/beef in moderation
- Nuts/seeds as snacks if weight is stable

**Carbohydrates**

- Rice, noodles, oats, potatoes, whole-grain bread
- Avoid extreme low-carb diets; carbohydrates help reduce protein breakdown during fasting.

**Vegetables and fruit**

- Daily vegetables, ideally at least 2 servings/day
- Fruit 1–2 servings/day, adjusted if diabetes or hyperglycemia is present

**Fats**

- Use moderate amounts of unsaturated fats: olive oil, canola oil, nuts, seeds, avocado
- Avoid frequent fried foods and trans fats

**Sodium / salt**

Because he has **no ascites or edema**, a strict sodium restriction is **not required**. Recommend a generally healthy salt intake:

- Avoid very salty processed foods, pickles, preserved meats, instant noodles, heavily salted sauces.
- Do not impose severe salt restriction unless ascites, edema, or hypertension develops.

If ascites appears later, sodium restriction is usually needed, commonly around **2 g sodium/day**, but that is not necessary for this current compensated case.

**Avoid or limit**

- **Alcohol: zero**
- Raw shellfish, raw seafood, undercooked meat/eggs
- Herbal remedies or supplements with unclear ingredients, especially “liver detox” products
- High-dose vitamin A, niacin, green tea extract, bodybuilding supplements, or unverified traditional medicines unless reviewed for hepatotoxicity
- Sugary beverages and excess refined snacks, especially if fatty liver, insulin resistance, or weight gain develops

**5. Additional nutritional considerations**

Monitor for **sarcopenia**, not just body weight. In cirrhosis, weight can look normal while muscle is declining. Track:

- Weight trend
- Appetite and intake
- Handgrip strength or functional status
- Muscle mass clinically or by imaging if available
- Albumin/prealbumin are not reliable standalone nutrition markers in liver disease

Check and supplement if deficient:

- **Vitamin D**
- Calcium intake / bone health
- Zinc if poor appetite, dysgeusia, diarrhea, or encephalopathy risk
- B vitamins if dietary intake is poor
- Fat-soluble vitamins if cholestasis is present

Exercise should accompany nutrition:

- Regular walking or aerobic activity
- Resistance training 2–3 times/week if medically safe
  Nutrition plus resistance exercise is more effective for preserving muscle than diet alone.

**Simple daily example**

- **Breakfast:** oatmeal with milk/soy milk + egg
- **Snack:** yogurt or soy milk + fruit
- **Lunch:** rice/noodles + fish/chicken/tofu + vegetables
- **Snack:** nuts or milk + whole-grain bread
- **Dinner:** lean protein + staple carbohydrate + vegetables
- **Before bed:** yogurt, milk/soy milk, tofu, or egg with toast/congee

**Bottom line:** For this stable compensated cirrhosis patient, prescribe **adequate calories, 82–102 g protein/day, 4–6 meals/snacks daily, and a protein-containing late-evening snack**, with **no protein restriction and no strict sodium restriction unless ascites develops**.

窗体顶端窗体底端

# C1-02

**Patient summary**

39-year-old woman, 160 cm, 54 kg, BMI ≈ **21.1 kg/m²**. She has **MASLD-related compensated cirrhosis**, stable, and can eat normally. The goal is to **maintain muscle mass, prevent malnutrition/sarcopenia, support liver health, and avoid MASLD progression**.

**1. Overall dietary principles**

Use a **balanced, high-protein Mediterranean-style diet**, not a “low-protein liver diet.” In cirrhosis, protein restriction is no longer recommended because it can worsen muscle loss; guidelines recommend **1.2–1.5 g/kg/day** protein to preserve or restore muscle mass.

Daily targets for her:

| **Target** | **Practical goal** |
| --- | --- |
| Energy | **~1,600–1,900 kcal/day** |
| Protein | **~65–80 g/day** |
| Meals | **3 meals + 2–3 snacks**, including late-evening snack |
| Weight goal | Maintain current healthy weight; avoid both weight gain and unintentional loss |

For MASLD, emphasize **unsaturated fats, whole grains, legumes, vegetables, fruit, nuts, fish**, and minimize **sugary drinks, refined carbohydrates, ultra-processed foods, and excess saturated fat**.

**2. Recommended daily protein intake**

Recommended protein: **1.2–1.5 g/kg/day × 54 kg = 65–81 g/day**.

A practical prescription: **70–80 g protein/day**, spread across the day.

Example distribution:

| **Time** | **Protein goal** |
| --- | --- |
| Breakfast | 15–20 g |
| Lunch | 20–25 g |
| Dinner | 20–25 g |
| Snacks | 10–20 g total |

Good protein choices include **fish, poultry, eggs, low-fat dairy or Greek yogurt, tofu, soy milk, beans, lentils, chickpeas, nuts, and seeds**. Vegetable and dairy proteins are often well tolerated in cirrhosis, and plant proteins also help with fiber intake.

**3. Meal frequency and eating pattern**

Avoid long fasting periods. Cirrhosis causes a “fasting-like” metabolism overnight, increasing risk of muscle breakdown. EASL and ESPEN guidance support **breakfast with protein and a late-evening snack** to shorten fasting time.

Recommended pattern:

**Breakfast within 1–2 hours of waking**
**Lunch**
**Afternoon snack**
**Dinner**
**Late-evening snack before bed**

Late-evening snack target: **carbohydrate + protein**, for example:

- Greek yogurt + berries
- Milk or unsweetened soy milk + whole-grain toast
- Oatmeal with nuts
- Whole-grain crackers + hummus
- Banana + peanut butter
- Tofu pudding or soy yogurt

**4. Specific food recommendations and restrictions**

**Encourage**

**Protein at every meal:** eggs, fish, chicken, tofu, tempeh, beans, lentils, Greek yogurt, milk, soy milk.

**High-fiber carbohydrates:** oats, brown rice, quinoa, whole-wheat bread, sweet potato, beans, lentils.

**Healthy fats:** olive oil, avocado, nuts, seeds, fatty fish.

**Vegetables and fruit daily:** aim for a variety; choose whole fruit rather than juice.

**Coffee is reasonable** if tolerated and not contraindicated. Regular coffee intake has been associated with lower risk of liver fibrosis progression in chronic liver disease, though it should not replace standard care.

**Limit or avoid**

**Alcohol: avoid completely.** In cirrhosis, alcohol abstinence is recommended regardless of the original cause of liver disease.

**Sugary drinks and fructose-heavy foods:** soda, sweet tea, fruit juice, energy drinks, desserts, sweetened coffee drinks.

**Ultra-processed foods:** chips, processed meats, packaged sweets, fast food.

**Excess saturated fat:** fatty meats, butter-heavy foods, cream-based sauces, deep-fried foods.

**Raw or undercooked shellfish**, especially oysters, because cirrhosis increases the risk of severe Vibrio infection.

**Unnecessary herbal supplements or “liver detox” products.** Many are unregulated and may be hepatotoxic.

**Sodium**

Because she is **compensated** and no ascites is mentioned, she does **not** need extreme sodium restriction. A practical goal is to avoid very salty foods and keep sodium moderate, roughly **≤2,000–2,300 mg/day**. If ascites or edema develops, sodium restriction becomes more important, typically around **2 g/day**, but overly restrictive diets can reduce intake and worsen nutrition. ESPEN cautions that sodium reduction should not compromise palatability and intake.

**5. Additional nutritional considerations**

Screen periodically for **sarcopenia and malnutrition**, even at a normal BMI. Malnutrition can occur in compensated cirrhosis, and sarcopenia may be missed if weight appears normal.

Check and replete as needed:

- **Vitamin D**
- **Calcium**
- **Zinc**, especially if poor appetite, dysgeusia, or encephalopathy concerns
- **B12/folate/iron** if anemia or dietary risk
- Fat-soluble vitamins if cholestasis is present

Pair diet with **regular resistance exercise plus aerobic activity**, as tolerated, because nutrition and strength training together are most effective for preserving muscle.

**Practical one-day example**

**Breakfast:** oatmeal with milk or soy milk, nuts, berries, plus boiled egg
**Snack:** Greek yogurt or soy yogurt
**Lunch:** brown rice or quinoa bowl with grilled fish/chicken/tofu, vegetables, olive oil dressing
**Snack:** fruit + nuts or hummus with whole-grain crackers
**Dinner:** lentil soup or chicken/fish/tofu, vegetables, sweet potato or whole grain
**Late snack:** milk/soy milk + whole-grain toast with peanut butter, or yogurt with fruit

**Bottom line:** For this patient, prescribe **normal-calorie, high-protein, Mediterranean-style eating**, aiming for **70–80 g protein/day**, **3 meals plus snacks**, and a **late-evening snack**, while avoiding alcohol, sugary drinks, ultra-processed foods, and unnecessary supplements.

# C1-03

**C1-03: 42-year-old man, BMI ~29.1, alcohol-related compensated cirrhosis**

**1. Overall dietary principles**

For compensated cirrhosis, the main goals are to **preserve muscle mass, avoid fasting, prevent malnutrition/sarcopenia, support gradual fat loss if needed, and maintain alcohol abstinence**. Cirrhosis is a catabolic state, so routine “low-protein liver diets” are **not recommended**. Current EASL/AASLD guidance supports adequate calories, high protein, frequent meals, and a late-evening snack.

For this patient, BMI is about **29 kg/m²**, so he is overweight. Weight reduction can be considered, but it should be **slow and protein-preserving**, not by skipping meals or crash dieting.

**2. Recommended daily protein intake**

Use **ideal body weight or estimated dry weight**. For a 168 cm male, ideal body weight is roughly **64 kg**.

Recommended protein: **1.2–1.5 g/kg/day**
Practical target: **~80–95 g protein/day**.

This can be divided as:

| **Time** | **Protein target** |
| --- | --- |
| Breakfast | 20–25 g |
| Lunch | 25–30 g |
| Dinner | 25–30 g |
| Late snack | 10–20 g |

Protein should **not** be restricted, even in many patients with hepatic encephalopathy; instead, source and timing are adjusted.

**3. Meal frequency and eating pattern**

Recommend **4–6 eating occasions per day**, avoiding fasting longer than about **3–4 hours while awake**. AASLD guidance emphasizes avoiding prolonged fasting and using early breakfast plus a late-evening high-protein snack.

Practical pattern:

- **Breakfast within 1 hour of waking**
- **Lunch**
- **Afternoon snack**
- **Dinner**
- **Late-evening snack before bed**

Good late-evening snack examples:

- Greek yogurt + fruit
- Milk or soy milk + whole-grain toast
- Cottage cheese + crackers
- Peanut butter on whole-grain bread
- Protein-fortified oral supplement, if appetite is poor
- Oatmeal made with milk or soy milk

The late snack should include **carbohydrate plus protein**, not just tea or fruit.

**4. Specific food recommendations and restrictions**

**Recommended protein sources**

Prioritize a mix of:

- Fish, poultry, eggs
- Low-fat dairy: milk, yogurt, Greek yogurt, cottage cheese
- Soy foods: tofu, soy milk, edamame
- Beans, lentils, chickpeas
- Nuts and nut butters
- Lean meats in moderation

Vegetable and dairy proteins are often well tolerated and are useful in cirrhosis.

**Carbohydrates**

Choose mostly complex carbohydrates:

- Oats, brown rice, whole-grain bread, potatoes, sweet potatoes
- Beans and lentils
- Fruits and vegetables

Avoid very high sugar intake, especially sweetened drinks, because he is overweight and may have insulin resistance risk.

**Fats**

Use mostly unsaturated fats:

- Olive oil, canola oil
- Avocado
- Nuts and seeds
- Fatty fish

Limit fried foods, processed meats, and large portions of saturated fat.

**Salt/sodium**

Because this case is **compensated cirrhosis with no ascites mentioned**, a strict 2 g sodium diet is not automatically required. Still, advise avoiding excessive sodium:

- Avoid frequent processed foods, instant noodles, cured meats, salty snacks, fast food
- Do not add large amounts of salt or high-sodium sauces
- If ascites or edema develops, then sodium restriction to about **2 g/day sodium** is usually needed under clinician guidance.

**Alcohol**

Complete **alcohol abstinence** is essential. In alcohol-related liver disease, ongoing alcohol use worsens liver injury and nutritional status; abstinence is a core part of treatment.

**Avoid**

- Alcohol in any amount
- Raw or undercooked shellfish
- Unnecessary herbal supplements or bodybuilding supplements
- Excess vitamin A, high-dose niacin, or unverified “liver detox” products
- Skipping breakfast or prolonged fasting

**5. Additional nutritional considerations**

Because this is alcohol-related cirrhosis, assess and supplement when needed:

- **Thiamine**, especially if recent alcohol intake or poor diet
- **Folate and B12**
- **Vitamin D** and calcium; consider bone health screening depending on clinical context
- **Magnesium, zinc, phosphate** if intake is poor or there are symptoms/biochemical abnormalities
- Screen for **sarcopenia** even if overweight; BMI can hide muscle loss.
- Encourage **resistance exercise** plus walking if medically safe, because nutrition works best when paired with muscle stimulation.
- Consider referral to a **liver dietitian** for individualized calorie planning.

A practical daily goal for him: **80–95 g protein/day, 4–6 meals/snacks, late-evening protein-carbohydrate snack, no alcohol, minimally processed Mediterranean-style diet, and slow weight optimization without fasting.**

# C1-04

**C1-04: Nutrition plan for 44-year-old woman with compensated HBV cirrhosis**

**Patient profile:** 158 cm, 70 kg, BMI ≈ **28.0 kg/m²**, so she is overweight. She has **compensated cirrhosis** and is stable as an outpatient.

**1. Overall dietary principles**

For compensated cirrhosis, the goal is **adequate energy and high-quality protein to prevent sarcopenia**, while avoiding excessive calories that worsen obesity or fatty liver risk. Cirrhosis is a “catabolic” state, so prolonged fasting should be avoided. Current EASL and AASLD guidance supports **high-protein intake, small frequent meals, and a late-evening snack** rather than protein restriction.

A practical daily energy target is about **25–30 kcal/kg/day using actual weight if weight is stable**, roughly **1,750–2,100 kcal/day**. Because her BMI is elevated, aim for the lower-to-middle end unless she is losing muscle or weight unintentionally. Weight loss, if desired, should be **slow and supervised**, with resistance exercise and enough protein to preserve muscle.

**2. Recommended daily protein intake**

Recommended protein for cirrhosis is **1.2–1.5 g/kg/day**. For this patient:

- Using actual body weight 70 kg: **84–105 g protein/day**
- Practical target: **90 g/day**, divided across meals and snacks

Do **not** restrict protein solely because she has cirrhosis. Even in hepatic encephalopathy, modern guidance generally avoids chronic protein restriction and instead adjusts source and timing.

Good distribution: about **20–25 g protein per main meal** plus **10–15 g protein in snacks**.

**3. Meal frequency and eating pattern**

Recommend **3 meals + 2–3 snacks daily**, eating every **3–4 hours while awake**. Avoid fasting longer than about **10–12 hours overnight**. AASLD guidance specifically recommends minimizing fasting intervals and using an early breakfast and/or late-night high-protein snack.

Suggested pattern:

- Breakfast within 1 hour of waking
- Lunch
- Afternoon snack
- Dinner
- **Late-evening snack** before bed

Late-evening snack examples:

- Greek yogurt or milk + whole-grain crackers
- Soy milk + steamed bun or oatmeal
- Egg + slice of whole-grain toast
- Tofu pudding or unsweetened soy yogurt + fruit
- Small bowl of oatmeal with milk
- Peanut butter on toast, if tolerated

A bedtime snack with carbohydrate and protein helps reduce overnight muscle breakdown; bedtime carbohydrate snacks around 50 g carbohydrate have been studied in cirrhosis.

**4. Specific food recommendations and restrictions**

**Encourage:**

- **Protein:** fish, poultry, eggs, lean meat, tofu, soy milk, beans/lentils, yogurt, milk, cheese in moderation
- **Plant and dairy proteins:** useful options, especially if meat tolerance is poor
- **Carbohydrates:** rice, oats, whole-grain bread, potatoes, corn, noodles in controlled portions
- **Vegetables and fruit:** daily, emphasizing fiber and micronutrients
- **Fats:** moderate amounts from olive/canola oil, nuts, seeds, avocado; avoid excess fried foods
- **Fluids:** normal hydration unless ascites, hyponatremia, or renal issues develop

**Limit or avoid:**

- **Alcohol: complete abstinence**
- Raw or undercooked seafood, especially raw shellfish, due to infection risk in chronic liver disease
- Highly processed, very salty foods: pickles, cured meats, instant noodles, chips, canned soups
- Sugary beverages and desserts, especially given BMI 28
- Herbal supplements or “liver detox” products unless cleared by her hepatology team

**Salt:** Since this is **compensated cirrhosis** with no ascites mentioned, she does not need an extremely restrictive diet. Still, avoid high-salt foods. If ascites or edema appears, use a stricter sodium target, commonly **≤2 g sodium/day**. AASLD outpatient cirrhosis guidance highlights sodium restriction particularly for ascites management.

**5. Additional nutritional considerations**

Monitor for **sarcopenia** even if BMI is high; overweight patients with cirrhosis can still have low muscle mass. Track handgrip strength, walking speed, muscle symptoms, and weight trend. Consider dietitian follow-up.

Check and replete common deficiencies as clinically indicated: **vitamin D, calcium, zinc, folate, B12, iron studies**, and fat-soluble vitamins if cholestasis is present.

Encourage **regular physical activity**, especially resistance training 2–3 times weekly plus walking, if medically safe. Nutrition plus exercise is more effective for preserving muscle than diet alone.

For HBV-related cirrhosis, continue antiviral and hepatology follow-up, HCC surveillance, and variceal screening as indicated; nutrition supports but does not replace disease-specific management.

# C1-05

**Patient profile**

Male, 47 years; 175 cm, 92 kg. BMI ≈ **30 kg/m²**, consistent with obesity. He has **MASLD-related compensated cirrhosis**, so the nutrition goal is **fat loss without muscle loss**.

**1. Overall dietary principles**

Use a **Mediterranean-style, high-protein, calorie-controlled diet**:

Aim for **gradual weight loss**, about **5–10% body weight over 6–12 months**, while preserving skeletal muscle. For this patient, an initial target is roughly **5–9 kg loss**, not rapid dieting.

Avoid “liver detox,” fasting, very-low-carbohydrate crash diets, or unnecessary protein restriction. Modern cirrhosis guidance emphasizes adequate protein and avoidance of prolonged fasting; AASLD notes a protein goal of **1.2–1.5 g/kg ideal body weight** and meals/snacks every **3–4 hours** with a late-night protein snack.

**2. Recommended daily protein intake**

Use **ideal/adjusted body weight**, not actual body weight, because BMI is around 30.

Estimated ideal body weight for 175 cm male: about **70–75 kg**.

Recommended protein: **1.2–1.5 g/kg/day**
Practical target: **90–110 g protein/day**

A simple distribution:

| **Eating time** | **Protein target** |
| --- | --- |
| Breakfast | 20–25 g |
| Lunch | 25–30 g |
| Dinner | 25–30 g |
| Snacks / late evening | 15–25 g |
| **Total** | **90–110 g/day** |

Protein should **not** be restricted, even if hepatic encephalopathy develops; instead, adjust sources and timing. Vegetable and dairy proteins are often useful in cirrhosis.

**3. Meal frequency and eating pattern**

Recommend **3 meals + 2–3 snacks daily**, avoiding fasting longer than **3–4 hours while awake**.

Most important: add a **late-evening snack** before bed to reduce overnight catabolism. EASL and AASLD-aligned guidance recommend small frequent meals and a late snack to shorten nocturnal fasting in cirrhosis.

Good late-evening snack options:

| **Option** | **Example** |
| --- | --- |
| Dairy + carbohydrate | Greek yogurt + berries / oats |
| Soy protein | Unsweetened soy milk + whole-grain toast |
| Legume-based | Hummus + whole-grain pita |
| Protein + complex carb | Cottage cheese + fruit |
| MASLD-friendly | Low-fat yogurt + chia + small portion oats |

Avoid skipping breakfast. A practical daily schedule: **breakfast, lunch, afternoon snack, dinner, bedtime snack**.

**4. Specific food recommendations and restrictions**

**Prefer**

**Protein foods**

- Fish, chicken, turkey, eggs
- Low-fat Greek yogurt, milk, kefir, cottage cheese
- Tofu, tempeh, edamame, beans, lentils, chickpeas
- Nuts in small portions

**Carbohydrates**

- Oats, barley, brown rice, quinoa, whole-grain bread
- Beans and lentils
- Fruit, especially whole fruit rather than juice
- High-fiber vegetables at most meals

**Fats**

- Olive oil as main added fat
- Nuts, seeds, avocado in controlled portions
- Fatty fish 2 times/week if feasible

**Beverages**

- Water, unsweetened tea/coffee
- Coffee is reasonable if tolerated and no contraindication; it is commonly encouraged in MASLD because observational data associate coffee intake with lower liver fibrosis progression risk.

**Limit or avoid**

- **Alcohol: complete abstinence**
- Sugar-sweetened beverages, fruit juice, sweet tea, soda
- Fructose-heavy foods: desserts, candy, sweetened cereals
- Ultra-processed foods, fast food, processed meats
- Large portions of white rice, white bread, pastries, refined noodles
- Saturated fat-heavy foods: fatty meats, butter, cream, fried foods

**Sodium**

Because this is **compensated cirrhosis** and no ascites is mentioned, strict sodium restriction is not automatically required. Still, a reasonable target is **≤2,000–2,300 mg sodium/day**, especially to prevent fluid retention and support cardiometabolic health. If ascites or edema develops, use the stricter **≤2,000 mg/day** target.

**5. Additional nutritional considerations**

**Calorie target:** For weight loss, start with a modest deficit, often around **500 kcal/day below maintenance**, while maintaining protein. Avoid rapid weight loss because it may worsen sarcopenia or gallstone risk.

**Exercise pairing:** Nutrition should be paired with resistance training. Recommend **2–3 sessions/week of strength training** plus regular walking or aerobic activity, as tolerated. This is especially important because cirrhosis increases sarcopenia risk.

**Screen for sarcopenia and micronutrient deficits:** Check or consider:

- Handgrip strength, muscle mass, frailty assessment
- Vitamin D
- Zinc if poor intake, dysgeusia, or encephalopathy risk
- B12/folate/iron studies if anemia or restrictive diet
- Diabetes, lipids, blood pressure, and renal function

**For MASLD:** Prioritize weight loss through Mediterranean-style eating, physical activity, and control of diabetes, dyslipidemia, and hypertension.

**Sample one-day pattern**

| **Time** | **Meal** |
| --- | --- |
| Breakfast | Oatmeal + Greek yogurt + berries; or eggs/tofu scramble with vegetables |
| Lunch | Grilled fish/chicken/tofu + quinoa/brown rice + large salad with olive oil |
| Snack | Apple + low-fat yogurt, or hummus with vegetables |
| Dinner | Lentil/bean soup or chicken/fish + vegetables + small whole-grain portion |
| Bedtime snack | Greek yogurt with oats, or soy milk + whole-grain toast |

**Bottom line:** For this patient, prescribe a **Mediterranean-style, calorie-controlled diet with 90–110 g protein/day**, spread across **4–6 eating occasions**, including a **protein-containing bedtime snack**, with alcohol abstinence and avoidance of crash dieting.

# C1-06

**Patient-specific targets**

51-year-old woman, 162 cm, 60 kg; BMI ≈ **22.9 kg/m²**, so she is normal weight. For compensated alcohol-related cirrhosis, the key goal is **preventing sarcopenia and malnutrition**, even when body weight looks normal.

**1. Overall dietary principles**

Use a **high-protein, adequate-calorie, Mediterranean-style diet**:

- **Energy:** about **30–35 kcal/kg/day**, roughly **1,800–2,100 kcal/day** for her, adjusted to weight trend, appetite, and activity.
- **Protein should not be restricted** in cirrhosis; current guidance recommends adequate-to-high protein to prevent muscle loss.
- Emphasize **whole grains, legumes, vegetables, fruits, dairy/soy, fish, eggs, poultry, nuts, and olive oil**.
- **Complete alcohol abstinence** is essential in alcohol-related cirrhosis; ongoing drinking worsens liver injury and nutritional status.

**2. Recommended daily protein intake**

Recommended protein intake: **1.2–1.5 g/kg/day**.

For her weight of **60 kg**:

- **Minimum target:** 60 × 1.2 = **72 g/day**
- **Higher target:** 60 × 1.5 = **90 g/day**

Practical goal: **75–90 g protein/day**, spread across the day.

Example distribution:

- Breakfast: 20 g
- Lunch: 20–25 g
- Dinner: 25–30 g
- Snacks: 10–20 g total

Good protein choices include **fish, eggs, poultry, Greek yogurt, milk, tofu, soy milk, beans/lentils, nuts/nut butters, and cheese/cottage cheese**. Vegetable and dairy protein are often well tolerated in cirrhosis, including in patients at risk of encephalopathy.

**3. Meal frequency and eating pattern**

Avoid prolonged fasting. Patients with cirrhosis enter a “starvation-like” state faster overnight, which promotes muscle breakdown.

Recommended pattern:

- **3 meals + 2–3 snacks daily**
- **Breakfast soon after waking**
- **Late-evening snack before bed**, ideally containing carbohydrate plus protein

Examples of bedtime snacks:

- Greek yogurt with fruit
- Milk or fortified soy milk plus whole-grain crackers
- Peanut butter toast
- Cottage cheese with fruit
- Oatmeal made with milk
- Protein smoothie with yogurt or soy milk

Small frequent meals and a late-night snack are specifically recommended in cirrhosis nutrition guidance.

**4. Specific food recommendations or restrictions**

**Recommended foods**

- **Protein:** fish, chicken, eggs, tofu, tempeh, beans, lentils, Greek yogurt, milk, soy milk, cheese, nuts.
- **Carbohydrates:** oats, brown rice, whole-grain bread, potatoes, sweet potatoes, quinoa, fruit.
- **Fats:** olive oil, avocado, nuts, seeds.
- **Vegetables:** aim for a variety daily; include leafy greens, cruciferous vegetables, carrots, peppers, tomatoes.
- **Fiber:** legumes, oats, vegetables, fruit; helps bowel regularity and metabolic health.

**Alcohol**

- **No alcohol at all**, including “small amounts,” cooking wine, and binge episodes.

**Salt / sodium**

- Since she has **compensated cirrhosis** and no ascites is mentioned, she does **not automatically need a severe sodium restriction**.
- A reasonable approach is to avoid very salty processed foods and aim for a generally heart-healthy sodium intake.
- If ascites, edema, or hypertension develops, typical sodium restriction is about **2 g sodium/day**, but this should be individualized.

**Avoid or limit**

- Raw oysters, raw shellfish, and unsafe raw seafood because cirrhosis increases risk from foodborne infections.
- Highly processed meats, fast food, salty snacks, instant noodles, and very high-sodium canned foods.
- Sugary drinks and large amounts of sweets, especially if insulin resistance or diabetes is present.
- Unregulated herbal supplements, “liver detox” products, high-dose green tea extract, kava, and other potentially hepatotoxic supplements.
- Excess vitamin A or niacin unless specifically prescribed.

**5. Additional nutritional considerations**

- **Screen for sarcopenia even if BMI is normal.** Ask about declining strength, low activity, falls, poor appetite, or unintentional weight loss.
- **Resistance exercise** is strongly helpful if medically safe: light weights, bands, chair squats, or supervised strength training 2–3 times/week, plus walking.
- **Micronutrients in alcohol-related liver disease:** check and replete as needed:
  - Thiamine, folate, vitamin B12
  - Vitamin D
  - Calcium intake, especially in postmenopausal women
  - Magnesium, zinc, and phosphate if intake is poor or there is prior heavy alcohol use
- **Bone health:** cirrhosis increases osteoporosis risk; consider vitamin D level, calcium intake, and DEXA screening per local practice.
- **Vaccination and food safety counseling** are relevant, especially hepatitis A/B vaccination if nonimmune.
- **If appetite is low:** use oral nutrition supplements, fortified milk/soy milk, smoothies, nut butter, olive oil, and protein-rich snacks rather than skipping meals.
- **If hepatic encephalopathy develops:** do **not** chronically restrict protein; instead optimize lactulose/rifaximin if prescribed and favor vegetable/dairy proteins if animal protein worsens symptoms. Protein restriction is no longer recommended as routine care.

**Practical one-day example**

Breakfast: oatmeal made with milk or soy milk + Greek yogurt + berries
Snack: boiled egg or tofu snack + fruit
Lunch: rice or whole-grain bread + lentils/beans or chicken/fish + vegetables + olive oil
Snack: nuts + yogurt or milk
Dinner: fish/tofu/chicken + potatoes or rice + vegetables
Bedtime snack: peanut butter toast or Greek yogurt with fruit

Daily goal: **1,800–2,100 kcal, 75–90 g protein, no alcohol, frequent meals, and a bedtime snack.**

# C1-07

For this 55-year-old man with **HBV-related compensated cirrhosis**, BMI ≈ **22.1 kg/m²** and weight **64 kg**, the main nutrition goal is to **maintain muscle mass, prevent malnutrition/sarcopenia, and avoid long fasting periods**.

**1. Overall dietary principles**

Use a **balanced, high-protein, adequate-energy diet**, not a “liver-resting” low-protein diet. In cirrhosis, inadequate intake and overnight fasting can accelerate muscle breakdown; guidelines recommend adequate calories, adequate protein, frequent meals, and a late-evening snack.

Estimated daily energy target: **30–35 kcal/kg/day**, roughly **1,900–2,250 kcal/day** for 64 kg, adjusted based on weight trend, appetite, activity, and body composition.

**2. Recommended daily protein intake**

Recommended protein: **1.2–1.5 g/kg/day**.

For this patient:
**64 kg × 1.2–1.5 = about 77–96 g protein/day**.

A practical target is **80–95 g/day**, divided across meals and snacks. Protein should **not** be restricted in compensated cirrhosis; even in hepatic encephalopathy, modern guidance generally avoids chronic protein restriction and instead adjusts protein sources/timing.

Practical distribution example:

| **Time** | **Protein goal** |
| --- | --- |
| Breakfast | 20–25 g |
| Lunch | 25–30 g |
| Dinner | 25–30 g |
| Snacks/late snack | 10–20 g |

**3. Meal frequency and eating pattern**

Recommend **3 main meals + 2–3 snacks daily**, with a **mandatory late-evening snack**.

Key pattern:

- Eat breakfast; avoid skipping meals.
- Avoid fasting longer than **3–4 hours during the day** when possible.
- Add a **bedtime snack** containing complex carbohydrate plus some protein, because late-evening snacks help shorten overnight fasting in cirrhosis.

Good bedtime snack examples:

- Greek yogurt or milk + oatmeal/whole-grain bread
- Soy milk + steamed bun/whole-grain toast
- Egg + small bowl of congee
- Tofu pudding/soy yogurt + banana
- Oral nutrition supplement if intake is poor

**4. Specific food recommendations and restrictions**

**Recommended foods**

Emphasize:

- **High-quality proteins:** fish, chicken, lean pork/beef in moderation, eggs, milk/yogurt, tofu, soy products, beans/lentils.
- **Plant and dairy proteins** are especially useful and well tolerated in cirrhosis.
- **Complex carbohydrates:** rice, noodles, oats, potatoes, whole grains, corn, beans.
- **Vegetables and fruits daily**, unless restricted for another condition.
- **Healthy fats:** nuts, olive/canola oil, avocado, fatty fish; avoid very greasy meals if dyspepsia occurs.

**Foods/substances to avoid or limit**

- **Alcohol: complete abstinence.**
- **Raw or undercooked seafood, especially raw shellfish**, because cirrhosis increases risk from foodborne infections.
- **Unverified herbal products, “liver detox” supplements, bodybuilding supplements, and high-dose vitamins**, due to hepatotoxicity risk.
- **Very salty processed foods:** pickles, cured meats, instant noodles, chips, salted fish, preserved foods.
  Since he is compensated and no ascites is mentioned, he does **not** need an overly strict sodium diet by default, but should avoid excess salt. If ascites/edema develops, use a stricter sodium target, commonly around **2 g sodium/day** under clinician guidance.
- Avoid unnecessary **protein restriction**.

**5. Additional nutritional considerations**

Monitor for **sarcopenia** even if BMI is normal. Check weight trend, handgrip strength, muscle mass, dietary intake, and activity level. Nutrition plus resistance exercise is important; compensated patients are usually encouraged to do regular walking plus light-to-moderate resistance training if clinically safe.

Screen and replete common deficiencies: **vitamin D, calcium, zinc, folate, B12, iron only if deficient**, and fat-soluble vitamins if cholestasis or malabsorption is present. A standard multivitamin may be reasonable, but avoid high-dose vitamin A, niacin, green-tea extract, and other potentially hepatotoxic supplements unless prescribed.

For HBV cirrhosis, nutrition should accompany standard liver care: antiviral adherence if prescribed, HCC surveillance, avoidance of alcohol, and regular follow-up. Diet cannot reverse cirrhosis by itself, but adequate protein, calories, meal timing, and exercise can reduce frailty and muscle loss.

# C1-08

**Patient-specific assessment**

58-year-old woman, 156 cm, 78 kg: **BMI ≈ 32.1 kg/m²**, consistent with obesity. She has **MASLD-related compensated cirrhosis** with **non-bleeding esophageal varices**. Main nutrition goals are: preserve or increase skeletal muscle, avoid fasting catabolism, support gradual fat loss, reduce cardiometabolic risk, and avoid decompensation.

**1. Overall dietary principles**

Recommend a **high-protein, Mediterranean-style, weight-conscious diet**, not a protein-restricted “liver diet.”

For this patient:

- Aim for **gradual weight loss**, about **5–10% of body weight** if tolerated, while avoiding rapid dieting or muscle loss. That means a practical target of roughly **4–8 kg over 6–12 months**, not crash dieting.
- Use a **Mediterranean-style pattern**: vegetables, legumes, whole grains, fish, low-fat dairy or yogurt, nuts in controlled portions, olive/canola oil, and limited refined carbohydrates.
- Avoid prolonged fasting. In cirrhosis, overnight fasting can resemble accelerated starvation; frequent meals and a late snack are recommended. ESPEN notes that even Child-Pugh A cirrhosis is associated with insulin resistance and altered fasting metabolism.
- **No alcohol.**
- Avoid unnecessary dietary restriction. In compensated cirrhosis without ascites, severe sodium restriction is usually not needed unless there is ascites, edema, uncontrolled hypertension, or clinician-directed sodium restriction.

**2. Recommended daily protein intake**

For stable adults with cirrhosis, AASLD recommends **1.2–1.5 g protein/kg ideal body weight/day**, with **1.5 g/kg/day** favored if sarcopenia is present or suspected. Protein should **not** be restricted even in hepatic encephalopathy; vegetable and dairy protein sources are encouraged.

For her height, an ideal body weight estimate is about **50–54 kg**.

**Practical protein target: 65–80 g/day**
A reasonable prescription is **~75 g/day**, distributed across the day.

Example distribution:

| **Time** | **Protein target** |
| --- | --- |
| Breakfast | 20 g |
| Lunch | 20–25 g |
| Dinner | 25 g |
| Late-evening snack | 10–15 g |

Good protein choices include eggs, fish, chicken, tofu, soy milk, beans/lentils, Greek yogurt, cottage cheese, low-fat milk, and unsalted nuts or nut butter in small portions.

**3. Meal frequency and eating pattern**

AASLD recommends minimizing fasting, with no more than **3–4 hours between nutritional intake during waking hours**, aiming for **3–5 meals per day**, plus an early breakfast and/or late-evening snack.

Practical pattern:

- **Breakfast within 1–2 hours of waking**
- **Lunch**
- **Dinner**
- **1–2 planned snacks**
- **Mandatory late-evening snack**, especially if dinner is early

Late snack examples, ideally with protein plus complex carbohydrate:

- Greek yogurt + berries
- Milk or soy milk + whole-grain toast
- Cottage cheese + fruit
- Oatmeal made with milk
- Tofu pudding or soy yogurt
- Whole-grain crackers + hummus

Avoid skipping breakfast, fasting for weight loss, or very-low-carbohydrate crash diets.

**4. Specific food recommendations and restrictions**

**Encourage**

**Protein-rich foods**

- Fish 2–3 times/week
- Skinless poultry
- Eggs
- Tofu, tempeh, edamame
- Beans, lentils, chickpeas
- Greek yogurt, milk, cottage cheese
- Protein supplement only if food intake is insufficient

**Carbohydrates**

- Oats, brown rice, barley, quinoa
- Whole-grain bread or noodles
- Beans and lentils
- Fruit in whole form rather than juice

**Fats**

- Olive oil or canola oil in measured amounts
- Avocado in modest portions
- Nuts/seeds, preferably unsalted, small handful portions

**Vegetables**

- Non-starchy vegetables at most meals
- High-fiber choices to support weight, glucose, and lipid control

**Limit or avoid**

- Alcohol completely
- Sugary drinks, fruit juice, sweet tea, desserts, refined snacks
- White bread, white rice, pastries, and large portions of noodles
- Processed meats: sausage, bacon, deli meats
- High-salt foods: instant noodles, pickles, salted fish, chips, canned soups, heavily processed foods
- Raw or undercooked shellfish because cirrhosis increases risk from foodborne infections
- Herbal or “liver detox” supplements unless specifically reviewed by her hepatology team

**Varices-specific notes**

Non-bleeding esophageal varices do **not** require a soft or liquid diet. She can eat normally unless she has dysphagia or post-banding ulcer symptoms. Avoid very sharp, hard foods only if they cause discomfort, but diet alone does not prevent variceal bleeding. Continue standard variceal management through hepatology.

**5. Additional nutritional considerations**

Because she has obesity with cirrhosis, the goal is **fat loss without muscle loss**. Do not prescribe severe calorie restriction. A reasonable starting approach is a modest energy deficit with high protein and resistance exercise. AASLD notes that caloric intake for stable non-hospitalized patients with BMI 30–40 should be individualized, generally around **25–35 kcal/kg/day**, while considering fluid overload.

Recommended add-ons:

- **Screen for sarcopenia**: grip strength, chair stands, muscle mass if available.
- **Exercise**: combine walking/aerobic activity with resistance training 2–3 times/week if medically cleared. AASLD recommends aerobic plus resistance exercise tailored to baseline function.
- **Check micronutrients annually**: vitamin D, zinc, B12, folate, iron studies as clinically appropriate. AASLD recommends annual micronutrient assessment and vitamin D repletion if deficient.
- **Bone health**: consider calcium/vitamin D adequacy and DEXA based on age, menopause status, steroid exposure, cholestasis, or other risk factors.
- **Metabolic risk control**: coordinate diabetes, lipids, and blood pressure management; MASLD outcomes depend heavily on cardiometabolic control.
- **Dietitian referral**: ideally to a dietitian familiar with cirrhosis and MASLD.

**Example one-day plan**

**Breakfast:** oatmeal cooked with milk or soy milk + boiled egg + berries
**Snack:** Greek yogurt or unsweetened soy yogurt
**Lunch:** grilled fish/chicken/tofu + brown rice or quinoa + vegetables + olive-oil-based dressing
**Snack:** fruit + unsalted nuts, or hummus with whole-grain crackers
**Dinner:** lentil/bean soup or chicken/tofu stir-fry + vegetables + small portion whole grain
**Late-evening snack:** milk/soy milk with whole-grain toast, or cottage cheese/Greek yogurt with fruit

**Daily target summary:**
Protein **65–80 g/day**, meals/snacks every **3–4 hours**, **late-evening snack**, Mediterranean-style diet, gradual weight loss, no alcohol, avoid crash dieting and unnecessary protein restriction.

# C1-09

**Patient summary**

61-year-old man, 167 cm, 58 kg; BMI ≈ **20.8 kg/m²**. He has **alcohol-related compensated cirrhosis**, so the main nutrition goals are to **prevent sarcopenia/malnutrition, avoid long fasting, maintain adequate protein and calories, and completely avoid alcohol**.

**1. Overall dietary principles**

Aim for a **high-protein, adequate-calorie, Mediterranean-style diet**, not a “liver-cleansing” or low-protein diet. In cirrhosis, protein restriction is generally discouraged because it worsens muscle loss; EASL and AASLD guidance recommend **1.2–1.5 g/kg/day protein** and frequent meals with a late-evening snack.

Practical daily targets for him:

| **Target** | **Recommendation** |
| --- | --- |
| Calories | about **1,800–2,100 kcal/day** |
| Protein | **70–87 g/day** |
| Fluids | normal intake unless hyponatremia/ascites management requires restriction |
| Alcohol | **0 alcohol** |

Energy intake in cirrhosis is commonly targeted around **30–35 kcal/kg/day**, with higher needs if malnourished or sarcopenic. For 58 kg, that is about **1,740–2,030 kcal/day**.

**2. Recommended daily protein intake**

Use **1.2–1.5 g/kg/day**.

For this patient:

**58 kg × 1.2–1.5 = 70–87 g protein/day**

A practical prescription: **aim for ~80 g/day**, divided across meals and snacks.

Example distribution:

| **Eating time** | **Protein goal** |
| --- | --- |
| Breakfast | 20 g |
| Lunch | 20–25 g |
| Dinner | 25 g |
| Snacks/late snack | 10–15 g |

Good protein choices include **fish, chicken, eggs, dairy/yogurt, tofu, soy milk, beans/lentils, nuts, and lean meats**. Plant and dairy proteins are often well tolerated and helpful if there is concern for encephalopathy, but **do not restrict total protein** unless there is a very specific short-term medical reason.

**3. Meal frequency and eating pattern**

Recommend **3 meals + 2–3 snacks daily**, with special emphasis on a **late-evening snack**. Cirrhosis is associated with accelerated fasting metabolism, so long overnight fasting can promote muscle breakdown; EASL recommends breakfast with protein and a late-evening snack to shorten fasting time.

Daily pattern:

- **Breakfast within 1 hour of waking**
- **Lunch and dinner with protein each time**
- **Mid-afternoon snack**
- **Late-evening snack before bed**, ideally containing carbohydrate plus protein

Late snack examples:

- Greek yogurt + fruit
- Milk or soy milk + whole-grain toast
- Peanut butter on toast
- Oatmeal made with milk
- Cheese + crackers
- Tofu pudding or soy milk + banana

**4. Specific food recommendations and restrictions**

**Encourage**

**Protein-rich foods daily:**
Fish, poultry, eggs, tofu, soy products, beans/lentils, milk, yogurt, kefir, cottage cheese, nuts, nut butters.

**Complex carbohydrates:**
Rice, oats, whole-grain bread, noodles, potatoes, corn, fruit. Carbohydrates are useful at snacks because they reduce fasting-related muscle breakdown.

**Healthy fats:**
Olive oil, avocado, nuts, seeds, fatty fish.

**Vegetables and fruit:**
Aim for a variety daily, unless potassium restriction is needed for renal disease or hyperkalemia.

**Coffee:**
Coffee is generally acceptable if tolerated and not contraindicated by reflux, insomnia, arrhythmia, or other individual issues.

**Avoid or limit**

**Alcohol: absolute avoidance.**
For alcohol-related cirrhosis, continued drinking increases the risk of decompensation, hepatocellular carcinoma, and mortality. Nutrition advice will not compensate for ongoing alcohol use.

**Unnecessary protein restriction:**
Avoid telling the patient to “eat less protein for the liver.” Normal-to-high protein intake does **not** generally precipitate hepatic encephalopathy and is recommended to prevent muscle loss.

**High-salt processed foods:**
Even though he is compensated and no ascites is described, avoid excess sodium from instant noodles, cured meats, chips, pickles, canned soups, fast food, and salty sauces. If ascites or edema develops, use a stricter sodium goal, commonly **≤2 g sodium/day**.

**Raw or high-risk foods:**
Avoid raw oysters/shellfish and unsafe raw seafood because cirrhosis increases susceptibility to severe infection. Use standard food safety precautions.

**Herbal “liver detox” supplements:**
Avoid non-prescribed herbal products and bodybuilding supplements because of hepatotoxicity risk and unpredictable ingredients.

**5. Additional nutritional considerations**

Because this is **alcohol-related cirrhosis**, screen and replete common deficiencies: **thiamine, folate, vitamin B12, vitamin D, calcium, magnesium, zinc**, and fat-soluble vitamins if clinically indicated. A daily multivitamin without excess vitamin A or iron is reasonable if intake is poor, but supplements should be individualized.

Assess for **sarcopenia** even if BMI looks normal. Ask about weight loss, grip strength, falls, walking speed, and muscle wasting. Nutrition works best with **resistance exercise**, for example light weights, bands, sit-to-stand exercises, and walking as tolerated.

A simple daily meal example:

- **Breakfast:** eggs or tofu + oatmeal with milk/soy milk + fruit
- **Snack:** yogurt or soy milk
- **Lunch:** rice/noodles/potato + fish/chicken/beans + vegetables + olive oil
- **Snack:** nuts + fruit or peanut butter toast
- **Dinner:** tofu/fish/chicken + grains + vegetables
- **Bedtime snack:** milk/soy milk or Greek yogurt + crackers/toast/oats

**Bottom line prescription:** about **2,000 kcal/day**, **80 g protein/day**, **3 meals + 2–3 snacks**, **bedtime snack every night**, **no alcohol**, avoid unnecessary sodium excess, and monitor for sarcopenia and micronutrient deficiencies.

# C1-10

For this **66-year-old woman with HBV-related compensated cirrhosis**, height **155 cm**, weight **50 kg**, BMI ≈ **20.8 kg/m²**, the goal is to **prevent sarcopenia, maintain weight and muscle, avoid unnecessary restriction, and reduce risk of decompensation**.

**1. Overall dietary principles**

She should follow a **balanced, high-protein, adequate-calorie diet**, not a “light” or protein-restricted liver diet.

Recommended daily targets:

| **Item** | **Target** |
| --- | --- |
| Energy | **30–35 kcal/kg/day = about 1500–1750 kcal/day** |
| Protein | **1.2–1.5 g/kg/day = about 60–75 g/day** |
| Pattern | **Small frequent meals + bedtime snack** |

Modern liver nutrition guidance recommends **30–35 kcal/kg/day** and **1.2–1.5 g/kg/day protein** for cirrhosis, with frequent meals and a late-evening snack to reduce fasting-related muscle breakdown.

**2. Recommended daily protein intake**

For her weight of **50 kg**:

**Protein goal: 60–75 g/day**

A practical distribution:

| **Meal/snack** | **Protein target** |
| --- | --- |
| Breakfast | 15–20 g |
| Lunch | 20–25 g |
| Dinner | 20–25 g |
| Snacks/bedtime snack | 10–15 g |

Good protein sources include:

- Eggs, fish, chicken, lean pork/beef in moderate portions
- Milk, yogurt, cheese, soy milk
- Tofu, soy products, beans, lentils
- Nuts or nut butter in small amounts
- Protein oral supplement if intake is poor

**Do not restrict protein** simply because she has cirrhosis. Protein restriction can worsen muscle loss and frailty. If hepatic encephalopathy ever occurs, protein should usually still be maintained, with more emphasis on **vegetable/dairy protein** rather than reducing total protein. AASLD guidance similarly emphasizes adequate protein and minimizing fasting in cirrhosis.

**3. Meal frequency and eating pattern**

Recommend:

- **3 main meals + 2–3 snacks daily**
- Avoid fasting longer than **4–6 hours during the day**
- Add a **late-evening snack before bed**
- Avoid skipping breakfast

A bedtime snack is especially useful in cirrhosis because patients enter a catabolic state faster overnight.

Good bedtime snack examples:

- Milk or yogurt + whole-grain bread
- Soy milk + steamed bun or oatmeal
- Tofu pudding or yogurt + fruit
- Peanut butter toast
- Small bowl of cereal/oats with milk
- Nutritional supplement drink if appetite is poor

**4. Specific food recommendations and restrictions**

**Recommended foods**

- Staple carbohydrates: rice, noodles, oats, potatoes, whole grains as tolerated
- Protein: fish, eggs, poultry, tofu, soy products, milk/yogurt
- Vegetables: daily, preferably cooked if digestion is poor
- Fruit: 1–2 servings/day
- Healthy fats: olive/canola oil, nuts, avocado in moderate amounts
- Adequate fluids unless later restricted for hyponatremia or ascites

**Limit or avoid**

- **Alcohol: completely avoid**
- Raw or undercooked seafood, especially raw oysters/shellfish, due to infection risk in liver disease
- Very salty foods if edema/ascites develops: pickles, salted fish, cured meats, instant noodles, preserved vegetables, processed snacks
- Sugary drinks and desserts in excess, especially if diabetes or insulin resistance is present
- Unverified herbal remedies or “liver detox” supplements, which may cause hepatotoxicity

For this patient with **compensated cirrhosis and no ascites mentioned**, I would not impose a very strict sodium restriction. A reasonable approach is **avoid high-salt processed foods**, but do not make the diet so bland that intake falls. ESPEN notes that low-sodium diets should be balanced against the risk of reduced food intake in cirrhosis.

**5. Additional nutritional considerations**

She is older, relatively small, and has cirrhosis, so **muscle preservation is a major priority** even if her weight appears normal.

Important additions:

- Assess for **sarcopenia/frailty**: handgrip strength, walking speed, muscle mass if available.
- Encourage **resistance exercise** if medically safe: light weights, sit-to-stand exercises, resistance bands, walking.
- Check and replete common deficiencies: **vitamin D, calcium, zinc, folate, B vitamins**, and iron only if deficient.
- Ensure adequate **calcium and vitamin D** because cirrhosis and older age increase osteoporosis risk.
- Monitor weight, appetite, edema, ascites, bowel habits, glucose, albumin/prealbumin trends if clinically useful, and dietary intake.
- Continue HBV antiviral management and routine cirrhosis surveillance per hepatology care.

**Practical one-day example**

Breakfast: egg + oatmeal with milk or soy milk + fruit
Snack: yogurt or soy milk
Lunch: rice/noodles + fish/chicken/tofu + vegetables
Snack: nuts or milk + bread
Dinner: rice + tofu/lean meat/fish + vegetables
Bedtime snack: milk/yogurt/soy milk + toast, oats, or small bun

**Bottom line:** For her, aim for **1500–1750 kcal/day**, **60–75 g protein/day**, **frequent meals**, and a **bedtime snack**, with no unnecessary protein restriction.

# C1-11

For this **70-year-old man with MASLD-related compensated cirrhosis**, height **171 cm**, weight **76 kg**, BMI is about **26.0 kg/m²**. The key goals are to **preserve muscle, avoid fasting-related catabolism, support metabolic health, and prevent progression of MASLD**, while avoiding unnecessary dietary restriction.

**1. Overall dietary principles**

Use a **Mediterranean-style, high-protein, metabolically healthy diet**:

- Emphasize **adequate protein at every meal**, not protein restriction.
- Avoid long fasting periods, especially overnight.
- Focus on **whole foods**: vegetables, legumes, whole grains, fruit, fish, eggs, dairy, tofu/soy, poultry, nuts, and olive oil.
- Limit **sugary drinks, desserts, refined starches, ultra-processed foods, and saturated fat**, which are particularly relevant in MASLD.
- Avoid alcohol completely.
- Maintain stable weight or pursue only **gradual weight loss** if clinically desired; avoid crash dieting because it can worsen sarcopenia.

Modern cirrhosis guidance recommends **1.2–1.5 g/kg/day protein** for clinically stable adults with cirrhosis and advises against routine protein restriction, even in hepatic encephalopathy.

**2. Recommended daily protein intake**

For this patient:

- Using actual body weight: **76 kg × 1.2–1.5 g/kg/day = ~91–114 g protein/day**
- Practical target: **about 95–110 g protein/day**

A reasonable distribution:

| **Eating time** | **Protein target** |
| --- | --- |
| Breakfast | 20–25 g |
| Lunch | 25–30 g |
| Dinner | 25–30 g |
| Snacks, including bedtime snack | 20–25 g total |

Protein sources should include a mix of:

- **Fish**, especially oily fish such as salmon, sardines, trout
- **Eggs**
- **Low-fat Greek yogurt, milk, kefir, cottage cheese**
- **Tofu, soy milk, edamame**
- **Beans, lentils, chickpeas**
- **Poultry**
- Lean red meat only occasionally, if used

Vegetable and dairy proteins are often well tolerated and are encouraged in cirrhosis nutrition guidance.

**3. Meal frequency and eating pattern**

Recommend **4–6 eating occasions daily**, including a **late-evening snack**.

Suggested pattern:

- Breakfast within 1–2 hours of waking
- Lunch
- Afternoon snack
- Dinner
- **Bedtime snack**, ideally within 1–2 hours before sleep

The bedtime snack is important because patients with cirrhosis enter a fasting/catabolic state earlier than healthy individuals. EASL guidance recommends avoiding long fasting periods and using frequent meals plus a late-evening snack; World Gastroenterology Organization commentary also highlights a bedtime snack with around **50 g carbohydrate** to support protein balance.

Good bedtime snack examples:

- Greek yogurt + berries + small handful of oats
- Milk or soy milk + whole-grain toast with peanut butter
- Cottage cheese + fruit
- Oatmeal made with milk
- Hummus with whole-grain crackers
- Banana + unsweetened yogurt
- Protein-fortified oral nutrition supplement if intake is poor

**4. Specific food recommendations and restrictions**

**Encourage**

**Protein-rich foods daily**

- Greek yogurt, eggs, tofu, fish, poultry, beans, lentils, cottage cheese.

**High-fiber carbohydrates**

- Oats, barley, brown rice, quinoa, whole-grain bread, beans, lentils, vegetables, fruit.

**Healthy fats**

- Olive oil, avocado, nuts, seeds, fatty fish.

**MASLD-friendly choices**

- Mediterranean-style meals.
- Replace refined carbohydrates with whole grains and legumes.
- Avoid sweetened beverages and fruit juice.
- Minimize pastries, candy, white bread, processed snacks.

**Limit or avoid**

**Alcohol**

- Complete abstinence.

**Sugary drinks and fructose-heavy foods**

- Soda, sweet tea, energy drinks, large amounts of juice, desserts.

**Ultra-processed and high-saturated-fat foods**

- Processed meats, fast food, fried foods, packaged sweets.

**Excess sodium**

- Since he has **compensated cirrhosis without stated ascites or edema**, severe sodium restriction is not automatically required.
- A practical goal is **avoid high-salt processed foods** and keep sodium roughly around **≤2,000 mg/day if ascites, edema, or hypertension is present**.
- Do not let sodium restriction reduce total calorie or protein intake.

**Raw or undercooked seafood**

- Avoid raw oysters, raw shellfish, and undercooked seafood because cirrhosis increases infection risk.

**5. Additional nutritional considerations**

**Energy intake**

For compensated cirrhosis, many guidelines use roughly **30–35 kcal/kg/day**, but in MASLD with BMI 26, energy should be individualized. A practical starting range is:

- **~1,900–2,300 kcal/day**, adjusted by weight trend, appetite, muscle mass, and metabolic goals.

If weight loss is desired, aim for **slow loss**, for example about **0.25–0.5 kg/week**, while maintaining the protein target and resistance exercise.

**Screen for sarcopenia and frailty**

Even overweight MASLD patients can have low muscle mass. Assess:

- Handgrip strength
- Chair stands or gait speed
- Muscle mass if available
- Unintentional weight loss
- Poor appetite

If sarcopenia is present, use the upper protein range: **~110–115 g/day**, and consider oral nutrition supplementation.

**Exercise pairing**

Nutrition works best with activity:

- **Resistance training 2–3 times/week**, adapted to safety and fitness.
- Daily walking or aerobic activity as tolerated.
- Avoid prolonged inactivity.

**Micronutrients**

Check and replete if low:

- Vitamin D
- Calcium intake
- Zinc, especially if poor intake, taste changes, or encephalopathy history
- B vitamins if dietary intake is poor
- Fat-soluble vitamins if cholestasis or malabsorption is present

Avoid high-dose supplements, herbal liver “detox” products, and unnecessary iron unless deficiency is confirmed.

**Practical one-day example**

**Breakfast:**
Oatmeal made with milk or soy milk + Greek yogurt + berries.

**Snack:**
Boiled egg or cottage cheese + fruit.

**Lunch:**
Grilled fish or tofu bowl with brown rice/quinoa, vegetables, olive oil dressing.

**Snack:**
Hummus with whole-grain crackers or nuts plus fruit.

**Dinner:**
Chicken, fish, lentils, or tofu + vegetables + whole grain or sweet potato.

**Bedtime snack:**
Greek yogurt with oats, or milk/soy milk with whole-grain toast and peanut butter.

**Bottom line:** target **~95–110 g protein/day**, split across **4–6 meals/snacks**, include a **protein-containing bedtime snack**, avoid alcohol and sugary/ultra-processed foods, and prioritize muscle preservation alongside MASLD metabolic control.

# C1-12

**Patient-specific targets**

73-year-old woman, 159 cm, 66 kg; BMI ≈ **26.1 kg/m²**. She has **compensated alcohol-related cirrhosis**, so the key goals are to **prevent sarcopenia/frailty, avoid long fasting, maintain adequate energy and protein, and support alcohol abstinence**.

**1. Overall dietary principles**

Recommend a **balanced, high-protein, nutrient-dense diet**, not a low-protein diet. In cirrhosis, inadequate protein increases muscle loss and frailty; current guidance recommends **1.2–1.5 g/kg/day protein** and does **not** recommend routine protein restriction, even in hepatic encephalopathy.

Daily energy target: approximately **30–35 kcal/kg/day**, adjusted to appetite, weight trend, ascites status, and activity level. For her actual weight, that is roughly **2,000–2,300 kcal/day**. Avoid intentional weight loss unless clearly indicated and supervised, because older adults with cirrhosis are at high risk for muscle loss.

Absolutely recommend **complete alcohol abstinence**.

**2. Recommended daily protein intake**

For 66 kg body weight:

**Protein goal: 80–100 g/day**
Calculation: 66 kg × 1.2–1.5 g/kg/day = **79–99 g/day**

A practical distribution:

| **Meal/snack** | **Protein target** |
| --- | --- |
| Breakfast | 20–25 g |
| Lunch | 20–25 g |
| Dinner | 25–30 g |
| Snacks, including bedtime snack | 15–25 g total |

Good protein sources include **fish, eggs, poultry, tofu, soy milk, beans/lentils, Greek yogurt, milk, cottage cheese, and other dairy**. Vegetable and dairy proteins are often well tolerated in cirrhosis and are useful if there is concern for encephalopathy.

**3. Meal frequency and eating pattern**

Recommend **small, frequent meals**: **3 meals plus 2–3 snacks daily**.

Avoid fasting longer than about **3–4 hours while awake**, and include a **late-evening snack** to reduce overnight muscle breakdown. AASLD/EASL-aligned guidance emphasizes minimizing fasting, using early breakfast and/or a late-evening snack; late-night snacks have been studied with a range of calorie/protein compositions.

Practical bedtime snack examples:

- Greek yogurt + fruit
- Milk or soy milk + whole-grain toast
- Peanut butter on toast or crackers
- Oatmeal made with milk
- Tofu/soy pudding
- Protein smoothie with milk/soy milk and fruit
- Rice ball or whole-grain cereal plus dairy/soy protein

Aim for **~200–300 kcal** and ideally **10–20 g protein** at bedtime.

**4. Specific food recommendations and restrictions**

**Encourage:**

- **High-quality protein at every meal**
- **Whole grains/starches**: rice, oats, potatoes, whole-grain bread, pasta
- **Fruits and vegetables** daily
- **Legumes and soy foods**: tofu, edamame, lentils, beans
- **Dairy or fortified alternatives**: milk, yogurt, cheese, calcium/vitamin D–fortified soy milk
- **Healthy fats**: olive oil, avocado, nuts/nut butters, seeds
- **Adequate fluid intake**, unless hyponatremia or ascites management requires restriction

**Limit or avoid:**

- **Alcohol: avoid completely**
- **Raw or undercooked shellfish**, especially oysters, due to infection risk in chronic liver disease
- Excess highly processed foods, sugary drinks, and low-nutrient snacks
- Herbal supplements or “liver detox” products unless reviewed by her clinician
- Very high sodium intake; if she develops ascites/edema or has hypertension, use a **2 g sodium/day** target. In compensated cirrhosis without ascites, avoid excessive salt but do not over-restrict if it compromises intake.

Do **not** prescribe protein restriction for compensated cirrhosis. Protein restriction is discouraged because it worsens malnutrition and does not reliably prevent encephalopathy.

**5. Additional nutritional considerations**

Because this is **alcohol-related cirrhosis** and she is older, assess and supplement as needed:

- **Thiamine**, especially if recent alcohol use or poor intake
- **Folate and vitamin B12**
- **Vitamin D and calcium**; consider bone density assessment because cirrhosis and older age increase osteoporosis risk
- **Zinc** if poor intake, dysgeusia, diarrhea, or encephalopathy concerns
- Screen for **sarcopenia/frailty**: grip strength, chair stands, gait speed, muscle mass if available
- Encourage **resistance exercise plus walking**, as tolerated, because protein works best with muscle stimulus
- Monitor weight trend, appetite, edema/ascites, bowel pattern, labs, and signs of hepatic encephalopathy

**Example one-day pattern**

Breakfast: oatmeal made with milk/soy milk + egg or Greek yogurt
Snack: fruit + nuts or yogurt
Lunch: rice/noodles/potato + fish/chicken/tofu + vegetables
Snack: milk/soy milk or cheese/tofu snack
Dinner: lean protein + starch + vegetables + olive oil
Bedtime: Greek yogurt, milk/soy milk with toast, or peanut butter crackers

**Bottom line:** For this patient, prescribe **~80–100 g protein/day**, **~2,000–2,300 kcal/day**, **3 meals plus 2–3 snacks**, and a **protein-containing late-evening snack**, with strict alcohol abstinence and attention to micronutrients, bone health, and sarcopenia prevention.

# C2-01

**Patient:** 33-year-old male, 173 cm, 69 kg, HBV-related cirrhosis with ascites, clinically stable, eating orally.
**Nutrition goal:** prevent sarcopenia and malnutrition while controlling ascites and maintaining adequate energy/protein intake.

**1. Overall dietary principles**

Use a **high-energy, high-protein, low-sodium diet**.

For a stable outpatient with cirrhosis, target about **30–35 kcal/kg/day**, ideally based on estimated dry weight if ascites is significant. For this patient, using 69 kg as a working weight:

**Energy target:** approximately **2,100–2,400 kcal/day**.

Patients with cirrhosis should **not routinely restrict protein**, even if there is concern for hepatic encephalopathy; modern guidelines recommend adequate protein to prevent muscle loss. EASL and ESPEN recommend **1.2–1.5 g/kg/day protein** in cirrhosis.

**2. Recommended daily protein intake**

**Protein target:** **1.2–1.5 g/kg/day**
For 69 kg:

**≈ 83–104 g protein/day**

A practical prescription: **90–100 g/day**, divided across meals and snacks.

Examples of approximate protein portions:

| **Food** | **Approximate protein** |
| --- | --- |
| 1 egg | 6 g |
| 250 mL milk or fortified soy milk | 8 g |
| 100 g fish/chicken/lean meat | 20–25 g |
| 100 g tofu | 8–12 g |
| 1 cup cooked beans/lentils | 15–18 g |
| 150–200 g Greek yogurt | 15–20 g |

Prefer a mix of **plant protein, dairy protein, fish, poultry, eggs, tofu, beans, and lean meat**. Plant and dairy proteins may be better tolerated in patients prone to encephalopathy.

**3. Meal frequency and eating pattern**

Cirrhosis patients enter a “fasting” metabolic state quickly, so avoid long gaps without food.

Recommended pattern:

**4–6 eating occasions daily**, including:

- Breakfast
- Lunch
- Dinner
- 1–2 daytime snacks
- **Late-evening snack before bed**

A late-evening snack is specifically recommended in cirrhosis to reduce overnight catabolism and support nitrogen balance. ESPEN notes supportive evidence for late-evening snacks in cirrhosis.

Good late-evening snack options:

- Milk or yogurt + unsalted crackers or bread
- Oatmeal with milk
- Egg sandwich with low-sodium bread
- Tofu pudding or soy milk + steamed bun
- Greek yogurt + fruit
- Rice porridge with egg or tofu

Aim for a bedtime snack containing **carbohydrate plus protein**, not just fruit or tea.

**4. Specific food recommendations and restrictions**

**Sodium restriction for ascites**

Because he has ascites, recommend:

**Sodium ≤ 2,000 mg/day**, equivalent to about **5 g salt/day**.

This is a key ascites-control measure. ESPEN also cautions that sodium restriction should not make the diet so unpalatable that intake falls, because poor intake worsens malnutrition risk.

Practical advice:

- Do not add salt at the table.
- Avoid salted pickles, preserved vegetables, cured meats, salted fish, instant noodles, chips, processed meats, canned soups, and high-sodium sauces.
- Limit soy sauce, oyster sauce, chili sauce, fermented bean paste, bouillon cubes, and hotpot soup bases.
- Use garlic, ginger, vinegar, lemon, herbs, pepper, onion, scallion, cumin, and other salt-free seasonings.
- Choose fresh foods over processed foods.
- Read labels: choose foods with **low sodium**, ideally <120 mg sodium/100 g when possible.

**Fluid restriction**

Do **not** routinely restrict fluids unless he has **hyponatremia**, especially serum sodium <125–130 mmol/L, or his hepatologist specifically recommends it. In ascites, sodium restriction is usually more important than fluid restriction.

**Alcohol**

**Complete alcohol avoidance** is recommended, even though the etiology is HBV, because alcohol accelerates liver injury.

**Protein restriction**

Avoid unnecessary protein restriction. Protein restriction increases risk of sarcopenia and poor outcomes. If encephalopathy develops, adjust protein source and meal pattern rather than simply cutting protein.

**Food safety**

Cirrhosis increases infection risk. Recommend avoiding:

- Raw or undercooked shellfish, especially oysters
- Raw fish or unsafe sashimi sources
- Undercooked eggs or meats
- Unpasteurized dairy
- Spoiled leftovers

Use careful hand hygiene and proper food storage.

**5. Additional nutritional considerations**

**Monitor for malnutrition and sarcopenia**

Even with normal BMI, ascites can mask muscle loss. Track:

- Dry weight trend
- Appetite and daily intake
- Handgrip strength if available
- Muscle wasting at temples, shoulders, thighs
- Frailty, fatigue, reduced walking speed

**Micronutrients**

Check and replete if deficient:

- Vitamin D and calcium
- Zinc, especially if poor intake, dysgeusia, diarrhea, or encephalopathy
- Thiamine if poor intake or any alcohol history
- Folate, B12, iron studies if anemia is present

Avoid high-dose vitamin A or niacin unless prescribed because of hepatotoxicity risk.

**Branched-chain amino acids**

If he cannot meet protein goals orally, has muscle loss, or has recurrent hepatic encephalopathy with poor protein tolerance, consider **oral nutrition supplements** or **BCAA-enriched supplements**, guided by hepatology/nutrition team.

**Exercise**

Nutrition works best with activity. If medically stable, recommend **regular resistance exercise plus walking**, adjusted for fatigue and ascites symptoms, to preserve muscle mass.

**Practical daily prescription for this patient**

**Calories:** about **2,100–2,400 kcal/day**
**Protein:** about **90–100 g/day**
**Sodium:** **≤2,000 mg/day**
**Meals:** **3 meals + 2–3 snacks**, including a **late-evening snack**
**Diet pattern:** low-sodium, high-protein, adequate calories, no alcohol, avoid long fasting periods.

A simple daily structure:

- **Breakfast:** egg + oatmeal or rice porridge + milk/soy milk
- **Snack:** yogurt or milk + fruit
- **Lunch:** rice/noodles + fish/chicken/tofu + vegetables, cooked with little/no salt
- **Snack:** unsalted nuts, yogurt, or tofu/soy milk
- **Dinner:** lean protein + staple carbohydrate + vegetables
- **Bedtime snack:** milk/yogurt/soy milk + bread/crackers/oatmeal or egg/tofu-based snack

# C2-02

Below is a practical outpatient plan for a **37-year-old woman with MASLD cirrhosis, ascites, and reduced appetite**. Because ascites can falsely raise body weight, use **estimated dry weight** when available; until then, 56 kg gives a reasonable starting target.

**1. Overall dietary principles**

Aim for **adequate energy, high protein, low sodium, and minimal fasting**. In cirrhosis, malnutrition and sarcopenia are common, especially with ascites and poor appetite; restrictive diets can worsen intake. AASLD notes that non-obese cirrhosis patients generally target about **35 kcal/kg/day**, with sodium restriction balanced against malnutrition risk.

For her:
**Energy goal:** about **1,700–2,000 kcal/day** using 30–35 kcal/kg/day, adjusted to dry weight, intake, ascites control, and metabolic goals.

For MASLD, do **not** pursue aggressive weight loss during decompensation/ascites. Focus on **muscle preservation**, stable intake, and metabolic quality: whole grains, legumes, vegetables, lean protein, unsaturated fats, and avoidance of sugar-sweetened beverages.

**2. Recommended daily protein intake**

Recommended protein intake in cirrhosis is **1.2–1.5 g/kg/day**; AASLD also emphasizes that protein should **not be restricted**, even in hepatic encephalopathy.

For 56 kg:
**Protein target: 67–84 g/day**
Given ascites, poor appetite, and sarcopenia risk, I would aim toward the upper range if tolerated: **75–85 g/day**, divided across the day.

Practical distribution:

- Breakfast: 15–20 g
- Lunch: 20–25 g
- Dinner: 20–25 g
- Snacks/bedtime snack: 15–20 g total

Good protein choices: eggs, fish, poultry, tofu/soy foods, Greek yogurt, milk, beans/lentils, unsalted nuts/nut butter, and protein-fortified oral nutrition supplements if meals are small.

**3. Meal frequency and eating pattern**

Use **small frequent meals** because ascites causes early satiety.

Recommended pattern:
**4–6 eating occasions/day**, avoiding fasting longer than about **3–4 hours while awake**, plus a **late-evening snack**. EASL recommends breakfast with protein and a late-evening snack to shorten overnight fasting in cirrhosis.

Suggested daily schedule:

- Breakfast within 1 hour of waking
- Mid-morning snack
- Lunch
- Afternoon snack
- Dinner
- **Bedtime snack**, ideally containing carbohydrate plus protein

Bedtime snack examples:

- Greek yogurt + fruit
- Milk or soy milk + oatmeal
- Peanut butter on low-sodium toast
- Unsalted crackers + hummus
- Oral nutrition supplement, preferably low-sodium/high-protein if intake is poor

**4. Specific food recommendations and restrictions**

**Sodium restriction for ascites**

Target **<2,000 mg sodium/day**, roughly **5 g salt/day**. AASLD outpatient guidance says sodium restriction to <2 g/day should be considered in ascites, while fluid restriction is generally unnecessary unless hyponatremia is present.

Practical sodium advice:

- Avoid: pickles, salted snacks, instant noodles, canned soups, processed meats, bacon/sausage, salted fish, soy sauce, oyster sauce, bouillon cubes, fast food, frozen meals.
- Choose: fresh foods, “no salt added” canned beans/vegetables, low-sodium seasonings, vinegar, lemon, garlic, onion, pepper, herbs, chili, ginger.
- Do not use “salt substitutes” containing potassium unless cleared by her clinician, especially if on spironolactone or with kidney dysfunction.

**Protein and food choices**

Encourage high-protein foods at every meal:

- Fish, chicken, eggs
- Tofu, tempeh, soy milk
- Beans/lentils, if tolerated
- Greek yogurt, milk, cottage cheese if sodium content is acceptable
- Unsalted nuts and nut butters

For MASLD/metabolic health:

- Prefer Mediterranean-style meals: vegetables, legumes, whole grains, fish, olive/canola oil, nuts.
- Limit refined carbohydrates: white bread, sweets, desserts, sweetened drinks, fruit juice.
- Avoid high-fructose/sugar-sweetened beverages.
- Avoid alcohol completely.

**Appetite and ascites strategies**

Because she has decreased appetite:

- Use smaller portions but enrich meals: add olive oil, avocado, nut butter, powdered milk, Greek yogurt, tofu, egg, or protein powder if appropriate.
- Drink fluids between meals rather than before meals if early satiety is prominent.
- Consider low-sodium oral nutrition supplements when she cannot meet targets with food.
- If ascites is tense and limiting intake, nutrition will not fully succeed without optimizing ascites management.

**5. Additional nutritional considerations**

Monitor for **sarcopenia and micronutrient deficiencies**. AASLD notes annual screening for deficiencies such as **vitamin D, vitamin E, folate, thiamine, zinc, and selenium**, and consideration of bone density screening in cirrhosis.

Additional recommendations:

- Check: vitamin D, zinc, folate/B12, iron studies if anemic, magnesium/potassium/sodium/creatinine if on diuretics.
- Calcium/vitamin D supplementation if deficient or osteopenic/osteoporotic.
- Zinc repletion only if deficient or clinically indicated.
- Encourage resistance exercise as tolerated: walking plus light resistance training 2–3 times/week, because nutrition works best with muscle stimulus.
- Avoid raw oysters or raw shellfish due to infection risk in cirrhosis.
- Avoid unnecessary fasting for procedures; resume nutrition promptly afterward.

**Bottom line prescription:** approximately **1,700–2,000 kcal/day**, **75–85 g protein/day**, **<2 g sodium/day**, **4–6 small meals/snacks daily**, and a **protein-containing bedtime snack**, with close monitoring of dry weight, ascites, electrolytes, renal function, appetite, and muscle mass.

# C2-03

**Patient summary**

41-year-old man, 168 cm, 84 kg, alcohol-related cirrhosis with **moderate ascites**. His measured BMI is about **29.8 kg/m²**, but ascites makes body weight overestimate true nutritional status. The main priorities are **preventing sarcopenia**, controlling ascites with sodium restriction, and maintaining complete alcohol abstinence.

**1. Overall dietary principles**

Aim for a **high-protein, adequate-calorie, low-sodium cirrhosis diet**. Do **not** prescribe a low-protein diet, even in cirrhosis, because protein restriction worsens muscle loss and outcomes. EASL guidance supports protein intake of **1.2–1.5 g/kg/day** and frequent meals with a late-evening snack in cirrhosis.

For ascites, use **moderate sodium restriction**, not starvation-level salt avoidance. AASLD outpatient guidance recommends considering sodium restriction to **<2 g sodium/day**, while noting that nutrition targets should not be sacrificed. Routine fluid restriction is **not needed unless hyponatremia is present**.

**2. Recommended daily protein intake**

Because his actual weight includes ascitic fluid, estimate needs using **dry weight or ideal/adjusted body weight**.

For practical counseling:

**Protein target: about 90–110 g/day**

A reasonable calculation is:

- Estimated dry/adjusted weight: roughly **70–75 kg**
- Protein: **1.2–1.5 g/kg/day**
- Daily target: **90–110 g protein/day**

Practical distribution:

- Breakfast: 20–25 g
- Lunch: 25–30 g
- Dinner: 25–30 g
- Snacks/late snack: 15–25 g total

Good protein choices include eggs, fish, chicken, lean meat, tofu, soy products, beans, lentils, Greek yogurt, milk, and unsalted nuts. If encephalopathy develops, do not stop protein; consider shifting more protein toward **plant and dairy sources**.

**3. Meal frequency and eating pattern**

Recommend **4–6 eating occasions per day**.

Avoid fasting longer than **10–12 hours**. Cirrhosis causes accelerated starvation physiology, so long overnight fasts promote muscle breakdown. EASL recommends breakfast with protein and a **late-evening snack** to shorten fasting time.

Suggested pattern:

- Breakfast within 1 hour of waking
- Lunch
- Dinner
- 1–2 high-protein snacks
- **Late-evening snack before bed**

Late snack examples:

- Greek yogurt + fruit
- Milk or soy milk + oatmeal
- Peanut butter on unsalted whole-grain toast
- Protein supplement formulated as low-sodium
- Tofu pudding or soy milk + banana

**4. Specific food recommendations and restrictions**

**Sodium restriction for ascites**

Target:

**Sodium <2,000 mg/day**
Equivalent to about **5 g salt/day**, including salt already present in foods.

Avoid or strictly limit:

- Cured meats: ham, bacon, sausage, salami
- Pickles, salted vegetables, kimchi unless low-sodium
- Instant noodles, canned soups, bouillon cubes
- Chips, crackers, salted nuts
- Fast food and restaurant sauces
- Soy sauce, oyster sauce, fish sauce, miso, teriyaki sauce
- Processed cheese and salty packaged meals

Practical substitutions:

- Use herbs, garlic, onion, lemon, vinegar, chili, pepper, cumin, paprika
- Choose “no salt added” canned beans/vegetables
- Rinse canned foods
- Use low-sodium bread and condiments where possible
- Cook at home most days

**Protein foods to encourage**

Daily options:

- Fish, poultry, eggs
- Tofu, tempeh, edamame
- Beans/lentils, preferably cooked without added salt
- Milk, yogurt, Greek yogurt
- Unsalted nuts or nut butter
- Lean pork/beef in moderate portions

**Carbohydrates and fat**

Choose energy-dense but low-sodium foods to prevent muscle loss:

- Rice, oats, potatoes, pasta, whole-grain bread
- Olive oil, avocado, unsalted nuts
- Fruits and vegetables

Avoid very restrictive dieting despite BMI near 30, because ascites can mask muscle wasting.

**Alcohol**

**Complete alcohol abstinence is essential.** No beer, wine, liquor, “small amounts,” or alcohol-containing herbal preparations.

**Food safety**

Because cirrhosis increases infection risk, avoid:

- Raw oysters or shellfish
- Raw or undercooked seafood, meat, or eggs
- Unpasteurized dairy products

**5. Additional nutritional considerations**

**Calories**

Estimate using dry/adjusted weight:

**About 2,100–2,600 kcal/day**
This corresponds roughly to **30–35 kcal/kg/day** depending on estimated dry weight and nutrition status. AASLD educational guidance notes at least **35 kcal/kg/day** for normal BMI and individualized targets for elevated BMI, while accounting for fluid overload.

**Micronutrients**

Alcohol-related cirrhosis commonly involves deficiencies. Check and replace as needed:

- **Thiamine**, especially with alcohol history
- Folate, vitamin B12
- Vitamin D and calcium
- Zinc, especially if poor intake, dysgeusia, diarrhea, or encephalopathy
- Magnesium and phosphate if malnourished or recently abstinent

Avoid high-dose vitamin A or iron unless deficiency is confirmed.

**Ascites monitoring**

Recommend:

- Daily morning weight if feasible
- Track abdominal girth if ascites fluctuates
- Report rapid weight gain, worsening abdominal distension, dyspnea, fever, confusion, GI bleeding, or reduced urine output

Fluid restriction is generally **not indicated** unless serum sodium is low; AASLD-related ascites guidance notes fluid restriction around **1 L/day only when sodium is very low, such as ≤125 mmol/L**.

**Example one-day plan**

Breakfast: oatmeal cooked with milk or soy milk, boiled egg, fruit
Snack: Greek yogurt or unsalted nuts
Lunch: rice, grilled chicken or tofu, vegetables cooked without salt, olive oil/lemon dressing
Snack: low-sodium protein drink or peanut butter on unsalted toast
Dinner: fish, potatoes or rice, vegetables, fruit
Late snack: milk/soy milk plus cereal or yogurt with fruit

**Bottom line:** For this patient, prescribe a **low-sodium, high-protein, frequent-meal diet**: sodium **<2 g/day**, protein **90–110 g/day**, adequate calories, **late-evening snack**, strict alcohol abstinence, and no routine fluid restriction unless hyponatremic.

# C2-04

**Patient summary**

44-year-old woman, 161 cm, 72 kg, HBV cirrhosis with ascites and mild leg edema. Her measured BMI is about **27.8 kg/m²**, but this likely overestimates true nutritional status because ascites/edema add fluid weight. The key goals are to **control ascites without worsening malnutrition**, preserve skeletal muscle, and avoid long fasting.

**1. Overall dietary principles**

Use a **high-protein, adequate-calorie, low-sodium diet** rather than a “liver diet” with broad restrictions.

Current cirrhosis nutrition guidance emphasizes avoiding protein restriction and preventing fasting-related muscle breakdown; ascites guidance generally recommends **moderate sodium restriction** rather than very strict salt avoidance.

Practical targets:

| **Item** | **Daily target** |
| --- | --- |
| Calories | ~**25–35 kcal/kg/day** using estimated dry weight |
| Protein | **1.2–1.5 g/kg/day** |
| Sodium | **<2,000 mg sodium/day**, roughly **≤5 g salt/day** |
| Fluid | **No routine fluid restriction** unless hyponatremia is present |

For her, because current weight includes fluid, I would estimate dry weight around **68–70 kg** unless a true dry weight is available. That gives a calorie goal of roughly **1,700–2,100 kcal/day**, adjusted based on appetite, muscle mass, weight trend, and ascites control.

**2. Recommended daily protein intake**

Recommend **about 85–105 g protein/day**, with a practical target of **90–100 g/day**.

This corresponds to **1.2–1.5 g/kg/day** using estimated dry weight. Protein restriction is **not recommended** in stable cirrhosis, even if encephalopathy occurs; if encephalopathy develops, the approach is usually to maintain protein intake while favoring vegetable, dairy, and possibly branched-chain amino acid–rich sources.

A practical distribution:

| **Meal/snack** | **Protein goal** |
| --- | --- |
| Breakfast | 20–25 g |
| Lunch | 25–30 g |
| Dinner | 25–30 g |
| Snacks, including late snack | 15–25 g total |

Examples of ~20–25 g protein: 2 eggs plus yogurt; 100 g fish/chicken/tofu; 250 mL milk plus Greek yogurt; or beans/lentils with tofu or egg.

**3. Meal frequency and eating pattern**

Recommend **4–6 eating times per day**, including a **late-evening snack**.

Patients with cirrhosis enter a catabolic state after relatively short fasting, so long overnight fasting should be avoided. EASL and ESPEN guidance support frequent meals and a late-evening snack in cirrhosis.

Suggested pattern:

| **Time** | **Example** |
| --- | --- |
| Breakfast | Oats or whole-grain bread + egg/tofu/yogurt |
| Mid-morning snack | Fruit + unsalted nuts or yogurt |
| Lunch | Rice/noodles/potato + fish/chicken/tofu/beans + vegetables |
| Afternoon snack | Milk/yogurt/soy milk + crackers or fruit |
| Dinner | Similar to lunch, low-sodium cooking |
| Late-evening snack | Carbohydrate + protein, e.g., milk + bread, yogurt + banana, oatmeal with milk, or soy milk + steamed bun |

The late snack should ideally contain **carbohydrate plus protein**, not just fruit or tea.

**4. Specific food recommendations and restrictions**

**Encourage**

Choose mostly fresh, minimally processed foods:

- **Protein:** fish, chicken, eggs, tofu, tempeh, soy milk, beans/lentils, low-sodium dairy, lean pork/beef in moderation.
- **Carbohydrates:** rice, noodles, oats, potatoes, sweet potatoes, whole grains as tolerated.
- **Vegetables and fruit:** daily, unless potassium restriction is prescribed because of renal dysfunction or hyperkalemia.
- **Fats:** plant oils, nuts, seeds, avocado; avoid excessive fried foods but do not make the diet too low-fat if calories are inadequate.

**Sodium restriction for ascites/edema**

Aim for **<2 g sodium/day**, roughly **≤5 g salt/day**. This is important for ascites and edema control, but over-restriction can reduce appetite and worsen malnutrition.

Practical low-sodium advice:

- Avoid or greatly limit: pickles, salted fish, cured meats, ham, sausage, bacon, instant noodles, chips, salted nuts, canned soup, hot pot broth, soy sauce-heavy dishes, oyster sauce, fermented bean paste, MSG-heavy seasonings.
- Use: lemon, vinegar, garlic, ginger, scallion, pepper, chili, herbs, spices, low-sodium seasonings.
- Do not use salt substitutes unless her clinician confirms potassium is safe, especially if she is taking spironolactone or has kidney dysfunction.

**Avoid**

- **Alcohol completely.**
- Raw or undercooked seafood, especially raw oysters/shellfish, because cirrhosis increases risk from foodborne infections.
- Unnecessary herbal supplements, “liver detox” products, high-dose vitamins, and unverified protein powders.
- Very-low-protein diets, fasting, crash dieting, or prolonged “cleanses.”

**5. Additional nutritional considerations**

Because she has ascites and edema, monitor nutrition by **dry weight, muscle mass, handgrip/strength, appetite, and dietary intake**, not scale weight alone. Fluid gain can hide muscle loss.

Check and replace deficiencies commonly seen in cirrhosis when clinically indicated: **vitamin D, thiamine, folate, B12, zinc, magnesium**, and iron studies if anemia is present. Fat-soluble vitamins may be relevant if cholestasis is present.

For ascites management, diet should be coordinated with medications such as spironolactone/furosemide. Monitor **serum sodium, potassium, creatinine, albumin, glucose, and weight/abdominal girth**. Fluid restriction is generally reserved for significant hyponatremia, not mild edema alone.

A practical prescription for this patient:

**Low-sodium, high-protein cirrhosis diet: about 1,800–2,100 kcal/day, protein 90–100 g/day, sodium <2,000 mg/day, 4–6 meals/day including a bedtime snack, no alcohol, no prolonged fasting.**

# C2-05

**Patient summary**

48-year-old man, 176 cm, 95 kg, **MASLD cirrhosis with recurrent ascites**. Current BMI is about **30.7 kg/m²**, but ascites likely inflates body weight. For nutrition targets, use **estimated dry weight or ideal/adjusted weight**, not the measured 95 kg.

Estimated ideal body weight at 176 cm is roughly **72–75 kg**.

**1. Overall dietary principles**

Main goals are to **control ascites, prevent sarcopenia, support gradual fat loss, and avoid fasting/catabolism**.

Use a **high-protein, low-sodium, Mediterranean-style diet**:

- **Sodium restriction:** target **≤2,000 mg sodium/day**, equivalent to about **5 g salt/day**, because recurrent ascites is present.
- **Do not restrict protein** unless there is a very specific short-term reason under specialist supervision. Modern cirrhosis nutrition guidance recommends adequate protein, including in patients at risk for hepatic encephalopathy.
- For MASLD/obesity: aim for **slow weight loss**, not crash dieting. A hypocaloric diet combined with high protein is used in obese patients with cirrhosis to promote fat loss while preserving muscle.

**2. Recommended daily protein intake**

Recommended protein for cirrhosis is **1.2–1.5 g/kg/day**; with ascites/obesity, calculate from **dry or ideal body weight**, not actual edematous weight.

For this patient:

**Protein target: about 90–110 g/day**

A practical target would be:

- Minimum: **~90 g/day**
- Preferable if sarcopenia, frailty, frequent hospitalizations, or poor intake: **100–110 g/day**

Distribute protein across the day rather than eating most at dinner:

- Breakfast: 20–25 g
- Lunch: 25–30 g
- Dinner: 25–30 g
- Snacks: 20–30 g total

**3. Meal frequency and eating pattern**

Cirrhosis patients enter a catabolic state after relatively short fasting periods, so avoid long gaps without food. EASL and AASLD-aligned guidance recommends **small frequent meals and a late-evening snack** to reduce overnight catabolism.

Recommended pattern:

- **4–6 eating occasions/day**
- **Breakfast daily**, with protein
- **Late-evening snack** within 1–2 hours before sleep
- Avoid fasting longer than **3–4 hours during the day**
- Avoid skipping breakfast

Good late-evening snack options:

- Greek yogurt or skyr + fruit
- Low-sodium cottage cheese + fruit
- Unsalted peanut butter on whole-grain toast
- Milk or soy milk + oatmeal
- Egg + unsalted whole-grain toast
- Oral nutrition supplement if intake is poor; choose low-sodium/high-protein when possible

**4. Specific food recommendations and restrictions**

**Strongly recommend**

**Protein foods**

- Fish, chicken, turkey, eggs
- Greek yogurt, milk, kefir, low-sodium cottage cheese
- Tofu, tempeh, edamame
- Beans, lentils, chickpeas; rinse canned versions thoroughly or use dried
- Unsalted nuts and nut butters

**Carbohydrates**

- Oats, barley, brown rice, quinoa, whole-grain bread or pasta
- Potatoes/sweet potatoes without added salt
- Fruits and vegetables daily

**Fats**

- Olive oil, avocado, nuts, seeds
- Fatty fish if tolerated

**MASLD-focused pattern**

- Mediterranean-style diet
- High fiber
- Minimize refined starches and added sugar
- Avoid sugar-sweetened beverages and large portions of juice

**Restrict or avoid**

**High-sodium foods**

- Processed meats: ham, bacon, sausage, salami, deli meats
- Canned soups, instant noodles, bouillon, seasoning packets
- Pickles, salted fish, soy sauce, oyster sauce, fish sauce
- Fast food, pizza, frozen meals
- Chips, salted crackers, salted nuts
- Restaurant foods unless specifically prepared low-salt

Practical sodium advice:

- Use “**no added salt**” or “**low sodium**” products.
- Avoid salt substitutes containing potassium unless potassium and renal function are known to be safe, especially if taking spironolactone.
- Flavor with lemon, vinegar, garlic, onion, herbs, pepper, chili, cumin, paprika, ginger.

**Alcohol**

- Complete abstinence is recommended in cirrhosis, even if etiology is MASLD.

**Raw/unsafe foods**

- Avoid raw oysters or raw shellfish due to severe infection risk in chronic liver disease.
- Use careful food safety: fully cooked meats/seafood, pasteurized dairy, clean produce.

**5. Additional nutritional considerations**

**Energy and weight management**

Because he has MASLD and BMI >30, aim for **gradual fat loss while preserving muscle**. Avoid very-low-calorie diets or prolonged fasting.

A practical outpatient calorie range, depending on dry weight, activity, and intake history:

- Often about **1,800–2,100 kcal/day**
- Adjust based on weight trend, ascites control, muscle mass, and appetite

Weight goal:

- Prefer **0.25–0.5 kg/week** fat loss
- Track **waist, strength, function, and dry weight**, not scale weight alone, because ascites can fluctuate.

**Ascites-specific counseling**

- Sodium control is more important than fluid restriction for most ascites patients.
- Fluid restriction is usually reserved for **significant hyponatremia**, not automatically for all ascites patients.
- Coordinate diet with diuretic regimen and monitor sodium, potassium, creatinine, and weight.

**Screen for sarcopenia and micronutrient deficiencies**

Even with obesity, cirrhosis patients can be sarcopenic. Assess grip strength, muscle wasting, gait speed/frailty, diet recall, and consider CT-based muscle assessment if imaging is available.

Consider checking/repleting:

- Vitamin D
- Thiamine if poor intake or any alcohol history
- Folate, B12
- Zinc if dysgeusia, poor intake, diarrhea, or encephalopathy concerns
- Magnesium, potassium, phosphate if diuretics or poor intake

**Exercise pairing**

Nutrition should be paired with activity if clinically safe:

- Resistance exercise **2–3 times/week**
- Walking or aerobic activity most days as tolerated
- Avoid heavy straining if large ascites or hernia risk; tailor to symptoms.

**Practical daily target for this patient**

**Daily prescription**

- Sodium: **≤2,000 mg/day**
- Protein: **90–110 g/day**
- Meals: **3 meals + 2–3 snacks**
- Include: **protein at every meal/snack**
- Late-evening snack: **daily**
- Calories: modest deficit, often **~1,800–2,100 kcal/day**, individualized
- Alcohol: **none**

Example day:

- Breakfast: oatmeal with milk/soy milk + Greek yogurt + berries
- Snack: boiled egg + fruit
- Lunch: grilled chicken/tofu bowl with brown rice, vegetables, olive oil/lemon dressing, no added salt
- Snack: unsalted nuts + yogurt
- Dinner: fish/beans + quinoa or potato + vegetables
- Late snack: Greek yogurt, milk + oats, or peanut butter toast, using low-sodium options.

# C2-06

For this **52-year-old woman, 157 cm, 61 kg, alcohol-related cirrhosis with ascites**, the goals are to prevent sarcopenia/malnutrition, control ascites, and maintain adequate protein intake. In cirrhosis, protein restriction is generally **not recommended**, even with hepatic encephalopathy, because it worsens muscle loss and nitrogen handling. Current guidance supports adequate calories/protein, small frequent meals, and a late-evening snack.

**1. Overall dietary principles**

Use a **high-protein, high-calorie, low-sodium diet**:

- **Energy:** about **30–35 kcal/kg/day**.
  Using actual weight 61 kg: approximately **1,830–2,135 kcal/day**.
  Because she has ascites, “dry weight” may be lower; avoid unnecessary calorie restriction.
- **Protein:** do **not** restrict protein.
- **Sodium:** target **≤2,000 mg sodium/day**, roughly equal to **5 g salt/day**, for ascites control. AASLD notes sodium restriction should be balanced against the risk of poor intake/malnutrition.
- **Fluid:** routine fluid restriction is **not needed** unless she has significant hyponatremia; some guidance restricts only when sodium is very low, for example around **≤125 mmol/L**.
- **Alcohol:** complete abstinence is essential.

**2. Recommended daily protein intake**

Recommended protein for cirrhosis is typically **1.2–1.5 g/kg/day**.

For her:

- Based on **61 kg actual weight:**
  **73–92 g protein/day**
- Because ascites adds fluid weight, a practical target is:
  **75–90 g protein/day**, adjusted upward if sarcopenic or losing muscle.

A simple distribution:

- Breakfast: 20 g
- Lunch: 20–25 g
- Dinner: 25–30 g
- Snacks: 10–20 g total

**3. Meal frequency and eating pattern**

Cirrhosis causes an “accelerated starvation” pattern, so long fasting periods should be avoided.

Recommend:

- **3 meals + 2–3 snacks daily**
- **Late-evening snack every night**, ideally containing carbohydrate plus protein
- Avoid fasting longer than **3–4 hours during the day**
- Eat something before bed, especially if appetite is poor during the day

Examples of late-evening snacks:

- Greek yogurt plus fruit
- Unsalted peanut butter on toast
- Milk or soy milk with oats
- Low-sodium protein shake
- Egg or tofu with rice
- Cottage cheese if low-sodium version is available

Late-evening snacks have evidence for improving nitrogen balance/protein status in cirrhosis.

**4. Specific food recommendations and restrictions**

**Encourage**

**Protein foods**

- Eggs
- Fish, chicken, lean meat
- Tofu, tempeh, soy milk
- Beans/lentils if tolerated
- Greek yogurt, milk, cottage cheese or cheese only if low sodium
- Unsalted nuts or nut butters

**Carbohydrate/energy foods**

- Rice, pasta, oats, potatoes, bread
- Fruit
- Olive oil, avocado, unsalted nuts for added calories
- Oral nutrition supplements if intake is low; choose low-sodium options when possible

**For ascites: low-sodium cooking**

- Use fresh foods rather than packaged foods
- Season with lemon, vinegar, garlic, onion, herbs, pepper, chili, ginger
- Read labels; aim for foods with **<140 mg sodium/serving** when possible
- Choose “no salt added” canned goods and rinse canned beans/vegetables

**Limit or avoid**

**High-sodium foods**

- Processed meats: ham, bacon, sausage, deli meat
- Instant noodles, packaged soups, bouillon cubes
- Pickles, salted fish, salted snacks
- Soy sauce, oyster sauce, fish sauce, teriyaki sauce
- Fast food and restaurant meals
- Regular canned soups and canned meats
- Chips, salted crackers
- High-sodium cheeses

**Alcohol**

- Avoid completely.

**Raw or high-risk seafood**

- Avoid raw oysters/shellfish because cirrhosis increases risk of severe infection, including Vibrio.

**Unnecessary protein restriction**

- Do not reduce protein just because she has cirrhosis. If encephalopathy occurs, prioritize lactulose/rifaximin management and consider more vegetable/dairy protein rather than cutting total protein.

**5. Additional nutritional considerations**

Because this is **alcohol-related cirrhosis**, assess and supplement as clinically indicated:

- **Thiamine** supplementation, especially if any recent alcohol use or poor intake
- **Folate**, **B12**, **magnesium**, **zinc**, and **vitamin D** levels as appropriate
- Screen for **sarcopenia**: handgrip strength, muscle wasting, functional decline
- Consider calcium/vitamin D and bone health assessment, since cirrhosis increases osteoporosis risk
- Monitor weight carefully: with ascites, daily weight reflects fluid as well as nutrition
- After large-volume paracentesis, appetite may improve; reinforce protein intake
- If appetite is poor, use **small, dense meals**: add oil, nut butter, eggs, tofu, yogurt, or oral supplements rather than increasing meal size

**Practical daily target summary**

For this patient:

| **Goal** | **Recommendation** |
| --- | --- |
| Calories | ~1,800–2,100 kcal/day |
| Protein | ~75–90 g/day |
| Sodium | ≤2,000 mg/day sodium, about 5 g salt/day |
| Meals | 3 meals + 2–3 snacks |
| Bedtime snack | Daily, protein + carbohydrate |
| Fluid | Do not restrict unless significant hyponatremia |
| Alcohol | Complete abstinence |

# C2-07

**Dietary and nutritional recommendations: HBV cirrhosis with ascites**

**Patient:** Male, 56 years, 170 cm, 66 kg. Because he has ascites and recent weight fluctuation, nutrition targets should ideally use **estimated dry weight**, but 66 kg can be used as a practical starting point and adjusted with clinical follow-up.

**1. Overall dietary principles**

The main goals are to **prevent muscle loss/sarcopenia, control ascites, maintain adequate calories, and avoid prolonged fasting**. Malnutrition and muscle loss are common in cirrhosis, and current guidance emphasizes adequate protein rather than protein restriction.

Practical targets:

- **Energy:** about **30–35 kcal/kg/day**
  For 66 kg: approximately **2,000–2,300 kcal/day**, adjusted using dry weight and appetite.
- **Protein:** **do not restrict protein routinely**, even in cirrhosis, unless directed temporarily for a specific acute issue.
- **Sodium restriction for ascites:** aim for **≤2 g sodium/day**, roughly equal to **≤5 g salt/day**.
- **Strict alcohol avoidance.**
- Prefer fresh, minimally processed foods; avoid highly salted and preserved foods.

**2. Recommended daily protein intake**

Guidelines recommend **1.2–1.5 g protein/kg/day** for cirrhosis to prevent or reverse muscle loss.

For this patient:

- Using 66 kg:
  **66 × 1.2–1.5 = about 80–100 g protein/day**
- Because ascites can falsely increase body weight, a practical range is:
  **approximately 75–95 g/day**, adjusted to estimated dry weight, renal function, and tolerance.

Protein should be spread across the day, for example:

- Breakfast: 20–25 g
- Lunch: 25–30 g
- Dinner: 25–30 g
- Bedtime snack: 10–15 g

Good protein sources include **fish, poultry, eggs, milk/yogurt, tofu, soy products, beans/lentils, and lean meat**. Vegetable and dairy proteins are often well tolerated.

**3. Meal frequency and eating pattern**

Patients with cirrhosis enter a “fasting-like” metabolic state quickly, so long fasting periods should be avoided. EASL guidance recommends **small frequent meals and a late-evening snack**, ideally including carbohydrate and protein.

Recommended pattern:

- **3 main meals + 2–3 snacks daily**
- Avoid fasting longer than **3–4 hours while awake**
- Include a **late-evening snack before bed**

Examples of bedtime snacks:

- Milk or yogurt + oatmeal/bread
- Soy milk + whole-grain crackers
- Egg or tofu + small bowl of rice/congee
- Greek yogurt + fruit, if tolerated
- Peanut butter on toast, if sodium content is low

**4. Specific food recommendations and restrictions**

**Recommended foods**

**Staple carbohydrates**

- Rice, noodles, oats, potatoes, sweet potatoes, whole grains if tolerated
- Avoid very restrictive low-carb diets.

**Protein**

- Fish, chicken, eggs, tofu, soy milk, beans, lentils
- Low-sodium dairy such as milk, yogurt, or cottage cheese if tolerated
- Lean pork/beef in moderate amounts

**Vegetables and fruits**

- Daily vegetables and fruits for fiber, potassium, and micronutrients
- Use caution with potassium-rich foods only if renal dysfunction or hyperkalemia is present.

**Fats**

- Moderate healthy fats: olive oil, canola oil, nuts, avocado
- Avoid excessive fried foods.

**Sodium restriction for ascites**

Avoid or minimize:

- Pickles, salted vegetables, kimchi, preserved mustard greens
- Cured meats, ham, bacon, sausages, salted fish
- Instant noodles, canned soups, hot pot soup bases
- Soy sauce, oyster sauce, fish sauce, MSG-heavy seasonings
- Chips, crackers, salted nuts
- Restaurant and takeout foods, which are often high sodium

Practical tip: use **vinegar, lemon, garlic, ginger, scallion, herbs, pepper, chili, and spices** instead of salt or soy sauce.

**Fluid restriction**

Routine fluid restriction is **not usually needed for ascites alone**. It is generally considered only if there is **significant hyponatremia**, based on clinician guidance.

**Food safety**

Because cirrhosis increases infection risk:

- Avoid **raw oysters/shellfish**
- Avoid raw or undercooked meat, fish, and eggs
- Use safe food handling and clean water

**5. Additional nutritional considerations**

- **Track dry weight, not just scale weight.** Ascites and diuretics can cause misleading weight changes. Monitor waist circumference, edema, appetite, and muscle strength.
- **Screen for sarcopenia.** Check muscle mass, handgrip strength, gait speed, or functional status when possible.
- **Micronutrients:** consider assessment and supplementation for vitamin D, calcium, thiamine, folate, zinc, and fat-soluble vitamins if intake is poor or deficiencies are suspected.
- **Constipation prevention:** adequate fiber and fluids as clinically appropriate; constipation may worsen hepatic encephalopathy risk.
- **Hepatic encephalopathy:** do not automatically stop protein. If encephalopathy occurs, prioritize regular meals, bowel regimen/lactulose as prescribed, and consider more vegetable/dairy protein.
- **HBV cirrhosis care:** nutrition should be coordinated with antiviral therapy, ascites management, renal function, electrolytes, and hepatocellular carcinoma surveillance.

**Practical daily target for this patient:** about **2,000–2,300 kcal/day**, **80–100 g protein/day**, **≤2 g sodium/day**, divided into **3 meals plus 2–3 snacks including a bedtime snack**.

# C2-08

**Dietary and nutritional recommendations: MASLD cirrhosis with ascites**

**Patient:** Female, 59 years old, 155 cm, 80 kg.
Her BMI is about **33.3 kg/m²**, which is in the obese range. However, because she has **cirrhosis with ascites**, body weight may overestimate nutritional status. The goal is **not rapid weight loss**, but **slow fat loss while preserving or improving muscle mass**.

**1. Overall dietary principles**

Main goals:

- **Control ascites** with sodium restriction.
- **Avoid malnutrition and sarcopenia**, which are common in cirrhosis.
- **Improve MASLD-related metabolic risk** through gradual weight reduction, better glucose control, and healthier fat quality.
- **Avoid fasting and crash diets.**
- **Completely avoid alcohol.**

Recommended daily intake:

- **Energy:** generally around **25–30 kcal/kg/day using estimated dry or adjusted body weight**, not ascites-inflated body weight.
- For practical outpatient counseling, a reasonable starting target is about **1,500–1,800 kcal/day**, individualized based on dry weight, appetite, diabetes status, activity level, and weight trend.
- Weight loss, if appropriate, should be gradual: about **0.5 kg/week or less**, while maintaining adequate protein.

**2. Recommended daily protein intake**

Protein should **not be routinely restricted** in cirrhosis, including in stable patients with ascites.

Recommended protein intake:

- **1.2–1.5 g/kg/day**, preferably using **estimated dry weight or adjusted body weight**.
- Her actual weight is 80 kg, but this may be inflated by ascites.
- A practical daily target is approximately **75–95 g protein/day**.

Protein distribution example:

| **Meal/snack** | **Protein goal** |
| --- | --- |
| Breakfast | 20–25 g |
| Lunch | 25–30 g |
| Dinner | 25–30 g |
| Bedtime snack | 10–15 g |

Good protein choices:

- Fish, skinless poultry, eggs
- Tofu, soy milk, edamame
- Beans and lentils, if tolerated
- Low-fat milk, yogurt, Greek yogurt
- Lean meat in moderate portions

For MASLD and obesity, emphasize **lean proteins and plant proteins**, while avoiding high-fat processed meats.

**3. Meal frequency and eating pattern**

Cirrhosis patients should avoid long fasting intervals because they develop accelerated starvation and muscle breakdown.

Recommended pattern:

- **3 small-to-moderate meals + 2–3 snacks daily**
- Avoid going more than **3–4 hours while awake without food**
- Include a **late-evening snack** before bedtime
- Avoid skipping breakfast
- Avoid extreme low-carbohydrate or ketogenic diets unless specifically supervised

Good bedtime snack examples:

- Unsweetened yogurt + small portion of oats or fruit
- Soy milk + whole-grain crackers
- Low-sodium tofu + small bowl of rice or congee
- Milk + slice of whole-grain toast
- Boiled egg + small serving of carbohydrate

The bedtime snack should contain **carbohydrate plus protein**, not just fruit or tea.

**4. Specific food recommendations and restrictions**

**Sodium restriction for ascites**

Target:

- **≤2,000 mg sodium/day**
- Equivalent to roughly **≤5 g salt/day**

Avoid or strongly limit:

- Pickles, salted vegetables, kimchi, preserved foods
- Salted fish, dried seafood, cured meats
- Ham, bacon, sausage, luncheon meat
- Instant noodles, packaged soups, soup bases
- Chips, salted crackers, salted nuts
- Soy sauce, oyster sauce, fish sauce, chili bean paste
- Restaurant food, takeout, hot pot broth, fast food

Use instead:

- Lemon, vinegar, garlic, ginger, scallion
- Pepper, chili, herbs, spices
- Low-sodium seasonings, used carefully

Do not use potassium-based salt substitutes unless her clinician confirms that **kidney function and potassium are safe**, especially if she takes spironolactone.

**MASLD-focused food pattern**

A Mediterranean-style, lower-sodium pattern is appropriate:

- More vegetables, legumes, whole grains, nuts in controlled portions
- Fish 2–3 times/week if possible
- Olive/canola oil instead of butter or lard
- Low-fat dairy if tolerated
- High-fiber carbohydrates in controlled portions

Limit:

- Sugar-sweetened beverages
- Juice, desserts, candy, sweet pastries
- Refined starches in large portions
- Fried foods
- Processed meats
- Large late-night high-calorie meals

Carbohydrates should be **controlled, not eliminated**, especially if she has diabetes or insulin resistance.

**5. Additional nutritional considerations**

**Ascites and weight monitoring**

- Track **daily morning weight**, edema, abdominal girth, appetite, and urine output if advised.
- Sudden weight changes may reflect fluid shifts rather than fat or muscle change.
- Dietary changes should be coordinated with diuretic therapy and electrolyte monitoring.

**Fluid intake**

- Fluid restriction is **not routinely required** for ascites alone.
- Restrict fluids only if advised, usually for significant hyponatremia.

**Micronutrients**

Consider checking or supplementing when appropriate:

- Vitamin D and calcium
- Zinc
- Thiamine, folate, B vitamins
- Magnesium
- Fat-soluble vitamins if cholestasis or poor intake is present

**Exercise**

Nutrition should be paired with activity as tolerated:

- Walking or low-impact aerobic activity most days
- Light resistance exercise 2–3 times/week if safe
- Avoid prolonged bed rest, which worsens sarcopenic obesity

**Food safety**

Because cirrhosis increases infection risk:

- Avoid raw oysters and raw shellfish
- Avoid undercooked meat, fish, and eggs
- Use safe food handling and clean water

**Practical daily prescription**

For this patient, a reasonable starting plan is:

- **Calories:** about **1,500–1,800 kcal/day**, individualized by dry weight and clinical status
- **Protein:** about **75–95 g/day**
- **Sodium:** **≤2 g/day**
- **Meals:** **3 meals + 2–3 snacks**, including a **protein-containing bedtime snack**
- **Diet pattern:** low-sodium, Mediterranean-style, high-protein, controlled-calorie diet focused on gradual fat loss and muscle preservation.

# C2-09

**Dietary and nutritional recommendations: alcohol-related cirrhosis with ascites and edema**

**Patient:** Male, 63 years old, 167 cm, 60 kg.
Because he has **ascites and edema**, his measured weight may be higher than his true “dry weight.” Nutrition goals should be adjusted using estimated dry weight when available.

**1. Overall dietary principles**

Main goals:

- **Strict lifelong alcohol abstinence**
- **Prevent malnutrition and muscle loss**
- **Control ascites and edema through sodium restriction**
- **Avoid prolonged fasting**
- **Maintain adequate calories and protein**
- Coordinate diet with diuretics, renal function, serum sodium, and potassium monitoring.

Estimated daily energy goal:

- **30–35 kcal/kg/day**
- Using 60 kg: about **1,800–2,100 kcal/day**
- If his dry weight is lower due to fluid retention, adjust accordingly, but avoid aggressive calorie restriction.

Protein should **not be routinely restricted** in stable cirrhosis. Even if hepatic encephalopathy occurs, long-term protein restriction is usually avoided because it worsens sarcopenia.

**2. Recommended daily protein intake**

Recommended protein intake:

- **1.2–1.5 g/kg/day**
- Using 60 kg: **72–90 g protein/day**

Practical target:

- Aim for **75–90 g protein/day**, divided across meals and snacks.

Suggested distribution:

| **Time** | **Protein goal** |
| --- | --- |
| Breakfast | 15–20 g |
| Lunch | 20–25 g |
| Dinner | 25–30 g |
| Snacks / bedtime snack | 15–20 g total |

Good protein choices:

- Fish, chicken, turkey
- Eggs
- Tofu, soy milk, edamame
- Beans, lentils, chickpeas if tolerated
- Milk, yogurt, Greek yogurt
- Lean pork or beef in moderate amounts

If he has poor appetite, use smaller portions more often rather than trying to eat large meals.

**3. Meal frequency and eating pattern**

Cirrhosis patients have reduced glycogen stores and can enter a fasting state quickly.

Recommended pattern:

- **3 meals + 2–3 snacks daily**
- Avoid fasting longer than **3–4 hours while awake**
- Include a **late-evening snack before bed**
- Avoid skipping breakfast
- Consider an early morning snack if overnight fasting is prolonged.

Good bedtime snack examples:

- Milk or yogurt + oats
- Soy milk + low-sodium crackers
- Boiled egg + small bowl of rice or congee
- Tofu + noodles or rice
- Peanut butter on toast, using low-sodium bread if possible

The bedtime snack should include **carbohydrate plus protein**.

**4. Specific food recommendations and restrictions**

**Sodium restriction for ascites and edema**

Target:

- **≤2,000 mg sodium/day**
- Equivalent to about **≤5 g salt/day**

Avoid or strongly limit:

- Pickles, salted vegetables, kimchi, preserved foods
- Salted fish, dried seafood, cured meats
- Ham, bacon, sausage, luncheon meat
- Instant noodles, canned soups, packaged soups
- Hot pot soup bases and broth
- Soy sauce, oyster sauce, fish sauce, chili bean paste
- Chips, salted crackers, salted nuts
- Restaurant food, takeout, fast food

Use instead:

- Lemon, vinegar, garlic, ginger, scallion
- Pepper, chili, cumin, herbs, spices
- Low-sodium seasoning in small amounts

Avoid potassium-based salt substitutes unless his clinician confirms that **kidney function and potassium level are safe**, especially if he takes spironolactone.

**Alcohol-related restrictions**

- **No alcohol at all**, including beer, wine, spirits, medicinal alcohol preparations, and “small amounts.”
- Avoid alcohol-containing tonics or supplements.
- Seek structured alcohol cessation support if relapse risk is present.

**Food choices to emphasize**

- Freshly prepared meals
- Rice, noodles, oats, potatoes, sweet potatoes, whole grains as tolerated
- Vegetables and fruits daily
- Lean proteins at every meal
- Moderate healthy fats such as olive oil, canola oil, avocado, and unsalted nuts

**Foods to avoid for safety**

Because cirrhosis increases infection risk:

- Avoid raw oysters and raw shellfish
- Avoid undercooked meat, fish, and eggs
- Avoid unpasteurized dairy
- Use safe food handling and clean drinking water.

**5. Additional nutritional considerations**

**Thiamine and micronutrients**

Alcohol-related cirrhosis commonly involves vitamin and mineral deficiencies. Assess and supplement as appropriate:

- **Thiamine / vitamin B1**
- Folate
- Vitamin B6 and B12
- Vitamin D and calcium
- Magnesium
- Zinc
- Fat-soluble vitamins if cholestasis or poor intake is present

Thiamine is especially important if intake is poor or there is ongoing/recent alcohol use.

**Fluid intake**

- Fluid restriction is **not routinely required** for ascites alone.
- Consider fluid restriction only if there is clinically significant hyponatremia and the treating team recommends it.

**Monitoring**

Track:

- Morning weight
- Abdominal girth
- Leg edema
- Appetite and oral intake
- Muscle strength and walking capacity
- Serum sodium, potassium, creatinine, albumin, and glucose as clinically indicated

**If appetite is poor**

Use nutrient-dense, low-sodium options:

- Oral nutrition supplements designed for liver disease or high-protein formulas, preferably low sodium
- Homemade shakes with milk/yogurt/soy milk plus oats or fruit
- Add unsalted nut butter, olive oil, or powdered milk when extra calories are needed

**Physical activity**

If medically safe:

- Daily walking or light activity
- Resistance exercise 2–3 times/week
- Avoid prolonged bed rest, which rapidly worsens muscle loss.

**Practical daily prescription**

For this patient:

- **Calories:** about **1,800–2,100 kcal/day**
- **Protein:** about **75–90 g/day**
- **Sodium:** **≤2 g/day**
- **Alcohol:** **complete abstinence**
- **Meal pattern:** **3 meals + 2–3 snacks**, including a **carbohydrate + protein bedtime snack**
- **Focus:** low-sodium, high-protein, frequent-meal diet to control ascites/edema while preventing malnutrition and sarcopenia.

# C2-10

**ietary and nutritional recommendations: HBV cirrhosis with ascites and poor appetite**

**Patient:** Female, 67 years old, 160 cm, 52 kg.
Because she has **ascites**, her measured weight may overestimate true dry weight. She also has **poor appetite**, so she is at high risk for inadequate intake, malnutrition, and sarcopenia.

**1. Overall dietary principles**

Main goals:

- **Prevent or treat malnutrition and muscle loss**
- **Control ascites with sodium restriction**
- **Increase intake despite poor appetite**
- **Avoid prolonged fasting**
- Maintain antiviral and hepatology follow-up for HBV cirrhosis
- Avoid alcohol completely

Estimated daily intake:

- **Energy:** about **30–35 kcal/kg/day**
- Using 52 kg: approximately **1,550–1,800 kcal/day**
- If her dry weight is lower because of ascites, adjust clinically, but avoid unnecessary calorie restriction.

Diet should be:

- **High-protein**
- **Low-sodium**
- **Small, frequent, energy-dense**
- **Easy to chew and digest**
- Based on fresh foods rather than processed foods

**2. Recommended daily protein intake**

Protein should **not be routinely restricted** in cirrhosis. Adequate protein is especially important because she is older and has poor appetite.

Recommended protein intake:

- **1.2–1.5 g/kg/day**
- Using 52 kg: **62–78 g protein/day**

Practical target:

- Aim for about **65–80 g protein/day**, divided into small portions.

Suggested distribution:

| **Time** | **Protein goal** |
| --- | --- |
| Breakfast | 15–20 g |
| Lunch | 20 g |
| Dinner | 20 g |
| Snacks / bedtime snack | 15–20 g total |

Good protein choices:

- Eggs
- Fish
- Soft tofu, soy milk, edamame
- Chicken, lean meat, minced meat
- Milk, yogurt, Greek yogurt
- Beans or lentils if tolerated

For poor appetite, protein should be included in every eating opportunity, even if portions are small.

**3. Meal frequency and eating pattern**

Because cirrhosis increases fasting-related muscle breakdown, she should avoid long gaps without food.

Recommended pattern:

- **5–6 small meals/snacks per day**
- Eat every **2–3 hours while awake**
- Avoid skipping breakfast
- Include a **late-evening snack before bed**
- Use small bowls and nutrient-dense foods rather than large meals

Good bedtime snack options:

- Warm milk or soy milk + soft bread
- Yogurt + oats
- Egg custard + small portion of rice/congee
- Tofu pudding or soft tofu + carbohydrate
- Low-sodium oral nutrition supplement
- Peanut butter on low-sodium toast, if tolerated

The bedtime snack should contain both **carbohydrate and protein**.

**4. Specific food recommendations and restrictions**

**Sodium restriction for ascites**

Target:

- **≤2,000 mg sodium/day**
- Equivalent to about **≤5 g salt/day**

Avoid or strongly limit:

- Pickles, salted vegetables, kimchi
- Salted fish, dried seafood, preserved meats
- Ham, bacon, sausage, luncheon meat
- Instant noodles, canned soups, packaged soup bases
- Soy sauce, oyster sauce, fish sauce, chili bean paste
- Salted crackers, chips, salted nuts
- Restaurant food, takeout, hot pot broth

Use instead:

- Lemon, vinegar, garlic, ginger, scallion
- Herbs, pepper, chili, cumin, sesame, spices
- Low-sodium seasonings in small amounts

Avoid potassium-based salt substitutes unless kidney function and serum potassium are confirmed safe, especially if she uses spironolactone.

**Foods to emphasize for poor appetite**

Choose soft, high-protein, energy-dense foods:

- Egg custard, boiled eggs, omelet with little/no salt
- Steamed fish
- Tofu, soy milk, tofu pudding without excess sugar
- Yogurt or milk
- Chicken porridge with added egg or tofu
- Minced meat or fish congee
- Oatmeal made with milk or soy milk
- Smoothies made with yogurt/soy milk and fruit
- Unsalted nut butter, olive oil, or canola oil added to foods in small amounts

Ways to increase calories without increasing meal size:

- Add milk powder to porridge or oatmeal
- Add olive/canola oil to rice, noodles, or vegetables
- Use yogurt or soy milk instead of water in snacks
- Add egg, tofu, minced fish, or chicken to congee
- Use low-sodium oral nutrition supplements if intake remains poor

**Foods to avoid for food safety**

Cirrhosis increases infection risk, so avoid:

- Raw oysters and raw shellfish
- Raw or undercooked meat, fish, and eggs
- Unpasteurized dairy products
- Unsafe water or high-risk street foods

**5. Additional nutritional considerations**

**Poor appetite management**

- Encourage intake at the time of day when appetite is best.
- Liquids may be easier than solids; use nutrient-rich drinks rather than plain tea or water.
- Avoid filling up on soup, tea, or water before meals.
- Consider low-sodium, high-protein oral nutrition supplements if she cannot meet needs with food.
- If early satiety from ascites is severe, optimize ascites treatment with the hepatology team.

**Micronutrients**

Assess and supplement as needed:

- Vitamin D and calcium
- Zinc
- Thiamine and other B vitamins
- Folate
- Magnesium
- Fat-soluble vitamins if cholestasis, poor intake, or fat malabsorption is present

**Fluid intake**

- Fluid restriction is **not routinely needed** for ascites alone.
- Restrict fluids only if significant hyponatremia is present and her clinician recommends it.

**Monitoring**

Follow:

- Estimated dry weight
- Daily or regular morning weight
- Abdominal girth and leg swelling
- Food intake and appetite
- Muscle strength, gait speed, grip strength if available
- Sodium, potassium, creatinine, glucose, and albumin as clinically indicated

**Physical activity**

If medically safe:

- Gentle walking daily
- Light resistance exercise or chair exercises
- Avoid prolonged bed rest, which worsens muscle loss

**Practical daily prescription**

For this patient:

- **Calories:** about **1,550–1,800 kcal/day**
- **Protein:** about **65–80 g/day**
- **Sodium:** **≤2 g/day**
- **Meal pattern:** **5–6 small meals/snacks daily**, including a **protein + carbohydrate bedtime snack**
- **Diet focus:** low-sodium, high-protein, soft, small-volume, energy-dense meals to manage ascites while improving poor appetite and preventing sarcopenia.

# C2-11

**Dietary and nutritional recommendations: MASLD cirrhosis with recurrent ascites**

**Patient:** Male, 71 years old, 169 cm, 78 kg.
His BMI is about **27.3 kg/m²**, but because he has **recurrent ascites**, measured weight may overestimate true dry weight. At age 71, he is also at high risk for **sarcopenia**, so nutrition should focus on **muscle preservation, ascites control, and cautious metabolic management**, not rapid weight loss.

**1. Overall dietary principles**

Main goals:

- **Control recurrent ascites** with consistent sodium restriction.
- **Prevent sarcopenia and malnutrition**, especially in an older patient.
- Support MASLD management by improving diet quality, glucose control, and fat quality.
- Avoid prolonged fasting.
- Avoid alcohol completely.
- Coordinate diet with diuretics, renal function, potassium, sodium level, and paracentesis history.

Estimated daily energy goal:

- In cirrhosis, usual target is about **30–35 kcal/kg/day** using estimated dry weight.
- Because he has MASLD and possible overweight, use **estimated dry weight or adjusted body weight** rather than ascites-inflated scale weight.
- Practical outpatient starting target: about **1,800–2,100 kcal/day**, adjusted based on dry weight, muscle mass, appetite, diabetes status, and weight trend.

Weight loss, if needed, should be **slow and supervised**. Avoid crash diets, ketogenic diets, prolonged fasting, and very-low-calorie diets.

**2. Recommended daily protein intake**

Protein should **not be routinely restricted** in cirrhosis, even with a history of ascites. Adequate protein is essential to prevent muscle loss.

Recommended protein intake:

- **1.2–1.5 g/kg/day**
- Use estimated dry or adjusted body weight when possible.
- Using 78 kg gives **94–117 g/day**, but this may be too high if weight includes ascites.
- A practical daily target is approximately **85–105 g protein/day**, individualized to dry weight, renal function, and tolerance.

Suggested distribution:

| **Time** | **Protein goal** |
| --- | --- |
| Breakfast | 20–25 g |
| Lunch | 25–30 g |
| Dinner | 25–30 g |
| Snacks / bedtime snack | 15–25 g total |

Good protein choices:

- Fish, skinless poultry, eggs
- Tofu, soy milk, edamame
- Beans, lentils, chickpeas if tolerated
- Low-fat milk, yogurt, Greek yogurt
- Lean meat in moderate portions

For MASLD, emphasize **lean animal protein, fish, low-fat dairy, and plant protein**, while limiting processed meats.

**3. Meal frequency and eating pattern**

Cirrhosis patients should avoid long fasting intervals because this accelerates muscle breakdown.

Recommended pattern:

- **3 meals + 2–3 snacks daily**
- Avoid fasting longer than **3–4 hours while awake**
- Include a **late-evening snack before bed**
- Avoid skipping breakfast
- Avoid large, salty restaurant meals that worsen fluid retention

Good bedtime snack examples:

- Unsweetened yogurt + oats or fruit
- Soy milk + whole-grain low-sodium crackers
- Milk + whole-grain toast
- Tofu + small bowl of rice or noodles
- Boiled egg + small serving of carbohydrate
- Low-sodium high-protein oral nutrition supplement if intake is poor

The bedtime snack should include both **carbohydrate and protein**.

**4. Specific food recommendations and restrictions**

**Sodium restriction for recurrent ascites**

Target:

- **≤2,000 mg sodium/day**
- Equivalent to about **≤5 g salt/day**

This should be followed consistently every day, including weekends and restaurant meals.

Avoid or strongly limit:

- Pickles, salted vegetables, kimchi, preserved foods
- Salted fish, dried seafood, cured meats
- Ham, bacon, sausage, luncheon meat
- Instant noodles, canned soups, packaged soups
- Hot pot broth, soup bases, bouillon cubes
- Soy sauce, oyster sauce, fish sauce, chili bean paste
- Chips, salted crackers, salted nuts
- Fast food, takeout, restaurant soups and sauces

Use instead:

- Lemon, vinegar, garlic, ginger, scallion
- Pepper, chili, cumin, herbs, spices
- Small amounts of low-sodium seasoning if approved

Avoid potassium-based salt substitutes unless his clinician confirms that **serum potassium and kidney function are safe**, especially if taking spironolactone.

**MASLD-focused food pattern**

Use a **low-sodium Mediterranean-style pattern**:

Emphasize:

- Vegetables daily
- Whole grains in controlled portions
- Beans/lentils if tolerated
- Fish 2–3 times per week
- Skinless poultry
- Tofu and soy products
- Low-fat dairy
- Olive oil or canola oil in moderate amounts
- Unsalted nuts in small portions

Limit:

- Sugar-sweetened beverages
- Fruit juice
- Desserts, candy, sweet pastries
- Large portions of refined rice/noodles/bread
- Fried foods
- Processed meats
- High-saturated-fat foods

Carbohydrates should be **portion-controlled**, not eliminated. This is especially important if he has diabetes or insulin resistance.

**5. Additional nutritional considerations**

**Recurrent ascites**

- Monitor daily morning weight if advised.
- Track abdominal girth, leg edema, appetite, and urine output.
- Sudden weight gain usually reflects fluid, not fat.
- Nutrition should be coordinated with diuretic dose, renal function, sodium, potassium, and need for paracentesis.

**Fluid restriction**

- Fluid restriction is **not routinely required** for ascites alone.
- Restrict fluids only if there is significant hyponatremia and the treating clinician recommends it.

**After large-volume paracentesis**

- Appetite may improve after fluid removal; use that period to increase protein intake.
- Continue strict sodium restriction to reduce recurrence.
- Follow medical advice regarding albumin infusion and diuretics.

**Sarcopenia prevention**

At age 71, muscle preservation is a priority:

- Protein at each meal
- Bedtime snack daily
- Avoid prolonged bed rest
- Gentle walking and resistance exercise as tolerated
- Consider physical therapy if frail or weak

**Micronutrients**

Consider checking or supplementing:

- Vitamin D and calcium
- Zinc
- B vitamins
- Magnesium
- Folate
- Fat-soluble vitamins if cholestasis or poor intake is present

**Food safety**

Because cirrhosis increases infection risk:

- Avoid raw oysters and raw shellfish
- Avoid raw or undercooked meat, fish, and eggs
- Avoid unpasteurized dairy
- Use safe food handling and clean drinking water

**Practical daily prescription**

For this patient:

- **Calories:** about **1,800–2,100 kcal/day**, individualized by estimated dry weight and metabolic status
- **Protein:** about **85–105 g/day**
- **Sodium:** **≤2 g/day**
- **Meal pattern:** **3 meals + 2–3 snacks daily**, including a **carbohydrate + protein bedtime snack**
- **Diet focus:** low-sodium, high-protein, Mediterranean-style diet that controls recurrent ascites while preserving muscle and improving MASLD-related metabolic risk.

# C2-12

**Dietary and nutritional recommendations: alcohol-related cirrhosis with ascites**

**Patient:** Female, 74 years old, 156 cm, 64 kg.
Because she has **ascites**, measured body weight may overestimate true dry weight. At age 74, she is at high risk for **malnutrition and sarcopenia**, so the priority is **adequate protein, adequate calories, strict sodium control, and complete alcohol abstinence**.

**1. Overall dietary principles**

Main goals:

- **Complete alcohol abstinence**
- **Control ascites with sodium restriction**
- **Prevent muscle loss and frailty**
- **Avoid prolonged fasting**
- Maintain adequate energy and protein intake
- Coordinate diet with diuretics, renal function, electrolytes, and ascites management

Estimated daily energy goal:

- **30–35 kcal/kg/day**, preferably using estimated dry weight
- Using current weight 64 kg: about **1,900–2,200 kcal/day**
- If ascites is significant, use estimated dry weight for adjustment, but avoid unnecessary calorie restriction.

Diet pattern:

- **Low sodium**
- **High protein**
- **Small frequent meals**
- Nutrient-dense foods
- Safe, freshly prepared foods

**2. Recommended daily protein intake**

Protein should **not be routinely restricted** in cirrhosis. Older patients with cirrhosis need enough protein to reduce muscle loss.

Recommended intake:

- **1.2–1.5 g/kg/day**
- Using 64 kg: **77–96 g protein/day**

Practical daily target:

- Aim for about **75–95 g protein/day**, adjusted to estimated dry weight, kidney function, and tolerance.

Suggested distribution:

| **Time** | **Protein goal** |
| --- | --- |
| Breakfast | 15–20 g |
| Lunch | 20–25 g |
| Dinner | 25–30 g |
| Snacks / bedtime snack | 15–20 g total |

Good protein choices:

- Fish, chicken, turkey
- Eggs
- Tofu, soy milk, edamame
- Beans, lentils, chickpeas if tolerated
- Milk, yogurt, Greek yogurt
- Lean meat in moderate amounts

If she has history of hepatic encephalopathy, do **not** automatically stop protein; consider more vegetable and dairy protein and coordinate with medical therapy.

**3. Meal frequency and eating pattern**

Cirrhosis patients should avoid long fasting periods because muscle breakdown occurs quickly.

Recommended pattern:

- **3 meals + 2–3 snacks daily**
- Eat every **3–4 hours while awake**
- Include a **late-evening snack before bed**
- Avoid skipping breakfast
- Use smaller meals if ascites causes early fullness

Good bedtime snack examples:

- Milk or soy milk + oats
- Yogurt + fruit or soft bread
- Egg custard + rice/congee
- Tofu + small bowl of noodles or rice
- Low-sodium high-protein oral nutrition supplement
- Peanut butter on low-sodium toast, if tolerated

The bedtime snack should contain both **carbohydrate and protein**.

**4. Specific food recommendations and restrictions**

**Sodium restriction for ascites**

Target:

- **≤2,000 mg sodium/day**
- Equivalent to about **≤5 g salt/day**

Avoid or strongly limit:

- Pickles, salted vegetables, kimchi, preserved foods
- Salted fish, dried seafood, cured meats
- Ham, bacon, sausage, luncheon meat
- Instant noodles, canned or packaged soups
- Hot pot broth, bouillon cubes, soup bases
- Soy sauce, oyster sauce, fish sauce, chili bean paste
- Chips, salted crackers, salted nuts
- Fast food, restaurant food, takeout

Use instead:

- Lemon, vinegar, garlic, ginger, scallion
- Herbs, pepper, chili, cumin, turmeric, spices
- Low-sodium seasonings in small amounts if needed

Avoid potassium-containing salt substitutes unless kidney function and potassium are confirmed safe, especially if taking spironolactone.

**Foods to emphasize**

Choose nutrient-dense, low-sodium foods:

- Rice, noodles, oats, potatoes, sweet potatoes
- Whole grains if tolerated
- Fresh vegetables and fruits
- Fish, poultry, eggs, tofu, soy products
- Low-sodium dairy
- Olive oil, canola oil, avocado
- Unsalted nuts or nut butter in small portions

For poor appetite or early satiety:

- Add egg, tofu, minced fish, or chicken to porridge/congee
- Use milk or soy milk instead of water in oatmeal
- Add olive/canola oil to cooked vegetables, rice, or noodles
- Choose small, soft, high-protein meals

**Alcohol-related restrictions**

- **No alcohol at all**, including beer, wine, spirits, “small amounts,” medicinal alcohol, and alcohol-containing tonics.
- Consider structured alcohol cessation support, counseling, or addiction medicine follow-up if relapse risk is present.
- Avoid herbal or “liver detox” products, which may worsen liver injury.

**Food safety**

Because cirrhosis increases infection risk:

- Avoid raw oysters and raw shellfish
- Avoid raw or undercooked meat, fish, and eggs
- Avoid unpasteurized dairy products
- Use clean drinking water and safe food handling

**5. Additional nutritional considerations**

**Micronutrients**

Alcohol-related cirrhosis is commonly associated with deficiencies. Assess and supplement as clinically appropriate:

- **Thiamine / vitamin B1**
- Folate
- Vitamin B6 and B12
- Vitamin D and calcium
- Magnesium
- Zinc
- Fat-soluble vitamins if cholestasis or poor intake is present

**Fluid intake**

- Fluid restriction is **not routinely needed** for ascites alone.
- Restrict fluids only if significant hyponatremia is present and her treating clinician recommends it.

**Monitoring**

Track:

- Estimated dry weight rather than fluid-influenced scale weight alone
- Morning weight
- Abdominal girth
- Leg edema
- Appetite and food intake
- Muscle strength, walking ability, falls risk
- Sodium, potassium, creatinine, glucose, and albumin as clinically indicated

**Exercise and function**

If medically safe:

- Gentle walking daily
- Chair exercises or resistance-band exercises
- Balance training to reduce fall risk
- Avoid prolonged bed rest, which accelerates muscle loss

**Practical daily prescription**

For this patient:

- **Calories:** about **1,900–2,200 kcal/day**, adjusted by estimated dry weight and intake tolerance
- **Protein:** about **75–95 g/day**
- **Sodium:** **≤2 g/day**
- **Alcohol:** **complete abstinence**
- **Meal pattern:** **3 meals + 2–3 snacks daily**, including a **carbohydrate + protein bedtime snack**
- **Diet focus:** low-sodium, high-protein, nutrient-dense frequent meals to control ascites and prevent frailty, malnutrition, and sarcopenia.

# C3-01

For this **35-year-old man, 171 cm/65 kg**, BMI is about **22.2 kg/m²**, so he is not underweight by BMI, but cirrhosis patients can still have **sarcopenia/muscle loss**. Because he has had **hepatic encephalopathy (HE)** but is now recovered, the key message is: **do not chronically restrict protein**. Current cirrhosis nutrition guidance recommends **1.2–1.5 g/kg/day protein**, and low-protein diets should be avoided in HE because they can worsen malnutrition and muscle loss.

**1. Overall dietary principles**

Aim for a **high-protein, adequate-calorie, plant-forward diet** with frequent meals.

Estimated daily targets for him:

| **Target** | **Practical goal** |
| --- | --- |
| Energy | about **30–35 kcal/kg/day = 1,950–2,275 kcal/day** |
| Protein | **1.2–1.5 g/kg/day = 78–98 g/day** |
| Pattern | **4–6 meals/snacks/day**, avoid long fasting |
| Late snack | Every night, preferably carbohydrate + protein |

The goal is to maintain or rebuild muscle, prevent fasting-related muscle breakdown, and reduce recurrence risk related to poor nutrition. Frequent meals and a late-evening snack are specifically recommended in cirrhosis to shorten overnight fasting.

**2. Recommended daily protein intake**

**Recommended protein: 78–98 g/day.**

A practical prescription would be:

**Target: about 90 g protein/day**, divided across the day.

Example distribution:

| **Time** | **Protein goal** |
| --- | --- |
| Breakfast | 20 g |
| Lunch | 25 g |
| Dinner | 25 g |
| Snacks/late snack | 20 g |
| **Total** | **~90 g/day** |

Because of prior HE, prioritize **vegetable and dairy proteins**, which are often better tolerated than large amounts of red meat. Guidelines note that, rather than reducing total protein, patients with HE may benefit from shifting toward **milk-based or vegetable protein**, and branched-chain amino acid formulas can be considered when adequate intake is difficult.

Good protein choices:

- Eggs, fish, poultry in moderate portions
- Tofu, soy milk, tempeh, beans, lentils, peas
- Milk, yogurt, Greek yogurt, cheese in reasonable amounts
- Nuts and nut butters, if tolerated
- Oral nutrition supplements if intake is poor

Avoid telling him to “eat less protein” unless he has an acute HE flare and his treating team gives short-term instructions. Even then, restriction should not be prolonged.

**3. Meal frequency and eating pattern**

He should avoid skipping meals and avoid fasting longer than about **3–4 hours during the day**.

Recommended pattern:

- **Breakfast**
- **Mid-morning snack**
- **Lunch**
- **Afternoon snack**
- **Dinner**
- **Late-evening snack before bed**

The **late-evening snack** is important. It can be something like:

- Yogurt + banana
- Milk or soy milk + whole-grain bread
- Oatmeal with milk
- Tofu pudding or soy milk + crackers
- Rice porridge with egg or tofu
- Oral nutrition supplement if appetite is poor

A useful bedtime snack target is **~200–300 kcal**, with **carbohydrate plus 10–15 g protein**.

**4. Specific food recommendations and restrictions**

**Encourage**

**Protein foods daily**

- Tofu/soy products, beans, lentils
- Milk, yogurt, eggs
- Fish or lean poultry
- Moderate portions of meat if tolerated, but avoid making red meat the main protein source

**Carbohydrates at each meal**

- Rice, noodles, oats, potatoes, corn, whole-grain bread
- Do not use a very-low-carb diet; cirrhosis patients are prone to accelerated starvation physiology.

**Vegetables and fruit**

- Aim for vegetables at lunch and dinner.
- Fruit 1–2 times/day, adjusted if diabetes is present.

**Healthy fats**

- Vegetable oils, nuts, seeds, avocado, oily fish if tolerated.

**Restrict or avoid**

**Alcohol: completely avoid.**
No safe amount of alcohol should be recommended in cirrhosis.

**Excess sodium**

- If he has ascites or leg edema: target **sodium <2 g/day**, roughly equivalent to **salt <5 g/day**.
- Avoid salted/preserved foods: pickles, cured meats, instant noodles, salted fish, soy sauce-heavy meals, chips, processed snacks.
- If no ascites/edema, still avoid very salty foods, but overly strict salt restriction may reduce intake.

**Raw or unsafe seafood**

- Avoid raw oysters, raw shellfish, and unsafe raw seafood because cirrhosis increases infection risk.

**Large red meat loads**

- Not absolutely forbidden, but avoid large portions, especially if they seem to trigger confusion, constipation, or sleep-wake reversal.

**Constipation**

- Prevent constipation because it can precipitate HE. Use adequate fiber, fluids as allowed, and follow lactulose/rifaximin prescriptions if prescribed.

**5. Additional nutritional considerations**

Monitor for **sarcopenia** even with normal BMI. Ask about strength, walking speed, falls, appetite, and weight trend. Consider checking handgrip strength or muscle mass if available. Malnutrition and sarcopenia are common in cirrhosis and can occur even when BMI looks normal.

Consider supplementation if intake is inadequate:

- **Oral nutrition supplement**: useful if he cannot reach calories/protein from food.
- **BCAA supplement**: may be considered if he cannot tolerate adequate dietary protein or has recurrent HE despite standard management; guidelines mention BCAA-enriched formulas as an option in HE nutrition support.
- Check and replace deficiencies as clinically indicated: **vitamin D, thiamine, folate, zinc, magnesium**, and fat-soluble vitamins if cholestasis is present.
- Encourage **resistance exercise** if medically stable: walking plus light resistance training helps preserve muscle; coordinate with hepatology if decompensated.

**One-day practical example aiming for ~90 g protein**

| **Meal** | **Example** |
| --- | --- |
| Breakfast | Oatmeal with milk + egg, or soy milk + whole-grain bread + egg |
| Snack | Yogurt or soy milk |
| Lunch | Rice + tofu/beans + fish/chicken + vegetables |
| Snack | Fruit + nuts, or milk/soy milk |
| Dinner | Noodles/rice + tofu/egg/fish + vegetables |
| Bedtime snack | Yogurt + banana, or milk/soy milk + bread/crackers |

**Bottom line:** For this patient, prescribe **adequate calories, 78–98 g protein/day, 4–6 eating occasions daily, and a mandatory late-evening snack**. Because of previous HE, choose more **vegetable and dairy protein**, avoid constipation and alcohol, and do **not** chronically restrict protein.

# C3-02

For this **38-year-old woman, 158 cm, 53 kg, alcohol-related cirrhosis with prior hepatic encephalopathy**, the main nutrition goal is to **prevent malnutrition and muscle loss**, because fasting and under-eating can worsen sarcopenia and may increase encephalopathy risk. Modern cirrhosis guidance does **not** recommend routine protein restriction in hepatic encephalopathy.

**1. Overall dietary principles**

Aim for a **high-protein, adequate-energy, small-frequent-meal diet**:

- **Energy:** about **30–35 kcal/kg/day** → for 53 kg, approximately **1,600–1,850 kcal/day**.
- **Protein:** do **not restrict protein** despite prior hepatic encephalopathy; inadequate protein may worsen nutrition and muscle loss.
- Avoid prolonged fasting, especially overnight. Cirrhotic patients should keep fasting periods short and include a late-evening snack.
- **Complete alcohol abstinence** is essential given alcohol-related cirrhosis.

**2. Recommended daily protein intake**

Recommended protein intake is **1.2–1.5 g/kg/day**. For this patient:

**53 kg × 1.2–1.5 g/kg/day = about 64–80 g protein/day**

A practical target would be **70–75 g/day**, divided across meals and snacks.

Example distribution:

| **Eating time** | **Protein target** |
| --- | --- |
| Breakfast | 15–20 g |
| Lunch | 20 g |
| Dinner | 20 g |
| Snacks / bedtime snack | 15–20 g total |

**3. Meal frequency and eating pattern**

Recommend **4–6 eating occasions per day**, rather than 2–3 large meals.

Suggested pattern:

- Breakfast soon after waking
- Lunch
- Afternoon snack
- Dinner
- **Late-evening snack before bed**, ideally containing carbohydrate plus protein

Examples of late-evening snacks:

- Yogurt or milk + oatmeal/bread
- Soy milk + whole-grain toast
- Egg + small bowl of rice/congee
- Tofu pudding or tofu + crackers
- Nutrition supplement if oral intake is poor

The late-evening snack is important because cirrhosis is associated with an accelerated starvation state overnight; guidelines recommend reducing fasting time with frequent meals and a bedtime snack.

**4. Specific food recommendations and restrictions**

**Protein foods to encourage**

Because she has prior hepatic encephalopathy, prioritize **vegetable and dairy protein**, which may be better tolerated in patients with protein intolerance or encephalopathy risk.

Good choices:

- **Dairy:** milk, yogurt, cheese, high-protein yogurt
- **Soy/legumes:** tofu, soy milk, edamame, lentils, beans
- **Eggs**
- **Fish and poultry**
- Moderate lean meat if tolerated, but avoid relying only on red meat
- Oral nutrition supplements if she cannot meet targets with food

**Carbohydrates and fats**

- Include carbohydrates at each meal: rice, noodles, oats, potatoes, bread, fruit.
- Choose unsaturated fats: olive oil, canola oil, nuts, seeds, avocado, fish.
- Do not follow a very low-carb or fasting diet.

**Sodium**

If she has **ascites or leg edema**, use a **low-sodium diet**, typically around **2 g sodium/day**, but avoid making the diet so unpalatable that intake falls. ESPEN specifically cautions that sodium restriction can reduce food intake and should be balanced against nutritional risk.

Practical sodium advice:

- Avoid pickles, salted fish, cured meats, instant noodles, chips, canned soups, and heavily seasoned restaurant foods.
- Use herbs, vinegar, lemon, garlic, ginger, pepper, and spices instead of salt-heavy sauces.
- Avoid “low-salt” potassium salt substitutes unless potassium and renal function are confirmed safe.

**Avoid or limit**

- **Alcohol: absolutely avoid**
- Raw or undercooked seafood, especially shellfish, due to infection risk in cirrhosis
- Large amounts of sugary drinks or desserts, especially if glucose intolerance/diabetes is present
- Unsupervised herbal supplements or “liver detox” products
- Prolonged fasting, meal skipping, crash dieting

**5. Additional nutritional considerations**

Given alcohol-related cirrhosis, assess and replete micronutrients commonly deficient in this setting:

- **Thiamine vitamin B1**, especially if any ongoing alcohol use, poor intake, vomiting, or hospitalization risk
- Folate, vitamin B12
- Vitamin D and calcium
- Zinc, particularly if taste changes, poor appetite, diarrhea, or recurrent encephalopathy
- Fat-soluble vitamins A, D, E, K if cholestasis or malabsorption is present

Also recommend:

- Screen for **sarcopenia/muscle loss** even though BMI is normal; her BMI is about **21.2 kg/m²**, so normal weight does not exclude malnutrition.
- Encourage safe resistance exercise and walking if clinically stable, because nutrition plus exercise is more effective for muscle maintenance.
- Continue prescribed hepatic encephalopathy therapy, such as lactulose/rifaximin, and avoid constipation.
- Monitor weight trends, appetite, handgrip strength or functional status, serum sodium, potassium, renal function, glucose, and ascites/edema status.

**Practical one-day example**

- **Breakfast:** egg + yogurt or milk + oatmeal/congee
- **Snack:** soy milk or yogurt + fruit
- **Lunch:** rice/noodles + tofu/fish/chicken + vegetables + oil for calories
- **Snack:** nuts or cheese/yogurt, depending on sodium allowance
- **Dinner:** rice/potato + fish/tofu/egg + vegetables
- **Bedtime snack:** milk/soy milk/yogurt + bread/oats/crackers

**Daily target:** about **1,600–1,850 kcal** and **64–80 g protein**, preferably closer to **70–75 g/day**, spread evenly across the day.

# C3-03

**Patient summary**

43-year-old man, 169 cm, 80 kg, **BMI ≈ 28.0 kg/m²**: overweight MASLD cirrhosis with prior hepatic encephalopathy. The key goal is **preserve or rebuild muscle while improving metabolic risk**, not “low-protein dieting.”

**1. Overall dietary principles**

Use a **high-protein, Mediterranean-style, low-ultra-processed diet**:

- **Do not restrict protein because of prior hepatic encephalopathy.** Current cirrhosis nutrition guidance emphasizes that protein restriction can worsen malnutrition/sarcopenia, and recommended intake is generally **1.2–1.5 g/kg/day**; AASLD guidance also notes protein should not be restricted in hepatic encephalopathy.
- Aim for **gradual fat loss if clinically stable**, but avoid crash dieting, prolonged fasting, or very-low-calorie diets.
- Prefer: vegetables, legumes, whole grains, nuts, fish, poultry, eggs, low-fat dairy or yogurt, tofu/soy products, and olive/canola-type oils.
- Reduce: sugary drinks, desserts, refined starches, processed meats, fried foods, and high-saturated-fat foods.
- **Absolute alcohol avoidance.**
- Maintain good glycemic control if he has diabetes/prediabetes, common in MASLD and associated with worse outcomes.

**2. Recommended daily protein intake**

For this overweight patient, calculate protein using **ideal or target body weight**, not necessarily actual 80 kg.

Estimated ideal/target weight for 169 cm is roughly **70–72 kg**.

Recommended protein:
**1.2–1.5 g/kg/day × 70–72 kg = about 85–105 g protein/day.**

Practical target: **90–100 g/day**, divided across meals.

Example distribution:

| **Time** | **Protein goal** |
| --- | --- |
| Breakfast | 20–25 g |
| Lunch | 25–30 g |
| Dinner | 25–30 g |
| Late-evening snack | 15–20 g |

Good protein choices for prior HE: **plant and dairy proteins are often better tolerated**, such as tofu, soy milk, beans/lentils, Greek yogurt, milk, eggs, fish, and lean poultry. ESPEN guidance notes low protein intake may worsen HE, while higher protein intake can improve mental state in some patients.

**3. Meal frequency and eating pattern**

Cirrhosis creates an “accelerated starvation” state, so spacing meals matters.

Recommended pattern:

- **3 main meals + 2–3 snacks daily**
- Avoid fasting longer than **3–4 hours during the day**
- Add a **late-evening snack** before bed, ideally with complex carbohydrate plus protein
- Eat breakfast soon after waking

Late-evening snack examples:

- Greek yogurt + oats or fruit
- Soy milk + whole-grain toast
- Tofu pudding or tofu + small bowl of rice
- Milk + unsweetened cereal
- Peanut butter on whole-grain bread
- Lentil/bean soup if tolerated

Frequent meals and minimizing fasting, including a late-night snack, are recommended in cirrhosis nutrition guidance to reduce muscle breakdown.

**4. Specific food recommendations and restrictions**

**Encourage daily**

**Protein**

- Fish, chicken, eggs
- Tofu, soy products, beans, lentils
- Low-fat milk, yogurt, Greek yogurt, cheese in moderation
- Nuts and seeds in small portions

**Carbohydrates**

- Whole grains: oats, brown rice, whole-wheat bread, barley, quinoa
- Avoid large loads of white rice/noodles alone; pair carbs with protein and vegetables.

**Vegetables and fruit**

- At least **500 g vegetables/day** if tolerated
- 1–2 servings fruit/day; choose whole fruit rather than juice.

**Fats**

- Prefer unsaturated fats: olive oil, canola oil, nuts, avocado, fish
- Limit butter, lard, cream, fatty meats.

**Restrict or avoid**

- **Alcohol: none**
- **Sugary drinks and fruit juice**
- **Processed meats**: sausage, bacon, ham, luncheon meat
- **High-salt foods**, especially if ascites/edema is present: pickles, instant noodles, chips, salted nuts, cured meats, hot pot soup base, soy sauce-heavy dishes
- Raw or undercooked shellfish/seafood because cirrhosis increases risk of severe infection.

**Sodium**

If he has **ascites or edema**, use a sodium target of about **2 g sodium/day**, equivalent to about **5 g salt/day**. Do not over-restrict food variety so much that protein/calorie intake falls. If no ascites or edema, still avoid very salty processed foods, but the plan can be less rigid.

**5. Additional nutritional considerations**

**Weight and MASLD target**

Because BMI is 28, a reasonable goal is **slow weight loss**, about **5–10% over 6–12 months**, while maintaining muscle. The priority is **waist reduction plus strength preservation**, not rapid scale loss.

Avoid:

- skipping dinner
- fasting diets
- ketogenic crash diets
- juice cleanses
- “detox” supplements

**Hepatic encephalopathy prevention**

- Maintain **adequate protein**, especially plant/dairy protein.
- Prevent constipation; aim for regular bowel movements as directed with lactulose if prescribed.
- Continue lactulose/rifaximin as prescribed.
- Consider **branched-chain amino acid supplementation** only if recurrent HE or protein intolerance persists despite diet optimization; this should be individualized.

**Micronutrients and bone health**

Patients with cirrhosis are prone to deficiencies and sarcopenia. Consider checking and replacing as needed:

- Vitamin D
- Calcium intake, usually through diet first
- Zinc if poor intake, dysgeusia, diarrhea, or recurrent HE
- B vitamins, especially if poor intake
- Magnesium, phosphate, potassium if on diuretics or poor intake

Bone density assessment is often appropriate in cirrhosis, especially with long-standing disease or transplant evaluation.

**Exercise pairing**

Nutrition works best with activity:

- **Resistance training 2–3 times/week**, adjusted to physical capacity
- Walking or aerobic activity most days
- Avoid heavy lifting if large varices, severe thrombocytopenia, or uncontrolled ascites unless cleared clinically.

**Practical daily prescription for this patient**

**Daily targets:**

- Protein: **90–100 g/day**
- Meals: **3 meals + 2–3 snacks**
- Late-evening snack: **mandatory**
- Pattern: Mediterranean/MASLD diet, high protein, high fiber, low added sugar
- Sodium: **≤2 g/day only if ascites/edema; otherwise avoid high-salt processed foods**
- Alcohol: **0**
- Goal: gradual fat loss while preserving muscle mass.

# C3-04

**Dietary and nutritional plan for C3-04**

**Patient:** Female, 44 years, 160 cm, 68 kg, HBV cirrhosis, recovering from hepatic encephalopathy.
**BMI:** ~26.6 kg/m², but in cirrhosis use **dry weight** if ascites or edema is present.

Current EASL/ESPEN guidance supports **adequate calories, no routine protein restriction in hepatic encephalopathy, frequent meals, and a late-evening snack** to reduce fasting-related muscle breakdown. Patients with HE should avoid long fasting and split calories/protein across small meals.

**1. Overall dietary principles**

Use a **high-protein, adequate-energy, low-salt, balanced diet** aimed at preventing sarcopenia and recurrent HE.

Daily targets:

| **Item** | **Recommendation for this patient** |
| --- | --- |
| Energy | About **30–35 kcal/kg/day** = **~2,000–2,400 kcal/day** using 68 kg; adjust lower if true obesity, higher if malnourished |
| Protein | **1.2–1.5 g/kg/day** = **~82–102 g/day** using 68 kg |
| Sodium | If ascites/edema/hypertension: **≤2 g sodium/day**, roughly **≤5 g salt/day** |
| Fluids | Do **not** restrict routinely; restrict only if severe hyponatremia or clinician-directed |
| Alcohol | **Absolute avoidance** |

**2. Recommended daily protein intake**

Recommend **~90 g protein/day**, divided across meals and snacks.

Do **not** restrict protein just because she had hepatic encephalopathy. Protein restriction can worsen muscle loss, and muscle helps clear ammonia. ESPEN recommends **1.2–1.5 g/kg/day** protein for cirrhosis; EASL advises frequent protein-containing meals and snacks in HE.

Preferred protein sources:

- **Dairy:** milk, yogurt, kefir, low-salt cheese
- **Eggs**
- **Soy products:** tofu, soy milk, edamame
- **Fish and poultry**
- **Legumes:** lentils, beans, chickpeas, if tolerated
- Moderate lean red meat only if tolerated; avoid large portions at one sitting

If animal protein seems to worsen confusion or bloating, shift more protein toward **vegetable and dairy protein**. If she cannot reach protein goals or has recurrent HE despite standard care, consider **BCAA-enriched oral nutrition supplements** under clinician/dietitian guidance.

**3. Meal frequency and eating pattern**

Recommend **4–6 small meals/day**, including breakfast and a **late-evening snack**.

A practical pattern:

| **Time** | **Example** |
| --- | --- |
| Breakfast | Oatmeal + egg + milk or soy milk |
| Mid-morning | Yogurt or tofu pudding + fruit |
| Lunch | Rice/noodles + fish/chicken/tofu + vegetables |
| Afternoon snack | Unsalted nuts or milk/yogurt |
| Dinner | Staple food + tofu/fish/egg + vegetables |
| Late-evening snack | Milk/yogurt + bread, oats, sweet potato, or crackers |

The late-evening snack should contain **carbohydrate plus protein**, for example:

- Milk + whole-grain bread
- Yogurt + banana
- Soy milk + steamed bun
- Oatmeal with milk
- Tofu pudding + small portion of rice cake

This reduces overnight fasting and muscle breakdown, which is important in cirrhosis.

**4. Specific food recommendations and restrictions**

**Encourage**

- Adequate staple foods: rice, noodles, oats, potatoes, sweet potatoes, whole grains if tolerated
- High-quality protein at every meal
- Vegetables and fruits daily, unless potassium restriction is required
- Calcium/vitamin D-rich foods: milk, yogurt, fortified soy milk
- Healthy fats in moderation: olive oil, canola oil, nuts, seeds
- Soft, easy-to-digest foods during HE recovery if appetite is poor

**Restrict or avoid**

- **Alcohol completely**
- High-salt foods: pickles, salted fish, cured meats, instant noodles, preserved vegetables, soy sauce-heavy dishes, hotpot soup base, processed snacks
- Raw or unsafe foods: raw seafood, undercooked eggs/meat, unpasteurized dairy, due to infection risk in cirrhosis
- Very large meat-heavy meals
- Constipation-promoting pattern: low fiber, low fluid, skipped meals
- Unsupervised herbal products or “liver detox” supplements

For seasoning, use vinegar, lemon, garlic, ginger, scallion, pepper, herbs, and low-sodium seasonings rather than salt-heavy sauces.

**5. Additional nutritional considerations**

Because she is recovering from hepatic encephalopathy:

- **Prevent constipation:** regular meals, fiber as tolerated, adequate fluid; coordinate with lactulose/rifaximin plan if prescribed.
- **Monitor mental status:** if sleepiness, confusion, personality change, or asterixis recurs, seek medical care promptly.
- **Check for sarcopenia:** ask about grip strength, walking speed, muscle loss. Nutrition plus light resistance exercise can help if medically stable.
- **Micronutrients:** assess vitamin D, calcium, zinc, folate, B vitamins, and fat-soluble vitamins if cholestasis or poor intake is present.
- **HBV cirrhosis:** maintain antiviral therapy follow-up; diet does not replace HBV treatment.
- **Weight management:** BMI is mildly elevated, but avoid crash dieting. The priority is preserving muscle while avoiding excess salt and empty calories.

**Practical daily protein distribution target**

Aim for roughly:

- Breakfast: **15–20 g**
- Lunch: **25–30 g**
- Dinner: **25–30 g**
- Snacks including bedtime snack: **20–25 g total**

Total: **~85–100 g/day**.

# C3-05

**Dietary and nutritional recommendations for C3-05**

**Patient:** Male, 49 years old, 174 cm, 90 kg.
**Diagnosis:** Alcohol-related cirrhosis with prior hepatic encephalopathy.
**BMI:** ~29.7 kg/m², overweight/obesity range. In cirrhosis, use **dry weight** if ascites or edema is present.

For this patient, the goals are to **prevent recurrent hepatic encephalopathy, preserve muscle mass, avoid alcohol completely, control sodium intake, and avoid long fasting**.

**1. Overall dietary principles**

Recommend a **high-protein, adequate-energy, low-sodium, alcohol-free diet**.

Key principles:

- **Do not restrict protein routinely**, even with prior hepatic encephalopathy. Protein restriction may worsen sarcopenia and increase HE risk.
- Provide adequate calories, but avoid excessive energy intake because he is overweight.
- Emphasize **vegetable and dairy proteins**, which are often better tolerated in patients with HE.
- Avoid long fasting; include a **late-evening snack**.
- Strict **lifelong alcohol abstinence** is essential.
- If ascites or edema is present, restrict sodium.

Guidelines support **30–35 kcal/kg/day energy** and **1.2–1.5 g/kg/day protein** in cirrhosis, with small frequent meals and a late-evening snack. ESPEN also states that patients with hepatic encephalopathy should not have routine protein restriction, and EASL recommends splitting caloric and protein intake into small frequent meals.

**2. Recommended daily protein intake**

Because his actual body weight is high, I would estimate protein using **ideal or adjusted body weight**, unless he is clearly sarcopenic.

- Height 174 cm
- Ideal body weight roughly **70 kg**
- Protein target: **1.2–1.5 g/kg/day**
- Practical daily target: **85–105 g protein/day**

A reasonable outpatient target is **about 90–100 g protein/day**, divided across the day.

Example distribution:

| **Meal/snack** | **Protein target** |
| --- | --- |
| Breakfast | 20 g |
| Lunch | 25–30 g |
| Dinner | 25–30 g |
| Snacks, including bedtime snack | 20–25 g |
| **Total** | **90–105 g/day** |

If he has obvious muscle wasting, recent weight loss, poor appetite, or recurrent HE, aim closer to the higher end, around **100–105 g/day**.

**3. Meal frequency and eating pattern**

Recommend **4–6 meals/snacks per day**, including a **late-evening snack**.

Suggested pattern:

| **Time** | **Example** |
| --- | --- |
| Breakfast | Oats or whole-grain bread + egg/low-fat milk/soy milk |
| Mid-morning | Yogurt, milk, or soy milk |
| Lunch | Rice/noodles/potato + tofu/fish/chicken + vegetables |
| Afternoon snack | Fruit + yogurt or unsalted nuts |
| Dinner | Staple food + vegetable/dairy protein or lean animal protein + vegetables |
| Late-evening snack | Milk/yogurt/soy milk + oats, bread, sweet potato, or crackers |

The **late-evening snack** is important in cirrhosis because overnight fasting promotes muscle breakdown. EASL recommends breakfast and late-evening snacks containing some protein, and ESPEN supports late-evening snacks in cirrhosis including alcoholic cirrhosis.

Good bedtime snack options:

- Milk + whole-grain bread
- Yogurt + banana
- Soy milk + oatmeal
- Tofu pudding + steamed bun
- Low-salt crackers + milk
- Oral nutrition supplement if intake is poor

**4. Specific food recommendations and restrictions**

**Recommended foods**

**Protein sources**

Prioritize:

- Tofu, soy milk, edamame
- Beans, lentils, chickpeas if tolerated
- Milk, yogurt, kefir
- Eggs
- Fish
- Skinless poultry
- Small portions of lean meat if tolerated

For prior HE, use more **plant and dairy protein** and avoid very large meat-heavy meals.

**Carbohydrates**

Include adequate complex carbohydrates:

- Rice, noodles, oats
- Potatoes, sweet potatoes
- Whole grains if tolerated
- Bread or steamed buns

Avoid very-low-carbohydrate dieting or prolonged fasting, because this can worsen catabolism.

**Fats**

Use moderate healthy fats:

- Olive oil, canola oil
- Nuts and seeds, preferably unsalted
- Fish rich in omega-3 fatty acids

**Foods and drinks to avoid or restrict**

**Absolutely avoid**

- Alcohol in all forms, including beer, wine, spirits, “medicinal alcohol,” and alcohol-containing tonics

**Limit sodium, especially if ascites/edema**

Aim for:

- **≤2 g sodium/day**, approximately **≤5 g salt/day**

Avoid or sharply limit:

- Pickles, salted fish, cured meat, bacon, sausage
- Instant noodles
- Preserved vegetables
- Hotpot soup base
- Soy sauce-heavy dishes
- Processed snacks
- Canned soups and processed ready meals

**Avoid infection-risk foods**

Patients with cirrhosis are more vulnerable to infection. Avoid:

- Raw oysters or raw seafood
- Undercooked meat
- Undercooked eggs
- Unpasteurized dairy products

**Avoid unsafe supplements**

Avoid:

- “Liver detox” products
- Unverified herbal medicines
- High-dose vitamin A, niacin, or iron unless prescribed
- Bodybuilding supplements or alcohol-containing extracts

**5. Additional nutritional considerations**

**Hepatic encephalopathy prevention**

- Maintain regular bowel movements.
- Avoid constipation.
- Continue lactulose/rifaximin if prescribed.
- Seek care promptly if confusion, reversed sleep rhythm, personality change, drowsiness, or hand flapping recurs.
- Avoid large high-meat meals; distribute protein throughout the day.

If protein intolerance occurs, ESPEN recommends using **vegetable protein or BCAA** to help achieve adequate protein intake; long-term oral BCAA may be considered in advanced cirrhosis under medical supervision.

**Alcohol-related cirrhosis considerations**

Because this is alcohol-related cirrhosis, assess and supplement as needed:

- **Thiamine, vitamin B1**
- Folate
- Vitamin B6 and B12
- Vitamin D and calcium
- Magnesium, phosphate, zinc

Thiamine is especially important in patients with current or prior heavy alcohol use.

**Weight and muscle management**

Although BMI is high, cirrhosis patients may still have **sarcopenic obesity**. Do not use crash diets.

Practical goal:

- Preserve or build muscle
- Avoid sugary drinks and excess refined snacks
- Use adequate protein
- Add safe physical activity if medically cleared, especially walking plus light resistance exercise

**Monitoring**

Follow regularly for:

- Weight using **dry weight**
- Appetite and food intake
- Muscle loss or grip strength
- Ascites/edema
- Serum sodium, potassium, magnesium, phosphate
- Glucose control
- Vitamin D and zinc if clinically indicated

**Practical daily target for this patient**

A realistic prescription:

- **Energy:** about **2,100–2,400 kcal/day**, adjusted to dry weight, activity, ascites, and weight trend
- **Protein:** **90–100 g/day**
- **Meals:** **3 meals + 2–3 snacks/day**
- **Bedtime snack:** every night
- **Sodium:** **≤2 g/day if ascites/edema**
- **Alcohol:** **0 intake**

# C3-06

**Dietary and nutritional recommendations for C3-06**

**Patient:** Female, 53 years old, 157 cm, 58 kg.
**Diagnosis:** MASLD cirrhosis with prior hepatic encephalopathy and mild weight loss.
**BMI:** ~23.5 kg/m², but the key issue is **recent weight loss**, which suggests nutritional risk and possible early sarcopenia.

The main goals are to **prevent recurrent hepatic encephalopathy, stop further weight and muscle loss, maintain adequate protein intake, improve metabolic health, and avoid long fasting**.

**1. Overall dietary principles**

Recommend a **high-protein, adequate-energy, Mediterranean-style, low-sodium diet**, with small frequent meals.

Key principles:

- **Do not restrict protein** because of prior hepatic encephalopathy.
- Prioritize **muscle preservation** because she has mild weight loss.
- Avoid fasting, skipped meals, and very-low-carbohydrate dieting.
- Use more **plant and dairy protein**, which may be better tolerated in patients with prior HE.
- For MASLD, avoid excess sugar, refined carbohydrates, and saturated fat, but **do not pursue aggressive weight loss** at this stage.
- If ascites or edema is present, restrict sodium.

Guidelines recommend **1.2–1.5 g/kg/day protein** in cirrhosis to prevent or reverse muscle loss, and note that normal-to-high protein intake does not precipitate hepatic encephalopathy.

**2. Recommended daily protein intake**

Use her current weight of **58 kg**, unless significant fluid retention is present; if ascites or edema is present, use estimated **dry weight**.

Recommended protein:

**1.2–1.5 g/kg/day × 58 kg = ~70–87 g protein/day**

Because she has **mild weight loss**, a practical target is:

**75–85 g protein/day**

Suggested distribution:

| **Meal/snack** | **Protein target** |
| --- | --- |
| Breakfast | 15–20 g |
| Lunch | 20–25 g |
| Dinner | 20–25 g |
| Snacks, including bedtime snack | 15–20 g |
| **Total** | **75–85 g/day** |

Protein should be spread across the day rather than concentrated in one large meal.

**3. Meal frequency and eating pattern**

Recommend **4–6 eating occasions per day**, including a **late-evening snack**.

This is especially important in cirrhosis because even an overnight fast can increase muscle breakdown. ESPEN supports a late-evening snack in cirrhosis to improve protein status.

Suggested daily pattern:

| **Time** | **Example** |
| --- | --- |
| Breakfast | Oats + milk/soy milk + egg |
| Mid-morning | Yogurt or fortified soy milk + fruit |
| Lunch | Rice/noodles/whole grain + tofu/fish/chicken + vegetables |
| Afternoon snack | Unsalted nuts + fruit, or yogurt |
| Dinner | Staple food + protein dish + vegetables |
| Late-evening snack | Milk/yogurt/soy milk + oats, bread, crackers, or sweet potato |

Good bedtime snack options:

- Yogurt + banana
- Milk + whole-grain bread
- Soy milk + oatmeal
- Tofu pudding + small steamed bun
- Low-salt crackers + milk
- Oral nutrition supplement if appetite is poor

The bedtime snack should include **carbohydrate plus protein**, not just fruit or tea.

**4. Specific food recommendations and restrictions**

**Recommended foods**

**Protein sources**

Prioritize:

- Tofu, soy milk, edamame
- Beans, lentils, chickpeas, if tolerated
- Milk, yogurt, kefir
- Eggs
- Fish
- Skinless poultry
- Small portions of lean meat if tolerated

For prior hepatic encephalopathy, use more **vegetable and dairy proteins**, and avoid very large meat-heavy meals. A recent ACG guideline summary also notes that protein should not be restricted in cirrhosis with HE, and that vegetable protein may be better tolerated in HE.

**Carbohydrates**

Choose mostly low-glycemic, fiber-containing carbohydrates:

- Oats
- Brown rice or mixed grains if tolerated
- Whole-grain bread
- Sweet potato, potato, corn
- Beans and lentils
- Fruit in moderate portions

Avoid strict ketogenic or very-low-carbohydrate diets, because they can worsen catabolism in cirrhosis.

**Fats**

Use mostly unsaturated fats:

- Olive oil or canola oil
- Nuts and seeds, preferably unsalted
- Fatty fish in moderate portions

Limit:

- Fried foods
- Fatty meat
- Butter, cream, lard
- Pastries and ultra-processed snacks

**Restrictions**

**Alcohol**

- Avoid completely.

**Sodium**

If she has ascites, edema, or hypertension:

- Aim for **≤2 g sodium/day**, roughly **≤5 g salt/day**.

Limit:

- Pickles, salted vegetables, salted fish
- Sausage, bacon, cured meat
- Instant noodles
- Hotpot soup base
- Soy sauce-heavy dishes
- Canned soups
- Processed snacks

**Food safety**

Avoid:

- Raw seafood, especially raw shellfish
- Undercooked meat or eggs
- Unpasteurized dairy
- Unclean street foods or foods stored unrefrigerated for long periods

**Supplements and herbs**

Avoid unprescribed “liver detox,” high-dose vitamin A, high-dose niacin, iron supplements, and herbal products with uncertain ingredients.

**5. Additional nutritional considerations**

**Energy intake**

Because she has mild weight loss, avoid calorie restriction. A practical energy target is:

**30–35 kcal/kg/day × 58 kg = ~1,750–2,030 kcal/day**

Start around **1,800–2,000 kcal/day**, then adjust based on weight trend, ascites, appetite, glucose control, and activity.

**Hepatic encephalopathy prevention**

- Maintain regular bowel movements.
- Avoid constipation.
- Continue lactulose and/or rifaximin if prescribed.
- Seek care if confusion, reversed sleep rhythm, personality change, drowsiness, or hand tremor/flapping recurs.
- Avoid large single protein loads; distribute protein across the day.

**MASLD-specific points**

Because this is MASLD cirrhosis, focus on **metabolic quality**, not rapid weight loss:

- Avoid sugary drinks, juice, desserts, and frequent refined snacks.
- Choose high-fiber carbohydrates in controlled portions.
- Include protein at each meal to stabilize glucose and preserve muscle.
- Screen and manage diabetes, dyslipidemia, and hypertension.
- Encourage safe physical activity if medically stable, especially walking plus light resistance exercise.

**Micronutrients**

Assess and supplement if deficient:

- Vitamin D and calcium
- Zinc, especially if recurrent HE, poor appetite, or taste changes
- Folate and B vitamins
- Magnesium and phosphate if poor intake
- Fat-soluble vitamins if cholestasis is present

**When oral intake is poor**

If she cannot consistently meet **75–85 g protein/day** or continues losing weight:

- Add 1–2 oral nutrition supplements daily.
- Consider a **BCAA-enriched supplement**, especially if HE recurs or protein intake is difficult.
- Refer to a liver dietitian for individualized meal planning.

**Practical daily prescription**

For this patient, a clear outpatient target would be:

- **Energy:** about **1,800–2,000 kcal/day**
- **Protein:** **75–85 g/day**
- **Meals:** **3 meals + 2–3 snacks/day**
- **Bedtime snack:** every night
- **Protein type:** emphasize **plant, dairy, fish, eggs, and poultry**
- **Sodium:** **≤2 g/day if ascites/edema**
- **Alcohol:** **0 intake**
- **Weight goal:** stop unintentional weight loss and preserve muscle, rather than intentional dieting right now.

# C3-07

**Dietary and nutritional recommendations for C3-07**

**Patient:** Male, 57 years old, 168 cm, 62 kg
**Diagnosis:** HBV cirrhosis with previous hepatic encephalopathy
**BMI:** ~22.0 kg/m², within normal range. In cirrhosis, weight may be misleading if ascites or edema is present, so use **estimated dry weight** when calculating needs.

The main goals are to **prevent recurrent hepatic encephalopathy, maintain muscle mass, avoid malnutrition, and support liver disease management**.

**1. Overall dietary principles**

Recommend an **adequate-energy, high-protein, low-sodium, balanced diet** with frequent meals.

Key points:

- **Do not routinely restrict protein**, even with previous hepatic encephalopathy.
- Avoid fasting and skipped meals.
- Include protein at each meal and snack.
- Emphasize **plant and dairy protein**, with fish, eggs, and poultry as additional options.
- Restrict sodium if ascites, edema, or hypertension is present.
- Avoid alcohol completely.
- Continue regular HBV and cirrhosis follow-up; diet does not replace antiviral therapy or HE medications.

**2. Recommended daily protein intake**

For cirrhosis, recommend:

**1.2–1.5 g/kg/day protein**

Using current body weight:

**62 kg × 1.2–1.5 g/kg/day = ~74–93 g protein/day**

A practical target for this patient:

**80–90 g protein/day**

Suggested distribution:

| **Eating occasion** | **Protein target** |
| --- | --- |
| Breakfast | 15–20 g |
| Lunch | 20–25 g |
| Dinner | 20–25 g |
| Snacks, including bedtime snack | 20 g |
| **Total** | **80–90 g/day** |

If he has sarcopenia, poor appetite, or recent weight loss, aim closer to **90–95 g/day**.

**3. Meal frequency and eating pattern**

Recommend:

**3 main meals + 2–3 snacks daily**

He should avoid fasting longer than about **4–6 hours during the day** and should take a **late-evening snack** before sleep.

Suggested daily pattern:

| **Time** | **Example** |
| --- | --- |
| Breakfast | Oats or rice porridge + egg + milk/soy milk |
| Mid-morning snack | Yogurt, milk, soy milk, or fruit with nuts |
| Lunch | Rice/noodles + tofu/fish/chicken + vegetables |
| Afternoon snack | Yogurt, tofu pudding, unsalted nuts, or whole-grain crackers |
| Dinner | Staple food + protein dish + vegetables |
| Late-evening snack | Milk/soy milk/yogurt + bread, oats, crackers, or sweet potato |

The late-evening snack should include **carbohydrate plus protein**, for example:

- Milk + whole-grain bread
- Soy milk + oatmeal
- Yogurt + banana
- Tofu pudding + steamed bun
- Low-salt crackers + milk

**4. Specific food recommendations and restrictions**

**Recommended foods**

**Protein sources**

Prioritize:

- Tofu, soy milk, edamame
- Beans, lentils, chickpeas, if tolerated
- Milk, yogurt, kefir
- Eggs
- Fish
- Skinless poultry
- Small portions of lean pork or beef if tolerated

For patients with previous HE, **plant and dairy proteins** may be better tolerated than large meat-heavy meals.

**Staple carbohydrates**

Include adequate carbohydrates to prevent muscle breakdown:

- Rice, noodles, oats
- Potatoes, sweet potatoes, corn
- Whole grains if tolerated
- Bread or steamed buns

Avoid very-low-carbohydrate or ketogenic dieting.

**Vegetables and fruits**

- Include vegetables daily.
- Include fruit in moderate portions.
- Adjust potassium intake only if hyperkalemia or kidney disease is present.

**Fats**

Use moderate amounts of healthy fats:

- Canola oil, olive oil
- Unsalted nuts and seeds
- Fish

Limit fried foods and excessive animal fat.

**Restrictions**

**Alcohol**

- **Complete avoidance**.

**Sodium**

If ascites, edema, or hypertension is present:

**≤2 g sodium/day, approximately ≤5 g salt/day**

Limit or avoid:

- Pickles and preserved vegetables
- Salted fish, cured meats, sausage, bacon
- Instant noodles
- Hotpot soup base
- Soy sauce-heavy dishes
- Canned soups
- Processed snacks

**Food safety**

Avoid:

- Raw seafood, especially raw shellfish
- Undercooked meat
- Undercooked eggs
- Unpasteurized dairy
- Foods left at room temperature for long periods

**Supplements and herbs**

Avoid unsupervised:

- “Liver detox” products
- Herbal remedies with unclear ingredients
- High-dose vitamin A
- High-dose niacin
- Iron supplements unless iron deficiency is confirmed

**5. Additional nutritional considerations**

**Energy intake**

For stable cirrhosis, estimate:

**30–35 kcal/kg/day**

Using 62 kg:

**~1,860–2,170 kcal/day**

A practical target:

**~1,900–2,100 kcal/day**

Adjust based on dry weight, physical activity, ascites, glucose control, and weight trend.

**Hepatic encephalopathy prevention**

Because he has previous HE:

- Maintain regular bowel movements.
- Avoid constipation.
- Take lactulose and/or rifaximin as prescribed.
- Avoid large protein loads at one meal.
- Do not skip meals.
- Seek medical care if confusion, excessive sleepiness, reversed sleep rhythm, personality change, or hand flapping recurs.

If protein intake worsens HE symptoms or he cannot meet targets, consider:

- Shifting more protein to **vegetable and dairy sources**
- Oral nutrition supplements
- **BCAA-enriched supplements** under clinician/dietitian supervision

**HBV cirrhosis considerations**

- Continue antiviral therapy if prescribed.
- Maintain regular monitoring for liver function, HBV DNA, varices, ascites, and hepatocellular carcinoma surveillance.
- Avoid alcohol and hepatotoxic medications unless specifically approved.

**Micronutrients**

Assess and supplement if deficient:

- Vitamin D and calcium
- Zinc, especially if recurrent HE, poor appetite, or taste changes
- Folate and B vitamins
- Magnesium and phosphate if intake is poor
- Fat-soluble vitamins if cholestasis is present

**Physical activity**

If medically stable:

- Encourage regular walking.
- Add light resistance exercise to preserve muscle mass.
- Avoid prolonged bed rest unless medically necessary.

**Practical daily prescription**

For this patient:

- **Energy:** about **1,900–2,100 kcal/day**
- **Protein:** **80–90 g/day**
- **Meal pattern:** **3 meals + 2–3 snacks/day**
- **Bedtime snack:** every night
- **Preferred protein:** tofu/soy, dairy, eggs, fish, poultry
- **Sodium:** **≤2 g/day if ascites/edema**
- **Alcohol:** **0**
- **Main priority:** prevent recurrent HE while preserving muscle and avoiding malnutrition.

# C3-08

**Dietary and nutritional recommendations for C3-08**

**Patient:** Female, 60 years old, 156 cm, 76 kg
**Diagnosis:** Alcohol-related cirrhosis with history of hepatic encephalopathy
**BMI:** ~31.2 kg/m², obesity range. In cirrhosis, use **dry weight** if ascites or edema is present.

Main goals: **prevent recurrent hepatic encephalopathy, preserve muscle mass, avoid alcohol completely, control sodium, and avoid long fasting**. Even though BMI is high, she may still have **sarcopenic obesity**, so protein should not be restricted.

**1. Overall dietary principles**

Recommend an **alcohol-free, high-protein, nutritionally balanced, low-sodium diet**, with frequent small meals.

Key principles:

- **No alcohol at all**: beer, wine, spirits, alcohol-containing tonics, and “medicinal alcohol” should be avoided.
- **Do not routinely restrict protein**, even with previous hepatic encephalopathy.
- Avoid fasting, skipped meals, and crash dieting.
- Prioritize **plant and dairy proteins**, with fish, eggs, and poultry as additional protein sources.
- Reduce sodium, especially if ascites, edema, or hypertension is present.
- Improve diet quality: more vegetables, legumes, whole grains as tolerated; fewer sugary drinks, sweets, fried foods, and ultra-processed foods.

**2. Recommended daily protein intake**

Because actual weight is high, estimate protein using **ideal or adjusted body weight**, especially if there is obesity or fluid retention.

For height **156 cm**, estimated ideal body weight is about **50–55 kg**.

Recommended protein in cirrhosis:

**1.2–1.5 g/kg/day**

Practical target:

**70–85 g protein/day**

If she has clear muscle loss, poor intake, recurrent HE, or recent weight loss, aim closer to **85–90 g/day**.

Suggested distribution:

| **Eating occasion** | **Protein target** |
| --- | --- |
| Breakfast | 15–20 g |
| Lunch | 20–25 g |
| Dinner | 20–25 g |
| Snacks, including bedtime snack | 15–20 g |
| **Total** | **70–85 g/day** |

Protein should be spread throughout the day rather than eaten in one large meal.

**3. Meal frequency and eating pattern**

Recommend:

**3 main meals + 2–3 snacks daily**

Include a **late-evening snack every night** to reduce overnight muscle breakdown.

Suggested daily pattern:

| **Time** | **Example** |
| --- | --- |
| Breakfast | Oats or rice porridge + egg + low-fat milk/soy milk |
| Mid-morning snack | Yogurt, soy milk, or fruit with a small amount of unsalted nuts |
| Lunch | Rice/noodles/sweet potato + tofu/fish/chicken + vegetables |
| Afternoon snack | Yogurt, tofu pudding, milk, or whole-grain crackers |
| Dinner | Smaller portion of staple food + protein dish + vegetables |
| Late-evening snack | Milk/soy milk/yogurt + oats, bread, crackers, or sweet potato |

Good bedtime snack options:

- Low-fat milk + whole-grain bread
- Unsweetened soy milk + oatmeal
- Yogurt + banana
- Tofu pudding + small steamed bun
- Low-salt crackers + milk

The bedtime snack should contain **carbohydrate plus protein**, not just tea or fruit.

**4. Specific food recommendations and restrictions**

**Recommended foods**

**Protein sources**

Prefer:

- Tofu, soy milk, edamame
- Beans, lentils, chickpeas, if tolerated
- Low-fat milk, yogurt, kefir
- Eggs
- Fish
- Skinless poultry
- Small portions of lean meat if tolerated

For patients with prior HE, **plant and dairy proteins** are often better tolerated than large meat-heavy meals.

**Carbohydrates**

Choose moderate portions of complex carbohydrates:

- Oats
- Rice, noodles, potatoes, sweet potatoes
- Whole grains if tolerated
- Corn
- Beans and lentils

Avoid very-low-carbohydrate or ketogenic diets because they can worsen catabolism in cirrhosis.

**Fats**

Use modest amounts of healthier fats:

- Canola oil or olive oil
- Unsalted nuts and seeds
- Fish

Limit:

- Fried foods
- Fatty meat
- Butter, cream, lard
- Pastries and ultra-processed snacks

**Vegetables and fruits**

- Vegetables daily, preferably cooked if bloating is present.
- Fruit in moderate portions.
- Potassium restriction is only needed if hyperkalemia or significant kidney disease is present.

**Restrictions**

**Alcohol**

- **Absolute abstinence** is essential.
- Refer for alcohol cessation support if ongoing cravings or relapse risk are present.

**Sodium**

If ascites, edema, or hypertension is present:

**≤2 g sodium/day, approximately ≤5 g salt/day**

Avoid or limit:

- Pickles and preserved vegetables
- Salted fish
- Sausage, bacon, cured meats
- Instant noodles
- Hotpot soup base
- Soy sauce-heavy dishes
- Canned soups
- Processed snacks

**Food safety**

Avoid:

- Raw seafood, especially raw shellfish
- Undercooked meat
- Undercooked eggs
- Unpasteurized dairy
- Foods stored unrefrigerated for long periods

**Supplements and herbs**

Avoid unsupervised:

- “Liver detox” products
- Herbal remedies with unclear ingredients
- High-dose vitamin A
- High-dose niacin
- Iron supplements unless prescribed
- Weight-loss pills or bodybuilding supplements

**5. Additional nutritional considerations**

**Energy intake**

Because she has obesity but cirrhosis with HE history, avoid both overfeeding and aggressive dieting.

Use **ideal or adjusted body weight** for planning. A practical target is:

**~1,600–1,900 kcal/day**

Adjust based on dry weight, ascites, appetite, glucose control, muscle mass, and weight trend. Weight loss, if needed, should be **slow and supervised**, with adequate protein and resistance exercise to avoid muscle loss.

**Hepatic encephalopathy prevention**

- Maintain regular bowel movements.
- Avoid constipation.
- Continue lactulose and/or rifaximin if prescribed.
- Avoid large meat-heavy meals.
- Avoid skipped meals and overnight fasting.
- Seek medical care if confusion, reversed sleep rhythm, personality change, drowsiness, or hand flapping recurs.

If protein intake appears poorly tolerated:

- Shift more protein to **plant and dairy sources**.
- Use smaller protein portions more frequently.
- Consider oral nutrition supplements or **BCAA-enriched supplements** under clinician/dietitian supervision.

**Alcohol-related cirrhosis considerations**

Assess and supplement deficiencies common with alcohol-related liver disease:

- **Thiamine, especially if recent alcohol use**
- Folate
- Vitamin B6 and B12
- Vitamin D and calcium
- Magnesium, phosphate, and zinc

Thiamine should be given before glucose-containing IV fluids if acute alcohol-related malnutrition or Wernicke risk is suspected.

**Muscle and activity**

Even with obesity, check for sarcopenia:

- Unintentional muscle loss
- Weak grip
- Slow walking speed
- Low physical activity

If medically stable, recommend:

- Regular walking
- Light resistance exercise
- Avoid prolonged bed rest

**Practical daily prescription**

For this patient:

- **Energy:** about **1,600–1,900 kcal/day**, individualized to dry weight and muscle status
- **Protein:** **70–85 g/day**, up to **90 g/day** if sarcopenic or poor intake
- **Meal pattern:** **3 meals + 2–3 snacks/day**
- **Bedtime snack:** every night
- **Preferred protein:** tofu/soy, dairy, eggs, fish, poultry
- **Sodium:** **≤2 g/day if ascites/edema**
- **Alcohol:** **0 intake**
- **Main priority:** alcohol abstinence, HE prevention, and muscle preservation without crash dieting.

# C3-09

**Dietary and nutritional recommendations for C3-09**

**Patient:** Male, 64 years old, 167 cm, 57 kg
**Diagnosis:** MASLD cirrhosis, recovering from hepatic encephalopathy, low BMI / nutritional risk
**BMI:** ~20.4 kg/m². This is not frankly underweight by general BMI criteria, but in cirrhosis, especially in an older male with HE recovery, it suggests **high risk of sarcopenia and malnutrition**.

Main goals: **restore nutritional status, preserve or rebuild muscle, prevent recurrent hepatic encephalopathy, and avoid further weight loss**.

**1. Overall dietary principles**

Recommend an **adequate-to-high energy, high-protein, low-sodium, Mediterranean-style diet**, with frequent meals and a mandatory bedtime snack.

Key principles:

- **Do not restrict protein**, even during recovery from hepatic encephalopathy.
- Avoid fasting, skipped meals, and very-low-carbohydrate dieting.
- Prioritize **plant and dairy proteins**, with fish, eggs, and poultry as additional sources.
- Increase total calories because of low BMI and high malnutrition risk.
- Control MASLD-related metabolic risks, but **do not aim for weight loss now**.
- Restrict sodium if ascites, edema, or hypertension is present.
- Avoid alcohol completely.

**2. Recommended daily protein intake**

For cirrhosis with HE recovery and low BMI, recommend:

**1.2–1.5 g/kg/day protein**

Using current body weight:

**57 kg × 1.2–1.5 g/kg/day = ~68–86 g protein/day**

Because he has low BMI/nutritional risk, a practical target is:

**75–85 g protein/day**

If sarcopenia, poor appetite, or continued weight loss is present, aim toward **85–90 g/day**, using oral nutrition supplements if needed.

Suggested distribution:

| **Eating occasion** | **Protein target** |
| --- | --- |
| Breakfast | 15–20 g |
| Lunch | 20–25 g |
| Dinner | 20–25 g |
| Snacks, including bedtime snack | 15–20 g |
| **Total** | **75–85 g/day** |

Protein should be divided across the day rather than concentrated in one large meat-heavy meal.

**3. Meal frequency and eating pattern**

Recommend:

**3 main meals + 3 snacks daily**

He should avoid fasting longer than **4–6 hours during the day** and should take a **late-evening snack every night**.

Suggested daily pattern:

| **Time** | **Example** |
| --- | --- |
| Breakfast | Oats or rice porridge + egg + milk/soy milk |
| Mid-morning snack | Yogurt or fortified soy milk + fruit |
| Lunch | Rice/noodles/potato + tofu/fish/chicken + cooked vegetables |
| Afternoon snack | Milk, yogurt, tofu pudding, or unsalted nuts |
| Dinner | Staple food + protein dish + vegetables |
| Late-evening snack | Milk/soy milk/yogurt + bread, oats, crackers, or sweet potato |

Good bedtime snack options:

- Milk + whole-grain bread
- Soy milk + oatmeal
- Yogurt + banana
- Tofu pudding + steamed bun
- Oral nutrition supplement
- Low-salt crackers + milk

The bedtime snack should contain **carbohydrate plus protein**, not just fruit, tea, or water.

**4. Specific food recommendations and restrictions**

**Recommended foods**

**Protein sources**

Prioritize well-tolerated, high-quality proteins:

- Tofu, soy milk, edamame
- Beans, lentils, chickpeas, if tolerated
- Milk, yogurt, kefir
- Eggs
- Fish
- Skinless poultry
- Small portions of lean meat if tolerated

For HE recovery, **plant and dairy proteins** are often better tolerated than large meat-heavy meals.

**Energy-dense nutritious foods**

Because of low BMI/nutritional risk, add calories without relying on sugar:

- Add milk or soy milk to oats/porridge
- Add olive or canola oil to cooked vegetables
- Use nut butter or unsalted nuts if tolerated
- Add avocado if available
- Use yogurt, tofu pudding, eggs, and fish as snacks
- Consider oral nutrition supplements between meals

**Carbohydrates**

Include adequate carbohydrates to reduce muscle breakdown:

- Rice, noodles, oats
- Potatoes, sweet potatoes
- Bread or steamed buns
- Corn
- Whole grains if tolerated

Avoid ketogenic or very-low-carbohydrate diets.

**Vegetables and fruits**

- Include vegetables daily, cooked if bloating occurs.
- Include fruit in moderate portions.
- Do not restrict potassium unless hyperkalemia, renal disease, or clinician-directed restriction is present.

**Fats**

Use moderate healthy fats:

- Olive oil or canola oil
- Unsalted nuts and seeds
- Fish

**Restrictions**

**Alcohol**

- Complete avoidance.

**Sodium**

If ascites, edema, or hypertension is present:

**≤2 g sodium/day, approximately ≤5 g salt/day**

Avoid or limit:

- Pickles, preserved vegetables, salted fish
- Sausage, bacon, cured meats
- Instant noodles
- Hotpot soup base
- Soy sauce-heavy dishes
- Canned soups
- Processed snacks

**Food safety**

Avoid:

- Raw seafood, especially shellfish
- Undercooked meat
- Undercooked eggs
- Unpasteurized dairy
- Foods left unrefrigerated for long periods

**Supplements and herbs**

Avoid unsupervised:

- “Liver detox” products
- Herbal remedies with unclear ingredients
- High-dose vitamin A
- High-dose niacin
- Iron supplements unless prescribed
- Weight-loss products, especially inappropriate for this patient

**5. Additional nutritional considerations**

**Energy intake**

For cirrhosis with low BMI/nutritional risk, recommend:

**35 kcal/kg/day, sometimes higher if weight loss continues**

Using 57 kg:

**57 × 35 = ~2,000 kcal/day**

Practical target:

**~2,000–2,200 kcal/day**

Adjust according to dry weight, appetite, ascites, glucose control, and weekly weight trend.

**Hepatic encephalopathy recovery**

Because he is recovering from HE:

- Maintain regular bowel movements.
- Avoid constipation.
- Continue lactulose and/or rifaximin if prescribed.
- Avoid large meat-heavy meals.
- Avoid sedatives and alcohol unless specifically approved.
- Seek medical care if confusion, sleep reversal, drowsiness, personality change, or hand flapping recurs.

If adequate protein is difficult or HE symptoms recur:

- Shift more protein to **vegetable and dairy sources**.
- Use smaller, more frequent protein portions.
- Consider **BCAA-enriched oral nutrition supplements** under clinician/dietitian supervision.

**MASLD-specific considerations**

Because this is MASLD cirrhosis but he has low BMI/nutritional risk:

- **Do not prescribe weight loss.**
- Avoid sugar-sweetened beverages and frequent desserts.
- Use Mediterranean-style foods: vegetables, legumes, whole grains as tolerated, fish, olive/canola oil, nuts.
- Screen and manage diabetes, dyslipidemia, and hypertension.
- Preserve muscle as the priority.

**Micronutrients**

Assess and replace deficiencies, especially:

- Vitamin D and calcium
- Zinc, particularly with HE history, poor appetite, taste changes, or diarrhea from lactulose
- Folate and B vitamins
- Magnesium and phosphate if intake is poor
- Fat-soluble vitamins if cholestasis is present

**Physical activity**

If medically stable:

- Encourage walking after meals as tolerated.
- Add light resistance training to preserve muscle.
- Avoid prolonged bed rest.
- Combine exercise with adequate protein intake.

**Practical daily prescription**

For this patient:

- **Energy:** about **2,000–2,200 kcal/day**
- **Protein:** **75–85 g/day**, up to **90 g/day** if sarcopenic or losing weight
- **Meal pattern:** **3 meals + 3 snacks/day**
- **Bedtime snack:** every night
- **Preferred protein:** tofu/soy, dairy, eggs, fish, poultry
- **Sodium:** **≤2 g/day if ascites/edema**
- **Alcohol:** **0 intake**
- **Weight goal:** stop further weight loss and rebuild muscle, not weight reduction.

# C3-10

For this **68-year-old woman, 159 cm/50 kg, HBV cirrhosis, prior hepatic encephalopathy (HE), poor appetite**, the key goal is to **prevent muscle loss and fasting-related catabolism while avoiding HE triggers**. Her BMI is about **19.8 kg/m²**, so she is at nutritional risk.

**1. Overall dietary principles**

Aim for a **high-energy, adequate-to-high protein diet** rather than protein restriction. Modern cirrhosis guidance recommends **not restricting protein even in patients with hepatic encephalopathy**, because inadequate protein worsens sarcopenia and may worsen outcomes.

Estimated daily targets:

- **Energy:** about **30–35 kcal/kg/day** → **1,500–1,750 kcal/day**
- **Protein:** **1.2–1.5 g/kg/day** → **60–75 g protein/day**

Because she has poor appetite, prioritize **nutrient-dense, small portions** rather than large meals.

**2. Recommended daily protein intake**

Recommend **60–75 g protein/day**, distributed across the day.

Practical protein distribution:

- Breakfast: **15–20 g**
- Lunch: **15–20 g**
- Dinner: **15–20 g**
- Snacks, including bedtime snack: **10–20 g total**

Good protein choices for prior HE:

- **Soy products:** tofu, soy milk, edamame
- **Eggs**
- **Fish and poultry**
- **Milk, yogurt, cheese if tolerated**
- **Beans/lentils**, if not causing excessive bloating
- **Oral nutrition supplements** if meals are insufficient

Vegetable and dairy proteins may be better tolerated in HE than large portions of red meat; this does **not** mean she must be vegetarian, but plant/dairy protein can be emphasized.

**3. Meal frequency and eating pattern**

She should avoid long fasting periods. Patients with cirrhosis and HE are advised to take **small, frequent meals** and include a **late-evening snack** to shorten overnight fasting.

Suggested pattern:

- **3 small meals + 2–3 snacks daily**
- Eat every **3–4 hours**
- Add a **bedtime snack** every night
- Avoid skipping breakfast

Examples of bedtime snacks:

- Warm milk or soy milk + oatmeal
- Yogurt + banana
- Tofu pudding or soy milk + whole-grain bread
- Egg custard
- Nutrition supplement drink
- Congee with egg/tofu/fish flakes

A good bedtime snack should contain **carbohydrate plus protein**, not just fruit or tea.

**4. Specific food recommendations and restrictions**

**Encourage**

**Staple carbohydrates:**
Rice, noodles, oats, potatoes, sweet potatoes, whole-grain bread, congee. Carbohydrates are useful in cirrhosis because they reduce fasting-related muscle breakdown.

**Protein foods:**
Tofu, soy milk, fish, chicken, eggs, yogurt, milk, beans, lentils.

**Vegetables and fruit:**
Daily intake is encouraged for fiber, vitamins, and constipation prevention. Constipation can precipitate HE.

**Healthy fats:**
Use moderate amounts of vegetable oils, nuts, sesame paste, avocado, or nut butter to increase calories if appetite is poor.

**Limit or avoid**

**Alcohol:** completely avoid.

**Large red-meat-heavy meals:** not strictly forbidden, but avoid large portions, especially if she notices sleepiness/confusion afterward.

**Very salty foods**, especially if ascites or leg edema is present: salted fish, pickles, preserved vegetables, cured meats, instant noodles, salty sauces, processed foods. If she has ascites/edema, a typical target is **sodium <2 g/day**, roughly equivalent to **salt <5 g/day**.

**Raw or unsafe foods:** avoid raw seafood, undercooked eggs/meat, and unpasteurized foods because cirrhosis increases infection risk.

**5. Additional nutritional considerations**

Because she has **poor appetite**, consider:

- Add **oral nutrition supplements** between meals, not replacing meals.
- Fortify foods: add egg, tofu, milk powder, peanut/sesame paste, or oil to congee/noodles.
- Choose soft, moist foods if chewing or early satiety is an issue.
- Monitor body weight, grip strength, walking ability, and appetite regularly.

For prior HE:

- **Do not self-restrict protein.**
- Maintain regular bowel movements; constipation can worsen HE.
- Continue prescribed HE therapy such as lactulose/rifaximin if ordered by her clinician.
- Seek medical review if new sleepiness, confusion, personality change, asterixis, poor intake, vomiting, infection, GI bleeding, or constipation occurs.

Also consider screening or supplementation for common cirrhosis-related deficiencies: **vitamin D, calcium, thiamine if intake is poor, zinc if deficiency or recurrent HE is suspected**, and bone health assessment. These should ideally be individualized based on labs and clinician review.

**Simple daily target for her:**
**1,500–1,750 kcal/day + 60–75 g protein/day**, split into **5–6 eating occasions**, with a **protein-containing bedtime snack every night**.

# C3-11

For this **72-year-old man, 170 cm/74 kg, alcohol-related cirrhosis with recurrent hepatic encephalopathy**, the goal is to **prevent sarcopenia, reduce HE recurrence triggers, and maintain adequate intake without protein restriction**.

His BMI is about **25.6 kg/m²**. Use **dry weight** if he has ascites or edema; if 74 kg is close to dry weight, the targets below are appropriate.

**1. Overall dietary principles**

He should follow a **high-energy, adequate-to-high protein, alcohol-free diet**.

Daily targets:

- **Energy:** about **30–35 kcal/kg/day**
  → approximately **2,200–2,600 kcal/day**
- **Protein:** **1.2–1.5 g/kg/day**
  → approximately **90–110 g protein/day**

Protein should **not be routinely restricted** in hepatic encephalopathy. EASL guidance notes that normal-to-high protein intake does not precipitate HE and may improve mental status, while inadequate protein worsens muscle loss; recommended protein intake in cirrhosis is **1.2–1.5 g/kg/day**.

**2. Recommended daily protein intake**

Recommend **90–110 g protein/day**, divided across meals and snacks.

A practical distribution:

| **Time** | **Protein goal** |
| --- | --- |
| Breakfast | 20–25 g |
| Lunch | 25–30 g |
| Dinner | 25–30 g |
| Snacks/bedtime snack | 20–30 g total |

Because he has **recurrent HE**, emphasize **vegetable and dairy protein** and avoid very large red-meat-heavy meals. ESPEN guidance supports vegetable protein or branched-chain amino acids in patients who are “protein intolerant,” and recommends late-evening or nocturnal supplementation when oral intake is inadequate.

Good protein choices:

- **Tofu, soy milk, edamame, bean curd**
- **Eggs**
- **Fish, chicken, lean pork in moderate portions**
- **Milk, yogurt, cheese if tolerated**
- **Beans/lentils**, if they do not cause troublesome bloating
- **Oral nutrition supplement** if meals are insufficient

**3. Meal frequency and eating pattern**

He should avoid prolonged fasting.

Recommended pattern:

- **3 meals + 2–3 snacks daily**
- Eat every **3–4 hours**
- **Mandatory late-evening snack** before sleep
- Avoid skipping breakfast
- Avoid long overnight fasting greater than about **10–12 hours**

ESPEN recommends **three to five meals per day plus a late-evening snack** to improve whole-body protein status in cirrhosis.

Examples of bedtime snacks:

- Soy milk + whole-grain bread
- Yogurt + banana or oats
- Milk + crackers
- Egg custard
- Tofu pudding
- Nutrition supplement drink
- Congee with egg, tofu, or fish

The bedtime snack should contain **carbohydrate plus protein**, not just tea, fruit, or plain rice porridge.

**4. Specific food recommendations and restrictions**

**Strongly recommend**

**Complete alcohol abstinence**
No beer, wine, liquor, “small amounts,” or medicinal alcohol preparations. In alcohol-related cirrhosis, continued alcohol use markedly increases risk of decompensation, HE recurrence, hospitalization, and mortality.

**Adequate carbohydrate intake**
Rice, noodles, oats, potatoes, sweet potatoes, bread, and congee help reduce fasting-related muscle breakdown.

**High-quality protein at every meal**
Tofu, eggs, fish, poultry, dairy, legumes, and oral nutrition supplements as needed.

**Vegetables and fruit daily**
Fiber helps prevent constipation, which is an important HE trigger.

**Safe food hygiene**
Avoid raw seafood, undercooked meat/eggs, unpasteurized dairy, and spoiled foods because cirrhosis increases infection risk.

**Limit or avoid**

**Large red meat portions**
Not absolutely forbidden, but avoid large servings, especially dinner-heavy meat meals. Use more fish, poultry, dairy, soy, and legumes.

**Excess salt**
If he has ascites or leg edema, target approximately:

- **Sodium <2 g/day**, equivalent to about **salt <5 g/day**

Avoid salted fish, pickles, cured meats, instant noodles, salty sauces, processed meats, canned soups, and heavily seasoned restaurant foods. Sodium restriction should not make food so unpalatable that intake falls; ESPEN cautions that low-sodium diets can worsen poor intake if overly restrictive.

**Constipation-promoting pattern**
Low-fiber intake, dehydration, and skipped lactulose can precipitate HE.

**5. Additional nutritional considerations**

Because this is **alcohol cirrhosis with recurrent HE**, also address:

**HE prevention through nutrition**

- Do **not** self-restrict protein.
- Maintain regular bowel movements, typically **2–3 soft stools/day** if on lactulose, according to clinician instruction.
- Ensure enough fluids unless restricted for hyponatremia or ascites.
- Treat constipation, infection, GI bleeding, dehydration, and sedative use as potential HE triggers.

**Micronutrients**

Alcohol-related cirrhosis commonly carries vitamin and mineral deficiencies. Discuss checking or supplementing:

- **Thiamine/vitamin B1**, especially important in alcohol-related disease
- **Folate and other B vitamins**
- **Vitamin D and calcium** for bone health
- **Zinc**, particularly if deficiency, poor taste, poor appetite, or recurrent HE is present
- **Magnesium and phosphate** if malnourished or recently abstinent

**Branched-chain amino acids**

If he cannot meet protein goals, has recurrent HE, or appears “protein intolerant,” consider **BCAA-enriched oral supplements**, especially as a late-evening snack. ESPEN notes long-term oral BCAA supplementation may improve event-free survival or quality of life in advanced cirrhosis.

**Exercise with nutrition**

If medically safe, combine nutrition with **resistance exercise and walking** to preserve muscle. Muscle mass helps metabolize ammonia and may reduce HE risk.

**Practical daily plan**

A simple day could look like:

- **Breakfast:** oatmeal or congee + egg + soy milk
- **Snack:** yogurt or nutrition supplement
- **Lunch:** rice/noodles + fish/chicken/tofu + vegetables
- **Snack:** fruit + milk/soy milk or crackers with cheese
- **Dinner:** rice/potato + tofu/fish/egg + vegetables
- **Bedtime snack:** soy milk/yogurt/nutrition supplement + bread/oats/crackers

**Bottom line:** target about **2,200–2,600 kcal/day** and **90–110 g protein/day**, split into **5–6 eating occasions**, with a **protein-containing late-evening snack every night**, **no alcohol**, **no unnecessary protein restriction**, and sodium restriction if ascites or edema is present.

# C3-12

For this **75-year-old woman, 155 cm/62 kg, MASLD cirrhosis with prior hepatic encephalopathy**, the goal is to **preserve muscle, avoid prolonged fasting, prevent recurrent HE, and manage metabolic risk** without causing undernutrition.

Her BMI is about **25.8 kg/m²**. In cirrhosis, BMI can be misleading if there is ascites or edema, so use **dry weight** for calculations when possible.

**1. Overall dietary principles**

She should follow a **high-protein, adequate-energy, Mediterranean-style diet** with careful meal timing.

Estimated daily targets, using 62 kg:

- **Energy:** about **25–30 kcal/kg/day** if weight is stable or overweight
  → approximately **1,550–1,850 kcal/day**
- If she is frail, losing weight, sarcopenic, or eating poorly:
  **30–35 kcal/kg/day** → approximately **1,850–2,170 kcal/day**
- **Protein:** **1.2–1.5 g/kg/day**
  → approximately **75–95 g protein/day**

Protein should **not be restricted** because of prior hepatic encephalopathy. EASL and ESPEN guidance recommend **1.2–1.5 g/kg/day protein** in cirrhosis and advise frequent meals plus a late-evening snack to reduce fasting-related muscle breakdown.

**2. Recommended daily protein intake**

Recommend **75–95 g protein/day**, divided throughout the day.

A practical distribution:

| **Time** | **Protein goal** |
| --- | --- |
| Breakfast | 20 g |
| Lunch | 25 g |
| Dinner | 25 g |
| Snacks/bedtime snack | 15–25 g total |

For a patient with prior HE, emphasize **vegetable and dairy protein**, which may be better tolerated than large red-meat-heavy meals. AASLD materials also emphasize that protein restriction in HE is not recommended because malnutrition and sarcopenia are common and clinically important in cirrhosis.

Good choices:

- **Tofu, soy milk, edamame, beans, lentils**
- **Eggs**
- **Fish and skinless poultry**
- **Low-sugar yogurt, milk, cheese if tolerated**
- **Small portions of lean meat if desired**
- **Oral nutrition supplements** if appetite or intake is poor

**3. Meal frequency and eating pattern**

She should avoid long fasting periods.

Recommended pattern:

- **3 small-to-moderate meals + 2–3 snacks daily**
- Eat every **3–4 hours**
- Include a **late-evening snack every night**
- Avoid skipping breakfast
- Avoid very large dinners

Late-evening snacks are recommended in cirrhosis to shorten overnight fasting and support protein balance.

Good bedtime snack options:

- Unsweetened soy milk + whole-grain bread
- Low-sugar yogurt + oats
- Milk + crackers
- Egg custard
- Tofu pudding without excess sugar
- Small bowl of oatmeal with milk
- Nutrition supplement drink, preferably diabetes-friendly if hyperglycemia is present

The bedtime snack should contain **carbohydrate plus protein**, not only fruit or tea.

**4. Specific food recommendations and restrictions**

**Encourage**

**Mediterranean-style MASLD pattern**

- Vegetables daily
- Beans, lentils, soy products
- Fish, especially oily fish if tolerated
- Poultry, eggs, low-fat or moderate-fat dairy
- Whole grains: oats, brown rice, whole-grain bread, buckwheat, corn, sweet potato
- Nuts or seeds in small portions
- Olive oil or other unsaturated vegetable oils

**Adequate fiber**

Fiber helps metabolic health and helps prevent constipation, an important HE trigger. Increase fiber gradually if bloating occurs.

**Protein at every meal**

Do not rely on plain porridge, noodles, or rice alone. Add egg, tofu, fish, poultry, milk, yogurt, or soy milk.

**Limit or avoid**

**Alcohol:** completely avoid.

**Sugary foods and drinks:** avoid sugar-sweetened beverages, sweetened tea, desserts, candy, sweet pastries, and large amounts of fruit juice. This is especially important in MASLD and if diabetes or insulin resistance is present.

**Refined carbohydrates:** limit white bread, sweet buns, biscuits, and large portions of refined noodles or rice. She does not need a very low-carbohydrate diet, but carbohydrate portions should be controlled and paired with protein.

**Large red-meat-heavy meals:** not absolutely forbidden, but avoid frequent or large portions, especially at dinner, because prior HE patients often tolerate plant/dairy/fish protein better.

**Excess salt:** if ascites, edema, or hypertension is present, target about **sodium <2 g/day**, equivalent to **salt <5 g/day**. Limit pickles, salted fish, cured meats, instant noodles, processed foods, canned soups, and salty sauces.

**Raw or unsafe foods:** avoid raw seafood, undercooked meat/eggs, and unpasteurized dairy due to higher infection risk in cirrhosis.

**5. Additional nutritional considerations**

**Weight and muscle**

Although her BMI is in the overweight range, she should **not follow a crash diet**. In MASLD cirrhosis, the priority is **fat reduction without muscle loss**. If weight loss is needed, aim for slow loss, usually about **0.25–0.5 kg/week**, while maintaining **75–95 g protein/day** and doing safe activity.

**Exercise**

If medically safe:

- Walk most days
- Add light resistance training 2–3 times weekly
- Avoid prolonged bed rest

Muscle helps ammonia metabolism, so preserving muscle is important for HE prevention.

**HE prevention**

- Do **not** self-restrict protein.
- Prevent constipation; if prescribed lactulose, follow the clinician’s stool target, commonly **2–3 soft stools/day**.
- Maintain hydration unless fluid restriction is prescribed.
- Seek medical review for new confusion, sleep reversal, drowsiness, falls, constipation, infection, GI bleeding, vomiting, or poor intake.

**Micronutrients**

Consider checking and supplementing as appropriate:

- **Vitamin D and calcium** for bone health
- **Zinc**, especially with recurrent HE, poor taste, or deficiency
- **B vitamins**, especially if intake is poor
- **Sarcopenia/frailty assessment**, such as grip strength, gait speed, or muscle mass evaluation

**If intake is inadequate**

Use **oral nutrition supplements** between meals or as a bedtime snack. If blood glucose is high, choose a **diabetes-friendly, high-protein formula**.

**Practical daily plan**

Example day:

- **Breakfast:** oatmeal with milk/soy milk + boiled egg
- **Snack:** low-sugar yogurt or soy milk
- **Lunch:** rice or whole grains + fish/chicken/tofu + vegetables
- **Snack:** fruit plus nuts, or milk/soy milk
- **Dinner:** tofu/egg/fish + vegetables + controlled portion of staple carbohydrate
- **Bedtime snack:** soy milk/yogurt/milk + oats, crackers, or whole-grain bread

**Bottom line:** target about **75–95 g protein/day**, avoid protein restriction despite prior HE, eat **5–6 times daily**, include a **protein-containing late-evening snack**, follow a **Mediterranean-style, low-sugar, moderate-carbohydrate diet**, and restrict sodium if ascites or edema is present.

窗体顶端

窗体底端
