# Supplementary material for: Guideline adherence and expert-rated potential safety concerns in AI-generated dietary advice for patients with cirrhosis: a comparative evaluation of large language models
Source: Front Nutr. 2026 Oct 8;13:1893597. doi: 10.3389/fnut.2026.1893597 (PMC13689687; doi:10.3389/fnut.2026.1893597)
Supplement: Supplementary file 1 [file Supplementary_file_1.zip › Supplementary Data S1 (Verbatim Model Responses).ZIP!/answer via Claude.docx]

## C101

**Dietary & Nutritional Recommendations**

**Patient:** Male, 34 y/o | HBV-related Compensated Cirrhosis | BMI: 23.0 kg/m² (Normal)

**1. Overall Dietary Principles**

This patient has **compensated cirrhosis with preserved hepatic synthetic function** and no current complications. The primary nutritional goals are:

- **Prevent malnutrition** — a common and underappreciated complication even in compensated cirrhosis
- **Support hepatic regeneration** and slow disease progression
- **Prevent muscle wasting (sarcopenia)**, which worsens prognosis
- **Avoid dietary triggers** that could precipitate decompensation
- Maintain a **well-balanced, energy-adequate diet** with no unnecessary restrictions

Blanket "low-protein" diets are **not recommended** in compensated cirrhosis and are actively harmful. This outdated practice should be avoided.

**2. Recommended Daily Protein Intake**

| **Parameter** | **Target** |
| --- | --- |
| **Daily protein** | **1.2 – 1.5 g/kg ideal body weight/day** |
| **Calculated target (68 kg)** | **~82 – 102 g protein/day** |
| **Protein source preference** | Mixed: plant-based + lean animal protein |
| **BCAA-enriched foods** | Encouraged (dairy, eggs, legumes) |

- **Branched-chain amino acids (BCAAs)** — leucine, isoleucine, valine — are particularly beneficial in cirrhosis due to impaired hepatic amino acid metabolism
- **Plant-based proteins** (legumes, soy, tofu) are preferable as they generate **less ammonia** than red meat
- **Dairy proteins** (whey, casein) are excellent and well-tolerated
- No protein restriction is needed given the **absence of hepatic encephalopathy**

**3. Meal Frequency and Eating Pattern**

A **modified meal timing strategy** is critical in cirrhosis due to accelerated starvation metabolism (cirrhotic patients shift to fat oxidation after only 2–3 hours of fasting, vs. ~12–16 hours in healthy individuals).

**Recommended Schedule:**

Breakfast	07:00 – 08:00	← Do NOT skip

Morning Snack	10:00 – 10:30

Lunch	12:00 – 13:00

Afternoon Snack 15:00 – 16:00

Dinner	18:00 – 19:00

Late-Evening Snack 21:00 – 22:00	← MOST IMPORTANT

**Late-Evening Snack (LES) — Priority Recommendation**

- Consume a **carbohydrate + protein snack** within **2–3 hours before sleep**
- This is the single most evidence-based dietary intervention in cirrhosis
- **Examples:** Whole grain toast + peanut butter, a glass of milk + crackers, Greek yogurt with fruit, or a BCAA supplement drink
- Rationale: Minimizes the prolonged overnight fasting state that accelerates gluconeogenesis and muscle catabolism

**4. Specific Food Recommendations and Restrictions**

**Encouraged Foods**

| **Category** | **Examples** |
| --- | --- |
| **High-quality protein** | Eggs, Greek yogurt, low-fat dairy, tofu, fish, skinless poultry |
| **Plant proteins** | Lentils, chickpeas, edamame, black beans |
| **Complex carbohydrates** | Oats, brown rice, whole wheat bread, sweet potatoes |
| **Healthy fats** | Olive oil, avocado, nuts, fatty fish (salmon, mackerel) |
| **BCAA-rich foods** | Whey protein, tuna, salmon, pumpkin seeds, edamame |
| **Fruits & vegetables** | All varieties; emphasize variety and color |
| **Coffee** | 2–3 cups/day associated with slower fibrosis progression |

**Foods to Restrict or Avoid**

| **Item** | **Reason** |
| --- | --- |
| **Alcohol (complete abstinence)** | Even low-level intake accelerates HBV-related fibrosis progression; absolute contraindication |
| **Raw shellfish/oysters** | Risk of *Vibrio vulnificus* infection, which is rapidly fatal in cirrhosis |
| **Raw or undercooked meats/eggs** | Infection risk due to impaired immune defense |
| **Highly processed/ultra-processed foods** | Pro-inflammatory, hepatotoxic additives |
| **High-fructose corn syrup, sugary beverages** | Promote hepatic steatosis and inflammation |
| **Red and processed meats** | High ammonia load; limit to <2 servings/week |
| **Excessive sodium** | Even in compensated disease, limit to **<2,000 mg/day** to reduce risk of ascites development |
| **Herbal supplements/traditional remedies** | Many are hepatotoxic (e.g., kava, comfrey, certain TCM herbs); consult physician before use |

**5. Additional Nutritional Considerations**

**Micronutrient Monitoring & Supplementation**

| **Nutrient** | **Relevance in This Patient** | **Action** |
| --- | --- | --- |
| **Vitamin D** | Frequently deficient in HBV cirrhosis; impacts bone health and immune regulation | Check 25-OH Vitamin D; supplement if <30 ng/mL |
| **Zinc** | Deficiency impairs ammonia metabolism, taste, and immune function; common in cirrhosis | Consider supplementation 25–50 mg/day if deficient |
| **Vitamin B complex (esp. B1, B6, B12, Folate)** | Impaired hepatic storage and metabolism | Supplement with B-complex if dietary intake inadequate |
| **Magnesium** | Often depleted; important for muscle function | Monitor serum levels |
| **Iron** | Avoid supplementation unless confirmed deficient — iron overload worsens HBV-related fibrosis | Check ferritin/TSAT before supplementing |
| **Fat-soluble vitamins (A, E, K)** | Absorption may be impaired if cholestasis present | Assess clinically; supplement only if deficient |

**Sarcopenia Prevention**

- Given his age and active lifestyle potential, **resistance exercise** 3–4×/week should be encouraged alongside adequate protein intake
- Low skeletal muscle mass is an **independent predictor of mortality** in cirrhosis
- Target: maintain or increase muscle mass, not just weight

**HBV-Specific Considerations**

- Confirm patient is on **antiviral therapy** (TDF or ETV) — nutrition cannot replace antiviral suppression
- Avoid **acetaminophen overuse** (safe at ≤2 g/day total) and all NSAIDs
- Avoid hepatotoxic supplements: high-dose Vitamin A, green tea extract, kava, chaparral

**Monitoring Plan**

- Reassess nutritional status every **3–6 months** using:
  - Hand grip strength (sarcopenia screening)
  - Mid-arm muscle circumference or DEXA scan
  - Serum albumin, prealbumin, total protein
  - Body weight trend
- Use validated screening tool: **Royal Free Hospital Global Assessment (RFH-GA)** or **Nutritional Risk Screening (NRS-2002)**

**Daily Intake Summary**

|  | **Target** |
| --- | --- |
| **Total calories** | ~2,000–2,200 kcal/day (~30–35 kcal/kg/day) |
| **Protein** | 82–102 g/day (1.2–1.5 g/kg/day) |
| **Carbohydrates** | 50–55% of total calories; complex sources preferred |
| **Fat** | 25–30% of total calories; unsaturated fats preferred |
| **Sodium** | <2,000 mg/day |
| **Fluid** | No restriction needed currently (~2 L/day) |
| **Alcohol** | Zero — absolute abstinence |
| **Meal frequency** | 5–6 times/day including mandatory late-evening snack |

**Clinical Note:** This patient is currently in a favorable nutritional and clinical position. Proactive nutritional optimization at this compensated stage is strongly associated with reduced risk of decompensation, hospitalization, and transplant listing. Early dietitian involvement and regular follow-up are strongly recommended.

## C102

**Dietary & Nutritional Recommendations**

**Patient C1-02** | Female, 39 y/o | 160 cm, 54 kg (BMI 21.1 kg/m²) **Diagnosis:** MASLD-related Compensated Cirrhosis

**1. Overall Dietary Principles**

This patient has **normal BMI** with compensated (Child-Pugh A) MASLD-cirrhosis. Since MASLD is the underlying etiology, dietary strategy must simultaneously:

- **Support hepatic regeneration and prevent decompensation**
- **Address the metabolic drivers** of MASLD (insulin resistance, dyslipidemia)
- **Prevent malnutrition** — a common and underrecognized risk even in compensated cirrhosis

**Key caveat:** Despite a normal BMI, cirrhotic patients frequently have *sarcopenic obesity* (reduced muscle mass with preserved or increased fat). Body weight alone underestimates nutritional risk.

**Caloric target:** ~25–35 kcal/kg/day → approximately **1,350–1,890 kcal/day**, titrated to maintain weight and muscle mass. Given her MASLD context, target the **lower-to-middle range (~1,600–1,800 kcal)** to avoid excess caloric load, while avoiding caloric restriction < 1,200 kcal.

**2. Recommended Daily Protein Intake**

| **Guideline** | **Recommendation** |
| --- | --- |
| EASL / ESPEN (Liver Disease) | **1.2–1.5 g/kg/day** |
| For this patient (54 kg) | **~65–80 g protein/day** |

**Critical points:**

- **Do NOT restrict protein** — the outdated practice of protein restriction to prevent hepatic encephalopathy (HE) is **contraindicated** in compensated cirrhosis. Adequate protein is essential to prevent sarcopenia, which itself worsens prognosis.
- Since this is a **MASLD** patient, prioritize **plant-based and lean protein sources** (see Section 4), which also improve insulin sensitivity.
- Branch-Chain Amino Acid (BCAA) supplementation (leucine, isoleucine, valine) may be considered if dietary protein targets are difficult to achieve, but is **not routinely mandatory** in compensated, stable disease.

**3. Meal Frequency and Eating Pattern**

**Recommended: 4–6 small meals per day**, including a mandatory **late-evening snack (LES).**

Breakfast	7:00–8:00 AM

Mid-morning	10:00–10:30 AM

Lunch	12:00–1:00 PM

Afternoon snack 3:00–4:00 PM

Dinner	6:00–7:00 PM

Late-evening snack (LES) ★ 9:00–10:00 PM

**★ Why the Late-Evening Snack Matters**

Cirrhotic patients experience **accelerated starvation physiology** — after as little as 4–6 hours of fasting, they begin catabolizing muscle for gluconeogenesis (a process that takes 24–48 hours in healthy individuals). The LES **minimizes the overnight fasting window** and reduces muscle protein breakdown.

**LES should contain:**

- **50 g complex carbohydrates** (e.g., whole grain crackers, oatmeal, a small banana)
- **10–15 g protein** (e.g., low-fat milk, soy milk, small portion of yogurt or nuts)
- Target: ~200 kcal

**4. Specific Food Recommendations and Restrictions**

**Recommended Foods**

| **Category** | **Examples** | **Rationale** |
| --- | --- | --- |
| **Lean proteins** | Fish (salmon, mackerel, tuna), skinless poultry, eggs, tofu, legumes, low-fat dairy | Hepatoprotective, insulin-sensitizing |
| **Complex carbohydrates** | Oats, brown rice, quinoa, whole-grain bread, sweet potato | Low glycemic index; avoids metabolic burden |
| **Vegetables** | All non-starchy vegetables (broccoli, spinach, kale, zucchini, mushrooms) | High fiber, micronutrients, anti-inflammatory |
| **Fruits** | Berries, apples, citrus (moderate portions; 1–2 servings/day) | Antioxidants; limit high-fructose fruits |
| **Healthy fats** | Olive oil (primary fat source), avocado, walnuts, flaxseed | Mediterranean-pattern fats; anti-steatotic |
| **Coffee** | 2–3 cups/day (unsweetened) | Strong evidence for reduced fibrosis progression in MASLD |

**Foods to Avoid or Restrict**

| **Category** | **Examples** | **Reason** |
| --- | --- | --- |
| **Alcohol** | All forms — beer, wine, spirits | **Absolute contraindication** in cirrhosis regardless of etiology |
| **Added sugar & fructose** | Soda, fruit juice, candy, pastries, sweetened beverages | Directly drives hepatic steatosis and insulin resistance |
| **Ultra-processed foods** | Fast food, packaged snacks, processed meats | High in saturated fat, additives, sodium |
| **Refined carbohydrates** | White bread, white rice, sugary cereals | High glycemic load, worsen insulin resistance |
| **Excessive saturated fats** | Fatty red meat, full-fat dairy, butter, coconut oil | Promote hepatic inflammation and steatosis |
| **Raw shellfish** | Oysters, clams, raw mussels | Risk of *Vibrio vulnificus* — **life-threatening in cirrhosis** |
| **High-sodium foods** | Processed/canned foods, pickles, soy sauce, deli meats | Even in compensated disease, limit sodium to **< 2 g/day** to prevent ascites |

**5. Additional Nutritional Considerations**

**Micronutrient Monitoring**

Cirrhosis commonly causes micronutrient deficiencies. Check and supplement as needed:

| **Nutrient** | **Clinical Relevance** | **Action** |
| --- | --- | --- |
| **Vitamin D** | Very commonly deficient in MASLD-cirrhosis; associated with fibrosis severity | Check 25-OH Vitamin D; supplement to maintain ≥ 30 ng/mL |
| **Zinc** | Deficiency contributes to HE risk and immune dysfunction | Supplement if deficient (zinc supplementation may reduce HE episodes) |
| **Vitamin E (α-tocopherol)** | 800 IU/day has evidence for reducing steatohepatitis in MASLD | Consider in MASLD patients without cirrhosis-related contraindications; discuss risk/benefit |
| **B vitamins (B1, B6, B12, folate)** | Impaired hepatic storage and metabolism | Screen; supplement as indicated |
| **Magnesium** | Often depleted | Check and replete |
| **Iron** | Avoid iron supplementation unless documented deficiency — excess iron is hepatotoxic | Check ferritin; do NOT supplement empirically |

**Physical Activity (Dietary Adjunct)**

- **Encourage regular moderate aerobic exercise**: 150 min/week of brisk walking, cycling, or swimming
- **Add resistance/strength training** 2–3×/week to combat sarcopenia
- Exercise independently improves insulin sensitivity and hepatic steatosis in MASLD

**Metabolic Risk Factor Management**

Given the MASLD etiology, dietary counseling must address:

- **Weight management:** A **5–10% body weight reduction** (if overweight/obese) dramatically improves steatohepatitis — however, this patient is already normal BMI, so focus on body composition (muscle preservation) rather than weight loss
- **Glycemic control:** If pre-diabetes or diabetes coexists, coordinate with endocrinology; dietary fiber and low-GI foods are first-line
- **Lipid management:** Mediterranean-type diet is preferred over pharmacotherapy where possible

**Follow-Up Monitoring**

- Reassess nutritional status every **3–6 months**: weight, handgrip strength, mid-arm muscle circumference, or BIA (bioelectrical impedance analysis) if available
- Screen for transition to **decompensated cirrhosis** (ascites, varices, HE) — dietary recommendations shift significantly if decompensation occurs

**Summary Card**

| **Parameter** | **Target** |
| --- | --- |
| Calories | ~1,600–1,800 kcal/day |
| Protein | **65–80 g/day** (1.2–1.5 g/kg) |
| Sodium | **< 2,000 mg/day** |
| Meals | **5–6/day** + mandatory late-evening snack |
| Alcohol | **Absolutely prohibited** |
| Dietary pattern | **Mediterranean-style**, low-fructose, low-refined carbohydrate |
| Key supplements | Vitamin D, Zinc (check levels first) |

**Clinical note:** This patient is at the intersection of metabolic liver disease and cirrhosis-specific nutritional needs. A Mediterranean-inspired, moderate-protein, low-glycemic diet with consistent meal timing is the cornerstone of management. Regular reassessment and a collaborative approach with a registered dietitian are strongly recommended.

## C103

**Dietary & Nutritional Recommendations**

**Patient C1-03 | Male, 42 y/o | Alcohol-Related Compensated Cirrhosis**

**BMI: 29.1 kg/m² (Overweight)** | Child-Pugh: Compensated (Class A assumed)

**1. Overall Dietary Principles**

- **Complete and permanent alcohol abstinence** — the single most critical intervention; continued alcohol use dramatically accelerates fibrosis progression and decompensation
- Adopt a **Mediterranean-style dietary pattern**: emphasis on whole grains, vegetables, legumes, olive oil, and lean protein
- Aim for **modest caloric restriction** given overweight status, targeting ~1,800–2,000 kcal/day, while avoiding aggressive caloric deficit that could precipitate muscle wasting (sarcopenia)
- Prioritize **anti-inflammatory, low-processed-food** choices to reduce hepatic oxidative stress
- Maintain adequate **micronutrient density** due to high risk of deficiency from prior alcohol use

**2. Recommended Daily Protein Intake**

| **Parameter** | **Recommendation** |
| --- | --- |
| **Target intake** | **1.2–1.5 g/kg body weight/day** (~98–123 g/day) |
| **Rationale** | Cirrhosis drives accelerated protein catabolism; adequate intake prevents sarcopenia and hepatic encephalopathy |
| **Protein restriction?** | **Not recommended** — outdated practice; protein restriction worsens outcomes |
| **Preferred sources** | Plant proteins (legumes, soy) + dairy (BCAA-rich whey) + lean poultry/fish |
| **Red/processed meat** | Minimize — increases ammonia load and colorectal cancer risk |

**Note on ammonia:** Vegetable-source protein is preferred as it produces less ammonia and has added fiber benefit for gut transit.

**3. Meal Frequency and Eating Pattern**

Given impaired hepatic glycogen storage in cirrhosis, **prolonged fasting is dangerous** and must be avoided.

Recommended Daily Schedule:

┌─────────────────────────────────────────────────┐

│ Breakfast	07:00 – 08:00	│

│ Mid-Morning	10:00 – 10:30 (snack)	│

│ Lunch	12:30 – 13:00	│

│ Afternoon	15:30 – 16:00 (snack)	│

│ Dinner	18:30 – 19:00	│

│ ★ Late Evening 21:00 – 22:00 (LES — critical) │

└─────────────────────────────────────────────────┘

- **4–6 small meals per day** rather than 2–3 large meals
- **Late Evening Snack (LES)** is an evidence-based intervention: 200–300 kcal containing **complex carbohydrate + protein** (e.g., whole grain crackers with peanut butter, or yogurt with oats) — reduces overnight fasting duration and improves nitrogen balance
- **Do not skip breakfast** — fasting overnight is already long enough given impaired gluconeogenesis

**4. Specific Food Recommendations & Restrictions**

**Encouraged Foods**

| **Category** | **Examples** |
| --- | --- |
| **Vegetables** | Leafy greens, broccoli, carrots, tomatoes, beets |
| **Fruits** | Berries, citrus, apples (high antioxidants) |
| **Whole grains** | Oats, brown rice, quinoa, whole wheat bread |
| **Legumes** | Lentils, chickpeas, black beans — excellent plant protein |
| **Lean protein** | Chicken breast, turkey, eggs, tofu, low-fat dairy |
| **Fish** | Salmon, mackerel, sardines (omega-3, 2–3×/week) |
| **Healthy fats** | Extra-virgin olive oil, avocado, nuts (in moderation) |
| **Coffee** | 2–3 cups/day — evidence supports hepatoprotective effect in cirrhosis |

**Foods/Substances to Restrict or Avoid**

| **Item** | **Reason** |
| --- | --- |
| **All alcohol** | Absolute contraindication |
| **High-sodium foods** | Limit to **<2,000 mg Na/day** — even compensated cirrhosis has risk of ascites; avoid processed/canned foods, pickles, soy sauce, deli meats |
| **Red & processed meats** | Increases ammonia, inflammatory load |
| **High-fructose corn syrup/sugary drinks** | Drives hepatic de novo lipogenesis, worsens liver steatosis |
| **Fried/ultra-processed foods** | Pro-inflammatory, high in trans fats |
| **Raw shellfish** | Risk of *Vibrio vulnificus* infection — **can be fatal** in cirrhosis |
| **Unpasteurized products** | Increased infection susceptibility |

**5. Additional Nutritional Considerations**

**Micronutrient Deficiencies (High Priority in Alcohol-Related Cirrhosis)**

| **Nutrient** | **Status** | **Action** |
| --- | --- | --- |
| **Thiamine (B1)** | Severely depleted by alcohol | Supplement: 100 mg/day orally; assess for Wernicke's |
| **Folate (B9)** | Commonly deficient | Supplement: 1 mg/day or dietary optimization |
| **Vitamin B6** | Often low | Supplement or ensure dietary adequacy |
| **Zinc** | Deficient in ~80% of cirrhotic patients | Supplement: 50 mg elemental zinc/day if deficient; supports ammonia metabolism and taste |
| **Vitamin D** | Deficient — impaired hydroxylation | Check 25-OH vitamin D; supplement 1,000–2,000 IU/day |
| **Magnesium** | Alcohol-depleted | Dietary optimization + supplement if low |
| **Vitamin A & E** | May be low | Avoid high-dose supplementation (hepatotoxic at excess); obtain through diet |

**Sarcopenia Prevention**

- This patient, despite being overweight (BMI 29.1), may have **sarcopenic obesity** — excess fat with insufficient muscle mass, common in cirrhosis
- Combine dietary protein optimization with **regular moderate physical activity**: 30–45 min of walking or resistance exercise 3–5×/week as tolerated
- Consider **branched-chain amino acid (BCAA) supplementation** (12 g/day) if dietary protein targets are difficult to meet or if early encephalopathy develops

**Monitoring Recommendations**

- Weight and waist circumference monthly
- Serum albumin, prealbumin, zinc, vitamin D quarterly
- Handgrip strength assessment at each visit (sarcopenia screening)
- Reassess sodium restriction if signs of fluid retention develop

**Hepatic Encephalopathy Prevention**

- Ensure **regular bowel movements** (1–2×/day): adequate fiber (25–35 g/day), hydration, and consider **lactulose** if constipation-prone
- Probiotic-rich foods (yogurt, kefir) may support gut microbiome and reduce ammonia-producing bacteria

**Summary Priority List:**

1. ☑ Zero alcohol — non-negotiable
2. ☑ Late evening snack every night
3. ☑ 1.2–1.5 g/kg/day protein with plant-forward approach
4. ☑ Sodium <2,000 mg/day
5. ☑ Thiamine + Zinc + Vitamin D supplementation
6. ☑ Avoid raw shellfish absolutely

## C104

**Dietary & Nutritional Recommendations**

**Patient: Female, 44 y/o | HBV-Compensated Cirrhosis | BMI 28.0 kg/m²**

**Baseline Assessment**

| **Parameter** | **Value** |
| --- | --- |
| Height | 158 cm |
| Weight | 70 kg |
| BMI | **28.0 kg/m²** (Overweight) |
| Ideal Body Weight (IBW) | ~54–58 kg |
| Disease Stage | Compensated cirrhosis (Child-Pugh A presumed) |
| Etiology | Hepatitis B (HBV) |

**Key nutritional concern:** Despite overweight BMI, patients with cirrhosis can harbor **sarcopenic obesity** — reduced muscle mass masked by excess fat. Weight alone must not be used to assess nutritional adequacy.

**1. Overall Dietary Principles**

- **Do not restrict calories excessively** — malnutrition in cirrhosis worsens prognosis even in overweight patients
- Aim for **moderate caloric intake** of **30–35 kcal/kg IBW/day** (~1,650–1,900 kcal/day based on IBW ~55 kg)
- Prioritize **hepatoprotective, anti-inflammatory** food patterns (Mediterranean-style diet preferred)
- **Avoid all alcohol** — absolute contraindication regardless of HBV etiology
- Maintain **consistent meal timing** to reduce gluconeogenic stress on the liver
- **Sodium restriction** is not strictly required at this stage (compensated, no ascites) but avoid excessive salt intake (keep <5–6 g NaCl/day as a general healthy habit)

**2. Recommended Daily Protein Intake**

**Target: 1.2–1.5 g protein/kg IBW/day**

≈ **66–82 g of protein per day** (using IBW ~55 kg)

| **Protein Guidance** | **Detail** |
| --- | --- |
| **Minimum** | 1.2 g/kg IBW/day (~66 g/day) |
| **Optimal target** | 1.3–1.5 g/kg IBW/day (~72–82 g/day) |
| **Source priority** | Plant-based + fish + lean poultry > red meat |
| **Restriction?** | **Do NOT restrict protein** — protein restriction to prevent hepatic encephalopathy (HE) is outdated and harmful |
| **HE not present** | No indication to limit protein in this stable, compensated patient |

**Preferred Protein Sources:**

- **Legumes** (lentils, tofu, edamame, chickpeas) — high BCAA, low ammoniagenic
- **Fish** (salmon, mackerel, sardines) — omega-3 benefits for hepatic steatosis
- **Eggs** — complete amino acid profile, well-tolerated
- **Lean poultry** (chicken breast, turkey)
- **Low-fat dairy** (Greek yogurt, cottage cheese)

**3. Meal Frequency and Eating Pattern**

Cirrhotic livers have reduced glycogen storage capacity → frequent meals prevent catabolism.

**Recommended: 4–6 small meals per day**, including a **mandatory late-evening snack (LES)**

| **Meal** | **Timing** | **Purpose** |
| --- | --- | --- |
| Breakfast | 7:00–8:00 AM | Break overnight fast promptly |
| Mid-morning snack | 10:00–10:30 AM | Prevent hypoglycemia, reduce gluconeogenesis |
| Lunch | 12:00–1:00 PM | Main protein-containing meal |
| Afternoon snack | 3:00–4:00 PM | Sustain energy |
| Dinner | 6:00–7:00 PM | Balanced, not heavy |
| ⭐ Late-Evening Snack (LES) | **9:00–10:00 PM** | **Critical — reduces overnight fasting** |

**⭐ Late-Evening Snack (LES) — Most Important Recommendation**

- Evidence-based intervention shown to **improve nitrogen balance and muscle mass** in cirrhosis
- Should contain **~200 kcal** with **complex carbohydrates + protein**
- Examples:
  - Whole grain crackers + peanut butter + warm milk
  - Oatmeal with Greek yogurt
  - A small banana + a boiled egg
  - Whole grain toast + cottage cheese

**Overnight fasting >6 hours** triggers muscle protein catabolism in cirrhosis — the LES directly counteracts this.

**4. Specific Food Recommendations & Restrictions**

**Recommended Foods**

| **Category** | **Examples** | **Rationale** |
| --- | --- | --- |
| **Whole grains** | Brown rice, oats, quinoa, whole wheat bread | Sustained energy, fiber, B vitamins |
| **Vegetables** | Leafy greens, broccoli, carrots, beets | Antioxidants, folate, hepatoprotective |
| **Fruits** | Berries, apples, citrus (moderate portions) | Antioxidants; monitor sugar if glucose-impaired |
| **Legumes** | Lentils, black beans, tofu, tempeh | Preferred plant protein, BCAA-rich |
| **Healthy fats** | Olive oil, avocado, walnuts, flaxseed | Anti-inflammatory, omega-3 |
| **Fish** | Salmon, sardines, mackerel (2–3×/week) | Omega-3s beneficial in NAFLD and HBV fibrosis |
| **Dairy (low-fat)** | Greek yogurt, kefir | Protein + probiotics (gut-liver axis support) |
| **Coffee** | 2–3 cups/day (black or with minimal additions) | Evidence supports hepatoprotective effect in cirrhosis |

**Foods to Avoid or Restrict**

| **Food** | **Reason** |
| --- | --- |
| **Alcohol (absolute)** | Accelerates fibrosis, triggers decompensation |
| **Raw shellfish** (oysters, clams) | Risk of *Vibrio vulnificus* — life-threatening in cirrhosis |
| **Raw/undercooked meat, eggs** | Infection risk (impaired hepatic immune function) |
| **High-fructose corn syrup, sugary drinks** | Promotes hepatic steatosis, worsens metabolic burden |
| **Ultra-processed foods** | High sodium, preservatives, trans fats |
| **Excessive red/processed meat** | High ammoniagenic potential, saturated fat |
| **High-sodium foods** | Canned soups, chips, soy sauce — habitual excess may predispose to fluid retention |
| **Herbal supplements** | Many are hepatotoxic (e.g., kava, comfrey, green tea extract) — avoid without hepatologist approval |

**5. Additional Nutritional Considerations**

**A. Micronutrient Monitoring & Supplementation**

| **Nutrient** | **Consideration** |
| --- | --- |
| **Zinc** | Commonly deficient in cirrhosis; supports ammonia metabolism and immune function. Consider supplementation (25–45 mg elemental zinc/day) if levels low |
| **Vitamin D** | Frequently low in HBV cirrhosis; monitor 25-OH Vitamin D and supplement to maintain >30 ng/mL |
| **B vitamins (B1, B6, B12, Folate)** | Impaired hepatic storage; ensure adequate dietary intake; B1 especially if any alcohol history |
| **Magnesium** | Often depleted; found in nuts, seeds, leafy greens |
| **Iron** | **Do NOT supplement routinely** — HBV cirrhosis can cause iron overload; check ferritin before any supplementation |
| **Fat-soluble vitamins (A, E, K)** | May be deficient if cholestasis is present; assess clinically |

**B. Hepatic Encephalopathy (HE) Prevention**

- No current HE in this compensated patient, but **preventive measures are prudent**
- Maintain **bowel regularity** (adequate fiber, hydration) to reduce intestinal ammonia production
- Consider **probiotic-rich foods** (yogurt, kefir) to support the gut-liver axis
- **Branched-Chain Amino Acid (BCAA) supplementation** (e.g., 12 g/day of BCAA supplement) may be considered if dietary protein goals are difficult to meet, or if sarcopenia is confirmed

**C. Weight Management (BMI 28 — Overweight)**

- **Do not pursue aggressive weight loss** — it risks accelerating sarcopenia
- If weight reduction is clinically desired, aim for **no more than 0.5 kg/week** with adequate protein preservation
- Focus on **improving body composition** (reduce fat, maintain/gain muscle) rather than weight loss per se
- Encourage **moderate physical activity** (walking, resistance training as tolerated) — exercise improves muscle mass and portal hemodynamics in compensated cirrhosis

**D. Hydration**

- Target **1.5–2 L of water/day**
- No fluid restriction needed at this compensated stage (absent ascites or hyponatremia)
- Avoid sugary beverages and energy drinks

**E. HBV-Specific Considerations**

- Ensure antiviral therapy compliance (e.g., tenofovir/entecavir) — nutritional status influences viral suppression and fibrosis progression
- Regular monitoring: LFTs, AFP, liver ultrasound every 6 months
- Avoid any **hepatotoxic supplements or herbal remedies** — this is especially critical with underlying viral hepatitis

**Summary Card for Patient**

|  | **Recommendation** |
| --- | --- |
| **Calories** | ~1,700–1,900 kcal/day |
| **Protein** | 1.2–1.5 g/kg IBW → **~70–80 g/day** |
| **Meals** | 4–6 small meals + **late-evening snack at 9–10 PM** |
| **Avoid** | Alcohol, raw shellfish, herbal supplements, ultra-processed foods |
| **Allow** | Coffee (2–3 cups/day), olive oil, legumes, fish |
| **Monitor** | Zinc, Vitamin D, B vitamins (check iron before supplementing) |
| **Exercise** | Moderate walking + light resistance training encouraged |

*These recommendations are based on current guidelines from EASL (European Association for the Study of the Liver) and ESPEN (European Society for Clinical Nutrition and Metabolism) for nutritional management of liver cirrhosis. Individual adjustments should be made based on follow-up labs, symptom progression, and patient tolerance.*

## C105

**Dietary & Nutritional Recommendations**

**Patient: Male, 47 yo | 175 cm | 92 kg | MASLD-Related Compensated Cirrhosis**

**Nutritional Assessment at a Glance**

| **Parameter** | **Value** | **Interpretation** |
| --- | --- | --- |
| BMI | 30.1 kg/m² | Obese Class I |
| Ideal Body Weight (IBW) | ~70.5 kg | Devine formula |
| Cirrhosis Stage | Compensated | No ascites/encephalopathy |
| Underlying Etiology | MASLD | Metabolic-driven; strongly modifiable |

**Key Clinical Challenge:** This patient requires a dual therapeutic strategy — supporting hepatic reserve while simultaneously achieving gradual, safe weight reduction to address the underlying MASLD pathophysiology.

**1. Overall Dietary Principles**

- **Adopt a Mediterranean-style dietary pattern** as the preferred framework: rich in whole grains, vegetables, legumes, fish, and olive oil. This pattern has the strongest evidence base for both MASLD regression and hepatoprotection.
- **Achieve a modest, sustained caloric deficit** targeting **gradual weight loss of 0.5–1.0 kg/week** (no more than 1 kg/week). Rapid weight loss (crash dieting, very low-calorie diets) is **contraindicated** — it precipitates sarcopenia, impairs hepatic regeneration, and can trigger decompensation.
- **Target 7–10% total body weight reduction** over 6–12 months, which is associated with meaningful histological improvement in MASLD.
- **Estimated daily caloric target:** ~1,750–2,100 kcal/day (25–30 kcal/kg IBW), adjusted per activity level and metabolic response.
- **Absolute alcohol abstinence** — even small amounts accelerate fibrosis progression in cirrhosis.
- Eliminate **ultra-processed foods, refined carbohydrates, and added sugars**, which directly drive hepatic steatosis and metabolic dysfunction.

**2. Recommended Daily Protein Intake**

In obese patients with cirrhosis, protein targets should be calculated based on **Ideal Body Weight (IBW)** — not actual body weight — to avoid overfeeding while preventing sarcopenic obesity.

| **Target** | **Recommendation** |
| --- | --- |
| **Daily protein goal** | **1.2–1.5 g/kg IBW/day** |
| **Calculated range** | **~85–106 g protein/day** |
| **Preferred sources** | Plant-based ≥ 50% (legumes, tofu, tempeh), supplemented with fish, poultry, eggs, low-fat dairy |
| **Branched-Chain Amino Acids (BCAA)** | Consider supplementation (7–12 g/day) if dietary protein goals are not consistently met, or if early signs of hepatic encephalopathy emerge |

- **Do NOT restrict protein** in compensated cirrhosis — this accelerates muscle loss and worsens outcomes. Protein restriction for encephalopathy prevention is an outdated practice.
- Plant-based proteins are preferred in MASLD-cirrhosis due to their lower ammonia generation, higher fiber content, and beneficial effects on gut microbiome.

**3. Meal Frequency and Eating Pattern**

Patients with cirrhosis have **reduced hepatic glycogen stores** and enter a catabolic, starvation-like state within 2–3 hours of fasting (vs. 12–16 hours in healthy individuals).

**Recommended Structure:**

Breakfast (7–8 AM)	──► Morning Snack (10 AM)	──► Lunch (12–1 PM)

↓

Afternoon Snack (3–4 PM) ──► Dinner (6–7 PM) ──► Late Evening Snack (LES) (9–10 PM)

- **4–6 small meals per day** rather than 2–3 large meals
- **Late Evening Snack (LES) is mandatory** — most critical intervention for cirrhosis nutrition:
  - Consume within **2 hours of bedtime**
  - Should contain **complex carbohydrates + protein**: e.g., whole grain toast with peanut butter, Greek yogurt with oats, or a small bowl of oatmeal with nuts
  - Reduces overnight catabolism and improves nitrogen balance
- **Do not fast for more than 3–4 hours** during waking hours
- Eat **slowly, in small volumes** per sitting to minimize bloating and early satiety (common in cirrhosis due to splanchnic congestion)

**4. Specific Food Recommendations and Restrictions**

**Encouraged Foods**

| **Category** | **Examples** | **Rationale** |
| --- | --- | --- |
| **Vegetables** | Leafy greens, broccoli, cauliflower, zucchini, bell peppers | High fiber, low glycemic, hepatoprotective phytochemicals |
| **Fruits** | Berries, apples, citrus (in moderation) | Antioxidants; limit high-fructose fruits (grapes, mangoes) |
| **Whole grains** | Oats, quinoa, brown rice, barley, whole wheat bread | Sustained energy, beta-glucan, gut microbiome support |
| **Legumes** | Lentils, chickpeas, black beans, edamame | High plant protein + fiber; low glycemic index |
| **Fish** | Salmon, sardines, mackerel, tuna (2–3×/week) | Omega-3 fatty acids reduce hepatic steatosis |
| **Lean poultry** | Skinless chicken/turkey breast | Adequate protein without excess saturated fat |
| **Healthy fats** | Extra-virgin olive oil, avocado, walnuts, almonds | Monounsaturated fats; anti-inflammatory |
| **Eggs** | Up to 1 per day | High biological value protein; acceptable in MASLD |
| **Low-fat dairy** | Greek yogurt, cottage cheese, skim milk | Calcium, protein, probiotics |
| **Coffee** | 2–3 cups/day (unsweetened) | Strong evidence for hepatoprotection and fibrosis reduction in MASLD/cirrhosis |

**Foods to Restrict or Avoid**

| **Category** | **Specific Items** | **Reason** |
| --- | --- | --- |
| **Alcohol** | All forms — absolutely prohibited | Fibrosis acceleration, decompensation risk |
| **Added sugars & fructose** | Sodas, fruit juices, candy, pastries, sweetened cereals | Primary driver of hepatic lipogenesis in MASLD |
| **Refined carbohydrates** | White bread, white rice, instant noodles | Rapid glucose spikes; worsens insulin resistance |
| **Saturated fats** | Fatty red meat, butter, full-fat dairy, coconut oil | Pro-inflammatory; worsens steatohepatitis |
| **Trans fats** | Fried fast food, commercially baked goods, margarine | Directly hepatotoxic |
| **Ultra-processed foods** | Chips, processed meats (sausage, deli meats), frozen meals | Additives, excess sodium/sugar/saturated fat |
| **High-sodium foods** | Canned soups, soy sauce, pickled foods, salted snacks | Sodium restriction not yet required (compensated), but prophylactically limit to **<2,000–2,300 mg/day** to preserve renal-hepatic reserve |
| **Raw shellfish** | Oysters, clams, raw seafood | Risk of *Vibrio vulnificus* — potentially fatal in cirrhosis |

**5. Additional Nutritional Considerations**

**Micronutrient Monitoring and Supplementation**

| **Nutrient** | **Consideration** |
| --- | --- |
| **Vitamin D** | Frequently deficient in cirrhosis; check 25(OH)D level; supplement if <30 ng/mL (typically 1,000–2,000 IU/day) |
| **Zinc** | Commonly depleted in cirrhosis; zinc deficiency contributes to hepatic encephalopathy risk; consider 25–45 mg elemental zinc/day |
| **Magnesium** | Often low; support via dietary sources (nuts, seeds, leafy greens) ± supplementation |
| **B vitamins (B1, B6, B12, Folate)** | Monitor levels; supplement as indicated; avoid excess B3 (niacin) which may be hepatotoxic in high doses |
| **Vitamin E** | 800 IU/day has evidence in non-diabetic NASH/MASLD — discuss with hepatologist before use |
| **Iron** | Avoid supplementation unless documented deficiency (iron overload worsens oxidative liver injury) |
| **Fat-soluble vitamins (A, K)** | May be malabsorbed; monitor and replace cautiously — avoid excess vitamin A (hepatotoxic) |

**Physical Activity (Synergistic with Nutrition)**

- Encourage **150 min/week of moderate aerobic exercise** (brisk walking, cycling, swimming) combined with **2× weekly resistance training**
- Exercise is independently hepatoprotective in MASLD and improves insulin sensitivity
- Avoid high-intensity exercise until cardiovascular fitness is assessed given obesity and metabolic risk profile

**Metabolic Comorbidity Management**

- Screen and manage **type 2 diabetes / insulin resistance**, **dyslipidemia**, and **hypertension** — all are drivers of MASLD progression
- Coordinate dietary plan with management of these conditions (e.g., low glycemic index diet is beneficial for both MASLD and glycemic control)

**Sarcopenia Surveillance**

- Despite obesity (BMI 30.1), **sarcopenic obesity is prevalent** in MASLD-cirrhosis and is an independent predictor of decompensation and mortality
- Consider **mid-arm muscle circumference, handgrip strength, or DEXA scan** to assess muscle mass at baseline and during follow-up
- The dietary and exercise plan above is specifically designed to promote **fat loss while preserving lean muscle mass**

**Monitoring and Follow-Up**

- Reassess nutritional status every **3–6 months**
- Monitor **liver function tests, albumin, prealbumin, CBC** alongside weight trends
- Refer to a **registered dietitian** (RD) with hepatology experience for individualized meal planning and behavioral support
- If weight loss stalls or complications emerge (ascites, encephalopathy), dietary targets must be promptly **reassessed and adjusted**

**Clinical Note:** Should this patient develop complications of cirrhosis (ascites → sodium restriction to 1,500–2,000 mg/day; hepatic encephalopathy → reassess protein source/distribution, consider BCAA therapy; variceal hemorrhage → modify texture/consistency as appropriate), the nutritional plan should be promptly revised in coordination with his hepatology team.

## C106

**Dietary & Nutritional Recommendations**

**Patient:** Female, 51 y/o | 162 cm | 60 kg | BMI: 22.9 kg/m² **Diagnosis:** Alcohol-related compensated cirrhosis

**1. Overall Dietary Principles**

- **Absolute alcohol abstinence** — the single most critical intervention; continued drinking accelerates progression to decompensation and hepatocellular carcinoma
- Aim for a **high-calorie, adequate-protein, nutrient-dense** diet to prevent and treat the malnutrition that is nearly universal in cirrhosis
- **Target energy intake: ~2,100–2,400 kcal/day** (35–40 kcal/kg IBW), as hypermetabolism is common even in compensated disease
- Avoid prolonged fasting; the cirrhotic liver has markedly reduced glycogen stores and switches rapidly to protein catabolism
- Prioritize **anti-inflammatory, whole-food** choices; minimize processed foods, added sugars, and saturated fats to reduce ongoing hepatic steatosis and oxidative stress

**2. Recommended Daily Protein Intake**

| **Parameter** | **Recommendation** |
| --- | --- |
| **Target** | **1.2–1.5 g protein/kg body weight/day** |
| **Absolute daily amount** | **~72–90 g protein/day** |
| **Preferred sources** | Plant-based proteins, dairy (whey/casein), fish, lean poultry |
| **Protein restriction?** | **Not recommended** — outdated practice; restriction worsens sarcopenia |

**Sarcopenia** is a major independent predictor of mortality in cirrhosis. Even without overt hepatic encephalopathy (HE), this patient should maintain full protein intake. Restriction is only considered if HE is refractory and uncontrolled by other means.

- **Branched-chain amino acids (BCAAs):** Consider BCAA-enriched oral supplements (e.g., 12–15 g BCAA/day) if dietary protein intake goals are difficult to meet, or if mild cognitive symptoms emerge — evidence supports BCAA supplementation in reducing HE risk and improving nutritional status in cirrhosis

**3. Meal Frequency & Eating Pattern**

This is one of the most impactful and underutilized interventions in cirrhosis nutrition:

- **4–6 small meals per day** rather than 2–3 large meals
- Include a **mandatory late-evening snack (LES)** — ideally within 1–2 hours of bedtime
  - Target: ~200–300 kcal with ~15–20 g complex carbohydrate + protein (e.g., whole grain crackers with cottage cheese, or a glass of milk with a small banana)
  - **Rationale:** Overnight fasting exceeding 6 hours should be avoided; the LES reduces overnight gluconeogenesis from muscle protein, measurably reducing nitrogen loss and preserving lean mass
- **Do not skip breakfast** — begin eating within 1 hour of waking

**4. Specific Food Recommendations & Restrictions**

**Encouraged Foods**

| **Category** | **Examples** |
| --- | --- |
| **Plant proteins** | Lentils, chickpeas, tofu, edamame, tempeh |
| **Dairy proteins** | Low-fat Greek yogurt, cottage cheese, milk, kefir |
| **Lean animal protein** | Fish (salmon, tuna, sardines), skinless poultry, eggs |
| **Complex carbohydrates** | Oats, brown rice, quinoa, sweet potato, whole grain bread |
| **Healthy fats** | Olive oil (primary fat source), avocado, walnuts, flaxseed |
| **Vegetables** | All varieties; especially cruciferous (broccoli, cauliflower) and leafy greens |
| **Fruits** | Berries, citrus, apple — moderate portions |

**Foods to Restrict or Avoid**

| **Item** | **Reason** |
| --- | --- |
| **All alcohol** | Direct hepatotoxin; accelerates fibrosis progression |
| **Raw shellfish/raw fish** | Risk of *Vibrio vulnificus* — potentially fatal in cirrhosis |
| **High-sodium foods** (>2,000 mg Na/day) | Even in compensated disease, sodium loading promotes fluid retention; restrict to ≤2 g/day proactively |
| **Processed meats, deli meats** | High sodium, nitrates, saturated fat |
| **Sugary beverages, fructose-rich foods** | Promote hepatic steatosis and metabolic stress |
| **High-fat/fried foods** | Impaired fat digestion due to reduced bile acid circulation |
| **Herbal supplements, unprescribed supplements** | Many are hepatotoxic (e.g., kava, comfrey, green tea extract) |

**5. Additional Nutritional Considerations**

**Micronutrient Deficiencies (Common in Alcohol-related Cirrhosis)**

Alcohol-related liver disease carries a particularly high burden of micronutrient depletion — active supplementation is warranted:

| **Nutrient** | **Recommendation** | **Rationale** |
| --- | --- | --- |
| **Thiamine (B1)** | 100 mg/day orally (or IV if any neurological signs) | Critical — prevents Wernicke's encephalopathy; depleted by alcohol |
| **Folate** | 1 mg/day | Poor intake + alcohol-mediated malabsorption |
| **Vitamin B6 (Pyridoxine)** | 50 mg/day | Commonly depleted |
| **Zinc** | 25–45 mg elemental zinc/day | Zinc deficiency worsens HE susceptibility and immune function |
| **Vitamin D** | 1,000–2,000 IU/day (check 25-OH-D level) | Cholestasis impairs fat-soluble vitamin absorption; high fracture risk |
| **Magnesium** | Dietary + consider supplementation if low | Alcohol causes significant renal magnesium wasting |
| **Vitamin K** | Dietary emphasis on leafy greens; supplement if INR elevated | Fat-soluble vitamin; impaired synthetic and absorptive capacity |

**Avoid high-dose fat-soluble vitamin supplements** (particularly vitamin A) without monitoring — hepatotoxic in excess.

**Additional Clinical Considerations**

- **Monitor weight weekly** — even stable compensated cirrhosis can shift fluid status; a gain of >2 kg/week warrants reassessment for occult ascites
- **Frailty & sarcopenia assessment** — consider a handgrip strength test or 6-minute walk test at baseline; mid-arm muscle circumference can help track lean mass over time
- **Glycemic monitoring** — hepatogenous diabetes occurs in up to 30–40% of cirrhotic patients; a low-glycemic-index diet and fasting glucose monitoring are prudent
- **Osteoporosis screening** — recommend DEXA scan; alcohol and cholestasis both impair bone density
- **Smoking cessation** — if applicable; compounds cardiovascular and hepatic risk
- **Referral to a registered dietitian (RD)** with hepatology experience for ongoing individualized follow-up and dietary recall assessment

**Summary Priority List:**

1. Complete alcohol abstinence
2. 4–6 meals/day + mandatory late-evening snack
3. 72–90 g protein/day — do NOT restrict
4. Sodium ≤ 2 g/day
5. Thiamine + zinc + vitamin D supplementation
6. No raw shellfish — ever

## C107

**Dietary & Nutritional Recommendations**

**Patient:** Male, 55 y/o | 170 cm / 64 kg | BMI: 22.2 kg/m² (Normal) **Diagnosis:** HBV-related Compensated Cirrhosis (Child-Pugh A)

**1. Overall Dietary Principles**

This patient has **compensated cirrhosis with normal nutritional status**, but is at ongoing risk for protein-energy malnutrition due to impaired hepatic metabolism. The core goals are:

- **Maintain current weight and muscle mass** — avoid both weight loss and excessive gain
- **Adequate energy intake:** **30–35 kcal/kg/day** → approximately **1,920–2,240 kcal/day**
- Prioritize **anti-inflammatory, liver-supportive foods** while avoiding hepatotoxic substances
- **Absolutely abstain from alcohol** (critical in HBV cirrhosis regardless of compensation status)
- Limit but do not eliminate sodium unnecessarily at this stage (no ascites currently)

**2. Recommended Daily Protein Intake**

| **Parameter** | **Recommendation** |
| --- | --- |
| **Target intake** | **1.2–1.5 g/kg/day** |
| **Daily amount** | **~77–96 g protein/day** |
| **Preferred sources** | Branched-chain amino acid (BCAA)-rich foods |
| **Restriction?** | Do NOT restrict protein — outdated practice |

**Key point:** Protein restriction to "protect the liver" is **contraindicated** in compensated cirrhosis. Sarcopenia is a major independent predictor of poor outcomes. This patient should maintain robust protein intake.

**Preferred protein sources:**

- **Plant proteins** (tofu, edamame, legumes) — favorable ammonia profile
- **Dairy** (Greek yogurt, cottage cheese, eggs) — high BCAA content
- **Lean fish and poultry** — easily digestible

**3. Meal Frequency and Eating Pattern**

Cirrhotic patients have **reduced hepatic glycogen storage** and enter a fasted/catabolic state rapidly. A structured eating pattern is essential:

Breakfast ── Morning Snack ── Lunch ── Afternoon Snack ── Dinner ── Late-Evening Snack

MOST IMPORTANT meal

- **4–6 small meals per day** (rather than 2–3 large meals)
- **Late-evening snack (LES) is mandatory:** 150–200 kcal carbohydrate + protein snack within **2 hours of bedtime**
  - Examples: whole-grain crackers + peanut butter, banana + Greek yogurt, oatmeal with milk
- **Do not fast for more than 3–4 hours** during the day
- Fasting overnight should not exceed **≤ 12 hours** (LES minimizes this)

**The LES is one of the most evidence-based interventions in cirrhosis nutrition** — it reduces overnight gluconeogenesis from muscle protein breakdown.

**4. Specific Food Recommendations & Restrictions**

**Recommended Foods**

| **Category** | **Examples** | **Rationale** |
| --- | --- | --- |
| **Complex carbohydrates** | Brown rice, oats, whole grain bread, sweet potato | Sustained glucose, fiber |
| **BCAA-rich proteins** | Eggs, Greek yogurt, tofu, lentils, fish | Muscle preservation |
| **Healthy fats** | Olive oil, avocado, nuts (moderate) | Anti-inflammatory |
| **Vegetables** | Leafy greens, broccoli, carrots, beets | Antioxidants, folate |
| **Fruits** | Berries, citrus, papaya | Vitamin C, antioxidants |
| **Coffee** | 2–3 cups/day (unsweetened) | Evidence supports reduced fibrosis progression |

**Foods/Substances to Restrict or Avoid**

| **Item** | **Reason** |
| --- | --- |
| **Alcohol** | Absolute contraindication — accelerates fibrosis, triggers decompensation |
| **Raw shellfish/seafood** | Risk of *Vibrio vulnificus* — potentially fatal in cirrhosis |
| **High-sodium processed foods** | Prophylactic; prevent future fluid retention |
| **High-fructose corn syrup / sugary drinks** | Promotes hepatic steatosis, metabolic stress |
| **Aflatoxin-contaminated foods** | Moldy grains/nuts — synergistic hepatocarcinogenesis with HBV |
| **Herbal supplements / TCM** | Many are directly hepatotoxic |
| **Excess red/processed meat** | Increases ammonia load, cardiovascular risk |

**5. Additional Nutritional Considerations**

**Micronutrient Monitoring**

Cirrhosis frequently causes micronutrient deficiencies even at the compensated stage:

| **Nutrient** | **Status in Cirrhosis** | **Action** |
| --- | --- | --- |
| **Zinc** | Commonly depleted | Consider supplementation (15–30 mg/day); supports ammonia metabolism |
| **Vitamin D** | Frequently low | Check 25-OH Vit D; supplement if < 30 ng/mL |
| **B vitamins (B1, B6, B12, Folate)** | Often deficient | Dietary emphasis; consider B-complex supplement |
| **Vitamin E** | May be low | Dietary sources (nuts, seeds, olive oil) |
| **Iron** | **Do NOT supplement** unless documented deficiency — excess iron is hepatotoxic in HBV cirrhosis |  |

**BCAA Supplementation**

- Consider **oral BCAA supplementation (12–15 g/day)** if dietary intake is insufficient or sarcopenia develops
- Particularly beneficial for patients with low appetite or early hepatic encephalopathy

**Surveillance & Follow-up**

- **Monitor weight monthly** — unintentional loss > 2 kg/month warrants urgent reassessment
- **Hand-grip strength** and calf circumference as simple sarcopenia screening tools
- Reassess dietary plan with each clinical follow-up (every 3–6 months)
- If liver disease progresses (development of ascites, HE, or variceal bleeding), dietary strategy must be **promptly revised**

**HBV-Specific Considerations**

- Ensure continued **antiviral therapy compliance** — nutritional status and antiviral efficacy are interdependent
- Maintain **healthy BMI** — both underweight *and* obesity independently worsen fibrosis outcomes in HBV cirrhosis
- Annual **HCC surveillance** (AFP + ultrasound) should complement nutritional care

**Summary for the Patient:** *"Eat small, frequent meals throughout the day, never skip breakfast, and always have a light snack before bed. Focus on eggs, tofu, yogurt, fish, and vegetables. Never drink alcohol, avoid raw seafood, and do not take any herbal medicines without checking with your doctor first."*

## C108

**Dietary & Nutritional Recommendations**

**Patient:** Female, 58 y/o | 156 cm / 78 kg | BMI: 32.1 kg/m² (Obese Class I) **Diagnosis:** MASLD-related Compensated Cirrhosis + Non-bleeding Esophageal Varices

**Key Clinical Tensions in This Case**

This case requires carefully **balancing two competing imperatives:**

- **Weight loss is indicated** (obesity drives MASLD progression) — but must be *gradual and controlled*
- **Nutritional adequacy must be preserved** — aggressive caloric restriction risks sarcopenia and decompensation
- **Esophageal varices add a third dimension** — dietary texture and eating behavior require specific modification

**1. Overall Dietary Principles**

| **Goal** | **Target** |
| --- | --- |
| **Caloric strategy** | Modest hypocaloric diet — **not** aggressive restriction |
| **Energy target** | **25–30 kcal/kg actual BW/day → ~1,950–2,340 kcal/day** |
| **Practical target with deficit** | **~1,600–1,900 kcal/day** (≈500 kcal/day deficit) |
| **Weight loss rate** | **0.5–1.0 kg/week maximum** — faster loss worsens sarcopenia & portal hypertension |
| **Dietary pattern** | **Mediterranean diet** — strongest evidence base for MASLD |
| **Alcohol** | Absolute contraindication |

**Critical:** Rapid weight loss (>1.5 kg/week) in cirrhosis can precipitate hepatic decompensation, worsen portal hypertension, and accelerate muscle wasting. *Slow and steady is the only safe approach.*

**Body Weight Reference for Calculations**

Since the patient is obese, use **Adjusted Body Weight (AdjBW)** for protein and detailed calculations:

Ideal Body Weight (IBW, Hamwi) = 45.5 + 2.3 × 1.4 inches over 5 ft ≈ 48.7 kg

Adjusted BW = IBW + 0.4 × (Actual – IBW) = 48.7 + 0.4 × (78 – 48.7) ≈ 60.4 kg

**2. Recommended Daily Protein Intake**

| **Parameter** | **Recommendation** |
| --- | --- |
| **Target** | **1.2–1.5 g/kg AdjBW/day** |
| **Daily amount** | **~72–90 g protein/day** |
| **Do NOT reduce protein** despite obesity | Sarcopenia is present in up to 40% of obese cirrhotic patients ("sarcopenic obesity") |
| **Priority sources** | Plant-based > lean animal protein |

**Why Protein Matters More in MASLD Cirrhosis**

- MASLD is strongly associated with **sarcopenic obesity** — excess fat mass coexists with critically low muscle mass
- Standard BMI *overestimates* nutritional status in these patients — muscle mass should be formally assessed
- **Hand-grip strength** and **calf circumference measurement** are recommended at baseline

**3. Meal Frequency and Eating Pattern**

**Daily Meal Structure**

7:00 AM	Breakfast	— Never skip; include protein + complex carb

10:00 AM Morning snack	— Small, protein-containing

1:00 PM	Lunch	— Largest meal of the day (better metabolic tolerance)

4:00 PM	Afternoon snack — Fruit + nuts or yogurt

7:00 PM	Dinner	— Moderate size, soft-textured

9:30 PM	Late-Evening Snack (LES) ← MANDATORY

- **4–6 small meals daily** — reduces metabolic demand per meal and prevents hypoglycemia
- **Late-Evening Snack (LES):** 150–200 kcal of complex carbohydrate + protein within 2 hours of bedtime
  - Examples: warm oat porridge with low-fat milk, wholegrain crackers + cottage cheese, banana + yogurt
- **Overnight fast ≤ 12 hours**
- **Eat slowly, chew thoroughly** — critical given esophageal varices (see Section 4)

**4. Specific Food Recommendations & Restrictions**

**MASLD-Targeted Dietary Recommendations**

**🫒 Mediterranean Diet Framework**

| **Food Group** | **Specific Recommendations** | **Daily/Weekly Target** |
| --- | --- | --- |
| **Vegetables** | All cooked/soft varieties; leafy greens, zucchini, carrots, beets | ≥ 5 servings/day |
| **Fruits** | Berries, citrus, pears, melons (moderate portion) | 2–3 servings/day |
| **Whole grains** | Oats, brown rice, quinoa, whole grain bread | 3–4 servings/day |
| **Legumes** | Lentils, chickpeas, soft-cooked beans | 4–5 times/week |
| **Fish** | Oily fish (salmon, sardines, mackerel) — omega-3 rich | 2–3 times/week |
| **Olive oil** | Extra virgin — primary cooking fat | 1–2 tbsp/day |
| **Nuts/seeds** | Walnuts, almonds, flaxseed (small portions) | Small handful/day |
| **Low-fat dairy** | Greek yogurt, cottage cheese, low-fat milk | 1–2 servings/day |
| **Eggs** | Excellent BCAA source, easy to prepare softly | 4–5/week |
| **Coffee** | Unsweetened black or with low-fat milk | 2–3 cups/day |

**Coffee:** Strong evidence supports 2–3 cups/day reducing hepatic fibrosis progression, liver-related mortality, and HCC risk in MASLD cirrhosis.

**Esophageal Varices — Dietary Texture & Behavior Modifications**

Non-bleeding varices do **not** require a liquid diet — but specific precautions are essential to avoid **traumatic variceal rupture** from food.

**Foods to Avoid Due to Variceal Risk**

| **Category** | **Examples** | **Reason** |
| --- | --- | --- |
| **Hard, sharp-edged foods** | Crusty bread crusts, hard crackers, raw carrot sticks, whole nuts | Mechanical trauma to varices |
| **Very coarse fibrous foods** | Tough fibrous meat, whole corn kernels, raw celery | Abrasive friction |
| **Extremely hot foods/drinks** | Boiling soups, very hot tea | Vasodilation, mucosal injury |
| **Very large boluses** | Eating too fast, large chunks | Pressure trauma |
| **Carbonated beverages** | Sparkling water, soda | Increased intragastric pressure |

**Safe Eating Practices for Varices**

Cook all vegetables until soft-tender

Choose well-cooked, tender cuts of fish/poultry rather than tough red meat

Break bread into small pieces; avoid crusty baguettes

Chew every bite 20–30 times before swallowing

Sit upright during and 30 minutes after meals

Eat slowly — minimum 20–30 minutes per meal

Grind or finely chop nuts if desired

Allow hot foods/drinks to cool to warm before consuming

**MASLD-Specific Restrictions**

| **Item** | **Reason** | **Alternative** |
| --- | --- | --- |
| **Alcohol** | Absolute contraindication | Sparkling water with lemon (room temp) |
| **Added sugars / SSBs** | Fructose directly drives hepatic steatosis | Water, unsweetened tea, coffee |
| **High-fructose corn syrup** | Present in many packaged foods — read labels | Whole fruit (moderate) |
| **Refined carbohydrates** | White bread, white rice, pastries — spike insulin, promote fat deposition | Whole grain alternatives |
| **Saturated / trans fats** | Red meat, full-fat dairy, fried foods, pastries | Olive oil, fish, avocado |
| **Ultra-processed foods** | Chips, instant noodles, fast food | Home-cooked meals |
| **Sodium > 2g/day** | Prophylactic — prevent ascites development | Herbs, lemon, spices for flavor |
| **Raw shellfish** | Fatal *Vibrio* risk in cirrhosis | Fully cooked seafood only |
| **Herbal supplements** | Multiple are directly hepatotoxic | Consult physician before any supplement |

**5. Additional Nutritional Considerations**

**Micronutrient Profile in MASLD Cirrhosis**

| **Nutrient** | **Clinical Relevance** | **Action** |
| --- | --- | --- |
| **Vitamin E (α-tocopherol)** | Has shown histological benefit in NASH/MASLD; antioxidant | Dietary sources (sunflower seeds, almonds, olive oil); discuss supplementation with hepatologist |
| **Vitamin D** | Deficiency common; worsens insulin resistance and fibrosis | Check 25-OH Vit D; supplement to maintain > 30 ng/mL |
| **Zinc** | Depleted in cirrhosis; supports insulin signaling and ammonia metabolism | Consider 15–30 mg/day |
| **Magnesium** | Often low in metabolic syndrome; insulin co-factor | Dietary emphasis: leafy greens, nuts, legumes |
| **B vitamins (B1, B12, Folate)** | Impaired hepatic storage | Dietary variety; consider B-complex |
| **Iron** | **Do NOT supplement empirically** — assess ferritin/transferrin first | Excess iron worsens oxidative stress in MASLD |
| **Omega-3 fatty acids** | Reduces hepatic steatosis and triglycerides | 2–3 portions oily fish/week; discuss EPA/DHA supplement |

**Metabolic Syndrome Management**

Given MASLD etiology, this patient almost certainly has underlying metabolic comorbidities:

**Likely comorbidities to screen/manage alongside nutrition:**

- Type 2 diabetes / insulin resistance → **reduce glycemic load, increase fiber**
- Hypertriglyceridemia → **reduce refined carbs + sugar; increase omega-3**
- Hypertension → **DASH + Mediterranean principles, sodium restriction**
- Hypothyroidism (common in women 58 y/o) → **check TSH if not already done**

**Bone Health (Post-menopausal Female)**

- Cirrhosis + estrogen decline = **high osteoporosis risk**
- **Calcium:** 1,000–1,200 mg/day from dietary sources (low-fat dairy, fortified plant milks, tofu, sardines with bones)
- **Vitamin D:** as above — essential for calcium absorption
- Consider **DEXA scan** if not recently performed

**Physical Activity Integration**

Nutrition alone is insufficient for MASLD — exercise is a co-treatment:

| **Type** | **Recommendation** |
| --- | --- |
| **Aerobic** | 150 min/week moderate intensity (walking, swimming, cycling) |
| **Resistance** | 2–3x/week — critical for preserving/building muscle mass |
| **Avoid** | High-impact or contact sports (variceal bleed risk with Valsalva) |
| **Start** | Gradually, supervised if deconditioned |

**Resistance training + adequate protein = the most effective intervention against sarcopenic obesity in MASLD cirrhosis**

**Monitoring & Follow-up Plan**

| **Parameter** | **Frequency** |
| --- | --- |
| Weight, BMI | Monthly |
| Hand-grip strength / calf circumference | Every 3 months |
| Liver function, albumin, INR | Every 3–6 months |
| Lipid panel, HbA1c, fasting glucose | Every 6 months |
| 25-OH Vitamin D, Zinc, B12 | Annually or as clinically indicated |
| Upper endoscopy (variceal surveillance) | Per hepatologist schedule (typically every 1–2 years) |
| Hepatocellular carcinoma surveillance | Every 6 months (ultrasound ± AFP) |

**Summary for the Patient:** *"Follow a Mediterranean-style diet — plenty of colorful vegetables (cooked soft), fish, olive oil, legumes, and whole grains. Lose weight slowly — no crash diets. Eat 5–6 small meals a day and always have a small snack before bed. Because of your esophageal varices, chew everything very thoroughly, avoid hard or crunchy foods, and eat slowly. No alcohol ever, limit sugar and white carbohydrates, and check with your doctor before taking any supplements or herbal medicines."*

## C109

**Dietary & Nutritional Recommendations**

**Patient:** Male, 61 y/o | 167 cm / 58 kg | BMI: 20.8 kg/m² (Low-normal — Malnutrition Risk) **Diagnosis:** Alcohol-related Compensated Cirrhosis (ALC)

**Critical Clinical Context for This Case**

Alcohol-related cirrhosis carries the **highest prevalence of malnutrition** among all cirrhosis etiologies — affecting **up to 100% of patients** to varying degrees. This patient's BMI of 20.8, while technically "normal," is **deceptively reassuring**: alcohol-related liver disease causes severe **micronutrient depletion, muscle wasting, and fat-soluble vitamin deficiency** that are invisible on standard weight metrics alone.

**Three compounding risk factors require simultaneous management:**

① Cirrhosis-related catabolism & malabsorption

+

② Chronic alcohol-induced specific nutrient depletion

+

③ Age-related sarcopenia (61 y/o male)

↓

High-priority nutritional rehabilitation required

**1. Overall Dietary Principles**

| **Goal** | **Target** |
| --- | --- |
| **Energy intake** | **35–40 kcal/kg/day** (higher end due to malnutrition risk) |
| **Daily caloric target** | **~2,030–2,320 kcal/day** |
| **Caloric strategy** | **Repletion diet** — not restriction, not maintenance |
| **Dietary pattern** | Balanced, nutrient-dense whole foods; Mediterranean-influenced |
| **Alcohol** | **Absolute and permanent abstinence — #1 priority** |
| **Caloric replacement** | Alcohol previously provided empty calories; replace with **nutrient-dense complex carbohydrates and protein** |

**Why Higher Calories Are Justified**

- Alcohol-related liver disease causes a **hypermetabolic state** in many patients
- Chronic alcohol use suppresses appetite → patients are often eating far less than estimated
- At BMI 20.8 with likely underlying **sarcopenia**, energy stores are already marginal
- Risk of **refeeding syndrome** must be monitored if patient was severely restricting food intake while drinking (see Section 5)

**Alcohol Abstinence is Non-Negotiable:** Even a single drinking relapse in compensated ALC dramatically accelerates fibrosis, precipitates acute-on-chronic liver failure (ACLF), and eliminates transplant candidacy. Nutrition counseling must be coupled with **addiction medicine referral and psychosocial support**.

**2. Recommended Daily Protein Intake**

| **Parameter** | **Recommendation** |
| --- | --- |
| **Target intake** | **1.5 g/kg/day** (upper range — prioritize due to sarcopenia risk) |
| **Daily amount** | **~87–100 g protein/day** |
| **Preferred sources** | BCAA-rich proteins; plant and lean animal proteins |
| **Restrict protein?** | Absolutely contraindicated — no evidence supports restriction in compensated cirrhosis |

**Protein Strategy Specific to ALC**

| **Priority** | **Rationale** |
| --- | --- |
| **Maximize BCAA intake** (leucine, isoleucine, valine) | ALC causes disproportionate BCAA depletion; BCAAs are metabolized by muscle, not liver |
| **Emphasize egg and dairy proteins** | Highest BCAA density; easy to prepare and eat |
| **Include plant proteins daily** | Legumes reduce ammonia generation vs. red meat |
| **Minimize red and processed meat** | High ammonia load; associated with hepatic encephalopathy |
| **Avoid protein restriction** even with elevated ammonia | Sarcopenia worsens HE outcomes paradoxically |

**Practical daily protein targets:**

- Breakfast: 20g protein (e.g., 2–3 scrambled eggs + milk)
- Lunch: 25–30g protein (e.g., fish fillet + lentils)
- Dinner: 25–30g protein (e.g., tofu/chicken + legume side)
- Snacks: 15–20g protein total across 2 snacks + LES

**3. Meal Frequency and Eating Pattern**

Patients with ALC frequently have **severely disrupted eating rhythms** from years of alcohol dependency — appetite suppression, meal skipping, and replacing food calories with alcohol are nearly universal. **Re-establishing a structured eating schedule is a core therapeutic intervention.**

**Recommended Daily Eating Schedule**

7:00 AM ── Breakfast (mandatory; never skip)

High protein + complex carb + micronutrient-rich

e.g., Oatmeal with milk + 2 eggs + orange juice (fortified)

10:30 AM ── Morning Snack

e.g., Banana + Greek yogurt / whole-grain toast + peanut butter

1:00 PM ── Lunch (largest meal of the day)

e.g., Salmon + brown rice + steamed broccoli + olive oil

4:30 PM ── Afternoon Snack

e.g., Handful of walnuts + low-fat cheese

7:00 PM ── Dinner

e.g., Tofu + lentil soup + whole grain bread + leafy greens

9:30 PM ── Late-Evening Snack ← MANDATORY

e.g., Warm oatmeal with low-fat milk / whole-grain crackers + cottage cheese

**Key Behavioral Principles**

- **4–6 structured meals per day** — re-trains appetite rhythm disrupted by alcohol use
- **Never fast > 3–4 hours** during waking hours
- **Late-Evening Snack (LES) is mandatory:** 150–200 kcal of carbohydrate + protein
  - Prevents overnight catabolism, which is especially severe in ALC due to depleted glycogen stores
  - Overnight fast must not exceed **≤ 12 hours**
- **Eat at a table, seated** — re-establishes healthy mealtime behavior patterns
- Ensure **adequate oral hydration** (2–2.5 L/day water) — alcohol is chronically dehydrating

**4. Specific Food Recommendations & Restrictions**

**Priority Foods for ALC Nutritional Rehabilitation**

**High-Quality Protein Sources**

| **Food** | **Benefit** | **Serving Suggestion** |
| --- | --- | --- |
| Eggs | Highest BCAA content; rich in choline (hepatoprotective) | 1–2 per meal, any preparation |
| Greek yogurt / cottage cheese | BCAA-rich; probiotic benefit | Snacks, breakfast, LES |
| Oily fish (salmon, mackerel, sardines) | Omega-3; B12; Vitamin D | 3–4 times/week |
| Tofu / tempeh / edamame | Plant BCAA; reduces ammonia; phytoestrogens | Daily or alternate days |
| Lentils / chickpeas / beans | Protein + fiber + folate | 4–5 times/week |
| Lean poultry | Easily digestible; low ammonia | 2–3 times/week |

**Carbohydrate & Energy Sources**

| **Food** | **Benefit** |
| --- | --- |
| Oats | Slow-release energy; beta-glucan; B vitamins |
| Brown rice / quinoa | Complex carb; magnesium; B vitamins |
| Sweet potato | Beta-carotene (pro-Vit A); potassium; fiber |
| Whole grain bread | Thiamine-fortified options available; fiber |
| Bananas | Potassium; rapid energy; easy to eat |

**Vegetables & Fruits (Anti-inflammatory, Antioxidant)**

| **Category** | **Recommendations** |
| --- | --- |
| **Dark leafy greens** | Spinach, kale, Swiss chard — folate, Vit K, magnesium |
| **Cruciferous** | Broccoli, cauliflower — sulforaphane supports hepatic detoxification |
| **Orange/yellow vegetables** | Carrots, pumpkin, sweet pepper — pro-Vitamin A (retinol precursor) |
| **Berries** | Blueberries, strawberries — polyphenols, antioxidants |
| **Citrus** | Oranges, grapefruit* — Vitamin C, folate |

*Grapefruit: check for drug interactions if patient is on medications metabolized by CYP3A4*

**Beverages**

| **Beverage** | **Recommendation** |
| --- | --- |
| **Coffee** (unsweetened) | 2–3 cups/day — strong evidence for anti-fibrotic, anti-HCC benefit in ALC |
| **Water** | Primary beverage; 2–2.5 L/day |
| **Low-fat milk / fortified plant milk** | Calcium, Vitamin D, protein |
| **Green tea** (moderate) | Antioxidant polyphenols |

**Foods and Substances to Avoid**

| **Item** | **Reason** | **Severity** |
| --- | --- | --- |
| **Alcohol (all forms)** | Absolute contraindication — drives fibrosis, mitochondrial toxicity, HCC | Critical |
| **Alcohol-containing condiments / cooking wines** | Hidden alcohol sources — read labels carefully | Critical |
| **Raw shellfish** (oysters, clams) | *Vibrio vulnificus* — potentially fatal in cirrhosis | Critical |
| **Herbal supplements / TCM preparations** | Many are hepatotoxic; dozens implicated in DILI | Avoid all |
| **High-sodium processed foods** | Prophylactic fluid/ascites prevention | Restrict |
| **Saturated/trans fats** | Promote hepatic steatosis, cardiovascular risk | Restrict |
| **High-fructose corn syrup / added sugars** | Hepatic lipogenesis; replace alcohol calories with nutrient-dense food | Restrict |
| **Red and processed meat** | Increased ammonia load; pro-inflammatory | Minimize |
| **Non-prescription NSAIDs** (ibuprofen, naproxen) | GI bleeding risk; nephrotoxic in cirrhosis | Avoid |
| **Acetaminophen (high-dose)** | Hepatotoxic in alcohol-related liver disease | Use with caution (max 2g/day if needed) |

**5. Additional Nutritional Considerations**

**Alcohol-Specific Micronutrient Deficiency Profile**

Chronic alcohol use causes a **distinctive and severe pattern of micronutrient depletion** through multiple mechanisms: reduced dietary intake, impaired intestinal absorption, increased urinary excretion, and impaired hepatic storage and activation.

| **Nutrient** | **Clinical Risk** | **Priority Action** |
| --- | --- | --- |
| **Thiamine (Vitamin B1)** | ⭐⭐⭐ CRITICAL — Wernicke's encephalopathy risk | **Oral thiamine 100 mg/day empirically — do not wait for blood levels** |
| **Folate (Vitamin B9)** | ⭐⭐⭐ Very common — megaloblastic anemia, elevated homocysteine | Supplement 1 mg/day; dietary emphasis on legumes, leafy greens |
| **Pyridoxine (Vitamin B6)** | ⭐⭐ Common — peripheral neuropathy, amino acid metabolism | B-complex supplement or B6 25–50 mg/day |
| **Vitamin B12** | ⭐⭐ May be falsely normal (released from damaged hepatocytes) | Monitor; dietary animal proteins |
| **Zinc** | ⭐⭐⭐ Severely depleted — impairs ammonia metabolism, immune function, taste | Supplement 30–45 mg elemental zinc/day |
| **Magnesium** | ⭐⭐⭐ Severely depleted — arrhythmia, muscle cramps, insulin resistance | Check serum Mg; supplement if low; dietary: nuts, legumes, greens |
| **Vitamin D** | ⭐⭐⭐ Almost universally deficient — bone disease, immune function | Check 25-OH Vit D; supplement 1,000–2,000 IU/day to achieve >30 ng/mL |
| **Vitamin A** | ⭐⭐ Depleted, but supplement cautiously | Dietary pro-Vit A (carotenoids) preferred; avoid high-dose retinol supplementation (hepatotoxic in ALD) |
| **Vitamin E** | ⭐⭐ Oxidative stress depletion | Dietary sources (olive oil, nuts, seeds); low-dose supplement if needed |
| **Vitamin K** | ⭐⭐ Reduced hepatic synthesis → coagulopathy | Dietary leafy greens; supplement if INR elevated |
| **Selenium** | ⭐ Depleted — antioxidant defense | Brazil nuts (1–2/day), fish, whole grains |
| **Phosphorus** | Monitor closely | See Refeeding Syndrome box below |
| **Potassium** | Risk if poor intake or vomiting | Bananas, potatoes, dairy, legumes |

**Refeeding Syndrome — A Specific Risk in ALC**

**Refeeding syndrome** occurs when a chronically malnourished/starved patient resumes eating — insulin surges drive phosphate, potassium, and magnesium into cells, causing life-threatening hypophosphatemia.

**This patient is at risk if:**

- Was consuming most calories as alcohol with minimal food intake
- Shows signs of significant malnutrition (muscle wasting despite BMI 20.8)
- Has been acutely ill or hospitalized recently

**Prevention protocol:**

Start nutritional rehabilitation gradually over 3–5 days

Check electrolytes (phosphate, potassium, magnesium) before refeeding

Supplement thiamine BEFORE refeeding carbohydrates

Monitor electrolytes daily for first week

If inpatient: start at 50% of target calories, escalate over 3–5 days

**BCAA Supplementation**

| **Indication** | **Recommendation** |
| --- | --- |
| If dietary protein target not achievable | Oral BCAA supplement 12–15 g/day |
| Timing | Distribute across meals; include in LES |
| Benefit | Reduces hepatic encephalopathy risk, improves nitrogen balance, supports muscle synthesis |
| Form | BCAA granules or powder mixed into food/drinks |

**Bone Health — ALC-Specific Concern**

Alcohol-related liver disease causes **hepatic osteodystrophy** through multiple pathways:

- Direct osteotoxicity of alcohol on osteoblasts
- Vitamin D deficiency and malabsorption
- Malnutrition-related calcium depletion
- Hypogonadism from alcohol (reduced testosterone → bone loss)

**Recommendations:**

- **Calcium:** 1,000–1,200 mg/day (dietary first: low-fat dairy, sardines, fortified plant milk, tofu)
- **Vitamin D:** Supplement as above
- **Baseline DEXA scan** strongly recommended
- **Testosterone level** check — hypogonadism is common in ALC males and independently worsens sarcopenia and bone density

**Neurological & Psychiatric Nutritional Support**

| **Concern** | **Nutritional Link** | **Action** |
| --- | --- | --- |
| **Wernicke's Encephalopathy** | Thiamine deficiency | Empiric oral thiamine — if acute neurological symptoms develop, IV thiamine urgently |
| **Peripheral neuropathy** | B1, B6, B12 deficiency | B-vitamin complex supplementation |
| **Depression / anxiety** (common in ALC) | Zinc, Omega-3, B12, folate deficiency all implicated | Nutritional repletion as adjunct to psychiatric care |
| **Alcohol cravings** | Zinc and glutamine depletion may exacerbate | Ensure zinc repletion; refer to addiction medicine |
| **Cognitive impairment** | Thiamine, B12 | Aggressive repletion; neurological assessment |

**Multidisciplinary Support — Essential in ALC**

Nutritional management of ALC **cannot succeed in isolation**. This patient requires coordinated care:

Hepatologist ──── Nutritionist/Dietitian ──── Addiction Medicine

│	│	│

Liver surveillance	Diet planning	Relapse prevention

Variceal screening	Micronutrient repletion Psychosocial support

Complication mgmt	Muscle monitoring	AA / counseling referral

│

Primary Care ─────────── Social Work

Metabolic monitoring	Housing/family support

**Alcohol abstinence support services** (Alcoholics Anonymous, motivational interviewing, pharmacotherapy with naltrexone or acamprosate if appropriate) are as important as nutritional intervention — they are complementary, not competing priorities.

**Monitoring & Follow-up Plan**

| **Parameter** | **Frequency** | **Rationale** |
| --- | --- | --- |
| Weight, BMI, hand-grip strength | Monthly | Early detection of sarcopenia/malnutrition |
| Alcohol use screening (AUDIT-C or CAGE) | Every visit | Relapse detection |
| LFTs, albumin, bilirubin, INR, CBC | Every 3 months | Disease progression, nutritional status |
| Thiamine, folate, B12, zinc, Vit D, Mg | Every 3–6 months initially, then annually if stable | Deficiency repletion monitoring |
| Phosphate, potassium (refeeding risk) | Weekly for first month if resuming nutrition after restriction | Refeeding syndrome surveillance |
| Bone density (DEXA) | Baseline, then every 2 years | Hepatic osteodystrophy |
| Testosterone level | Baseline | Hypogonadism assessment |
| HCC surveillance (ultrasound ± AFP) | Every 6 months | Mandatory in ALC cirrhosis |
| Upper endoscopy (varices) | Per hepatologist (typically every 2–3 years if no varices at baseline) | Portal hypertension surveillance |

**Summary for the Patient:** *"Your liver has been significantly affected by alcohol, and now that you have stopped drinking, good nutrition is one of the most powerful tools to help it recover. Eat 5–6 small meals a day — never skip breakfast and always have a small snack before bed. Focus on eggs, fish, tofu, lentils, yogurt, and plenty of vegetables. Your body is likely low in several vitamins — especially Vitamin B1, folate, and zinc — so your doctor will start you on supplements. Drink coffee and plenty of water. Never drink any alcohol again in any form, and check with your doctor before taking any supplements or herbal remedies."*

## C110

**Clinical Nutrition Consultation**

**Patient: Female, 66 y/o | HBV-Related Compensated Cirrhosis**

**BMI: 20.8 kg/m²** (Normal range) | **IBW: ~50 kg** | **Nutritional Risk: Moderate**

**1. Overall Dietary Principles**

| **Principle** | **Rationale** |
| --- | --- |
| **High-energy, nutrient-dense diet** | Prevent sarcopenia and malnutrition, common even in compensated cirrhosis |
| **Adequate protein — do NOT restrict** | Protein restriction is outdated and harmful; the liver needs amino acids for regeneration |
| **Small, frequent meals** | Reduces gluconeogenesis from muscle breakdown during fasting |
| **Mediterranean-style pattern** | Anti-inflammatory, hepatoprotective, reduces fibrosis progression |
| **Strict alcohol abstinence** | Absolute contraindication regardless of HBV etiology |

**Key principle:** This patient's BMI of 20.8 is at the lower end of normal for her age. At 66 years old with cirrhosis, she is at significant risk for **sarcopenic obesity** (muscle loss with preserved fat mass), which may not be apparent from weight alone.

**2. Recommended Daily Protein Intake**

**Target: 1.2 – 1.5 g/kg/day = 60 – 75 g protein/day**

*(EASL/ESPEN guidelines for cirrhosis without acute encephalopathy)*

| **Protein Source** | **Daily Target Example** |
| --- | --- |
| Lean poultry / fish | 1 palm-sized serving (×2/day) |
| Eggs | 1–2 whole eggs |
| Low-fat dairy / tofu | 1–2 servings |
| Legumes (beans, lentils) | ½ cup cooked |

- **Branch-Chain Amino Acid (BCAA)-rich foods** are preferred: eggs, fish, lean poultry, soybeans
- **Vegetable protein** (tofu, legumes) is encouraged — may reduce ammonia load compared to red meat
- **Do NOT restrict protein** unless overt hepatic encephalopathy (HE) is present — she is currently compensated

**3. Meal Frequency and Eating Pattern**

**4–6 small meals per day + mandatory late-evening snack**

7:00 AM	Breakfast	~400–450 kcal

10:00 AM Morning snack	~200 kcal

12:30 PM Lunch	~450–500 kcal

3:30 PM	Afternoon snack ~200 kcal

6:30 PM	Dinner	~400–450 kcal

9:00 PM	★ Late-night snack (LNS) ~200 kcal ← CRITICAL

**⭐ Why the Late-Night Snack (LNS) Matters**

Patients with cirrhosis have a **shortened overnight fasting tolerance** — they enter a catabolic starvation state after only **4–6 hours** (vs. 12+ hours in healthy adults). The LNS prevents overnight muscle catabolism and improves nitrogen balance.

**Ideal LNS options:**

- 200 mL warm soy milk + 1–2 whole-grain crackers
- Small bowl of congee with egg
- Branched-chain amino acid (BCAA) supplement if intake is poor

**4. Specific Food Recommendations and Restrictions**

**Recommended Foods**

| **Category** | **Examples** | **Benefit** |
| --- | --- | --- |
| **Lean protein** | Fish (salmon, cod), chicken breast, tofu, eggs | BCAA-rich, easy to digest |
| **Complex carbohydrates** | Brown rice, oats, sweet potato, whole grain bread | Steady glucose, fiber |
| **Vegetables** | Broccoli, spinach, carrots, cucumber | Antioxidants, folate |
| **Fruits** | Banana, papaya, apple (moderate portions) | Potassium, vitamins |
| **Dairy** | Low-fat yogurt, warm milk | Calcium, protein |
| **Healthy fats** | Olive oil, small amounts of nuts/avocado | Anti-inflammatory |

**Foods to Restrict or Avoid**

| **Category** | **Reason** |
| --- | --- |
| **Alcohol** (any amount) | Absolute contraindication — accelerates cirrhosis progression |
| **Raw shellfish / raw fish (sushi/sashimi)** | High risk of *Vibrio vulnificus* infection — potentially fatal in cirrhosis |
| **Highly processed foods, cured meats** | High sodium → risk of ascites/fluid retention |
| **High-sodium foods (>2g Na/day)** | Even in compensated disease, limit sodium to prevent decompensation |
| **High-fructose corn syrup, sugary drinks** | Promotes hepatic steatosis, worsens liver injury |
| **Excessive red/processed meat** | High ammonia precursors; pro-inflammatory |
| **Herbal supplements, traditional remedies** | Many are **hepatotoxic** (e.g., kava, comfrey, certain TCM herbs) — always disclose to physician |

**5. Additional Nutritional Considerations**

**Total Daily Energy**

**Target: 30–35 kcal/kg/day → ~1,500 – 1,750 kcal/day** *(Upper range preferred given her age and risk of sarcopenia)*

**Micronutrient Monitoring**

Chronic HBV cirrhosis is commonly associated with the following deficiencies — consider monitoring and supplementation:

| **Nutrient** | **Clinical Concern** | **Action** |
| --- | --- | --- |
| **Zinc** | Often depleted in cirrhosis; supports ammonia metabolism | Supplement if low (zinc sulfate 50mg/day) |
| **Vitamin D** | Frequently low; important for bone and immune health | Supplement + sun exposure; check 25-OH-VitD level |
| **B vitamins (B1, B6, B12, Folate)** | Impaired hepatic storage and activation | Multivitamin or targeted supplementation |
| **Calcium** | Risk of osteoporosis with cirrhosis + age 66 | 1,000–1,200 mg/day from diet/supplements |
| **Iron** | Avoid excess supplementation in HBV — may accelerate fibrosis and oxidative stress | Check ferritin; supplement only if truly deficient |

**Hydration**

- Target **1.5–2 L water/day** unless ascites develops (at which point sodium and fluid restriction may be needed)
- Avoid caffeinated drinks as primary fluid source

**Bone Health**

- Age 66 + cirrhosis = elevated risk of **hepatic osteodystrophy** (osteoporosis)
- Encourage **weight-bearing exercise** (walking 20–30 min/day if tolerated)
- Consider DEXA scan if not recently performed

**Monitoring & Follow-Up**

- Reassess nutritional status every **3–6 months**
- Use **hand-grip strength** and/or **mid-arm muscle circumference (MAMC)** to monitor for sarcopenia — these are more sensitive than weight alone in cirrhosis
- If oral intake is persistently insufficient, consider **BCAA oral supplements** (e.g., BCAA granules 4g × 3/day), which are proven to improve albumin levels and quality of life in cirrhosis

**Disclaimer:** These are evidence-based general recommendations for clinical guidance purposes. Individual management should be tailored by the treating physician and registered dietitian based on laboratory values, current medications, and clinical progression. This does not constitute a patient-specific medical prescription.

## C111

**Dietary & Nutritional Recommendations**

**Patient:** Male, 70 y/o | 171 cm | 76 kg | BMI ~26.0 kg/m² **Diagnosis:** MASLD-related compensated cirrhosis

**1. Overall Dietary Principles**

This patient presents a nuanced challenge: **compensated cirrhosis requiring adequate nutrition** combined with **MASLD requiring metabolic risk reduction**, in the context of an elderly patient with mildly elevated BMI.

- **Avoid both undernutrition and overnutrition.** Sarcopenia is highly prevalent in cirrhosis and worsens prognosis — aggressive caloric restriction is contraindicated.
- **Modest caloric target:** ~**28–35 kcal/kg/day** of actual body weight → approximately **2,100–2,650 kcal/day**, tailored to activity level. Given MASLD context, aim toward the lower end if sedentary.
- **Prioritize metabolic improvement:** reduce refined carbohydrates, saturated fats, and ultra-processed foods — the drivers of MASLD progression.
- **Mediterranean dietary pattern** is the preferred framework: anti-inflammatory, hepatoprotective, and metabolically favorable.
- **Avoid alcohol entirely** — no safe threshold in cirrhosis.

**2. Recommended Daily Protein Intake**

| **Principle** | **Detail** |
| --- | --- |
| **Target** | **1.2–1.5 g/kg/day** of actual body weight |
| **Daily amount** | ≈ **91–114 g protein/day** |
| **Rationale** | Elderly patients have anabolic resistance; cirrhosis accelerates muscle catabolism. Higher protein intake preserves muscle mass without precipitating hepatic encephalopathy in compensated disease. |
| **Protein restriction** | **Not recommended** — outdated practice. Restriction worsens sarcopenia and outcomes. |
| **Preferred sources** | Plant proteins (legumes, soy), fish, poultry, low-fat dairy, eggs |

**MASLD note:** Limit red and processed meat due to associations with metabolic dysfunction and colorectal risk.

**3. Meal Frequency and Eating Pattern**

**Cirrhotic patients have accelerated starvation metabolism** — even brief fasting can trigger gluconeogenesis from muscle. This is especially critical in elderly patients.

- **4–6 small meals per day**, evenly spaced (every 3–4 hours)
- **Late-evening snack (LES) — mandatory:** consume a complex carbohydrate + protein snack **within 1–2 hours of bedtime**
  - Examples: whole-grain crackers with peanut butter, low-fat Greek yogurt with oats, a small bowl of lentil soup
  - Provides ~200 kcal; significantly reduces overnight protein catabolism
- **Do not skip breakfast** — aim for a protein-rich morning meal
- Avoid prolonged fasting periods >4–5 hours during the day

**4. Food Recommendations and Restrictions**

**Encouraged Foods**

| **Category** | **Examples** |
| --- | --- |
| **Vegetables** | All non-starchy vegetables: leafy greens, broccoli, cauliflower, tomatoes, zucchini |
| **Fruits** | Berries, apples, pears, citrus (moderate portions due to fructose) |
| **Whole grains** | Oats, barley, quinoa, whole wheat bread — high fiber, low glycemic index |
| **Legumes** | Lentils, chickpeas, beans — excellent plant protein + fiber |
| **Fish** | Fatty fish 2–3×/week (salmon, mackerel, sardines) — anti-inflammatory omega-3s |
| **Lean protein** | Chicken breast, turkey, eggs, low-fat dairy (yogurt, cottage cheese) |
| **Healthy fats** | Extra virgin olive oil (primary fat), nuts, avocado |
| **Coffee** | 2–3 cups/day (unsweetened) — hepatoprotective evidence in MASLD/cirrhosis |

**Foods to Restrict or Avoid**

| **Category** | **Reason** |
| --- | --- |
| **Alcohol** | Absolute contraindication |
| **Added sugars / fructose** | Drives hepatic steatosis, metabolic syndrome; avoid soft drinks, fruit juices, sweets |
| **Refined carbohydrates** | White bread, white rice, pastries — worsen insulin resistance |
| **Saturated/trans fats** | Red meat, processed meat, butter, fried foods — promote steatosis and inflammation |
| **Ultra-processed foods** | Chips, fast food, packaged snacks — high sodium, additives, unhealthy fats |
| **High-sodium foods** | Even without overt ascites: limit sodium to **<2,000–2,400 mg/day**; avoid canned soups, deli meats, pickled foods |
| **Raw shellfish** | Risk of *Vibrio vulnificus* — potentially fatal in cirrhosis |

**5. Additional Nutritional Considerations**

**Micronutrient Monitoring**

| **Nutrient** | **Concern** | **Action** |
| --- | --- | --- |
| **Zinc** | Frequently deficient in cirrhosis; supports ammonia metabolism | Consider supplementation 25–50 mg/day if deficient; zinc-rich foods (pumpkin seeds, legumes) |
| **Vitamin D** | Very common deficiency in MASLD + cirrhosis + elderly | Check 25(OH)D; supplement if <30 ng/mL (typically 1,000–2,000 IU/day) |
| **B vitamins (B1, B6, B12, Folate)** | Impaired hepatic storage and metabolism | Multivitamin or B-complex may be appropriate |
| **Magnesium** | Often low in liver disease | Monitor; found in nuts, seeds, leafy greens |
| **Iron** | MASLD may involve elevated ferritin — avoid supplementation unless confirmed deficient | Do not supplement empirically |

**Weight Management**

- Modest weight loss of **5–10% body weight** can improve liver histology in MASLD, but must be **slow and gradual** (0.5–1 kg/week maximum)
- Avoid crash diets or very low-calorie diets — risk of precipitating hepatic decompensation

**Physical Activity**

- Encourage **moderate aerobic exercise** (e.g., 30 min brisk walking, 5 days/week) + **resistance training** 2×/week
- Improves insulin sensitivity, reduces hepatic steatosis, combats sarcopenia
- Adjust based on tolerance and absence of complications (e.g., varices, ascites)

**Glycemic and Lipid Control**

- Screen for **diabetes/prediabetes** (common in MASLD) — dietary management as above; coordinate with primary care
- Dyslipidemia management through diet: reduce saturated fat, increase soluble fiber (oats, psyllium, legumes)

**Monitoring**

- Regular nutritional assessment every 3–6 months: body weight, hand-grip strength, mid-arm muscle circumference
- Consider referral to a **registered dietitian (RD)** for individualized meal planning

**Summary:** This patient's dietary strategy prioritizes **muscle preservation** through adequate protein and frequent meals, **metabolic improvement** through a Mediterranean-style, low-sugar, anti-inflammatory diet, and **liver protection** through alcohol abstinence and micronutrient optimization — all while avoiding the twin risks of sarcopenia and progressive steatohepatitis.

## C112

**Dietary & Nutritional Recommendations**

**Patient:** Female, 73 y/o | 159 cm | 66 kg | BMI: 26.1 kg/m² **Diagnosis:** Alcohol-related compensated cirrhosis

**1. Overall Dietary Principles**

- **Absolute alcohol abstinence** is the single most critical intervention — continued drinking accelerates fibrosis progression even in compensated disease
- Aim for a **balanced, anti-inflammatory diet** with adequate energy intake: approximately **30–35 kcal/kg/day** (~1,980–2,310 kcal/day)
- Avoid prolonged fasting; the liver's glycogen reserve is impaired in cirrhosis, making overnight fasting metabolically stressful
- Maintain current weight or achieve modest reduction if metabolic syndrome features are present; **aggressive caloric restriction is discouraged**
- Prioritize **nutrient-dense whole foods** over processed or refined products

**2. Recommended Daily Protein Intake**

| **Parameter** | **Recommendation** |
| --- | --- |
| **Target intake** | **1.2–1.5 g/kg/day** → ~79–99 g protein/day |
| **Rationale** | Older age increases sarcopenia risk; cirrhosis elevates protein catabolism |
| **Protein restriction?** | **No** — outdated practice; restriction worsens outcomes |
| **Preferred sources** | Plant proteins + lean animal proteins (see Section 4) |

**Note on hepatic encephalopathy (HE):** This patient is currently compensated with no HE. Protein restriction is **not indicated**. If overt HE develops in the future, **do not restrict protein** — instead, favor branched-chain amino acid (BCAA) supplementation and plant/dairy protein sources.

**3. Meal Frequency and Eating Pattern**

Cirrhosis impairs gluconeogenesis and glycogen storage, mimicking a **prolonged fasting state** even after short gaps between meals.

- **4–6 small meals per day**, evenly distributed
- **Mandatory late-evening snack (LES):** ~200–300 kcal, ideally complex carbohydrates + protein (e.g., whole-grain crackers with cheese, or a small bowl of oatmeal with milk) — this is evidence-based and reduces overnight protein catabolism
- **Do not skip breakfast** — the overnight fast should be broken as early as possible
- Suggested distribution example:

Breakfast (8am) → Mid-morning snack (10:30am) → Lunch (1pm)

→ Afternoon snack (3:30pm) → Dinner (6:30pm) → LES (9–10pm)

**4. Specific Food Recommendations & Restrictions**

**Recommended Foods**

| **Category** | **Examples** |
| --- | --- |
| **Lean protein** | Eggs, poultry (skinless), fish (especially oily fish 2×/week), low-fat dairy |
| **Plant protein** | Lentils, chickpeas, tofu, edamame (also BCAA-rich) |
| **Complex carbohydrates** | Oats, brown rice, sweet potato, whole grain bread |
| **Healthy fats** | Olive oil, avocado, nuts and seeds (in moderation) |
| **Vegetables** | Leafy greens, broccoli, carrots, cooked vegetables (easier to digest) |
| **Fruits** | Berries, apples, pears (moderate portions to manage sugar load) |

**Foods to Restrict or Avoid**

| **Item** | **Reason** |
| --- | --- |
| **All alcohol** | Direct hepatotoxin; #1 priority to eliminate |
| **High-sodium foods** (processed meats, canned soups, fast food) | Risk of ascites; target **<2,000 mg sodium/day** even in compensated stage as preventive measure |
| **Raw shellfish** (oysters, clams) | Risk of *Vibrio vulnificus* — potentially fatal in cirrhosis |
| **Raw/undercooked eggs, unpasteurized products** | Infection risk due to impaired immune function |
| **High-fructose corn syrup, sugary beverages** | Worsens hepatic steatosis and metabolic burden |
| **Excessive red/processed meat** | Pro-inflammatory; increases ammonia load |
| **Grapefruit** | Inhibits CYP3A4 — may interact with medications |

**5. Additional Nutritional Considerations**

**Micronutrient Repletion**

Alcohol-related liver disease commonly causes deficiencies that require attention:

| **Nutrient** | **Action** |
| --- | --- |
| **Thiamine (B1)** | Supplement routinely (100 mg/day orally) — Wernicke's risk |
| **Folate (B9)** | Supplement 1 mg/day; alcohol depletes folate |
| **Vitamin B6, B12** | Check levels; supplement if deficient |
| **Zinc** | Deficiency is common in cirrhosis; 25–45 mg/day elemental zinc if low (also helps with HE prevention) |
| **Vitamin D** | Check 25-OH vitamin D; supplement if <30 ng/mL (cholecalciferol 1,000–2,000 IU/day) |
| **Magnesium** | Monitor; often depleted in alcohol use disorder |
| **Fat-soluble vitamins (A, E, K)** | Assess levels; supplement cautiously (avoid excess vitamin A — hepatotoxic) |

**Bone Health**

- At 73 years old with alcohol-related liver disease, **osteoporosis risk is significantly elevated**
- Ensure adequate **calcium (1,200 mg/day from diet/supplements)** and **vitamin D**
- Consider DEXA scan referral

**Sarcopenia Prevention**

- BMI 26.1 may mask **sarcopenic obesity** (low muscle mass with preserved/elevated fat mass) — consider hand-grip strength or body composition assessment
- **Resistance exercise** (light weights, resistance bands) in combination with adequate protein is strongly recommended
- BCAA supplementation (e.g., 12–15 g/day) may be considered if dietary protein targets are not achievable

**Medication Interactions**

- If on **lactulose or rifaximin** (for HE prophylaxis): maintain adequate fluid and fiber intake
- If on **diuretics** (e.g., spironolactone): monitor potassium-rich foods (bananas, oranges, potatoes) — may need moderation
- If on **warfarin**: maintain **consistent** vitamin K intake (do not eliminate green vegetables — just keep intake stable day-to-day)

**Psychosocial Support**

- Given alcohol-related etiology, referral to **addiction counseling / alcohol use disorder treatment** is integral to nutritional success
- Social eating support and structured meal planning may improve adherence in an elderly patient living alone

**Summary Priority List:**

1. Complete alcohol cessation
2. Late-evening snack every night
3. Meet protein targets (1.2–1.5 g/kg/day)
4. Thiamine + folate supplementation
5. Moderate sodium restriction (<2,000 mg/day)
6. Avoid raw shellfish absolutely

*Reassess nutritional status (including albumin, pre-albumin, hand-grip strength, and anthropometrics) every 3–6 months.*

## C201

**Dietary & Nutritional Recommendations**

**Patient:** Male, 33 y/o | 173 cm, 69 kg | BMI 23.1 kg/m² **Diagnosis:** HBV-related cirrhosis with ascites (decompensated)

**Clinical Note:** Despite a normal BMI, ascites may be masking underlying sarcopenia or protein-energy malnutrition. Dry weight (post-diuresis) should be used for nutritional calculations when possible.

**1. Overall Dietary Principles**

| **Principle** | **Rationale** |
| --- | --- |
| **High-energy, nutrient-dense diet** | Hypermetabolic state common in cirrhosis; prevent catabolism |
| **Do NOT restrict protein** | Old practice; harmful in modern management |
| **Sodium restriction** | Critical for ascites management |
| **Small, frequent meals** | Reduces early satiety from ascites; minimizes overnight fasting |
| **Avoid alcohol absolutely** | Zero tolerance, even with HBV etiology |

**2. Daily Protein Intake**

**Target: 1.2 – 1.5 g/kg of dry body weight/day**

Estimated dry body weight: **~64–66 kg** (assuming ~3–5 kg of ascitic fluid contribution)

| **Calculation** | **Amount** |
| --- | --- |
| Conservative target (1.2 g/kg × 65 kg) | **~78 g protein/day** |
| Optimal target (1.5 g/kg × 65 kg) | **~98 g protein/day** |
| **Practical recommendation** | **≥ 80–100 g/day** |

- **Plant-based proteins preferred** (BCAA-rich, less ammoniagenic): tofu, lentils, edamame, chickpeas
- **Whey protein or BCAA supplements** if oral intake is inadequate
- **Fish, eggs, and lean poultry** are acceptable
- **No need to restrict protein** unless overt hepatic encephalopathy (HE) is present — this patient has none documented

**3. Meal Frequency & Eating Pattern**

Recommended: 4–6 small meals per day + mandatory late-evening snack (LES)

| **Time** | **Meal** |
| --- | --- |
| ~7:00 AM | Breakfast |
| ~10:00 AM | Mid-morning snack |
| ~12:30 PM | Lunch |
| ~3:30 PM | Afternoon snack |
| ~6:30 PM | Dinner |
| **~9:00–10:00 PM** | **Late-evening snack (LES) ← Critical** |

**Why the Late-Evening Snack Matters**

Cirrhotic patients exhaust glycogen stores in only **~10 hours of fasting** (vs. ~24 hours in healthy individuals). Overnight fasting triggers gluconeogenesis from muscle protein → accelerates sarcopenia.

**Ideal LES composition:**

- 200–400 kcal
- 15–20 g of complex carbohydrate + protein
- Examples: whole grain bread with peanut butter, banana with Greek yogurt, rice crackers with cottage cheese, or a BCAA-enriched supplement drink

**4. Food Recommendations & Restrictions**

**Recommended Foods**

| **Category** | **Examples** |
| --- | --- |
| **Protein sources** | Tofu, tempeh, eggs, fish (salmon, mackerel), skinless chicken, lentils, chickpeas |
| **Carbohydrates** | Brown rice, oats, sweet potato, whole grain bread (low-sodium) |
| **Fats (healthy)** | Olive oil, avocado, nuts (unsalted), flaxseed |
| **Vegetables** | All varieties — steamed or fresh; avoid pickled/canned |
| **Fruits** | Banana, apple, papaya (potassium-rich if on diuretics, monitor levels) |

**Foods to Restrict or Avoid**

| **Item** | **Reason** |
| --- | --- |
| **Salt / sodium > 2,000 mg/day** | Worsens ascites and fluid retention |
| **Processed/canned foods** | High hidden sodium content |
| **Alcohol** | Absolute contraindication |
| **Raw shellfish (oysters, clams)** | Risk of *Vibrio vulnificus* — potentially fatal in cirrhosis |
| **Raw/undercooked meat or eggs** | Immunocompromised state increases infection risk |
| **High-fructose corn syrup, sugary drinks** | Risk of metabolic stress and hepatic fat |
| **Herbal supplements** | Many are hepatotoxic (e.g., kava, comfrey, green tea extract) |

**Sodium Restriction Guidance**

- **Target: < 2,000 mg sodium/day** (≈ 5 g table salt)
- Teach patient to read food labels
- Use herbs, lemon juice, vinegar, garlic as flavor alternatives
- Avoid soy sauce, fish sauce, MSG-heavy seasonings

**5. Additional Nutritional Considerations**

**Micronutrients — Commonly Deficient in Cirrhosis**

| **Nutrient** | **Recommendation** |
| --- | --- |
| **Zinc** | Often depleted; consider 25–45 mg/day elemental zinc; supports ammonia metabolism |
| **Vitamin D** | Check serum 25-OH-D; supplement 1,000–2,000 IU/day if deficient (common) |
| **B vitamins (B1, B6, B12, folate)** | Supplement B-complex; B1 (thiamine) especially important |
| **Magnesium** | Monitor if on loop diuretics/spironolactone |
| **Potassium** | Monitor carefully — diuretics cause loss; replace via diet (banana, potato) or supplement |
| **Vitamin K** | May be deficient; affects coagulation (discuss with hepatologist before supplementing) |

**Muscle Mass & Sarcopenia Prevention**

- Sarcopenia affects **>50% of cirrhotic patients** and is a major predictor of poor outcomes
- Encourage **light resistance exercise** if tolerated (walking, resistance bands)
- Consider **BCAA supplementation** (leucine-enriched) if dietary protein targets are not being met — evidence supports improvement in muscle mass and HE prevention

**Fluid Management**

- **Do not over-restrict fluids** unless hyponatremia is present (Na⁺ < 125–130 mEq/L)
- If hyponatremia develops: restrict to **1,000–1,500 mL fluid/day**
- Coordinate with hepatologist regarding diuretic regimen (spironolactone ± furosemide)

**HBV-Specific Considerations**

- Confirm patient is on appropriate **antiviral therapy** (tenofovir or entecavir) — this directly impacts liver disease progression and nutritional outcomes
- Avoid hepatotoxic supplements and over-the-counter NSAIDs

**Monitoring Plan**

| **Parameter** | **Frequency** |
| --- | --- |
| Weight & abdominal girth | Weekly (at home) |
| Serum electrolytes, albumin, renal function | Every 4–8 weeks |
| Nutritional assessment (MNA or RFH-NPT score) | Each clinic visit |
| Hand-grip strength (sarcopenia screening) | Every 3–6 months |

**Summary Goal:** Achieve ≥80–100 g protein/day · <2,000 mg sodium/day · 4–6 meals/day including a late-evening snack · supplementation of zinc, Vitamin D, and B-complex · absolute alcohol avoidance · regular monitoring of electrolytes and muscle mass.

*These recommendations align with current EASL (2018) and ESPEN (2019) guidelines on nutrition in chronic liver disease.*

## C202

**Dietary & Nutritional Recommendations**

**Patient:** Female, 37 y/o | 159 cm / 56 kg | BMI: 22.2 kg/m² **Diagnosis:** MASLD Cirrhosis with Ascites & Decreased Appetite

**Clinical Note:** BMI appears normal, but ascites causes fluid-related weight overestimation. True lean body mass may reflect early protein-energy malnutrition — assess with hand-grip strength or mid-arm muscle circumference.

**1. Overall Dietary Principles**

| **Priority** | **Rationale** |
| --- | --- |
| **High-calorie, high-protein diet** | Prevent/reverse sarcopenia; support hepatic regeneration |
| **Low sodium** | Manage ascites and fluid retention |
| **Anti-inflammatory, low saturated fat** | Address underlying MASLD pathophysiology |
| **Small, frequent meals** | Overcome reduced appetite and early satiety from ascites |
| **Avoid fasting periods >4–6 hours** | Prevent accelerated gluconeogenesis from muscle catabolism |

**2. Recommended Daily Protein Intake**

**Target: 1.2 – 1.5 g/kg ideal body weight/day**

Estimated ideal body weight ≈ **54–57 kg** (consistent with actual weight after correcting for ascites) → **~67–85 g protein/day**

- **Protein restriction is NOT recommended** — even in patients with prior hepatic encephalopathy, unless actively symptomatic
- Prefer **branched-chain amino acid (BCAA)-rich** sources and **plant-based proteins** to reduce ammonia load
- If oral intake is consistently insufficient, consider **BCAA oral supplementation** (e.g., 12 g/day in divided doses)

**3. Meal Frequency & Eating Pattern**

Given her **ascites and poor appetite**, the following pattern is strongly recommended:

Breakfast	— Light but protein-containing (e.g., egg + oats)

Mid-morning snack — Small; fruit + nuts or yogurt

Lunch	— Main meal of the day

Afternoon snack	— Protein-rich (e.g., cheese, tofu, legume dip)

Dinner	— Moderate; avoid large volumes

Late-night snack (LNS) ← CRITICAL

**⭐ Late-Night Snack (LNS) — Key Recommendation**

Consuming **~200 kcal of complex carbohydrates ± protein** before bed (e.g., whole-grain crackers with peanut butter, or a small bowl of oatmeal):

- Shortens the overnight fasting window
- Significantly reduces protein catabolism in cirrhotic patients
- Supported by EASL and JSH guidelines

**Total daily energy target: ~30–35 kcal/kg = ~1,620–1,890 kcal/day** *(adjust upward if malnutrition confirmed)*

**4. Specific Food Recommendations & Restrictions**

**Recommended Foods**

| **Category** | **Examples** |
| --- | --- |
| **Lean proteins** | Egg whites, steamed fish, skinless poultry, tofu, tempeh |
| **Plant proteins** | Lentils, chickpeas, edamame (also provide fiber) |
| **Complex carbohydrates** | Oats, brown rice, sweet potato, whole-grain bread |
| **Healthy fats** | Olive oil (in moderation), avocado, walnuts, flaxseed |
| **Vegetables** | Leafy greens, broccoli, carrots — steamed or cooked |
| **Fruits** | Berries, papaya, apples (watch potassium if hyperkalemic) |

**Foods to Restrict or Avoid**

| **Item** | **Reason** |
| --- | --- |
| **High-sodium foods** (processed meats, canned soups, soy sauce, pickles) | Worsens ascites and fluid retention |
| **All alcohol** | Absolute contraindication — accelerates fibrosis |
| **Saturated/trans fats** (fried food, fast food, full-fat dairy) | Drives MASLD progression |
| **Added sugars & refined carbs** (soda, white bread, pastries) | Worsens insulin resistance central to MASLD |
| **Red/processed meats in excess** | High ammonia precursor load |
| **Herbal supplements** | Many are hepatotoxic (e.g., kava, comfrey, green tea extract) |

**Sodium Target**

**< 2,000 mg sodium/day** (approximately 5 g table salt)

- Teach patient to read food labels
- Use herbs, lemon juice, vinegar for flavoring instead

**5. Additional Nutritional Considerations**

**Micronutrient Monitoring & Supplementation**

| **Nutrient** | **Consideration** |
| --- | --- |
| **Zinc** | Commonly deficient in cirrhosis; supports appetite and ammonia metabolism → consider 25–50 mg/day |
| **Vitamin D** | Frequently low in MASLD cirrhosis; check serum 25-OH-D → supplement if deficient |
| **Thiamine (B1)** | Risk of deficiency even without alcohol history in advanced liver disease |
| **Folate & B12** | Monitor; supplement if deficient |
| **Magnesium** | May be low, especially if on diuretics (spironolactone/furosemide) |
| **Fat-soluble vitamins (A, E, K)** | Malabsorption possible; assess clinically |
| **Iron** | Do NOT supplement empirically — iron overload worsens MASLD/fibrosis |

**Appetite Management Strategies**

Since she presents with **decreased appetite**:

- Offer **nutrient-dense, small-volume foods** over bulky low-calorie items
- Consider **liquid nutritional supplements** (e.g., hepatic-specific formulas like Fresubin Hepa®) if oral food intake < 70% of target
- Serve meals at **preferred temperature and texture** to maximize compliance
- Reassess appetite at every visit — early satiety may improve with better ascites control

**Weight & Fluid Monitoring**

- **Daily morning weight** at home (after voiding, before eating) to track fluid shifts
- Report weight gain **> 1 kg/day or > 2 kg/week** to care team (likely fluid accumulation)
- Fluid restriction is generally **not required** unless serum Na⁺ < 125 mmol/L

**Coordination with Medical Team**

- Ensure diuretic therapy (if prescribed) is optimized — ascites control will directly improve appetite and dietary tolerance
- Refer to a **registered dietitian** for ongoing individualized monitoring
- Screen for **sarcopenic obesity** or frailty using functional measures at each visit

**Summary Goal:** Maximize nutrient density within a sodium-restricted, small-volume eating pattern — prioritizing protein adequacy, a late-night snack, and MASLD-appropriate fat/carbohydrate quality to support both liver health and quality of life.

## C203

**Clinical Nutrition Recommendations**

**Patient:** Male, 41 y/o | 168 cm / 84 kg | BMI ~29.8* **Diagnosis:** Alcohol-related cirrhosis with moderate ascites

**BMI is likely overestimated due to fluid retention from ascites. True nutritional status must be assessed clinically (e.g., mid-arm muscle circumference, handgrip strength). Malnutrition may be masked.*

**1. Overall Dietary Principles**

| **Priority** | **Rationale** |
| --- | --- |
| **Strict alcohol abstinence** | Continued alcohol use directly accelerates hepatic fibrosis and worsens prognosis |
| **Prevent/treat malnutrition** | Sarcopenic malnutrition is present in up to 70% of cirrhotic patients regardless of apparent body weight |
| **Sodium restriction** | Essential for ascites management |
| **Avoid prolonged fasting** | Prevents hypoglycemia and accelerated protein catabolism due to impaired hepatic glycogen storage |
| **Caloric adequacy** | Supports liver regeneration and prevents muscle wasting |

**Target Energy Intake:**

- **35–40 kcal/kg dry body weight/day**
- Estimated dry weight: ~76–78 kg (subtracting ~6–8 kg ascitic fluid)
- → Approx. **2,660–3,120 kcal/day**

**2. Daily Protein Intake**

**Recommended: 1.2–1.5 g/kg dry body weight/day** → Approximately **90–115 g protein/day**

**Protein restriction is NOT recommended** — even in patients at risk for hepatic encephalopathy (HE). Current evidence shows protein restriction worsens sarcopenia and outcomes.

**Protein Source Prioritization:**

| **Preferred Sources** | **Rationale** |
| --- | --- |
| Vegetable proteins (legumes, soy) | Higher fiber content, favorable ammonia metabolism |
| Dairy proteins (Greek yogurt, cottage cheese) | Well-tolerated, good BCAA content |
| Lean poultry, fish, eggs | High biological value, lower in saturated fat |
| Branched-Chain Amino Acid (BCAA) supplements | Consider if HE develops or oral intake is insufficient |

**3. Meal Frequency and Eating Pattern**

Cirrhosis impairs glycogen storage, causing a fasting state after only **~4–6 hours** (equivalent to a healthy person fasting overnight for 24+ hours).

**Recommended Schedule:**

Breakfast	07:00 – 08:00	~600–700 kcal

Mid-morning	10:00 – 10:30	~300 kcal

Lunch	12:30 – 13:00	~700–800 kcal

Afternoon snack 15:30 – 16:00	~300 kcal

Dinner	18:30 – 19:00	~600–700 kcal

Late-night snack 21:00 – 22:00 ~200–300 kcal ← CRITICAL

**The late-night snack is essential.** A carbohydrate + protein snack before bed (e.g., whole grain toast with peanut butter, or a small bowl of oatmeal with milk) significantly reduces overnight gluconeogenesis from muscle protein.

**4. Food Recommendations and Restrictions**

**Recommended Foods**

- **Complex carbohydrates:** oats, whole grain bread, brown rice, sweet potato
- **Lean proteins:** eggs, tofu, lentils, chickpeas, low-fat dairy, fish, skinless chicken
- **Vegetables:** cooked or steamed (easier digestion); high in folate (spinach, broccoli)
- **Healthy fats (moderate):** olive oil, avocado — supports fat-soluble vitamin absorption
- **Probiotic foods:** yogurt, kefir — may modulate gut-liver axis

**Foods and Substances to Avoid**

| **Item** | **Reason** |
| --- | --- |
| **All alcohol** | Directly hepatotoxic |
| **High-sodium foods** | Worsens ascites (cured meats, canned soups, fast food, pickles, soy sauce) |
| **Raw shellfish/oysters** | Risk of *Vibrio vulnificus* — potentially fatal in cirrhosis |
| **Herbal supplements** | Many are hepatotoxic (e.g., kava, comfrey, green tea extract) |
| **High-fructose/ultra-processed foods** | Worsen liver fat and metabolic burden |
| **Excess red/processed meat** | Increases ammonia load; limit to occasional use |

**Sodium Target**

**< 2,000 mg sodium/day (≈ 5 g table salt)**

- Do not add salt at the table
- Use herbs, lemon, garlic as flavor alternatives
- Read food labels carefully

**5. Additional Nutritional Considerations**

**Micronutrient Repletion (alcohol-related cirrhosis commonly causes deficiencies)**

| **Nutrient** | **Clinical Concern** | **Action** |
| --- | --- | --- |
| **Thiamine (B1)** | Wernicke's encephalopathy risk | Supplement routinely: 100 mg/day orally |
| **Folate (B9)** | Deficient due to poor intake + alcohol | 1 mg/day supplement |
| **Zinc** | Commonly low; supports ammonia metabolism | 25–50 mg elemental zinc/day |
| **Vitamin D** | Impaired hepatic activation, low sun exposure | Check 25-OH Vit D; supplement 1,000–2,000 IU/day |
| **Magnesium** | Depleted by alcohol and diuretics | Monitor serum levels; dietary or supplement repletion |
| **Fat-soluble vitamins (A, E, K)** | Impaired bile production may reduce absorption | Assess levels; supplement if deficient |

**Fluid Management**

- If on **diuretics (spironolactone ± furosemide):** Monitor electrolytes closely
- **Fluid restriction** is generally only needed if **serum sodium < 125 mEq/L** (dilutional hyponatremia)
- Otherwise, fluids are not routinely restricted — but avoid excessive intake

**Hepatic Encephalopathy Prevention**

- Maintain **regular bowel movements** (avoid constipation): fiber-rich diet, lactulose if prescribed
- Adequate zinc intake supports urea cycle function
- **BCAA supplementation** (e.g., 12–15 g/day) may be beneficial if HE episodes occur

**Physical Activity**

- Encourage **light-to-moderate exercise** (walking 20–30 min/day) to preserve muscle mass and improve insulin sensitivity
- Avoid strenuous activity with significant ascites

**Summary Card**

| **Parameter** | **Target** |
| --- | --- |
| Calories | ~35–40 kcal/kg dry wt/day (~2,700–3,100 kcal) |
| Protein | 1.2–1.5 g/kg dry wt/day (~90–115 g) |
| Sodium | < 2,000 mg/day |
| Meals | 5–6 times/day including **late-night snack** |
| Alcohol | **Zero tolerance** |
| Key supplements | Thiamine, Folate, Zinc, Vitamin D |

**Follow-up Recommendation:** Reassess nutritional status every 4–8 weeks using dry weight, handgrip dynamometry, and mid-arm circumference. Coordinate with hepatology regarding diuretic dosing and transplant evaluation if indicated. Referral to a registered dietitian (RD) with hepatology expertise is strongly advised for ongoing management.

## C204

**Clinical Nutrition Recommendations**

**HBV Cirrhosis with Ascites — Female, 44 y/o | 161 cm | 72 kg**

**Clinical Note on Body Weight:** Apparent BMI is 27.8 kg/m², suggesting overweight. However, with **active ascites and peripheral edema**, true (dry) body weight is likely **5–8 kg lower**, placing this patient closer to normal weight or even at risk for **occult malnutrition**. All calculations use **estimated dry weight ≈ 64–67 kg**.

**1. Overall Dietary Principles**

| **Principle** | **Rationale** |
| --- | --- |
| **Adequate energy intake** | Prevent catabolism and muscle wasting (sarcopenia is highly prevalent in cirrhosis) |
| **Sodium restriction** | Manage ascites and edema without overly aggressive restriction |
| **No protein restriction** | Outdated practice — protein restriction worsens outcomes in cirrhosis |
| **Soft, easily digestible foods** | Reduce risk of variceal bleeding and GI discomfort |
| **Avoid hepatotoxic substances** | Absolute alcohol abstinence; limit herbal supplements |

**Target Energy Intake:** **30–35 kcal/kg/day** (based on dry weight)

- Estimated: **~1,950–2,250 kcal/day**

**2. Daily Protein Intake**

**Recommended: 1.2 – 1.5 g/kg dry body weight/day**

Estimated target: **≈ 77–100 g protein/day**

**Protein Source Guidance:**

| **Source** | **Recommendation** | **Notes** |
| --- | --- | --- |
| Fish / seafood | Preferred | High biological value, low in saturated fat |
| Lean poultry | Preferred | Chicken breast, turkey |
| Plant protein (soy, legumes) | Beneficial | May reduce ammonia generation vs. animal protein |
| Eggs | Acceptable | Limit yolks if dyslipidemia present |
| Dairy (low-fat) | Acceptable | Good BCAA source |
| Red meat | Limit | Higher ammonia load; max 1–2×/week |
| Processed/cured meats | Avoid | High sodium content |

**Note on Hepatic Encephalopathy (HE):** This patient has **no documented HE**, so protein restriction is **not indicated**. If HE develops, a trial of **branched-chain amino acid (BCAA) enriched formula** (e.g., 12–20 g BCAA/day) should be considered rather than cutting protein.

**3. Meal Frequency and Eating Pattern**

Patients with cirrhosis have **shortened glycogen stores** and enter a fasting/catabolic state much faster than healthy individuals. Frequent feeding is essential.

Recommended: 4–6 small meals per day + mandatory Late Evening Snack (LES)

**Sample Daily Schedule:**

| **Time** | **Meal** | **Purpose** |
| --- | --- | --- |
| **7:00 AM** | Breakfast | Break overnight fast promptly |
| **10:00 AM** | Mid-morning snack | Prevent catabolism |
| **12:30 PM** | Lunch (main meal) | Largest calorie/protein load |
| **3:30 PM** | Afternoon snack | Sustain energy |
| **6:30 PM** | Dinner (moderate) | Balanced, not heavy |
| **9:30–10:00 PM** | ⭐ **Late Evening Snack (LES)** | **Critical** — reduces overnight fasting period |

**⭐ Late Evening Snack (LES) — Key Recommendation**

- **Content:** Complex carbohydrate + protein
- **Examples:**
  - Whole grain crackers + low-fat cheese
  - Small bowl of oatmeal with milk
  - BCAA-enriched oral supplement
- **Evidence:** LES significantly improves nitrogen balance and reduces muscle wasting in cirrhosis patients

**4. Specific Food Recommendations and Restrictions**

**Foods to Encourage:**

- **Complex carbohydrates:** Brown rice, oats, whole-grain bread, sweet potato
- **Vegetables:** All non-starchy vegetables (steamed/boiled preferred); spinach, broccoli, carrots
- **Fruits:** Moderate amounts; berries, apple, pear (watch portion — fructose load)
- **Healthy fats:** Olive oil (in moderation), avocado, nuts (unsalted)
- **Zinc-rich foods:** Beef (limited), pumpkin seeds, legumes — cirrhosis often causes zinc deficiency

**Foods to Restrict or Avoid:**

| **Category** | **Restriction** | **Reason** |
| --- | --- | --- |
| **High-sodium foods** | **< 2,000 mg Na/day** | Ascites and edema management |
| **Alcohol** | **Absolute abstinence** | Ongoing liver damage |
| Fried/fatty foods | Avoid | Difficult to metabolize; steatosis risk |
| Raw shellfish/seafood | Strictly avoid | Risk of *Vibrio vulnificus* — fatal in cirrhosis |
| Canned/processed foods | Avoid | Hidden high sodium |
| Herbal supplements | Avoid unless approved | Many are hepatotoxic |
| Excessive caffeine | Limit | Modest coffee (1–2 cups/day plain) may be hepatoprotective — but avoid if insomnia/anxiety |

**Practical Sodium Restriction Tips:**

- Cook at home with herbs and spices instead of salt
- Read labels: target < 600 mg Na per serving
- Avoid soy sauce, fish sauce, MSG, pickled foods
- Restaurant meals: request sauces on the side, no added salt

**5. Additional Nutritional Considerations**

**Micronutrient Supplementation (likely deficient in cirrhosis):**

| **Nutrient** | **Consideration** | **Action** |
| --- | --- | --- |
| **Zinc** | Commonly deficient; supports ammonia metabolism | Supplement 25–45 mg/day elemental zinc if deficient |
| **Vitamin D** | Frequently low; affects bone health | Check 25-OH Vit D; supplement 800–2000 IU/day |
| **B vitamins (B1, B6, B12, Folate)** | Impaired hepatic storage | Low-dose B-complex supplement |
| **Vitamin K** | Impaired synthesis → coagulopathy | Dietary sources (leafy greens); supplement if PT/INR elevated |
| **Magnesium** | Often depleted (especially if on diuretics) | Monitor levels; supplement if low |

Avoid fat-soluble vitamin **megadosing** (esp. Vitamin A) — hepatotoxic in liver disease.

**Fluid Management:**

- **Mild ascites with edema:** Moderate fluid restriction **~1,500 mL/day** if hyponatremia (Na < 130 mEq/L) is present
- If sodium is normal, fluid restriction is **less strict** but avoid excess free water
- Coordinate with treating hepatologist regarding diuretic therapy (spironolactone ± furosemide)

**Bone Health:**

- HBV cirrhosis + likely vitamin D deficiency → **hepatic osteodystrophy risk**
- Ensure adequate calcium intake: **1,000–1,200 mg/day** through diet (low-fat dairy, fortified foods, tofu)

**HBV-Specific Consideration:**

- If on **antiviral therapy** (e.g., tenofovir), monitor for **renal function and phosphate levels** — tenofovir can impair renal tubular function affecting nutrition
- Maintain **healthy weight trajectory** — obesity worsens fibrosis progression in HBV; however, rapid weight loss is contraindicated with ascites

**Monitoring & Follow-up:**

- Reassess nutritional status every **4–8 weeks**
- Track **mid-arm muscle circumference (MAMC)** and **handgrip strength** as ascites confounds weight measurements
- Repeat labs: albumin, prealbumin, zinc, vitamin D, B12, magnesium at 3 months

**Summary Card**

| **Parameter** | **Target** |
| --- | --- |
| Calories | 1,950–2,250 kcal/day |
| Protein | 77–100 g/day (1.2–1.5 g/kg) |
| Sodium | < 2,000 mg/day |
| Fluid | ~1,500 mL/day if hyponatremic |
| Meals | 5–6 times/day + LES at 9:30–10 PM |
| Alcohol | Absolute zero |
| Raw seafood | Strictly forbidden |

## C205

**Clinical Nutrition Recommendations**

**Patient:** Male, 48 y/o | MASLD Cirrhosis with Recurrent Ascites **Anthropometrics:** 176 cm / 95 kg → BMI 30.6 kg/m² *(Note: True BMI likely overestimated due to ascitic fluid; dry weight assessment recommended)*

**Key Clinical Context**

This patient presents a complex nutritional challenge requiring balance between **three competing priorities**:

- Weight management (obesity/MASLD etiology)
- Prevention of sarcopenia (highly prevalent even in obese cirrhotics)
- Fluid/sodium control (decompensated disease with recurrent ascites)

**Sarcopenic obesity** is common in MASLD cirrhosis and carries higher mortality than either condition alone. **Do not aggressively restrict calories.**

**1. Overall Dietary Principles**

| **Principle** | **Rationale** |
| --- | --- |
| **Moderate caloric restriction** | Target ~1,800–2,200 kcal/day (25–35 kcal/kg *estimated dry weight*) |
| **Strict sodium restriction** | ≤ **2,000 mg Na/day** (≤5 g table salt) to manage ascites |
| **Fluid restriction** | Generally 1,000–1,500 mL/day if dilutional hyponatremia (Na <130 mEq/L) is present; reassess with hepatology |
| **Avoid hepatotoxins** | **Absolute alcohol abstinence**; minimize processed/ultra-processed foods high in fructose and saturated fat |
| **Anti-inflammatory diet pattern** | Mediterranean-style framework is strongly supported in MASLD |

**2. Recommended Daily Protein Intake**

**Target: 1.2–1.5 g protein per kg of *dry body weight* per day**

Estimated dry weight ≈ 85–88 kg (rough estimate subtracting ascites); adjust as ascites resolves.

| **Calculation** | **Amount** |
| --- | --- |
| Conservative (1.2 g/kg × 86 kg) | ~**103 g protein/day** |
| Optimal (1.5 g/kg × 86 kg) | ~**129 g protein/day** |
| **Practical daily target** | **~110–120 g/day** |

**Important Notes on Protein:**

- **Do NOT restrict protein** to prevent hepatic encephalopathy — this practice is outdated and harmful
- **Prefer vegetable and dairy protein** (branched-chain amino acid–rich) over large boluses of red meat
- **Branched-chain amino acids (BCAAs)** — consider supplementation (e.g., 12 g BCAA/day) if oral intake goals are not met or if encephalopathy risk is elevated

**3. Meal Frequency and Eating Pattern**

**Recommendation: 4–6 small meals per day + mandatory late-evening snack**

Breakfast	07:00	~400–450 kcal

Mid-morning	10:00	~200 kcal

Lunch	13:00	~450–500 kcal

Afternoon snack 16:00	~200 kcal

Dinner	19:00	~400–450 kcal

★ Late-night snack 21:00–22:00	~200 kcal (CRITICAL — see below)

**★ The Late-Evening Snack (LES) — Non-Negotiable**

- Cirrhotic patients enter a **catabolic fasting state after only 2–4 hours** of overnight fast (vs. 24–36 hours in healthy individuals)
- A **complex carbohydrate + protein snack** before bed dramatically reduces overnight muscle catabolism
- **Options:** Whole grain crackers with cottage cheese; low-sodium Greek yogurt with oats; a small banana with a handful of unsalted nuts

**4. Specific Food Recommendations and Restrictions**

**Encouraged Foods**

| **Category** | **Examples** |
| --- | --- |
| **Lean protein** | Eggs, tofu, low-fat dairy (unsalted), white fish, skinless poultry, legumes |
| **Complex carbohydrates** | Oats, brown rice, sweet potato, whole grain bread (check Na content) |
| **Healthy fats** | Olive oil (primary fat source), avocado, unsalted nuts/seeds |
| **Vegetables** | Fresh or frozen (not canned/pickled); leafy greens, broccoli, cauliflower |
| **Fruit** | Moderate portions; berries, apples, citrus (limit high-sugar fruits) |
| **Coffee** | 2–3 cups/day — evidence supports hepatoprotective effect in cirrhosis |

**Foods to Restrict or Avoid**

| **Category** | **Reason** |
| --- | --- |
| **Table salt, soy sauce, fish sauce** | Sodium control — ascites management |
| **Canned/processed/deli foods** | Hidden sodium content |
| **Added sugars, sugary beverages, fruit juice** | Worsens MASLD; drives hepatic fat |
| **Saturated and trans fats** (red meat, fried foods, butter) | Promotes steatosis and inflammation |
| **Raw shellfish (oysters, clams)** | Risk of *Vibrio vulnificus* — potentially fatal in cirrhosis |
| **All alcohol** | Absolute contraindication regardless of etiology |
| **High-fructose corn syrup products** | Directly implicated in MASLD pathogenesis |

**Special Sodium Guidance**

- Teach patient to read food labels: target **<140 mg Na per serving**
- Use herbs, lemon juice, garlic, vinegar as flavor substitutes
- Avoid commercial salt substitutes containing **potassium chloride** if hyperkalemia is present (check electrolytes)

**5. Additional Nutritional Considerations**

**Micronutrient Supplementation**

| **Nutrient** | **Status in Cirrhosis** | **Recommendation** |
| --- | --- | --- |
| **Zinc** | Commonly deficient; impairs taste, worsens encephalopathy | 30–45 mg/day elemental zinc |
| **Vitamin D** | Frequently low; worsens osteodystrophy | 1,000–2,000 IU/day (check 25-OH-D level first) |
| **B vitamins (B1, B6, B12, folate)** | Impaired storage and metabolism | Consider B-complex supplementation |
| **Magnesium** | Depleted by diuretics (likely on spironolactone) | Monitor; supplement if low |
| **Fat-soluble vitamins (A, E, K)** | May be malabsorbed | Assess levels; supplement if deficient — avoid excess vitamin A |

**Physical Activity**

- Light-to-moderate exercise (walking, resistance band training) is **safe and recommended** in compensated/stable decompensated cirrhosis
- Resistance training is particularly important to **preserve/build muscle mass** — target 2–3 sessions/week
- Avoid high-impact activity when tense ascites is present

**Monitoring Parameters**

- Reassess **dry weight** monthly as ascites is managed with diuretics
- Monitor electrolytes (**Na, K, Mg**) — especially on diuretic therapy
- Periodic **handgrip strength** or **mid-arm muscle circumference** to track sarcopenia
- Reassess caloric and protein targets if ascites resolves significantly

**Multidisciplinary Coordination**

- Align dietary sodium targets with **hepatology/nephrology** diuretic management (spironolactone ± furosemide)
- Consider referral to a **registered dietitian (RD)** experienced in hepatology for ongoing monitoring
- If oral intake is consistently inadequate (<60% of targets), discuss **oral nutritional supplements** (e.g., low-sodium, branched-chain amino acid–enriched formulas); nasogastric feeding if severely malnourished

**Summary Card for Patient**

**Eat 5–6 small meals daily — never skip the bedtime snack** **Keep sodium under 2,000 mg/day — no added salt, no processed foods** **Aim for ~115 g of protein daily — don't fear protein** **Zero alcohol, ever** **Coffee is fine — 2–3 cups/day is beneficial** 🚶 **Walk or do light resistance exercise daily**

## C206

**Dietary & Nutritional Recommendations**

**Patient:** Female, 52 y/o | 157 cm | 61 kg (BMI ~24.7*) | Alcohol-related cirrhosis with ascites

*Note: Dry weight is likely lower given ascites. True nutritional status may be worse than BMI suggests — sarcopenia can be masked by fluid retention.

**1. Overall Dietary Principles**

- **Avoid all alcohol** — absolute contraindication; ongoing consumption accelerates hepatic fibrosis and decompensation
- **Goal:** Prevent/treat malnutrition and sarcopenia, minimize hepatic encephalopathy risk, and manage fluid/electrolyte balance
- **Caloric target:** **30–35 kcal/kg estimated dry body weight/day** (approximate dry weight ~55–57 kg after accounting for ascites, so ~1,650–2,000 kcal/day)
- Prioritize **nutrient-dense**, easily digestible foods; avoid prolonged fasting (>3–4 hours) which promotes catabolism in cirrhosis

**2. Recommended Daily Protein Intake**

| **Context** | **Target** |
| --- | --- |
| General cirrhosis with ascites (no encephalopathy) | **1.2–1.5 g/kg dry body weight/day** (~66–85 g/day) |
| If sarcopenia is present | Up to **1.5 g/kg/day** |

- **Do NOT restrict protein** to prevent hepatic encephalopathy — this is outdated and harmful
- If encephalopathy develops, prefer **branched-chain amino acids (BCAAs)** and **vegetable/dairy protein** over red meat
- BCAAs (leucine, isoleucine, valine) supplementation (e.g., 12–15 g/day) may be considered to support muscle mass

**3. Meal Frequency and Eating Pattern**

This is critically important in cirrhosis due to reduced glycogen storage capacity:

- **4–6 small meals per day** rather than 2–3 large meals
- **Mandatory late-evening snack (LES):** Consume a complex carbohydrate + protein snack **within 2–3 hours of bedtime**
  - Example: Whole grain toast with peanut butter, or a small bowl of oatmeal with milk
  - This reduces overnight fasting and prevents gluconeogenesis from muscle protein
- **Do not skip breakfast** — begin eating within 1 hour of waking
- Avoid fasting periods longer than 3–4 hours during the day

**4. Specific Food Recommendations & Restrictions**

**Recommended Foods**

| **Category** | **Examples** |
| --- | --- |
| **Protein sources** | Eggs, dairy (yogurt, milk, cottage cheese), poultry, fish, legumes, tofu |
| **Complex carbohydrates** | Oats, brown rice, whole grain bread, sweet potatoes |
| **Healthy fats** | Olive oil, avocado, small amounts of nuts (if no encephalopathy) |
| **Fruits & vegetables** | All types; cooked vegetables may be better tolerated |
| **Hydration** | Water, dilute herbal teas |

**Foods/Substances to Restrict or Avoid**

| **Item** | **Reason** |
| --- | --- |
| **Alcohol** | Absolute contraindication |
| **High-sodium foods** (processed meats, canned soups, fast food, soy sauce, pickles) | Worsens fluid retention and ascites — target **<2,000 mg sodium/day** |
| **Raw shellfish/undercooked seafood** | Risk of fatal *Vibrio vulnificus* infection in cirrhosis |
| **High-sodium condiments** | Hidden sodium contributes significantly |
| **Grapefruit** | CYP3A4 inhibition; may interact with medications |
| **Excess red meat** | Higher ammonia production; substitute with white meat/plant protein |
| **Herbal supplements** (kava, valerian, comfrey) | Hepatotoxic potential |

**5. Additional Nutritional Considerations**

**Sodium & Fluid Management**

- **Sodium restriction: <2,000 mg/day** (some guidelines suggest <1,500 mg/day for refractory ascites)
- Fluid restriction is generally **only needed if serum sodium <125 mEq/L**; otherwise unnecessary
- Teach the patient to read food labels — >20% DV sodium per serving = high sodium

**Micronutrient Supplementation (High Priority in Alcohol-Related Cirrhosis)**

| **Nutrient** | **Rationale** | **Consideration** |
| --- | --- | --- |
| **Thiamine (B1)** | Depleted by alcohol; prevents Wernicke's encephalopathy | 100 mg/day (oral); IV if neurological symptoms |
| **Folate (B9)** | Commonly deficient | 1 mg/day |
| **Zinc** | Supports ammonia metabolism; often low in cirrhosis | 30–45 mg/day elemental zinc |
| **Vitamin D** | Impaired hepatic 25-hydroxylation; bone disease risk | Supplement per level; typically 1,000–2,000 IU/day |
| **Magnesium** | Depleted with diuretic use (if on spironolactone/furosemide) | Monitor and replace as needed |
| **Vitamin K** | Impaired synthesis may worsen coagulopathy | 10 mg/day if deficient or PT elevated |
| **B12, B6** | Common deficiency in alcohol use | Included in a B-complex supplement |

A general **multivitamin without iron** is reasonable (excess iron is harmful in cirrhosis).

**Monitoring & Follow-Up**

- **Weigh daily** at home — gain of >1 kg/day or >2 kg/week signals fluid accumulation; notify provider
- Monitor for signs of **hepatic encephalopathy** (confusion, asterixis) — if they emerge, reassess protein distribution and increase BCAA intake
- Refer to a **registered dietitian (RD)** with hepatology experience for ongoing monitoring
- Screen regularly for **sarcopenia** using mid-arm muscle circumference or handgrip strength

**Behavioral Considerations**

- Provide **alcohol cessation support** — referral to addiction medicine/counseling is essential
- Poor appetite is common; suggest **small, appealing, calorie-dense** meals and oral nutritional supplements (e.g., Ensure, Boost) if oral intake remains inadequate
- Consider **appetite stimulants** or an **oral nutritional supplement (ONS)** if caloric targets cannot be met through food alone

**Summary Card for Patient**

Eat **4–6 small meals** daily · Always eat a **bedtime snack** · Aim for **70–80 g protein/day** · Keep salt **very low** · **No alcohol, ever** · Take your vitamin supplements · Weigh yourself every morning

## C207

**Clinical Nutrition Recommendations**

**Patient: Male, 56 y/o | HBV Cirrhosis with Ascites | HT: 170 cm | WT: 66 kg (wet weight)**

**Clinical Note on Body Weight** With active ascites, the recorded weight of 66 kg is likely **fluid-overloaded**. True dry (lean body) weight is estimated to be **lower** — potentially 60–63 kg. All nutritional calculations below are based on an **estimated dry weight of ~62 kg**. Nutritional assessment should be supplemented with anthropometrics (mid-arm circumference, handgrip strength) given the unreliability of BMI in ascites.

**1. Overall Dietary Principles**

| **Principle** | **Rationale** |
| --- | --- |
| **High-calorie, adequate-protein diet** | Counteract hypercatabolic state of cirrhosis |
| **Sodium restriction (< 2,000 mg/day)** | Manage ascites and fluid retention |
| **Fluid intake monitoring** | Restrict to ~1,000–1,500 mL/day if dilutional hyponatremia present |
| **Avoid prolonged fasting** | Cirrhotic liver has limited glycogen reserve; fasting accelerates muscle catabolism |
| **Small, frequent meals** | Improves tolerance, reduces risk of encephalopathy, optimizes nitrogen utilization |
| **Abstain completely from alcohol** | Ongoing hepatotoxicity even in HBV-related cirrhosis |

**2. Recommended Daily Protein Intake**

**Target: 1.2 – 1.5 g/kg dry body weight/day → approximately 74 – 93 g protein/day**

**Protein restriction is NOT recommended** in stable cirrhosis — this is an outdated practice and accelerates sarcopenia. Protein restriction is only transiently considered during **acute, refractory hepatic encephalopathy**.

**Protein Source Prioritization:**

- **Preferred:** Plant-based proteins (legumes, soy) and **branched-chain amino acid (BCAA)-rich** sources
- **Good options:** Eggs, low-fat dairy, fish, lean poultry
- **Limit:** Red meat and processed meat (high ammoniagenic potential)
- **Consider:** BCAA oral supplementation (e.g., 12–14 g BCAA/day) if dietary intake is insufficient or sarcopenia is evident — supported by ESPEN and JSH guidelines

**3. Meal Frequency and Eating Pattern**

A structured **4–6 meal/day pattern** is strongly recommended:

Breakfast	~7:00 AM	20–25% of daily calories

Mid-morning	~10:00 AM	10% of daily calories (snack)

Lunch	~12:30 PM	25–30% of daily calories

Afternoon	~3:30 PM	10% of daily calories (snack)

Dinner	~6:30 PM	20–25% of daily calories

━━━━━━━━━━━━━━━━━━━━━━━━━━━━━━━━━━━━━━━

★ Late Evening Snack ~9:30–10:00 PM ~200 kcal (CRITICAL)

**⭐ Late Evening Snack (LES) — High Priority Recommendation**

- Consume **200–250 kcal** before bed, ideally carbohydrate + protein (e.g., whole-grain bread with peanut butter, or a BCAA-enriched drink)
- **Evidence-based:** LES reduces overnight fasting duration, decreases protein catabolism, and has been shown to improve nitrogen balance and muscle mass in cirrhotic patients (Miwa et al., Hepatology)

**4. Specific Food Recommendations and Restrictions**

**Recommended Foods**

| **Category** | **Examples** |
| --- | --- |
| **Complex carbohydrates** | Oatmeal, brown rice, whole-grain bread, sweet potato |
| **Lean protein** | Egg whites, tofu, steamed fish, skinless chicken |
| **Plant-based protein** | Lentils, edamame, chickpeas, soy milk |
| **Healthy fats** | Olive oil, avocado, walnuts (in moderation) |
| **Vegetables** | Cooked/soft vegetables (easier digestion); avoid raw if thrombocytopenic |
| **Fruit** | Banana, papaya, apple — monitor potassium if on diuretics |

**Foods to Restrict or Avoid**

| **Category** | **Reason** |
| --- | --- |
| **High-sodium foods** (processed, canned, pickled, soy sauce) | Worsens ascites and fluid retention |
| **Raw shellfish / undercooked seafood** | Risk of *Vibrio vulnificus* — life-threatening in cirrhosis |
| **Alcohol** (all forms) | Ongoing hepatocellular injury |
| **High-ammonia foods** (excess red meat, organ meats) | Encephalopathy risk |
| **Herbal supplements / TCM remedies** | Potential hepatotoxicity; always verify before use |
| **High-fat, fried foods** | Impair gastric emptying; aggravate portal hypertension symptoms |
| **Vitamin A supplements** | Hepatotoxic in excess in cirrhosis |

**5. Additional Nutritional Considerations**

**Caloric Target**

- **Total energy: 30–35 kcal/kg dry weight/day → ~1,860–2,170 kcal/day**
- If evidence of malnutrition or sarcopenia: titrate toward **35–40 kcal/kg/day**

**Micronutrients — High-Risk Deficiencies in HBV Cirrhosis**

| **Nutrient** | **Recommendation** |
| --- | --- |
| **Zinc** | Often deficient in cirrhosis; supplement 50 mg/day if low — also reduces ammonia |
| **Vitamin D** | Frequently deficient; check 25-OH Vitamin D; supplement 800–2000 IU/day |
| **B vitamins (B1, B6, B12, Folate)** | Supplement as part of multivitamin; B1 critical if any prior alcohol use |
| **Magnesium** | Monitor if on diuretics (spironolactone/furosemide); replete as needed |
| **Potassium** | Diuretic use increases excretion — monitor serum levels; banana, potato may help |
| **Iron** | **Do NOT supplement empirically** — iron overload is harmful in HBV cirrhosis |

**Fluid and Sodium Monitoring**

- Weigh daily at the **same time each morning** (after voiding, before eating)
- **Alert threshold:** Weight gain > 1 kg in 1 day or > 2 kg in 3 days → contact physician
- Track urine output if on diuretic therapy

**Physical Activity**

- Encourage **light aerobic exercise** (20–30 min walking, 3–5×/week) if not contraindicated by decompensation severity
- Resistance exercise for sarcopenia preservation — always individualized

**Referral & Follow-up**

- Formal **dietitian follow-up every 4–8 weeks**
- Consider **BCAA supplementation reassessment** at 3 months with repeat handgrip/albumin
- Re-evaluate dietary plan after any hospitalization, procedure (e.g., paracentesis), or change in diuretic regimen

**Summary Priority List for This Patient:**

1. Adequate protein ≥1.2 g/kg/day — do not restrict
2. Sodium < 2,000 mg/day — strict
3. Daily late evening snack — non-negotiable
4. 4–6 small meals — no prolonged fasting
5. Daily weight monitoring — track fluid status

## C208

**Clinical Nutrition Recommendations**

**Patient: Female, 59 y/o | MASLD Cirrhosis with Ascites | HT: 155 cm | WT: 80 kg (wet weight)**

**Critical Weight & Body Composition Assessment**

- **Recorded BMI: 33.3 kg/m²** (Obese Class I) — but this is fluid-confounded
- With active ascites, estimated **dry weight ≈ 72–75 kg** (subtracting ~5–8 kg ascitic fluid)
- **Dry weight BMI ≈ 30.0–31.2 kg/m²** — still in the obese range
- **Sarcopenic obesity** is highly prevalent in MASLD cirrhosis — excess adipose tissue can mask significant underlying muscle wasting. Do NOT rely on BMI alone. Assess with **handgrip strength, mid-arm muscle circumference (MAMC), or CT-based psoas muscle index** if available.
- All nutritional calculations use **estimated dry weight of ~73 kg**

**MASLD-Specific Metabolic Context**

Unlike viral or alcoholic cirrhosis, **MASLD cirrhosis is rooted in metabolic dysfunction.** This patient likely carries one or more of the following comorbidities that must be integrated into the dietary plan:

| **Metabolic Feature** | **Dietary Implication** |
| --- | --- |
| **Insulin resistance / Type 2 DM** | Prioritize low-glycemic carbohydrates; limit refined sugars |
| **Central obesity** | Moderate caloric restriction is appropriate — but cautiously |
| **Dyslipidemia** | Limit saturated fat and trans fats; favor unsaturated fats |
| **Hypertension** | Reinforces the need for strict sodium restriction |
| **Metabolic inflammation** | Emphasize anti-inflammatory dietary pattern (Mediterranean) |

**1. Overall Dietary Principles**

| **Principle** | **Rationale** |
| --- | --- |
| **Moderate caloric deficit with high diet quality** | Promote gradual fat loss while preserving lean mass |
| **Mediterranean-style dietary pattern** | Evidence-based for MASLD: reduces hepatic steatosis, inflammation, and cardiovascular risk |
| **Sodium restriction (< 2,000 mg/day)** | Management of ascites and fluid retention |
| **Eliminate added sugars and fructose** | Fructose drives de novo hepatic lipogenesis — central to MASLD pathogenesis |
| **Avoid prolonged fasting** | Cirrhotic liver has severely limited glycogen stores — fasting is catabolic |
| **Small, frequent meals** | Optimize nitrogen utilization; reduce encephalopathy risk; improve glycemic control |
| **Complete alcohol abstinence** | Even small amounts accelerate fibrosis progression in MASLD cirrhosis |

**Target weight loss rate: 0.5–1.0 kg/week** (dry weight) — rapid weight loss (>1.5 kg/week) is harmful and can precipitate hepatic decompensation and worsen portal hypertension

**2. Recommended Daily Protein Intake**

**Target: 1.2 – 1.5 g/kg dry body weight/day → approximately 88 – 110 g protein/day**

**Do NOT restrict protein** despite obesity. In cirrhosis, protein restriction dramatically worsens sarcopenia — the dominant predictor of mortality regardless of adiposity.

Dry weight (~73 kg) × 1.2 g = 88 g protein/day (minimum)

Dry weight (~73 kg) × 1.5 g = 110 g protein/day (target)

**Protein Source Strategy for MASLD:**

| **Priority** | **Source** | **Benefit** |
| --- | --- | --- |
| ⭐⭐⭐ | **Plant-based proteins** (legumes, tofu, tempeh, edamame) | Low ammoniagenic load, high fiber, improves insulin sensitivity |
| ⭐⭐⭐ | **Fatty fish** (salmon, mackerel, sardines — low sodium prep) | Omega-3 fatty acids reduce hepatic steatosis and inflammation |
| ⭐⭐ | **Eggs, low-fat dairy, skinless poultry** | Complete amino acid profile, moderate fat |
| ⭐ | **Lean red meat** | Limit to 1–2×/week maximum |
|  | **Processed meats, organ meats** | High sodium, saturated fat, ammoniagenic |

**Consider BCAA supplementation (12–14 g/day)** if dietary protein targets cannot be met or if sarcopenia is confirmed — BCAAs improve nitrogen balance and are associated with reduced hepatic encephalopathy risk

**3. Meal Frequency and Eating Pattern**

A structured **4–6 meal/day pattern** is essential:

Breakfast	~7:00 AM	20% of daily calories

Mid-morning	~10:00 AM	10% (protein-rich snack)

Lunch	~12:30 PM	25–30% of daily calories

Afternoon	~3:30 PM	10% (low-GI snack)

Dinner	~6:00 PM	20–25% of daily calories

━━━━━━━━━━━━━━━━━━━━━━━━━━━━━━━━━━━━━━━━━━━━━━━

★ Late Evening Snack	~9:30 PM	~200 kcal (MANDATORY)

**⭐ Late Evening Snack (LES) — Non-Negotiable**

- **200 kcal**, ideally **complex carbohydrate + protein combination**
- Examples: whole-grain crackers with hummus; unsweetened soy milk with oats; a small portion of Greek yogurt (low-fat, low-sugar) with walnuts
- Prevents overnight starvation-equivalent catabolism unique to cirrhotic metabolism
- **Avoid simple sugars at this time** (e.g., fruit juice, white bread) — worsens overnight glycemia in insulin-resistant patients

**4. Specific Food Recommendations and Restrictions**

**Recommended Foods — Mediterranean-MASLD Framework**

| **Category** | **Best Choices** | **Notes** |
| --- | --- | --- |
| **Carbohydrates** | Oats, quinoa, barley, whole-grain bread, sweet potato, brown rice | Low glycemic index; high fiber |
| **Vegetables** | Leafy greens, broccoli, cauliflower, zucchini, tomato, bell pepper | Aim for ≥ 5 portions/day; cooked preferred |
| **Fruit** | Berries, apple, pear, citrus (in moderation) | High fiber, lower fructose options preferred |
| **Protein** | Legumes, tofu, fish, eggs, low-fat yogurt | See protein section above |
| **Fats** | Extra-virgin olive oil (1–2 tbsp/day), walnuts, flaxseed, avocado | Anti-inflammatory; hepatoprotective |
| **Beverages** | Water, unsweetened green tea, black coffee (2–3 cups/day) | Coffee is evidence-based for MASLD — reduces fibrosis progression |

**Foods to Strictly Restrict or Avoid**

| **Food / Beverage** | **Reason** |
| --- | --- |
| **Sugar-sweetened beverages** (sodas, juices, sports drinks) | Fructose directly drives hepatic steatosis — top priority to eliminate |
| **Added sugars, sweets, pastries, desserts** | High glycemic load; worsens insulin resistance |
| **Refined carbohydrates** (white bread, white rice, instant noodles) | Rapid glucose spike; promotes lipogenesis |
| **High-sodium foods** (canned goods, processed foods, pickles, soy sauce) | Exacerbates ascites — use low-sodium soy sauce or herbs instead |
| **Saturated and trans fats** (butter, fried foods, full-fat dairy, pastries) | Worsens hepatic steatosis and dyslipidemia |
| **Alcohol** (all forms, including "light" beer or wine) | Zero tolerance in MASLD cirrhosis |
| **Raw shellfish / undercooked seafood** | Fatal *Vibrio* infection risk in cirrhosis |
| **Fructose-rich foods** (honey, high-fructose corn syrup, agave) | Even "natural" fructose is harmful in MASLD |
| **Herbal supplements / weight loss teas** | High hepatotoxicity risk — especially in MASLD |

**5. Additional Nutritional Considerations**

**Caloric Target**

Maintenance: ~25–30 kcal/kg dry weight = 1,825–2,190 kcal/day

With deficit: ~25 kcal/kg dry weight = ~1,825 kcal/day

A **moderate deficit of ~500 kcal/day from maintenance** is appropriate for this patient, targeting ~0.5 kg/week weight reduction. Avoid aggressive restriction below **1,600 kcal/day** as this risks precipitating malnutrition in a cirrhotic patient.

**Carbohydrate Quality — Critical in MASLD**

| **Avoid** | **Prefer** |
| --- | --- |
| Simple sugars, fructose, refined grains | Complex carbs, high-fiber whole grains |
| High glycemic index foods | Low–moderate glycemic index foods |
| Large carbohydrate portions in single sitting | Distributed, smaller carbohydrate portions across meals |

- **Daily fiber target: 25–35 g/day** — supports gut microbiome, reduces ammonia absorption, improves insulin sensitivity
- If Type 2 diabetes is confirmed: coordinate with endocrinology; consider dietitian-guided carbohydrate counting

**🫒 Fat Quality — Mediterranean Emphasis**

| **Type** | **Recommendation** |
| --- | --- |
| **Monounsaturated fat** (olive oil) | Preferred cooking fat — 1–2 tbsp/day |
| **Omega-3 PUFA** (fatty fish, flaxseed, walnuts) | 2–3 servings fatty fish/week — reduces hepatic fat |
| **Saturated fat** | < 7–10% of total calories |
| **Trans fat** | Eliminate entirely |

**Micronutrients — MASLD-Specific Deficiency Profile**

| **Nutrient** | **Recommendation** |
| --- | --- |
| **Vitamin D** | Deficiency nearly universal in MASLD + obesity; check 25-OH Vit D; supplement **1,000–4,000 IU/day** |
| **Vitamin E** | 800 IU/day alpha-tocopherol has evidence for non-cirrhotic MASLD — use **cautiously in cirrhosis** only under physician supervision; **avoid in diabetes** |
| **Zinc** | Often deficient; supplement **50 mg/day** elemental zinc — also reduces ammonia and improves taste perception |
| **Magnesium** | Monitor if on spironolactone/furosemide; low magnesium worsens insulin resistance |
| **Potassium** | Diuretic-related loss; monitor serum levels; replete with food (cooked spinach, banana, potato) |
| **B vitamins** | Ensure adequate folate and B12 — support methylation and homocysteine metabolism |
| **Iron** | Do NOT supplement empirically — iron overload worsens hepatic oxidative stress in MASLD |
| **Calcium** | 1,000–1,200 mg/day through diet (post-menopausal female) — low-fat dairy or fortified plant milk |

**Fluid and Weight Monitoring Protocol**

| **Parameter** | **Target / Action** |
| --- | --- |
| **Daily sodium intake** | < 2,000 mg/day (strictly); use herbs, lemon, vinegar for flavor |
| **Fluid intake** | Restrict to 1,000–1,500 mL/day only if hyponatremia (Na < 130 mEq/L) present |
| **Daily morning weight** | Same time, after voiding, before eating |
| **Alert: Weight gain** | > 1 kg/day or > 2 kg in 3 days → contact physician (fluid reaccumulation) |
| **Alert: Weight loss** | > 1 kg/week (dry) → reassess caloric adequacy |

**🫀 Cardiovascular Risk — Heightened in MASLD**

- MASLD cirrhosis carries **significantly elevated cardiovascular mortality** — dietary modifications benefit both liver and heart
- **DASH + Mediterranean hybrid diet** is the optimal framework for this patient
- Limit dietary cholesterol to < 200 mg/day if dyslipidemia confirmed
- Coordinate lipid-lowering strategies with hepatologist (statins may be used cautiously in compensated cirrhosis)

**Physical Activity**

- **Aerobic exercise:** 30–45 min, moderate intensity, 4–5×/week (e.g., brisk walking, cycling)
- **Resistance training:** 2–3×/week — critical for preserving/rebuilding muscle mass in sarcopenic obesity
- Exercise improves insulin sensitivity, reduces hepatic steatosis, and improves portal pressure
- Avoid high-impact activity if varices present — confirm with hepatologist

**Summary Priority Action List**

| **Priority** | **Recommendation** |
| --- | --- |
| **#1** | Eliminate all sugar-sweetened beverages and added fructose — immediately |
| **#2** | Sodium < 2,000 mg/day — strict ascites management |
| **#3** | Protein ≥ 1.2 g/kg dry weight/day — protect against sarcopenia |
| **#4** | 4–6 small meals/day including mandatory late evening snack |
| **#5** | Mediterranean dietary pattern — whole grains, olive oil, legumes, fish |
| **#6** | Moderate caloric deficit (~500 kcal) — target 0.5 kg/week weight loss |
| **#7** | Daily weight monitoring — distinguish fat loss from fluid shifts |
| **#8** | Vitamin D and Zinc supplementation after laboratory confirmation |
| **#9** | Combined aerobic + resistance exercise program |
| **#10** | Dietitian follow-up every 4–6 weeks with reassessment of body composition |

## C209

**Clinical Nutrition Recommendations**

**Patient: Male, 63 y/o | Alcohol Cirrhosis with Ascites + Peripheral Edema | HT: 167 cm | WT: 60 kg (wet weight)**

**Critical Weight & Fluid Assessment**

- **Recorded BMI: 21.5 kg/m²** — appears within normal range but is **profoundly misleading**
- Patient has **dual fluid overload**: ascites (intraperitoneal) **+** peripheral edema (interstitial)
- Combined fluid excess estimated at **6–10 kg**
- **Estimated dry weight: ~52–54 kg** → **Estimated dry BMI: ~18.7–19.4 kg/m²** — places this patient at the **borderline underweight/malnourished threshold**
- This patient is at **high risk of severe protein-calorie malnutrition** masked by fluid retention — arguably the most nutritionally vulnerable profile among cirrhosis subtypes
- All calculations based on **estimated dry weight of ~53 kg**
- Assess with **handgrip dynamometry, mid-arm circumference (MAC), mid-arm muscle circumference (MAMC)**, and **triceps skinfold thickness** — standard scales are unreliable

**Alcohol Cirrhosis — Unique Nutritional Context**

Alcoholic cirrhosis carries the **most severe and multidimensional malnutrition burden** of all cirrhosis etiologies. Alcohol displaces nutrient-dense food intake, impairs intestinal absorption, increases urinary micronutrient excretion, and directly damages hepatocytes responsible for nutrient metabolism and storage.

| **Mechanism** | **Nutritional Consequence** |
| --- | --- |
| **Caloric displacement by alcohol** | Chronic protein-calorie malnutrition |
| **Impaired pancreatic and bile secretion** | Fat malabsorption → fat-soluble vitamin deficiency |
| **Intestinal mucosal injury (leaky gut)** | Reduced absorption of thiamine, folate, zinc, magnesium |
| **Increased urinary losses** | Zinc, magnesium, potassium wasting |
| **Impaired hepatic activation** | Reduced conversion of Vit D, B6, folate to active forms |
| **Anorexia and early satiety** | Inadequate oral intake, worsened by ascites-related gastric compression |
| **Thiamine-specific depletion** | Risk of **Wernicke's Encephalopathy** — neurological emergency |
| **Refeeding syndrome risk** | Dangerous electrolyte shifts upon aggressive nutritional repletion |

**1. Overall Dietary Principles**

| **Principle** | **Rationale** |
| --- | --- |
| **High-calorie, high-protein nutritional rehabilitation** | Severe malnutrition reversal is the primary nutritional goal |
| **Absolute alcohol abstinence — zero tolerance** | The single most impactful intervention; continued drinking abolishes all nutritional benefit |
| **Sodium restriction (< 2,000 mg/day)** | Dual fluid overload (ascites + edema) requires strict sodium control |
| **Fluid restriction if hyponatremia present** | Restrict to 1,000–1,500 mL/day if serum Na < 130 mEq/L |
| **Thiamine repletion before glucose loading** | Administering glucose before thiamine can precipitate acute Wernicke's encephalopathy |
| **Cautious refeeding approach** | Severely malnourished patients risk fatal refeeding syndrome — gradual caloric escalation |
| **Small, frequent meals to maximize intake** | Ascites compresses stomach; early satiety limits single-meal intake |
| **Anti-inflammatory, nutrient-dense food choices** | Counteract ongoing hepatic inflammation and oxidative stress |
| **Structured mealtimes** | Re-establish eating regularity disrupted by years of alcohol-driven behavior |

⭐ **The single most important intervention** for this patient beyond abstinence is **aggressive but carefully titrated nutritional rehabilitation** — malnutrition in alcoholic cirrhosis is independently associated with hepatic encephalopathy, infections, variceal bleeding, and 90-day mortality.

**2. Recommended Daily Protein Intake**

**Target: 1.5 g/kg dry body weight/day → approximately 80 g protein/day** *(Higher end of the cirrhosis range justified by degree of malnutrition)*

Dry weight (~53 kg) × 1.2 g = 64 g/day	(absolute minimum)

Dry weight (~53 kg) × 1.5 g = 80 g/day	(recommended target)

Dry weight (~53 kg) × 1.8 g = 95 g/day (if sarcopenia confirmed + encephalopathy-free)

**Protein restriction is absolutely contraindicated** — historical practice of protein restriction in cirrhosis has been definitively abandoned. Only transiently reconsider in **acute, refractory hepatic encephalopathy (Grade III–IV)** and even then limit to 48–72 hours maximum.

**Protein Source Hierarchy for Alcoholic Cirrhosis:**

| **Priority** | **Source** | **Rationale** |
| --- | --- | --- |
| ⭐⭐⭐ | **Plant-based proteins** (lentils, tofu, tempeh, soy, chickpeas) | Low ammoniagenic load; gut microbiome benefits; high fiber |
| ⭐⭐⭐ | **Eggs** (whole eggs, egg whites) | Complete amino acid profile; highly bioavailable; easy to prepare |
| ⭐⭐⭐ | **Low-fat dairy** (yogurt, cottage cheese, milk) | Protein + calcium + B12; palatable even with anorexia |
| ⭐⭐ | **Lean fish** (cod, tilapia, salmon) | Omega-3 anti-inflammatory benefit; high-quality protein |
| ⭐⭐ | **Skinless poultry** (chicken, turkey) | Lean complete protein; versatile preparation |
| ⭐ | **Lean red meat** | Limit to ≤ 1–2×/week — higher ammonia precursor load |
|  | **Processed meats** (bacon, sausage, ham, deli meats) | High sodium + saturated fat + preservatives — avoid entirely |

**BCAA Oral Supplementation: Strongly Recommended**

- Target: **12–14 g BCAA/day** (leucine-enriched formulation preferred)
- Alcoholic cirrhosis depletes BCAAs while elevating aromatic amino acids (AAAs) — this imbalance directly contributes to hepatic encephalopathy
- BCAA supplementation improves encephalopathy frequency, nitrogen balance, and event-free survival (Grade A evidence — ESPEN, JSH)

**3. Meal Frequency and Eating Pattern**

A strictly structured **5–6 meal/day pattern** is essential, with special attention to meal palatability given anorexia:

Early Breakfast	~7:00 AM	~20% of daily calories

(prioritize — appetite typically best in morning)

Mid-morning	~9:30 AM	~10% (protein-rich snack)

Lunch	~12:30 PM	~25% of daily calories

Afternoon	~3:30 PM	~10% (nutrient-dense snack)

Early Dinner	~6:00 PM	~20–25% of daily calories

━━━━━━━━━━━━━━━━━━━━━━━━━━━━━━━━━━━━━━━━━━━━━━━━

★ Late Evening Snack ~9:30 PM ~200–250 kcal [CRITICAL]

**⭐ Late Evening Snack (LES) — Highest Priority Intervention**

- **200–250 kcal**: Complex carbohydrate + protein + small amount of healthy fat
- Ideal examples:
  - Whole-grain toast with peanut butter + warm low-fat milk
  - Oatmeal with soy protein powder and walnuts
  - Low-fat Greek yogurt with banana and oats
  - BCAA-enriched oral nutritional supplement drink
- **Clinical rationale:** Cirrhotic liver has only 1/3 the glycogen storage capacity of a healthy liver. An overnight fast of 8+ hours is metabolically equivalent to a **72-hour fast in a healthy individual** — accelerating protein catabolism, gluconeogenesis from muscle, and ammonia generation
- LES has Level I evidence in cirrhosis for improving nitrogen balance, muscle mass, and quality of life

**Practical Intake Strategies (Addressing Anorexia)**

| **Challenge** | **Strategy** |
| --- | --- |
| **Early satiety from ascites** | Smaller plates; eat slowly; liquid calories between (not during) meals |
| **Taste changes from zinc deficiency** | Zinc supplementation improves palatability within 2–4 weeks |
| **Meal preparation difficulty** | Simple, pre-prepared options; family/caregiver involvement |
| **Psychological aversion to eating** | Address alcohol withdrawal-related depression and anxiety with mental health team |
| **Nausea or dyspepsia** | Ginger tea, small cold meals, avoid strong odors during cooking |

**4. Specific Food Recommendations and Restrictions**

**Recommended Foods**

| **Category** | **Best Choices** | **Notes** |
| --- | --- | --- |
| **Complex carbohydrates** | Oatmeal, whole-grain bread, sweet potato, brown rice, barley | Steady glucose; high B vitamins; high fiber |
| **Protein foods** | Eggs, tofu, legumes, low-fat dairy, fish, lean chicken | See protein section above |
| **Vegetables** | Cooked leafy greens, broccoli, carrots, zucchini, beets | Soft-cooked for palatability; folate-rich greens prioritized |
| **Fruit** | Banana (K+), citrus (Vit C), berries (antioxidants), melon | Monitor K+ if on diuretics |
| **Healthy fats** | Olive oil, walnuts, flaxseed, avocado | Anti-inflammatory omega-3/MUFA |
| **Beverages** | Water, herbal teas, black coffee (2–3 cups/day) | Coffee is hepatoprotective; reduces fibrosis and HCC risk |
| **Oral supplements** | Nutritional drinks (e.g., Ensure, Fresubin Liver) if intake insufficient | BCAA-enriched formulas preferred in cirrhosis |

**Foods and Substances to Strictly Avoid**

| **Item** | **Reason** |
| --- | --- |
| **Alcohol** (beer, wine, spirits, cooking wine, kombucha) | Zero tolerance — single most critical restriction |
| **High-sodium foods** (processed, canned, pickled, soy sauce, MSG, fast food) | Exacerbates ascites and edema — strict < 2,000 mg/day |
| **Raw or undercooked shellfish and seafood** | *Vibrio vulnificus* and *Listeria* — rapidly fatal in cirrhosis; immunocompromised state |
| **Unpasteurized foods** (raw milk, soft cheeses, raw sprouts) | Bacteremia risk in cirrhotic immunodeficiency |
| **High-fat fried foods, trans fats** | Impairs gastric emptying; worsens portal hemodynamics |
| **Sugary beverages and foods** | Empty calories that displace nutrient-dense intake |
| **Herbal supplements / traditional remedies / detox teas** | Extremely high hepatotoxicity risk — polypharmacy danger in cirrhosis |
| **Excess red meat / organ meats** | High ammoniagenic load — increases encephalopathy risk |
| **Non-steroidal anti-inflammatory drugs (NSAIDs)** | Worsen ascites and precipitate hepatorenal syndrome — flag to prescribing physician |

**5. Additional Nutritional Considerations**

**Caloric Target**

Maintenance: 30–35 kcal/kg dry weight × 53 kg = 1,590–1,855 kcal/day

Malnourished: 35–40 kcal/kg dry weight × 53 kg = 1,855–2,120 kcal/day ← Target for this patient

Given the estimated dry weight BMI of ~18.7–19.4 kg/m², this patient should target the **higher caloric range (35–40 kcal/kg/day)** to facilitate nutritional rehabilitation. However, initiate at a **lower level (~20–25 kcal/kg/day)** and escalate gradually over 5–7 days to avoid refeeding syndrome.

**Refeeding Syndrome — High-Risk Patient**

**This patient is at HIGH RISK for refeeding syndrome** due to chronic malnutrition from alcoholism. Refeeding syndrome is a potentially fatal complication characterized by dangerous shifts of phosphate, potassium, and magnesium into cells upon reintroduction of nutrition, causing cardiac arrhythmias, respiratory failure, and neurological deterioration.

| **Refeeding Precaution** | **Action** |
| --- | --- |
| **Check baseline electrolytes** | Serum phosphate, potassium, magnesium, sodium before initiating nutritional support |
| **Gradual caloric escalation** | Start at 10–20 kcal/kg/day for first 2–3 days; increase by 200–300 kcal every 2 days |
| **Thiamine before glucose** | **MANDATORY** — administer thiamine 100–300 mg/day **before or simultaneously** with any glucose or carbohydrate-containing nutrition |
| **Daily electrolyte monitoring** | Monitor phosphate, K+, Mg2+ daily for first week of nutritional rehabilitation |
| **Replete electrolytes proactively** | IV or oral phosphate, potassium, magnesium supplementation as guided by levels |

**Micronutrient Repletion — Alcohol Cirrhosis Priority Profile**

| **Nutrient** | **Status in Alcoholic Cirrhosis** | **Recommendation** |
| --- | --- | --- |
| **Thiamine (B1)** | Critically depleted — Wernicke's risk | **100–300 mg/day orally** (parenteral if encephalopathy present); START BEFORE glucose |
| **Folate (B9)** | Severely deficient — impaired absorption + intake | **1–5 mg/day** oral supplementation |
| **Pyridoxine (B6)** | Deficient — alcohol directly inactivates B6 | **50–100 mg/day** as part of B-complex |
| **Cobalamin (B12)** | Variable — serum levels may be falsely normal | Monitor methylmalonic acid; supplement 500–1,000 mcg/day |
| **Vitamin D** | Severely deficient — fat malabsorption + poor intake | Check 25-OH Vit D; supplement **2,000–4,000 IU/day** |
| **Zinc** | Severely depleted — urinary wasting + poor intake | **50 mg elemental zinc/day** — also reduces encephalopathy frequency |
| **Magnesium** | Severely depleted — diuretic + alcohol-related urinary loss | Check serum Mg2+; oral or IV repletion as needed; target high-normal |
| **Potassium** | Depleted — diuretic therapy + poor intake | Monitor daily; replete with food + oral KCl supplement; banana, cooked spinach, potato |
| **Phosphate** | Risk of refeeding hypophosphatemia | Monitor during refeeding — see above |
| **Vitamin K** | Fat malabsorption-related deficiency | Supplement if INR elevated beyond coagulopathy of cirrhosis; 10 mg/day orally |
| **Vitamin A** | Deficient but supplement cautiously | Low-dose only (< 5,000 IU/day) — hepatotoxic in excess; assess levels first |
| **Vitamin E** | Fat malabsorption-related deficiency | Moderate-dose supplement (100–200 IU/day); avoid high doses |
| **Iron** | Do NOT supplement empirically | Alcoholic cirrhosis may have iron overload (hemosiderosis) — check ferritin/transferrin first |
| **Calcium** | At risk — Vit D deficiency + malabsorption | 1,000–1,200 mg/day via diet + supplement; critical for bone health |

**A comprehensive B-complex supplement** (B1 + B2 + B3 + B5 + B6 + B9 + B12) is standard of care for alcoholic cirrhosis. Individual thiamine dosing must be explicitly addressed beyond what a standard B-complex provides.

**Fluid, Sodium and Weight Monitoring Protocol**

| **Parameter** | **Target / Action** |
| --- | --- |
| **Sodium intake** | < 2,000 mg/day; use herbs, lemon juice, spices for flavor enhancement |
| **Fluid intake** | Restrict to 1,000–1,500 mL/day **only** if Na < 130 mEq/L; otherwise free fluids |
| **Daily morning weight** | After voiding, before eating, same scale, same clothing |
| **Alert: Weight gain** | > 1 kg/day or > 2 kg in 3 days → fluid reaccumulation → contact physician |
| **Alert: Rapid weight loss** | Distinguish diuretic-driven fluid loss from muscle catabolism — serial MAMC measurement |
| **Target weight loss** | 0.5 kg/day maximum (ascites); 0.3 kg/day (edema without ascites) via diuretics |

**Hepatic Encephalopathy Prevention Through Diet**

| **Strategy** | **Detail** |
| --- | --- |
| **Optimize protein intake (don't restrict)** | As outlined above; plant > animal protein |
| **BCAA supplementation** | Corrects BCAA:AAA imbalance — direct encephalopathy prevention |
| **High-fiber diet** | 25–35 g/day — reduces colonic ammonia absorption; promotes *Lactobacillus* |
| **Zinc supplementation** | Zinc is a cofactor for ornithine transcarbamylase (urea cycle enzyme) — deficiency impairs ammonia clearance |
| **Lactulose coordination** | Confirm lactulose dose with hepatologist; dietary fiber works synergistically |
| **Avoid constipation** | Adequate hydration and fiber; constipation markedly increases ammonia absorption |

**Gut Microbiome and Intestinal Health**

- Alcoholic cirrhosis causes severe **gut dysbiosis** and **intestinal permeability** ("leaky gut") — contributing to endotoxemia, bacterial translocation, and SBP risk
- Dietary interventions supporting microbiome restoration:
  - **Fermented dairy** (low-sodium yogurt, kefir) — *Lactobacillus* and *Bifidobacterium* support
  - **High dietary fiber** from diverse whole plant foods
  - **Prebiotic foods** (garlic, onion, oats, banana, asparagus)
  - Avoid artificial sweeteners which further disrupt gut microbiota

**Physical Activity**

- **Light structured walking:** 15–20 min/day initially, gradually increasing to 30 min as tolerated
- **Seated resistance exercises** (resistance bands, light weights): preserve and rebuild muscle mass
- Avoid: heavy exertion, Valsalva maneuvers, contact sports if thrombocytopenic or varices present
- Physical activity improves portal pressure, insulin sensitivity, and psychological well-being during alcohol recovery

**Psychosocial and Behavioral Considerations**

- Address **alcohol use disorder with addiction medicine / psychiatry** — nutritional rehabilitation cannot succeed without sustained abstinence support
- Screen for and treat **depression and anxiety** (prevalent in alcohol withdrawal and cirrhosis) — these directly impair appetite and dietary adherence
- Engage family or caregivers in meal preparation and dietary education — social support significantly improves nutritional outcomes
- Consider structured **cooking skill sessions** with a dietitian — patients with long-standing alcohol dependency often have severely disrupted cooking and eating habits

**Summary Priority Action List**

| **Priority** | **Recommendation** |
| --- | --- |
| **#1** | **Complete alcohol abstinence** — engage addiction medicine support immediately |
| **#2** | **Thiamine 100–300 mg/day** — initiate before any glucose-containing nutrition |
| **#3** | **Refeeding precautions** — gradual caloric escalation; monitor electrolytes daily |
| **#4** | **Sodium < 2,000 mg/day** — strict dual fluid overload management |
| **#5** | **Protein ≥ 1.5 g/kg dry weight/day (~80 g/day)** — aggressive malnutrition rehabilitation |
| **#6** | **35–40 kcal/kg dry weight/day (~1,855–2,120 kcal)** — caloric rehabilitation target |
| **#7** | **5–6 meals/day including mandatory late evening snack** |
| **#8** | **BCAA supplementation 12–14 g/day** — sarcopenia and encephalopathy prevention |
| **#9** | **Comprehensive B-complex + folate** + individual thiamine dosing |
| **#10** | **Zinc 50 mg/day, Vitamin D 2,000–4,000 IU/day, Magnesium repletion** |
| **#11** | **Daily weight monitoring** — differentiate fluid shifts from nutritional changes |
| **#12** | **High-fiber, plant-forward diet** — gut microbiome and ammonia control |
| **#13** | **Light aerobic + seated resistance exercise** — graduated program |
| **#14** | **Dietitian follow-up every 2–4 weeks** during active rehabilitation phase |
| **#15** | **Psychosocial support** for alcohol use disorder and depression |

## C210

**Dietary & Nutritional Recommendations**

**Patient:** Female, 67 y/o | HBV Cirrhosis with Ascites | Height: 160 cm | Weight: 52 kg | BMI: 20.3 kg/m²

**1. Overall Dietary Principles**

This patient presents with **decompensated cirrhosis** (Child-Pugh B/C level implied by ascites), placing her at high risk for **protein-energy malnutrition (PEM)** — a critical prognostic factor. Despite the modest BMI appearing near-normal, **sarcopenia and muscle wasting** are highly prevalent and likely underestimated in cirrhotics due to fluid retention masking true lean body mass loss.

**Core goals:**

- Prevent/reverse malnutrition and muscle wasting
- Avoid precipitating **hepatic encephalopathy (HE)**
- Manage **fluid and sodium** to control ascites
- Compensate for poor appetite with caloric density and meal timing strategies

**Use dry/ideal body weight (~54–58 kg estimated) for all calculations**, not actual weight, as ascites falsely elevates measured weight.

**2. Recommended Daily Protein Intake**

| **Parameter** | **Target** |
| --- | --- |
| **Total protein** | **1.2 – 1.5 g/kg/day** (~66–82 g/day based on dry weight ~55 kg) |
| **Caloric intake** | **30–35 kcal/kg/day** (~1,650–1,925 kcal/day) |
| **Protein source priority** | Plant-based ≥ dairy > lean animal protein |

- **Do NOT restrict protein** to prevent HE — this is an outdated practice that accelerates sarcopenia and worsens outcomes
- **BCAA (branched-chain amino acid)-enriched formulas** should be considered if HE episodes occur or protein tolerance is poor
- If overt HE is present, **temporarily** moderate animal protein and favor **vegetable + dairy proteins**, then gradually restore full protein intake

**3. Meal Frequency & Eating Pattern**

Given her **poor appetite and ascites** (which causes early satiety via gastric compression), meal patterning is critical:

Breakfast	→	Mid-Morning	→	Lunch	→	Afternoon	→	Dinner	→	★ Late Evening Snack

~300 kcal	~150 kcal	~350 kcal	~200 kcal	~350 kcal	~200–300 kcal

**4–6 small meals per day** rather than 2–3 large meals **Late-evening snack (LES) is mandatory** — ideally **200–300 kcal with complex carbohydrates ± protein** (e.g., whole grain crackers with low-fat yogurt or a BCAA supplement)

**Why LES matters:** Cirrhotics enter a fasted, catabolic state much faster than healthy individuals (~12 hours' worth of starvation in just 3–4 hours overnight). LES reduces overnight gluconeogenesis from muscle and significantly improves nitrogen balance.

**4. Food Recommendations & Restrictions**

**Recommended Foods**

| **Category** | **Examples** |
| --- | --- |
| **Plant proteins** | Tofu, edamame, lentils, chickpeas, mung beans |
| **Dairy proteins** | Low-fat yogurt, cottage cheese, eggs (1–2/day) |
| **Complex carbohydrates** | Oatmeal, brown rice, whole grain bread, sweet potato |
| **Healthy fats** | Olive oil, avocado (small portions), walnuts |
| **Fruits & vegetables** | Soft-cooked or low-potassium options; avoid raw/hard textures if varices suspected |
| **BCAA supplements** | Granule or oral solution form if food intake is insufficient |

**Dietary Restrictions**

| **Restriction** | **Rationale** |
| --- | --- |
| **Sodium: ≤ 2,000 mg/day (≤ 5 g salt)** | Essential for ascites management; avoid processed foods, canned goods, soy sauce, pickled items |
| **Fluid: 1,000–1,500 mL/day** | Only if dilutional hyponatremia (Na⁺ <125 mEq/L) is present; do not restrict routinely |
| **Alcohol: Absolute zero** | Ongoing hepatotoxicity even with HBV etiology |
| **Raw shellfish / undercooked meat** | High risk of *Vibrio* and bacterial infection in immunocompromised cirrhotic patients |
| **High-ammonia foods** | Limit red meat portions if HE history; avoid gelatin-heavy broths |
| **Hard/sharp-textured foods** | Caution if esophageal varices present (risk of mucosal trauma) |

**5. Additional Nutritional Considerations**

**Micronutrient Supplementation**

Cirrhosis causes fat-soluble vitamin malabsorption and widespread deficiencies:

| **Nutrient** | **Action** |
| --- | --- |
| **Zinc** | Supplement (zinc acetate 50 mg/day) — deficiency worsens HE and taste perception (contributing to poor appetite) |
| **Vitamin D + Calcium** | Supplement routinely; hepatic osteodystrophy risk is high in elderly female |
| **Vitamin B complex (B1, B6, B12, folate)** | Supplement; commonly depleted |
| **Magnesium** | Monitor if on diuretics (spironolactone/furosemide) |
| **Fat-soluble vitamins (A, E, K)** | Check levels; supplement if deficient, but avoid excess Vitamin A (hepatotoxic) |

**Appetite & Practical Strategies**

- **Zinc supplementation** may improve dysgeusia (altered taste) and appetite
- **Small, calorie-dense meals** — prioritize nutrient density over volume
- **Oral nutritional supplements (ONS)** (e.g., hepatic formula like Fresubin Hepa® or Ensure®) if dietary intake remains < 25 kcal/kg/day
- **Nasogastric feeding** should be considered if oral intake is persistently inadequate (< 60% of target for > 5–7 days)
- Serve meals at **preferred temperature and texture** to maximize palatability

**Monitoring Schedule**

| **Parameter** | **Frequency** |
| --- | --- |
| Body weight (dry) + abdominal girth | Weekly |
| Serum albumin, prealbumin | Every 4–6 weeks |
| Electrolytes (Na⁺, K⁺, Mg²⁺) | Every 2–4 weeks (especially if on diuretics) |
| Hand grip strength / DEXA scan | Every 3–6 months |
| Dietary intake assessment | Each clinic visit |

**Summary:** This patient's primary nutritional risk is **protein-energy malnutrition masked by ascites**. The cornerstone of management is **adequate protein (1.2–1.5 g/kg), frequent small meals with a mandatory late-evening snack, strict sodium restriction for ascites control, and targeted micronutrient supplementation** — all tailored to her poor appetite and elderly age. Early initiation of oral nutritional supplements is strongly warranted if food intake remains suboptimal.

## C211

**Clinical Nutrition Recommendations**

**Patient:** Male, 71 yo | 169 cm / 78 kg | BMI 27.3 kg/m² **Diagnosis:** MASLD Cirrhosis with Recurrent Ascites (Decompensated)

**Clinical Note on Body Weight:** The recorded weight of 78 kg likely includes ascitic fluid. Estimated dry weight is approximately **70–73 kg**, which should be used as the reference weight for all nutritional calculations below.

**1. Overall Dietary Principles**

This patient requires a carefully balanced approach that addresses **three overlapping priorities**:

| **Priority** | **Rationale** |
| --- | --- |
| **Anti-sarcopenic nutrition** | Elderly + cirrhosis = very high sarcopenia risk; muscle loss worsens prognosis |
| **Sodium restriction** | Essential for ascites control and to reduce diuretic burden |
| **Metabolic optimization** | MASLD etiology requires limiting refined carbohydrates and saturated fat |

- Maintain **adequate caloric intake** — undernutrition is common and dangerous in cirrhosis
- **Do not restrict protein** — the historical practice of protein restriction in cirrhosis is obsolete and harmful
- Favor **complex carbohydrates** over simple sugars, given the MASLD/metabolic background
- Limit **saturated fat and fructose**, which drive hepatic steatosis and inflammation
- Maintain strict **sodium restriction** to help manage ascites

**2. Recommended Daily Protein & Energy Intake**

**Energy**

- **Target:** 30–35 kcal/kg/day (based on estimated dry weight ~72 kg)
- **Daily goal: ~2,150–2,520 kcal/day**
- Avoid excess calories given overweight status and metabolic liver disease, but do not restrict below 2,100 kcal

**Protein**

- **Target:** 1.2–1.5 g/kg/day (EASL/ESPEN guidelines for cirrhosis)
- **Daily goal: ~85–110 g protein/day**
- Prioritize **high-quality, complete protein sources** at each meal
- If hepatic encephalopathy (HE) episodes occur: do **not** restrict protein — instead consider **branched-chain amino acid (BCAA) supplementation** (leucine-enriched or BCAA formulas 12–15 g/day) as a protein-sparing strategy and to optimize ammonia metabolism
- **Plant-based proteins** (legumes, soy) are encouraged as they produce less ammonia per gram vs. red meat, and align with MASLD management

**Carbohydrates & Fat**

- Carbohydrates: ~50–55% of total calories — emphasize **complex, low-GI sources**
- Fat: ~25–30% — favor **monounsaturated and omega-3 fats**; limit saturated fat to <7% of calories

**3. Meal Frequency & Eating Pattern**

Cirrhotic patients have a **shortened fasting tolerance** (equivalent to a healthy person fasting 2–3 days overnight) due to depleted hepatic glycogen. Prolonged fasting accelerates muscle catabolism.

**Recommended Pattern: 4–6 small meals per day**

Breakfast	~7:00 AM	— Do not skip; include protein + complex carbs

Mid-morning	~10:00 AM	— Light protein snack

Lunch	~12:30 PM	— Largest meal of the day

Afternoon snack ~3:30 PM	— Fruit + protein source

Dinner	~6:30 PM	— Moderate meal; reduce sodium-heavy foods

⭐ Late Evening Snack (LES) ~9:30–10:00 PM — CRITICAL (see below)

**⭐ Late Evening Snack (LES) — High Priority**

- Strongly recommended by ESPEN/EASL for all cirrhotic patients
- **Composition:** 50 g complex carbohydrates + protein
- **Examples:** Whole grain toast with peanut butter and a glass of milk; oatmeal with nuts; rice ball with tofu or egg
- **Benefit:** Reduces overnight gluconeogenesis from muscle, directly preventing sarcopenia

**4. Specific Food Recommendations & Restrictions**

**Encouraged Foods**

| **Category** | **Examples** |
| --- | --- |
| **Lean protein** | Chicken breast, fish (especially salmon/mackerel for omega-3), tofu, eggs, low-fat dairy |
| **Plant protein** | Lentils, edamame, chickpeas, soy milk — reduce ammonia vs. red meat |
| **Complex carbs** | Oatmeal, brown rice, sweet potato, whole grain bread, barley |
| **Healthy fats** | Olive oil (primary cooking fat), avocado, walnuts, flaxseed |
| **Vegetables** | All fresh/frozen non-pickled vegetables; high potassium options (spinach, broccoli) if on diuretics |
| **Coffee** | 2–3 cups/day — evidence supports hepatoprotective effect in MASLD cirrhosis |

**Foods to Restrict or Avoid**

| **Category** | **Reason** |
| --- | --- |
| **High-sodium foods** | Soy sauce, pickled/cured foods, deli meats, canned soups, fast food — worsens ascites |
| **Added table salt** | Remove salt shaker; use herbs, lemon, garlic for flavoring |
| **Alcohol** | Strictly prohibited — directly hepatotoxic |
| **Sugar-sweetened beverages, fruit juice, fructose corn syrup** | Drives hepatic steatosis (MASLD mechanism) |
| **Red and processed meat** | High ammonia generation; increases cardiovascular and oncologic risk |
| **Raw shellfish (oysters, clams)** | Risk of *Vibrio vulnificus* — life-threatening in cirrhosis |
| **Grapefruit** | Inhibits CYP3A4 — may interact with medications |
| **High-fat fried foods** | Worsens MASLD and dyslipidemia |

**Sodium Target**

- **< 2,000 mg sodium/day** (≈ 5 g table salt equivalent)
- Read food labels carefully; most processed and restaurant food exceeds this rapidly
- Home cooking is strongly preferred

**5. Additional Nutritional Considerations**

**Micronutrient Monitoring & Supplementation**

| **Nutrient** | **Concern in this Patient** | **Recommendation** |
| --- | --- | --- |
| **Zinc** | Commonly deficient in cirrhosis; cofactor for ammonia metabolism | Supplement 25–50 mg/day if serum zinc low; zinc-rich foods (pumpkin seeds, legumes) |
| **Vitamin D** | Frequently deficient; critical for muscle and bone health in elderly | Check 25-OH vitamin D; supplement if <30 ng/mL (typically 1000–2000 IU/day) |
| **B vitamins (B1, B6, B12, Folate)** | Impaired hepatic storage and activation | B-complex supplementation reasonable; avoid mega-doses |
| **Magnesium** | Diuretic therapy (spironolactone, furosemide) causes depletion | Monitor serum levels; dietary sources: nuts, seeds, leafy greens |
| **Potassium** | Diuretic use increases loss | Monitor closely; increase dietary sources if on loop diuretics |
| **Fat-soluble vitamins (A, E, K)** | Malabsorption if cholestatic component | Monitor; supplement cautiously (avoid excess Vitamin A — hepatotoxic) |

**Iron supplementation is NOT recommended** unless confirmed iron-deficiency anemia — excess iron promotes hepatic oxidative stress in MASLD.

**Physical Activity**

- Light to moderate **resistance exercise** (e.g., resistance bands, bodyweight exercises) 3–4x/week is recommended alongside nutrition to combat sarcopenia
- Even 20–30 min of **walking daily** improves muscle mass and insulin sensitivity
- Avoid high-impact activity if significant ascites is present

**Nutritional Assessment & Follow-Up**

- Consider **dietitian-led DEXA scan or bioelectrical impedance** to assess true muscle mass (BMI is unreliable with ascites)
- Reassess **hand grip strength** at each visit as a proxy for sarcopenia
- Recheck labs every 3 months: albumin, prealbumin, zinc, vitamin D, electrolytes
- **BCAA oral supplement** (e.g., Livact® or equivalent) if sarcopenia is confirmed or HE recurs

**Summary Card for Patient**

- Eat **4–6 small meals daily** — never skip breakfast, never fast overnight
- Have a **bedtime snack** every night (e.g., whole grain toast + egg or peanut butter)
- Target **~90–100 g protein/day** from lean meats, fish, eggs, beans, and tofu
- Strictly **no alcohol**, **no raw shellfish**
- Keep sodium **under 2,000 mg/day** — cook at home, avoid processed food
- Use **olive oil**; avoid sugar drinks and fried foods
- Take **Vitamin D and Zinc** as directed; ask about BCAA supplement
- Stay active with gentle **daily walking and light resistance exercise**

## C212

**Clinical Nutrition Consultation**

**Patient: Female, 74 y/o | Alcohol-Related Cirrhosis with Ascites**

**Key Clinical Assessment Note**

**BMI = 26.3 kg/m²** (64 kg ÷ 1.56²) — however, this is likely **falsely elevated** due to ascites. True "dry weight" is estimated lower (~58–60 kg). All nutritional calculations should be based on **estimated dry weight or ideal body weight (~49–52 kg)**, not current weight.

**1. Overall Dietary Principles**

| **Principle** | **Rationale** |
| --- | --- |
| **Sodium restriction: ≤ 2,000 mg/day** | Reduces ascites accumulation and fluid retention |
| **Caloric target: 30–35 kcal/kg/day** (dry weight) | ~1,500–1,800 kcal/day to prevent malnutrition |
| **Absolute alcohol abstinence** | Continued alcohol accelerates fibrosis and decompensation |
| **Avoid prolonged fasting (> 4–6 hours)** | Cirrhotic liver has reduced glycogen stores; fasting triggers protein catabolism |
| **Soft, easily digestible foods** | Reduces risk of variceal irritation; improves tolerance |

**2. Recommended Daily Protein Intake**

**Target: 1.2 – 1.5 g protein/kg dry body weight/day**

Approximately **65–78 g of protein per day**

**Critical points:**

- **Do NOT restrict protein** out of fear of hepatic encephalopathy — this is an outdated practice that worsens sarcopenia and outcomes
- Protein restriction is only indicated during **acute encephalopathy episodes**, and even then, only transiently
- **Branched-chain amino acids (BCAAs)** — leucine, isoleucine, valine — are preferentially recommended. Consider BCAA supplementation (e.g., 12 g/day oral BCAA formula) if adequate dietary intake cannot be achieved
- At age 74, sarcopenia risk is compounded by both cirrhosis and aging → **protein adequacy is essential**

**3. Meal Frequency and Eating Pattern**

**Recommended: 4–6 small meals per day + mandatory late-evening snack (LES)**

Breakfast	~7:00–8:00 AM

Mid-morning	~10:00–10:30 AM

Lunch	~12:00–1:00 PM

Afternoon	~3:00–3:30 PM

Dinner	~6:00–7:00 PM

Late-Evening	~9:00–10:00 PM ← CRITICAL

**Why the Late-Evening Snack Matters:**

- The overnight fast is the **longest fasting period** and most metabolically stressful in cirrhosis
- A **200–300 kcal LES** containing complex carbohydrates + protein significantly reduces muscle catabolism
- **Ideal LES options:** whole grain crackers with peanut butter, a small bowl of oatmeal, or a BCAA-enriched formula drink

**4. Specific Food Recommendations and Restrictions**

**ENCOURAGED Foods**

| **Category** | **Recommended Choices** |
| --- | --- |
| **Protein sources** | Eggs, tofu, low-fat dairy (yogurt, cottage cheese), fish (steamed/baked), skinless poultry, legumes |
| **Carbohydrates** | Oatmeal, brown rice, sweet potato, whole grain bread (low-sodium), fruits |
| **Vegetables** | Fresh or frozen (no added salt): spinach, broccoli, carrots, zucchini |
| **Fats** | Olive oil, avocado (in moderation), nuts/seeds (unsalted) |
| **Fluids** | Water, herbal teas; fluid intake guided by physician based on ascites severity |

**RESTRICTED / AVOIDED Foods**

| **Category** | **Reason** |
| --- | --- |
| **High-sodium foods** | Canned soups, deli meats, pickles, soy sauce, processed snacks, fast food → worsen ascites |
| **Raw shellfish / raw fish** | Risk of *Vibrio vulnificus* — life-threatening in cirrhosis |
| **Alcohol — absolute zero** | Any amount accelerates disease progression |
| **High-fructose corn syrup, sugary drinks** | Worsen hepatic steatosis and metabolic stress |
| **Very high-protein red meat (large portions)** | Higher ammonia load; prefer plant/fish protein |
| **Unpasteurized foods** | Immunocompromised state in cirrhosis increases infection risk |

**5. Additional Nutritional Considerations**

**Micronutrient Deficiencies (Common in Alcoholic Cirrhosis)**

| **Nutrient** | **Issue** | **Action** |
| --- | --- | --- |
| **Thiamine (B1)** | Severely depleted from alcohol use | Supplement: **100 mg/day orally** |
| **Folate (B9)** | Depleted by alcohol and poor intake | Supplement: **1 mg/day** |
| **Zinc** | Deficiency worsens encephalopathy risk | Supplement: **25–45 mg elemental zinc/day** |
| **Vitamin D** | Impaired hepatic 25-hydroxylation | Check 25-OH vitamin D; supplement if deficient (800–2000 IU/day) |
| **Magnesium** | Depleted by alcohol and diuretics | Monitor; supplement as needed |
| **Vitamin K** | Impaired synthesis/absorption | Monitor PT/INR; supplement if clinically indicated |

**If Patient Is on Diuretics (e.g., Spironolactone/Furosemide for Ascites):**

- Monitor **potassium and sodium** levels closely
- Spironolactone is potassium-sparing → **avoid potassium supplements and high-K salt substitutes** unless directed
- Daily weight monitoring at home to track fluid shifts (report >2 kg gain in 2 days)

**Physical Activity**

- Gentle, **low-intensity activity** (e.g., walking 20–30 min/day) is recommended if tolerated
- Helps preserve **muscle mass** and reduces sarcopenic risk
- Avoid strenuous Valsalva maneuvers (heavy lifting) if esophageal varices are present

**Summary Card for Patient**

- **Eat 5–6 small meals/day — never skip the bedtime snack**
- **Keep sodium under 2,000 mg/day — avoid all processed/canned foods**
- **Eat adequate protein every day — do not restrict it**
- **Zero alcohol — ever**
- **No raw seafood**
- **Take prescribed vitamin supplements (especially B1, folate, zinc)**
- **Weigh yourself daily; report sudden weight gain to your doctor**

## C301

**Dietary & Nutritional Recommendations**

**Patient:** Male, 35 y/o | 171 cm / 65 kg | BMI: 22.2 kg/m² **Diagnosis:** Liver cirrhosis, history of hepatic encephalopathy (HE), currently recovered

**1. Overall Dietary Principles**

- **Priority goal:** Prevent malnutrition and sarcopenia, which are highly prevalent in cirrhosis and independently worsen prognosis.
- Maintain a **high-calorie, adequate-protein** diet — protein restriction is **no longer recommended** even with prior HE, as it worsens muscle wasting.
- Avoid **prolonged fasting** (>6 hours overnight), as patients with cirrhosis have accelerated starvation metabolism resembling a 3-day fast in healthy individuals.
- Favor **small, frequent meals** to minimize metabolic stress and reduce the risk of HE recurrence.
- Minimize **dietary triggers** for HE (see below), but do **not** eliminate protein.

**2. Recommended Daily Protein Intake**

| **Goal** | **Target** |
| --- | --- |
| **Total daily protein** | **1.2 – 1.5 g/kg/day** → **78 – 98 g/day** |
| Preferred protein sources | Plant-based + dairy > red meat |
| BCAA supplementation | Consider if HE recurs on standard diet |

**Key point on HE history:** Protein restriction was historically advised but is now contraindicated per EASL/ESPEN guidelines. Sarcopenia itself is a major HE precipitant. The goal is to **optimize protein quality**, not restrict quantity.

- **Branched-chain amino acids (BCAAs)** — leucine, isoleucine, valine — are preferred as they are metabolized by muscle, not the liver, and do not generate ammonia centrally. BCAA-enriched supplements (e.g., 12–15 g BCAA/day) may be used if standard food sources are insufficient or HE recurs.
- **Vegetable and dairy proteins** (tofu, legumes, eggs, low-fat yogurt) produce less ammoniagenic aromatic amino acids compared to red/processed meat.

**3. Meal Frequency and Eating Pattern**

Morning	Mid-morning	Lunch	Afternoon	Dinner	Late-night snack

Breakfast	Small snack	Main meal	Small snack	Main meal	MANDATORY

(key intervention)

- **4–6 small meals per day** rather than 2–3 large meals
- **Late-night snack (LNS) — critical:** A carbohydrate-rich snack of **~50 g complex carbohydrates** (e.g., whole-grain crackers, a small bowl of oatmeal, or a banana with low-fat milk) at **~9–10 PM** is strongly evidence-backed (JPEN, ESPEN guidelines) to:
  - Reduce overnight gluconeogenesis from muscle protein
  - Preserve lean body mass
  - Reduce ammonia production from muscle catabolism
- Avoid fasting periods longer than **4–6 hours** during the day

**4. Specific Food Recommendations and Restrictions**

**Recommended Foods**

| **Category** | **Examples** |
| --- | --- |
| **Protein (preferred)** | Eggs, low-fat dairy (yogurt, cottage cheese), tofu, tempeh, legumes (lentils, chickpeas), fish (salmon, cod), skinless poultry |
| **Complex carbohydrates** | Oats, brown rice, sweet potato, whole-grain bread, quinoa |
| **Healthy fats** | Olive oil, avocado, small amounts of nuts/seeds |
| **Vegetables** | Leafy greens, broccoli, zucchini, carrots (well-cooked if appetite is poor) |
| **Fruits** | Bananas, apples, berries (moderate portions; watch sugar load) |
| **Hydration** | Water, herbal teas; individualize fluid intake based on ascites status |

**Foods to Restrict or Avoid**

| **Item** | **Reason** |
| --- | --- |
| **Alcohol** | Absolute contraindication — accelerates fibrosis and precipitates decompensation |
| **Red/processed meats** | High ammoniagenic amino acids; increase HE risk |
| **High-sodium foods** (processed foods, canned soups, soy sauce, pickled foods) | Promotes sodium retention and ascites/edema — target **<2,000 mg sodium/day** |
| **Raw shellfish/undercooked seafood** | Risk of *Vibrio vulnificus* — life-threatening in cirrhosis |
| **High-fructose corn syrup / sugary beverages** | Promotes hepatic steatosis and metabolic stress |
| **Herbal supplements** (kava, pennyroyal, comfrey, etc.) | Hepatotoxic; many interact with liver metabolism |

**5. Additional Nutritional Considerations**

**Micronutrient Supplementation**

Cirrhosis commonly causes fat-soluble vitamin malabsorption and micronutrient deficiencies:

| **Nutrient** | **Consideration** |
| --- | --- |
| **Zinc** | Often deficient in cirrhosis; zinc deficiency directly impairs urea cycle → increases ammonia. Supplement **25–45 mg elemental zinc/day** if deficient |
| **Vitamin D** | Frequently low; assess and supplement to maintain 25-OH-D >20 ng/mL |
| **B vitamins (B1, B6, B12, folate)** | Monitor and supplement, especially if any alcohol history |
| **Magnesium** | Commonly depleted; monitor levels |
| **Iron** | Do **not** supplement empirically — iron overload worsens liver fibrosis; check ferritin first |

**HE Recurrence Prevention (Nutritional Strategies)**

Given his **prior HE episode**, specific dietary steps to reduce recurrence:

- **Lactulose-compatible diet:** Fiber-rich foods support colonic transit and ammonia clearance
- **Probiotic foods** (yogurt, kefir): May modulate gut microbiome and reduce ammonia-producing bacteria
- **Minimize constipation** through adequate hydration and fiber
- If HE recurs, consider formal **BCAA oral supplementation** (Livact® or equivalent) under medical supervision
- Educate patient on **HE precipitants** to avoid: dehydration, infections, constipation, GI bleeding, benzodiazepines

**Nutritional Monitoring**

- Reassess nutritional status every **1–3 months** using handgrip strength, mid-upper arm circumference (MUAC), or BIA (if no significant ascites)
- A formal **dietitian referral** is strongly recommended for ongoing individualized guidance
- Watch for **refeeding syndrome** if significant malnutrition has been present

**Summary of key takeaways for this patient:** Do NOT restrict protein. Eat 5–6 small meals daily. Always have a late-night snack. Prefer plant/dairy protein over red meat. Avoid all alcohol and raw shellfish. Check and correct zinc and vitamin D. Monitor for HE triggers proactively.

## C302

**Clinical Nutrition Recommendations**

**Patient C3-02** | F, 38 y/o | 158 cm / 53 kg | BMI: 21.2 kg/m² *Alcohol-related cirrhosis with prior hepatic encephalopathy (HE)*

**Clinical Note:** Despite a normal BMI, patients with cirrhosis frequently have **sarcopenia masked by fluid retention or redistribution.** A formal body composition assessment (e.g., handgrip strength, DEXA, or CT L3 muscle index) is strongly recommended. Nutritional risk should not be judged by weight alone.

**1. Overall Dietary Principles**

This patient requires a **high-calorie, high-protein, low-ammonia-burden diet** that simultaneously supports liver regeneration, prevents HE recurrence, and addresses the metabolic consequences of alcohol-related liver disease.

| **Goal** | **Rationale** |
| --- | --- |
| Prevent/reverse sarcopenia | Loss of muscle mass is the single strongest predictor of HE recurrence and mortality in cirrhosis |
| Maintain stable blood glucose | Cirrhotic liver has impaired glycogen storage; fasting leads to rapid muscle catabolism |
| Minimize ammonia load | Prior HE indicates reduced hepatic detoxification capacity |
| Correct micronutrient deficiencies | Alcohol-related disease causes predictable deficiencies (see §5) |
| Achieve **complete alcohol abstinence** | Non-negotiable; continued alcohol use accelerates fibrosis and negates all nutritional intervention |

**Estimated Daily Energy Target:**

- **35–40 kcal/kg/day** = approximately **1,850–2,120 kcal/day**
- Use **dry weight** or **ideal body weight** if ascites/edema is present (not applicable here currently, but monitor)

**2. Recommended Daily Protein Intake**

**Target: 1.2–1.5 g/kg/day → approximately 64–80 g protein/day**

This is a critical and often mismanaged area. The outdated practice of **protein restriction to prevent HE is strongly discouraged** by EASL, AASLD, and ESPEN guidelines — it worsens sarcopenia and paradoxically increases HE risk.

| **Protein Strategy** | **Detail** |
| --- | --- |
| **Total daily target** | 1.2–1.5 g/kg → **~65–80 g/day** |
| **Preferred sources** | Plant-based proteins (legumes, tofu, tempeh) + dairy (whey, yogurt, cheese) — these have lower ammoniagenic amino acid profiles |
| **Branched-Chain Amino Acids (BCAAs)** | Consider BCAA supplementation (e.g., 12–15 g/day) if dietary protein is poorly tolerated or if HE recurs — strong evidence in cirrhosis |
| **Limit** | Red meat and processed meat (high in aromatic amino acids; increased ammonia load) |
| **Vegetable protein advantage** | High fiber content promotes colonic transit, reducing ammonia absorption |

**3. Meal Frequency and Eating Pattern**

This is **one of the most impactful and underutilized interventions** in cirrhosis management.

RECOMMENDED DAILY EATING SCHEDULE

────────────────────────────────────────────────────────

Breakfast	07:00–08:00	Include protein + complex carbs

Mid-morning	10:00–10:30	Small snack (fruit + nuts or yogurt)

Lunch	12:00–13:00	Largest meal of the day

Afternoon	15:30–16:00	Small snack (e.g., whole grain + cheese)

Dinner	18:00–19:00	Moderate-sized, protein-containing meal

★ Late-night	21:00–22:00	MANDATORY bedtime snack

────────────────────────────────────────────────────────

**★ The Late-Night Snack (LNS) — Critical Intervention**

Patients with cirrhosis enter a **catabolic fasting state within 2–3 hours** (vs. 12–16 hours in healthy individuals) due to depleted hepatic glycogen stores. Overnight fasting causes **significant muscle protein breakdown.**

- **Timing:** Within 1–2 hours of sleeping
- **Composition:** 200–250 kcal, **containing 30–40 g complex carbohydrate + 10–15 g protein**
- **Practical options:**
  - Whole grain toast + 2 tablespoons peanut butter + small glass of milk
  - Greek yogurt + granola + banana
  - Oatmeal with nuts and honey
  - BCAA-enriched nutritional supplement drink (e.g., Ensure/commercial BCAA formula)

**Evidence base:** Multiple RCTs demonstrate that a LNS reduces nitrogen loss, improves albumin levels, and reduces HE episodes in cirrhotic patients.

**4. Specific Food Recommendations and Restrictions**

**Encouraged Foods**

| **Category** | **Examples** | **Benefit** |
| --- | --- | --- |
| **Legumes** | Lentils, chickpeas, edamame, tofu | High BCAA, high fiber, low ammonia burden |
| **Dairy** | Greek yogurt, cottage cheese, kefir | Easily absorbed protein; probiotics support gut-liver axis |
| **Eggs** | Whole eggs or egg whites | Complete protein, well tolerated |
| **Complex carbohydrates** | Oats, sweet potato, whole grain bread, brown rice | Sustained glucose release; prevents fasting catabolism |
| **Fatty fish** | Salmon, sardines, mackerel (2–3×/week) | Omega-3 anti-inflammatory; reduces hepatic steatosis |
| **Vegetables** | Broccoli, spinach, kale, carrots | Antioxidants, fiber, folate |
| **Fruits** | Berries, banana, kiwi | Antioxidants, potassium (if not hyperkalemic) |
| **Olive oil** | As primary cooking fat | Monounsaturated fats; anti-inflammatory |

**Foods to Avoid or Limit**

| **Item** | **Reason** |
| --- | --- |
| **All alcohol** | Absolute contraindication — accelerates fibrosis, depletes thiamine/folate, triggers HE |
| **Red and processed meats** | High ammoniagenic amino acids; sulfur-containing compounds worsen HE |
| **High-sodium foods** | Canned goods, fast food, pickles, soy sauce → risk of ascites/fluid retention (even if absent now) |
| **Raw shellfish/oysters** | Risk of *Vibrio vulnificus* — life-threatening in cirrhosis |
| **Raw/undercooked eggs, meat, fish** | Immunocompromised state; food safety is paramount |
| **High-fructose corn syrup / sugary drinks** | Promotes hepatic steatosis and inflammation |
| **Herbal supplements** | Many are hepatotoxic (e.g., kava, comfrey, green tea extract) — avoid unless physician-approved |

**Sodium Guidance**

- **Limit to < 2,000 mg sodium/day** prophylactically (even without current ascites), given cirrhosis and risk of decompensation
- Read labels; avoid processed/packaged foods as a default strategy

**5. Additional Nutritional Considerations**

Given **alcohol-related** etiology, this patient has a highly predictable pattern of micronutrient deficiencies that must be actively addressed:

**Key Micronutrients to Assess and Supplement**

| **Nutrient** | **Rationale** | **Recommendation** |
| --- | --- | --- |
| **Thiamine (B1)** | **PRIORITY** — alcohol depletes thiamine; deficiency causes Wernicke's encephalopathy, which can mimic/compound HE | Supplement **100 mg/day orally** empirically; IV if symptomatic |
| **Folate (B9)** | Depleted by alcohol; essential for cell regeneration | 1 mg/day |
| **Pyridoxine (B6)** | Alcohol-related depletion; involved in amino acid metabolism | 50 mg/day or as part of B-complex |
| **Zinc** | Low zinc → impaired urea cycle → increased ammonia → HE risk; also impairs taste (reduces food intake) | 25–50 mg elemental zinc/day with meals (monitor copper if long-term) |
| **Vitamin D** | Near-universal deficiency in cirrhosis; critical for muscle function and immunity | Check 25-OH vitamin D; supplement 1,000–2,000 IU/day (higher if deficient) |
| **Magnesium** | Commonly low in alcohol-related disease | Supplement if serum level low |
| **Vitamin K** | Impaired hepatic synthesis of clotting factors; deficiency worsens coagulopathy | Supplement if dietary intake poor; monitor INR |

**Hepatic Encephalopathy — Ongoing Dietary Strategies**

| **Strategy** | **Detail** |
| --- | --- |
| **Lactulose compliance** | If prescribed, ensure patient understands dietary fiber supports its action |
| **Gut microbiome support** | Probiotic-rich foods (yogurt, kefir) or probiotic supplements may reduce gut-derived ammonia |
| **Hydration** | Adequate fluid intake prevents constipation, which is a major HE precipitant |
| **Recognize warning signs** | Educate patient: worsening cognition, sleep reversal, and asterixis often precede overt HE — dietary triggers should be reviewed at that time |

**Monitoring and Follow-Up**

- **Dietitian follow-up:** Every 4–8 weeks initially; monthly handgrip strength assessment
- **Labs to monitor:** Albumin, prealbumin, zinc, vitamin D, B12, folate, thiamine, CBC, LFTs, ammonia (if symptomatic)
- **Psychosocial:** Refer to addiction medicine/support services (AA, counseling) — nutritional intervention cannot succeed without sustained sobriety support
- **Consider referral:** Registered Dietitian with hepatology expertise for ongoing individualized counseling

**One-Page Patient Summary (Counseling Talking Points)**

*"Your liver is working harder than normal, so your body needs more fuel and more protein — not less. Eat every 3–4 hours, never skip breakfast, and always have a small snack before bed. Choose beans, dairy, eggs, and fish over red meat. Avoid all alcohol completely. Take your vitamins as prescribed. The goal is to keep your muscles strong, which is your best protection against future brain episodes."*

## C303

**Clinical Nutrition Recommendations**

**Patient C3-03:** Male, 43 y/o | 169 cm / 80 kg | BMI: 28.0 kg/m² (Overweight) **Diagnosis:** MASLD Cirrhosis + Prior Hepatic Encephalopathy (HE)

**1. Overall Dietary Principles**

This patient presents a dual challenge: **metabolic-driven liver disease** requiring cardiometabolic dietary control, combined with **decompensated cirrhosis** requiring adequate nutrition to prevent sarcopenia and HE recurrence.

| **Goal** | **Rationale** |
| --- | --- |
| Moderate caloric management | Overweight BMI on a metabolic background; avoid aggressive restriction |
| Prevent protein malnutrition | Sarcopenia dramatically worsens prognosis in cirrhosis |
| Minimize HE triggers | Prior HE mandates attention to protein source and gut health |
| Reduce metabolic burden | Address underlying insulin resistance/dyslipidemia driving MASLD |
| **Absolute alcohol abstinence** | Non-negotiable; further hepatotoxicity is unacceptable |

**Target energy intake:** ~30–35 kcal/kg actual body weight/day (~2,400–2,800 kcal/day), avoiding both overfeeding and underfeeding.

**2. Recommended Daily Protein Intake**

**Critical point:** Protein restriction for HE is **outdated and harmful**. Current EASL/AASLD guidelines explicitly recommend *adequate* protein intake even with prior HE.

- **Target:** **1.2–1.5 g/kg body weight/day = ~96–120 g protein/day**
- **Preferred protein sources** (to reduce ammoniagenic load):

| **Preferred** | **Limit/Avoid** |
| --- | --- |
| Dairy proteins (whey, yogurt, cheese) | Red meat (high ammonia production) |
| Plant proteins (legumes, tofu, nuts) | Processed meats |
| Eggs | Excessive red meat at a single meal |
| Fish and white-meat poultry |  |

- **Branched-Chain Amino Acid (BCAA) supplementation** (e.g., 12–20 g/day of leucine-enriched BCAA): Consider if oral intake is suboptimal or if HE recurs on adequate protein intake.

**3. Meal Frequency and Eating Pattern**

Cirrhotic livers have severely limited glycogen storage. Prolonged fasting accelerates muscle catabolism.

Recommended Schedule (4–6 meals/day):

Breakfast (7–8 AM)	──► Moderate carb + protein

Mid-morning snack	──► Fruit + dairy or nuts

Lunch (12–1 PM)	──► Main protein source + vegetables

Afternoon snack (3 PM) ──► Complex carb + protein

Dinner (6–7 PM)	──► Lighter protein + vegetables

★ Late Evening Snack	──► 200–250 kcal carbohydrate-rich snack

(within 2–3 hrs of sleep)	(e.g., whole grain toast, oatmeal, banana)

**★ Late Evening Snack (LES) is ESSENTIAL** — reduces overnight fasting duration, preserves muscle mass, and decreases risk of hypoglycemia. This is a Level A recommendation in cirrhosis management.

**4. Specific Food Recommendations and Restrictions**

**Recommended Foods**

- **Complex carbohydrates:** Oats, brown rice, sweet potato, whole grain bread (favor low glycemic index, given MASLD/insulin resistance)
- **Vegetables:** All non-starchy vegetables freely; aim for ≥400 g/day
- **Fruits:** 1–2 servings/day (limit high-fructose fruits like grapes, mangoes, given MASLD)
- **Dairy:** Low-fat yogurt, cottage cheese, milk — excellent protein with low ammonia burden
- **Legumes:** Lentils, chickpeas, edamame — dual benefit of protein + fiber (supports gut microbiome, reduces ammonia)
- **Fish:** 2–3 servings/week (omega-3 benefit for MASLD)
- **Healthy fats:** Olive oil (primary fat source), avocado, small handful of nuts

**Foods to Restrict or Avoid**

| **Category** | **Reason** |
| --- | --- |
| **Alcohol** | Absolute contraindication |
| **Added sugars / fructose / SSBs** | Drives MASLD progression and insulin resistance |
| **Ultra-processed foods** | Pro-inflammatory, metabolic burden |
| **Red and processed meats** | High ammoniagenic potential → HE risk |
| **High-sodium foods** | Risk of fluid retention / ascites |
| **Raw shellfish** | *Vibrio vulnificus* risk — potentially fatal in cirrhosis |
| **Saturated and trans fats** | Worsens hepatic steatosis |

**Sodium:** Limit to **<2,000 mg/day** if any evidence of fluid retention; monitor closely.

**5. Additional Nutritional Considerations**

**Micronutrient Monitoring & Supplementation**

| **Nutrient** | **Relevance** | **Action** |
| --- | --- | --- |
| **Zinc** | Commonly deficient in cirrhosis; cofactor in urea cycle (ammonia metabolism) | Consider supplementation 25–50 mg/day elemental zinc |
| **Vitamin D** | Almost universally deficient in MASLD cirrhosis | Check 25-OH Vit D; supplement to maintain >30 ng/mL |
| **B vitamins (B1, B6, B12, folate)** | Impaired hepatic storage and metabolism | Multivitamin or targeted supplementation |
| **Magnesium** | Frequently low; important for insulin sensitivity | Dietary and/or supplemental |
| **Iron** | Do NOT supplement empirically — risk of iron overload in cirrhosis | Check ferritin/TSAT first |
| **Fat-soluble vitamins (A, E, K)** | Impaired bile acid-dependent absorption | Assess individually; avoid excess Vit A (hepatotoxic) |

**HE Prevention Strategy**

- Ensure **regular bowel movements** (1–2/day): Adequate fiber (25–35 g/day), hydration, lactulose if prescribed
- **Probiotics** (e.g., *Lactobacillus* species): Emerging evidence for reducing ammonia via gut microbiome modulation
- **Avoid constipation** — a major precipitant of HE recurrence
- Monitor for and treat **muscle cramps** (common in cirrhosis): adequate sodium, magnesium, and hydration

**Weight Management in MASLD Cirrhosis**

- **Target:** Modest weight loss of **0.5–1 kg/week** if well-compensated; avoid aggressive restriction in decompensated disease
- A **5–10% total body weight reduction** can reduce hepatic steatosis and inflammation
- Avoid crash dieting or very low-calorie diets — precipitates malnutrition and muscle loss in cirrhosis

**Follow-Up**

- **Dietitian follow-up:** Every 1–3 months; reassess nutritional status (hand grip strength, mid-arm muscle circumference)
- **Lab monitoring:** LFTs, albumin, prealbumin, zinc, Vit D, CBC, metabolic panel
- **Screen for sarcopenia:** CT L3 skeletal muscle index or bedside assessments — present in up to 70% of cirrhotic patients regardless of BMI

## C304

**Clinical Nutrition Recommendations**

**Patient: Female, 44 y/o | HBV Cirrhosis — Hepatic Encephalopathy Recovery Phase**

**BMI: 26.6 kg/m² (Height 160 cm / Weight 68 kg)**

**Clinical Note:** Despite a normal-to-slightly-overweight BMI, patients with cirrhosis are at high risk for **sarcopenic obesity** — reduced muscle mass masked by fluid retention or fat. Nutritional assessment should not rely on weight alone.

**1. Overall Dietary Principles**

| **Principle** | **Rationale** |
| --- | --- |
| **Do NOT restrict protein** | Outdated practice; protein restriction worsens sarcopenia and HE outcomes |
| **Energy-adequate diet** | Target **30–35 kcal/kg/day** (~2,040–2,380 kcal/day) |
| **Avoid prolonged fasting** | Fasting >6 hours accelerates protein catabolism in cirrhosis |
| **Low-ammonia dietary pattern** | Reduce red meat; favor plant and dairy protein sources |
| **Sodium moderation** | 2,000–2,400 mg/day if ascites is present or developing |
| **Abstain from alcohol** | Absolute contraindication |

**2. Recommended Daily Protein Intake**

**Target: 1.2 – 1.5 g/kg/day = approximately 82–102 g protein/day**

This is consistent with **ESPEN 2019** and **AASLD** guidelines for cirrhosis, including patients in HE recovery.

**Protein Source Prioritization:**

PREFERRED: Vegetable protein (legumes, soy, tofu)

Dairy protein (yogurt, eggs, cheese)

Fish / poultry (lean, well-cooked)

LIMIT: Red meat & processed meat

(higher ammoniagenic amino acid profile)

AVOID: Raw/undercooked proteins (infection risk in cirrhosis)

**Branched-Chain Amino Acids (BCAAs):**

- **Strongly consider BCAA supplementation** (leucine, isoleucine, valine)
- Dose: **BCAA-enriched formula 12–15 g/day** or oral BCAA supplement
- Benefits in HE recovery: reduces ammonia metabolism burden, improves nitrogen balance, supports muscle synthesis
- Evidence-based in Japanese/Asian cirrhosis guidelines

**3. Meal Frequency and Eating Pattern**

Given the impaired glycogen storage and accelerated fasting metabolism in cirrhosis:

RECOMMENDED DAILY MEAL PATTERN

─────────────────────────────────────────────────────

Breakfast	7:00–8:00 AM	(Do not skip)

Mid-morning snack 10:00–10:30 AM

Lunch	12:00–1:00 PM

Afternoon snack	3:00–4:00 PM

Dinner	6:00–7:00 PM	(Not too late)

Late-evening snack 9:00–10:00 PM	← CRITICAL

─────────────────────────────────────────────────────

**⭐ Late-Evening Snack (LES) — Key Recommendation**

- Timing: **within 3 hours of sleep**
- Content: **complex carbohydrates + protein**
- Examples:
  - Whole grain crackers + low-fat cheese
  - Small bowl of oatmeal with milk
  - Soy milk + 1–2 slices whole-grain bread
- **Purpose:** Prevents overnight fasting catabolism, maintains muscle mass, reduces ammonia production from muscle breakdown

**4. Specific Food Recommendations and Restrictions**

**Recommended Foods**

| **Category** | **Examples** |
| --- | --- |
| **Carbohydrates** | Oats, brown rice, whole grain bread, sweet potato, congee |
| **Protein (preferred)** | Tofu, soy products, eggs, low-fat yogurt, fish, chicken breast |
| **Vegetables** | All cooked vegetables; spinach, broccoli, carrots, squash |
| **Fruits** | Bananas, apples, pears — moderate portions |
| **Fats** | Olive oil, flaxseed oil — small amounts |
| **Fluids** | Water, herbal teas; monitor if ascites present |

**Foods to Avoid or Restrict**

| **Category** | **Reason** |
| --- | --- |
| **Red meat, organ meats** | High ammoniagenic amino acids → risk of HE relapse |
| **High-sodium foods** | Pickles, soy sauce, cured meats, instant noodles — worsen fluid retention |
| **Raw shellfish / sushi** | Risk of *Vibrio vulnificus* or *Listeria* — life-threatening in cirrhosis |
| **High-fat / fried foods** | Impaired fat metabolism, risk of steatorrhea |
| **Simple sugars / sugary drinks** | Risk of hepatic steatosis, blood sugar instability |
| **Alcohol** | Absolute contraindication |
| **Herbal supplements** | Many are hepatotoxic (e.g., kava, comfrey, green tea extract) |

**5. Additional Nutritional Considerations**

**Micronutrient Monitoring & Supplementation**

| **Nutrient** | **Consideration** |
| --- | --- |
| **Zinc** | Commonly deficient in cirrhosis; zinc deficiency worsens HE; supplement 25–50 mg/day elemental zinc if deficient — zinc is cofactor for urea cycle enzymes |
| **Vitamin D** | Monitor serum 25-OH vitamin D; deficiency very common in HBV cirrhosis; supplement 800–2,000 IU/day as indicated |
| **B vitamins (B1, B6, B12, Folate)** | Often depleted; consider B-complex supplement |
| **Magnesium** | Monitor especially if on diuretics |
| **Fat-soluble vitamins (A, E, K)** | Risk of deficiency if cholestasis present; monitor and supplement cautiously |

**HE-Specific Nutritional Considerations (Recovery Phase)**

- **Lactulose compliance:** Continue prescribed lactulose to maintain 2–3 soft stools/day; reduces colonic ammonia absorption
- **Constipation prevention:** High fiber intake (via vegetables, whole grains) — crucial, as constipation is a major HE precipitant
- **Hydration:** Adequate fluid intake (~1,500–2,000 mL/day unless fluid restriction is needed)
- **Monitor for precipitating factors** through diet: dehydration, GI bleeding, excessive dietary protein load all can re-trigger HE

**Monitoring Parameters to Follow Up**

□ Serum albumin & prealbumin (nutritional status)

□ Serum ammonia (HE monitoring)

□ Serum zinc, Vitamin D, Mg levels

□ Hand-grip strength or 6-minute walk test (sarcopenia screening)

□ Anthropometrics: mid-arm circumference, triceps skinfold

□ Child-Pugh / MELD score trend

**Summary Card for Patient**

**Eat 5–6 small meals per day — never skip the bedtime snack** **Eat 82–100g of protein daily — favor eggs, tofu, fish, dairy over red meat** **Keep salt low — avoid pickles, processed foods, soy sauce** **Eat cooked vegetables daily to prevent constipation** **Ask your doctor about zinc and Vitamin D supplements** **No alcohol. No raw seafood. No herbal supplements without physician approval**

## C305

**Dietary & Nutritional Recommendations**

**Patient:** Male, 49 y/o | 174 cm / 90 kg | BMI ≈ 29.7 kg/m² **Diagnosis:** Alcoholic cirrhosis with prior hepatic encephalopathy (HE)

**1. Overall Dietary Principles**

This patient presents a nuanced challenge: he is **overweight (BMI ~29.7)** yet has cirrhosis — a state of **obligate catabolism and sarcopenia risk**. The instinct to restrict calories for weight loss must be resisted. Priorities are:

- **Prevent/treat sarcopenia** — muscle wasting is the dominant mortality risk in cirrhosis, regardless of excess body weight
- **Support hepatic regeneration** while avoiding metabolic stressors
- **Minimize triggers for hepatic encephalopathy** — without unnecessarily restricting protein
- **Absolute alcohol abstinence** — non-negotiable; any alcohol consumption will accelerate hepatocellular injury
- **Achieve adequate but not excessive energy intake** — target approximately **30–35 kcal/kg of ideal body weight/day** (not actual weight, to avoid overfeeding). Ideal BW ≈ 69–74 kg → roughly **2,100–2,500 kcal/day**

**2. Recommended Daily Protein Intake**

**The outdated practice of protein restriction in HE patients is harmful and should be abandoned** (EASL/ESPEN guidelines).

| **Recommendation** | **Target** |
| --- | --- |
| **Daily protein intake** | **1.2 – 1.5 g/kg ideal body weight/day** |
| For this patient (IBW ~70 kg) | **~85–105 g protein/day** |
| Preferred protein sources | Plant-based > dairy > lean poultry/fish |

**Rationale for HE history:**

- Branched-chain amino acids (BCAAs) — found in plant proteins, dairy, and supplements — are preferentially metabolized in muscle, reducing ammoniagenic load
- Aromatic amino acids (AAAs), which compete with BCAAs for CNS transport and worsen HE, are enriched in red meat → **limit red and processed meat**
- If encephalopathy recurs, **BCAA supplementation (9–15 g/day)** is evidence-based and preferable to protein restriction

**3. Meal Frequency and Eating Pattern**

This is one of the most impactful, yet underappreciated, interventions in cirrhosis:

**Recommended Pattern: 4–6 Small Meals + Mandatory Late-Evening Snack (LES)**

| **Time** | **Meal** |
| --- | --- |
| ~7:00 AM | Breakfast |
| ~10:00 AM | Mid-morning snack |
| ~1:00 PM | Lunch |
| ~4:00 PM | Afternoon snack |
| ~7:00 PM | Dinner |
| **~9:30–10:00 PM** | **Late-evening snack (LES) — critical** |

**Why the late-evening snack matters:** The cirrhotic liver cannot maintain adequate glycogen stores. After just 2–3 hours of fasting, the body shifts to gluconeogenesis from muscle protein — accelerating sarcopenia. The LES **shortens the overnight fast** and significantly preserves muscle mass.

**Ideal LES content:** 200–250 kcal with **complex carbohydrates + protein**

- Examples: whole grain crackers with cottage cheese, a small bowl of oatmeal with milk, or a BCAA-enriched supplement drink

**4. Specific Food Recommendations and Restrictions**

**Encouraged Foods**

| **Category** | **Examples** |
| --- | --- |
| **Plant proteins** | Lentils, chickpeas, edamame, tofu, tempeh |
| **Dairy proteins** | Low-fat yogurt, cottage cheese, eggs, skim milk |
| **Lean animal protein** | Chicken breast, turkey, white fish (salmon, cod) |
| **Complex carbohydrates** | Oats, brown rice, quinoa, sweet potato, whole grain bread |
| **Vegetables** | All varieties — especially leafy greens (folate), brassicas |
| **Fruits** | Moderate portions; limit high-sugar juices |
| **Healthy fats** | Olive oil, avocado, small amounts of nuts/seeds |

**Foods to Restrict or Avoid**

| **Category** | **Reason** |
| --- | --- |
| **All alcohol** | Directly hepatotoxic; accelerates cirrhosis progression |
| **Red and processed meat** | High AAA content, ammoniagenic; increases HE risk |
| **High-sodium foods** | Risk of ascites/edema — target **< 2,000 mg sodium/day** |
| **Raw shellfish** | Risk of *Vibrio vulnificus* sepsis — potentially fatal in cirrhosis |
| **High-fructose/added sugar foods** | Worsen hepatic steatosis and metabolic stress |
| **Very high-fiber supplements (bran)** | Can impair drug absorption; use dietary fiber instead |
| **Herbal supplements/hepatotoxic remedies** | Many are directly hepatotoxic (e.g., kava, comfrey, green tea extract) |

**5. Additional Nutritional Considerations**

**Micronutrient Deficiencies — Highly Prevalent in Alcoholic Cirrhosis**

| **Nutrient** | **Clinical Concern** | **Action** |
| --- | --- | --- |
| **Thiamine (B1)** | Wernicke's encephalopathy risk | **Supplement: 100 mg/day oral** (or IV if acute) |
| **Folate (B9)** | Megaloblastic anemia, common in alcoholism | Supplement: 1 mg/day |
| **Pyridoxine (B6)** | Peripheral neuropathy | Supplement as part of B-complex |
| **Zinc** | Deficiency worsens HE; impairs ammonia metabolism | Supplement: **zinc acetate 50 mg/day** if deficient |
| **Vitamin D** | Nearly universal in cirrhosis; linked to bone disease and immune function | Check 25-OH Vit D; supplement to achieve 30–50 ng/mL |
| **Magnesium** | Often depleted in alcoholism | Monitor and replete PRN |
| **Vitamin K** | Fat-soluble; malabsorption worsens coagulopathy | Supplement if prolonged PT not explained by synthetic failure |

**Lactulose and Diet**

If the patient is on **lactulose** for HE prophylaxis/treatment, dietary consistency is important — irregular fiber and carbohydrate intake can make lactulose titration unpredictable. Adequate **fluid intake (1.5–2 L/day, unless fluid-restricted due to ascites)** is essential to prevent dehydration from lactulose-induced diarrhea.

**Weight Management Caution**

Despite a BMI of 29.7, **intentional caloric restriction and weight loss programs are contraindicated** unless carefully supervised. Rapid weight loss accelerates sarcopenia and can precipitate acute-on-chronic liver failure. Any weight reduction, if clinically warranted, should be **gradual, protein-preserving, and monitored** with serial assessment of muscle mass (e.g., hand-grip strength, DEXA, CT psoas index).

**Follow-Up Monitoring Recommendations**

- **Regular dietary review** every 3–6 months with a registered dietitian familiar with liver disease
- Monitor **serum ammonia, albumin, pre-albumin, zinc, and vitamin D** at each follow-up
- **Nutrition screening tool**: Royal Free Hospital Global Assessment (RFH-GA) or NUTRIC score
- Assess for **sarcopenic obesity** (normal/excess fat mass + muscle depletion) — common and easily missed in this patient's weight category

**Summary Priority List:**

1. Zero alcohol — reinforce at every visit
2. Do not restrict protein — 1.2–1.5 g/kg IBW/day
3. Eat every 3–4 hours; never skip the late-evening snack
4. Favor plant and dairy proteins over red meat
5. Supplement thiamine, folate, zinc, and vitamin D
6. Sodium < 2,000 mg/day; no raw shellfish

## C306

**Clinical Nutrition Recommendations for Cirrhosis Patient**

**Patient Summary:** Female, 53 y/o | 157 cm | 58 kg | BMI: 23.5 kg/m² **Diagnosis:** MASLD Cirrhosis | Prior Hepatic Encephalopathy | Mild Weight Loss

**Clinical Context Before Recommendations**

Despite a seemingly normal BMI, this patient is at **high risk for sarcopenic obesity** — a condition common in MASLD cirrhosis where visceral fat coexists with muscle loss. The mild weight loss likely reflects lean mass loss rather than fat loss, which is a **poor prognostic indicator** and an independent risk factor for recurrent hepatic encephalopathy (HE). Nutrition therapy is therefore a **core treatment**, not merely adjunctive.

**1. Overall Dietary Principles**

| **Principle** | **Rationale** |
| --- | --- |
| **High-protein, calorie-adequate diet** | Prevent/reverse sarcopenia; support hepatic regeneration |
| **Do NOT protein-restrict** | Outdated practice; protein restriction worsens outcomes in cirrhosis with HE |
| **Anti-inflammatory, low-saturated fat** | Addresses underlying MASLD pathophysiology |
| **Low simple sugar & refined carbohydrate** | Reduces hepatic lipogenesis and insulin resistance (MASLD-specific) |
| **Avoid prolonged fasting** | Prevents catabolic gluconeogenesis from muscle |
| **Sodium moderation** | Risk of ascites management (even if currently absent) |

**2. Recommended Daily Protein Intake**

**Target: 1.2 – 1.5 g of protein per kg of body weight per day** → **For this patient: approximately 70 – 87 g protein/day**

**Key Points on Protein:**

- **Do not restrict protein** due to prior HE. Modern guidelines (EASL, ESPEN 2019) confirm adequate protein *reduces* HE risk by preventing sarcopenia
- **Prefer plant-based and dairy protein sources** over red meat:
  - Plant proteins (legumes, soy, tofu) produce less ammonia per gram than animal proteins
  - Dairy (yogurt, cheese, milk) is well-tolerated and rich in branched-chain amino acids (BCAAs)
- **Branched-chain amino acids (BCAAs):** Consider BCAA-enriched foods or supplementation (leucine, isoleucine, valine) if dietary target is consistently unmet — BCAAs have evidence for reducing HE recurrence

**Practical Protein Targets:**

| **Meal** | **Protein Goal** | **Example** |
| --- | --- | --- |
| Breakfast | ~15–20 g | 2 eggs + 1 cup Greek yogurt |
| Lunch | ~20–25 g | 90g tofu + ½ cup lentils |
| Dinner | ~20–25 g | 90g salmon or chicken |
| Late-night snack | ~15 g | 1 cup cottage cheese or casein-rich snack |

**3. Meal Frequency and Eating Pattern**

This is **critically important** for cirrhotic patients due to impaired glycogen storage.

**Recommended Pattern: 4–6 Small Meals Per Day**

7:00 AM	→ Breakfast (with protein)

10:00 AM → Mid-morning snack

12:30 PM → Lunch (largest meal)

3:30 PM	→ Afternoon snack

6:30 PM	→ Dinner

9:30 PM	→ ★ LATE-NIGHT SNACK (non-negotiable)

**★ The Late-Night Snack (LNS) — Most Critical Recommendation**

- **Evidence-based, guideline-recommended** for all cirrhotic patients
- The cirrhotic liver has ~50% reduced glycogen storage capacity; without LNS, the patient enters a prolonged fasting catabolic state overnight
- **Ideal LNS:** Complex carbohydrate + protein (e.g., whole grain crackers with cottage cheese, or a small bowl of oatmeal with milk)
- **Target:** 200–250 kcal, ≥15 g protein

**4. Specific Food Recommendations and Restrictions**

**Encouraged Foods**

**Protein Sources (Low Ammonia-Generating):**

- Eggs, Greek yogurt, cottage cheese, ricotta
- Tofu, edamame, tempeh, lentils, chickpeas
- Fish (salmon, sardines, tuna) — 3–4×/week
- Skinless poultry in moderation

**Carbohydrates (Complex, Low-Glycemic Index):**

- Oats, quinoa, brown rice, sweet potato
- Legumes (dual benefit: protein + fiber)
- Whole grain bread and crackers

**Fats (Anti-Inflammatory):**

- Olive oil as primary cooking fat (extra virgin preferred)
- Avocado, walnuts, flaxseed (omega-3 rich)
- Fatty fish (EPA/DHA — reduces hepatic inflammation in MASLD)

**Vegetables & Fruits:**

- Cruciferous vegetables (broccoli, Brussels sprouts)
- Leafy greens (folate, magnesium)
- Berries (antioxidants, low glycemic)
- Citrus fruits (vitamin C, flavonoids)

**Foods to Restrict or Avoid**

| **Category** | **Specific Items** | **Reason** |
| --- | --- | --- |
| **Alcohol** | All forms — **absolute contraindication** | Direct hepatotoxin; accelerates cirrhosis progression |
| **Simple sugars & SSBs** | Soda, juice, candy, pastries | Worsens MASLD, insulin resistance, hepatic steatosis |
| **Red and processed meats** | Beef, pork, sausage, deli meats | High ammonia generation → ↑ HE risk |
| **Saturated & trans fats** | Fried food, fast food, margarine | Promotes hepatic inflammation and steatosis |
| **High-sodium foods** | Canned soups, soy sauce, pickled foods | Risk of sodium retention and ascites |
| **Raw shellfish** | Oysters, clams, raw seafood | Risk of *Vibrio vulnificus* infection — potentially fatal in cirrhosis |
| **High-fructose foods** | Corn syrup products, agave | Directly drives hepatic lipogenesis in MASLD |

**5. Additional Nutritional Considerations**

**A. Caloric Target**

- **Total energy: 30–35 kcal/kg/day** = approximately **1,740 – 2,030 kcal/day**
- Given mild weight loss, target the **upper end (35 kcal/kg)** to achieve weight stabilization and lean mass recovery
- Goal: weight *stability* (not loss, despite MASLD background) — aggressive caloric restriction is harmful in cirrhosis

**B. Micronutrient Supplementation**

Patients with cirrhosis are prone to multiple deficiencies:

| **Nutrient** | **Concern** | **Recommendation** |
| --- | --- | --- |
| **Zinc** | Commonly deficient; zinc deficiency contributes to HE | Supplement 25–45 mg/day elemental zinc; increase dietary sources (pumpkin seeds, legumes) |
| **Vitamin D** | Near-universal deficiency in cirrhosis; bone loss risk | Check 25-OH Vitamin D; supplement 1,000–2,000 IU/day or per level |
| **B vitamins (B1, B6, B12, folate)** | Impaired hepatic metabolism | Consider B-complex supplementation; prioritize dietary sources |
| **Magnesium** | Often low; affects neuromuscular function | Leafy greens, nuts, seeds; supplement if deficient |
| **Iron** | Check ferritin/TIBC — avoid supplementing without deficiency | Excess iron is pro-oxidant in MASLD |
| **Fat-soluble vitamins (A, E, K)** | Impaired if cholestasis present | Assess and supplement cautiously; Vitamin E has emerging MASLD evidence |

**C. Hepatic Encephalopathy Prevention Through Diet**

- Maintain **bowel regularity** — constipation increases ammonia production; ensure 25–30 g dietary fiber/day
- **Lactulose** (if prescribed) should be taken consistently; nutritional fiber supports its effect
- Identify and address **HE precipitants**: avoid dehydration, high-protein red meat loads, constipation, infection
- Consider **probiotics** — emerging evidence supports gut microbiome modulation to reduce ammonia production

**D. Physical Activity**

- Gentle **resistance exercise** 2–3×/week strongly recommended
- Resistance training is synergistic with protein intake for sarcopenia reversal
- Aerobic activity (walking 30 min/day) improves insulin sensitivity — particularly relevant for MASLD

**E. Monitoring and Follow-Up**

- **Monthly weight monitoring** — any further unintentional weight loss requires urgent reassessment
- Consider **mid-arm muscle circumference** or **handgrip strength** to track sarcopenia
- Reassess dietary intake with a **3-day food diary** at follow-up
- **Dietitian follow-up every 3 months** recommended

**One-Day Sample Meal Plan**

| **Time** | **Meal** | **Approx. Protein** |
| --- | --- | --- |
| 7:00 AM | Oatmeal with milk + 2 scrambled eggs + ½ cup berries | ~22 g |
| 10:00 AM | Greek yogurt (150g) + small handful of walnuts | ~12 g |
| 12:30 PM | Lentil and vegetable soup + 90g grilled salmon + brown rice | ~30 g |
| 3:30 PM | Hummus with whole grain crackers + apple | ~8 g |
| 6:30 PM | Stir-fried tofu and broccoli with olive oil + quinoa | ~22 g |
| **9:30 PM** | **Cottage cheese (½ cup) + whole grain crackers** | **~12 g** |
| **TOTAL** |  | **~106 g protein / ~1,850 kcal** |

## C307

**Dietary & Nutritional Recommendations**

**Patient:** Male, 57 y/o | 168 cm / 62 kg | BMI: 22.0 kg/m² **Diagnosis:** HBV Cirrhosis, history of Hepatic Encephalopathy (HE)

**1. Overall Dietary Principles**

This patient's nutritional strategy must balance **adequate caloric and protein support** to prevent sarcopenia and malnutrition — both highly prevalent and prognostically important in cirrhosis — while **mitigating the risk of recurrent hepatic encephalopathy**.

- **Target total daily energy intake:** 35–40 kcal/kg/day → approximately **2,170–2,480 kcal/day**
- Avoid prolonged fasting (>4–6 hours), which accelerates muscle catabolism in cirrhotic patients
- Maintain a **sodium restriction** of **<2 g/day** (even without overt ascites at this visit, prophylaxis is appropriate in decompensated cirrhosis)
- Avoid all alcohol absolutely

**2. Recommended Daily Protein Intake**

**Critical point:** Despite the history of HE, protein restriction is **no longer recommended**. Evidence consistently shows it worsens sarcopenia and outcomes. The focus is on **protein source and distribution**, not restriction.

| **Parameter** | **Recommendation** |
| --- | --- |
| **Total daily protein** | **1.2–1.5 g/kg/day** → ~74–93 g/day |
| **Preferred protein source** | Vegetable protein & dairy (casein/whey) |
| **Branched-Chain Amino Acids (BCAAs)** | Consider supplementation (e.g., 12–15 g/day) |
| **Animal protein (red meat)** | Minimize — high in aromatic amino acids and ammoniagenic |

**Rationale for BCAA supplementation:** This patient has a history of HE, placing him at risk for a low BCAA:aromatic amino acid ratio. BCAAs (leucine, isoleucine, valine) improve nitrogen balance without increasing ammonia load and may reduce HE recurrence risk.

**3. Meal Frequency and Eating Pattern**

The **"multiple small meals + late-evening snack (LES)"** pattern is the cornerstone recommendation for cirrhosis, supported by EASL and ESPEN guidelines.

Breakfast	~7:00 AM	── ~500–600 kcal

Mid-Morning	~10:00 AM ── ~200–250 kcal

Lunch	~12:30 PM ── ~500–600 kcal

Afternoon	~3:30 PM	── ~200–250 kcal

Dinner	~6:30 PM	── ~500–600 kcal

Late-Evening	~9:30 PM	── ~200–250 kcal ← most important

**Late-Evening Snack (LES) — High Priority**

- Consume **within 2–3 hours of sleep**
- Ideal composition: **complex carbohydrate + protein**
- Examples: whole grain crackers with low-fat cheese, a small bowl of oatmeal with milk, or a BCAA-enriched supplement drink
- **Purpose:** Shortens the overnight fasting interval, reduces gluconeogenesis from muscle, and has been shown to improve nitrogen balance in cirrhotic patients

**4. Specific Food Recommendations and Restrictions**

**Recommended Foods**

| **Category** | **Examples** |
| --- | --- |
| **Vegetable proteins** | Tofu, edamame, lentils, chickpeas, tempeh |
| **Dairy proteins** | Low-fat yogurt, low-fat milk, cottage cheese, whey protein |
| **Complex carbohydrates** | Brown rice, oats, whole wheat bread, sweet potato |
| **Healthy fats** | Olive oil, avocado (moderate), walnuts |
| **Fruits & vegetables** | All well-tolerated options; emphasize leafy greens, berries |
| **Zinc-rich foods** | Pumpkin seeds, legumes, dairy (zinc deficiency is common in cirrhosis) |

**Foods to Restrict or Avoid**

| **Item** | **Reason** |
| --- | --- |
| **Red and processed meats** | High in ammoniagenic amino acids → HE risk |
| **High-sodium foods** (processed foods, pickles, canned soups, soy sauce) | Promotes fluid retention, ascites |
| **Alcohol** | Absolutely contraindicated |
| **Raw shellfish and raw fish** | Risk of *Vibrio vulnificus* infection — potentially fatal in cirrhosis |
| **High-fat fried foods** | Worsens hepatic steatosis, poor tolerance |
| **High-fructose corn syrup / sugary drinks** | Promotes hepatic stress and metabolic complications |
| **Herbal supplements / TCM remedies** | Many are hepatotoxic; must be cleared by physician |

**5. Additional Nutritional Considerations**

**Micronutrient Supplementation**

Cirrhosis frequently causes fat-soluble vitamin and micronutrient deficiencies:

| **Nutrient** | **Consideration** |
| --- | --- |
| **Vitamin D** | Check 25-OH vitamin D; supplement if <30 ng/mL (deficiency very common in HBV cirrhosis) |
| **Zinc** | Often depleted; consider 25–45 mg elemental zinc/day — zinc also helps reduce ammonia production |
| **Vitamin B1 (Thiamine)** | Supplement routinely, especially if any prior alcohol use |
| **Vitamin K** | Monitor if INR is elevated; cautious supplementation may be appropriate |
| **Magnesium** | Check levels, especially if diuretics are in use |
| **Iron** | Do NOT supplement empirically — iron overload can worsen HBV-related liver fibrosis |

**Hepatic Encephalopathy Prevention**

- **Lactulose** compliance (if prescribed) should be reinforced at dietary counseling — target 2–3 soft stools/day
- Maintain **regular bowel habits** through dietary fiber intake (~25–30 g/day) to reduce intestinal ammonia absorption
- Stay well-hydrated (~1.5–2 L water/day unless fluid-restricted due to hyponatremia)
- Avoid **constipation** — a known precipitant of HE episodes

**Sarcopenia Monitoring**

- This patient's BMI of 22.0 appears normal, but **cirrhotic patients can have significant muscle wasting masked by fluid retention** — consider mid-arm muscle circumference, handgrip strength, or CT-based psoas muscle index for more accurate assessment
- Encourage **light to moderate physical activity** (e.g., walking 30 min/day) as tolerated — exercise improves muscle mass, insulin sensitivity, and may reduce portal hypertension

**Follow-Up**

- Reassess nutritional status every **1–3 months**
- Monitor weight trends carefully (sudden gain >2 kg in 1 week may indicate fluid accumulation, not true weight gain)
- Coordinate with hepatologist regarding **rifaximin** use for secondary HE prophylaxis, which complements dietary management

## C308

**Dietary & Nutritional Recommendations**

**Patient C3-08 | Female, 60 y/o | Alcohol Cirrhosis + HE History**

**BMI: 31.2 kg/m²** (Obese Class I) | **IBW: ~52 kg** | **Note: Obesity may mask underlying sarcopenia — assess muscle mass carefully**

**1. Overall Dietary Principles**

- **Primary goals:** Prevent/manage hepatic encephalopathy (HE), preserve muscle mass, avoid further hepatic insult, and address metabolic complications of obesity with cirrhosis
- **Absolute alcohol abstinence** — non-negotiable; even minimal alcohol accelerates fibrosis and HE risk
- **Avoid prolonged fasting** — the cirrhotic liver has severely limited glycogen stores; fasting >4–6 hours can precipitate catabolism and worsen HE
- **Energy target:** ~25–35 kcal/kg of **dry body weight** per day. Given obesity, use adjusted body weight to avoid overfeeding. Estimated target: **~1,600–1,900 kcal/day** (reassess with indirect calorimetry if available)
- Maintain a **consistent, moderate-calorie diet** — do not pursue aggressive caloric restriction despite obesity, as this risks muscle wasting, which is the true driver of poor prognosis in cirrhosis

**2. Recommended Daily Protein Intake**

**Critical principle:** Protein restriction is *NOT* recommended in cirrhosis with HE history. It worsens sarcopenia and outcomes. The old paradigm of low-protein diets is obsolete.

| **Recommendation** | **Amount** |
| --- | --- |
| **Target protein intake** | **1.2 – 1.5 g/kg adjusted body weight/day** |
| **Estimated daily target** | **~85 – 105 g protein/day** |
| **In active/recent HE** | Start at 1.0–1.2 g/kg; titrate up as tolerated |

- **Prioritize plant-based and dairy proteins** over red meat. Plant proteins (legumes, soy, tofu) produce less ammonia during gut metabolism and are better tolerated in HE-prone patients
- **Branched-chain amino acids (BCAAs):** Consider BCAA supplementation (leucine, isoleucine, valine — 12–18 g/day) if dietary protein targets cannot be met or if HE recurs despite adequate intake. Supported by EASL and AASLD guidelines for sarcopenic cirrhotics
- **Avoid high-methionine, high-aromatic amino acid sources** (e.g., large portions of red meat, processed meats) as these are more ammoniagenic

**3. Meal Frequency and Eating Pattern**

This is one of the **most impactful interventions** in cirrhosis nutrition:

- **4–6 small meals per day** rather than 2–3 large meals — reduces post-meal hyperammonemia and prevents hypoglycemia
- **Mandatory late-evening snack (LES):** Consume a carbohydrate- and protein-containing snack **within 1–2 hours of bedtime**
  - Example: 1 cup low-fat yogurt + small banana, or whole grain crackers + peanut butter
  - Evidence-based: LES reduces overnight fasting duration, improves nitrogen balance, and reduces muscle catabolism
- **Do not skip breakfast** — begin eating within 1 hour of waking
- Suggested schedule:

| **Time** | **Meal** |
| --- | --- |
| 7:00–8:00 AM | Breakfast |
| 10:30–11:00 AM | Mid-morning snack |
| 12:30–1:00 PM | Lunch |
| 3:30–4:00 PM | Afternoon snack |
| 6:30–7:00 PM | Dinner |
| 9:30–10:00 PM | **Late-evening snack** ✱ |

**4. Specific Food Recommendations and Restrictions**

**Encouraged Foods**

| **Category** | **Examples** |
| --- | --- |
| **Plant proteins** | Tofu, tempeh, edamame, lentils, chickpeas, black beans |
| **Dairy proteins** | Low-fat Greek yogurt, low-fat cottage cheese, low-fat milk |
| **Complex carbohydrates** | Oatmeal, brown rice, quinoa, whole grain bread, sweet potato |
| **Healthy fats** | Olive oil, avocado, walnuts, flaxseed (moderate portions) |
| **Vegetables** | All non-starchy vegetables; cooked preferred if ascites present |
| **Fruits** | Berries, apples, pears (monitor portion size given obesity) |
| **Lean fish/poultry** | Salmon, sardines (omega-3 benefit), skinless chicken/turkey — in moderate portions |

**Foods to Restrict or Avoid**

| **Category** | **Reason** |
| --- | --- |
| **All alcohol** | Direct hepatotoxin; precipitates HE and accelerates cirrhosis |
| **Red and processed meats** | High ammoniagenic amino acid content; increased HE risk |
| **High-sodium foods** (processed, canned, fast food) | Risk of/exacerbating ascites and fluid retention; target **<2,000 mg Na/day** (especially if ascites present) |
| **Raw shellfish (oysters, clams)** | Risk of *Vibrio vulnificus* — fatal in cirrhosis |
| **Raw/undercooked eggs, unpasteurized products** | Infection risk (immunocompromised state) |
| **High-fructose corn syrup, sugary beverages** | Worsens metabolic profile; hepatic lipotoxicity |
| **Excessive dietary fat** | May worsen hepatic steatosis; limit saturated fat |
| **Herbal supplements, kava, high-dose Vitamin A** | Hepatotoxic potential |

**5. Additional Nutritional Considerations**

**Micronutrient Deficiencies (Common in Alcohol Cirrhosis)**

| **Nutrient** | **Clinical Note** |
| --- | --- |
| **Thiamine (B1)** | Critical — supplement 100 mg/day routinely; prevent Wernicke's encephalopathy |
| **Folate (B9)** | Often depleted; supplement 1 mg/day |
| **Vitamin B6 (Pyridoxine)** | Supplement if deficient |
| **Zinc** | Zinc deficiency impairs urea cycle → worsens HE; supplement 220 mg zinc sulfate BID if serum zinc low; zinc also improves taste perception encouraging adequate intake |
| **Magnesium** | Frequently low in alcohol-related disease; replace as needed |
| **Vitamin D** | Check 25-OH Vitamin D; most cirrhotics are deficient; supplement 1,000–2,000 IU/day |
| **Fat-soluble vitamins (A, E, K)** | May be deficient due to cholestasis/fat malabsorption; avoid megadosing Vitamin A (hepatotoxic) |

**Hepatic Encephalopathy — Dietary Management**

- **Lactulose compliance:** Ensure patient understands dietary protein is *not* the enemy — lactulose/rifaximin (if prescribed) are the primary HE tools; dietary protein should not be sacrificed
- **Fiber intake:** 25–30 g/day — soluble fiber feeds beneficial gut bacteria, reduces ammonia production, and promotes bowel regularity (important for ammonia clearance)
- **Hydration:** 1.5–2 L water/day (adjust if ascites/fluid restriction is in place)
- **Avoid constipation** — a precipitant of HE; high fiber, adequate fluids, and lactulose dosing should target 2–3 soft stools per day

**Weight Management Nuance**

- Despite BMI of 31.2, **aggressive weight loss dieting is contraindicated** — risk of accelerating sarcopenia
- A **modest caloric deficit of 200–300 kcal/day** combined with **resistance exercise** (if tolerated and not contraindicated by varices/ascites) is the safest approach
- If ascites is present, note that true body weight is likely lower once fluid is accounted for; reassess dry weight

**Monitoring & Follow-Up Recommendations**

- Reassess nutritional status every **4–8 weeks** with handgrip strength, mid-arm muscle circumference, or BIA (if no ascites)
- Monitor serum albumin, prealbumin, zinc, magnesium, phosphate, LFTs regularly
- Refer to a **registered dietitian (RD) specializing in hepatology** for ongoing individualized meal planning
- Assess for **alcohol use disorder treatment** (concurrent addiction medicine/psychiatry referral) — nutritional optimization is undermined without sobriety support

## C309

**Dietary & Nutritional Recommendations**

**Patient: Male, 64 y/o | 167 cm | 57 kg | BMI 20.4 | MASLD Cirrhosis + Recovered HE**

**Preliminary Assessment**

| **Parameter** | **Value** | **Interpretation** |
| --- | --- | --- |
| BMI | 20.4 kg/m² | Low-normal; clinically significant in cirrhosis |
| Ideal Body Weight | ~63–67 kg | Current weight is ~85–90% IBW |
| Nutritional Status | **At high risk for sarcopenic malnutrition** | Requires aggressive nutritional support |

**Critical Note:** Despite the history of hepatic encephalopathy (HE), **protein restriction is contraindicated** and outdated. Adequate protein intake is essential to prevent sarcopenia, which itself worsens HE prognosis.

**1. Overall Dietary Principles**

- **Priority goal:** Reverse malnutrition and prevent/treat sarcopenia while supporting hepatic regeneration
- **Caloric target:** **35–40 kcal/kg actual body weight/day** = approximately **2,000–2,300 kcal/day**
- Maintain a **high-calorie, high-protein, low-simple-sugar** dietary pattern consistent with the MASLD background
- Avoid prolonged fasting (>4–6 hours) — fasting accelerates protein catabolism in cirrhotic patients
- Favor **small, frequent meals** over large portions to reduce metabolic stress on the liver
- Ensure **adequate micronutrient and branched-chain amino acid (BCAA) intake**

**2. Recommended Daily Protein Intake**

| **Recommendation** | **Details** |
| --- | --- |
| **Target** | **1.2 – 1.5 g protein/kg body weight/day** |
| **Daily amount** | ≈ **68 – 86 g protein/day** (based on 57 kg) |
| **Preferred sources** | Plant proteins (legumes, soy) + dairy + eggs + lean fish/poultry |
| **BCAA supplementation** | **Strongly recommended** if oral intake is insufficient or HE recurs |

- **Do NOT restrict protein** due to HE history — current EASL/AASLD guidelines oppose protein restriction in HE
- If ammonia remains a concern, **prioritize vegetable-source and dairy proteins** over red meat, as they generate less ammonia and are better tolerated in cirrhosis
- BCAA-enriched oral supplements (leucine, isoleucine, valine) improve nitrogen balance and reduce HE risk

**3. Meal Frequency and Eating Pattern**

Recommended Schedule (6-meal pattern):

─────────────────────────────────────────────────────

07:00	Breakfast	~500–550 kcal

10:00	Morning Snack	~200–250 kcal

12:30	Lunch	~550–600 kcal

15:30	Afternoon Snack	~200–250 kcal

18:30	Dinner	~450–500 kcal

21:00	Late-Evening Snack	~200–250 kcal ⭐

─────────────────────────────────────────────────────

TOTAL: ~2,100–2,400 kcal

⭐ **Late-Evening Snack (LES) is mandatory** — This is a cornerstone recommendation in cirrhosis nutrition. A carbohydrate + protein snack (e.g., 1 cup oatmeal + milk, or yogurt + banana) before bed reduces overnight fasting-induced catabolism and has been shown in RCTs to improve nitrogen balance and muscle mass.

**4. Specific Food Recommendations and Restrictions**

**Recommended Foods**

| **Category** | **Examples** | **Rationale** |
| --- | --- | --- |
| **Plant proteins** | Lentils, chickpeas, tofu, edamame, soy milk | Lower ammonia load; BCAA-rich |
| **Dairy & eggs** | Low-fat yogurt, cottage cheese, eggs | High biological value protein; well tolerated |
| **Lean fish** | Salmon, cod, tilapia (2–3×/week) | Omega-3s; anti-inflammatory; high protein |
| **Complex carbohydrates** | Oats, brown rice, sweet potato, whole grain bread | Sustained energy; avoid glucose spikes |
| **Healthy fats** | Olive oil, avocado, nuts/seeds (moderate) | Anti-inflammatory; caloric density for weight gain |
| **Vegetables** | All cooked vegetables; leafy greens | Fiber; micronutrients; modulate gut flora → ↓ ammonia |
| **Fruits** | Bananas, berries, apples (fresh or stewed) | Energy; potassium; antioxidants |

**Foods to Restrict or Avoid**

| **Category** | **Reasoning** |
| --- | --- |
| **Alcohol** (absolute contraindication) | Direct hepatotoxin; worsens cirrhosis regardless of MASLD etiology |
| **Red and processed meats** | High ammonia-generating amino acids; pro-inflammatory |
| **Added sugars & ultra-processed foods** | Worsens MASLD metabolic dysfunction; promotes hepatic fat |
| **High-fructose corn syrup / sugary drinks** | Directly promotes hepatic steatosis and fibrosis progression |
| **Raw shellfish** (oysters, clams) | Risk of *Vibrio vulnificus* — potentially fatal in cirrhosis |
| **High-sodium foods** | If ascites or edema present — target **< 2 g Na/day** |
| **Excess saturated fats** | Promotes inflammation and insulin resistance in MASLD |

**5. Additional Nutritional Considerations**

**Micronutrient Supplementation**

| **Nutrient** | **Recommendation** | **Rationale** |
| --- | --- | --- |
| **Zinc** | 25–50 mg/day elemental zinc | Commonly deficient in cirrhosis; zinc depletion worsens HE by impairing urea cycle |
| **Vitamin D** | 1,000–2,000 IU/day (check serum 25-OH-D) | Near-universal deficiency in MASLD/cirrhosis; critical for muscle function and immunity |
| **Magnesium** | Replete if low (dietary or supplemental) | Often depleted; important for neuromuscular function |
| **B Vitamins (B1, B6, B12, Folate)** | Via diet or multivitamin | Impaired hepatic storage and activation in cirrhosis |
| **Vitamin K** | Dietary (leafy greens) or supplement with caution | Coagulopathy support; monitor if on anticoagulants |
| **Iron** | **Avoid supplementation unless confirmed deficient** | Iron overload worsens hepatic oxidative stress in MASLD |

**BCAA Oral Supplementation**

- **Strongly consider** in this patient given low BMI and HE history
- Available as powdered supplements (e.g., Livact® granules)
- Target: **12–15 g BCAA/day** divided across meals
- Particularly beneficial for improving albumin synthesis and reducing HE recurrence

**Physical Activity (Adjunct to Nutrition)**

- Encourage **light-to-moderate resistance exercise** (e.g., resistance bands, bodyweight exercises) 3×/week
- Combined with nutrition therapy, exercise is the most effective intervention for **sarcopenia reversal** in cirrhosis
- Avoid high-intensity exercise if esophageal varices are known and uncontrolled

**Hepatic Encephalopathy Prevention (Dietary)**

- Ensure **adequate dietary fiber** (25–30 g/day) to promote bowel regularity and reduce colonic ammonia absorption
- **Lactulose compliance** (if prescribed) complements dietary management
- Consider **probiotic-containing foods** (yogurt, kefir) — emerging evidence supports gut microbiome modulation to reduce ammonia production
- Monitor for **constipation** — a common precipitant of HE

**Monitoring & Follow-Up**

- Reassess weight, muscle mass (hand-grip strength, calf circumference) **every 4–6 weeks**
- Target **weight gain of 0.5–1 kg/month** given current low BMI
- Consider referral to a **registered dietitian with hepatology expertise** for ongoing monitoring
- Reassess ammonia and albumin levels alongside dietary adherence at each hepatology visit

**Summary Priority List for This Patient:**

1. ↑ Total caloric intake to 2,000–2,300 kcal/day
2. ↑ Protein to 1.2–1.5 g/kg/day via plant/dairy sources
3. Implement mandatory **late-evening snack**
4. Supplement **zinc + Vitamin D + BCAA**
5. Absolute alcohol avoidance
6. Light resistance exercise program

## C310

**Dietary & Nutritional Recommendations**

**Patient: Female, 68 y/o | HBV Cirrhosis | Prior HE | Poor Appetite | BMI 19.8 kg/m²**

**Preliminary Assessment**

| **Parameter** | **Value** | **Interpretation** |
| --- | --- | --- |
| Height / Weight | 159 cm / 50 kg | — |
| BMI | **19.8 kg/m²** | Low-normal; at risk for sarcopenia |
| Key concerns | Prior HE, poor appetite, advanced age, HBV etiology | High nutritional risk |

**This patient is at significant risk for protein-calorie malnutrition and sarcopenia**, both of which worsen cirrhosis prognosis and increase HE recurrence risk independently.

**1. Overall Dietary Principles**

- **Prioritize adequate nutrition over restriction.** The outdated practice of protein restriction to prevent HE is **contraindicated** per current EASL/AASLD guidelines — malnutrition is far more dangerous in this patient than dietary protein load.
- Maintain a **high-calorie, high-protein, low-simple-sugar** dietary pattern tailored to hepatic metabolic capacity.
- Aim for **25–35 kcal/kg/day** of actual body weight → approximately **1,250–1,750 kcal/day**.
- Emphasize **small, frequent, palatable meals** to overcome poor appetite.
- Avoid prolonged fasting (>4–6 hours), which accelerates gluconeogenesis from muscle in cirrhosis.
- Sodium restriction (**<2,000 mg/day**) if ascites is present or anticipated.

**2. Recommended Daily Protein Intake**

**Target: 1.2–1.5 g/kg/day → approximately 60–75 g protein/day**

| **Condition** | **Rationale** |
| --- | --- |
| Cirrhosis (general) | 1.2–1.5 g/kg/day (EASL 2019) |
| Prior hepatic encephalopathy | **Do NOT restrict** — maintain ≥1.2 g/kg/day |
| Sarcopenia risk (elderly + low BMI) | Favor upper range ~1.5 g/kg/day |

**Protein Source Strategy (Critical for HE History):**

- **Prefer branched-chain amino acid (BCAA)-rich proteins**: dairy (especially whey), eggs, legumes, fish
- **Vegetable and dairy protein** is preferred over red meat — lower ammoniagenic amino acid content, higher fiber (promotes colonic ammonia excretion)
- **BCAA oral supplementation** (e.g., 12–15 g BCAA/day) should be considered given poor appetite and HE history — strong evidence for reducing HE recurrence and improving nutritional status
- Limit (do not eliminate) red meat and processed meat — higher aromatic amino acid load
- Avoid large single-bolus protein loads — distribute evenly across all meals/snacks

**3. Meal Frequency and Eating Pattern**

Given **poor appetite** and the **metabolic instability of cirrhosis**, meal pattern restructuring is essential:

Recommended Daily Schedule

━━━━━━━━━━━━━━━━━━━━━━━━━━━━━━━━━━━━━━━━━━

7:00 AM	Breakfast (main meal — capitalize on morning appetite)

10:00 AM	Mid-morning snack

12:30 PM	Lunch

3:30 PM	Afternoon snack

6:30 PM	Dinner (lighter)

9:00 PM	★ Late-evening snack (LES) ← MANDATORY

━━━━━━━━━━━━━━━━━━━━━━━━━━━━━━━━━━━━━━━━━━

**★ Late-Evening Snack (LES) — Highest Priority Intervention**

- **Time:** Within 2 hours of bedtime
- **Composition:** ~200 kcal + 15–20 g complex carbohydrate + protein (e.g., BCAA supplement or dairy)
- **Rationale:** Cirrhotic patients enter a fasting-equivalent catabolic state after only 2–3 hours without food. LES has the highest level of evidence (Level A) for reducing overnight muscle catabolism and improving nitrogen balance.
- **Examples:** Whole grain crackers + cheese, warm oatmeal with milk, or a commercial BCAA-enriched snack drink

**4. Specific Food Recommendations and Restrictions**

**Recommended Foods**

| **Category** | **Examples** | **Benefit** |
| --- | --- | --- |
| **Dairy** | Low-fat yogurt, milk, cottage cheese | High BCAA, well-tolerated protein |
| **Eggs** | Soft-boiled, scrambled | Complete protein, easy to prepare |
| **Fish / Seafood** | Salmon, tilapia, cod (well-cooked) | Lean protein + omega-3 anti-inflammatory |
| **Legumes** | Tofu, lentils, edamame | Vegetable protein, fiber aids ammonia clearance |
| **Complex carbohydrates** | Oats, brown rice, sweet potato | Sustained energy, prevents hypoglycemia |
| **Vegetables** | Cooked leafy greens, carrots, squash | Fiber + micronutrients; cook to improve palatability |
| **Healthy fats** | Olive oil, avocado (small amounts) | Calorie-dense; useful for poor appetite |
| **Soft/palatable foods** | Congee, steamed dishes, soups | Accommodates poor appetite and early satiety |

**Foods to Limit**

| **Food** | **Reason** |
| --- | --- |
| Red meat, organ meats | High ammoniagenic aromatic amino acids |
| High-sodium foods (pickles, soy sauce, processed foods) | Risk of fluid retention / ascites |
| Refined sugars, sugary drinks | Risk of hepatic steatosis, glucose instability |
| Raw shellfish / raw fish | High *Vibrio vulnificus* risk in cirrhosis — potentially fatal |
| Large portions in single sitting | Worsens early satiety, increases ammonia spike |

**Absolute Restrictions**

| **Item** | **Reason** |
| --- | --- |
| **Alcohol** | Accelerates hepatic fibrosis progression regardless of etiology |
| **Raw/undercooked shellfish or meat** | Life-threatening infection risk in cirrhosis |
| **Herbal supplements / TCM hepatotoxic herbs** | Many are hepatotoxic (e.g., pyrrolizidine alkaloids) |
| **High-dose iron supplements** (unless deficient) | Iron overload worsens HBV cirrhosis |

**5. Additional Nutritional Considerations**

**Micronutrient Supplementation**

| **Nutrient** | **Recommendation** | **Rationale** |
| --- | --- | --- |
| **Zinc** | 50 mg elemental zinc/day (e.g., zinc acetate) | Commonly deficient in cirrhosis; essential cofactor for urea cycle enzymes — directly reduces ammonia |
| **Vitamin D** | 1,000–2,000 IU/day (check 25-OH-D level first) | Near-universal deficiency in cirrhosis; important for muscle and bone in elderly female |
| **B vitamins (B1, B6, B12, folate)** | Standard supplementation | Depleted in liver disease; B1 (thiamine) especially important |
| **Vitamin K** | As needed (if INR elevated) | Impaired hepatic synthesis |
| **Magnesium** | Monitor and supplement if deficient | Common in cirrhosis, affects neuromuscular function |

**BCAA Supplementation:** Strongly recommended given HE history + poor oral intake. Target **12–15 g/day** of oral BCAA (leucine, isoleucine, valine). Can be taken as powder mixed into food/drinks to improve palatability.

**Sarcopenia Prevention**

- This 68-year-old female with low-normal BMI is at **high risk for sarcopenic obesity** (normal weight but muscle-depleted).
- Encourage **gentle resistance exercise** (chair exercises, resistance bands) as tolerated — exercise + nutrition synergistically preserve muscle mass in cirrhosis.
- Consider **handgrip strength** and **calf circumference** monitoring at follow-up visits.

**Hepatic Encephalopathy Prevention Through Diet**

- Maintain **bowel regularity** — constipation increases ammonia absorption. High-fiber diet + adequate hydration (1,500 mL/day unless fluid-restricted).
- **Lactulose** (if prescribed) should be coordinated with dietary fiber intake.
- Recognize early HE symptoms (sleep reversal, mild confusion) as triggers to reassess dietary ammonia load.

**Monitoring & Follow-Up**

- Reassess nutritional status every **4–8 weeks** initially
- Track: body weight, mid-arm muscle circumference, serum albumin, prealbumin, zinc levels
- Referral to a **registered dietitian** for ongoing individualized meal planning is strongly advised

**Summary Priority List for This Patient:**

1. ☑ Ensure ≥60–75 g protein/day — do NOT restrict
2. ☑ Implement late-evening snack every night
3. ☑ Start BCAA supplementation + zinc supplementation
4. ☑ 4–6 small meals daily; never fast >4 hours
5. ☑ Absolutely no alcohol, no raw shellfish

## C311

**Clinical Nutrition Recommendations**

**Patient: Male, 72 y/o | Alcohol Cirrhosis with Recurrent Hepatic Encephalopathy**

**BMI: 25.6 kg/m²** (Height 170 cm, Weight 74 kg)

**Key Clinical Context Before Recommendations**

Despite a seemingly normal BMI, this patient is at **high risk for sarcopenic obesity** — excess fat mass masking significant muscle loss, which is extremely common in cirrhosis and is an independent driver of hepatic encephalopathy (HE). Nutritional assessment must go beyond weight alone.

**1. Overall Dietary Principles**

| **Principle** | **Rationale** |
| --- | --- |
| **Absolute alcohol abstinence** | Continued alcohol use accelerates hepatocellular damage and directly precipitates HE |
| **Prevent and treat malnutrition/sarcopenia** | Muscle wasting reduces ammonia detoxification capacity, worsening HE |
| **Avoid prolonged fasting** | Fasting triggers gluconeogenesis from muscle protein, generating ammonia and worsening encephalopathy |
| **Energy balance** | Target **30–35 kcal/kg/day** (~2,220–2,590 kcal/day); avoid both under- and overfeeding |
| **Soft, easily digestible foods** | Reduces gut transit time and bacterial ammonia production |

**2. Recommended Daily Protein Intake**

**CRITICAL POINT:** Protein restriction is **no longer recommended** in HE. It worsens sarcopenia and paradoxically increases ammonia production from muscle catabolism.

**Target: 1.2 – 1.5 g/kg/day → ~89–111 g protein/day**

*(Per EASL, AASLD, and ESPEN guidelines for cirrhosis with HE)*

**Protein Source Strategy:**

**Prioritize — Vegetable & Dairy Proteins**

- **Branched-chain amino acids (BCAAs)** — found in legumes, soy, dairy
- Vegetable proteins produce less ammonia than animal proteins (lower aromatic amino acid content, higher fiber content speeds colonic transit)
- **Dairy protein** (whey, casein) is well-tolerated and supports muscle synthesis

**Acceptable — White Meat & Fish**

- Chicken breast, turkey, freshwater fish, salmon (2–3×/week)
- Lower in aromatic amino acids compared to red meat

**Use with Caution — Red & Processed Meat**

- Beef, pork, lamb, sausage, deli meats
- High in aromatic amino acids and methionine → increased ammonia load
- Limit to ≤2 servings/week, if used at all

**Consider BCAA Oral Supplementation (if dietary intake is insufficient)**

- 12–15 g/day of BCAA supplement (leucine, isoleucine, valine) has evidence for improving HE and muscle mass in cirrhosis

**3. Meal Frequency and Eating Pattern**

This is **one of the most important interventions** for HE management.

**Recommended: 4–6 small meals per day + mandatory late-evening snack (LES)**

07:00 AM ──── Breakfast (complex carbs + protein)

10:00 AM ──── Mid-morning snack (fruit + yogurt)

01:00 PM ──── Lunch (main meal — protein + vegetables + grains)

04:00 PM ──── Afternoon snack (nuts + whole grain crackers)

07:00 PM ──── Dinner (lighter — protein + vegetables)

10:00 PM ──── ★ Late Evening Snack (LES) ← Most Critical

**★ Late Evening Snack (LES) — Non-Negotiable**

- **What:** 50 g complex carbohydrates + protein (e.g., whole grain bread with peanut butter, or a BCAA drink)
- **Why:** Reduces overnight fasting duration from ~12 hours to ~4 hours, preventing catabolic gluconeogenesis from muscle. Studies show LES significantly reduces HE episodes and improves nitrogen balance
- Overnight fasting in cirrhotics metabolically mimics a **3-day fast** in healthy individuals

**4. Specific Food Recommendations and Restrictions**

**RECOMMENDED FOODS**

**Protein Sources:**

- Tofu, tempeh, edamame, lentils, chickpeas, black beans
- Low-fat dairy: Greek yogurt, cottage cheese, milk
- Eggs (1–2/day, prefer egg whites if HE is active)
- Fish: salmon, tilapia, cod (2–3×/week)
- Chicken/turkey breast (skinless)

**Carbohydrates (complex, low glycemic index):**

- Oatmeal, brown rice, quinoa, whole grain bread/pasta
- Sweet potato, yam (high potassium — monitor if on diuretics)

**Fruits & Vegetables:**

- Bananas, apples, berries, papaya
- Spinach, kale, broccoli, carrots, zucchini
- High fiber content → promotes gut motility → reduces colonic ammonia absorption

**Healthy Fats:**

- Olive oil (primary cooking fat), avocado, walnuts
- Omega-3 rich foods: flaxseed, chia seeds, fatty fish

**FOODS TO RESTRICT OR AVOID**

| **Category** | **Restriction** | **Reason** |
| --- | --- | --- |
| **Alcohol** | **Absolute prohibition** | Direct hepatotoxin, precipitates HE |
| **Red/processed meats** | Minimize | High ammonia precursor load |
| **High-sodium foods** | **< 2,000 mg Na/day** | Manage/prevent ascites and edema |
| **Raw shellfish** | Avoid entirely | Risk of *Vibrio vulnificus* — fatal in cirrhosis |
| **Unpasteurized foods** | Avoid | Infection risk with impaired immunity |
| **High-sugar foods** | Limit | Risk of hepatic steatosis, blood glucose instability |
| **Excessive red meat** | ≤1–2×/week | Ammonia generation |
| **Large meals** | Avoid | Increases metabolic demand on liver |

**5. Additional Nutritional Considerations**

**5a. Micronutrient Supplementation (High Priority in Alcohol Cirrhosis)**

| **Nutrient** | **Recommendation** | **Rationale** |
| --- | --- | --- |
| **Thiamine (B1)** | 100 mg/day oral (or IV if Wernicke's suspected) | **Critical** — alcohol depletes thiamine; deficiency causes Wernicke's encephalopathy, which mimics/worsens HE |
| **Zinc** | 25–45 mg elemental zinc/day | Zinc deficiency is nearly universal in alcohol cirrhosis; zinc is a cofactor for urea cycle enzymes — supplementation reduces ammonia and improves HE |
| **Folate (B9)** | 1 mg/day | Commonly deficient in alcoholism |
| **Vitamin B6 & B12** | Daily supplementation | Depleted by alcohol |
| **Vitamin D** | 1,000–2,000 IU/day (monitor levels) | Deficiency in >90% of cirrhotics; impacts bone and immune health |
| **Magnesium** | Per serum levels | Alcohol-related renal wasting |
| **Vitamin K** | Per clinical need | Fat-soluble vitamin malabsorption in cirrhosis |

**Fat-soluble vitamins (A, D, E, K):** Use with caution — Vitamin A megadoses are hepatotoxic; supplement only with confirmed deficiency.

**5b. Sodium Management**

- Target **< 2,000 mg sodium/day** (< 5g salt)
- Avoid: canned soups, processed meats, soy sauce, pickled foods, fast food
- Use herbs, lemon juice, garlic, and spices for flavor

**5c. Fluid Management**

- If **ascites is present**: fluid restriction may be needed (**1,000–1,500 mL/day**)
- Monitor daily weights; report >2 kg gain in 1 week (suggests fluid accumulation)
- Coordinate fluid guidance with hepatologist/nephrology team

**5d. HE Precipitant Awareness (Dietary Triggers)**

Counsel patient to watch for and report:

- Constipation → increases colonic ammonia absorption → **ensure high fiber intake + adequate hydration**
- GI bleeding → blood in gut fermented to ammonia (report black stools immediately)
- High-protein binge eating → acute ammonia load

**5e. Sarcopenia Monitoring**

At age 72 with alcohol cirrhosis, this patient should have:

- **Hand grip strength** measured at each visit
- **6-minute walk test** or gait speed assessment
- Consider **DEXA scan** to assess lean body mass (true nutritional status may be masked by fluid retention)

**Summary Card for Patient**

**Eat 5–6 small meals daily — never skip the bedtime snack** **Focus protein on legumes, dairy, fish, and poultry** **Zero alcohol — ever** **Low salt diet — avoid processed and canned foods** **Take thiamine and zinc supplements as prescribed** **Eat plenty of fruits, vegetables, and whole grains** **Weigh yourself daily — report sudden weight gain**

## C312

**Dietary & Nutritional Recommendations**

**Patient:** Female, 75 y/o | 155 cm / 62 kg | BMI ≈ 25.8 kg/m² **Diagnosis:** MASLD Cirrhosis with prior Hepatic Encephalopathy (HE)

**1. Overall Dietary Principles**

This patient presents with a dual nutritional challenge: **metabolic risk** (MASLD etiology requires cardiometabolic attention) alongside **cirrhosis-related malnutrition risk** (sarcopenia is prevalent even at a seemingly normal BMI in elderly cirrhotics, as BMI masks muscle loss). The overarching goals are:

- **Prevent/treat sarcopenia** — a major driver of HE, decompensation, and mortality
- **Support hepatic regeneration** while reducing metabolic burden
- **Minimize HE triggers** without unnecessary protein restriction (outdated practice)
- **Maintain glycemic control** — MASLD cirrhosis carries high risk of hepatogenous diabetes
- **Optimize micronutrient status** commonly depleted in cirrhosis

**Important:** Protein restriction is *no longer recommended* to prevent HE in cirrhosis. Adequate protein is essential for muscle mass and is protective.

**2. Recommended Daily Protein Intake**

| **Parameter** | **Recommendation** |
| --- | --- |
| **Target protein** | **1.2 – 1.5 g/kg body weight/day** |
| **Daily amount** | **~75 – 93 g protein/day** |
| **Preferred sources** | Plant-based proteins, dairy, eggs, poultry, fish |
| **Protein to limit** | Red meat (high ammoniagenic potential) |

**Rationale for Protein Strategy:**

- **BCAA-enriched or plant protein** is preferred — branched-chain amino acids (leucine, isoleucine, valine) support muscle synthesis and may reduce HE risk
- **Vegetable + dairy proteins** produce less ammonia than red meat (lower aromatic amino acid load)
- Given **prior HE**, monitor tolerance but do **not** restrict protein preemptively — reassess only during active HE episodes

**3. Meal Frequency and Eating Pattern**

This is one of the **most impactful** interventions in cirrhosis nutrition:

Breakfast	Mid-Morning	Lunch	Afternoon	Dinner	Late-Night Snack

**Key Recommendations:**

- **4–6 small meals per day** (rather than 2–3 large meals) to:
  - Minimize gluconeogenesis from muscle protein during fasting intervals
  - Reduce postprandial hyperammonemia
  - Improve glycemic control (critical in MASLD)
- ⭐ **Late-Night Snack (LNS) — Highest Priority Intervention:**
  - Consume a carbohydrate + protein snack **within 1 hour of bedtime**
  - Overnight fast should not exceed **3–4 hours** (vs. 12+ hours in healthy adults)
  - Example: 1 cup of low-fat milk + 2 whole grain crackers, or 1 small banana + Greek yogurt
  - Evidence shows LNS reduces muscle catabolism and improves nitrogen balance in cirrhosis
- **Do not skip breakfast** — begin eating within 1 hour of waking

**4. Specific Food Recommendations and Restrictions**

**Recommended Foods**

| **Category** | **Examples** | **Benefit** |
| --- | --- | --- |
| **Plant proteins** | Tofu, edamame, lentils, chickpeas, beans | Lower ammonia generation, fiber-rich |
| **Dairy proteins** | Greek yogurt, low-fat milk, cottage cheese | BCAA-rich, good tolerability |
| **Eggs** | Whole eggs, egg whites | High-quality protein, leucine-rich |
| **Lean poultry/fish** | Chicken breast, salmon, tilapia | Better protein quality vs. red meat |
| **Complex carbohydrates** | Oats, brown rice, quinoa, sweet potato | Sustained energy, prevents catabolism |
| **Vegetables** | Leafy greens, broccoli, cauliflower | Antioxidants, fiber, low ammonia load |
| **BCAA supplements** | Oral BCAA granules (if dietary intake insufficient) | Muscle synthesis, HE prevention |

**Foods to Limit or Avoid**

| **Food/Substance** | **Reason** |
| --- | --- |
| **Alcohol** | Absolute contraindication — hepatotoxic |
| **Red & processed meats** | High ammoniagenic potential → HE risk |
| **High-fructose foods, sugary drinks** | Worsens MASLD, promotes hepatic steatosis |
| **Refined carbohydrates** (white bread, pastries) | Glycemic dysregulation, insulin resistance |
| **High-sodium foods** (processed/packaged foods) | Promotes ascites and fluid retention |
| **Raw shellfish** | Risk of *Vibrio vulnificus* — fatal in cirrhosis |
| **Large single meals** | Promotes hyperammonemia, glycemic spikes |

**Sodium**

- Target **< 2,000 mg/day** (< 5 g table salt) if ascites is present or at risk
- Avoid adding salt at the table; use herbs, lemon, and spices for flavor

**5. Additional Nutritional Considerations**

**Micronutrient Supplementation (Commonly Deficient in Cirrhosis)**

| **Nutrient** | **Consideration** |
| --- | --- |
| **Zinc** | Often depleted; zinc deficiency contributes to HE; supplement if low (15–45 mg/day elemental zinc) |
| **Vitamin D** | High deficiency risk in elderly + cirrhosis; check 25-OH vitamin D level; supplement accordingly |
| **Magnesium** | Frequently low; important for muscle function and glucose metabolism |
| **Thiamine (B1)** | Monitor, especially if any history of poor intake |
| **Folate & B12** | Common deficiencies in liver disease |
| **Vitamin K** | May be deficient if cholestasis present; relevant to coagulopathy |
| **Fat-soluble vitamins (A, E)** | Check if cholestasis; avoid over-supplementation of Vit A (hepatotoxic in excess) |

**Avoid high-dose Vitamin A supplements** — hepatotoxic in cirrhosis

**Considerations Specific to Elderly Patients (Age 75)**

- **Sarcopenic obesity:** BMI 25.8 may mask significant muscle loss — consider DEXA scan or hand grip strength assessment
- **Appetite decline** and early satiety are common → small, nutrient-dense meals are essential
- **Dysphagia** screening recommended if applicable
- **Polypharmacy interactions** with diet: check lactulose dosing (used for HE), diuretics (electrolyte monitoring), and rifaximin if prescribed

**Hepatic Encephalopathy — Dietary Trigger Prevention**

| **Strategy** | **Detail** |
| --- | --- |
| **Avoid constipation** | Increase dietary fiber (25–35 g/day); ensure adequate fluid intake (~1.5–2 L/day unless fluid restricted) |
| **Limit red meat** | Substitute with plant/dairy protein |
| **Recognize triggers** | GI bleeding, infection, electrolyte disturbance, excessive protein binge — not everyday protein intake |
| **Lactulose compliance** | Dietary counseling should complement (not replace) medical HE management |

**Estimated Daily Caloric Target**

|  |  |
| --- | --- |
| **Target energy intake** | **30–35 kcal/kg/day** |
| **Daily calories** | **~1,860 – 2,170 kcal/day** |
| **Macronutrient distribution** | Protein 15–20% · Carbohydrate 50–55% · Fat 25–30% (favor unsaturated fats) |

**Summary Card**

**Core message for the patient:** *"Eat more often, not less. Don't skip meals or snacks — especially at night. Focus on plant proteins, dairy, eggs, and fish. Avoid alcohol and red meat. A small snack before bed every night is one of the most important things you can do for your liver and muscle health."*
